# Calculation of Five Thermodynamic Molecular Descriptors by Means of a General Computer Algorithm Based on the Group-Additivity Method: Standard Enthalpies of Vaporization, Sublimation and Solvation, and Entropy of Fusion of Ordinary Organic Molecules and Total Phase-Change Entropy of Liquid Crystals

**DOI:** 10.3390/molecules22071059

**Published:** 2017-06-25

**Authors:** Rudolf Naef, William E. Acree

**Affiliations:** 1 Department of Chemistry, University of Basel, Basel 4003, Switzerland; 2 Department of Chemistry, University of North Texas, Denton, TX 76203, USA; acree@unt.edu

**Keywords:** enthalpy of vaporization, enthalpy of sublimation, enthalpy of solvation, entropy of fusion, total phase-change entropy, tpc entropy, group-additivity method

## Abstract

The calculation of the standard enthalpies of vaporization, sublimation and solvation of organic molecules is presented using a common computer algorithm on the basis of a group-additivity method. The same algorithm is also shown to enable the calculation of their entropy of fusion as well as the total phase-change entropy of liquid crystals. The present method is based on the complete breakdown of the molecules into their constituting atoms and their immediate neighbourhood; the respective calculations of the contribution of the atomic groups by means of the Gauss-Seidel fitting method is based on experimental data collected from literature. The feasibility of the calculations for each of the mentioned descriptors was verified by means of a 10-fold cross-validation procedure proving the good to high quality of the predicted values for the three mentioned enthalpies and for the entropy of fusion, whereas the predictive quality for the total phase-change entropy of liquid crystals was poor. The goodness of fit (*Q*^2^) and the standard deviation (σ) of the cross-validation calculations for the five descriptors was as follows: 0.9641 and 4.56 kJ/mol (*N* = 3386 test molecules) for the enthalpy of vaporization, 0.8657 and 11.39 kJ/mol (*N* = 1791) for the enthalpy of sublimation, 0.9546 and 4.34 kJ/mol (*N* = 373) for the enthalpy of solvation, 0.8727 and 17.93 J/mol/K (*N* = 2637) for the entropy of fusion and 0.5804 and 32.79 J/mol/K (*N* = 2643) for the total phase-change entropy of liquid crystals. The large discrepancy between the results of the two closely related entropies is discussed in detail. Molecules for which both the standard enthalpies of vaporization and sublimation were calculable, enabled the estimation of their standard enthalpy of fusion by simple subtraction of the former from the latter enthalpy. For 990 of them the experimental enthalpy-of-fusion values are also known, allowing their comparison with predictions, yielding a correlation coefficient *R*^2^ of 0.6066.

## 1. Introduction

The reliable prediction of certain properties/descriptors of a molecule prior to its synthetic preparation has always been the goal of theoretical and experimental scientists, be it that they wanted to focus their experimental working hours on the synthesis of worthwhile compounds, be it that they wanted to verify their experimental results by means of the predictions. Among the many approaches, from the most elaborate ones such as the time-consuming ab initio methods to the fastest semiempirical self-consistent field procedures, one has turned out to be the most versatile and accurate and is not even quantum-theory-related: the atomic group-additivity method. A recent paper [1] demonstrated its versatility in that it enabled the calculation of mutually totally unrelated descriptors such as heat of combustion, solubility, refractivity, polarizability and toxicity by means of one single computer algorithm. This approach marks the endpoint, so to speak, of the various earlier group-additivity methods focusing on specific fields of application such as the prediction of the logP_O/W_ values [2,3], the molar refractivity [4], the molecular polarizability [5,6], or—closer to the present goal—the “simultaneous” evaluation of the logP, the aqueous solubility and the brain/blood distribution ratio logBB using individual parameter sets [7]. It is no secret, however, that the unsuccessful attempts in paper [1] to reliably predict just the latter descriptor, logBB, put a damper on the expectation of a universal applicability of the present atomic group-additivity method. Yet, the exceptionally high prediction quality for the heat of combustion values across the entire structural spectrum of compounds presented in paper [1]—showing a cross-validated correlation coefficient of better than 0.9999 for 1965 compounds—at least gave rise to the hope that this method might successfully be extended to further thermodynamic descriptors.

The standard enthalpies of vaporization and sublimation were the first targets to be examined, not only because of their importance in chemical and environmental science, but also because a great deal of groundwork had already been done by Acree, Jr. and Chickos [8], who collected a large number of experimental vaporization and sublimation data covering more than a century. Several attempts to estimate the standard enthalpies of vaporization and sublimation have already been published: Roux et al. [9] evaluated the standard phase-change enthalpies of molecules from their experimental phase-change enthalpies at any given temperatures using their estimated heat capacity at room temperature. In cases where the number of experimental data was insufficient, they extrapolated the data from compounds with known experimental values. This estimation method, however, was limited to the vaporization enthalpy of liquid hydrocarbons. Similarly, Chickos et al. [10,11] estimated the vaporization enthalpies of larger even-numbered linear *n*-alkanes from a series of smaller ones [12,13] using their temperature dependence of the gas chromatographic retention time. A further indication of the potential applicability of the group-additivity method to predict the heats of vaporization and sublimation was found in the high correlation of the chain length of the homologues of saturated and unsaturated fatty acids with their experimental values [14].

Determination of the enthalpy of solvation has recently been based on the Abraham solute parameters model [15,16,17,18], the model consisting of a linear equation of five parameters relating to the molecule’s excess molar refraction, the polarity/dipolarity, solute hydrogen-bond acidity and hydrogen-bond basicity, and the McGowan (i.e., molecular) volume. These parameters have been derived from the molecular structure of a series of compounds using multilinear regression analysis and artificial neural networks [19]. Earlier, Cabani et al. [20] described a group-contribution method for the estimation of the enthalpy, Gibbs free energy and heat capacity of liquids of non-ionic solutes in water, limiting the method for the calculation of the group contributions to compounds with not more than one heteroatom and then applying correction parameters for molecules containing more than one heteroatom.

The entropy of fusion (often—and more logically—called entropy of phase change or even better: entropy of melting) of ordinary organic molecules as well as its special manifestation with liquid crystals, called total phase-change entropy, generally mean the entropy of the transition of a molecule from its most stable crystalline form to the isotropic melt. While for ordinary molecules this transition in most cases occurs in one step or two consecutive steps upon addition of thermal energy, this process is much more complex with liquid crystals in that they know several intermediate, semi-crystalline phases melting at considerably different temperatures. In the first case, occurrence of more than one melting step may be explained by polymorphism of the crystalline form, their various polymorphic forms often showing distinct differences in their fusion enthalpies. In the second, the various semi-crystalline forms can be stable over a considerable temperature range, thus consuming a large amount of thermal energy prior to their next phase change. The thermodynamic consequences of the difference in the melting processes between ordinary molecules and compounds exhibiting liquid crystal properties forced Chickos et al. [21] and Acree, Jr. et al. [22] to treat these two categories of compounds as separate entities in their collective volumes.

The present work, being a continuation of the principle to calculate the molecular descriptors published earlier [1], will show the extendability of the approach to reliably predict the enthalpies of vaporization, sublimation and solvation, as well as the entropy of fusion. In order to clearly distinguish the phase-change entropy of ordinary compounds from that of liquid crystals, the term “entropy of fusion” will remain reserved for the former, while for the latter the well-established term “total phase-change entropy” will be used throughout.

## 2. General Procedure 

All the calculations are based on a knowledge database encompassing at present more than 28,500 records, containing the compounds in their geometry-optimized 3D form and carrying all the required (and several more) data. The database includes—besides ordinary organic molecules—organic salts, ionic liquids, liquid crystals and metal-organic compounds.

The algorithm for the calculation of the present descriptors follows the atom-group additivity principle outlined in detail in the earlier paper [1]. Consequently, the naming and meaning of the atom groups in the parameters tables is the same, the tables being complemented by further atom groups, where necessary, following the rules described in Table 1 of [1]. The results of the evaluation of the atom-group contributions are stored in a separate parameters list for each descriptor. The only difference to the earlier work lies in the addition of a further special group as a consequence of attempts to optimize calculations of the group contributions for the entropies of fusion, where it turned out that the difference between the experimental values of open-chained and cyclical compounds was not resolvable by the given ordinary atom groups themselves. Therefore, a special group called “Endocyclic bonds” has been introduced which counts the number of endocyclic bonds in a molecule but is restricted to single bonds to take account of their reduced freedom of mobility within a ring system (bonds of higher order are by themselves restricted). Its treatment within the calculation is identical to the one described for all the other special groups.

Once the group contributions have been evaluated as described earlier, the prediction of the descriptors follows the general Equation (1), where *a_i_* and *b_j_* are the contributions, *A_i_* is the number of occurrences of the *i*th atom group, *B_j_* is the number of occurrences of the special groups and *C* is a constant:

(1) $$Y=\sum_{i}a_{i}A_{i}+\sum_{j}b_{j}B_{j}+C$$

It is immediately evident that this equation excludes prediction of descriptors for molecules for which not all atom groups are present in the corresponding parameters table. Yet, a further limitation is given by the condition that only atom groups are valid for consideration that have been represented by at least three independent molecules in the parameters-evaluation process. The number of molecules representing a given atom group is listed in the rightmost column of the parameters tables shown below. The remaining atom groups represented by less than three molecules are kept in the parameters tables solely for future use in this continuing project (and to invite researchers experimenting in these areas to focus on compounds carrying these atom groups). The calculations are generally restricted to molecules containing the elements H, B, C, N, O, P, S, Si and/or halogen.

Plausibility tests have been carried out for each of the atom-group additivity parameters evaluations applying a 10-fold cross-validation procedure as described in [1], making sure that each compound has been used once as a test sample in the process. The results of these calculations are condensed in row A to H at the end of each parameters table. In the corresponding correlation diagrams (Figure 7) and histograms presented below the results of the cross-validation calculations are superpositioned in red over the training data.

## 3. Results 

### 3.1. General Remarks

(1) The experimental values of enthalpies and entropies are temperature-dependent. Any relationship within these properties or with other ones only make sense if they are referenced to the same temperature. The usual temperature of reference is 298.15 K, and thus it was ensured in this work that experimental data from literature were only accepted if they had been either measured at or adjusted to the standard temperatur of 298.15 K and standard pressure of 100 kPa.
(2) All lists of molecules used in the atom-group parameters evaluations have been collected in standard SDF files, stored in the supplementary material, ready to be imported by external chemistry software. The supplementary material also provides the lists of results containing molecule names, experimental, training and cross-validation values. Beyond this, it also contains lists of experimental outliers.

### 3.2. Enthalpy of Vaporization

Experimental data of vaporization enthalpies have essentially been extracted for this work from the large collection of Acree, Jr. and Chickos [8] and Chickos et al. [10,11,12,13,14], supplemented by recent data from a number of further authors publishing experimental vaporization values of several acetophenones [23], aliphatic tertiary amines [24], azidomethyl-*N*-nitrooxazolidines [25], benzamides [26], benzocaine [27], bisabolol and menthol [28], crown ethers [29], *N*,*N*-dialkyl monoamides [30], fenpropidin and phencyclidine [31], flavors [32], long-chain fluorinated alcohols [33], whiskey- and metha-lactone [34], halogenated fluorenes [35], ibuprofen and naproxen [36], imidazo[1,2-*a*]pyrazine and phthalazine [37], insect pheromones [38], morpholines [39], organo(thio)phosphates [40], dialkyl phthalates [41], nitrogen heteroaromatics [42], phenylimidazoles [43], 2-acetylthiophene [44], dicarboxylic *n*-pentyl esters [45], and cyclic amines, ethers and alcohols [46]. The result of the atom-group parameters, based on 3581 compounds, is summarized in Table 1. Several tentative calculations with or without inclusion of certain special groups outlined in Table 2 of the earlier paper [1] revealed a minor improvement of the goodness of fit upon inclusion of the “atom group” responsible for intramolecular acid-base bonds, named “H/H Acceptor”, as well as of those reserved for saturated and unsaturated pure hydrocarbons, called “Alkane/No. of C atoms” and “Unsaturated HC/No. of C atoms”, which add a correction value for each carbon atom.

The total number of atom groups in Table 1, required to take account of the complete set of 3581 molecules for which experimental vaporization data are known, is 302. However, the condition to restrict their applicability to those resting on at least three independent molecules, reduces the number of “valid groups” to 187, as is shown in row A of Table 1. Accordingly, the number of compounds viable for the evaluation of the result of the complete training set and of the test sets in the 10-fold cross-validation calculation was reduced to 3460 and 3381, respectively, as listed in the right-most column. The high correlation coefficients *R*^2^ and *Q*^2^ of the training and the cross-validated sets (rows B and F) of better than 0.96 and the small difference between them is clear proof of the viability of the present group-additivity model for the prediction of the enthalpy of vaporization. Furthermore, the small standard deviations for the training and test sets of 4.3 and 4.56 (rows D and H) also speaks for the model’s accuracy. In order to put these deviations into perspective with the reality of the experimental practice, a few examples should be given for comparison: the compilation of Acree and Chickos [8] presented eight values for 1-butanol ranging from 48.4 to 55.2 kJ/mol, seven values for methyl *t*-amyl ether ranging from 33.5 to 35.8 kJ/mol, and four values for ethylenediamine of between 41 and 54.4 kJ/mol. It goes without saying, therefore, that the standard errors of the group-parameters calculations (lines D and H in the parameters table), covering the complete set of available data, are always larger than the individual errors and, thus better reflect the general uncertainty of the experimental data.

**Figure 1:**
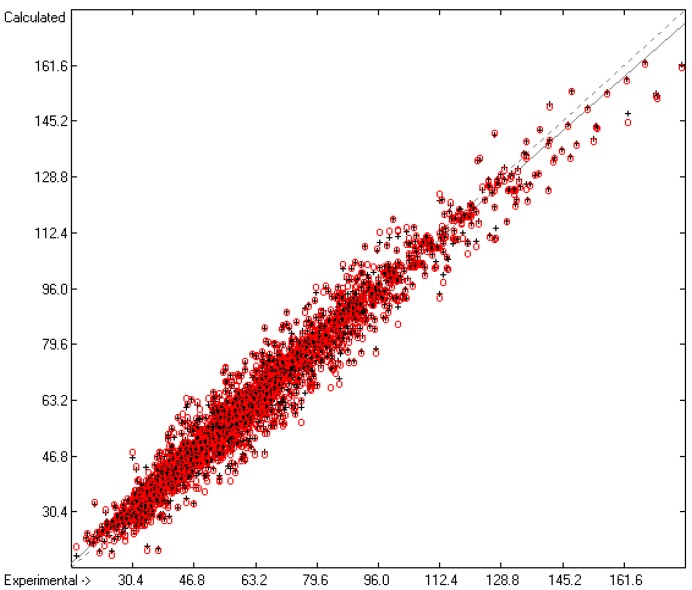
Correlation Diagram of the Enthalpy-of-Vaporization Data (*N* = 3460; *R*^2^ = 0.9677; *Q*^2^ = 0.9640; regression line: intercept = 1.9756, slope = 0.9681).

**Figure 2:**
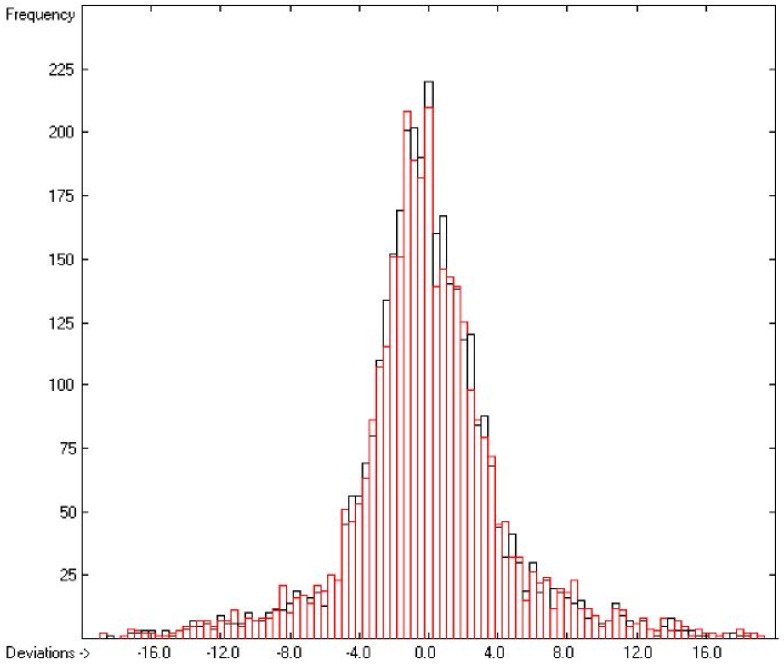
Histogram of the Enthalpy-of-Vaporization Data (*S* = 4.56 kJ/mol; Exp. values range: 15.6–177.2 kJ/mol).

The correlation diagram in Figure 1, showing a fairly even distribution of the vaporization data along the regression line, also reveals a narrow overlap of the cross-validated test data with those of the training set. The related histogram in Figure 2, exhibiting a nearly perfect Gaussian bell curve, proves the evenness of the distribution of the deviations of both test and training data about the regression line. The analysis of the distribution of the deviations yielded the following result: 79.2% of the presently 3460 tested compounds deviated by less than or equal to one cross-validated standard error of 4.56 kJ/mol, whereas 6.8% exceeded a deviation of twice that standard error. Beyond this, 32 molecules had to be viewed as outliers as their deviation surpassed by at least four times this standard deviation. 

Despite the detailed distinction of the atom groups in Table 1, resulting in an extended list of groups of which about one third is “invalid”, the still large number of “valid” atom groups enabled the calculation of reliable enthalpy-of-vaporization data for 78.2% of the complete set of compounds in the database.

### 3.3. Enthalpy of Sublimation

The enthalpy of sublimation is the sum of the enthalpies of vaporization and fusion, provided that all of them are referenced to the same temperature. This precondition has been thoroughly followed when selecting experimental data from literature. Again, as in the previous section, the main contribution of experimental sublimation values has been provided by the compendium of Acree, Jr. et al. [8], supplemented by a number of later publications, referencing the heat of sublimation of acetophenones [23], substituted benzamides [26], crown ethers [29], long-chain fluorinated alcohols [33], halogenated fluorenes [35], tricyclic nitrogen heteroaromatics [42], polyphenylbenzenes [47], adamantylideneadamantane [48], cyclic *N*,*N*′-thioureas [49], indole-3-carboxylic acids [50], vanillyl alcohol [51], alkanoylphenols [52], adamantanes [53], six-membered ring aliphatics [54], fluoroquinolones [55], oxazolidinones [56], nitrogen-containing substituted adamantanes [57], 2,7-di-*t*-butylfluorene [58] and nitroimidazoles [59].

The correlation coefficients *R*^2^ and *Q*^2^ (rows B and F at the bottom of Table 2) exhibit a higher scatter of the experimental data in comparison with the heat-of-vaporization data. The increased uncertainty might be partly ascribed to the fact that in many cases molecules form several crystal structures at different temperatures, having different enthalpies of fusion, and that, therefore, the starting point of the measurements is not clearly defined. In other cases the molecules may not be completely crystalline due to impurities. Another reason may be that while many of the compounds in the enthalpy of the vaporization dataset of the previous chapter are liquid at ambient room temperature and the vaporization measurements have been performed at temperatures not too far removed from 298.15 K, requiring only a small correction back to this reference temperature, the enthalpy of sublimation measurements, on the other hand, are often carried out at higher temperatures where the compounds are more volatile. In these cases, the uncertainty in the correction term needed to extrapolate the experimental value back to the reference temperature is higher and increases with the difference between the experimental and the reference temperature. The consequences of these uncertainties are reflected in the spread of experimental data originating from different authors for the same compounds: for example, for the enthalpy of sublimation of anthracene, there are seven values given in the 2010 Acree and Chickos compilation [8] that range from 88.3 to 93.3 kJ/mol, and for coumarin there are two values for the same property that range from 83.1 to 95.4 kJ/mol.

**Figure 3:**
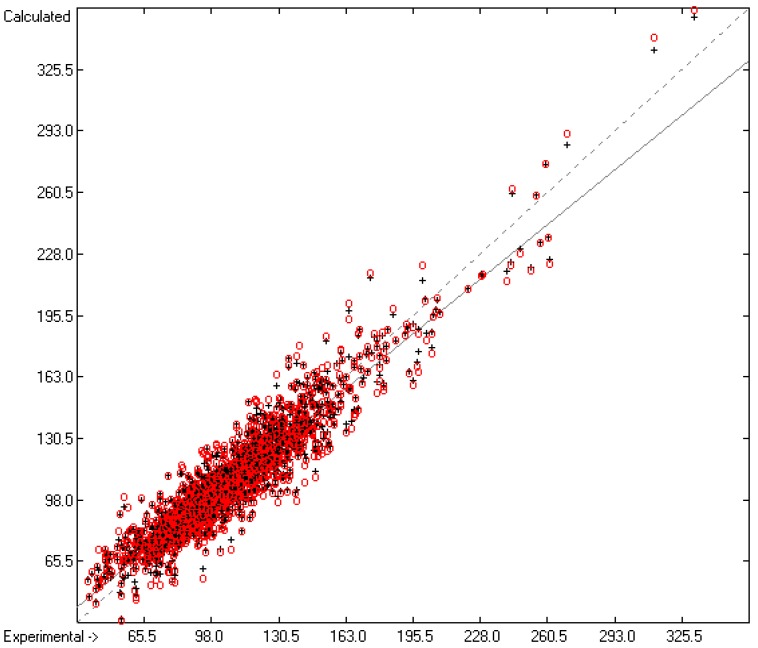
Correlation Diagram of the Enthalpy-of-Sublimation Data (*N* = 1866; *R*^2^ = 0.8887; *Q*^2^ = 0.8657; regression line: intercept = 12.0233, slope = 0.8884).

**Figure 4:**
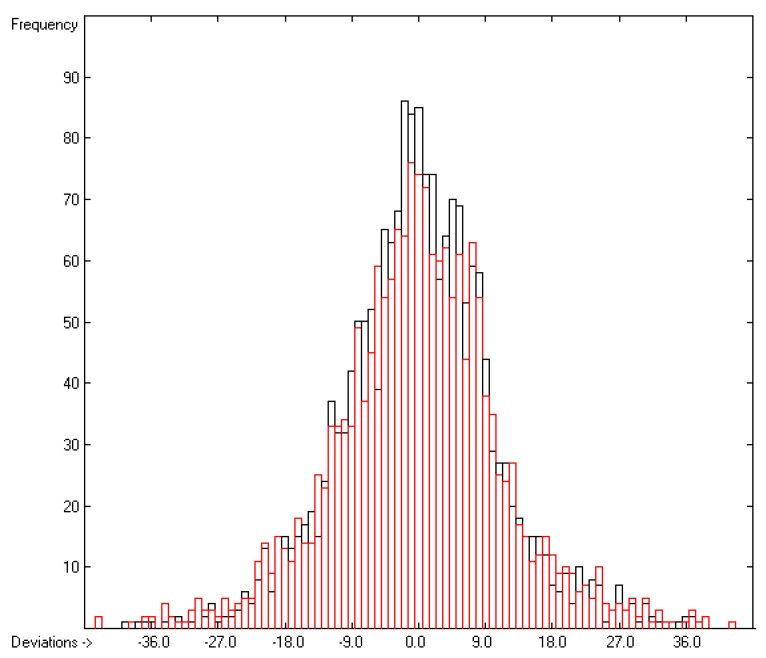
Histogram of the Enthalpy-of-Sublimation Data (*S* = 11.39 kJ/mol; Exp. values range: 38.7–331.88 kJ/mol).

Figure 3 demonstrates the larger scatter of the data about the regression line, leading to a cross-validated standard deviation of 11.39 (see row H in Table 2), i.e., 2.5 times larger than for the heat of vaporization. Figure 4 visualizes the error distribution, showing that, according to an analysis, 74% of the molecules’ predicted values differ by less one cv-standard deviation and only 5.6% by more than twice that amount. Only 16 compunds had to be declared as outliers because their experimental value exceeded four times the cv-standard deviation. One compound, norcamphor, had to be excluded from calculation because its experimental enthalpy of sublimation was lower than its experimental enthalpy of vaporization, an obviously impossible finding.

The lower number of “valid” atom groups of 154, as shown in row A of Table 2, led to the slightly reduced amount of 75.9% of the molecules in the representative database for which the heat of sublimation was calculable.

### 3.4. Enthalpy of Fusion

It seems obvious to try to apply the atom-group additivity method as described in the preceding chapters for the prediction of the enthalpy of fusion, all the more as several authors [21,60] have already used this principle very successfully. However, since the presented predictions of the enthalpies of sublimation and vaporization rest exclusively on experimental values at 298 K, it is legitimate to refer to the simple Equation (2) which defines a molecule’s enthalpy of fusion at standard conditions as the difference between its enthalpy of sublimation and its enthalpy of vaporization:

Δ*H*°_fus_(298 K) calc. = Δ*H*°_sub_(298 K) calc. − Δ*H*°_vap_(298 K) calc. (2)

Accordingly, the standard deviation of the thus evaluated enthalpy of fusion can be calculated by means of the error-propagation equation for the sum of two cross-validation standard errors *Q*^2^(Δ*H*°_sub_) (=11.39 kJ/mol) and *Q*^2^(Δ*H*°_vap_) (=4.56 kJ/mol), resulting in a standard deviation σ for the calculated enthalpy of fusion of 12.27 kJ/mol. Evidently, this deviation is largely dominated by the uncertainty of the experimental heats of sublimation and, thus would gain the most upon the provision of more accurate sublimation data.

**Figure 5:**
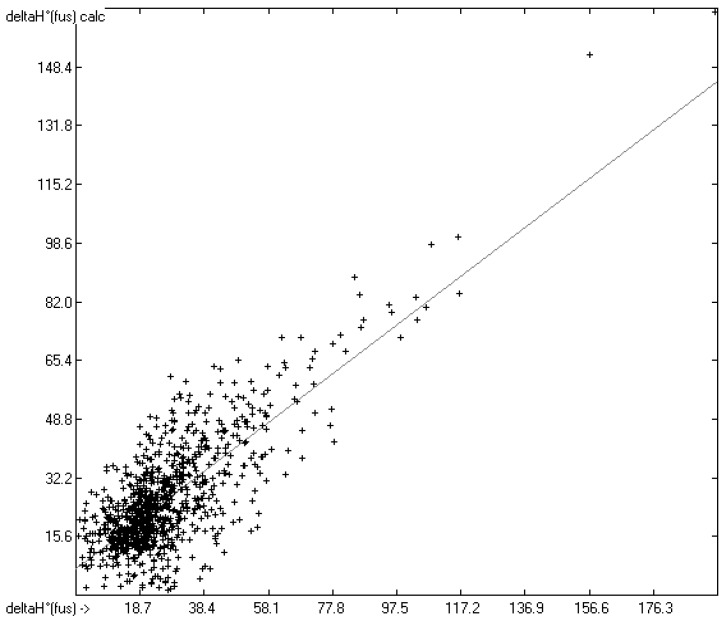
Correlation Diagram of the Enthalpy-of-Fusion Data (*N* = 990; *R*^2^ = 0.6066; calculated values evaluated by means of Equation (2)).

**Figure 6:**
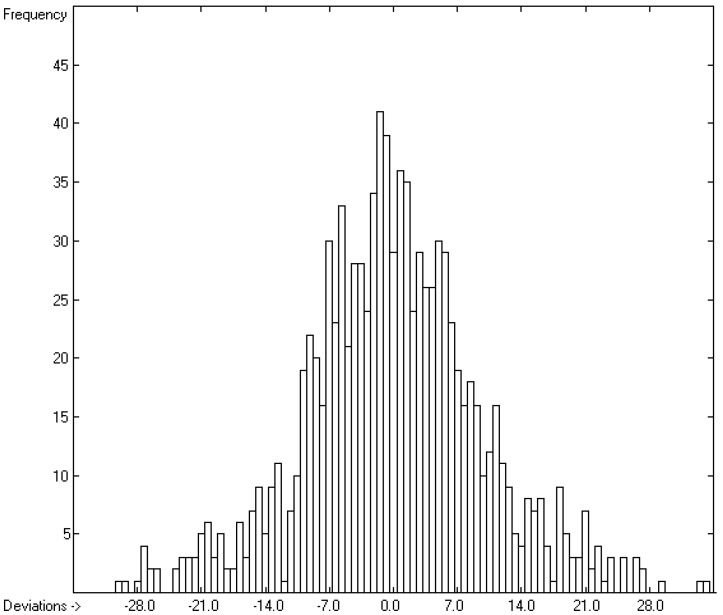
Histogram of the Enthalpy-of-Fusion Data (*S* = 9.78 kJ/mol; Exp. values range: 0.30–164 kJ/mol).

How well do the predictions of Equation (2) compare to experimental heat-of-fusion data? In order to answer this question more than 1200 experimental values have been inserted into the database, taken from Acree’s compendium publication [8], complemented by recent values for crown ethers [29], fluorinated alcohols [33], adamantanes [53], 2-chloro-3-(trifluoromethyl)pyridine [61], cyanatophenyl derivatives [62], diphenylamines [63], fatty acids [64], pyridinecarbothioamides [65], isoniazid [66] and phenylthiazole-thione [67]. Figure 5 shows a comparison of the experimental with the predicted values, independently calculated by means of Equation (2). After removal of the worst 28 outliers the correlation coefficient *R*^2^ for the remaining 990 samples (for which both the experimental and predicted values were available) was calculated to 0.60. This rather low value is at least in part explicable by findings outlined in several papers revealing that for certain compounds experimental values originating from different authors often scatter over a large range. For instance, Eckert et al. [64] graphically demonstrated for various fatty acids that the value of their enthalpy of fusion varied drastically over a period of up to 80 years of repeated examination. Some examples: the enthalpy of fusion for palmitic acid randomly varied over the years between ca. 41 and 60 kJ/mol, and for stearic acid the range, varying between ca. 45 and 74 kJ/mol, was even wider. Analogous observations were made by Leitner and Jurik [68], who discovered similar discrepancies by different authors also for small molecules, exemplified by paracetamol and aspirin, for which the published values varied between 26 and 34.1, and between 29.89 and 32.92 kJ/mol, respectively. Figure 5, also demonstrating that the overwhelming number of experimental data is concentrated in the narrow range of below 40 kJ/mol, provides another explanation for the difficulty to enable exact predictions. The related histogram in Figure 6 nevertheless proves a satisfyingly even distribution of the deviations about the regression line drawn in Figure 5. Thanks to the broad applicability of the “valid” number of atom-group parameters for both the heat of sublimation as well as the heat of vaporization, Equation (2) enabled the estimation of the heat of fusion of 68% of the database’s molecules.

### 3.5. Enthalpy of Solvation

Literature referencing experimental enthalpy-of-solvation data is relatively scarce. The most yielding source was found in Mintz et al.’s [69] paper on the application of the Abraham model mentioned earlier on gaseous solutes dissolved in water and 1-octanol. Further studies were made on *N*-methylimidazole [70], urea and its derivatives [71,72,73], thiourea and its derivatives [74], carboxamides and their *N*-substituted derivatives [75,76,77,78], and uracil and its alkyl-, amino-, nitro- and halosubstituted derivatives [79,80,81,82]. Of the accordingly limited number of 465 compounds having experimental enthalpy-of-solvation values for water as solvent in their datalist, 436 have been entered into the calculation of the atom-group parameters, resulting in 61 valid groups allowing the evaluation of the cross-validated prediction of the solvation enthalpy of 373 compounds with a cv-goodness of fit of 0.9546 and a corresponding standard deviation *Q*^2^ of 4.34 kJ/mol as is shown in aggregated manner in Table 3. 

**Figure 7:**
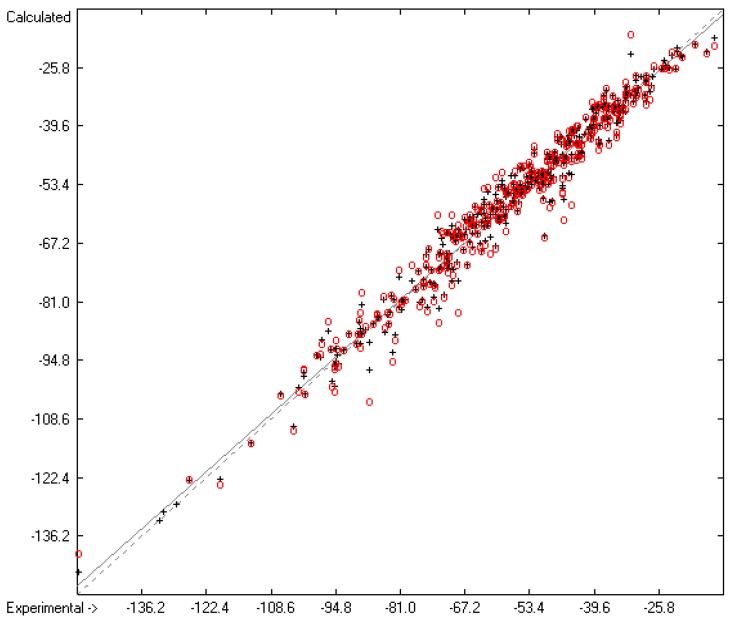
Correlation Diagram of the Enthalpy-of-Solvation Data (*N* = 388; *R*^2^ = 0.9731; *Q*^2^ = 0.9546; regression line: intercept = −1.4422, slope = 0.9759).

**Figure 8:**
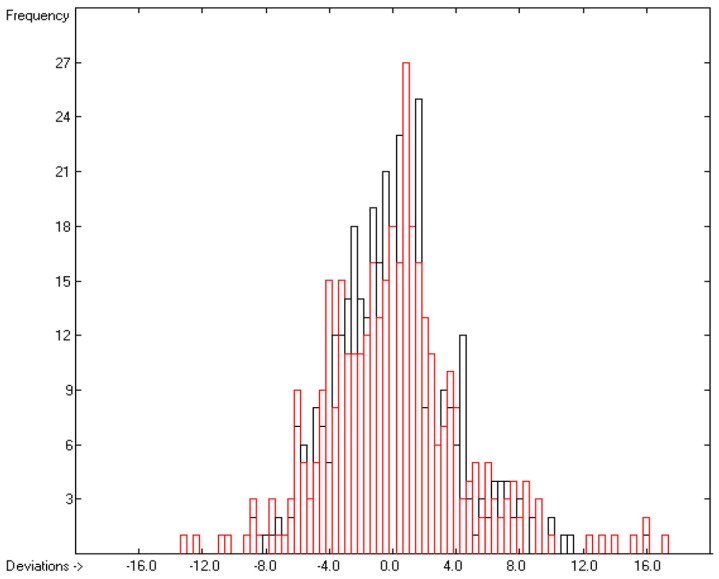
Histogram of the Enthalpy-of-Solvation Data (*S* = 4.34 kJ/mol; exp. values range: −149.51–−13.7 kJ/mol).

Due to the limited number of compounds, the histogram in Figure 7 and Figure 8 reveals a slightly distorted Gaussian bell form. Nevertheless, the analysis of the error distribution reveals that 78.8% of the compounds deviated by less than one cv-standard deviation, whereas for only 5% the deviation was larger than twice this value. The small number of only 61 valid atom groups limited the range of compounds in the database eligible for a heat-of-solvation prediction to 40%. As an informational note, the Abraham model used by Mintz et al. [69] described the enthalpy-of-solvation data of the 369 compounds in their data set to within a standard deviation of 4.04 kJ/mol, which is slightly larger than our standard deviation of 3.53 kJ/mol based on a data set of 388 compounds. Beyond this, of the thermodynamic properties considered here and in the previous paper [1], the Abraham model can only predict enthalpies of solvation.

The observant reader may have noticed that the goodness of fit of the heat-of-solvation calculation is better than that for the heat of sublimation, although the experimental source for the former is the difference between the heat of solution and the heat of sublimation (or vaporization). Hence, one would expect that the uncertainty of the heat of sublimation would be reflected in the goodness of fit of the heat of solvation. The reason as to why this is not the case lies in the nature of the experimental measurements which reduces the chemical diversity: while the determination of the heat of sublimation in principle allows molecules of nearly any size and complexity, the solvation experiments are limited to mostly simple organic liquids and solids having only one, two or three functional groups because these molecules had to exhibit sufficient solubility in water to enable the measurement and they had to readily dissolve within a reasonable amount of time. This precondition eliminated compounds with poor water solubility, in other words many of the larger species. These limitations are also visible in the scope of the experimental enthalpy values: while the range for the heat of sublimation is between about 30 and 330 kJ/mol, for the heat of solvation it is only between about −12 and −150 kJ/mol, i.e., much smaller. It is reasonable to presume that if the solvation experiments would include structurally complex compounds, the correlation deviations would be larger. This size limitation has also a negative effect on the diversity of the atom groups, as can be seen in Table 3, row A, where the number of “valid” groups, available for the calculation of the heat of solvation, is only 61 in relation to 154 (see Table 2, row A) for the heat of sublimation.

### 3.6. Entropy of Fusion

The entropy of fusion under this subtitle is defined as the entropy change associated with the phase change from the crystalline to the isotropic liquid state of a molecule without passing any intermediate anisotropic, semiliquid phases. In most cases this transition indeed occurs in one stage, but several molecules, on addition of heat, undergo a change from one crystalline phase to a second or even third energetically less stable phase prior to melting. In the following, the entropy-of-fusion values cited in the tables are the sum of all the entropies associated with these solid-solid phase changes including the final solid-liquid phase change. The main source for these values was found in the comprehensive collection of Chickos, Acree and Liebman [21] and in its update [60]. More recent entropy-of-fusion data were found for long-chain fluorinated alcohols [33], halogenated fluorenes [35], di- and tri(cyanatophenyl)alkanes and -silanes [62], 2-cyano-4′-methylbiphenyl [83], diphenyl cyclohexyl-phosphoramidate [84] and 3,4-dinitrofurazanfuroxan [85]. The complete set of compounds with experimental entropy-of-fusion values amounted to a total of 2809 used for the evaluation of the atom-group parameters, yielding 188 valid atom groups. Various tentative calculations including or excluding certain special groups revealed a distinct improvement of the goodness of fit of the optimization process, if the group “Endocyclic bonds” was involved, which counts all single endocyclic bonds in a molecule. However, for small molecules containing small rings this group parameter tended to overcompensate the decrease of freedom of mobility and, therefore, the three special groups “Angle60”, “Angle90” and “Angle102” were added as counter-correctives. The cross-validation calculation with 2637 samples resulted in a very satisfying goodness of fit *Q*^2^ of 0.8727 and a standard deviation of 17.93 J/mol/K. In Table 4 the results of these calculations are summarized. Fifty-five compounds had to be removed from the calculations as their experimental values deviated from prediction by more than three times the cv-standard deviation. The large number of valid atom groups, on the other hand, enabled the calculation of the entropy of fusion for 81.8% of the database’s compounds.

**Figure 9:**
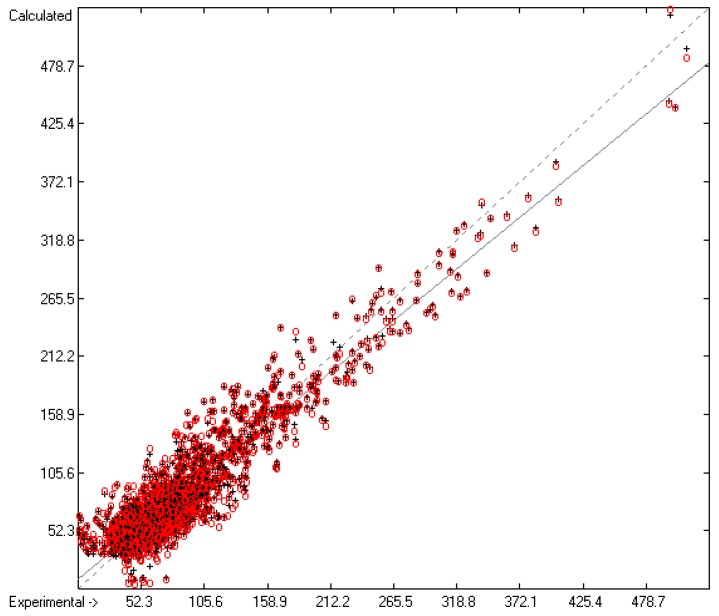
Correlation Diagram of the Entropy-of-Fusion Data (*N* = 2701; *R*^2^ = 0.8874; *Q*^2^ = 0.8727; regression line: intercept = 8.6540; slope = 0.8883).

**Figure 10:**
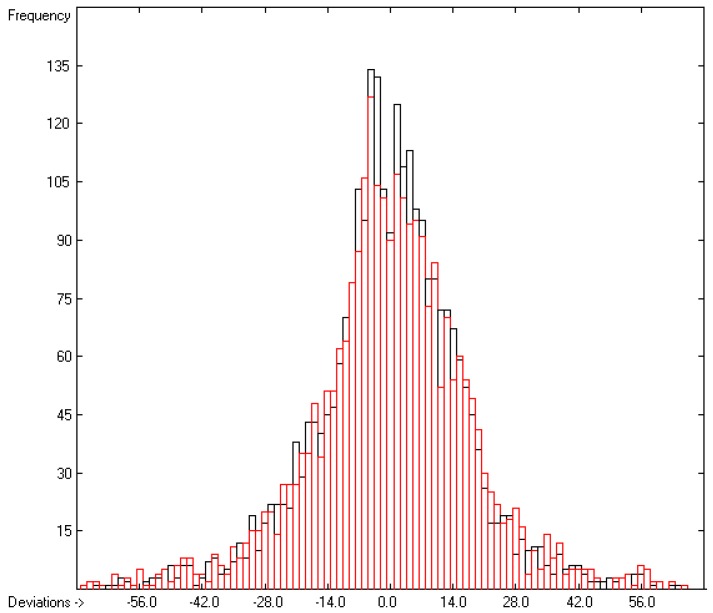
Histogram of the Entropy-of-Fusion Data (*S* = 17.93 J/mol/K; Exp. values range: 0.65–513.5 J/mol/K).

The correlation diagram in Figure 9 exhibits a large concentration of the entropy values in the range between 0 and ca. 140 J/mol/K; values of 200 J/mol/K or more are exclusively reserved for molecules carrying long, mostly un-branched methylene or poly-ether chains. The histogram (Figure 10) reveals a slight overweight of the positive deviations, indicating a minor trend to predict too low values.

### 3.7. Total Phase-Change Entropy of Liquid Crystals

Liquid crystals are a class of molecules characterized by the special feature to often exhibit several distinct semiliquid states between their crystalline and isotropic liquid phases, i.e., anisotropic phases which are stable over an extended temperature range. Depending on their intermediate structure these phases are either called meso, cholesteric, smectic or nematic. This strange self-associative behaviour has typically been found with compounds the molecular structure of which contains rigid moieties and highly flexible pendant alkyl or polyether chains of various length, but also with molecules where certain parts exhibit strong intermolecular hydrogen bonds besides moieties of intermolecular inertness. Due to the variability of their entire melting processes resulting from their structural characteristics, the only common entropy term to possibly be generally applicable is the total phase-change entropy, defined as “the sum of all the entropy changes associated with phase transitions occurring from *T* = 0 K to the clearing temperature, *T* = *T*_iso_.” [22]. This definition only differs from the one given for the entropy of fusion in the previous chapter, in that here not only the potential solid–solid entropy-phase changes but also the entropy changes of the semiliquid intermediate phases are considered. Based on this definition, only the total phase-change entropy data of liquid crystals have been entered into the evaluation of the related atom-group parameters. The only source for these data was the large collection of more than 3000 compounds in the compendium work of Acree, Jr. and Chickos [22]. The parameters calculation finally rested on 2686 compounds, yielding a direct goodness of fit *R*^2^ of 0.6094 and a cross-validated goodness *Q*^2^ of 0.5804 with a standard deviation of 32.79, as condensed at the bottom of Table 5. (In order to compare these data directly with those of the entropy-of-fusion calculation, the special groups “Angle60” and “Angle90” are kept in the parameters list although obviously no compound met any of these two criteria, i.e., bond angle ≤90 or <60.) These data compare favourably with those of Acree and Chickos [22], who reported a correlation coefficient of only 0.35 for 627 liquid crystals. The present results, however, required the removal of 56 compounds from the evaluation of the parameters, as their deviation from prediction was much larger than three times the cv-standard deviation.

**Figure 11:**
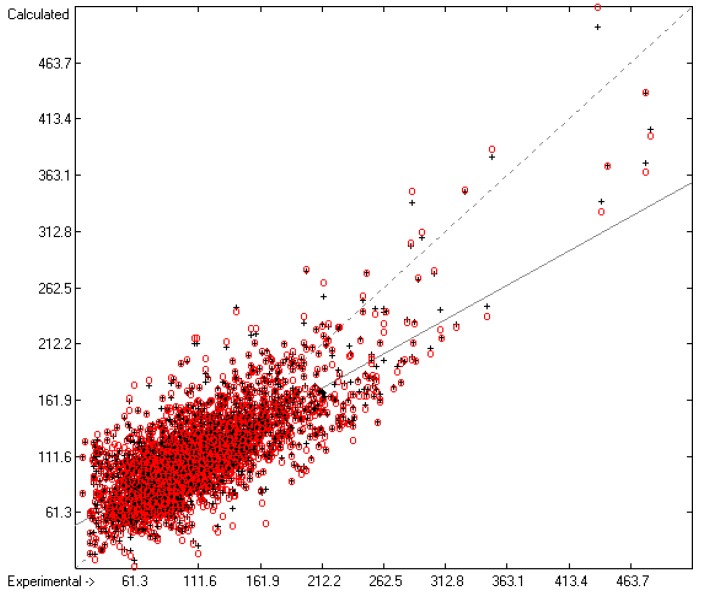
Correlation Diagram of the Total Phase-change Entropy Data (*N* = 2663; *R*^2^ = 0.6091; *Q*^2^ = 0.5804; regression line: intercept = 43.5325, slope = 0.6083).

**Figure 12:**
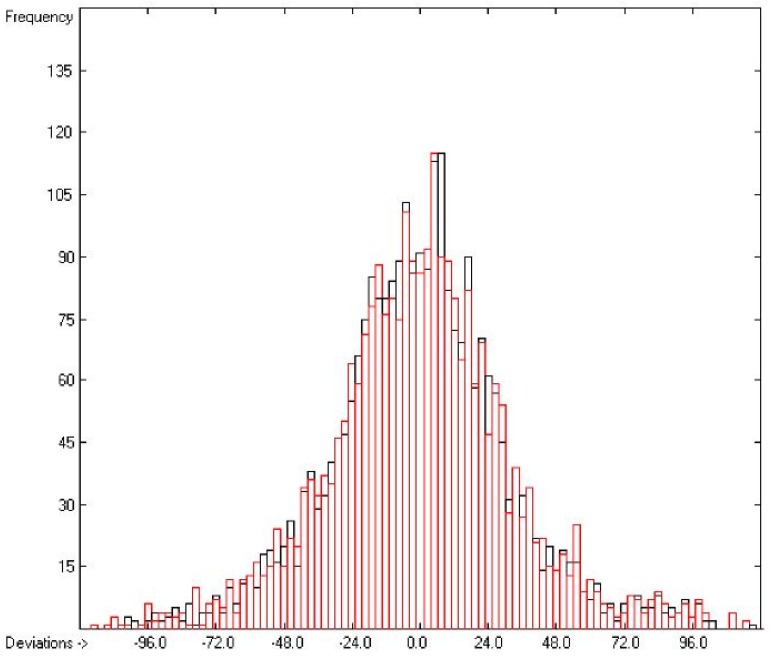
Histogram of the Total Phase-change Entropy Data (*S* = 32.79 J/mol/K; Exp. values range: 17.6–480.76 J/mol/K).

Nevertheless, it was to be expected that the additional entropy terms relating to the semiliquid phases would blur the picture in comparison with the previous chapter, since not only each homologous series of liquid crystals but even individual molecules proceed via different melting pathways. This feature is even observable in the list of outliers where several entire homologous series had to be removed. As a consequence of this inhomogeneity, the scatter of the total phase-change entropy of the liquid crystals in Figure 11 is extraordinarily high, but, as the histogram in Figure 12 shows, is evenly distributed about the regression line. This, and the close similarities of *R*^2^ and *Q*^2^ as well as of the direct and the cross-validated standard deviations, collected at the bottom of Table 5, may lead to the assumption that the associated atom-group parameters are reliable enough for phase-change entropy predictions within the class of liquid crystals. Two homologous examples may prove whether this assumption is justified.

**Figure 13:**
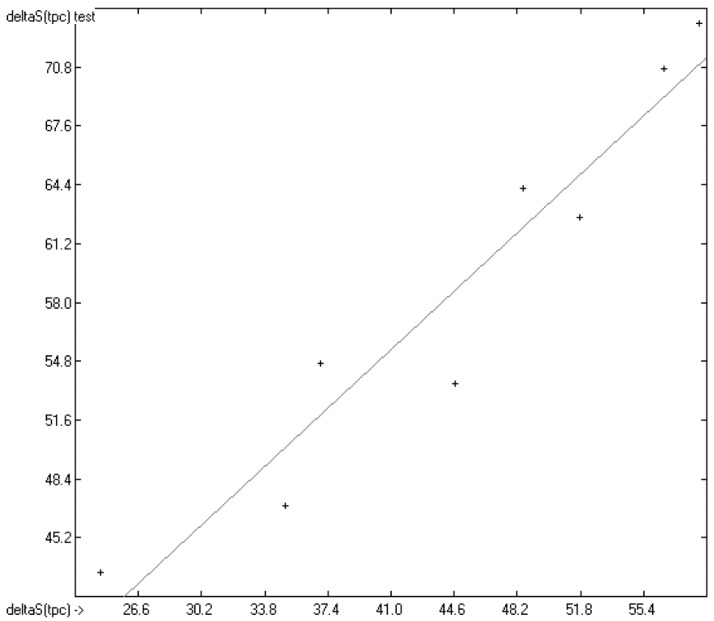
Correlation Diagram of the Total Phase-change Entropy Data of the homologues of 7-alkyl-2-(4-cyanophenyl)-fluorenes. (*N* = 8; *R*^2^ = 0.9176; σ = 2.90 J/mol/K).

**Figure 14:**
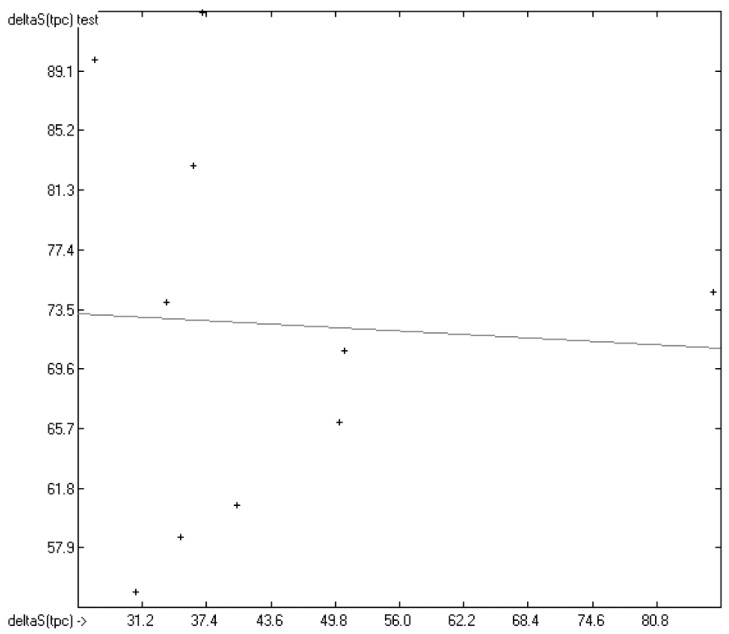
Correlation Diagram of the Total Phase-change Entropy Data of the homologues of 3-(4-alkyloxyphenylamino)-1-(2-(5-cyanothienyl))-2-propen-1-one. (*N* = 10; *R*^2^ = 0.0023; σ = 12.29 J/mol/K).

In Figure 13, the experimental total phase-change entropy data of the liquid-crystal homologues of 7-alkyl-2-(4-cyanophenyl)-fluorene (with alkyl = ethyl, propyl, butyl, pentyl, hexyl, heptyl, octyl and nonyl) are correlated with predicted values, revealing an excellent correlation coefficient *R*^2^ of 0.9176. The slope of the regression line, however, is at 0.8830 considerably lower than 1.0. Figure 14 shows the analogous correlation of the homologues of 3-(4-alkyloxyphenylamino)-1-(2-(5-cyanothienyl))-2-propen-1-one (with alkyl = pentyl, hexyl, heptyl, octyl, nonyl, decyl, undecyl, dodecyl, tridecyl and tetradecyl). Here, the correlation coefficient has been calculated to 0.0023 and the slope of the regression line is even slightly negative at −0.0364. These examples prove that the class of liquid crystals is too heterogeneous for the present atom-group additivity model to be applicable. Consequently, if even within the class of liquid crystals reliable predictions are impossible, attempts to do so outside this class would not make sense at all.

## 4. Conclusions

The application of a computer algorithm described in detail in an earlier paper [1], based on the atom-group additivity principle to calculate reliable values of the heat of combustion (and indirectly-formation), logP_o/w_, logS, refractivity, polarizability and toxicity, has successfully been extended to the prediction of the heats of vaporization, sublimation and solvation, and the entropy of fusion of ordinary molecules as well as the total phase-change entropy of liquid crystals. The principle to only accept experimental vaporization and sublimation data measured at or reduced to standard conditions also enabled the indirect calculation of the standard heat of fusion by applying Equation (2). It has been shown, however, that this indirect approach leads to rather rough estimates, yet still comparable to the often large differences of experimental values originating from different authors. The limits of the present method have been reached in the attempt to predict the total phase-change entropy of liquid crystals. In contrast to ordinary molecules which allow the entropy of fusion to be determined in a mutually comparable manner due to a mostly straightforward, uniform melting process, liquid crystals have proven to be an inconsistent class of compounds in that their melting processes pass through several individual semiliquid phases, preventing a standardized approach for the prediction of their phase-change entropy.

On the whole, the present computer algorithm, integrated in a project called ChemBrain IXL, has proven its versatility in that any extension to calculate the presented and future descriptors only requires a few more lines of controlling code to include the corresponding tables and descriptor names. At present, the project covers thermodynamic (heats of combustion, formation, solvation, vaporization, sublimation and fusion as well as entropy of fusion), solubility-related (logP_o/w_ and logS), optics-related (refractivity), charge-related (polarizability) and environment-related (toxicity) descriptors. On the other hand, it also shows its limitations where the descriptor is either not addressable by the atom groups (e.g., with logBB) or does not describe a uniform characteristic (e.g., the total phase-change entropy). Yet, there is no limit to this ongoing project to extend the number of calculable descriptors beyond the present twelve, provided that there is a number of experimental data available that are large enough and reliable. ChemBrain IXL is available from Neuronix Software (www.neuronix.ch, Rudolf Naef, Lupsingen, Switzerland).

## Figures and Tables

**Table 1:** Atom Groups and their Contributions (in kJ/mol) for Heat-of-Vaporization Calculations.

Entry	Atom Type	Neighbours	Contribution	Occurrences	Molecules
1	Const		8.61	3581	3581
2	B	C3	21.55	2	2
3	B	N2Cl	33.19	1	1
4	B	NCl2	28.59	1	1
5	B	O2Cl	28.23	2	2
6	B	OCl2	25.53	1	1
7	B	S3	76.74	4	4
8	C sp^3^	H3C	3.07	5380	2388
9	C sp^3^	H3N	15.65	242	133
10	C sp^3^	H3N(+)	31.33	2	2
11	C sp^3^	H3O	16.71	372	263
12	C sp^3^	H3S	14.44	31	25
13	C sp^3^	H3P	9.04	6	4
14	C sp^3^	H3Si	5.87	136	53
15	C sp^3^	H2BC	−3.07	6	2
16	C sp^3^	H2C2	4.67	10,588	2030
17	C sp^3^	H2CN	15.00	430	243
18	C sp^3^	H2CN(+)	29.15	10	9
19	C sp^3^	H2CO	15.79	1147	779
20	C sp^3^	H2CS	15.50	159	101
21	C sp^3^	H2CP	6.67	6	2
22	C sp^3^	H2CF	6.20	11	11
23	C sp^3^	H2CCl	14.13	76	65
24	C sp^3^	H2CBr	16.69	24	21
25	C sp^3^	H2CJ	20.90	29	26
26	C sp^3^	H2CSi	2.01	134	54
27	C sp^3^	H2N2	28.27	5	3
28	C sp^3^	H2NO	20.46	4	4
29	C sp^3^	H2O2	27.43	19	16
30	C sp^3^	H2OS	22.40	1	1
31	C sp^3^	H2OF	18.90	1	1
32	C sp^3^	H2OCl	23.06	2	2
33	C sp^3^	H2OSi	10.30	1	1
34	C sp^3^	H2S2	24.08	2	2
35	C sp^3^	H2SSi	6.66	9	9
36	C sp^3^	H2Si2	2.87	2	1
37	C sp^3^	HC3	3.54	939	615
38	C sp^3^	HC2N	12.69	75	64
39	C sp^3^	HC2N(+)	28.39	3	3
40	C sp^3^	HC2O	14.99	243	203
41	C sp^3^	HC2S	13.61	26	22
42	C sp^3^	HC2Si	7.20	6	4
43	C sp^3^	HC2F	5.96	7	6
44	C sp^3^	HC2Cl	9.66	40	38
45	C sp^3^	HC2Br	12.12	21	16
46	C sp^3^	HC2J	18.79	4	4
47	C sp^3^	HCN2(+)	47.10	3	3
48	C sp^3^	HCO2	25.39	25	22
49	C sp^3^	HCOCl	20.93	1	1
50	C sp^3^	HCF2	7.10	15	14
51	C sp^3^	HCFCl	12.61	15	15
52	C sp^3^	HCCl2	16.96	23	22
53	C sp^3^	HCClBr	18.23	1	1
54	C sp^3^	HNO2	32.31	1	1
55	C sp^3^	HO3	37.33	4	4
56	C sp^3^	HOF2	17.06	7	7
57	C sp^3^	HOFCl	20.49	1	1
58	C sp^3^	HSiCl2	23.89	1	1
59	C sp^3^	C4	1.92	335	274
60	C sp^3^	C3N	12.60	28	23
61	C sp^3^	C3N(+)	26.15	4	4
62	C sp^3^	C3O	12.21	135	116
63	C sp^3^	C3S	13.69	18	16
64	C sp^3^	C3F	2.94	31	19
65	C sp^3^	C3Cl	7.77	8	6
66	C sp^3^	C3Br	11.95	3	3
67	C sp^3^	C3J	19.63	2	2
68	C sp^3^	C2NO	20.34	1	1
69	C sp^3^	C2NF	8.88	1	1
70	C sp^3^	C2O2	23.16	35	27
71	C sp^3^	C2OF	18.38	3	3
72	C sp^3^	C2F2	4.75	328	70
73	C sp^3^	C2FCl	8.73	5	5
74	C sp^3^	C2Cl2	13.35	5	5
75	C sp^3^	CN3(+)	46.89	3	3
76	C sp^3^	CNF2	15.25	15	6
77	C sp^3^	CNF2(+)	30.77	3	2
78	C sp^3^	CN2F(+)	28.25	4	3
79	C sp^3^	CO3	28.48	6	6
80	C sp^3^	COF2	13.65	36	30
81	C sp^3^	COCl2	20.61	4	4
82	C sp^3^	CSF2	12.70	2	1
83	C sp^3^	CF3	2.96	147	90
84	C sp^3^	CF2Cl	6.64	10	9
85	C sp^3^	CF2Br	9.02	5	4
86	C sp^3^	CFCl2	13.41	7	7
87	C sp^3^	CFClBr	17.37	1	1
88	C sp^3^	CCl3	17.43	22	21
89	C sp^3^	NF3	14.48	5	4
90	C sp^3^	NF3(+)	−1.76	2	1
91	C sp^3^	N3F(+)	32.36	1	1
92	C sp^3^	O4	38.15	2	2
93	C sp^3^	O2F2	24.80	14	2
94	C sp^3^	OF3	9.71	9	7
95	C sp^3^	OF2Cl	17.84	2	2
96	C sp^3^	OCl3	27.40	2	2
97	C sp^3^	PF3	2.73	2	1
98	C sp^2^	H2=C	2.17	182	170
99	C sp^2^	HC=C	5.03	1314	694
100	C sp^2^	HC=N	8.81	15	15
101	C sp^2^	HC=O	11.44	122	122
102	C sp^2^	H=CN	17.18	103	57
103	C sp^2^	H=CO	10.25	35	32
104	C sp^2^	H=CS	8.20	49	35
105	C sp^2^	H=CSi	10.77	4	4
106	C sp^2^	H=CF	−0.09	1	1
107	C sp^2^	H=CCl	10.38	8	6
108	C sp^2^	H=CBr	13.73	1	1
109	C sp^2^	HN=N	30.13	39	39
110	C sp^2^	HN=O	34.46	6	6
111	C sp^2^	H=NO	14.07	1	1
112	C sp^2^	H=NS	18.07	2	2
113	C sp^2^	HO=O	18.86	14	12
114	C sp^2^	C2=C	5.27	220	190
115	C sp^2^	C2=N	8.22	15	14
116	C sp^2^	C2=O	13.59	149	140
117	C sp^2^	C=CN	15.36	14	10
118	C sp^2^	C=CO	12.54	39	31
119	C sp^2^	C2=S	71.29	2	2
120	C sp^2^	C=CS	9.45	29	24
121	C sp^2^	C=CF	2.72	11	5
122	C sp^2^	C=CCl	5.83	8	5
123	C sp^2^	C=CBr	15.79	1	1
124	C sp^2^	=CN2	9.12	3	2
125	C sp^2^	CN=N	28.80	16	16
126	C sp^2^	CN=N(+)	11.32	2	2
127	C sp^2^	CN=O	35.35	47	47
128	C sp^2^	C=NO	22.79	5	5
129	C sp^2^	CN=S	18.27	3	2
130	C sp^2^	C=NS	17.49	1	1
131	C sp^2^	C=NCl	11.93	1	1
132	C sp^2^	=CNCl	22.67	2	1
133	C sp^2^	CO=O	17.20	684	594
134	C sp^2^	=COS	17.48	1	1
135	C sp^2^	C=OS	12.33	9	9
136	C sp^2^	=COF	15.53	1	1
137	C sp^2^	C=OCl	15.41	11	9
138	C sp^2^	C=OBr	22.28	3	3
139	C sp^2^	C=OJ	25.82	2	2
140	C sp^2^	=CF2	−0.26	3	3
141	C sp^2^	=CFCl	9.81	3	2
142	C sp^2^	=CCl2	17.52	6	5
143	C sp^2^	N2=N	29.25	2	2
144	C sp^2^	N2=O	35.05	3	3
145	C sp^2^	N=NS	13.50	5	5
146	C sp^2^	NO=O	33.48	3	3
147	C sp^2^	=NOCl	24.27	1	1
148	C sp^2^	NS=S	44.39	2	2
149	C sp^2^	O2=O	31.57	13	13
150	C sp^2^	O=OCl	22.73	2	2
151	C sp^2^	S2=S	34.03	1	1
152	C aromatic	H:C2	4.64	4749	928
153	C aromatic	H:C:N	11.74	118	70
154	C aromatic	H:C:N(+)	22.04	2	1
155	C aromatic	H:N2	15.36	7	5
156	C aromatic	:C3	6.67	233	69
157	C aromatic	C:C2	5.29	1053	618
158	C aromatic	C:C:N	9.94	38	30
159	C aromatic	:C2N	14.44	140	115
160	C aromatic	:C2N(+)	24.38	33	31
161	C aromatic	:C2:N	10.60	21	14
162	C aromatic	:C2O	8.04	443	253
163	C aromatic	:C2S	9.47	30	25
164	C aromatic	:C2Si	4.67	10	8
165	C aromatic	:C2F	4.45	143	72
166	C aromatic	:C2Cl	9.43	429	146
167	C aromatic	:C2Br	12.49	149	69
168	C aromatic	:C2J	19.48	29	26
169	C aromatic	:CN:N	16.72	2	2
170	C aromatic	:C:NO	13.67	4	3
171	C aromatic	:C:NF	14.34	1	1
172	C aromatic	:C:NCl	15.74	3	3
173	C aromatic	:C:NBr	25.24	1	1
174	C aromatic	N:N2	20.19	5	2
175	C aromatic	:N2O	16.44	2	2
176	C sp	H#C	2.42	15	14
177	C sp	C#C	6.05	62	33
178	C sp	=C2	5.50	4	4
179	C sp	C#N	17.38	72	70
180	C sp	#CCl	9.31	3	2
181	C sp	=N=O	10.44	6	5
182	C sp	=N=S	23.08	3	3
183	N sp^3^	H2C	2.30	78	58
184	N sp^3^	H2C(pi)	8.05	61	59
185	N sp^3^	H2N	19.23	8	7
186	N sp^3^	H2S	28.18	2	2
187	N sp^3^	HC2	−11.34	59	56
188	N sp^3^	HC2(pi)	−1.94	27	26
189	N sp^3^	HC2(2pi)	−2.43	21	21
190	N sp^3^	HCN	−0.76	3	2
191	N sp^3^	HCN(pi)	−13.33	3	3
192	N sp^3^	HCN(2pi)	4.97	1	1
193	N sp^3^	HCS(pi)	5.34	7	7
194	N sp^3^	HCSi	−4.02	6	6
195	N sp^3^	HSi2	1.94	1	1
196	N sp^3^	BC2	−31.30	3	2
197	N sp^3^	C3	−30.50	111	101
198	N sp^3^	C3(pi)	−25.56	37	31
199	N sp^3^	C3(2pi)	−22.95	52	50
200	N sp^3^	C3(3pi)	−27.03	13	13
201	N sp^3^	C2N	−19.64	4	3
202	N sp^3^	C2N(+)	0.00	1	1
203	N sp^3^	C2N(pi)	−27.16	3	2
204	N sp^3^	C2N(+)(pi)	3.24	4	4
205	N sp^3^	C2N(2pi)	−24.28	4	4
206	N sp^3^	C2N(3pi)	−26.84	2	2
207	N sp^3^	C2O	8.24	1	1
208	N sp^3^	C2P	−17.98	5	2
209	N sp^3^	C2Si	−19.79	12	8
210	N sp^3^	CN2(2pi)	−36.43	1	1
211	N sp^3^	CN2(+)(2pi)	16.44	1	1
212	N sp^3^	CF2	−4.56	2	2
213	N sp^3^	CF2(pi)	−12.61	1	1
214	N sp^3^	CSi2	−17.81	1	1
215	N sp^3^	Si3	−1.79	1	1
216	N sp^2^	H=C	1.29	2	2
217	N sp^2^	C=C	−10.46	85	82
218	N sp^2^	C=N	−5.89	19	10
219	N sp^2^	C=N(+)	−2.79	15	13
220	N sp^2^	=CN	18.81	9	9
221	N sp^2^	=CO	10.27	17	14
222	N sp^2^	=CF	0.00	1	1
223	N sp^2^	N=N	15.91	5	3
224	N sp^2^	O=O	0.59	7	7
225	N aromatic	:C2	−5.10	104	78
226	N aromatic	:C:N	5.35	8	4
227	N(+) sp^3^	C2NO(-)	0.00	1	1
228	N(+) sp^2^	CO=O(-)	−2.09	78	56
229	N(+) sp^2^	C=NO(-)	−19.89	3	3
230	N(+) sp^2^	NO=O(-)	0.35	6	5
231	N(+) sp^2^	O2=O(-)	9.02	17	11
232	N(+) aromatic	:C2O(-)	0.00	1	1
233	N(+) sp	C#C(-)	−8.48	2	2
234	N(+) sp	=N2(-)	5.96	12	10
235	O	HC	14.55	322	288
236	O	HC(pi)	20.98	174	157
237	O	HN	0.00	1	1
238	O	HN(pi)	19.03	2	2
239	O	HO	23.75	5	5
240	O	HSi	26.41	1	1
241	O	BC	−17.91	5	3
242	O	C2	−17.86	424	270
243	O	C2(pi)	−13.29	744	629
244	O	C2(2pi)	−7.15	145	120
245	O	CN(pi)	0.00	7	7
246	O	CN(+)(pi)	2.17	17	11
247	O	CN(2pi)	−2.82	9	9
248	O	CO	−8.76	54	20
249	O	CS	2.45	18	9
250	O	CP	−2.71	104	42
251	O	CP(pi)	1.25	7	5
252	O	CSi	−11.39	79	29
253	O	CSi(pi)	−14.85	37	13
254	O	N2(2pi)	−0.72	3	3
255	O	OSi	4.23	9	4
256	O	P2	16.68	1	1
257	O	Si2	−6.52	15	4
258	P3	C3	−6.83	3	3
259	P3	C2O	2.71	1	1
260	P3	N3	−7.09	1	1
261	P3	N2Cl	10.64	1	1
262	P3	O3	−4.07	1	1
263	P4	HO2=O	9.23	2	2
264	P4	CO2=O	5.40	3	3
265	P4	O3=O	−3.86	16	15
266	P4	O3=S	1.10	9	9
267	P4	O2=OS	1.77	4	4
268	P4	O2S=S	1.73	8	8
269	S2	HC	1.49	33	29
270	S2	HC(pi)	6.23	1	1
271	S2	HP	23.50	3	3
272	S2	BC	−24.53	12	4
273	S2	C2	−10.51	67	65
274	S2	C2(pi)	−2.71	23	22
275	S2	C2(2pi)	0.53	44	44
276	S2	CS	−0.35	16	8
277	S2	CS(pi)	2.39	2	1
278	S2	CP	−1.99	9	9
279	S2	Si2	−3.40	1	1
280	S4	C2=O	22.60	4	4
281	S4	C2=O2	27.80	9	9
282	S4	C2F2	−5.92	1	1
283	S4	CN=O2	1.94	9	9
284	S4	C=O2S	37.54	2	1
285	S4	O2=O	−3.83	5	5
286	S4	O2=O2	4.79	4	4
287	Si	H3C	0.00	1	1
288	Si	H2CN	2.20	1	1
289	Si	HC3	−4.21	24	24
290	Si	HC2O	2.36	2	1
291	Si	HC2S	0.00	2	1
292	Si	HCO2	8.33	5	1
293	Si	HN3	8.01	2	2
294	Si	C4	−0.57	21	20
295	Si	C3N	−1.80	18	14
296	Si	C3O	0.35	6	6
297	Si	C2O2	5.64	18	11
298	Si	CO3	−2.40	26	26
299	Si	O4	−16.14	6	6
300	H	H Acceptor	−12.45	16	16
301	Alkane	No. of C atoms	0.09	3072	286
302	Unsaturated HC	No. of C atoms	−0.07	4100	413
A	Based on	Valid groups	185		3581
B	Goodness of fit	*R*^2^	0.9678		3460
C	Deviation	Average	2.99		3460
D	Deviation	Standard	4.30		3460
E	K-fold cv	K	10		3386
F	Goodness of fit	*Q*^2^	0.9641		3386
G	Deviation	Average (cv)	3.14		3386
H	Deviation	Standard (cv)	4.56		3386

**Table 2:** Atom Groups and their Contributions (in kJ/mol) for Heat-of-Sublimation Calculations.

Entry	Atom Type	Neighbours	Contribution	Occurrences	Molecules
1	Const		21.03	1960	1960
2	B	C3	65.82	2	2
3	C sp^3^	H3C	5.99	1322	623
4	C sp^3^	H3N	26.96	143	87
5	C sp^3^	H3N(+)	98.98	1	1
6	C sp^3^	H3O	28.51	181	122
7	C sp^3^	H3S	30.06	7	6
8	C sp^3^	H2C2	6.88	2602	508
9	C sp^3^	H2CN	21.98	224	116
10	C sp^3^	H2CN(+)	27.46	13	11
11	C sp^3^	H2CO	29.62	242	134
12	C sp^3^	H2CS	23.29	50	31
13	C sp^3^	H2CF	15.91	1	1
14	C sp^3^	H2CCl	17.59	3	3
15	C sp^3^	H2CBr	22.76	5	4
16	C sp^3^	H2CJ	21.83	3	2
17	C sp^3^	H2N2	43.95	18	6
18	C sp^3^	H2NCl	36.29	1	1
19	C sp^3^	H2O2	53.35	25	13
20	C sp^3^	H2OS	54.78	1	1
21	C sp^3^	H2S2	47.45	6	4
22	C sp^3^	HBC2	−36.17	3	1
23	C sp^3^	HC3	2.28	509	190
24	C sp^3^	HC2N	14.28	34	30
25	C sp^3^	HC2N(+)	21.01	9	9
26	C sp^3^	HC2O	24.27	82	47
27	C sp^3^	HC2S	17.59	14	11
28	C sp^3^	HC2F	5.18	1	1
29	C sp^3^	HC2Cl	11.49	7	2
30	C sp^3^	HC2Br	−0.95	1	1
31	C sp^3^	HCN2	39.48	8	2
32	C sp^3^	HCN2(+)	39.93	2	2
33	C sp^3^	HCNO	34.73	2	1
34	C sp^3^	HCNS	20.56	2	1
35	C sp^3^	HCO2	39.96	3	3
36	C sp^3^	HCF2	−0.19	1	1
37	C sp^3^	HCCl2	15.78	1	1
38	C sp^3^	HN3(+)	37.31	1	1
39	C sp^3^	HO3	72.23	3	3
40	C sp^3^	C4	−4.25	209	137
41	C sp^3^	C3N	5.87	18	13
42	C sp^3^	C3N(+)	18.44	14	11
43	C sp^3^	C3O	15.18	40	31
44	C sp^3^	C3S	6.40	5	5
45	C sp^3^	C3F	1.89	3	3
46	C sp^3^	C3Cl	−8.06	1	1
47	C sp^3^	C3Br	2.34	1	1
48	C sp^3^	C2N2(+)	34.78	7	6
49	C sp^3^	C2O2	39.73	8	8
50	C sp^3^	C2S2	37.28	4	1
51	C sp^3^	C2F2	7.07	62	8
52	C sp^3^	CN3(+)	43.89	19	12
53	C sp^3^	CN2F(+)	25.98	1	1
54	C sp^3^	CO3	57.42	2	2
55	C sp^3^	CF3	−4.71	27	23
56	C sp^3^	CCl3	16.10	3	2
57	C sp^3^	N3F(+)	44.00	1	1
58	C sp^3^	O4	73.43	1	1
59	C sp^2^	H2=C	7.97	12	12
60	C sp^2^	HC=C	5.10	452	213
61	C sp^2^	HC=N	35.49	21	19
62	C sp^2^	HC=N(+)	72.64	7	7
63	C sp^2^	H=CN	32.79	83	69
64	C sp^2^	HC=O	20.74	15	15
65	C sp^2^	H=CO	16.89	16	14
66	C sp^2^	H=CS	15.22	49	36
67	C sp^2^	HN=N	55.52	19	18
68	C sp^2^	HN=O	35.41	4	3
69	C sp^2^	H=NO	40.91	1	1
70	C sp^2^	H=NS	33.85	2	2
71	C sp^2^	C2=C	3.91	78	61
72	C sp^2^	C2=N	30.47	35	26
73	C sp^2^	C2=N(+)	13.76	5	5
74	C sp^2^	C=CN	26.81	57	48
75	C sp^2^	C=CN(+)	41.65	7	7
76	C sp^2^	C2=O	15.10	200	161
77	C sp^2^	C=CO	22.08	40	31
78	C sp^2^	C2=S	18.21	3	3
79	C sp^2^	C=CS	15.64	36	27
80	C sp^2^	C=CF	16.81	2	2
81	C sp^2^	C=CCl	11.02	9	5
82	C sp^2^	C=CBr	34.06	2	2
83	C sp^2^	C=CJ	32.46	1	1
84	C sp^2^	=CN2	64.94	6	6
85	C sp^2^	=CN2(+)	60.65	4	4
86	C sp^2^	CN=N	54.51	27	25
87	C sp^2^	CN=N(+)	44.16	3	3
88	C sp^2^	CN=O	39.66	234	194
89	C sp^2^	C=NO	42.74	2	2
90	C sp^2^	CN=S	39.85	8	7
91	C sp^2^	C=NS	34.89	1	1
92	C sp^2^	=CNS(+)	41.29	2	2
93	C sp^2^	=CNCl	38.14	4	3
94	C sp^2^	CO=O	34.06	424	345
95	C sp^2^	CO=O(-)	80.89	22	22
96	C sp^2^	C=OCl	29.03	1	1
97	C sp^2^	CS=S	56.97	3	3
98	C sp^2^	N2=N	80.72	4	4
99	C sp^2^	N2=N(+)	65.95	6	5
100	C sp^2^	N2=O	59.57	76	70
101	C sp^2^	N2=S	66.62	29	29
102	C sp^2^	N=NS	51.62	22	22
103	C sp^2^	NO=O	52.79	8	8
104	C sp^2^	=NO2	61.12	1	1
105	C sp^2^	N=OS	48.27	1	1
106	C sp^2^	NO=S	58.04	11	11
107	C sp^2^	=NOS	52.75	1	1
108	C sp^2^	NS=S	60.83	5	3
109	C sp^2^	=NS2	64.37	1	1
110	C sp^2^	O2=O	41.40	7	7
111	C sp^2^	=OS2	41.22	2	2
112	C sp^2^	OS=S	73.06	1	1
113	C sp^2^	S2=S	49.39	5	5
114	C aromatic	H:C2	5.36	7115	1269
115	C aromatic	H:C:N	18.20	150	96
116	C aromatic	H:C:N(+)	28.26	48	28
117	C aromatic	H:N2	23.27	7	5
118	C aromatic	B:C2	−25.04	3	1
119	C aromatic	:C3	5.51	454	155
120	C aromatic	C:C2	3.12	1684	835
121	C aromatic	C:C:N	11.10	80	48
122	C aromatic	C:C:N(+)	16.04	33	21
123	C aromatic	:C2N	22.21	354	258
124	C aromatic	:C2N(+)	28.67	169	134
125	C aromatic	:C2:N	17.03	79	61
126	C aromatic	:C2:N(+)	18.05	35	20
127	C aromatic	:C2O	20.46	617	387
128	C aromatic	:C2P	−1.63	12	4
129	C aromatic	:C2S	16.31	80	64
130	C aromatic	:C2F	4.45	77	42
131	C aromatic	:C2Cl	12.48	424	166
132	C aromatic	:C2Br	14.66	63	43
133	C aromatic	:C2J	20.68	31	27
134	C aromatic	:C2Si	4.80	10	2
135	C aromatic	C:N2	28.80	4	2
136	C aromatic	:CN:N	29.72	11	9
137	C aromatic	:CN:N(+)	33.74	3	2
138	C aromatic	:C:NO	41.44	13	12
139	C aromatic	:C:NO(+)	33.50	5	5
140	C aromatic	:C:NCl	21.70	18	13
141	C aromatic	:C:NBr	31.31	3	2
142	C aromatic	N:N2	43.11	13	8
143	C aromatic	:N2O	39.92	3	1
144	C aromatic	:N2S	36.08	3	3
145	C aromatic	:N2Cl	35.90	3	3
146	C sp	=C2	6.39	3	2
147	C sp	C#C	3.24	14	7
148	C sp	C#N	16.49	96	67
149	C sp	C#N(+)	11.33	4	3
150	C sp	#CS	28.03	2	2
151	C sp	N#N	47.80	1	1
152	C sp	#NP	12.53	3	1
153	N sp^3^	H2C	5.03	23	12
154	N sp^3^	H2C(pi)	6.38	223	199
155	N sp^3^	H2N	17.97	10	8
156	N sp^3^	H2S	41.98	1	1
157	N sp^3^	HC2	−23.83	14	13
158	N sp^3^	HC2(pi)	−13.51	72	55
159	N sp^3^	HC2(2pi)	−20.10	200	165
160	N sp^3^	HCN	−0.15	2	1
161	N sp^3^	HCN(pi)	6.71	14	9
162	N sp^3^	HCN(2pi)	−6.84	25	25
163	N sp^3^	HCS(pi)	−15.10	20	20
164	N sp^3^	C3	−51.07	16	11
165	N sp^3^	C3(pi)	−53.90	59	49
166	N sp^3^	C3(2pi)	−60.80	72	54
167	N sp^3^	C3(3pi)	−61.26	18	14
168	N sp^3^	C2N(pi)	−7.05	6	3
169	N sp^3^	C2N(+)(pi)	−5.52	24	9
170	N sp^3^	C2N(2pi)	−36.36	4	4
171	N sp^3^	C2N(+)(2pi)	−20.13	1	1
172	N sp^3^	C2N(3pi)	−54.74	3	3
173	N sp^3^	C2S	−49.13	4	2
174	N sp^3^	C2F(2pi)	−64.78	1	1
175	N sp^3^	CN2(pi)	30.74	4	3
176	N sp^3^	CN2(2pi)	−49.40	3	3
177	N sp^3^	CN2(+)(2pi)	3.72	1	1
178	N sp^3^	CNF(2pi)	−34.74	5	4
179	N sp^2^	C=C	−32.77	79	74
180	N sp^2^	C=N	−4.54	13	9
181	N sp^2^	C=N(+)	−15.43	5	5
182	N sp^2^	=CN	−4.63	38	36
183	N sp^2^	=CN(+)	36.68	1	1
184	N sp^2^	C=O	−12.04	9	9
185	N sp^2^	C=P	−49.18	1	1
186	N sp^2^	=CO	−16.24	18	13
187	N sp^2^	=CS	−26.78	10	8
188	N sp^2^	N=N	12.19	21	13
189	N sp^2^	N=O	0.00	10	6
190	N sp^2^	=NO	−6.67	2	1
191	N aromatic	:C2	−14.01	208	145
192	N aromatic	:C:N	−4.98	4	2
193	N(+) sp^3^	H3C	2.77	13	13
194	N(+) sp^3^	H2C2	−82.36	3	3
195	N(+) sp^2^	C=CO(-)	−68.61	7	7
196	N(+) sp^2^	C=NO	−26.37	10	5
197	N(+) sp^2^	C=NO(-)	−11.30	3	3
198	N(+) sp^2^	CO=O(-)	−4.38	270	163
199	N(+) sp^2^	=CO2(-)	2.17	5	5
200	N(+) sp^2^	NO=O(-)	0.15	28	12
201	N(+) sp^2^	O2=O(-)	6.00	14	6
202	N(+) aromatic	H:C2	−46.79	6	6
203	N(+) aromatic	:C2O(-)	−7.10	56	40
204	N(+) sp	C#C(-)	−14.36	3	3
205	N(+) sp	#CO(-)	0.00	4	3
206	N(+) sp	=N2(-)	19.14	2	2
207	O	HC	4.49	143	92
208	O	HC(pi)	8.19	560	470
209	O	HN(pi)	2.28	4	3
210	O	HO	29.95	4	4
211	O	C2	−39.23	94	37
212	O	C2(pi)	−31.33	292	201
213	O	C2(2pi)	−24.06	147	121
214	O	CN(pi)	0.00	2	1
215	O	CN(+)(pi)	0.00	14	6
216	O	CN(2pi)	4.91	1	1
217	O	CO(pi)	−27.16	8	6
218	O	CP(pi)	−16.12	3	1
219	O	N2(2pi)	5.87	4	4
220	O	N2(+)(2pi)	6.27	5	5
221	P3	C3	16.70	2	2
222	P3	S3	−66.68	1	1
223	P4	C3=N	0.00	1	1
224	P4	C3=O	−30.50	1	1
225	P4	C3=S	46.30	1	1
226	P4	O3=O	0.00	1	1
227	S2	HC	−2.58	1	1
228	S2	HC(pi)	18.47	2	2
229	S2	C2	−22.69	19	12
230	S2	C2(pi)	−15.86	34	29
231	S2	C2(2pi)	−7.94	59	49
232	S2	CN(pi)	25.96	1	1
233	S2	CN(2pi)	−6.82	6	6
234	S2	CS(pi)	−6.16	8	4
235	S2	CP(pi)	0.00	3	1
236	S2	N2	−2.00	1	1
237	S2	N2(2pi)	21.36	2	2
238	S2	NS	1.00	2	1
239	S4	C2=O	−5.89	2	2
240	S4	C2=O2	−4.26	27	27
241	S4	CN=O2	9.20	20	20
242	Si	C4	2.02	1	1
243	Si	C3Si	−0.67	2	1
244	H	H Acceptor	−8.63	107	89
245	Alkane	No. of C atoms	−0.53	849	59
246	Unsaturated HC	No. of C atoms	−0.10	2679	148
A	Based on	Valid groups	154		1960
B	Goodness of fit	*R*^2^	0.8887		1866
C	Deviation	Average	7.81		1866
D	Deviation	Standard	10.33		1866
E	K-fold cv	K	10		1791
F	Goodness of fit	*Q*^2^	0.8657		1791
G	Deviation	Average (cv)	8.56		1791
H	Deviation	Standard (cv)	11.39		1791

**Table 3:** Atom Groups and their Contributions (in kJ/mol) for Heat-of-Solvation Calculations.

Entry	Atom Type	Neighbours	Contribution	Occurrences	Molecules
1	Const		−13.33	436	436
2	C sp^3^	H3C	−4.44	483	265
3	C sp^3^	H3N	−31.51	47	28
4	C sp^3^	H3N(+)	−31.22	1	1
5	C sp^3^	H3O	−15.38	34	29
6	C sp^3^	H3S	−12.79	7	4
7	C sp^3^	H2C2	−3.86	506	186
8	C sp^3^	H2CN	−31.29	55	37
9	C sp^3^	H2CN(+)	−22.60	2	2
10	C sp^3^	H2CO	−15.26	178	90
11	C sp^3^	H2CS	−12.03	9	6
12	C sp^3^	H2CF	−6.02	1	1
13	C sp^3^	H2CCl	−8.52	15	11
14	C sp^3^	H2CBr	−11.73	1	1
15	C sp^3^	H2CJ	−13.80	2	2
16	C sp^3^	H2O2	−14.86	1	1
17	C sp^3^	HC3	−2.51	45	35
18	C sp^3^	HC2N	−29.99	6	5
19	C sp^3^	HC2N(+)	−20.74	1	1
20	C sp^3^	HC2O	−14.95	32	29
21	C sp^3^	HC2F	−5.77	1	1
22	C sp^3^	HC2Cl	−8.53	1	1
23	C sp^3^	HC2J	−14.39	1	1
24	C sp^3^	HCF2	−5.07	3	3
25	C sp^3^	HCCl2	−11.02	5	4
26	C sp^3^	C4	0.43	10	10
27	C sp^3^	C3N	−24.37	3	3
28	C sp^3^	C3O	−16.23	6	6
29	C sp^3^	C3Cl	−1.29	1	1
30	C sp^3^	C3Br	1.24	1	1
31	C sp^3^	C3J	−7.51	1	1
32	C sp^3^	C2F2	−5.12	2	2
33	C sp^3^	COF2	0.74	1	1
34	C sp^3^	CF3	−2.85	11	9
35	C sp^3^	CF2Cl	−3.44	3	2
36	C sp^3^	CFCl2	−12.04	1	1
37	C sp^3^	CCl3	−12.64	2	2
38	C sp^2^	H2=C	−2.93	15	13
39	C sp^2^	HC=C	−2.16	26	20
40	C sp^2^	HC=O	−16.45	9	9
41	C sp^2^	H=CN	−13.78	17	13
42	C sp^2^	H=CO	−10.21	1	1
43	C sp^2^	H=CS	−6.13	2	1
44	C sp^2^	H=CCl	−7.34	5	3
45	C sp^2^	HN=N	−10.70	2	2
46	C sp^2^	HN=O	−33.05	4	4
47	C sp^2^	HO=O	−14.45	7	7
48	C sp^2^	C2=C	1.28	11	11
49	C sp^2^	C=CN	−15.51	2	2
50	C sp^2^	C=CN(+)	−39.48	1	1
51	C sp^2^	C2=O	−17.65	20	20
52	C sp^2^	C=CF	−6.97	2	2
53	C sp^2^	C=CCl	−31.39	1	1
54	C sp^2^	C=CBr	−28.79	1	1
55	C sp^2^	C=CJ	−31.42	1	1
56	C sp^2^	=CN2	−32.45	3	3
57	C sp^2^	CN=O	−39.35	30	30
58	C sp^2^	=CNCl	−30.33	1	1
59	C sp^2^	CO=O	−17.24	63	52
60	C sp^2^	=CF2	0.44	3	2
61	C sp^2^	=CCl2	−11.89	2	2
62	C sp^2^	N2=O	−35.29	25	25
63	C sp^2^	N2=S	−41.79	6	6
64	C aromatic	H:C2	−2.84	437	100
65	C aromatic	H:C:N	−14.82	29	18
66	C aromatic	:C3	−3.23	13	6
67	C aromatic	C:C2	−1.72	90	63
68	C aromatic	C:C:N	−15.13	7	6
69	C aromatic	:C2N	−10.35	13	13
70	C aromatic	:C2N(+)	−21.83	6	6
71	C aromatic	:C2:N	−15.19	1	1
72	C aromatic	:C2O	−9.63	21	17
73	C aromatic	:C2F	−1.79	1	1
74	C aromatic	:C2Cl	−3.91	37	19
75	C aromatic	:C2Br	−5.99	1	1
76	C aromatic	:CN:N	−16.20	1	1
77	C sp	H#C	−1.37	1	1
78	C sp	C#C	0.00	1	1
79	C sp	C#N	−17.66	15	12
80	N sp^3^	H2C	−2.40	25	20
81	N sp^3^	H2C(pi)	−16.13	32	30
82	N sp^3^	HC2	24.30	6	6
83	N sp^3^	HC2(pi)	11.97	26	22
84	N sp^3^	HC2(2pi)	3.09	21	12
85	N sp^3^	C3	57.51	5	5
86	N sp^3^	C3(pi)	52.51	10	9
87	N sp^3^	C3(2pi)	36.53	13	8
88	N sp^2^	C=C	−19.81	2	2
89	N aromatic	:C2	5.38	19	19
90	N(+) sp^2^	CO=O(-)	8.85	11	11
91	O	HC	−17.23	61	50
92	O	HC(pi)	−18.29	32	26
93	O	HO	−22.54	2	1
94	O	C2	8.60	68	39
95	O	C2(pi)	10.97	56	49
96	O	C2(2pi)	9.97	2	2
97	S2	HC	1.98	4	4
98	S2	C2	6.62	3	3
99	S2	C2(2pi)	0.00	1	1
100	S2	CS	2.30	4	2
101	S4	C2=O	−33.00	1	1
102	H	H Acceptor	10.02	2	2
103	Alkane	No. of C atoms	0.96	142	23
104	Unsaturated HC	No. of C atoms	0.25	307	37
A	Based on	Valid groups	61		436
B	Goodness of fit	*R*^2^	0.9731		388
C	Deviation	Average	2.68		388
D	Deviation	Standard	3.53		388
E	K-fold cv	K	10		373
F	Goodness of fit	*Q*^2^	0.9546		373
G	Deviation	Average (cv)	3.22		373
H	Deviation	Standard (cv)	4.34		373

**Table 4:** Atom Groups and their Contributions (in J/mol/K) for Entropy-of-Fusion Calculations.

Entry	Atom Type	Neighbours	Contribution	Occurrences	Molecules
1	Const		31.12	2809	2809
2	B	C3	12.34	2	2
3	B	CO2	51.11	5	5
4	C sp^3^	H3B	−4.93	3	1
5	C sp^3^	H2BC	4.93	3	1
6	C sp^3^	H3C	1.90	2944	1402
7	C sp^3^	H3N	15.63	279	149
8	C sp^3^	H3N(+)	7.07	2	2
9	C sp^3^	H3O	14.42	366	232
10	C sp^3^	H3P	21.07	3	3
11	C sp^3^	H3S	12.93	35	31
12	C sp^3^	H3Si	8.19	283	46
13	C sp^3^	H2C2	8.46	8600	1239
14	C sp^3^	H2CN	14.85	505	257
15	C sp^3^	H2CN(+)	19.09	29	21
16	C sp^3^	H2CO	14.52	952	473
17	C sp^3^	H2CP	17.50	3	2
18	C sp^3^	H2CS	16.77	166	83
19	C sp^3^	H2CF	12.36	1	1
20	C sp^3^	H2CCl	10.67	30	24
21	C sp^3^	H2CBr	11.79	24	17
22	C sp^3^	H2CJ	3.10	2	2
23	C sp^3^	H2CSi	8.50	62	20
24	C sp^3^	H2N2	5.03	20	11
25	C sp^3^	H2NO	8.98	8	7
26	C sp^3^	H2NS	43.70	4	4
27	C sp^3^	H2O2	22.34	23	14
28	C sp^3^	H2S2	29.21	7	5
29	C sp^3^	H2SCl	22.89	1	1
30	C sp^3^	H2Si2	12.02	6	3
31	C sp^3^	HC3	0.64	817	388
32	C sp^3^	HC2N	18.09	117	103
33	C sp^3^	HC2N(+)	−9.91	16	16
34	C sp^3^	HC2O	10.63	357	226
35	C sp^3^	HC2S	9.80	18	13
36	C sp^3^	HC2F	8.23	2	2
37	C sp^3^	HC2Cl	10.38	22	10
38	C sp^3^	HC2Br	8.94	5	4
39	C sp^3^	HC2Si	−14.02	1	1
40	C sp^3^	HCN2	1.21	2	1
41	C sp^3^	HCNO	23.14	7	6
42	C sp^3^	HCNS	23.70	1	1
43	C sp^3^	HCO2	19.18	30	26
44	C sp^3^	HCOCl	19.13	2	1
45	C sp^3^	HCF2	4.20	4	4
46	C sp^3^	HCFCl	−10.16	1	1
47	C sp^3^	HCCl2	9.01	10	9
48	C sp^3^	HCClBr	−3.80	1	1
49	C sp^3^	C4	−0.23	435	256
50	C sp^3^	C3N	14.87	22	20
51	C sp^3^	C3N(+)	12.86	6	5
52	C sp^3^	C3O	4.63	81	74
53	C sp^3^	C3S	16.54	6	6
54	C sp^3^	C3F	18.64	14	12
55	C sp^3^	C3Cl	9.23	14	9
56	C sp^3^	C3Br	3.44	2	2
57	C sp^3^	C3J	31.10	1	1
58	C sp^3^	C2N2	52.69	3	2
59	C sp^3^	C2N2(+)	4.24	7	6
60	C sp^3^	C2NO	34.66	1	1
61	C sp^3^	C2NF	47.27	1	1
62	C sp^3^	C2NCl(+)	13.35	1	1
63	C sp^3^	C2O2	13.44	47	29
64	C sp^3^	C2S2	10.13	1	1
65	C sp^3^	C2F2	−0.09	262	37
66	C sp^3^	C2Cl2	10.32	9	7
67	C sp^3^	CN3(+)	7.29	6	5
68	C sp^3^	CNF2	6.86	7	3
69	C sp^3^	COF2	−3.57	4	3
70	C sp^3^	CS3	30.56	4	1
71	C sp^3^	CSF2	41.61	2	1
72	C sp^3^	CSCl2	46.90	2	2
73	C sp^3^	CF3	3.38	91	76
74	C sp^3^	CF2Cl	−1.55	6	5
75	C sp^3^	CF2Br	8.94	4	3
76	C sp^3^	CFCl2	−6.89	3	2
77	C sp^3^	CCl3	0.92	17	16
78	C sp^3^	NF3	11.04	1	1
79	C sp^3^	O2F2	20.23	1	1
80	C sp^3^	OF3	2.25	2	2
81	C sp^3^	SF3	24.96	4	4
82	C sp^3^	SCl3	46.90	1	1
83	C sp^3^	SiCl3	14.20	1	1
84	C sp^2^	H2=C	5.49	84	76
85	C sp^2^	HC=C	2.46	607	323
86	C sp^2^	HC=N	−0.81	48	40
87	C sp^2^	H=CN	3.18	44	37
88	C sp^2^	HC=O	8.29	18	18
89	C sp^2^	H=CO	5.29	19	17
90	C sp^2^	H=CS	−1.85	43	33
91	C sp^2^	H=CCl	10.11	3	3
92	C sp^2^	H=CSi	2.92	3	3
93	C sp^2^	HN=N	9.78	30	22
94	C sp^2^	HN=O	−10.25	3	3
95	C sp^2^	H=NO	21.94	1	1
96	C sp^2^	H=NS	1.04	4	4
97	C sp^2^	HO=O	14.63	2	2
98	C sp^2^	C2=C	0.30	212	166
99	C sp^2^	C2=N	7.33	35	33
100	C sp^2^	C2=N(+)	2.31	1	1
101	C sp^2^	C=CN	−2.70	51	45
102	C sp^2^	C=CN(+)	0.00	2	1
103	C sp^2^	C2=O	1.57	386	298
104	C sp^2^	C=CO	5.58	70	52
105	C sp^2^	C=CS	0.18	38	25
106	C sp^2^	C=CCl	3.68	20	13
107	C sp^2^	C=CBr	45.90	1	1
108	C sp^2^	=CN2	12.85	17	17
109	C sp^2^	=CN2(+)	6.14	1	1
110	C sp^2^	CN=N	1.47	25	19
111	C sp^2^	=CNO	−1.47	6	4
112	C sp^2^	CN=O	0.63	366	234
113	C sp^2^	C=NO	9.33	5	5
114	C sp^2^	C=NS	7.20	7	7
115	C sp^2^	CN=S	−2.87	10	8
116	C sp^2^	=CNCl	11.25	1	1
117	C sp^2^	CO=O	5.68	718	546
118	C sp^2^	CO=O(-)	−16.84	19	19
119	C sp^2^	C=OF	9.78	3	2
120	C sp^2^	C=OCl	14.97	2	1
121	C sp^2^	C=OS	16.72	1	1
122	C sp^2^	=CS2	−7.29	12	2
123	C sp^2^	=CSCl	2.93	3	2
124	C sp^2^	=CSBr	−4.03	1	1
125	C sp^2^	=CF2	11.60	3	2
126	C sp^2^	=CFCl	1.87	1	1
127	C sp^2^	=CCl2	5.32	9	8
128	C sp^2^	=CBr2	46.05	1	1
129	C sp^2^	N2=N	11.87	9	9
130	C sp^2^	N2=O	−3.48	90	84
131	C sp^2^	N=NO	3.41	1	1
132	C sp^2^	N2=S	0.55	32	31
133	C sp^2^	N=NS	−3.08	23	23
134	C sp^2^	NO=O	0.38	62	60
135	C sp^2^	N=OS	20.86	2	2
136	C sp^2^	NO=S	−2.08	8	8
137	C sp^2^	NS=S	25.24	3	3
138	C sp^2^	=NS2	−12.86	2	2
139	C sp^2^	O2=O	−9.60	10	10
140	C sp^2^	=OS2	6.53	1	1
141	C aromatic	B:C2	−47.51	5	5
142	C aromatic	H:C2	2.57	8600	1498
143	C aromatic	H:C:N	1.17	108	68
144	C aromatic	H:N2	−1.12	5	3
145	C aromatic	:C3	−1.60	481	153
146	C aromatic	C:C2	−2.58	2198	1062
147	C aromatic	C:C:N	5.44	46	38
148	C aromatic	:C2N	−0.38	524	389
149	C aromatic	:C2:N	−5.26	33	20
150	C aromatic	:C2N(+)	4.26	203	144
151	C aromatic	:C2O	2.82	853	532
152	C aromatic	:C2P	−2.68	12	5
153	C aromatic	:C2S	0.30	98	73
154	C aromatic	:C2Si	3.80	45	21
155	C aromatic	:C2F	4.24	150	69
156	C aromatic	:C2Cl	5.68	860	318
157	C aromatic	:C2Br	4.73	92	57
158	C aromatic	:C2J	6.30	26	19
159	C aromatic	:CN:N	5.87	28	27
160	C aromatic	:CN:N(+)	0.05	2	1
161	C aromatic	:C:NO	3.76	9	7
162	C aromatic	:C:NS	2.70	2	1
163	C aromatic	:C:NCl	9.38	8	8
164	C aromatic	N:N2	−9.59	85	40
165	C aromatic	:N2O	−5.16	4	2
166	C aromatic	:N2S	−2.43	5	5
167	C aromatic	:N2Cl	19.07	8	7
168	C sp	H#C	2.83	26	23
169	C sp	C#C	−0.52	183	83
170	C sp	=C2	7.54	4	4
171	C sp	C#N	2.66	120	94
172	C sp	#CSi	3.40	3	2
173	C sp	N#N	−16.19	1	1
174	C sp	=N2	23.07	1	1
175	C sp	#NO	6.78	10	4
176	C sp	=N=O	14.08	6	3
177	N sp^3^	H2C	9.39	34	21
178	N sp^3^	H2C(pi)	7.89	190	160
179	N sp^3^	H2N	0.92	5	5
180	N sp^3^	H2P	−16.37	1	1
181	N sp^3^	H2S	10.07	7	7
182	N sp^3^	HC2	−1.65	20	20
183	N sp^3^	HC2(pi)	−9.81	190	133
184	N sp^3^	HC2(2pi)	4.73	204	169
185	N sp^3^	HCN	−5.80	4	3
186	N sp^3^	HCN(pi)	−2.85	8	6
187	N sp^3^	HCN(+)(pi)	16.06	4	2
188	N sp^3^	HCN(2pi)	0.95	12	11
189	N sp^3^	HCO(pi)	30.19	1	1
190	N sp^3^	HCP	−6.83	2	2
191	N sp^3^	HCS	17.10	2	2
192	N sp^3^	HCS(pi)	9.38	22	22
193	N sp^3^	HSi2	1.67	7	2
194	N sp^3^	C3	−32.04	41	37
195	N sp^3^	C3(pi)	−17.08	137	97
196	N sp^3^	C3(2pi)	−12.64	136	108
197	N sp^3^	C3(3pi)	4.26	22	20
198	N sp^3^	C2N	−18.10	3	3
199	N sp^3^	C2N(pi)	−6.67	7	5
200	N sp^3^	C2N(+)(pi)	20.95	32	17
201	N sp^3^	C2N(2pi)	−3.87	15	14
202	N sp^3^	C2N(3pi)	1.17	6	6
203	N sp^3^	C2N(+)(2pi)	−0.16	12	12
204	N sp^3^	C2O	−41.10	5	5
205	N sp^3^	C2O(pi)	9.25	39	15
206	N sp^3^	C2O(2pi)	29.03	1	1
207	N sp^3^	C2P	7.24	1	1
208	N sp^3^	C2S	−25.22	3	3
209	N sp^3^	C2S(pi)	−22.07	1	1
210	N sp^3^	C2S(2pi)	−6.25	3	3
211	N sp^3^	CF2	−2.10	6	2
212	N(+) sp^3^	H2C2	4.33	19	19
213	N(+) sp^3^	C3O(-)	−33.09	1	1
214	N sp^2^	H=C	16.94	3	3
215	N sp^2^	C=C	−7.28	122	101
216	N sp^2^	C=N	−11.24	64	32
217	N sp^2^	C=N(+)	10.95	10	7
218	N sp^2^	=CN	−0.51	38	31
219	N sp^2^	=CO	0.98	32	31
220	N sp^2^	=CS	−4.17	3	2
221	N sp^2^	N=N	−0.32	10	6
222	N sp^2^	N=O	18.24	4	2
223	N aromatic	:C2	5.43	222	128
224	N aromatic	:C:N	−4.60	6	3
225	N(+) sp^2^	C=NO(-)	−19.90	4	4
226	N(+) sp^2^	CO=O(-)	1.45	248	163
227	N(+) sp^2^	=CO2(-)	−3.88	1	1
228	N(+) sp^2^	NO=O(-)	−1.33	48	31
229	N(+) sp^2^	O2=O(-)	1.85	7	5
230	N(+) sp	C#C(-)	10.24	1	1
231	N(+) sp	=N2(-)	2.76	6	3
232	O	HC	−2.00	452	254
233	O	HC(pi)	3.39	478	400
234	O	HN	0.63	36	12
235	O	HN(pi)	−1.02	19	19
236	O	HP	−8.39	2	1
237	O	HS	60.03	5	2
238	O	BC	0.00	5	5
239	O	BN	0.00	5	5
240	O	C2	−4.67	357	135
241	O	C2(pi)	−5.72	740	513
242	O	C2(2pi)	−3.04	267	217
243	O	CN	−20.33	4	4
244	O	CN(pi)	0.00	1	1
245	O	CN(2pi)	1.82	12	11
246	O	CN(+)(pi)	0.47	7	5
247	O	CO	1.80	8	4
248	O	CP	−6.11	47	25
249	O	CP(pi)	6.35	20	17
250	O	CS(pi)	1.11	3	3
251	O	CSi	−12.94	5	2
252	O	N2(2pi)			
253	O	N2(+)(2pi)	0.00	1	1
254	O	Si2	2.53	84	24
255	P3	C3	−6.01	3	2
256	P4	C3=O	−6.07	1	1
257	P4	C=OF2	−1.93	1	1
258	P4	C=OFCl	−4.92	1	1
259	P4	C=OCl2	6.84	1	1
260	P4	N2O=O	6.11	1	1
261	P4	NO2=O	−7.48	1	1
262	P4	NOS=S	6.11	1	1
263	P4	O3=O	−5.29	2	2
264	P4	O3=S	−3.13	13	12
265	P4	CO2=O	0.00	1	1
266	P4	CO2=S	7.66	2	2
267	P4	O2S=S	−5.52	7	7
268	S2	HC	−0.29	19	19
269	S2	HC(pi)	−11.91	2	2
270	S2	C2	−10.10	74	47
271	S2	C2(pi)	1.44	44	37
272	S2	C2(2pi)	8.54	74	60
273	S2	CN	0.00	3	3
274	S2	CN(pi)	5.57	1	1
275	S2	CS	1.49	8	4
276	S2	CS(pi)	0.18	6	4
277	S2	CP	0.00	8	8
278	S2	N2(2pi)	−3.71	1	1
279	S4	C2=O	−10.46	6	4
280	S4	C2=O2	−10.18	22	22
281	S4	CN=O2	1.23	31	31
282	S4	CO=O2	0.00	8	5
283	S4	C=OS	4.07	2	2
284	S4	N2=O2	4.49	2	2
285	Si	H3C	0.00	1	1
286	Si	HC2O	−77.65	3	3
287	Si	HCO2	18.28	1	1
288	Si	C4	−12.05	23	18
289	Si	C3O	−15.58	14	9
290	Si	C3Cl	−8.02	2	2
291	Si	C3Si	−6.42	6	3
292	Si	C2N2	0.00	7	2
293	Si	C2O2	1.03	75	18
294	Si	C2Cl2	−1.79	2	2
295	Si	C2Si2	−10.09	34	5
296	Si	CCl3	4.64	8	7
297	Si	O4	13.30	1	1
298	H	H Acceptor	6.31	153	128
299	Angle60		0.54	120	33
300	Angle90		2.37	138	29
301	Angle102		0.12	1131	342
302	Endocyclic bonds	No. of single bonds	−4.42	5302	680
A	Based on	Valid groups	188		2809
B	Goodness of fit	*R*^2^	0.8875		2701
C	Deviation	Average	12.33		2701
D	Deviation	Standard	16.72		2701
E	K-fold cv	K	10		2637
F	Goodness of fit	*Q*^2^	0.8727		2637
G	Deviation	Average (cv)	13.23		2637
H	Deviation	Standard (cv)	17.93		2637

**Table 5:** Atom Groups and their Contributions (in J/mol/K) for Total Phase-Change Entropy Calculations.

Entry	Atom Type	Neighbours	Contribution	Occurrences	Molecules
1	Const		60.14	2686	2686
2	C sp^3^	H3C	5.33	5873	2490
3	C sp^3^	H3N	16.05	12	6
4	C sp^3^	H3O	2.66	195	172
5	C sp^3^	H3Si	3.08	110	5
6	C sp^3^	H2C2	4.04	30,650	2478
7	C sp^3^	H2CN	−1.70	286	114
8	C sp^3^	H2CO	−0.01	3584	1901
9	C sp^3^	H2CS	−8.01	68	42
10	C sp^3^	H2CCl	−27.41	2	2
11	C sp^3^	H2CBr	−10.24	3	3
12	C sp^3^	H2CJ	30.88	1	1
13	C sp^3^	H2CSi	−2.48	6	3
14	C sp^3^	HC3	−9.84	1088	414
15	C sp^3^	HC2N	−17.47	4	4
16	C sp^3^	HC2O	−19.96	428	324
17	C sp^3^	HC2S	−42.59	18	18
18	C sp^3^	HC2Cl	−12.96	53	53
19	C sp^3^	HC2Br	6.97	4	4
20	C sp^3^	HCO2	7.19	34	28
21	C sp^3^	HCF2	−21.83	11	11
22	C sp^3^	C4	−0.53	212	120
23	C sp^3^	C3O	12.06	10	10
24	C sp^3^	C3F	−25.29	2	2
25	C sp^3^	C2F2	4.67	272	57
26	C sp^3^	CSF2	−1.17	5	5
27	C sp^3^	CF3	−8.30	67	54
28	C sp^3^	OF3	24.11	2	2
29	C sp^3^	SF3	−196.06	1	1
30	C sp^2^	H2=C	14.81	58	56
31	C sp^2^	HC=C	−2.97	946	440
32	C sp^2^	HC=N	−2.07	922	704
33	C sp^2^	HC=N(+)	32.39	9	9
34	C sp^2^	HC=O	15.32	6	6
35	C sp^2^	H=CN	−16.69	43	41
36	C sp^2^	H=CO	−2.30	28	28
37	C sp^2^	H=CS	−4.67	2	2
38	C sp^2^	H=NS	74.91	1	1
39	C sp^2^	C2=C	−13.21	186	160
40	C sp^2^	C2=N	9.17	17	17
41	C sp^2^	C2=O	2.80	266	202
42	C sp^2^	C=CN	2.69	28	21
43	C sp^2^	C=CO	−53.38	21	21
44	C sp^2^	C=CS	−5.66	340	150
45	C sp^2^	C=CF	31.70	10	5
46	C sp^2^	CN=N	−13.68	15	15
47	C sp^2^	CN=O	−1.75	326	171
48	C sp^2^	C=NO	−39.68	45	30
49	C sp^2^	CN=S	−6.95	8	6
50	C sp^2^	C=NS	38.49	105	77
51	C sp^2^	=CNS	−47.14	22	11
52	C sp^2^	CO=O	8.07	3115	1580
53	C sp^2^	=COS	128.10	5	5
54	C sp^2^	C=OS	5.46	91	81
55	C sp^2^	=CSCl	15.27	9	9
56	C sp^2^	=CSJ	10.36	2	2
57	C sp^2^	N=NS	−11.16	72	72
58	C sp^2^	NO=O	38.80	6	6
59	C sp^2^	=NOS	96.96	24	12
60	C sp^2^	O2=O	26.06	3	3
61	C aromatic	H:C2	3.37	28,602	2538
62	C aromatic	H:C:N	−0.02	151	82
63	C aromatic	H:C:N(+)	−9.49	12	6
64	C aromatic	:C3	−8.40	322	107
65	C aromatic	C:C2	−9.58	7933	2410
66	C aromatic	C:C:N	−38.40	89	61
67	C aromatic	:C2N	−13.66	1866	1124
68	C aromatic	:C2N(+)	−5.68	135	119
69	C aromatic	:C2:N	16.73	34	33
70	C aromatic	:C2O	−4.24	5711	2230
71	C aromatic	:C2S	−29.84	116	105
72	C aromatic	:C2Si	10.60	4	2
73	C aromatic	:C2F	4.38	525	266
74	C aromatic	:C2Cl	−3.87	197	151
75	C aromatic	:C2Br	2.55	24	23
76	C aromatic	:C2J	−35.42	9	9
77	C aromatic	C:N2	−43.07	27	21
78	C aromatic	:C:NCl	−51.42	2	2
79	C aromatic	N:N2	−17.88	6	3
80	C aromatic	:N2O	−31.16	4	4
81	C sp	H#C	15.40	1	1
82	C sp	C#C	−1.90	929	304
83	C sp	=C2	−15.98	9	9
84	C sp	C#N	4.72	229	212
85	C sp	#CO	29.96	2	1
86	C sp	=N=O	0.85	3	2
87	C sp	=N=S	15.48	42	42
88	C sp	#NS	7.49	26	26
89	N sp^3^	H2C	−12.08	5	5
90	N sp^3^	H2C(pi)	−66.66	6	6
91	N sp^3^	HC2(pi)	18.61	17	9
92	N sp^3^	HC2(2pi)	−4.58	233	143
93	N sp^3^	HCN(pi)	−6.87	6	3
94	N sp^3^	HCN(2pi)	42.99	12	12
95	N sp^3^	HCS(pi)	157.30	1	1
96	N sp^3^	C3	−75.12	10	10
97	N sp^3^	C3(pi)	−20.84	64	33
98	N sp^3^	C3(2pi)	8.12	34	25
99	N sp^3^	C3(3pi)	29.75	24	14
100	N sp^2^	C=C	14.07	1014	778
101	N sp^2^	C=N	9.88	722	295
102	N sp^2^	C=N(+)	8.87	32	32
103	N sp^2^	=CN	−40.91	206	94
104	N sp^2^	=CO	33.53	26	26
105	N aromatic	:C2	18.59	169	125
106	N aromatic	:C:N	17.07	12	3
107	N(+) sp^2^	CO=O(-)	0.77	94	78
108	N(+) sp^2^	C=CO(-)	−3.27	9	9
109	N(+) sp^2^	C=NO(-)	0.00	32	32
110	N(+) aromatic	:C2O(-)	23.39	6	6
111	O	HC	20.86	186	70
112	O	HC(pi)	16.46	202	156
113	O	C2	1.72	100	57
114	O	C2(pi)	−0.12	3901	2018
115	O	C2(2pi)	−2.52	2419	1340
116	O	CN(2pi)	−4.06	26	26
117	S2	HC(pi)	−10.11	2	2
118	S2	C2	12.90	18	18
119	S2	C2(pi)	14.58	55	42
120	S2	C2(2pi)	15.10	379	314
121	S4	CN=O2	−36.49	1	1
122	Si	C3Si	0.00	10	5
123	Si	C2Si2	−3.55	45	5
124	H	H Acceptor	−17.84	151	107
125	Angle60		0.00	0	0
126	Angle90		0.00	0	0
127	Angle102		7.37	513	138
128	Endocyclic bonds	No of single bonds	−1.14	3024	309
A	Based on	Valid groups	108		2686
B	Goodness of fit	*R*^2^	0.6094		2663
C	Deviation	Average	23.83		2663
D	Deviation	Standard	31.62		2663
E	K-fold cv	K	10		2643
F	Goodness of fit	*Q*^2^	0.5804		2643
G	Deviation	Average (cv)	24.65		2643
H	Deviation	Standard (cv)	32.79		2643

## Supplementary Materials

The following files are available online.

The entire set of experimental and calculated data of the heat-of-vaporization calculations is available under the name of “S1. Experimental and Calculated Heat-of-Vaporization Data Table.doc”; the corresponding list of compounds is added as an SD file named “S2. Compounds List for Heat-of-Vaporization Calculations.sdf” and the outliers list as an Excel file under the name “S3. Compounds List of Heat-of-Vaporization Outliers.xls”.

The list of compounds, their experimental and calculated data and 3D structures of the heat-of-sublimation calculations are available under the names of “S4. Experimental and Calculated Heat-of-Sublimation Data Table.doc” and “S5. Compounds List for Heat-of-Sublimation Calculations.sdf”. A list of the outliers has been added under the name of “S6. Compounds List of Heat-of-Sublimation Outliers.xls”.

The supplementary material also offers the list of molecules for the enthalpy-of-fusion calculations together with the experimental data under the file names “S7. Experimental and Calculated Heat-of-Fusion Data Table.doc” and “S8. Compounds List for Heat-of-Fusion Calculations.sdf”. The list of the outliers is available under the name of “S9. Compounds List of Heat-of-Fusion Outliers.xls”.

The heat-of-solvation result list, encompassing the molecule names, experimental and calculated data, are available under the name “S10. Experimental and Calculated Heat-of-Solvation Data Table.doc”; and the molecules list, encompassing their name and 3D coordinates is collected under the name “S11. Compounds List for Heat-of-Solvation Calculations.sdf”.

The list of compounds for entropy-of-fusion calculations, together with experimental and calculated data is provided under the name of “S12. Experimental and Calculated Entropy-of-Fusion Data Table.doc”. The related compounds’ 3D-structures are available in “S13. Compounds List for Entropy-of-Fusion Calculations.sdf”, the list of outliers in the Excel sheet called “S14. Compounds List of Entropy-of-Fusion Outliers.xls”.

The list of the experimental and calculated data for the total phase-change entropy calculations is provided under “S15. Experimental and Calculated Tpc-Entropy Data Table.doc”, the related compounds under the name “S16. Compounds List for Tpc-Entropy Calculations.sdf”, and the outliers list under the name “S17. Compounds List of Tpc-Entropy Outliers.xls”.

All the figures are available under the names given in the text as gif files, and the tables as doc files.

## References

[1] Naef R. (2015). A Generally Applicable Computer Algorithm Based on the Group Additivity Method for the Calculation of Seven Molecular Descriptors: Heat of Combustion, LogP_O/W_, LogS, Refractivity, Polarizability, Toxicity and LogBB of Organic Compounds; Scope and Limits of Applicability. Molecules.

[2] Ghose A.K., Crippen G.M. (1986). Atomic physicochemical parameters for three-dimensional structure-directed quantitative structure-activity relationships I. Partition coefficients as a measure of hydrophobicity. J. Comput. Chem..

[3] Ghose A.K., Pritchett A., Crippen G.M. (1988). Atomic physicochemical parameters for three dimensional structure directed quantitative structure-activity relationships III: Modeling hydrophobic interactions. J. Comput. Chem..

[4] Ghose A.K., Crippen G.M. (1987). Atomic Physicochemical parameters for three-dimensional-structure-directed quantitative structure-activity relationships. 2. Modeling dispersive and hydrophobic interactions. J. Chem. Inf. Comput. Sci..

[5] Miller K.J., Savchik J.A. (1979). A new empirical Method to calculate Average Molecular Polarizabilities. J. Am. Chem. Soc..

[6] Miller K.J. (1990). Additivity methods in molecular polarizability. J. Am. Chem. Soc..

[7] Sun H. (2004). A universal molecular descriptor system for prediction of LogP, LogS, LogBB, and absorption. J. Chem. Inf. Comput. Sci..

[8] Acree W.E., Chickos J.S. (2010). Phase Transition Enthalpy Measurements of Organic and Organometallic Compounds. Sublimation, Vaporization and Fusion Enthalpies from 1880 to 2010. J. Phys. Chem. Ref. Data.

[9] Roux M.V., Temprado M., Chickos J., Nagano Y. (2008). Critically Evaluated Thermo-chemical Properties of Polycyclic Aromatic Hydrocarbons. J. Phys. Chem. Ref. Data.

[10] Chickos J., Wang T., Sharma E. (2008). Hypothetical Thermodynamic Properties: Vapor Pressures and Vaporization Enthalpies of the Even *n*-Alkanes from C40 to C76 at *T* = 298.15 K by Correlation-Gas Chromatography. Are the Vaporization Enthalpies a Linear Function of Carbon Number?. J. Chem. Eng. Data.

[11] Chickos J., Lipkind D. (2008). Hypothetical Thermodynamic Properties: Vapor Pressures and Vaporization Enthalpies of the Even *n*-Alkanes from C78 to C92 at *T* = 298.15 K by Correlation-Gas Chromatography. J. Chem. Eng. Data.

[12] Chickos J., Hanshaw W. (2004). Vapor Pressures and Vaporization Enthalpies of the *n*-Alkanes from C21 to C30 at *T* = 298.15 K by Correlation Gas Chromatography. J. Chem. Eng. Data.

[13] Chickos J., Hanshaw W. (2004). Vapor Pressures and Vaporization Enthalpies of the *n*-Alkanes from C31 to C38 at *T* = 298.15 K by Correlation Gas Chromatography. J. Chem. Eng. Data.

[14] Wilson J., Gobble C., Chickos J. (2015). Vaporization, Sublimation, and Fusion Enthalpies of Some Saturated and Unsaturated Long Chain Fatty Acids by Correlation Gas Chromatography. J. Chem. Eng. Data.

[15] Abraham M.H. (1993). Scales of Hydrogen-bonding: Their Construction and Application to Physicochemical and Biochemical Processes. Chem. Rev..

[16] Abraham M.H., Chadha H.S., Whinting G.S., Mitchell R.C. (1994). Hydrogen-bonding. 32. An Analysis of Water-Octanol and Water-Alkane Partitioning and the Δlog*P* Parameter of Seiler. J. Pharm. Sci..

[17] Abraham M.H., Zissimos A.M., Acree W.E. (2001). Partition of solutes from the gas phase and from water to wet and dry di-*n*-butyl Ether: A linear free energy relationship analysis. Phys. Chem. Chem. Phys..

[18] Abraham M.H., Le J. (1999). The Correlation and Prediction of the Solubility of Compounds in Water using an amended Solvation Energy Relationship. J. Pharm. Sci..

[19] Jover J., Bosque R., Sales J. (2004). Determination of Abraham Solute Parameters from Molecular Structure. J. Chem. Inf. Comput. Sci..

[20] Cabani S., Gianni P., Mollica V., Lepori L. (1981). Group contributions to the thermodynamic properties of non-ionic organic solutes in dilute aqueous solution. J. Sol. Chem..

[21] Chickos J.S., Acree W.E. Jr., Liebman J.F. (1999). Estimating Solid-Liquid Phase Change Enthalpies and Entropies. J. Phys. Chem. Ref. Data.

[22] Acree W.E., Chickos J.S. (2006). Phase Change Enthalpies and Entropies of Liquid Crystals. J. Phys. Chem. Ref. Data.

[23] Almeida A.R.R., Monte M.J.S. (2016). Vapour pressures and phase transition properties of four substituted acetophenones. J. Chem. Thermodyn..

[24] Gobble C., Vikman J., Chickos J.S. (2014). Evaluation of the Vaporization Enthalpies and Liquid Vapor Pressures of (*R*)-Deprenyl, (*S*)-Benzphetamine, Alverine, and a Series of Aliphatic Tertiary Amines by Correlation Gas Chromatography at *T*/K = 298.15. J. Chem. Eng. Data.

[25] Miroshnichenko E.A., Kon’kova T.S., Pashchenko L.L., Matyushin Y.N., Inozemtsev Y.O., Tartakovskii V.A. (2016). Energy characteristics of nitrooxazolidines and their radicals. Russ. Chem. Bull. Int. Ed..

[26] Emel’yanenko V.N., Zaitseva K.V., Nagrimanov R.N., Solomonov B.N., Verevkin S.P. (2016). Benchmark Thermondynamic Properties of Methyl- and Methoxy-Benzamides: Comprehensive Experimental and Theoretical Study. J. Phys. Chem. A.

[27] Gobble C., Gutterman A., Chickos J.S. (2013). Some thermodynamic properties of benzocaine. Struct. Chem..

[28] Keating L., Harris H.H., Chickos J.S. (2017). Vapor pressures and vaporization enthalpy of (−) α-bisabolol and (*dl*) menthol by correlation gas chromatography. J. Chem. Thermodyn..

[29] Sanchez-Buläs T., Cruz-Väsquez O., Hernändez-Obregon J., Rojas A. (2017). Enthalpies of fusion, vaporisation and sublimation of crown ethers determined by thermogravimetry and differential scanning calorimetry. Thermochim. Acta.

[30] Panneerselvam K., Anthony M.P., Srinivasan T.G., Rao P.R.V. (2009). Enthalpies of vaporization of *N*,*N*-dialkyl monamides at 298.15K. Thermochim. Acta.

[31] Gobble C., Walker B., Chickos J.S. (2016). The Vaporization Enthalpy and Vapor Pressure of Fenpropidin and Phencyclidine (PCP) at *T*/K = 298.15 by Correlation Gas Chromatography. J. Chem. Eng. Data.

[32] Kozlovskiy M., Gobble C., Chickos J.S. (2015). Vapor pressures and vaporization enthalpies of a series of esters used in flavors by correlation gas chromatography. J. Chem. Thermodyn..

[33] Costa J.C.S., Lima C.F.R.A.C., Mendes A., Santos L.M.N.B.F. (2016). Fluorination effect on the thermodynamic properties of long-chain hydrocarbons and alcohols. J. Chem. Thermodyn..

[34] Simmons D., Chickos J. (2017). Enthalpy of vaporization and vapor pressure of whiskey lactone and menthalactone by correlation gas chromatography. J. Chem. Thermodyn..

[35] Oliveira J.A.S.A., Oliveira T.S.M., Gaspar A., Borges F., Ribeiro da Silva M.D.M.C., Monte M.J.S. (2016). Study on the volatility of halogenated fluorenes. Chemosphere.

[36] Maxwell R., Chickos J. (2012). An Examination of the Thermodynamics of Fusion, Vaporization, and Sublimation of Ibuprofen and Naproxen by Correlation Gas Chromatography. J. Pharm. Sci..

[37] Mori M., Rath N., Gobble C., Chickos J. (2016). Vaporization, Sublimation Enthalpy, and Crystal Structures of Imidazo[1,2-*a*]pyrazine and Phthalazine. J. Chem. Eng. Data.

[38] Goodrich S., Hasanovic J., Gobble C., Chickos J.S. (2016). Vaporization Enthalpies and Vapor Pressures of Some Insect Pheromones by Correlation Gas Chromatography. J. Chem. Eng. Data.

[39] Freitas V.L.S., Silva C.A.O., Paiva M.A.T., Ribeiro da Silva M.D.M.C. (2017). Energetic effects of alkyl groups (methyl and ethyl) on the nitrogen of the morpholine structure. J. Therm. Anal. Calorim..

[40] Althoff M.A., Grieger K., Härtel M.A.C., Karaghiosoff K.L., Klapötke T.M., Metzulat M. (2017). Application of the Transpiration Method to Determine the Vapor Pressure and Related Physico-Chemical Data of Low Volatile, Thermolabile, and Toxic Organo(thio)phosphates. J. Phys. Chem. A.

[41] Gobble C., Chickos J., Verevkin S.P. (2014). Vapor Pressures and Vaporization Enthalpies of a Series of Dialkyl Phthalates by Correlation Gas Chromatography. J. Chem. Eng. Data.

[42] Brunetti B., Lapi A., Ciprioti S.V. (2016). Thermodynamic study on six tricyclic nitrogen heterocyclic compounds by thermal analysis and effusion techniques. Thermochim. Acta.

[43] Emel’yanenko V.N., Kaliner M., Strassner T., Verevkin S.P. (2017). Thermochemical properties of different 1-(*R*-phenyl)-1*H*-imidazoles. Fluid Phase Equilib..

[44] Antón V., Artigas H., Muñoz-Embid J., Artal M., Lafuente C. (2017). Thermophysical study of 2-acetylthiophene: Experimental and modelled results. Fluid Phase Equilib..

[45] Portnova S.V., Krasnykh E.L., Levanova S.V. (2016). Temperature Dependences of Saturated Vapor Pressure and the Enthalpy of Vaporization of *n*-Pentyl Esters of Dicarboxylic Acids. Russ. J. Phys. Chem. A.

[46] Lepori L., Matteoli E., Gianni P. (2017). Vapor Pressure and Its Temperature Dependence of 28 Organic Compounds: Cyclic Amines, Cyclic Ethers, and Cyclic and Open Chain Secondary Alcohols. J. Chem. Eng. Data.

[47] Lima C.F.R.A.C., Rodrigues A.S.M.C., Santos L.M.N.B.F. (2017). Effect of Confined Hindrance in Polyphenylbenzenes. J. Phys. Chem. A.

[48] Abboud J.-L.M., Alkorta I., Davalos J.Z., Koppel I.A., Koppel I., Lenoir D., Martínez S., Mishima M. (2016). The Thermodynamic Stability of Adamantylideneadamantane and Its Proton- and Electron-Exchanges. Comparison with Simple Alkenes. Bull. Chem. Soc. Jpn..

[49] Silva A.L.R., Ribeira da Silva M.D.M.C. (2017). Comprehensive Thermochemical Study of Cyclic Five- and Six-Membered *N*,*N′*-Thioureas. J. Chem. Eng. Data.

[50] Carvalho T.M.T., Amaral L.M.P.F., Morais V.M.F., Ribeiro da Silva M.D.M.C. (2017). Energetic Effect of the Carboxylic Acid Functional Group in Indole Derivatives. J. Phys. Chem. A.

[51] Freitas V.L.S., Lima A.C.M.O., Sapei E., Ribeiro da Silva M.D.M.C. (2016). Comprehensive thermophysical and thermochemical studies of vanillyl alcohol. J. Chem. Thermodyn..

[52] Lopes C.S.D., Agapito F., Bernardes C.E.S., Minas da Piedade M.E. (2017). Thermochemistry of 4-HOC_6_H_4_COR (R = H, CH_3_, C_2_H_5_, *n*-C_3_H_7_, *n*-C_4_H_9_, *n*-C_5_H_11_, and *n*-C_6_H_13_) compounds. J. Chem. Thermodyn..

[53] Emel’yanenko V.N., Nagrimanov R.N., Solomonov B.N., Verevkin S.P. (2016). Adamantanes: Benchmarking of thermochemical properties. J. Chem. Thermodyn..

[54] Nagrimanov R.N., Solomonov B.N., Emel’yanenko V.N., Verevkin S.P. (2016). Six-membered ring aliphatic compounds: A search for regularities in phase transitions. Thermochim. Acta.

[55] Blokhina S., Sharapova A., Ol’khovich M., Perlovich G. (2017). Sublimation thermodynamics of four fluoroquinolone antimicrobial compounds. J. Chem. Thermodyn..

[56] Flores H., Ledo J.M., Hernandez-Pérez J.M., Camarillo E.A., Sandoval-Lira J., Amador M.P. (2016). Thermochemical and theoretical study of 2-oxazolidinone and 3-acetyl-2-oxazolidinone. J. Chem. Thermodyn..

[57] Emel’yanenko V.N., Nagrimanov R.N., Verevkin S.P. (2017). Benchmarking thermochemical experiments and calculations of nitrogen-containing substituted adamantanes. J. Thermal. Anal. Calorim..

[58] Oliveira J.A.S.A., Freitas V.L.S., Notario R., da Silva M.D.M.C.R., Monte M.J.S. (2016). Thermodynamic properties of 2,7-di-*tert*-butylfluorene—An experimental and computational study. J. Chem. Thermodyn..

[59] Carvalho T.M.T., Amaral L.M.P.F., Morais V.M.F., Ribeiro da Silva M.D.M.C. (2016). Calorimetric and computational studies for three nitroimidazole isomers. J. Chem. Thermodyn..

[60] Chickos J.S., Acree W.E. (2009). Total phase change entropies and enthalpies. An update on fusion enthalpies and their estimation. Thermochim. Acta.

[61] Wang L., Xing C., Zhao L., Xu L., Liu G. (2017). Measurement and correlation of solubility of 2-chloro-3-(trifluoromethyl)pyridine in pure solvents and ethanol + *n*-propanol mixtures. J. Mol. Liq..

[62] Guenthner A.J., Ramirez S.M., Ford M.D., Soto D., Boatz J.A., Ghiassi K.B., Mabry J.M. (2016). Organic Crystal Engineering of Thermosetting Cyanate Ester Monomers: Influence of Structure on Melting Point. Cryst. Growth Des..

[63] Trache D., Khimeche K., Dahmani A. (2013). Study of (Solid–Liquid) Phase Equilibria for Mixtures of Energetic Material Stabilizers and Prediction for Their Subsequent Performance. Int. J. Thermophys..

[64] Eckert K.-A., Dasgupta S., Selge B., Ay P. (2016). Solid liquid phase diagrams of binary fatty acid mixtures—Palmitic/stearic with oleic/linoleic/linolenic acid mixture. Thermochim. Acta.

[65] Blokhina S., Sharapova A., Ol’khovich M., Volkova T., Perlovich G. (2015). Studying the sublimation thermodynamics of ethionamide and pyridinecarbothioamide isomers by transpiration method. Thermochim. Acta.

[66] Forte A., Melo C.I., Bogel-Lukasik R., Bogel-Lukasik E. (2012). A favourable solubility of isoniazid, an antitubercular antibiotic drug, in alternative solvents. Fluid Phase Equil..

[67] Carletta A., Meinguet C., Wouters J., Tilborg A. (2015). Solid-State Investigation of Polymorphism and Tautomerism of Phenylthiazole-thione: A Combined Crystallographic, Calorimetric, and Theoretical Survey. Cryst. Growth Des..

[68] Leitner J., Jurik S. (2017). DSC study and thermodynamic modelling of the system paracetamol–*o*-acetylsalicylic acid. J. Therm. Anal. Calorim..

[69] Mintz C., Clark M., Acree W.E., Abraham M.H. (2007). Enthalpy of Solvation Correlations for Gaseous Solutes Dissolved in Water and in 1-Octanol Based on the Abraham Model. J. Chem. Inf. Model..

[70] Catalan J., Couto A., Gomez J., Saiz J.L., Laynez J. (1992). Towards a solvent acidity scale: The calorimetry of the *N*-methyl imidazole probe. J. Chem. Soc. Perkin Trans. 2.

[71] Spencer J.N., Hovick J.W. (1988). Solvation of urea and methyl-substituted ureas by water and DMF. Can. J. Chem..

[72] Gatta G.D., Badea E. (2007). Thermodynamics of Solvation of Urea and Some Monosubstituted *N*-Alkylureas in Water at 298.15 K. J. Chem. Eng. Data.

[73] Rouw A., Somsen G. (1982). Solvation and Hydrophobic Hydration of Alkyl-substituted Ureas and Amides in *N*,*N*-Dimethylformamide + Water Mixtures. J. Chem. Soc. Faraday Trans. 1.

[74] Badea E., della Gatta G., Jozwiak M., Giancola C. (2011). Hydration of Thiourea and Mono-, Di-, and Tetra-*N*-Alkylthioureas at Infinite Dilution: A Thermodynamic Study at a Temperature of 298.15 K. J. Chem. Eng. Data.

[75] Stimson E.R., Schrier E.E. (1974). Calorimetric Investigation of Salt-Amide Interactions in Aqueous Solution. J. Chem. Eng. Data.

[76] Batov D.V., Zaichikov A.M. (2003). Group Contributions to the Enthalpy Characteristics of Solutions of Formic and Acetic Acid Amides in Water-1,2-Propanediol Mixtures. Russ. J. Gen. Chem..

[77] Starzewski P., Wadsö I., Zielenkiewicz W. (1984). Enthalpies of vaporization of some *N*-alkylamides at 298.15 K. J. Chem. Thermodyn..

[78] Morgan K.M., Kopp D.A. (1998). Solvent effects on the stability of simple secondary amides. J. Chem. Soc. Perkin Trans. 2.

[79] Teplitsky A.B., Glukhova O.T., Sukhodub L.F., Yanson I.K., Zielenkiewicz A., Zielenkiewicz W., Kosinski J., Wierzchowski K.L. (1982). Thermochemistry of aqueous Solutions of alkylated Nucleic Acid Bases. IV. Enthalpies of 5-Alkyluracils. Biophys. Chem..

[80] Zielenkiewicz W., Szterner P., Kaminski M. (2003). Vapor Pressures, Molar Enthalpies of Sublimation, and Molar Enthalpies of Solution in Water of Selected Amino Derivatives of Uracil and 5-Nitrouracil. J. Chem. Eng. Data.

[81] Zielenkiewicz W., Szterner P. (2004). Vapor Pressures, Molar Enthalpies of Sublimation, and Molar Enthalpies of Solution in Water of 5-(Trifluoromethyl)uracil. J. Chem. Eng. Data.

[82] Zielenkiewicz W., Szterner P. (2005). Thermodynamic Investigation of Uracil and Its Halo Derivatives. Enthalpies of Solution and Solvation in Methanol. J. Chem. Eng. Data.

[83] Zhou Y., Wang J., Fang B., Guo N., Xiao Y., Hao H., Bao Y., Huang X. (2017). Solubility and dissolution thermodynamic properties of 2-Cyano-4′-methylbiphenyl in binary solvent mixtures. J. Mol. Liq..

[84] Zhang Q.-A., Du C.-J. (2015). Solubility of cyclohexyl-phosphoramidic acid diphenyl ester in selected solvents. J. Mol. Liq..

[85] Zhao F.-Q., Pei C., Hu R.-Z., Yang L., Zhang Z.-Z., Zhou Y.-S., Yang X.-W., Yin G., Gao S.L., Shi Q.-Z. (2004). Thermochemical properties and non-isothermal decomposition reaction kinetics of 3,4-dinitrofurazanfuroxan (DNTF). J. Hazard. Mat..

