# Supplementary material for: Calculation of Five Thermodynamic Molecular Descriptors by Means of a General Computer Algorithm Based on the Group-Additivity Method: Standard Enthalpies of Vaporization, Sublimation and Solvation, and Entropy of Fusion of Ordinary Organic Molecules and Total Phase-Change Entropy of Liquid Crystals
Source: Molecules. 2017 Jun 25;22(7):1059. doi: 10.3390/molecules22071059 (PMC6152037; doi:10.3390/molecules22071059)
Supplement: Supplementary file 1 [file molecules-22-01059-s001.zip › S1. Experimental and Calculated Heat-of-Vaporization Data Table.doc]

|  | Molecules sorted by deviation |  |  |  |  |
| --- | --- | --- | --- | --- | --- |
| No. | Molecule name | ΔH°(vap) exp | ΔH°(vap) calc | ΔH°(vap) test | Deviation |
|  |  |  |  |  |  |
| 1 | 1,4-Diethylbenzene | 52.50 | 52.50 | 52.50 | 0.00 |
| 2 | 1-Ethylimidazole | 57.50 | 57.80 | 57.50 | 0.00 |
| 3 | 1-Tetralone | 65.00 | 65.30 | 65.00 | 0.00 |
| 4 | 2-Chloro-1,1,2-trifluoroethyl propyl ether | 41.00 | 40.50 | 41.00 | 0.00 |
| 5 | Dimethylvinylcarbinol | 48.50 | 48.70 | 48.50 | 0.00 |
| 6 | 2-Methylbenzaldehyde | 52.50 | 52.30 | 52.50 | 0.00 |
| 7 | 3,4-Dimethyl-1,2,5-oxadiazole | 51.00 | 51.00 |  | 0.00 |
| 8 | Ethyl propyl sulfide | 40.00 | 39.90 | 40.00 | 0.00 |
| 9 | s-Butylnitrite | 35.00 | 35.00 | 35.00 | 0.00 |
| 10 | trans-2,5-Dimethyl-3-hexene | 37.50 | 37.50 | 37.50 | 0.00 |
| 11 | 2,3-Dihydrofuran | 30.80 | 31.10 | 30.80 | 0.00 |
| 12 | Ethyl vinyl ether | 26.60 | 26.60 | 26.60 | 0.00 |
| 13 | 1-Hexyne | 33.60 | 33.70 | 33.60 | 0.00 |
| 14 | 2,3-Dimethyl-2-pentene | 35.60 | 35.60 | 35.60 | 0.00 |
| 15 | 2,5-Dimethylhexane | 37.90 | 38.00 | 37.90 | 0.00 |
| 16 | 3,3-Dimethylheptane | 42.60 | 42.30 | 42.60 | 0.00 |
| 17 | Cyclopentanecarbonitrile | 48.10 | 48.20 | 48.10 | 0.00 |
| 18 | Methyl 2-thiophenecarboxylate | 57.60 | 57.50 | 57.60 | 0.00 |
| 19 | Amyl acetate | 48.60 | 48.50 | 48.60 | 0.00 |
| 20 | Trimethylphosphine | 28.90 | 28.90 |  | 0.00 |
| 21 | Neohexane | 27.70 | 28.00 | 27.70 | 0.00 |
| 22 | 2-Butyne | 26.70 | 26.60 | 26.70 | 0.00 |
| 23 | 3-Methylnonane | 50.20 | 50.30 | 50.20 | 0.00 |
| 24 | Diamylamine | 61.20 | 61.40 | 61.20 | 0.00 |
| 25 | Dipropyl sulfide | 44.20 | 44.60 | 44.20 | 0.00 |
| 26 | 4-Methylpyridine | 44.70 | 44.60 | 44.70 | 0.00 |
| 27 | n-Butyl acetate | 43.70 | 43.80 | 43.70 | 0.00 |
| 28 | (E) 11-Hexadecenal | 89.20 | 89.20 | 89.20 | 0.00 |
| 29 | 1-Nonanol | 74.70 | 74.70 | 74.70 | 0.00 |
| 30 | 2,2',5-Trichlorobiphenyl | 80.20 | 80.00 | 80.20 | 0.00 |
| 31 | 2,4-Dichlorobiphenyl | 75.30 | 75.20 | 75.30 | 0.00 |
| 32 | Decanenitrile | 66.30 | 66.40 | 66.30 | 0.00 |
| 33 | 3,3-Diethylhexane | 47.30 | 47.10 | 47.30 | 0.00 |
| 34 | 3,5-Di-t-butyltoluene | 63.30 | 62.70 | 63.30 | 0.00 |
| 35 | 3-Methyl-1-hexene | 34.30 | 34.30 | 34.30 | 0.00 |
| 36 | Benzo[b]furan | 48.80 | 48.60 | 48.80 | 0.00 |
| 37 | Bromobenzene | 44.30 | 44.30 | 44.30 | 0.00 |
| 38 | cis-2-Methylcyclohexanol | 63.30 | 63.40 | 63.30 | 0.00 |
| 39 | 2-Methyl-1-propanol | 48.80 | 48.60 | 48.80 | 0.00 |
| 40 | (E) 9-Dodecenal | 70.40 | 70.50 | 70.40 | 0.00 |
| 41 | (Z) 13-Pentadecenyl acetate | 95.90 | 95.90 | 95.90 | 0.00 |
| 42 | (Z) 9-Dodecenal | 70.40 | 70.50 | 70.40 | 0.00 |
| 43 | Arachic alcohol | 125.90 | 126.10 | 125.90 | 0.00 |
| 44 | 2-Decanone | 60.90 | 61.00 | 60.90 | 0.00 |
| 45 | 3-(2-Chloro-1,1,2-trifluoroethoxy)-1,1,2,2-tetrafluoropropane | 44.40 | 44.60 | 44.40 | 0.00 |
| 46 | Dibenzofuran | 65.40 | 65.20 | 65.40 | 0.00 |
| 47 | Hexanenitrile | 47.90 | 47.70 | 47.90 | 0.00 |
| 48 | Methyl 4-chlorobenzoate | 62.40 | 62.50 | 62.40 | 0.00 |
| 49 | trans-2,3-Dimethyl-3-hexene | 38.90 | 38.80 | 38.90 | 0.00 |
| 50 | Di-t-butyl succinate | 68.60 | 68.60 | 68.60 | 0.00 |
| 51 | Tridecylbenzene | 94.60 | 94.90 | 94.60 | 0.00 |
| 52 | (E) 10-Tetradecenal | 79.80 | 79.90 | 79.80 | 0.00 |
| 53 | (Z) 3-Dodecen-1-ol | 89.30 | 89.40 | 89.30 | 0.00 |
| 54 | 1-Benzyl-2-piperidone | 91.30 | 90.90 | 91.30 | 0.00 |
| 55 | 2-Methylbenzoic acid | 79.30 | 79.00 | 79.30 | 0.00 |
| 56 | 1-Octadecanol | 116.80 | 116.70 | 116.80 | 0.00 |
| 57 | N-Methylpyrrolidine | 33.16 | 33.10 | 33.10 | 0.06 |
| 58 | (E) 4-Tetradecenal | 79.90 | 79.90 | 79.80 | 0.10 |
| 59 | 2,2',5,6'-Tetrachlorobiphenyl | 84.90 | 84.70 | 84.80 | 0.10 |
| 60 | 4-Chlorophenol | 65.70 | 65.60 | 65.60 | 0.10 |
| 61 | Demeton | 78.70 | 78.70 | 78.60 | 0.10 |
| 62 | 2,2,4,6,6-Pentamethylheptane | 49.00 | 47.90 | 48.90 | 0.10 |
| 63 | Allyl-t-butylsulfide | 44.00 | 43.70 | 43.90 | 0.10 |
| 64 | cis-2-Hexahydroindanone | 57.50 | 57.30 | 57.40 | 0.10 |
| 65 | Di-s-butylsulfide | 47.00 | 46.90 | 46.90 | 0.10 |
| 66 | Diisobutyl ketone | 51.00 | 50.90 | 50.90 | 0.10 |
| 67 | Pivalonitrile | 37.40 | 37.10 | 37.30 | 0.10 |
| 68 | 4-Ethyl-1-hexene | 38.90 | 38.90 | 38.80 | 0.10 |
| 69 | 4-Methyl-1-heptene | 38.90 | 38.90 | 38.80 | 0.10 |
| 70 | 5-Methyl-1-heptene | 38.90 | 38.90 | 38.80 | 0.10 |
| 71 | cis-2,3-Dimethyl-3-hexene | 38.90 | 38.80 | 38.80 | 0.10 |
| 72 | 1-t-Butylimidazole | 61.90 | 61.50 | 61.80 | 0.10 |
| 73 | Styrene | 43.90 | 43.70 | 43.80 | 0.10 |
| 74 | trans-3,5-Dimethyl-2-hexene | 38.90 | 38.80 | 38.80 | 0.10 |
| 75 | 1,4-Difluorobenzene | 35.80 | 36.10 | 35.70 | 0.10 |
| 76 | 2,2-Dimethylheptane | 42.30 | 42.30 | 42.20 | 0.10 |
| 77 | alpha-Ethylstyrene | 51.80 | 51.60 | 51.70 | 0.10 |
| 78 | alpha-Isopropylstyrene | 53.30 | 53.40 | 53.20 | 0.10 |
| 79 | (Z) 12-Hexadecenal | 89.30 | 89.20 | 89.20 | 0.10 |
| 80 | 3,4-Dichlorophenol | 70.80 | 70.40 | 70.70 | 0.10 |
| 81 | Ethyl methyl sulfide | 31.50 | 31.10 | 31.40 | 0.10 |
| 82 | 1,2-Difluorobenzene | 36.20 | 36.10 | 36.10 | 0.10 |
| 83 | 1,2-Pentadiene | 28.70 | 28.70 | 28.60 | 0.10 |
| 84 | 2,4,4-Trimethyl-2-nitropentane | 54.70 | 54.60 | 54.60 | 0.10 |
| 85 | 5-Methyl-1-hexene | 34.30 | 34.30 | 34.40 | -0.10 |
| 86 | Methyl acetate | 32.30 | 32.30 | 32.40 | -0.10 |
| 87 | cis-2,2-Dimethyl-3-hexene | 37.20 | 37.00 | 37.10 | 0.10 |
| 88 | N-Methylbenzaldehydeimine | 51.20 | 51.10 | 51.10 | 0.10 |
| 89 | Tripropylamine | 46.20 | 46.30 | 46.10 | 0.10 |
| 90 | (E) 11-Tridecenyl acetate | 86.40 | 86.50 | 86.50 | -0.10 |
| 91 | (Z) 11-Tridecenyl acetate | 86.40 | 86.50 | 86.50 | -0.10 |
| 92 | 1,1,4,7-Tetramethylindane | 61.40 | 61.70 | 61.50 | -0.10 |
| 93 | 1,8-Diazabicyclo[5.4.0]undec-7-ene | 68.40 | 69.70 | 68.50 | -0.10 |
| 94 | 1-(2-Fluorophenyl)-1H-imidazole | 73.90 | 73.10 | 74.00 | -0.10 |
| 95 | Isobutyl bromide | 34.90 | 35.00 | 35.00 | -0.10 |
| 96 | Enanthic acid | 72.90 | 73.20 | 73.00 | -0.10 |
| 97 | Isooctane | 34.90 | 34.80 | 35.00 | -0.10 |
| 98 | 1,4-Dioxaspiro[4.5]decane | 50.60 | 51.00 | 50.50 | 0.10 |
| 99 | 2-Methyl-1-butanethiol | 39.90 | 40.00 | 40.00 | -0.10 |
| 100 | 2-Methyl-2-hexanol | 58.60 | 58.60 | 58.50 | 0.10 |
| 101 | 2-Methylbenzoxazole | 57.10 | 57.90 | 57.00 | 0.10 |
| 102 | 3-Hexanol | 58.60 | 58.30 | 58.50 | 0.10 |
| 103 | Cyclopentyl methyl sulfide | 45.10 | 44.80 | 45.00 | 0.10 |
| 104 | Diisopropylamine | 34.60 | 34.90 | 34.50 | 0.10 |
| 105 | N,N-Dimethylcyclohexylamine | 45.60 | 45.50 | 45.50 | 0.10 |
| 106 | Pelargonic acid | 82.40 | 82.50 | 82.50 | -0.10 |
| 107 | Undecanonitrile | 71.10 | 71.10 | 71.00 | 0.10 |
| 108 | 3,3-Dimethyl-1-butene | 26.60 | 26.50 | 26.50 | 0.10 |
| 109 | cis-2,4-Dimethyl-1,3-dioxane | 40.00 | 39.90 | 40.10 | -0.10 |
| 110 | 3,3-Dimethylhexane | 37.50 | 37.50 | 37.40 | 0.10 |
| 111 | 4-Heptanol | 62.50 | 63.00 | 62.60 | -0.10 |
| 112 | Caprylic aldehyde | 51.00 | 51.10 | 51.10 | -0.10 |
| 113 | 1,2-Dichlorobutane | 40.10 | 40.10 | 40.20 | -0.10 |
| 114 | 2,5-Dimethylpyrazine | 47.60 | 47.90 | 47.70 | -0.10 |
| 115 | 2-Chloro-1,1,2-trifluoroethyl butyl ether | 45.10 | 45.20 | 45.20 | -0.10 |
| 116 | 2-Pentanol | 53.60 | 53.60 | 53.70 | -0.10 |
| 117 | 3,3'-Dimethylbiphenyl | 71.90 | 72.00 | 71.80 | 0.10 |
| 118 | 3-Octanol | 67.90 | 67.60 | 67.80 | 0.10 |
| 119 | Cumylhydroperoxide | 69.90 | 70.40 | 69.80 | 0.10 |
| 120 | Ethyl decyl ether | 65.90 | 65.80 | 65.80 | 0.10 |
| 121 | 9H-Fluoren-9-one | 80.60 | 80.50 | 80.70 | -0.10 |
| 122 | 1,3-Diisopropylbenzene | 56.20 | 56.30 | 56.30 | -0.10 |
| 123 | 2-Methyl-1,2-propanediamine | 47.20 | 47.00 | 47.30 | -0.10 |
| 124 | 4-Ethyl-4-methylheptane | 47.20 | 47.10 | 47.30 | -0.10 |
| 125 | Benzylamine | 54.20 | 54.40 | 54.30 | -0.10 |
| 126 | Chlorobenzene | 41.20 | 41.20 | 41.30 | -0.10 |
| 127 | Dipropyl disulfide | 54.20 | 54.40 | 54.30 | -0.10 |
| 128 | Isobutyraldehyde isobutylhydrazone | 57.20 | 57.30 |  | -0.10 |
| 129 | Amyl formate | 45.20 | 47.00 | 45.30 | -0.10 |
| 130 | (E) 13-Octadecenyl acetate | 109.80 | 109.90 | 109.90 | -0.10 |
| 131 | 4-Phenylpyrimidine | 68.80 | 68.60 | 68.90 | -0.10 |
| 132 | Dihexylamine | 70.80 | 70.80 | 70.90 | -0.10 |
| 133 | Diisopropyl glutarate | 72.80 | 72.70 | 72.90 | -0.10 |
| 134 | Methyl Z 15-tetracosenoate | 135.30 | 135.80 | 135.40 | -0.10 |
| 135 | t-Pentylbenzene | 52.30 | 52.10 | 52.40 | -0.10 |
| 136 | 1,1-Difluoro-3-phenylpropane | 53.30 | 53.50 | 53.40 | -0.10 |
| 137 | 2,6-Dichlorostyrene | 53.80 | 53.90 | 53.90 | -0.10 |
| 138 | 3-Buten-1-ol | 50.80 | 50.80 | 50.90 | -0.10 |
| 139 | 3-Methyl-2-butenyl acetate | 47.80 | 47.80 | 47.90 | -0.10 |
| 140 | Benzylbromide | 53.30 | 53.80 | 53.40 | -0.10 |
| 141 | Chloropentafluorobenzene | 41.30 | 40.30 | 41.40 | -0.10 |
| 142 | Di-t-butyldisulfide | 53.80 | 53.70 | 53.90 | -0.10 |
| 143 | Diethyl disulfide | 44.80 | 45.00 | 44.90 | -0.10 |
| 144 | 2-Methylpropanoic acid | 56.30 | 56.50 | 56.40 | -0.10 |
| 145 | Pentyl 3-methylbut-2-enoate | 61.80 | 61.80 | 61.90 | -0.10 |
| 146 | syn-Tricyclo[4.2.0.02.5]octane | 41.80 | 42.20 | 41.90 | -0.10 |
| 147 | trans-2-Methylcyclohexanol | 63.30 | 63.40 | 63.40 | -0.10 |
| 148 | Triisobutylamine | 52.30 | 52.10 | 52.40 | -0.10 |
| 149 | 1-Bromo-2-(2-methoxyethoxy)benzene | 64.50 | 64.80 | 64.60 | -0.10 |
| 150 | 2-Methylanthracene | 84.50 | 84.40 | 84.60 | -0.10 |
| 151 | Ethyl phenylacetate | 64.50 | 64.50 | 64.60 | -0.10 |
| 152 | 1,2,3,7,8,9-Hexachlorodibenzo-p-dioxin | 91.70 | 92.30 | 91.80 | -0.10 |
| 153 | (E) 12-Hexadecenal | 89.30 | 89.20 | 89.10 | 0.20 |
| 154 | (Z) 6-Decen-1-ol | 80.30 | 80.10 | 80.10 | 0.20 |
| 155 | 1-Phenyl-1H-imidazole | 74.30 | 73.20 | 74.10 | 0.20 |
| 156 | Benzylsalicylate | 96.80 | 97.20 | 96.60 | 0.20 |
| 157 | Ethyl stearate | 109.30 | 109.20 | 109.10 | 0.20 |
| 158 | Nonadecane | 95.80 | 95.80 | 95.60 | 0.20 |
| 159 | 1,2-Diiodobenzene | 65.60 | 66.10 | 65.40 | 0.20 |
| 160 | 2',4'-Dichloroacetophenone | 63.60 | 63.30 | 63.40 | 0.20 |
| 161 | 2,2-diphenyl-1,3-dioxolane | 84.60 | 84.60 | 84.40 | 0.20 |
| 162 | 2,5-Dimethylbenzoxazole | 60.10 | 61.60 | 59.90 | 0.20 |
| 163 | 2-(Diethylamino)-1-phenylethanone | 71.60 | 71.30 | 71.40 | 0.20 |
| 164 | N-(2-Aminoethyl)ethanolamine | 75.10 | 74.90 | 74.90 | 0.20 |
| 165 | Dihydropinene | 46.10 | 45.30 | 45.90 | 0.20 |
| 166 | Tetradecylbenzene | 99.60 | 99.50 | 99.40 | 0.20 |
| 167 | 8,8-Dipentylheptadecane | 128.10 | 128.00 | 127.90 | 0.20 |
| 168 | 3-Isopropyltoluene | 50.00 | 49.80 | 49.80 | 0.20 |
| 169 | 3,3-Dimethylpentane | 33.00 | 32.80 | 32.80 | 0.20 |
| 170 | Benzaldehyde | 49.00 | 48.50 | 48.80 | 0.20 |
| 171 | 2,6-Dimethylheptan-4-one | 51.00 | 50.90 | 50.80 | 0.20 |
| 172 | Perfluorotoluene | 40.50 | 39.10 | 40.30 | 0.20 |
| 173 | (E) 13-Pentadecenyl acetate | 95.90 | 95.90 | 95.70 | 0.20 |
| 174 | 1,2,3,4-Tetrachlorodibenzofuran | 84.40 | 84.40 | 84.20 | 0.20 |
| 175 | 2,4-Dimethylheptane | 42.90 | 42.80 | 42.70 | 0.20 |
| 176 | 2,5-Dimethyl-2-hexene | 38.90 | 38.80 | 38.70 | 0.20 |
| 177 | 4-Ethyl-2-methylhexane | 42.90 | 42.80 | 42.70 | 0.20 |
| 178 | 5,5-Bis(3,3'-dimethylbutyl)-2,2,8,8-tetramethylnonane | 91.90 | 91.60 | 91.70 | 0.20 |
| 179 | Biphenylene | 66.40 | 66.00 | 66.20 | 0.20 |
| 180 | cis-3,5-Dimethyl-2-hexene | 38.90 | 38.80 | 38.70 | 0.20 |
| 181 | Diethyleneglycol diethyl ether | 56.40 | 55.90 | 56.20 | 0.20 |
| 182 | Heptadecanonitrile | 98.90 | 99.10 | 98.70 | 0.20 |
| 183 | Butanedioic acid | 94.40 | 94.30 | 94.20 | 0.20 |
| 184 | Tetradecane | 71.60 | 72.00 | 71.80 | -0.20 |
| 185 | trans-1,2-Divinylcyclobutane | 38.90 | 38.90 | 38.70 | 0.20 |
| 186 | trans-4-Methyl-3-ethyl-2-pentene | 38.90 | 38.80 | 38.70 | 0.20 |
| 187 | 1,1,1,2,4,4-Hexafluoro-2-(trifluoromethoxy)butane | 33.80 | 33.60 |  | 0.20 |
| 188 | 1,1-Difluoro-1,2,2-trichloroethane | 32.80 | 32.20 | 32.60 | 0.20 |
| 189 | alpha-Methyl benzylamine | 55.30 | 55.20 | 55.10 | 0.20 |
| 190 | 1-Bromo-3-fluorobenzene | 44.30 | 44.10 | 44.10 | 0.20 |
| 191 | 2,4,6-Trimethyl-3,5-dioxaheptane | 43.30 | 43.60 | 43.10 | 0.20 |
| 192 | p-Toluidine | 57.80 | 58.00 | 57.60 | 0.20 |
| 193 | cis-2,5-Dimethyl-3-hexene | 37.20 | 37.50 | 37.40 | -0.20 |
| 194 | cis-Bicyclo[6.1.0]nonane | 49.30 | 49.20 | 49.10 | 0.20 |
| 195 | Thioanisole | 54.30 | 53.00 | 54.10 | 0.20 |
| 196 | (E) 13-Hexadecenyl acetate | 100.30 | 100.50 | 100.50 | -0.20 |
| 197 | 1-Decylazide | 67.80 | 67.20 | 68.00 | -0.20 |
| 198 | 1-Iododecane | 69.80 | 69.90 | 70.00 | -0.20 |
| 199 | Dimethyl azelate | 82.30 | 82.50 | 82.50 | -0.20 |
| 200 | n-Dodecylamine | 75.30 | 75.70 | 75.50 | -0.20 |
| 201 | Tri-n-butyl phosphate | 81.30 | 81.20 | 81.50 | -0.20 |
| 202 | 1,4-Dichlorobutane | 46.70 | 46.20 | 46.50 | 0.20 |
| 203 | Isobutyl iodide | 38.80 | 39.20 | 39.00 | -0.20 |
| 204 | 1-Propoxy-2-ethoxyethane | 46.80 | 46.90 | 47.00 | -0.20 |
| 205 | 2,2,3,3-Tetrafluoropropyl-1',1',2',3',3'-hexafluoropropyl ether | 39.70 | 39.30 | 39.50 | 0.20 |
| 206 | 2,2-Dimethylhexane | 37.30 | 37.50 | 37.50 | -0.20 |
| 207 | 2,7-Dimethyloctane | 47.70 | 47.50 | 47.50 | 0.20 |
| 208 | 3,4-Diethylhexane | 47.70 | 47.50 | 47.50 | 0.20 |
| 209 | 3,6-Dimethyloctane | 47.30 | 47.50 | 47.50 | -0.20 |
| 210 | 3-Ethyl-1-pentene | 34.30 | 34.30 | 34.50 | -0.20 |
| 211 | 3-Ethyl-2-methyl-1-pentene | 37.70 | 37.60 | 37.50 | 0.20 |
| 212 | 4,4-Dimethylheptane | 42.20 | 42.30 | 42.00 | 0.20 |
| 213 | 4-Ethyl-2-methylheptane | 47.30 | 47.50 | 47.50 | -0.20 |
| 214 | 4-Isopropylheptane | 47.30 | 47.50 | 47.50 | -0.20 |
| 215 | 4-t-Butylcyclohexyl acetate | 63.80 | 63.90 | 64.00 | -0.20 |
| 216 | 5-Ethyl-3-methylheptane | 47.70 | 47.50 | 47.50 | 0.20 |
| 217 | Allyl ethyl sulfide | 39.30 | 39.40 | 39.50 | -0.20 |
| 218 | Allyl t-butyl sulfide | 44.80 | 45.20 | 45.00 | -0.20 |
| 219 | Di-t-butylsulfide | 43.80 | 43.90 | 44.00 | -0.20 |
| 220 | Dipropylnitramine | 57.30 | 57.70 | 57.50 | -0.20 |
| 221 | 3-Methylbutanoic acid | 61.20 | 61.10 | 61.00 | 0.20 |
| 222 | trans-2,2-Dimethyl-3-hexene | 37.20 | 37.00 | 37.00 | 0.20 |
| 223 | trans-2-Phenylcyclopropylamine | 60.30 | 60.30 | 60.50 | -0.20 |
| 224 | 2,4-Dimethyl-1-pentene | 33.10 | 33.00 | 32.90 | 0.20 |
| 225 | 2-Methyl-2-heptanol | 62.90 | 63.30 | 63.10 | -0.20 |
| 226 | 4,6-Dimethylindane | 57.90 | 58.40 | 58.10 | -0.20 |
| 227 | 6-Methyl-1-heptene | 38.90 | 38.90 | 39.10 | -0.20 |
| 228 | 2-Phenylpropionitrile | 60.90 | 61.10 | 61.10 | -0.20 |
| 229 | Benzyl methyl ether | 51.40 | 51.70 | 51.60 | -0.20 |
| 230 | cis-4,4-Dimethyl-2-pentene | 32.60 | 32.40 | 32.40 | 0.20 |
| 231 | cis-Crotononitrile | 38.90 | 39.10 | 39.10 | -0.20 |
| 232 | trans-4-Octene | 42.90 | 42.90 | 43.10 | -0.20 |
| 233 | 1,2-Dichlorohexafluoropropane | 26.90 | 26.90 | 27.10 | -0.20 |
| 234 | 1,2-Butadiene | 23.90 | 24.10 | 24.10 | -0.20 |
| 235 | 3-(Difluoromethoxy)-1,1,1,2,2-pentafluoropropane | 31.20 | 31.30 | 31.40 | -0.20 |
| 236 | 4,4-Dimethyl-1-pentene | 31.20 | 31.10 | 31.40 | -0.20 |
| 237 | (E) 9-Tetradecenal | 79.50 | 79.90 | 79.70 | -0.20 |
| 238 | 1,3-Diethylbenzene | 52.50 | 52.50 | 52.70 | -0.20 |
| 239 | 1-Bromo-2-chloro-1,1,2-trifluoroethane | 30.00 | 30.20 |  | -0.20 |
| 240 | 1-Bromo-4-fluorobenzene | 44.00 | 44.10 | 44.20 | -0.20 |
| 241 | 1-Formylnaphthalene | 71.00 | 71.20 | 71.20 | -0.20 |
| 242 | 2-Butyn-1,4-diol | 81.50 | 81.40 | 81.70 | -0.20 |
| 243 | Isopropenylacetylene | 27.00 | 27.20 | 27.20 | -0.20 |
| 244 | Di-n-pentyl adipate | 100.50 | 100.80 | 100.70 | -0.20 |
| 245 | Heptadecane | 86.00 | 86.30 | 86.20 | -0.20 |
| 246 | 2-Propanol | 44.00 | 44.30 | 44.20 | -0.20 |
| 247 | Isopropyl 3-methyl-2-butenoate | 50.00 | 50.10 | 50.20 | -0.20 |
| 248 | 2-Propanethiol | 29.50 | 29.80 | 29.70 | -0.20 |
| 249 | Methyl 2-phenylpropionate | 64.00 | 64.30 | 64.20 | -0.20 |
| 250 | O,O-Diethyl O-2-dimethylaminopropyl thiophosphate | 71.50 | 71.70 | 71.70 | -0.20 |
| 251 | 3-t-Butyltoluene | 51.10 | 51.20 | 51.30 | -0.20 |
| 252 | 1a,2a,4b-1,2,4-Trimethylcyclopentane | 38.10 | 38.50 | 38.30 | -0.20 |
| 253 | 2-Dimethylaminoethanethiol | 41.10 | 41.40 | 41.30 | -0.20 |
| 254 | Methyltripropylsilane | 42.60 | 43.20 | 42.80 | -0.20 |
| 255 | 1-Propyl-2-methylimidazole | 63.70 | 64.20 | 63.90 | -0.20 |
| 256 | 3-Pentanol | 53.20 | 53.60 | 53.40 | -0.20 |
| 257 | 5-Hexen-1-ol | 60.20 | 60.20 | 60.40 | -0.20 |
| 258 | Isobutyl 3-methylbut-2-enoate | 54.70 | 54.80 | 54.90 | -0.20 |
| 259 | 3-Methyl-1-butanethiol | 39.70 | 40.00 | 39.90 | -0.20 |
| 260 | Methyl pentyl sulfide | 45.20 | 45.10 | 45.40 | -0.20 |
| 261 | (E) 3-Dodecen-1-ol | 89.20 | 89.40 | 89.40 | -0.20 |
| 262 | (E) 3-Dodecenal | 70.20 | 70.50 | 70.40 | -0.20 |
| 263 | Cetane | 81.20 | 81.60 | 81.40 | -0.20 |
| 264 | (E) 4-Hexadecenal | 88.90 | 89.20 | 89.10 | -0.20 |
| 265 | (Z) 12-Tetradecenyl acetate | 90.90 | 91.20 | 91.10 | -0.20 |
| 266 | N,N-Bis(2-hydroxyethyl)ethylenediamine | 106.40 | 106.60 | 106.60 | -0.20 |
| 267 | (Z) 10-Tetradecenal | 79.60 | 79.90 | 79.80 | -0.20 |
| 268 | s-Butyl isopropyl ether | 37.58 | 37.70 | 37.30 | 0.28 |
| 269 | (E) 3-Tetradecenal | 80.10 | 79.90 | 79.80 | 0.30 |
| 270 | Dimethyl suberate | 78.10 | 77.90 | 77.80 | 0.30 |
| 271 | (E) 10-Dodecenal | 70.90 | 70.50 | 70.60 | 0.30 |
| 272 | 2-Fluorofluorene | 71.90 | 71.40 | 71.60 | 0.30 |
| 273 | 3-Ethyl-2-methylhexane | 43.20 | 42.80 | 42.90 | 0.30 |
| 274 | Adamantane | 51.70 | 51.70 | 51.40 | 0.30 |
| 275 | Camphene | 44.70 | 44.50 | 44.40 | 0.30 |
| 276 | 1,1,2-Trichloroethane | 40.10 | 39.70 | 39.80 | 0.30 |
| 277 | Chlorocyclohexane | 42.10 | 41.60 | 41.80 | 0.30 |
| 278 | 1,4-Diisopropylbenzene | 56.50 | 56.30 | 56.20 | 0.30 |
| 279 | 1-Heptadecanol | 112.50 | 112.10 | 112.20 | 0.30 |
| 280 | 2-(Ethylamino)ethanol | 61.00 | 60.70 | 60.70 | 0.30 |
| 281 | 2-Methyl-3-pentanol | 56.00 | 55.60 | 55.70 | 0.30 |
| 282 | 3,3-Dimethyl-1-hexene | 36.00 | 35.70 | 35.70 | 0.30 |
| 283 | 3-Phenyltoluene | 68.50 | 68.40 | 68.20 | 0.30 |
| 284 | Dimethyl pimelate | 73.50 | 73.20 | 73.20 | 0.30 |
| 285 | n-Butylmethylsulfide | 40.50 | 40.50 | 40.20 | 0.30 |
| 286 | 1,9-Nonanediamine | 75.50 | 75.90 | 75.80 | -0.30 |
| 287 | Octylcyanide | 62.00 | 61.70 | 61.70 | 0.30 |
| 288 | Nonyl acetate | 67.00 | 67.10 | 67.30 | -0.30 |
| 289 | Isoprene | 26.40 | 26.00 | 26.10 | 0.30 |
| 290 | 1,2,3-Trimethylcyclopentane | 38.90 | 38.50 | 38.60 | 0.30 |
| 291 | 2-Hexyne | 35.90 | 35.80 | 35.60 | 0.30 |
| 292 | N-Isopropylimidazole | 58.40 | 58.50 | 58.10 | 0.30 |
| 293 | N-Propylpiperidine | 44.90 | 44.90 | 44.60 | 0.30 |
| 294 | trans-(+)-Bicyclo-[6.1.0]nonane | 49.40 | 49.20 | 49.10 | 0.30 |
| 295 | 1,2-Diethylbenzene | 52.80 | 52.50 | 52.50 | 0.30 |
| 296 | 1-Fluorononane | 50.80 | 50.60 | 50.50 | 0.30 |
| 297 | 2,3-Dimethyl-2-hexene | 39.70 | 40.20 | 40.00 | -0.30 |
| 298 | 2-Bromotoluene | 47.70 | 48.00 | 48.00 | -0.30 |
| 299 | 2-Ethoxy-4,4,5,5-tetramethyl-1,3-dioxolane | 47.70 | 47.90 | 48.00 | -0.30 |
| 300 | 3-Chloro-2-methylpropene | 33.30 | 33.20 | 33.00 | 0.30 |
| 301 | Cyclopentanol | 56.70 | 56.80 | 57.00 | -0.30 |
| 302 | Ethyl 3-thiopheneacetate | 63.20 | 63.30 | 63.50 | -0.30 |
| 303 | Ethyl valerate | 48.20 | 48.50 | 48.50 | -0.30 |
| 304 | Thiolane | 38.80 | 38.40 | 38.50 | 0.30 |
| 305 | trans-3,4-Dimethyl-3-hexene | 39.70 | 40.20 | 40.00 | -0.30 |
| 306 | trans-4,4-Dimethyl-2-pentene | 32.80 | 32.40 | 32.50 | 0.30 |
| 307 | 1-Iodoundecane | 74.80 | 74.60 | 74.50 | 0.30 |
| 308 | 1,10-Decanediamine | 80.20 | 80.60 | 80.50 | -0.30 |
| 309 | Tri(butylthio)borane | 95.80 | 95.50 |  | 0.30 |
| 310 | 2-Ethyl-1-butene | 31.00 | 31.10 | 31.30 | -0.30 |
| 311 | 1,2-Diiodoethane | 49.80 | 50.40 | 50.10 | -0.30 |
| 312 | Neopentyl t-butyl ketone | 48.80 | 49.10 | 49.10 | -0.30 |
| 313 | 2,4-Dimethylhexane | 37.80 | 38.00 | 38.10 | -0.30 |
| 314 | o-Toluidine | 57.30 | 58.00 | 57.60 | -0.30 |
| 315 | Allyltrichloroacetate | 52.30 | 52.90 | 52.60 | -0.30 |
| 316 | anti-Tricyclo[4.2.0.02.5]octane | 41.80 | 42.20 | 42.10 | -0.30 |
| 317 | Pyridine | 40.30 | 40.90 | 40.60 | -0.30 |
| 318 | 1-Butanol | 50.90 | 51.40 | 51.20 | -0.30 |
| 319 | 2,2-Dimethylpentane | 32.40 | 32.80 | 32.70 | -0.30 |
| 320 | Divinyl sulfone | 56.40 | 57.10 | 56.70 | -0.30 |
| 321 | Capraldehyde | 60.40 | 60.50 | 60.70 | -0.30 |
| 322 | Isopentene | 25.90 | 26.50 | 26.20 | -0.30 |
| 323 | Benzyl ethyl ether | 53.50 | 53.90 | 53.80 | -0.30 |
| 324 | Butyl ethyl sulfide | 44.50 | 44.60 | 44.80 | -0.30 |
| 325 | Ethyl crotonate | 44.00 | 44.50 | 44.30 | -0.30 |
| 326 | Ethyltripropylsilane | 41.00 | 42.40 | 41.30 | -0.30 |
| 327 | n-Butylnitrite | 37.00 | 37.40 | 37.30 | -0.30 |
| 328 | t-Butylbenzene | 47.50 | 47.50 | 47.80 | -0.30 |
| 329 | trans-2,4-Dimethyl-3-hexene | 38.50 | 38.80 | 38.80 | -0.30 |
| 330 | Dimethyl phthalate | 78.60 | 79.00 | 78.90 | -0.30 |
| 331 | 1a,2b,4a-1,2,4-Trimethylcyclopentane | 38.10 | 38.50 | 38.40 | -0.30 |
| 332 | 2,2',3,6,6'-Pentachlorobiphenyl | 89.60 | 89.50 | 89.90 | -0.30 |
| 333 | Carvone | 58.60 | 59.00 | 58.90 | -0.30 |
| 334 | Dimethylterephthalate | 78.60 | 79.00 | 78.90 | -0.30 |
| 335 | Dodecane | 62.10 | 62.50 | 62.40 | -0.30 |
| 336 | Octadecane | 90.60 | 91.10 | 90.90 | -0.30 |
| 337 | (E) 10-Hexadecenal | 88.80 | 89.20 | 89.10 | -0.30 |
| 338 | Tridecanonitrile | 80.30 | 80.40 | 80.60 | -0.30 |
| 339 | (Z) 13-Hexadecenyl acetate | 100.00 | 100.50 | 100.30 | -0.30 |
| 340 | 1-Pentadecanol | 102.50 | 102.70 | 102.80 | -0.30 |
| 341 | Methyl arachidate | 116.00 | 116.40 | 116.30 | -0.30 |
| 342 | (Z) 15-Octadecenyl acetate | 110.20 | 109.90 | 109.80 | 0.40 |
| 343 | 1-Hexadecanol | 107.70 | 107.40 | 107.30 | 0.40 |
| 344 | 1-Fluorotetradecane | 73.50 | 73.90 | 73.10 | 0.40 |
| 345 | 1-Methylphenanthrene | 84.50 | 84.40 | 84.10 | 0.40 |
| 346 | 2,4-Diisopropylphenol | 82.00 | 81.50 | 81.60 | 0.40 |
| 347 | Methyl Z,Z,Z,Z,Z 5,8,11,14,17-eicosapentaeneoate | 121.00 | 120.00 | 120.60 | 0.40 |
| 348 | 1,1,2-Trimethylpropyl 2,2-dimethylpropanoate | 52.80 | 51.70 | 52.40 | 0.40 |
| 349 | Ethylisopropylketone | 39.80 | 39.60 | 39.40 | 0.40 |
| 350 | Eicosane | 100.80 | 100.60 | 100.40 | 0.40 |
| 351 | 1,4-Dicyclopropylbuta-1,3-diyne | 58.20 | 57.90 | 57.80 | 0.40 |
| 352 | 2-Propyltoluene | 52.70 | 52.50 | 52.30 | 0.40 |
| 353 | 2,2-Dimethyl-4-ethylhexane | 45.20 | 44.30 | 44.80 | 0.40 |
| 354 | 4-Ethyl-3-methylheptane | 47.70 | 47.50 | 47.30 | 0.40 |
| 355 | 4-Methyl-1-hexene | 34.70 | 34.30 | 34.30 | 0.40 |
| 356 | (E) 3-Hexadecenal | 89.60 | 89.20 | 89.20 | 0.40 |
| 357 | 2-Acetyl-3-methylthiophene | 57.10 | 56.80 | 56.70 | 0.40 |
| 358 | 2-Nonanone | 56.60 | 56.40 | 56.20 | 0.40 |
| 359 | Tetraethylmethane | 42.60 | 42.30 | 42.20 | 0.40 |
| 360 | 1,1-Diethoxyethane | 39.60 | 39.10 | 39.20 | 0.40 |
| 361 | Dibutyldisulfide | 64.10 | 63.70 | 63.70 | 0.40 |
| 362 | Ethyldiisopentylsilane | 42.60 | 42.20 | 42.20 | 0.40 |
| 363 | t-Butyl-1,1,3,3-tetra(methylbutyl)diazene | 53.60 | 53.20 | 53.20 | 0.40 |
| 364 | 2,4-Dimethyl-2-pentene | 34.50 | 34.20 | 34.10 | 0.40 |
| 365 | 2-Fluoroiodobenzene | 51.50 | 51.10 | 51.10 | 0.40 |
| 366 | 3-Hexyne | 36.00 | 35.80 | 35.60 | 0.40 |
| 367 | Cyclohexanol | 62.00 | 61.50 | 61.60 | 0.40 |
| 368 | Ethyl propanoate | 39.50 | 39.10 | 39.10 | 0.40 |
| 369 | 2-Methyl-1-propanethiol | 35.50 | 35.30 | 35.10 | 0.40 |
| 370 | Isobutylnitrite | 35.00 | 34.70 | 34.60 | 0.40 |
| 371 | Nortricyclene | 38.00 | 37.40 | 37.60 | 0.40 |
| 372 | Propyl 3-methyl-2-butenoate | 53.00 | 52.50 | 52.60 | 0.40 |
| 373 | S-Ethyl thioacetate | 40.00 | 39.90 | 39.60 | 0.40 |
| 374 | 1,1,1-Trichlorotrifluoroethane | 29.20 | 29.00 | 28.80 | 0.40 |
| 375 | Perfluoromethyldiethylamine | 29.40 | 29.00 |  | 0.40 |
| 376 | (E) 9-Hexadecenal | 88.60 | 89.20 | 89.00 | -0.40 |
| 377 | (Z) 4-Dodecen-1-ol | 89.90 | 89.40 | 89.50 | 0.40 |
| 378 | 1,2,4,6,7,9-Hexachlorodibenzo-p-dioxin | 92.10 | 92.30 | 92.50 | -0.40 |
| 379 | 1,3-Diiodobenzene | 66.10 | 66.10 | 66.50 | -0.40 |
| 380 | 3-Propyltoluene | 52.10 | 52.50 | 52.50 | -0.40 |
| 381 | 1-Methylnaphthalene | 62.40 | 62.00 | 62.00 | 0.40 |
| 382 | 2-Chloro-1,1,2-trifluoroethyl methyl ether | 34.40 | 33.70 | 34.00 | 0.40 |
| 383 | 2-Butanethiol | 34.10 | 34.50 | 34.50 | -0.40 |
| 384 | 2-Octanol | 67.90 | 67.60 | 67.50 | 0.40 |
| 385 | 3-Bromotoluene | 48.40 | 48.00 | 48.00 | 0.40 |
| 386 | 3-Fluorophenol | 60.10 | 60.60 | 60.50 | -0.40 |
| 387 | 3-Methyl-trans-2-pentene | 32.10 | 32.40 | 32.50 | -0.40 |
| 388 | Biphenyl | 64.90 | 64.70 | 64.50 | 0.40 |
| 389 | Decahydroazulene | 53.60 | 53.90 | 54.00 | -0.40 |
| 390 | Propyl trichloroacetate | 53.10 | 53.50 | 53.50 | -0.40 |
| 391 | Allyl chloride | 29.90 | 29.90 | 30.30 | -0.40 |
| 392 | 1,2-Dibromoethane | 41.70 | 42.00 | 42.10 | -0.40 |
| 393 | Diisobutyl ether | 41.20 | 41.70 | 41.60 | -0.40 |
| 394 | Ethanol | 41.70 | 42.00 | 42.10 | -0.40 |
| 395 | Methylisothiocyanate | 37.30 | 36.90 |  | 0.40 |
| 396 | 1,2,3,4-Tetrahydronaphthalene | 55.20 | 55.70 | 55.60 | -0.40 |
| 397 | (E) 12-Tetradecenyl acetate | 90.80 | 91.20 | 91.20 | -0.40 |
| 398 | (E) 6-Tetradecenal | 79.30 | 79.90 | 79.70 | -0.40 |
| 399 | Ethylene glycol dibutyrate | 73.20 | 72.80 | 72.80 | 0.40 |
| 400 | Hexadecylcyclopentane | 105.30 | 105.80 | 105.70 | -0.40 |
| 401 | 2,3-Dimethyl-2-butanethiol | 39.30 | 39.60 | 39.70 | -0.40 |
| 402 | 2-Pentyne | 30.80 | 31.20 | 31.20 | -0.40 |
| 403 | Ethyl 2-diethylaminobutanoate | 57.30 | 57.40 | 57.70 | -0.40 |
| 404 | Ethyl-t-butylsulfide | 39.30 | 39.60 | 39.70 | -0.40 |
| 405 | Perfluoro(dibutylmethylamine) | 48.80 | 49.20 |  | -0.40 |
| 406 | t-Amyl isobutyrate | 47.80 | 48.30 | 48.20 | -0.40 |
| 407 | Thiane | 42.80 | 43.10 | 43.20 | -0.40 |
| 408 | trans-8-Methyl-2-hydrindanone | 58.30 | 58.70 | 58.70 | -0.40 |
| 409 | 1-Decene | 50.40 | 50.90 | 50.80 | -0.40 |
| 410 | 2-Iodobutane | 37.90 | 38.20 | 38.30 | -0.40 |
| 411 | (Iodomethyl)benzene | 57.40 | 58.00 | 57.80 | -0.40 |
| 412 | Cyclooctanone | 54.40 | 54.90 | 54.80 | -0.40 |
| 413 | Heptanonitrile | 51.90 | 52.40 | 52.30 | -0.40 |
| 414 | (E) 10-Dodecenyl acetate | 81.50 | 81.90 | 81.90 | -0.40 |
| 415 | (Z) 8-Dodecenal | 70.00 | 70.50 | 70.40 | -0.40 |
| 416 | 1-Fluorododecane | 64.00 | 64.60 | 64.40 | -0.40 |
| 417 | 3-Ethyl-1-hexene | 38.50 | 38.90 | 38.90 | -0.40 |
| 418 | 4-Fluorotoluene | 39.50 | 40.00 | 39.90 | -0.40 |
| 419 | delta-Decanolactone | 63.00 | 63.30 | 63.40 | -0.40 |
| 420 | Ethyl 4-hydroxybenzoate | 84.00 | 84.20 | 84.40 | -0.40 |
| 421 | Methyl Z,Z 11,14-eicosadieneoate | 117.50 | 117.80 | 117.90 | -0.40 |
| 422 | N-Butylcarbazole | 92.50 | 89.60 | 92.90 | -0.40 |
| 423 | (Z) 4-Hexadecenal | 88.70 | 89.20 | 89.10 | -0.40 |
| 424 | Tridecane | 66.70 | 67.30 | 67.10 | -0.40 |
| 425 | (Z) 10-Dodecenyl acetate | 81.40 | 81.90 | 81.80 | -0.40 |
| 426 | (E) 4-Decen-1-ol | 80.30 | 80.10 | 79.80 | 0.50 |
| 427 | (Z) 11-Tetradecenal | 80.30 | 79.90 | 79.80 | 0.50 |
| 428 | (Z) 5-Decen-1-ol | 80.30 | 80.10 | 79.80 | 0.50 |
| 429 | Methyl Z,Z,Z,Z 5,8,11,14-eicosatetraeneoate | 118.30 | 119.20 | 117.80 | 0.50 |
| 430 | N-Ethyltetrahydrocarbazole | 82.80 | 81.30 | 82.30 | 0.50 |
| 431 | (E) 5-Decen-1-ol | 80.60 | 80.10 | 80.10 | 0.50 |
| 432 | Methyl 4-bromobenzoate | 66.10 | 65.60 | 65.60 | 0.50 |
| 433 | 2-Nonanol | 72.90 | 72.30 | 72.40 | 0.50 |
| 434 | 3,3,3-Trimethoxypropionitrile | 55.90 | 55.70 | 55.40 | 0.50 |
| 435 | Diisoamyl ether | 51.40 | 51.00 | 50.90 | 0.50 |
| 436 | Dimethyl isophthalate | 79.40 | 79.00 | 78.90 | 0.50 |
| 437 | Methyl heneicosanoate | 120.90 | 121.00 | 120.40 | 0.50 |
| 438 | 2,6-Dimethylheptane | 43.30 | 42.80 | 42.80 | 0.50 |
| 439 | 3-Acetyl-2,5-dimethylthiophene | 61.30 | 61.10 | 60.80 | 0.50 |
| 440 | Acetic acid | 50.30 | 49.90 | 49.80 | 0.50 |
| 441 | cis-4-Methyl-3-ethyl-2-pentene | 39.30 | 38.80 | 38.80 | 0.50 |
| 442 | Diethyl sulphide | 35.80 | 35.20 | 35.30 | 0.50 |
| 443 | 2,2,4-Trimethyl-3-pentanone | 43.30 | 43.00 | 42.80 | 0.50 |
| 444 | Citronellyl acetate | 67.80 | 68.40 | 68.30 | -0.50 |
| 445 | 1-Bromododecane | 74.80 | 75.10 | 75.30 | -0.50 |
| 446 | 1,1,5-trichloro-1-(2,2-Dichloro-1,1,2-trifluoroethoxy)-2,2,3,3,4,4,5,5-octafluoropentane | 59.20 | 59.30 | 58.70 | 0.50 |
| 447 | Methyl 2-dimethylamino-2-methylpropanoate | 49.20 | 48.80 | 48.70 | 0.50 |
| 448 | Methyl propyl sulfide | 36.20 | 35.80 | 35.70 | 0.50 |
| 449 | t-Butylacetylene | 28.20 | 27.80 | 27.70 | 0.50 |
| 450 | Vinylcyclopropane | 28.90 | 28.30 | 28.40 | 0.50 |
| 451 | Dibutyl acetal | 58.60 | 57.70 | 58.10 | 0.50 |
| 452 | Neopentyl mercaptan | 36.40 | 36.70 | 36.90 | -0.50 |
| 453 | 4-Methyl-2-thiapentane | 36.90 | 37.70 | 37.40 | -0.50 |
| 454 | cis-2-Heptene | 38.60 | 38.30 | 38.10 | 0.50 |
| 455 | cis-3-Methyl-3-hexene | 36.40 | 37.00 | 36.90 | -0.50 |
| 456 | Suberone | 50.60 | 50.20 | 50.10 | 0.50 |
| 457 | 2,3-Pentadiene | 29.50 | 30.00 |  | -0.50 |
| 458 | 2,4-Dimethyl-2-hexene | 38.50 | 38.80 | 39.00 | -0.50 |
| 459 | 2-Chloroethyl vinyl ether | 38.00 | 37.70 | 37.50 | 0.50 |
| 460 | 2-Iodopropane | 34.00 | 33.50 |  | 0.50 |
| 461 | 2-Octyne | 44.50 | 45.00 | 45.00 | -0.50 |
| 462 | 3-Ethyl-2-methylpentane | 38.50 | 38.00 | 38.00 | 0.50 |
| 463 | 3-Ethyl-3-methylpentane | 38.00 | 37.50 | 37.50 | 0.50 |
| 464 | 3-Methyl-1-heptene | 38.50 | 38.90 | 39.00 | -0.50 |
| 465 | 5,5-Dimethyl-1,3-dioxane | 41.00 | 40.00 | 40.50 | 0.50 |
| 466 | 3-Methylpyridine | 44.50 | 44.60 | 45.00 | -0.50 |
| 467 | Bis(1,1,3,3-tetramethylbutyl)diazene | 66.50 | 65.90 | 66.00 | 0.50 |
| 468 | cis-2,4-Dimethyl-3-hexene | 38.50 | 38.80 | 39.00 | -0.50 |
| 469 | Diethyl sulfite | 48.50 | 47.40 | 48.00 | 0.50 |
| 470 | Dimethyl adipate | 69.00 | 68.50 | 68.50 | 0.50 |
| 471 | Isopropylacetate | 37.00 | 36.70 | 36.50 | 0.50 |
| 472 | 3-(Dimethylamino)propylamine | 46.00 | 46.40 | 46.50 | -0.50 |
| 473 | 1,1,2-Trifluoro-2-chloroethyl-2,2,3,3,4,4,5,5-octafluoropentyl ether | 54.10 | 54.10 | 54.60 | -0.50 |
| 474 | 2-Ethyl-1-pentene | 35.10 | 35.70 | 35.60 | -0.50 |
| 475 | Bromopentafluorobenzene | 43.10 | 43.30 | 43.60 | -0.50 |
| 476 | Perfluorotripropylamine | 46.60 | 47.00 | 47.10 | -0.50 |
| 477 | p-Cymene | 49.20 | 49.80 | 49.70 | -0.50 |
| 478 | 2,4-Dimethylpentane | 32.70 | 33.30 | 33.20 | -0.50 |
| 479 | 3-Methyleneheptane | 39.70 | 40.30 | 40.20 | -0.50 |
| 480 | 3-Butyn-1-ol | 51.70 | 52.10 | 52.20 | -0.50 |
| 481 | Isopentyliodide | 43.20 | 43.90 | 43.70 | -0.50 |
| 482 | Tri-(2-methylbutyl) phosphate | 86.70 | 87.00 | 87.20 | -0.50 |
| 483 | 1,2-Dichlorohexane | 48.80 | 49.50 | 49.30 | -0.50 |
| 484 | 1-Bromooctane | 55.80 | 56.40 | 56.30 | -0.50 |
| 485 | 2,5-Dioxaheptane | 39.80 | 40.00 | 40.30 | -0.50 |
| 486 | 1-Methyl-2-pyrrolecarboxaldehyde | 55.80 | 55.30 | 56.30 | -0.50 |
| 487 | s-Butylamine | 33.80 | 34.40 | 34.30 | -0.50 |
| 488 | 2-Methyl-5-hexen-3-yn-2-ol | 60.80 | 60.80 | 61.30 | -0.50 |
| 489 | 3,5-Dithiaheptane | 50.80 | 51.30 |  | -0.50 |
| 490 | 4,7-Dimethylindane | 58.30 | 58.40 | 58.80 | -0.50 |
| 491 | 5-Methyl-3-hexanol | 59.80 | 60.20 | 60.30 | -0.50 |
| 492 | 5-Methylnonane | 49.80 | 50.30 | 50.30 | -0.50 |
| 493 | n-Butyl acrylate | 47.30 | 47.90 | 47.80 | -0.50 |
| 494 | (E) 4-Dodecenal | 69.90 | 70.50 | 70.40 | -0.50 |
| 495 | (Z) 3-Tetradecenal | 79.40 | 79.90 | 79.90 | -0.50 |
| 496 | 1-Butoxy-2-ethoxyethane | 50.90 | 51.50 | 51.40 | -0.50 |
| 497 | 2-Methyloctane | 44.90 | 45.50 | 45.40 | -0.50 |
| 498 | 3-Methyloctane | 44.90 | 45.50 | 45.40 | -0.50 |
| 499 | Methyl 2-thiopheneacetate | 61.90 | 62.10 | 62.40 | -0.50 |
| 500 | Tetradecyl acetate | 89.90 | 90.50 | 90.40 | -0.50 |
| 501 | Amphepramone | 71.60 | 72.10 | 72.10 | -0.50 |
| 502 | N,2-Dimethylpropanamide | 67.10 | 67.40 | 67.60 | -0.50 |
| 503 | n-Undecanal | 64.60 | 65.20 | 65.10 | -0.50 |
| 504 | (E) 8-Tetradecenal | 79.30 | 79.90 | 79.80 | -0.50 |
| 505 | 1,3,6,8-Tetrachlorodibenzofuran | 83.80 | 84.40 | 84.30 | -0.50 |
| 506 | Anthracene | 79.80 | 80.70 | 80.30 | -0.50 |
| 507 | Pentadecylcyclopentane | 100.30 | 101.10 | 100.80 | -0.50 |
| 508 | 2-Methyltetrahydrofuran | 33.63 | 33.90 | 34.20 | -0.57 |
| 509 | Pyrrolidine | 37.38 | 36.60 | 36.80 | 0.58 |
| 510 | Behenyl alcohol | 135.90 | 135.40 | 135.30 | 0.60 |
| 511 | (Z) 13-Hexadecenal | 89.70 | 89.20 | 89.10 | 0.60 |
| 512 | 1,8-Octanediamine | 71.70 | 71.20 | 71.10 | 0.60 |
| 513 | 2,5-Dimethyl-2-hydroperoxide-5-tert-butylperoxyhexane | 74.20 | 73.50 | 73.60 | 0.60 |
| 514 | 4-Bromophenol | 68.70 | 68.70 | 68.10 | 0.60 |
| 515 | Benzo[a]pyrene | 116.70 | 116.20 | 116.10 | 0.60 |
| 516 | n-Dodecanal | 70.20 | 69.80 | 69.60 | 0.60 |
| 517 | (Z) 10-Dodecenal | 71.00 | 70.50 | 70.40 | 0.60 |
| 518 | 1-Nitropropane | 43.90 | 43.40 | 43.30 | 0.60 |
| 519 | Diethyl methylphosphonate | 55.90 | 55.30 |  | 0.60 |
| 520 | Benzaldehyde dimethyl acetal | 60.90 | 60.20 | 60.30 | 0.60 |
| 521 | 1,1-Dichlorononane | 62.30 | 61.30 | 61.70 | 0.60 |
| 522 | 2,5-Dimethylheptane | 43.30 | 42.80 | 42.70 | 0.60 |
| 523 | 3,5-Dimethylheptane | 43.30 | 42.80 | 42.70 | 0.60 |
| 524 | cis-3,4-Dimethyl-2-hexene | 39.30 | 38.80 | 38.70 | 0.60 |
| 525 | Di-n-butylsulfide | 54.30 | 53.90 | 53.70 | 0.60 |
| 526 | Phenylacetate | 53.30 | 53.00 | 52.70 | 0.60 |
| 527 | 1-Tridecene | 65.30 | 64.70 | 64.70 | 0.60 |
| 528 | 2,8-Dichlorodibenzofuran | 74.20 | 74.80 | 74.80 | -0.60 |
| 529 | 4-Amino-N-(2,3-dichlorophenyl)benzene sulfonamide | 114.30 | 113.60 | 113.70 | 0.60 |
| 530 | Azacyclononan-2-one | 85.30 | 85.00 | 84.70 | 0.60 |
| 531 | Diethylperoxide | 30.00 | 28.80 | 29.40 | 0.60 |
| 532 | 2,2,5-Trimethylhexane | 40.20 | 39.60 | 39.60 | 0.60 |
| 533 | cis-1,2-Dimethylcyclopentane | 35.80 | 36.50 | 36.40 | -0.60 |
| 534 | Dimethyldipropylsilane | 40.20 | 39.30 | 39.60 | 0.60 |
| 535 | (E) 6-Decen-1-ol | 80.60 | 80.10 | 80.00 | 0.60 |
| 536 | 1,2,3,7-Tetrachlorodibenzofuran | 84.40 | 84.40 | 85.00 | -0.60 |
| 537 | 1-Iodo-2,6-dimethylbenzene | 57.90 | 58.70 | 58.50 | -0.60 |
| 538 | 1-Pentanol | 55.40 | 56.00 | 56.00 | -0.60 |
| 539 | 2,3-Dimethyloctane | 48.10 | 47.50 | 47.50 | 0.60 |
| 540 | 2-Fluoro-5-methylaniline | 56.90 | 57.80 | 57.50 | -0.60 |
| 541 | 2-Methyl-1-butanol | 54.10 | 53.30 | 53.50 | 0.60 |
| 542 | 3,4-Dimethyloctane | 48.10 | 47.50 | 47.50 | 0.60 |
| 543 | 3-Chlorododecane | 65.90 | 66.40 | 66.50 | -0.60 |
| 544 | 3-Ethyl-2-methylheptane | 48.10 | 47.50 | 47.50 | 0.60 |
| 545 | 3-Ethyl-4-methylheptane | 48.10 | 47.50 | 47.50 | 0.60 |
| 546 | 5-Chlorododecane | 65.90 | 66.40 | 66.50 | -0.60 |
| 547 | 5-Ethyl-2-methylheptane | 48.10 | 47.50 | 47.50 | 0.60 |
| 548 | Cyclohexanone | 46.60 | 45.50 | 46.00 | 0.60 |
| 549 | Ethyl N,N-dimethylglycinate | 47.60 | 47.20 | 47.00 | 0.60 |
| 550 | s-Butylethylamine | 37.90 | 38.80 | 38.50 | -0.60 |
| 551 | Octyl propanoate | 66.40 | 67.10 | 67.00 | -0.60 |
| 552 | Propanoic acid | 54.40 | 54.50 | 55.00 | -0.60 |
| 553 | 1-Pentyne | 28.40 | 29.10 | 29.00 | -0.60 |
| 554 | 2,3-Dimethylbutane | 29.10 | 28.50 | 28.50 | 0.60 |
| 555 | 1,3-Dichloropropane | 41.00 | 41.50 | 41.60 | -0.60 |
| 556 | 2-Methyl-1-pentene | 30.50 | 31.10 | 31.10 | -0.60 |
| 557 | 3-Tolunitrile | 57.50 | 58.20 | 58.10 | -0.60 |
| 558 | 4-Fluorophenol | 60.00 | 60.60 | 60.60 | -0.60 |
| 559 | Bicyclo[2.2.2]octan-2-one | 52.00 | 52.60 | 52.60 | -0.60 |
| 560 | Ethyl octanoate | 62.00 | 62.50 | 62.60 | -0.60 |
| 561 | Fluorocyclohexane | 37.50 | 37.90 | 38.10 | -0.60 |
| 562 | 3-Methyl-1-butyne | 25.80 | 26.40 | 26.40 | -0.60 |
| 563 | cis-1,3-Pentadiene | 28.30 | 28.60 | 28.90 | -0.60 |
| 564 | Trimethylethylene | 27.10 | 27.80 | 27.70 | -0.60 |
| 565 | (E) 5-Hexadecenal | 88.60 | 89.20 | 89.20 | -0.60 |
| 566 | (E) 7-Hexadecenal | 88.60 | 89.20 | 89.20 | -0.60 |
| 567 | (Z) 4-Decen-1-ol | 79.60 | 80.10 | 80.20 | -0.60 |
| 568 | 2-Bromonaphthalene | 66.10 | 66.90 | 66.70 | -0.60 |
| 569 | Acrylic acid | 53.10 | 54.00 | 53.70 | -0.60 |
| 570 | Butyl 3-methylbut-2-enoate | 56.60 | 57.20 | 57.20 | -0.60 |
| 571 | 1-Phenyldodecane | 89.60 | 90.30 | 90.20 | -0.60 |
| 572 | Hexyl propanoate | 57.10 | 57.80 | 57.70 | -0.60 |
| 573 | Triisopentyl phosphate | 86.60 | 87.00 | 87.20 | -0.60 |
| 574 | s-Butylacetate | 40.70 | 41.40 | 41.30 | -0.60 |
| 575 | Isopentyl isobutyrate | 51.70 | 52.30 | 52.30 | -0.60 |
| 576 | 1,3-Dimethylbenzene | 42.70 | 43.30 | 43.30 | -0.60 |
| 577 | (E) 3-Octadecenyl acetate | 109.30 | 109.90 | 109.90 | -0.60 |
| 578 | Glycerol tri(2-methylpropanoate) | 95.30 | 95.90 | 95.90 | -0.60 |
| 579 | 2-Propylpentanoic acid | 74.80 | 75.20 | 75.40 | -0.60 |
| 580 | 3,4-Difluoroaniline | 53.30 | 53.90 | 53.90 | -0.60 |
| 581 | 4-Bromotoluene | 47.30 | 48.00 | 47.90 | -0.60 |
| 582 | 2-Methylaminoethanol | 57.80 | 58.30 | 58.40 | -0.60 |
| 583 | n-Nonanal | 55.30 | 55.80 | 55.90 | -0.60 |
| 584 | (E) 6-Hexadecenal | 88.50 | 89.20 | 89.10 | -0.60 |
| 585 | (Z) 11-Hexadecenal | 88.50 | 89.20 | 89.10 | -0.60 |
| 586 | 1-Undecanol | 83.50 | 84.00 | 84.10 | -0.60 |
| 587 | 2-Nitro-t-butylbenzene | 65.00 | 65.90 | 65.60 | -0.60 |
| 588 | 1-Decanethiol | 65.50 | 66.00 | 66.10 | -0.60 |
| 589 | (E) 7-Tetradecenal | 79.20 | 79.90 | 79.80 | -0.60 |
| 590 | (Z) 4-Tetradecenal | 79.20 | 79.90 | 79.80 | -0.60 |
| 591 | 3,4,4'-Tribromodiphenyl ether | 86.70 | 87.50 | 87.30 | -0.60 |
| 592 | 6,6-Dimethylfulvene | 44.18 | 44.80 | 44.80 | -0.62 |
| 593 | 4-Hydroxy-4-methyl-2-pentanone | 52.13 | 50.40 | 51.50 | 0.63 |
| 594 | (E) 11-Tetradecenal | 80.50 | 79.90 | 79.80 | 0.70 |
| 595 | Benzoic acid | 75.80 | 75.30 | 75.10 | 0.70 |
| 596 | (-)-Myrtenal | 55.10 | 54.80 | 54.40 | 0.70 |
| 597 | 1,1,1-Trimethoxy-2-methylpropane | 44.10 | 43.30 | 43.40 | 0.70 |
| 598 | 1,8-Dichlorooctane | 65.60 | 64.90 | 64.90 | 0.70 |
| 599 | 1-Thiaindan | 59.10 | 59.40 | 58.40 | 0.70 |
| 600 | 3-t-Butylbiphenyl | 77.10 | 76.20 | 76.40 | 0.70 |
| 601 | Pentadecylbenzene | 104.60 | 104.10 | 103.90 | 0.70 |
| 602 | Pentanenitrile | 43.60 | 43.10 | 42.90 | 0.70 |
| 603 | Benzo[ghi]perylene | 128.90 | 129.40 | 129.60 | -0.70 |
| 604 | 1,2,3,5-Tetrafluorobenzene | 36.00 | 35.70 | 35.30 | 0.70 |
| 605 | 3,5-Lutidine | 48.50 | 48.30 | 47.80 | 0.70 |
| 606 | Phenylisocyanate | 46.50 | 46.20 | 45.80 | 0.70 |
| 607 | 1-Iodododecane | 79.90 | 79.30 | 79.20 | 0.70 |
| 608 | 2-Nitrobutane | 46.40 | 45.70 |  | 0.70 |
| 609 | Ethyl trans-cinnamate | 70.40 | 69.90 | 69.70 | 0.70 |
| 610 | 1-Ethylthiooctane | 63.90 | 63.30 | 63.20 | 0.70 |
| 611 | Isoamyl acetate | 46.40 | 45.70 | 45.70 | 0.70 |
| 612 | Isopropyl trichloroacetate | 51.90 | 51.10 | 51.20 | 0.70 |
| 613 | 2-Methyl-2-propanol | 44.90 | 44.60 | 44.20 | 0.70 |
| 614 | Isobutyl chloride | 31.70 | 32.40 | 32.40 | -0.70 |
| 615 | 1-Dodecene | 60.80 | 60.10 | 60.10 | 0.70 |
| 616 | 2,3-Dimethylhexane | 38.80 | 38.00 | 38.10 | 0.70 |
| 617 | m-Toluidine | 58.30 | 58.00 | 57.60 | 0.70 |
| 618 | Cyclobutanecarbonitrile | 44.30 | 43.50 | 43.60 | 0.70 |
| 619 | Isopropyl propyl sulfide | 41.80 | 41.10 | 41.10 | 0.70 |
| 620 | 1-Methyl-4-isopropylcyclohexene | 49.30 | 48.50 | 48.60 | 0.70 |
| 621 | n-Propyl acetate | 39.80 | 39.10 | 39.10 | 0.70 |
| 622 | Thiophene | 35.20 | 35.60 | 35.90 | -0.70 |
| 623 | trans-3,4-Dimethyl-2-hexene | 39.30 | 38.80 | 38.60 | 0.70 |
| 624 | 3-Methyl-1,2-butadiene | 28.00 | 27.30 |  | 0.70 |
| 625 | (E) 2-Tetradecenyl acetate | 90.30 | 91.20 | 91.00 | -0.70 |
| 626 | (Z) 5-Dodecen-1-ol | 90.20 | 89.40 | 89.50 | 0.70 |
| 627 | 1-Octanol | 69.30 | 70.00 | 70.00 | -0.70 |
| 628 | Di-n-pentyl oxalate | 81.30 | 82.20 | 82.00 | -0.70 |
| 629 | Methyl Z,Z-9,12-octadecadieneoate | 107.80 | 108.50 | 108.50 | -0.70 |
| 630 | 1-Chloropentane | 38.80 | 39.80 | 39.50 | -0.70 |
| 631 | Butyronitrile | 39.20 | 38.40 | 38.50 | 0.70 |
| 632 | 2-Methyl-3-ethyl-2-pentene | 39.30 | 40.20 | 40.00 | -0.70 |
| 633 | 3-Fluoro-2-methylaniline | 57.80 | 57.80 | 58.50 | -0.70 |
| 634 | 4-Methyl-1,3-dioxane | 39.20 | 38.80 | 38.50 | 0.70 |
| 635 | alpha-Phellandrene | 48.30 | 49.20 | 49.00 | -0.70 |
| 636 | Hexamethylenimine | 46.70 | 46.00 | 46.00 | 0.70 |
| 637 | Butyl vinyl ether | 36.70 | 35.90 | 36.00 | 0.70 |
| 638 | cis-3,4-Dimethyl-2-pentene | 34.70 | 34.20 | 34.00 | 0.70 |
| 639 | Diisobutylsulfide | 48.70 | 48.50 | 48.00 | 0.70 |
| 640 | Ethyl nonyl ether | 60.30 | 61.20 | 61.00 | -0.70 |
| 641 | Chloroethane | 24.80 | 25.80 | 25.50 | -0.70 |
| 642 | Methanethiol | 23.80 | 24.50 | 24.50 | -0.70 |
| 643 | 2-Diethylaminoethylamine | 45.80 | 46.50 | 46.50 | -0.70 |
| 644 | Butanal | 33.20 | 32.50 | 32.50 | 0.70 |
| 645 | trans,trans-2,4-Hexadienyl acetate | 54.70 | 54.60 | 54.00 | 0.70 |
| 646 | trans-5-Methyl-2-heptene | 39.30 | 40.20 | 40.00 | -0.70 |
| 647 | 1,2-Dichloropentane | 44.40 | 44.80 | 45.10 | -0.70 |
| 648 | 1,3-Diethoxypropane | 45.90 | 46.90 | 46.60 | -0.70 |
| 649 | 4-Propyltoluene | 51.90 | 52.50 | 52.60 | -0.70 |
| 650 | N-Ethylformamide | 58.40 | 59.20 | 59.10 | -0.70 |
| 651 | o-Xylene | 42.90 | 43.30 | 43.60 | -0.70 |
| 652 | trans-3,4-Dimethyl-2-pentene | 35.10 | 34.20 | 34.40 | 0.70 |
| 653 | (E) 2-Dodecenyl acetate | 81.00 | 81.90 | 81.70 | -0.70 |
| 654 | (E) 9-Dodecenyl acetate | 81.00 | 81.90 | 81.70 | -0.70 |
| 655 | (Z) 8-Decenal | 60.50 | 61.20 | 61.20 | -0.70 |
| 656 | 1,2-Dibromopentane | 49.00 | 49.80 | 49.70 | -0.70 |
| 657 | 1,4-Diiodobutane | 59.00 | 59.70 | 59.70 | -0.70 |
| 658 | 1-Iodononane | 64.50 | 65.30 | 65.20 | -0.70 |
| 659 | 4-Methylnonane | 49.50 | 50.30 | 50.20 | -0.70 |
| 660 | 4-t-Butylcatechol | 96.50 | 97.00 | 97.20 | -0.70 |
| 661 | 5-Ethyl-5-methyldecane | 60.50 | 61.30 | 61.20 | -0.70 |
| 662 | Pentanoic acid | 63.00 | 63.90 | 63.70 | -0.70 |
| 663 | 2-Methyl-2-pentene | 31.60 | 32.40 | 32.30 | -0.70 |
| 664 | 3,3,6,6-Tetraethyl-1,2,4,5-tetroxane | 50.10 | 50.80 | 50.80 | -0.70 |
| 665 | 3-Methylhexane | 35.10 | 36.00 | 35.80 | -0.70 |
| 666 | 1,1-Dimethylbutyl acetate | 45.60 | 46.30 | 46.30 | -0.70 |
| 667 | N,N,N',N'-Tetramethyl-1,3-propanediamine | 44.10 | 44.90 | 44.80 | -0.70 |
| 668 | n-Heptylacetate | 57.10 | 57.80 | 57.80 | -0.70 |
| 669 | 2,2-Difluorononane | 46.70 | 47.50 | 47.40 | -0.70 |
| 670 | 8,8-Dimethyl-6,10-dioxaspiro[4.5]decane | 53.70 | 54.40 | 54.40 | -0.70 |
| 671 | cis-2-pentenenitrile | 43.20 | 43.80 | 43.90 | -0.70 |
| 672 | 1,11-Undecanediamine | 84.70 | 85.20 | 85.40 | -0.70 |
| 673 | (E) 8-Hexadecenal | 88.40 | 89.20 | 89.10 | -0.70 |
| 674 | 2-(1-Hydroxy-1-methylethyl)-2-methyl-1,3-dioxane | 67.40 | 68.30 | 68.10 | -0.70 |
| 675 | Lauronitrile | 74.90 | 75.80 | 75.60 | -0.70 |
| 676 | 1,4-Dihydroxybenzene | 84.40 | 85.20 | 85.10 | -0.70 |
| 677 | TEGDN | 88.40 | 90.00 | 89.10 | -0.70 |
| 678 | Nopinene | 43.47 | 44.50 | 44.20 | -0.73 |
| 679 | trans-1,2-Dichloroethylene | 29.47 | 29.40 | 28.70 | 0.77 |
| 680 | 3,4-Dihydro-2H-pyran | 34.92 | 35.70 | 35.70 | -0.78 |
| 681 | Tetrahydrofuran | 32.29 | 31.70 | 31.50 | 0.79 |
| 682 | (Z) 3-Hexadecenal | 89.90 | 89.20 | 89.10 | 0.80 |
| 683 | 1-Bromohexadecane | 94.40 | 93.70 | 93.60 | 0.80 |
| 684 | O,O-Diethyl O-3-diethylaminopropyl thiophosphate | 81.40 | 80.40 | 80.60 | 0.80 |
| 685 | Diethyl cellosolve | 43.20 | 42.20 | 42.40 | 0.80 |
| 686 | 2,3,4-Trifluoroaniline | 53.70 | 53.70 | 52.90 | 0.80 |
| 687 | 2-Methyl-2-pentanol | 54.70 | 53.90 | 53.90 | 0.80 |
| 688 | 3-Ethyl-3-methylheptane | 47.70 | 47.10 | 46.90 | 0.80 |
| 689 | 2-Hexanone | 43.10 | 42.30 | 42.30 | 0.80 |
| 690 | 2-Octanone | 52.60 | 51.70 | 51.80 | 0.80 |
| 691 | 3-Methyl-2-butanol | 51.60 | 50.90 | 50.80 | 0.80 |
| 692 | 1,6-Hexanediamine | 63.10 | 61.90 | 62.30 | 0.80 |
| 693 | 1,3,5-Trimethylbenzene | 47.60 | 47.00 | 46.80 | 0.80 |
| 694 | (E) 13-Hexadecenal | 90.00 | 89.20 | 89.20 | 0.80 |
| 695 | (E) 15-Octadecenyl acetate | 110.50 | 109.90 | 109.70 | 0.80 |
| 696 | 2-Ethylnitrobenzene | 63.00 | 62.50 | 62.20 | 0.80 |
| 697 | 1-Tetradecylamine | 85.50 | 85.00 | 84.70 | 0.80 |
| 698 | 2-Ethyl-4-methyl-1-pentene | 38.50 | 37.60 | 37.70 | 0.80 |
| 699 | 2-Methylnonane | 51.00 | 50.30 | 50.20 | 0.80 |
| 700 | 2-Phenyloctane | 70.00 | 69.10 | 69.20 | 0.80 |
| 701 | Diethylketone | 38.50 | 37.70 | 37.70 | 0.80 |
| 702 | 4-Nonanol | 71.50 | 72.30 | 72.30 | -0.80 |
| 703 | 6-Chlorododecane | 65.50 | 66.40 | 66.30 | -0.80 |
| 704 | Isobutyl isobutyrate | 48.50 | 47.70 | 47.70 | 0.80 |
| 705 | Methyl isopropyl sulfone | 70.00 | 70.60 | 70.80 | -0.80 |
| 706 | Nitrobenzene | 54.50 | 54.10 | 53.70 | 0.80 |
| 707 | Propyl 4-hydroxybenzoate | 89.50 | 88.90 | 88.70 | 0.80 |
| 708 | Perfluoro-2-methylpentane | 31.40 | 29.90 | 30.60 | 0.80 |
| 709 | 1-Propanol | 47.40 | 46.70 | 46.60 | 0.80 |
| 710 | 2-Methyl-1-hexene | 35.10 | 35.70 | 35.90 | -0.80 |
| 711 | 3-Ethyl-3-methylhexane | 42.90 | 42.30 | 42.10 | 0.80 |
| 712 | 3-Ethylpentane | 35.10 | 36.00 | 35.90 | -0.80 |
| 713 | Cyclohexyl propanoate | 59.40 | 58.60 | 58.60 | 0.80 |
| 714 | N,N-Diethyl-1,3-propanediamine | 52.40 | 51.20 | 51.60 | 0.80 |
| 715 | 1,1,1,2,2-Pentafluoro-3-methoxypropane | 31.60 | 31.00 | 30.80 | 0.80 |
| 716 | MTBE | 29.60 | 28.90 | 28.80 | 0.80 |
| 717 | (E) 8-Decenal | 60.20 | 61.20 | 61.00 | -0.80 |
| 718 | 1,1,1,2-Tetrafluoro-2-(trifluoromethoxy)butane | 30.30 | 29.50 |  | 0.80 |
| 719 | 1,1,2-Trimethylcyclopentane | 37.20 | 38.00 | 38.00 | -0.80 |
| 720 | 1-Butyne | 23.70 | 24.50 | 24.50 | -0.80 |
| 721 | 1-Ethylpiperidine | 40.80 | 40.20 | 40.00 | 0.80 |
| 722 | 4-Bromoiodobenzene | 59.80 | 59.10 | 59.00 | 0.80 |
| 723 | Benzyl t-butyl ether | 57.30 | 56.40 | 56.50 | 0.80 |
| 724 | cis-3,4-Dimethyl-3-hexene | 39.70 | 40.20 | 40.50 | -0.80 |
| 725 | cis-5-Methyl-2-hexene | 34.70 | 35.60 | 35.50 | -0.80 |
| 726 | Diisopropyl ether | 32.70 | 33.00 | 33.50 | -0.80 |
| 727 | Tetracyclo[6,2,1,02,7,03,5]undecane | 55.30 | 54.20 | 54.50 | 0.80 |
| 728 | (Z) 7-Decen-1-ol | 80.80 | 80.10 | 80.00 | 0.80 |
| 729 | 1,1-Diphenylethylene | 71.20 | 72.00 | 72.00 | -0.80 |
| 730 | 1,12-Dodecanediamine | 89.20 | 89.90 | 90.00 | -0.80 |
| 731 | Cyclohexyl valerate | 67.20 | 67.90 | 68.00 | -0.80 |
| 732 | Pentadecane | 75.70 | 76.80 | 76.50 | -0.80 |
| 733 | Myristonitrile | 84.20 | 85.10 | 85.00 | -0.80 |
| 734 | 1-Octyne | 42.30 | 42.90 | 43.10 | -0.80 |
| 735 | 2-Propyl-1-pentene | 39.30 | 40.30 | 40.10 | -0.80 |
| 736 | 3-Methyl-cis-2-pentene | 31.30 | 32.40 | 32.10 | -0.80 |
| 737 | Methyl acrylate | 35.80 | 36.40 | 36.60 | -0.80 |
| 738 | cis-5-Methyl-2-heptene | 39.30 | 40.20 | 40.10 | -0.80 |
| 739 | Formic acid | 46.30 | 48.50 | 47.10 | -0.80 |
| 740 | Methyl cyclopropanecarboxylate | 41.30 | 42.10 | 42.10 | -0.80 |
| 741 | t-Amyl butyrate | 50.30 | 51.00 | 51.10 | -0.80 |
| 742 | (E) 11-Pentadecenyl acetate | 94.90 | 95.90 | 95.70 | -0.80 |
| 743 | 1,1,4,6-Tetramethylindane | 61.40 | 61.70 | 62.20 | -0.80 |
| 744 | 1,7-Heptanediamine | 67.10 | 66.60 | 66.30 | 0.80 |
| 745 | 3-Difluoromethoxy-1,1,2,2-tetrafluoropropane | 35.90 | 35.50 | 36.70 | -0.80 |
| 746 | 5-Nonanol | 71.40 | 72.30 | 72.20 | -0.80 |
| 747 | Dimethyl sebacate | 86.40 | 87.20 | 87.20 | -0.80 |
| 748 | n-Pentyl-2-mercaptan | 38.40 | 39.20 | 39.20 | -0.80 |
| 749 | Tetradecylcyclopentane | 95.40 | 96.30 | 96.20 | -0.80 |
| 750 | 3-Ethylheptane | 44.50 | 45.50 | 45.30 | -0.80 |
| 751 | Propyl nitrite | 32.00 | 32.70 | 32.80 | -0.80 |
| 752 | Tricyclo[4.1.0.02.4]heptane | 36.50 | 37.40 | 37.30 | -0.80 |
| 753 | (E) 11-Octadecenyl acetate | 109.10 | 109.90 | 109.90 | -0.80 |
| 754 | (E) 5-Tetradecenal | 79.10 | 79.90 | 79.90 | -0.80 |
| 755 | (E) 7-Dodecenal | 69.60 | 70.50 | 70.40 | -0.80 |
| 756 | (Z) 12-Pentadecenyl acetate | 95.10 | 95.90 | 95.90 | -0.80 |
| 757 | (Z) 9-Tetradecenal | 79.10 | 79.90 | 79.90 | -0.80 |
| 758 | 1,2-Dipropoxyethane | 50.60 | 51.50 | 51.40 | -0.80 |
| 759 | 2,2',4,4'-Tetrabromodiphenyl ether | 94.60 | 95.30 | 95.40 | -0.80 |
| 760 | 2-Heptanol | 62.10 | 63.00 | 62.90 | -0.80 |
| 761 | 3-Fluorobenzonitrile | 53.60 | 54.30 | 54.40 | -0.80 |
| 762 | Acenaphthylene | 69.10 | 69.60 | 69.90 | -0.80 |
| 763 | Benzoyl bromide | 58.60 | 59.40 |  | -0.80 |
| 764 | 1-Methoxydecane | 62.60 | 63.70 | 63.40 | -0.80 |
| 765 | Dicyclohexyl oxalate | 92.10 | 93.10 | 92.90 | -0.80 |
| 766 | N-Cyclopentylimidazole | 70.60 | 71.10 | 71.40 | -0.80 |
| 767 | (E) 12-Hexadecenyl acetate | 99.80 | 100.50 | 100.60 | -0.80 |
| 768 | (E) 8-Dodecenal | 69.80 | 70.50 | 70.60 | -0.80 |
| 769 | Diethyleneglycol-di-n-butylether | 73.80 | 74.60 | 74.60 | -0.80 |
| 770 | 1-Ethylnaphthalene | 67.42 | 66.60 | 66.60 | 0.82 |
| 771 | 2,5-Dimethyltetrahydrofuran | 35.08 | 36.20 | 35.90 | -0.82 |
| 772 | Acetaldoxime | 49.03 | 48.20 |  | 0.83 |
| 773 | Diethyl phthalate | 82.42 | 83.30 | 83.30 | -0.88 |
| 774 | 1,1,1-Trifluoro-2-propanol | 44.80 | 44.20 | 43.90 | 0.90 |
| 775 | (E,E)-9,11-Tetradecenyl acetate | 92.30 | 91.90 | 91.40 | 0.90 |
| 776 | N-Phenylpiperidine | 64.30 | 64.70 | 63.40 | 0.90 |
| 777 | 1-Chloro-2,2-dimethylpropane | 34.70 | 33.90 | 33.80 | 0.90 |
| 778 | 2-Adamantanone | 60.70 | 59.70 | 59.80 | 0.90 |
| 779 | Ethyl isothiocyanate | 40.20 | 39.30 |  | 0.90 |
| 780 | Pentafluorobenzene | 36.20 | 35.50 | 35.30 | 0.90 |
| 781 | 1,1,1,2-Tetrachloroethane | 41.10 | 40.20 | 40.20 | 0.90 |
| 782 | 2-Bromoiodobenzene | 59.60 | 59.10 | 58.70 | 0.90 |
| 783 | 2,3-Dimethylheptane | 43.60 | 42.80 | 42.70 | 0.90 |
| 784 | 2-Fluorobenzonitrile | 55.10 | 54.30 | 54.20 | 0.90 |
| 785 | 3,4-Dimethylheptane | 43.60 | 42.80 | 42.70 | 0.90 |
| 786 | 3-Ethyl-4-methylhexane | 43.60 | 42.80 | 42.70 | 0.90 |
| 787 | 4-Benzylpiperidine | 72.40 | 73.30 | 73.30 | -0.90 |
| 788 | 2-Methoxy-p-cresol | 70.90 | 71.40 | 71.80 | -0.90 |
| 789 | Propyl propanoate | 44.60 | 43.80 | 43.70 | 0.90 |
| 790 | 2-Isopropyltoluene | 50.60 | 49.80 | 49.70 | 0.90 |
| 791 | Perfluoro-3-methylpentane | 30.80 | 29.90 | 29.90 | 0.90 |
| 792 | 1,3-Dichlorobenzene | 47.00 | 46.00 | 46.10 | 0.90 |
| 793 | 2-Methyl-4-methylene-1,3-dioxolane | 37.00 | 36.40 | 36.10 | 0.90 |
| 794 | 3,4-Dimethylhexane | 39.00 | 38.00 | 38.10 | 0.90 |
| 795 | 4,5-Dimethyloctane | 48.50 | 47.50 | 47.60 | 0.90 |
| 796 | iso-Propyl nitrite | 31.00 | 30.30 | 30.10 | 0.90 |
| 797 | Caproic aldehyde | 42.50 | 41.80 | 41.60 | 0.90 |
| 798 | cis-2,4,6-Trimethyl-1,3,5-trioxane | 41.00 | 40.40 | 40.10 | 0.90 |
| 799 | Perfluorocyclohexane | 36.00 | 37.10 | 36.90 | -0.90 |
| 800 | t-Butylacetate | 38.00 | 37.00 | 37.10 | 0.90 |
| 801 | Trimethylphosphate | 47.50 | 46.70 | 46.60 | 0.90 |
| 802 | 2,3-Dimethyl-1-butene | 29.20 | 28.40 | 28.30 | 0.90 |
| 803 | 5-Chloro-1,2,3,4,5-pentafluoro-1,3-cyclopentadiene | 28.70 | 28.20 | 27.80 | 0.90 |
| 804 | Dimethyl sulfide | 27.90 | 27.00 | 27.00 | 0.90 |
| 805 | (1-Iodoethyl)benzene | 59.90 | 59.00 |  | 0.90 |
| 806 | (E) 5-Dodecenal | 69.60 | 70.50 | 70.50 | -0.90 |
| 807 | (Z) 3-Dodecenal | 69.60 | 70.50 | 70.50 | -0.90 |
| 808 | Hexyl bromide | 46.10 | 47.00 | 47.00 | -0.90 |
| 809 | Myristyl alcohol | 98.90 | 98.10 | 98.00 | 0.90 |
| 810 | 2-Heptanone | 46.10 | 47.00 | 47.00 | -0.90 |
| 811 | 2-Pentadecanone | 85.40 | 84.40 | 84.50 | 0.90 |
| 812 | Methyl-n-propylketone | 38.40 | 37.70 | 37.50 | 0.90 |
| 813 | Diethyl sulfate | 56.90 | 56.00 |  | 0.90 |
| 814 | Diisopropyl malonate | 63.90 | 63.40 | 63.00 | 0.90 |
| 815 | gamma-Terpinene | 51.40 | 50.60 | 50.50 | 0.90 |
| 816 | Methyl Z 13-docoseneoate | 125.60 | 126.40 | 126.50 | -0.90 |
| 817 | N,N,N',N'-Tertramethylethylenediamine | 41.40 | 40.20 | 40.50 | 0.90 |
| 818 | Perfluoro-1,3-dimethylcyclohexane | 38.60 | 39.40 | 39.50 | -0.90 |
| 819 | trans-2-Hexahydroindanone | 56.10 | 57.30 | 57.00 | -0.90 |
| 820 | 1-Iodo-2,4-dimethylbenzene | 57.70 | 58.70 | 58.60 | -0.90 |
| 821 | n-Octylacetate | 61.70 | 62.50 | 62.60 | -0.90 |
| 822 | Amyl propionate | 52.20 | 53.10 | 53.10 | -0.90 |
| 823 | trans-4-Methyl-2-hexene | 34.70 | 35.60 | 35.60 | -0.90 |
| 824 | Ethylisopropylether | 30.00 | 30.70 | 30.90 | -0.90 |
| 825 | 1,2,3,4-Tetrahydrodibenzothiophene | 75.30 | 75.90 | 76.20 | -0.90 |
| 826 | 2-Pyrrolecarbonitrile | 65.30 | 66.20 | 66.20 | -0.90 |
| 827 | Undecyl acetate | 77.20 | 76.50 | 76.30 | 0.90 |
| 828 | 3-Methylheptane | 39.80 | 40.80 | 40.70 | -0.90 |
| 829 | Cyclohexyl t-amyl ether | 54.30 | 55.20 | 55.20 | -0.90 |
| 830 | N-t-Butylaminotriethylsilane | 40.30 | 39.80 | 41.20 | -0.90 |
| 831 | Perfluoro-trans-decahydronaphthalene | 51.30 | 52.50 | 52.20 | -0.90 |
| 832 | trans-6-Methyl-2-heptene | 39.30 | 40.20 | 40.20 | -0.90 |
| 833 | Perfluorotributylamine | 60.30 | 61.20 | 61.20 | -0.90 |
| 834 | Bicyclo[4.1.0]hept-3-ene | 38.40 | 39.30 | 39.30 | -0.90 |
| 835 | Butyl 2-methylbutyrate | 54.40 | 55.10 | 55.30 | -0.90 |
| 836 | Cyclohexanecarbonitrile | 51.90 | 52.90 | 52.80 | -0.90 |
| 837 | Ethyl butanoate | 42.90 | 43.80 | 43.80 | -0.90 |
| 838 | Methyl t-butylacetate | 43.90 | 45.00 | 44.80 | -0.90 |
| 839 | N-Butylpiperidine | 48.90 | 49.50 | 49.80 | -0.90 |
| 840 | (Z) 8-Decenyl acetate | 71.50 | 72.50 | 72.40 | -0.90 |
| 841 | 2-Piperidone | 70.50 | 71.00 | 71.40 | -0.90 |
| 842 | Bicyclo[5.1.0]octane | 43.50 | 44.40 | 44.40 | -0.90 |
| 843 | Tridecylcyclopentane | 90.50 | 91.60 | 91.40 | -0.90 |
| 844 | 2,4,8-Trichlorodibenzofuran | 78.70 | 79.60 | 79.60 | -0.90 |
| 845 | 2,3-Dihydroxyanisole | 91.70 | 92.00 | 92.60 | -0.90 |
| 846 | N-Methylbenzamide | 84.90 | 86.20 | 85.80 | -0.90 |
| 847 | Benzonitrile | 55.48 | 54.50 | 54.50 | 0.98 |
| 848 | (E) 12-Tetradecenal | 80.80 | 79.90 | 79.80 | 1.00 |
| 849 | (Z) 12-Tetradecenal | 80.80 | 79.90 | 79.80 | 1.00 |
| 850 | Tetramethylene glycol | 79.30 | 78.60 | 78.30 | 1.00 |
| 851 | (E) 7-Decen-1-ol | 81.10 | 80.10 | 80.10 | 1.00 |
| 852 | 1,1-Dinitrobutane | 64.90 | 63.90 |  | 1.00 |
| 853 | 1,2-Dibromobutane | 45.90 | 45.20 | 44.90 | 1.00 |
| 854 | 1H,1H-Perfluorododecan-1-ol | 90.40 | 89.40 | 89.40 | 1.00 |
| 855 | 2,2,4-Trimethyl-1,3-dioxane | 41.90 | 40.70 | 40.90 | 1.00 |
| 856 | 4-Phenyltoluene | 69.40 | 68.40 | 68.40 | 1.00 |
| 857 | 5-Ethyl-m-xylene | 52.40 | 51.60 | 51.40 | 1.00 |
| 858 | Imidazo[1,2-a]pyridine | 67.40 | 66.50 | 66.40 | 1.00 |
| 859 | Methyl 3,3-dimethylacrylate | 46.90 | 45.70 | 45.90 | 1.00 |
| 860 | 1,1,2-Trimethylpropyl butanoate | 53.80 | 53.00 | 52.80 | 1.00 |
| 861 | 4-Fluoroaniline | 54.80 | 54.10 | 53.80 | 1.00 |
| 862 | Dimethyl methylmalonate | 57.80 | 56.50 | 56.80 | 1.00 |
| 863 | (Z) 6-Dodecen-1-ol | 90.20 | 89.40 | 89.20 | 1.00 |
| 864 | n-Decylamine | 65.30 | 66.30 | 66.30 | -1.00 |
| 865 | p-Acetotoluide | 86.20 | 86.00 | 85.20 | 1.00 |
| 866 | Bis(4-methylphenoxy)dimethylsilane | 65.30 | 66.20 | 66.30 | -1.00 |
| 867 | Tri-t-Butylperoxyvinylsilane | 68.80 | 69.80 |  | -1.00 |
| 868 | 2,4,4-Trimethylheptane | 45.20 | 44.30 | 44.20 | 1.00 |
| 869 | Tetramethylhydrazine | 32.90 | 31.90 |  | 1.00 |
| 870 | 1,1,2-Trimethylpropyl 3,3-dimethylbutanoate | 57.10 | 56.30 | 56.10 | 1.00 |
| 871 | 1,1-Dichloro-3,3,3-trifluoropropane | 34.10 | 33.20 | 33.10 | 1.00 |
| 872 | 1,3-Di-t-butylbenzene | 59.60 | 59.00 | 58.60 | 1.00 |
| 873 | Isopentyl chloride | 38.10 | 37.10 | 37.10 | 1.00 |
| 874 | 2,6-Dimethylpiperidine | 43.10 | 42.80 | 42.10 | 1.00 |
| 875 | Norcarane | 40.60 | 39.70 | 39.60 | 1.00 |
| 876 | 3,4-Dihydro-1H-2-benzopyran | 57.10 | 56.10 | 56.10 | 1.00 |
| 877 | t-Butyl propyl ether | 36.60 | 35.70 | 35.60 | 1.00 |
| 878 | (E) 11-Hexadecenyl acetate | 99.50 | 100.50 | 100.50 | -1.00 |
| 879 | (E) 9-Tridecenyl acetate | 85.50 | 86.50 | 86.50 | -1.00 |
| 880 | (Z) 12-Hexadecenyl acetate | 99.50 | 100.50 | 100.50 | -1.00 |
| 881 | 1,1,1-Trimethoxy-2-chloroethane | 47.50 | 47.80 | 48.50 | -1.00 |
| 882 | 1-Chlorodecane | 64.00 | 63.20 | 63.00 | 1.00 |
| 883 | 1-Iodoheptane | 55.00 | 55.90 | 56.00 | -1.00 |
| 884 | 1-Iodotridecane | 85.00 | 83.90 | 84.00 | 1.00 |
| 885 | s-Phenethyl alcohol | 68.50 | 69.70 | 69.50 | -1.00 |
| 886 | 2,2',4,4',5,5'-Hexabromodiphenyl ether | 110.00 | 111.00 | 111.00 | -1.00 |
| 887 | 2,2',4,4',6-Pentabromodiphenyl ether | 102.00 | 103.20 | 103.00 | -1.00 |
| 888 | 2,3-Dimethyl-1-hexene | 38.50 | 37.60 | 37.50 | 1.00 |
| 889 | 2,4-Dimethyl-1-hexene | 38.50 | 37.60 | 37.50 | 1.00 |
| 890 | 2,4-Dimethyloctane | 48.50 | 47.50 | 47.50 | 1.00 |
| 891 | 3,5-Dimethyloctane | 48.50 | 47.50 | 47.50 | 1.00 |
| 892 | 3-Fluorotoluene | 39.00 | 40.00 | 40.00 | -1.00 |
| 893 | 3-Methylthiophene | 39.50 | 38.90 | 38.50 | 1.00 |
| 894 | 4-Methyl-trans-2-pentene | 30.00 | 31.00 | 31.00 | -1.00 |
| 895 | 4-Methyloctane | 44.50 | 45.50 | 45.50 | -1.00 |
| 896 | 7-Heptadecanone | 94.50 | 93.70 | 93.50 | 1.00 |
| 897 | 9-Heptadecanol | 108.50 | 109.70 | 109.50 | -1.00 |
| 898 | Ethyl acrylate | 39.50 | 38.60 | 38.50 | 1.00 |
| 899 | cis-4,5-Dimethyl-2-hexene | 38.50 | 37.50 | 37.50 | 1.00 |
| 900 | Dibutylsulfate | 75.50 | 74.70 | 74.50 | 1.00 |
| 901 | Pivalone | 45.50 | 44.50 | 44.50 | 1.00 |
| 902 | Dipropyl adipate | 81.00 | 82.20 | 82.00 | -1.00 |
| 903 | N,N-Diethylaniline | 56.00 | 56.80 | 55.00 | 1.00 |
| 904 | Triisobutylsilane | 40.00 | 39.50 | 39.00 | 1.00 |
| 905 | 3-Methylpentane | 30.30 | 31.20 | 31.30 | -1.00 |
| 906 | trans-1,3-Pentadiene | 27.80 | 28.60 | 28.80 | -1.00 |
| 907 | 2-Difluoromethoxy-1,1,1,3,3,3-hexafluoropropane | 31.10 | 32.10 |  | -1.00 |
| 908 | Caprylene | 40.60 | 41.70 | 41.60 | -1.00 |
| 909 | 2-Butanol | 48.10 | 49.00 | 49.10 | -1.00 |
| 910 | 5,6,7,8-Tetrahydroquinoline | 57.60 | 58.40 | 58.60 | -1.00 |
| 911 | alpha-Pinene | 44.60 | 45.80 | 45.60 | -1.00 |
| 912 | Ethyl formate | 32.10 | 33.00 | 33.10 | -1.00 |
| 913 | Butyric acid methylester | 40.60 | 41.60 | 41.60 | -1.00 |
| 914 | 1,2-Dichloroheptane | 53.20 | 54.10 | 54.20 | -1.00 |
| 915 | 1-Iodooctane | 59.70 | 60.60 | 60.70 | -1.00 |
| 916 | 4-Methylheptane | 39.70 | 40.80 | 40.70 | -1.00 |
| 917 | cis-1-Ethyl-2-methylcyclopentane | 40.20 | 41.20 | 41.20 | -1.00 |
| 918 | cis-4-Methyl-2-hexene | 34.70 | 35.60 | 35.70 | -1.00 |
| 919 | Dimethylvinylethynylmethanol butyl ether | 55.70 | 56.60 | 56.70 | -1.00 |
| 920 | Ethyl 2-methylbutanoate | 44.70 | 45.70 | 45.70 | -1.00 |
| 921 | Ethyl iodide | 31.70 | 32.60 | 32.70 | -1.00 |
| 922 | 2-Methylheptane | 39.70 | 40.80 | 40.70 | -1.00 |
| 923 | Methyl nicotinate | 61.20 | 62.20 | 62.20 | -1.00 |
| 924 | trans-5-Methyl-2-hexene | 34.70 | 35.60 | 35.70 | -1.00 |
| 925 | (Z) 10-Hexadecenal | 88.20 | 89.20 | 89.20 | -1.00 |
| 926 | Cycloheptanol | 64.70 | 66.20 | 65.70 | -1.00 |
| 927 | Propyl dodecanoate | 84.70 | 85.80 | 85.70 | -1.00 |
| 928 | 1,1-Dimethylpropyl propanoate | 45.30 | 46.30 | 46.30 | -1.00 |
| 929 | 1-Undecene | 54.30 | 55.50 | 55.30 | -1.00 |
| 930 | 3-Pentanethiol | 38.30 | 39.20 | 39.30 | -1.00 |
| 931 | cis-6-Methyl-2-heptene | 39.30 | 40.20 | 40.30 | -1.00 |
| 932 | Ethyl pivalate | 41.30 | 42.50 | 42.30 | -1.00 |
| 933 | Methyl m-toluate | 60.30 | 61.40 | 61.30 | -1.00 |
| 934 | Methyl methacrylate | 38.80 | 39.70 | 39.80 | -1.00 |
| 935 | Diethylnitramine | 47.30 | 48.30 |  | -1.00 |
| 936 | 1,4-Dimethylbenzene | 42.30 | 43.30 | 43.30 | -1.00 |
| 937 | trans-2,2,4,6-Tetramethyl-1,3-dioxane | 42.30 | 43.00 | 43.30 | -1.00 |
| 938 | (2-Methoxyphenyl)trimethylsilane | 59.40 | 60.30 | 60.40 | -1.00 |
| 939 | 2-Ethyltoluene | 46.90 | 47.90 | 47.90 | -1.00 |
| 940 | (Ethylthio)benzene | 56.40 | 57.10 | 57.40 | -1.00 |
| 941 | 6-Heptadecanol | 108.60 | 109.70 | 109.60 | -1.00 |
| 942 | Fluoranthene | 87.10 | 88.40 | 88.10 | -1.00 |
| 943 | (Z) 8-Tetradecenal | 78.80 | 79.90 | 79.80 | -1.00 |
| 944 | Hexadecylcyclohexane | 109.30 | 110.60 | 110.30 | -1.00 |
| 945 | Methyl (dimethylamino) acetate | 43.93 | 45.00 | 45.00 | -1.07 |
| 946 | Quinoline | 59.31 | 60.40 | 60.40 | -1.09 |
| 947 | Methyl Z,Z-13,16-docosadienoate | 127.90 | 127.10 | 126.80 | 1.10 |
| 948 | 1,1-Dichloroundecane | 71.70 | 70.70 | 70.60 | 1.10 |
| 949 | 1,2,3,7,8-Pentachlorodibenzo-p-dioxin | 88.70 | 87.50 | 87.60 | 1.10 |
| 950 | 1-Tetradecene | 70.20 | 69.30 | 69.10 | 1.10 |
| 951 | n-Hexanoic acid | 69.20 | 68.50 | 68.10 | 1.10 |
| 952 | (Z) 6-Tetradecen-1-ol | 100.00 | 98.80 | 98.90 | 1.10 |
| 953 | 2,5-Dihydrothiophene | 40.00 | 39.20 | 38.90 | 1.10 |
| 954 | 7-Oxabicyclo[4.1.0]heptane | 40.50 | 39.40 | 39.40 | 1.10 |
| 955 | Diethyl adipate | 74.00 | 72.80 | 72.90 | 1.10 |
| 956 | Dodecanedioic acid | 130.00 | 131.70 | 131.10 | -1.10 |
| 957 | 3,3-Dimethylbutanoic acid | 64.00 | 62.60 | 62.90 | 1.10 |
| 958 | Isopropyl t-butyl ether | 34.40 | 33.30 | 33.30 | 1.10 |
| 959 | n-Butyl-2-chloropropanoate | 54.40 | 53.40 | 53.30 | 1.10 |
| 960 | N-sec-Pentylaminotriethylsilane | 46.90 | 46.20 | 45.80 | 1.10 |
| 961 | trans-2-Pentenenitrile | 44.90 | 43.80 | 43.80 | 1.10 |
| 962 | 1,6-Dichlorohexane | 56.30 | 55.50 | 55.20 | 1.10 |
| 963 | 1-Chloro-2,6-dimethylbenzene | 49.80 | 48.70 | 48.70 | 1.10 |
| 964 | 2,3-Dimethylpentane | 34.30 | 33.30 | 33.20 | 1.10 |
| 965 | 2-Cyanoethanol | 62.30 | 61.00 | 61.20 | 1.10 |
| 966 | 4-Heptanone | 47.80 | 47.00 | 46.70 | 1.10 |
| 967 | Thiirane | 30.30 | 29.10 | 29.20 | 1.10 |
| 968 | trans-3-Pentenenitrile | 44.80 | 43.80 | 43.70 | 1.10 |
| 969 | (E) 3-Tetradecen-1-ol | 99.80 | 98.80 | 98.70 | 1.10 |
| 970 | Ethyl cis-2-phenylcyclopropanecarboxylate | 70.70 | 71.60 | 71.80 | -1.10 |
| 971 | N-Propylcarbazole | 87.80 | 85.00 | 86.70 | 1.10 |
| 972 | 1,5-Dichloropentane | 52.20 | 50.90 | 51.10 | 1.10 |
| 973 | 1-Iodopropane | 36.30 | 37.20 | 37.40 | -1.10 |
| 974 | 4-tert-Butyltoluene | 52.20 | 51.20 | 51.10 | 1.10 |
| 975 | trans-2-Methyl-3-hexene | 34.30 | 35.60 | 35.40 | -1.10 |
| 976 | (E) 4-Dodecen-1-ol | 90.60 | 89.40 | 89.50 | 1.10 |
| 977 | (Z) 7-Dodecenal | 69.40 | 70.50 | 70.50 | -1.10 |
| 978 | 2-Ethyl-p-xylene | 52.60 | 51.60 | 51.50 | 1.10 |
| 979 | 2-Methylhexane | 34.90 | 36.00 | 36.00 | -1.10 |
| 980 | 3,3',4-Tribromodiphenyl ether | 86.40 | 87.50 | 87.50 | -1.10 |
| 981 | cis-4,4-Dimethyl-2-hexene | 38.10 | 37.00 | 37.00 | 1.10 |
| 982 | cis-5,5-Dimethyl-2-hexene | 38.10 | 37.00 | 37.00 | 1.10 |
| 983 | cis-Diethylcyclopropane | 35.40 | 36.50 | 36.50 | -1.10 |
| 984 | Ethyl 2-diethylamino-2-propanoate | 54.90 | 55.80 | 56.00 | -1.10 |
| 985 | Ethyl acetate | 35.60 | 34.50 | 34.50 | 1.10 |
| 986 | trans-Geraniol | 76.90 | 78.10 | 78.00 | -1.10 |
| 987 | Methyl 3-thiopheneacetate | 60.90 | 61.10 | 62.00 | -1.10 |
| 988 | Pyrene | 92.40 | 93.90 | 93.50 | -1.10 |
| 989 | trans-4,4-Dimethyl-2-hexene | 38.10 | 37.00 | 37.00 | 1.10 |
| 990 | trans-5,5-Dimethyl-2-hexene | 38.10 | 37.00 | 37.00 | 1.10 |
| 991 | (E) 4-Decenal | 60.00 | 61.20 | 61.10 | -1.10 |
| 992 | Propyl crotonate | 48.00 | 49.20 | 49.10 | -1.10 |
| 993 | trans-4,5-Dimethyl-2-hexene | 38.50 | 37.50 | 37.40 | 1.10 |
| 994 | Tris(methylthio)borane | 54.00 | 55.10 |  | -1.10 |
| 995 | 1,2-Dichloro-1,1,2-trifluoroethane | 26.80 | 27.90 | 27.90 | -1.10 |
| 996 | (2-Chloroethyl)(2-chlorocyclopentyl) sulfide | 65.90 | 65.00 | 64.80 | 1.10 |
| 997 | (E) 10-Pentadecenyl acetate | 94.60 | 95.90 | 95.70 | -1.10 |
| 998 | 2-Bromoethanol | 54.10 | 55.60 | 55.20 | -1.10 |
| 999 | 2-Fluoro-4-methylaniline | 56.60 | 57.80 | 57.70 | -1.10 |
| 1000 | Cumene | 45.10 | 46.10 | 46.20 | -1.10 |
| 1001 | 2-Phenylpyridine | 68.40 | 67.60 | 67.30 | 1.10 |
| 1002 | Acetanilide | 82.10 | 82.20 | 83.20 | -1.10 |
| 1003 | O,O-Diethyl O-3-dimethylaminopropyl thiophosphate | 76.90 | 75.60 | 75.80 | 1.10 |
| 1004 | Phenylacetic acid | 79.10 | 79.90 | 80.20 | -1.10 |
| 1005 | trans-3-Methyl-2-hexene | 35.60 | 37.00 | 36.70 | -1.10 |
| 1006 | 1,1-Dimethylpropyl 3,3-dimethylbutanoate | 53.20 | 54.40 | 54.30 | -1.10 |
| 1007 | 1-Heptene | 35.70 | 37.10 | 36.80 | -1.10 |
| 1008 | 3-Ethylhexane | 39.70 | 40.80 | 40.80 | -1.10 |
| 1009 | Diisobutyl disulfide | 57.20 | 58.30 | 58.30 | -1.10 |
| 1010 | (Z) 13-Octadecenyl acetate | 108.80 | 109.90 | 109.90 | -1.10 |
| 1011 | 3-t-Butylphenol | 71.30 | 72.60 | 72.40 | -1.10 |
| 1012 | 9-Methyl-9H-fluorene | 71.30 | 72.50 | 72.40 | -1.10 |
| 1013 | Methyl tetracosanoate | 135.70 | 135.00 | 134.60 | 1.10 |
| 1014 | (+)-alpha-Thujene | 44.80 | 45.80 | 45.90 | -1.10 |
| 1015 | Fenchone | 51.80 | 52.80 | 52.90 | -1.10 |
| 1016 | Propyl 3-chlorobutanoate | 52.30 | 53.40 | 53.40 | -1.10 |
| 1017 | Triethoxy((ethylthio)methyl)silane | 42.30 | 43.30 | 43.40 | -1.10 |
| 1018 | (Z) 11-Tetradecenyl acetate | 90.00 | 91.20 | 91.10 | -1.10 |
| 1019 | 1-Benzosuberone | 69.00 | 70.00 | 70.10 | -1.10 |
| 1020 | Dodecylcyclopentane | 85.50 | 86.80 | 86.60 | -1.10 |
| 1021 | (E) 9-Octadecenyl acetate | 108.70 | 109.90 | 109.80 | -1.10 |
| 1022 | (Z) 7-Tetradecenal | 78.70 | 79.90 | 79.80 | -1.10 |
| 1023 | (Z) 9-Dodecenyl acetate | 80.70 | 81.90 | 81.80 | -1.10 |
| 1024 | Di-n-pentyl glutarate | 95.20 | 96.20 | 96.30 | -1.10 |
| 1025 | 2-Iodofluorene | 87.41 | 86.40 | 86.30 | 1.11 |
| 1026 | trans-1,2-Dibromocyclopentane | 47.82 | 46.90 | 46.70 | 1.12 |
| 1027 | 7-Norbornanone | 46.65 | 48.00 | 47.80 | -1.15 |
| 1028 | Cyclopentanethiol | 41.44 | 42.40 | 42.60 | -1.16 |
| 1029 | Fenthion | 89.31 | 90.50 |  | -1.19 |
| 1030 | (Z) 7-Dodecen-1-ol | 90.50 | 89.40 | 89.30 | 1.20 |
| 1031 | N-(4-(trifluoromethyl)phenyl)-1-thia-3-azaspiro[5.5]undec-2-en-2-amine | 105.30 | 104.50 | 104.10 | 1.20 |
| 1032 | (Z) 7-Tridecen-1-ol | 95.10 | 94.10 | 93.90 | 1.20 |
| 1033 | 1,1,2-Trifluoro-2-chloroethyl 2,2,3,3,4,4,5,5,6,6,7,7-dodecafluoroheptyl ether | 64.60 | 63.60 | 63.40 | 1.20 |
| 1034 | 4,4-Dimethyloctane | 48.10 | 47.10 | 46.90 | 1.20 |
| 1035 | Butyl 1,1-dimethylpropyl ether | 46.10 | 45.00 | 44.90 | 1.20 |
| 1036 | N-Methylpropionamide | 66.60 | 65.40 | 65.40 | 1.20 |
| 1037 | Quinaldine | 62.60 | 61.60 | 61.40 | 1.20 |
| 1038 | S-Propylthioacetate | 44.10 | 44.50 | 42.90 | 1.20 |
| 1039 | 2,4,6-Trimethyl-1,3-Dioxane | 43.00 | 42.10 | 41.80 | 1.20 |
| 1040 | 3-Methyl-2-butanethiol | 37.50 | 36.50 | 36.30 | 1.20 |
| 1041 | 4-Fluoronitrobenzene | 56.00 | 53.90 | 54.80 | 1.20 |
| 1042 | Ethyl 2-pentynoate | 52.00 | 51.20 | 50.80 | 1.20 |
| 1043 | 1,2,4-Trimethylbenzene | 48.00 | 47.00 | 46.80 | 1.20 |
| 1044 | (Z) 7-Tetradecen-1-ol | 99.90 | 98.80 | 98.70 | 1.20 |
| 1045 | 1,10-Dichlorodecane | 73.10 | 74.20 | 74.30 | -1.20 |
| 1046 | Tripropionine | 91.40 | 90.10 | 90.20 | 1.20 |
| 1047 | 1-Azidooctane | 58.90 | 57.90 | 57.70 | 1.20 |
| 1048 | 1-(2-Methylphenyl)ethanone | 58.90 | 57.50 | 57.70 | 1.20 |
| 1049 | 3-Methylpiperidine | 44.40 | 43.20 | 43.20 | 1.20 |
| 1050 | Methylcyclopropylketone | 39.40 | 38.10 | 38.20 | 1.20 |
| 1051 | Ethyl t-butyl ketone | 42.30 | 41.10 | 41.10 | 1.20 |
| 1052 | 3-Fluoronitrobenzene | 54.80 | 53.90 | 53.60 | 1.20 |
| 1053 | 6-Oxabicyclo[3.1.0]hexane | 33.70 | 34.70 | 34.90 | -1.20 |
| 1054 | Bromocyclohexane | 45.30 | 44.10 | 44.10 | 1.20 |
| 1055 | 2-Bromo-2-methylpropane | 31.00 | 29.80 |  | 1.20 |
| 1056 | 1,3,7,9-Tetrachlorodibenzofuran | 83.30 | 84.40 | 84.50 | -1.20 |
| 1057 | 1-Tridecanol | 94.70 | 93.40 | 93.50 | 1.20 |
| 1058 | 2-Piperidineethanol | 75.20 | 74.00 | 74.00 | 1.20 |
| 1059 | Methyl (Z) 11-eicosenoate | 115.80 | 117.10 | 117.00 | -1.20 |
| 1060 | 1,1,2-Trimethylpropyl 2-methylpropanoate | 51.70 | 50.20 | 50.50 | 1.20 |
| 1061 | 1-Bromo-2,6-dimethylbenzene | 52.70 | 51.70 | 51.50 | 1.20 |
| 1062 | 1-Chloro-1-methylcyclopentane | 39.70 | 38.10 | 38.50 | 1.20 |
| 1063 | 1-Methyl-3-piperidinol | 63.70 | 62.60 | 62.50 | 1.20 |
| 1064 | N-Methylpiperidine | 36.80 | 37.80 | 38.00 | -1.20 |
| 1065 | 2,2,4-Trimethylhexane | 40.70 | 39.60 | 39.50 | 1.20 |
| 1066 | 2,4-Difluorophenol | 58.80 | 60.40 | 60.00 | -1.20 |
| 1067 | 2-Methyl-1-heptene | 39.30 | 40.30 | 40.50 | -1.20 |
| 1068 | 3-Fluoroaniline | 54.70 | 54.10 | 53.50 | 1.20 |
| 1069 | 4-Chlorophenylisocyanate | 49.80 | 51.00 | 51.00 | -1.20 |
| 1070 | cis-2-Methyl-3-hexene | 34.30 | 35.60 | 35.50 | -1.20 |
| 1071 | Di-n-butylamine | 50.80 | 52.10 | 52.00 | -1.20 |
| 1072 | Isopropyl propylamine | 37.30 | 38.80 | 38.50 | -1.20 |
| 1073 | 4-Methylpent-3-en-2-one | 42.70 | 41.70 | 41.50 | 1.20 |
| 1074 | N-Methylformamide | 56.20 | 56.80 | 55.00 | 1.20 |
| 1075 | Propyl 2-chlorobutanoate | 52.30 | 53.40 | 53.50 | -1.20 |
| 1076 | (Z) 7-Decenal | 59.90 | 61.20 | 61.10 | -1.20 |
| 1077 | 2-Acetoxy-2,6-dimethylheptane | 56.40 | 57.60 | 57.60 | -1.20 |
| 1078 | 3-Octyne | 43.90 | 45.00 | 45.10 | -1.20 |
| 1079 | cis-4-Ethyl-2-hexene | 38.90 | 40.20 | 40.10 | -1.20 |
| 1080 | cis-5-Methyl-3-heptene | 38.90 | 40.20 | 40.10 | -1.20 |
| 1081 | cis-6-Methyl-3-heptene | 38.90 | 40.20 | 40.10 | -1.20 |
| 1082 | trans-2-Methyl-3-heptene | 38.90 | 40.20 | 40.10 | -1.20 |
| 1083 | trans-6-Methyl-3-heptene | 38.90 | 40.20 | 40.10 | -1.20 |
| 1084 | 3-Methyl-1-butene | 23.90 | 25.10 | 25.10 | -1.20 |
| 1085 | N-Methyl isopropyl amine | 30.70 | 31.70 | 31.90 | -1.20 |
| 1086 | (E) 12-Pentadecenyl acetate | 94.50 | 95.90 | 95.70 | -1.20 |
| 1087 | (Z) 9-Hexadecenal | 88.00 | 89.20 | 89.20 | -1.20 |
| 1088 | trans-1,2-Dibromocyclohexane | 50.50 | 51.50 | 51.70 | -1.20 |
| 1089 | 2,2',3,4,5,5',6-Heptabromodiphenyl ether | 118.00 | 118.90 | 119.20 | -1.20 |
| 1090 | 3-Acetyl-2,5-dimethylfuran | 57.50 | 59.60 | 58.70 | -1.20 |
| 1091 | 3-Ethyloctane | 49.00 | 50.30 | 50.20 | -1.20 |
| 1092 | 1-Amino-3-methoxypropane | 44.00 | 45.20 | 45.20 | -1.20 |
| 1093 | Butanoic acid | 58.50 | 59.20 | 59.70 | -1.20 |
| 1094 | Ethyl decanoate | 70.50 | 71.80 | 71.70 | -1.20 |
| 1095 | Ethyl trans-2-pentenoate | 48.00 | 49.20 | 49.20 | -1.20 |
| 1096 | Isopropyl crotonate | 46.00 | 46.80 | 47.20 | -1.20 |
| 1097 | Methyl crotonate | 41.00 | 42.40 | 42.20 | -1.20 |
| 1098 | n-Heptylamine | 51.00 | 52.30 | 52.20 | -1.20 |
| 1099 | 1,2-Dichlorooctane | 57.60 | 58.80 | 58.80 | -1.20 |
| 1100 | 1,3-Diiodopropane | 54.10 | 55.10 | 55.30 | -1.20 |
| 1101 | 1-Hexanol | 59.60 | 60.70 | 60.80 | -1.20 |
| 1102 | 1-Nitrobutane | 48.10 | 48.10 | 49.30 | -1.20 |
| 1103 | 2-Methoxypyridine | 45.10 | 46.30 |  | -1.20 |
| 1104 | (Chloromethyl)benzene | 50.10 | 51.20 | 51.30 | -1.20 |
| 1105 | Butyl isopropylamine | 42.10 | 43.50 | 43.30 | -1.20 |
| 1106 | 1,1,1,2-Tetramethoxyethane | 47.20 | 48.30 | 48.40 | -1.20 |
| 1107 | 2,5-Dioxaoctane | 43.70 | 44.70 | 44.90 | -1.20 |
| 1108 | 2,4-Dimethyl-2-pentyl propanoate | 51.70 | 53.00 | 52.90 | -1.20 |
| 1109 | 2-Methylthiophene | 38.70 | 39.90 | 39.90 | -1.20 |
| 1110 | (Z) 3-Octadecenyl acetate | 108.70 | 109.90 | 109.90 | -1.20 |
| 1111 | Butyl dodecanoate | 89.20 | 90.50 | 90.40 | -1.20 |
| 1112 | (Z) 4-Dodecenal | 69.40 | 70.50 | 70.60 | -1.20 |
| 1113 | 3-Phenylpyridine | 68.40 | 70.00 | 69.60 | -1.20 |
| 1114 | 4-Iodobenzoic acid | 87.90 | 90.10 | 89.10 | -1.20 |
| 1115 | Tri-p-cresyl phosphate | 113.40 | 113.40 | 114.60 | -1.20 |
| 1116 | Pentadecylcyclohexane | 104.40 | 105.80 | 105.60 | -1.20 |
| 1117 | Methyl octadecanoate | 106.10 | 107.00 | 107.30 | -1.20 |
| 1118 | Tris-(carboethoxy)methane | 79.10 | 80.50 | 80.30 | -1.20 |
| 1119 | Chloropropham | 88.67 | 89.90 |  | -1.23 |
| 1120 | Ethyl amyl ether | 41.06 | 42.50 | 42.30 | -1.24 |
| 1121 | Isobutyl vinyl ether | 34.56 | 33.20 | 33.30 | 1.26 |
| 1122 | 2,7-Difluorofluorene | 72.96 | 71.20 | 71.70 | 1.26 |
| 1123 | Bisabolol | 96.60 | 95.10 | 95.30 | 1.30 |
| 1124 | 2,5-Dimethyl-2,5-hexanediol | 85.20 | 83.70 | 83.90 | 1.30 |
| 1125 | Diheptylamine | 81.20 | 80.10 | 79.90 | 1.30 |
| 1126 | Hexadecanal | 89.70 | 88.50 | 88.40 | 1.30 |
| 1127 | 1,7-Dichloroheptane | 61.20 | 60.20 | 59.90 | 1.30 |
| 1128 | 4-Methylpyrimidine | 44.20 | 43.20 | 42.90 | 1.30 |
| 1129 | Methoxyacetonitrile | 41.70 | 40.60 | 40.40 | 1.30 |
| 1130 | N,N-Dimethyloctylamine | 56.20 | 55.50 | 54.90 | 1.30 |
| 1131 | 1,1-Diethynylcyclopropane | 37.60 | 36.30 | 36.30 | 1.30 |
| 1132 | 2,4,4-Trimethylhexane | 41.10 | 39.60 | 39.80 | 1.30 |
| 1133 | Cyclopentanone | 42.10 | 40.90 | 40.80 | 1.30 |
| 1134 | 1,4-Dioxaspiro[4.4]nonane | 47.60 | 46.30 | 46.30 | 1.30 |
| 1135 | Ethyl benzoate | 61.10 | 59.90 | 59.80 | 1.30 |
| 1136 | 1,3-Dibromopropane | 47.60 | 46.70 | 46.30 | 1.30 |
| 1137 | (+)-trans-Myrtanol | 73.00 | 71.60 | 71.70 | 1.30 |
| 1138 | (Z) 4-Tetradecen-1-ol | 100.00 | 98.80 | 98.70 | 1.30 |
| 1139 | 2-Ethyl-1-hexanol | 68.50 | 67.30 | 67.20 | 1.30 |
| 1140 | Anisole | 44.00 | 43.30 | 42.70 | 1.30 |
| 1141 | Phenylacetonitrile | 60.50 | 59.10 | 59.20 | 1.30 |
| 1142 | 1,1-Dichlorobutane | 39.50 | 38.00 | 38.20 | 1.30 |
| 1143 | trans-Crotononitrile | 40.00 | 39.10 | 38.70 | 1.30 |
| 1144 | trans-Bicyclo[3.3.0]octan-2-one | 54.00 | 52.60 | 52.70 | 1.30 |
| 1145 | 3-Methyl-3-propyl-1-heptene | 50.90 | 49.50 | 49.60 | 1.30 |
| 1146 | Sulcatone | 52.40 | 51.00 | 51.10 | 1.30 |
| 1147 | N-Ethylisopropylamine | 33.10 | 34.20 | 34.40 | -1.30 |
| 1148 | n-Propylformate | 36.60 | 37.70 | 37.90 | -1.30 |
| 1149 | 2,3-Dimethyl-2-butene | 32.60 | 31.00 | 31.30 | 1.30 |
| 1150 | 1,2,3-Trichloropropane | 47.80 | 46.50 | 46.50 | 1.30 |
| 1151 | Heptyl bromide | 50.20 | 51.70 | 51.50 | -1.30 |
| 1152 | 1-Butoxy-2-propoxyethane | 54.70 | 56.20 | 56.00 | -1.30 |
| 1153 | 2-Chloroiodobenzene | 57.30 | 56.10 | 56.00 | 1.30 |
| 1154 | Methyl propanoate | 35.70 | 37.00 | 37.00 | -1.30 |
| 1155 | t-Amyl acetate | 42.80 | 41.70 | 41.50 | 1.30 |
| 1156 | (Z) 3-Decen-1-ol | 78.70 | 80.10 | 80.00 | -1.30 |
| 1157 | (Z) 3-Tetradecen-1-ol | 99.80 | 98.80 | 98.50 | 1.30 |
| 1158 | (Z) 6-Dodecenal | 69.20 | 70.50 | 70.50 | -1.30 |
| 1159 | (Z) 8-Hexadecenal | 87.70 | 89.20 | 89.00 | -1.30 |
| 1160 | 2-Dodecanone | 71.80 | 70.40 | 70.50 | 1.30 |
| 1161 | 4,4'-Di-t-butylbiphenyl | 86.20 | 87.70 | 87.50 | -1.30 |
| 1162 | 7-Heptadecanol | 108.20 | 109.70 | 109.50 | -1.30 |
| 1163 | Methyl hexadecanoate | 96.20 | 97.70 | 97.50 | -1.30 |
| 1164 | Undecylbenzene | 84.70 | 85.70 | 86.00 | -1.30 |
| 1165 | 4-Methyl-cis-2-pentene | 29.50 | 31.00 | 30.80 | -1.30 |
| 1166 | (E) 3-Decenal | 59.80 | 61.20 | 61.10 | -1.30 |
| 1167 | (E) 7-Decenal | 59.80 | 61.20 | 61.10 | -1.30 |
| 1168 | Glyme | 36.80 | 37.90 | 38.10 | -1.30 |
| 1169 | 2-Chloro-1,1,2-trifluoroethyl isopropyl ether | 39.20 | 38.10 | 37.90 | 1.30 |
| 1170 | Triethylmethanol | 57.30 | 58.60 | 58.60 | -1.30 |
| 1171 | N,N-Dimethyl-3-methyl-3-phenyl-2-butaneamine | 60.30 | 61.70 | 61.60 | -1.30 |
| 1172 | trans-3-Methyl-3-hexene | 35.80 | 37.00 | 37.10 | -1.30 |
| 1173 | 3-Methyl-1-pentene | 28.60 | 29.70 | 29.90 | -1.30 |
| 1174 | s-trans-1,3-Butadiene | 21.10 | 22.70 | 22.40 | -1.30 |
| 1175 | (E) 10-Tetradecenyl acetate | 89.90 | 91.20 | 91.20 | -1.30 |
| 1176 | (Z) 6-Hexadecenal | 87.90 | 89.20 | 89.20 | -1.30 |
| 1177 | 1-Bromobutane | 36.40 | 37.70 | 37.70 | -1.30 |
| 1178 | 1-Chloroheptane | 47.90 | 49.20 | 49.20 | -1.30 |
| 1179 | Butyl pivalate | 50.40 | 51.80 | 51.70 | -1.30 |
| 1180 | cis-4-Methyl-2-heptene | 38.90 | 40.20 | 40.20 | -1.30 |
| 1181 | n-Hexylacetate | 51.90 | 53.10 | 53.20 | -1.30 |
| 1182 | 1-Iodohexane | 49.90 | 51.30 | 51.20 | -1.30 |
| 1183 | thrans-4-Methyl-2-heptene | 38.90 | 40.20 | 40.20 | -1.30 |
| 1184 | trans-4-Ethyl-2-hexene | 38.90 | 40.20 | 40.20 | -1.30 |
| 1185 | trans-5-Methyl-3-heptene | 38.90 | 40.20 | 40.20 | -1.30 |
| 1186 | Undecane | 56.40 | 57.80 | 57.70 | -1.30 |
| 1187 | 1-Bromopropane | 31.90 | 33.00 | 33.20 | -1.30 |
| 1188 | 2-Methylpentane | 29.90 | 31.20 | 31.20 | -1.30 |
| 1189 | Isopropylamine | 28.40 | 29.70 | 29.70 | -1.30 |
| 1190 | 1-Propanethiol | 31.90 | 33.30 | 33.20 | -1.30 |
| 1191 | 2,5-Difluoroaniline | 52.50 | 53.90 | 53.80 | -1.30 |
| 1192 | s-Butylbenzene | 49.50 | 50.70 | 50.80 | -1.30 |
| 1193 | 4-Ethyltoluene | 46.50 | 47.90 | 47.80 | -1.30 |
| 1194 | 1,1-Dimethoxycyclobutane | 42.00 | 43.50 | 43.30 | -1.30 |
| 1195 | Di-n-propylamine | 41.50 | 42.70 | 42.80 | -1.30 |
| 1196 | Linalyl acetate | 62.50 | 63.80 | 63.80 | -1.30 |
| 1197 | (Z) 11-Pentadecenyl acetate | 94.60 | 95.90 | 95.90 | -1.30 |
| 1198 | (Z) 5-Dodecenal | 69.10 | 70.50 | 70.40 | -1.30 |
| 1199 | (Z) 9-Tridecenyl acetate | 85.10 | 86.50 | 86.40 | -1.30 |
| 1200 | 1,2-Dichlorononane | 62.10 | 63.50 | 63.40 | -1.30 |
| 1201 | 1-Cyclohexenecarbonitrile | 53.60 | 55.00 | 54.90 | -1.30 |
| 1202 | 3-Ethyltoluene | 46.60 | 47.90 | 47.90 | -1.30 |
| 1203 | Allyl isothiocyanate | 42.10 | 43.40 |  | -1.30 |
| 1204 | Butyl t-octyl ether | 51.60 | 53.10 | 52.90 | -1.30 |
| 1205 | Undecylcyclopentane | 80.60 | 82.00 | 81.90 | -1.30 |
| 1206 | (E) 3-Decen-1-ol | 78.80 | 80.10 | 80.10 | -1.30 |
| 1207 | (Z) 5-Hexadecenal | 87.80 | 89.20 | 89.10 | -1.30 |
| 1208 | 2-Chlorododecane | 65.30 | 66.40 | 66.60 | -1.30 |
| 1209 | Isovaleronitrile | 41.64 | 40.30 | 40.30 | 1.34 |
| 1210 | Decane | 51.42 | 53.00 | 52.80 | -1.38 |
| 1211 | (E) 6-Dodecen-1-ol | 90.70 | 89.40 | 89.30 | 1.40 |
| 1212 | (E) 8-Decen-1-ol | 81.50 | 80.10 | 80.10 | 1.40 |
| 1213 | 1-Dodecanol | 90.00 | 88.70 | 88.60 | 1.40 |
| 1214 | 1-Iodotetradecane | 90.00 | 88.60 | 88.60 | 1.40 |
| 1215 | 2,3-Dimethoxyphenol | 76.50 | 74.50 | 75.10 | 1.40 |
| 1216 | 3,3,4,4-Tetramethylhexane | 42.30 | 41.10 | 40.90 | 1.40 |
| 1217 | Spiro[4.5]decane | 54.80 | 53.50 | 53.40 | 1.40 |
| 1218 | (E) 7-Dodecen-1-ol | 90.80 | 89.40 | 89.40 | 1.40 |
| 1219 | 1,1-Dichlorohexane | 48.70 | 47.30 | 47.30 | 1.40 |
| 1220 | 2-Nitroisobutane | 42.70 | 41.90 | 41.30 | 1.40 |
| 1221 | Ethyl acetoacetate | 54.20 | 52.70 | 52.80 | 1.40 |
| 1222 | Epoxycycloheptane | 45.20 | 44.10 | 43.80 | 1.40 |
| 1223 | Perfluoro-1-methyl-4-isopropylcyclohexane | 46.70 | 45.30 | 45.30 | 1.40 |
| 1224 | (2,2,2-Trifluoroethyl)benzene | 46.10 | 44.70 | 44.70 | 1.40 |
| 1225 | 1-Iodopentadecane | 94.60 | 93.30 | 93.20 | 1.40 |
| 1226 | 1-Nitro-2-isopropylbenzene | 65.60 | 64.40 | 64.20 | 1.40 |
| 1227 | 2,2,4-Trimethylheptane | 45.60 | 44.30 | 44.20 | 1.40 |
| 1228 | 3-Nonanol | 70.90 | 72.30 | 72.30 | -1.40 |
| 1229 | Methyl 4-hydroxybenzoate | 83.10 | 82.10 | 81.70 | 1.40 |
| 1230 | perfluoro-2-methylbutane | 26.30 | 25.20 | 24.90 | 1.40 |
| 1231 | Pyruvaldehyde | 38.00 | 36.70 | 36.60 | 1.40 |
| 1232 | 4-Chlorotoluene | 46.50 | 45.00 | 45.10 | 1.40 |
| 1233 | 1-Butanethiol | 36.50 | 38.00 | 37.90 | -1.40 |
| 1234 | N-Propylaminotriethylsilane | 41.50 | 40.80 | 40.10 | 1.40 |
| 1235 | 4-Methylbenzaldehyde | 53.50 | 52.30 | 52.10 | 1.40 |
| 1236 | 1,1-Dimethylcyclopropane | 25.10 | 26.50 | 26.50 | -1.40 |
| 1237 | Cyanoethylene | 31.60 | 33.20 | 33.00 | -1.40 |
| 1238 | Cyclopropylacethylene | 30.90 | 29.60 | 29.50 | 1.40 |
| 1239 | Ethyl ether | 27.10 | 28.50 | 28.50 | -1.40 |
| 1240 | Chloroethylene | 20.10 | 21.20 | 21.50 | -1.40 |
| 1241 | (E) 3-Hexadecenyl acetate | 99.10 | 100.50 | 100.50 | -1.40 |
| 1242 | (E) 7-Decenyl acetate | 71.10 | 72.50 | 72.50 | -1.40 |
| 1243 | (E) 9-Tetradecenyl acetate | 89.60 | 91.20 | 91.00 | -1.40 |
| 1244 | 2,3,5-Trimethylhexane | 41.40 | 40.10 | 40.00 | 1.40 |
| 1245 | 2-Methyl-2-hexene | 35.60 | 37.00 | 37.00 | -1.40 |
| 1246 | 4-Ethylheptane | 44.10 | 45.50 | 45.50 | -1.40 |
| 1247 | Dispiro[2.0.2.1]heptane | 35.10 | 36.40 | 36.50 | -1.40 |
| 1248 | Cyclohexyl iodide | 49.60 | 50.70 | 51.00 | -1.40 |
| 1249 | 1-Heptanethiol | 50.60 | 52.00 | 52.00 | -1.40 |
| 1250 | N-Methyl-trimethylsilane | 37.40 | 36.00 | 36.00 | 1.40 |
| 1251 | n-Pentylmalononitrile | 67.10 | 68.70 | 68.50 | -1.40 |
| 1252 | S-Butyl thiolacetate | 48.10 | 49.20 | 49.50 | -1.40 |
| 1253 | 1,3,5-Triethylbenzene | 59.20 | 60.80 | 60.60 | -1.40 |
| 1254 | 2-Ethyl-3-methyl-1-butene | 34.30 | 33.00 | 32.90 | 1.40 |
| 1255 | Benzylmethylsulfide | 55.20 | 56.50 | 56.60 | -1.40 |
| 1256 | Methyl t-butyl sulfide | 34.20 | 35.40 | 35.60 | -1.40 |
| 1257 | (E) 9-Pentadecenyl acetate | 94.30 | 95.90 | 95.70 | -1.40 |
| 1258 | (Z) 7-Hexadecenal | 87.80 | 89.20 | 89.20 | -1.40 |
| 1259 | 2,3,4,4',6-Pentabromodiphenyl ether | 101.80 | 103.20 | 103.20 | -1.40 |
| 1260 | 2-Nitrobenzamide | 98.30 | 98.20 | 99.70 | -1.40 |
| 1261 | Deprenyl | 64.30 | 66.10 | 65.70 | -1.40 |
| 1262 | Tridecanal | 73.30 | 74.50 | 74.70 | -1.40 |
| 1263 | Allyl alcohol | 44.80 | 46.10 | 46.20 | -1.40 |
| 1264 | 2-Chloronaphthalene | 62.30 | 63.90 | 63.70 | -1.40 |
| 1265 | Butylisopropylether | 38.80 | 40.10 | 40.20 | -1.40 |
| 1266 | Ethanethiol | 27.30 | 28.70 | 28.70 | -1.40 |
| 1267 | Ethyl isobutyrate | 39.80 | 41.10 | 41.20 | -1.40 |
| 1268 | 2,2-Dimethylpropane | 21.80 | 23.30 | 23.20 | -1.40 |
| 1269 | Perfluoro-3,6-dioxaheptanoic acid | 62.30 | 61.70 | 63.70 | -1.40 |
| 1270 | Ethyl orthoformate | 47.80 | 48.90 | 49.20 | -1.40 |
| 1271 | (1-Methoxy-1-methylethyl)-benzene | 52.90 | 54.30 | 54.30 | -1.40 |
| 1272 | cis-2,2,4,6-Tetramethyl-1,3-dioxane | 41.90 | 43.00 | 43.30 | -1.40 |
| 1273 | cis-2-Methyl-3-heptene | 38.90 | 40.20 | 40.30 | -1.40 |
| 1274 | (E) 8-Dodecenyl acetate | 80.50 | 81.90 | 81.90 | -1.40 |
| 1275 | (Z) 6-Tetradecenal | 78.50 | 79.90 | 79.90 | -1.40 |
| 1276 | 1-Ethylcyclopentene | 38.50 | 40.20 | 39.90 | -1.40 |
| 1277 | 3-Iodoaniline | 67.50 | 69.10 | 68.90 | -1.40 |
| 1278 | Diethylcarbonate | 50.00 | 51.30 | 51.40 | -1.40 |
| 1279 | n-Octylamine | 55.50 | 57.00 | 56.90 | -1.40 |
| 1280 | Pulegone | 58.00 | 59.50 | 59.40 | -1.40 |
| 1281 | Trimethylisoxazole | 50.00 | 51.30 | 51.40 | -1.40 |
| 1282 | (Z) 11-Hexadecenyl acetate | 98.90 | 100.50 | 100.30 | -1.40 |
| 1283 | (Z) 5-Tetradecenal | 78.40 | 79.90 | 79.80 | -1.40 |
| 1284 | Tetradecylcyclohexane | 99.40 | 101.10 | 100.80 | -1.40 |
| 1285 | Vinylidenechloride | 26.74 | 28.30 | 28.20 | -1.46 |
| 1286 | (Z) 8-Tetradecen-1-ol | 100.30 | 98.80 | 98.80 | 1.50 |
| 1287 | 1-Iodohexadecane | 99.60 | 98.00 | 98.10 | 1.50 |
| 1288 | 1-Pentadecene | 75.10 | 73.90 | 73.60 | 1.50 |
| 1289 | 1-Phenylnaphthalene | 88.60 | 87.10 | 87.10 | 1.50 |
| 1290 | 2-Acetylthiophene | 55.40 | 53.50 | 53.90 | 1.50 |
| 1291 | 2,3-Dibromobutane | 40.90 | 39.00 | 39.40 | 1.50 |
| 1292 | 2-Chlorotoluene | 46.40 | 45.00 | 44.90 | 1.50 |
| 1293 | 2-Methyl-1-pentanol | 59.40 | 58.00 | 57.90 | 1.50 |
| 1294 | 3-Bromoaniline | 63.40 | 62.10 | 61.90 | 1.50 |
| 1295 | Ethoxymethyl propionate | 49.90 | 48.70 | 48.40 | 1.50 |
| 1296 | Ethyldibutylsilane | 39.90 | 38.30 | 38.40 | 1.50 |
| 1297 | Phenylformate | 52.90 | 51.60 | 51.40 | 1.50 |
| 1298 | 3-Bromoiodobenzene | 60.80 | 59.10 | 59.30 | 1.50 |
| 1299 | 2-Isopropyl-1-pentene | 38.80 | 37.60 | 37.30 | 1.50 |
| 1300 | 4-Methyl-2-pentanol | 57.30 | 55.60 | 55.80 | 1.50 |
| 1301 | Dimethyl disulfide | 37.80 | 36.80 | 36.30 | 1.50 |
| 1302 | Oxocan-2-one | 53.30 | 51.70 | 51.80 | 1.50 |
| 1303 | Methyl t-pentyl ether | 35.30 | 33.60 | 33.80 | 1.50 |
| 1304 | (E) 5-Dodecen-1-ol | 90.70 | 89.40 | 89.20 | 1.50 |
| 1305 | alpha-Ethylbenzylcyanide | 64.30 | 65.80 | 65.80 | -1.50 |
| 1306 | Nonanedioic acid | 119.70 | 117.70 | 118.20 | 1.50 |
| 1307 | 1-(1-Adamantyl)ethylamine | 68.70 | 67.20 | 67.20 | 1.50 |
| 1308 | Undecanedioic acid | 128.20 | 127.00 | 126.70 | 1.50 |
| 1309 | 1,1-Dichlorooctane | 57.70 | 56.70 | 56.20 | 1.50 |
| 1310 | Chlorooxoacetic acid propyl ester | 52.70 | 51.50 | 51.20 | 1.50 |
| 1311 | Methyl dichloroacetate | 47.70 | 46.20 | 46.20 | 1.50 |
| 1312 | 1,4-Dibromobutane | 52.60 | 51.30 | 51.10 | 1.50 |
| 1313 | 2-Chloroacetophenone | 60.10 | 58.50 | 58.60 | 1.50 |
| 1314 | Isobutyronitrile | 37.10 | 35.70 | 35.60 | 1.50 |
| 1315 | Diethyl succinate | 65.10 | 63.50 | 63.60 | 1.50 |
| 1316 | Isobutyl t-butylether | 39.10 | 37.60 | 37.60 | 1.50 |
| 1317 | Tetramethylurea | 56.60 | 55.10 |  | 1.50 |
| 1318 | (E) 8-Dodecen-1-ol | 91.00 | 89.40 | 89.50 | 1.50 |
| 1319 | 1,1-Dimethylpropyl 2,2-dimethylpropanoate | 48.00 | 49.70 | 49.50 | -1.50 |
| 1320 | 1-Chloropropane | 29.00 | 30.50 | 30.50 | -1.50 |
| 1321 | 2,4-Dimethyl-1,3-dioxole | 35.00 | 36.30 | 36.50 | -1.50 |
| 1322 | 2,5-Dimethyloctane | 49.00 | 47.50 | 47.50 | 1.50 |
| 1323 | 2-Hexanol | 57.00 | 58.30 | 58.50 | -1.50 |
| 1324 | Allyl iodide | 38.00 | 36.70 | 36.50 | 1.50 |
| 1325 | 4,5-Dimethyl-1,3-dioxane | 42.00 | 40.80 | 40.50 | 1.50 |
| 1326 | 4,5-Dimethyl-2-sulfanyl-1,3,2-dioxaphospholane 2-sulfide | 66.00 | 64.50 |  | 1.50 |
| 1327 | Bicyclo[3.1.0]hexane | 33.50 | 34.90 | 35.00 | -1.50 |
| 1328 | cis-Bicyclo[3.3.0]octan-2-one | 54.00 | 52.60 | 52.50 | 1.50 |
| 1329 | Diethylene glycol monohexyl ether | 86.50 | 88.10 | 88.00 | -1.50 |
| 1330 | 1,2,3,5-Tetramethylbenzene | 52.00 | 50.60 | 50.50 | 1.50 |
| 1331 | Methyl 2-methylbutyrate | 42.00 | 43.60 | 43.50 | -1.50 |
| 1332 | Methyl 4-fluorobenzoate | 56.00 | 57.50 | 57.50 | -1.50 |
| 1333 | Propyl 2-dimethylamino-2-methylpropanoate | 54.00 | 55.60 | 55.50 | -1.50 |
| 1334 | Methylbenzene | 38.00 | 39.70 | 39.50 | -1.50 |
| 1335 | Tridecylcyclohexane | 94.50 | 96.30 | 96.00 | -1.50 |
| 1336 | Isopentane | 24.80 | 26.50 | 26.30 | -1.50 |
| 1337 | Isobutylene | 20.60 | 21.90 | 22.10 | -1.50 |
| 1338 | Dimethylacetal | 36.40 | 34.80 | 34.90 | 1.50 |
| 1339 | 1,3-Difluorobenzene | 34.60 | 36.10 | 36.10 | -1.50 |
| 1340 | 2,4-Difluoroaniline | 52.10 | 53.90 | 53.60 | -1.50 |
| 1341 | 2,5-Dimethyl-1-hexene | 38.90 | 37.60 | 37.40 | 1.50 |
| 1342 | 3-Ethyl-2-pentene | 35.60 | 37.00 | 37.10 | -1.50 |
| 1343 | 2-Ethyl-3-methyl-1-pentene | 38.90 | 37.60 | 37.40 | 1.50 |
| 1344 | cis-3-Methyl-2-hexene | 35.60 | 37.00 | 37.10 | -1.50 |
| 1345 | 1,2-Diaminoethane | 41.60 | 43.20 | 43.10 | -1.50 |
| 1346 | Ethylidenecyclopentane | 38.60 | 40.20 | 40.10 | -1.50 |
| 1347 | Quinazoline | 59.60 | 61.30 | 61.10 | -1.50 |
| 1348 | Trispiro[2.0.2.0.2.0]nonane | 42.10 | 43.20 | 43.60 | -1.50 |
| 1349 | Caprylonitrile | 55.70 | 57.10 | 57.20 | -1.50 |
| 1350 | 2,5,8,11-Tetraoxododecane | 63.70 | 65.30 | 65.20 | -1.50 |
| 1351 | 2-Methylbenzophenone | 81.20 | 82.90 | 82.70 | -1.50 |
| 1352 | Decylcyclopentane | 75.70 | 77.30 | 77.20 | -1.50 |
| 1353 | Di-t-butyl glutarate | 71.70 | 73.30 | 73.20 | -1.50 |
| 1354 | (Z) 7-Dodecenyl acetate | 80.20 | 81.90 | 81.70 | -1.50 |
| 1355 | n-Decylacetate | 70.20 | 71.80 | 71.70 | -1.50 |
| 1356 | 1,4-Bis(trifluoromethyl)benzene | 41.80 | 43.70 | 43.30 | -1.50 |
| 1357 | Cyclopropylamine | 31.30 | 32.90 | 32.80 | -1.50 |
| 1358 | (E) 4-Hexadecenyl acetate | 98.90 | 100.50 | 100.40 | -1.50 |
| 1359 | (E) 9-Hexadecenyl acetate | 98.90 | 100.50 | 100.40 | -1.50 |
| 1360 | (Z) 11-Octadecenyl acetate | 108.40 | 109.90 | 109.90 | -1.50 |
| 1361 | 5-Nonanone | 54.90 | 56.40 | 56.40 | -1.50 |
| 1362 | Dibutyl oxalate | 71.40 | 72.80 | 72.90 | -1.50 |
| 1363 | Methylacetamide | 59.40 | 60.70 | 60.90 | -1.50 |
| 1364 | (E) 10-Hexadecenyl acetate | 99.10 | 100.50 | 100.60 | -1.50 |
| 1365 | (Z) 10-Tetradecenyl acetate | 89.60 | 91.20 | 91.10 | -1.50 |
| 1366 | Dodecyl acetate | 79.60 | 81.20 | 81.10 | -1.50 |
| 1367 | 1,1-Dimethoxy-2-butene | 43.26 | 44.80 | 44.80 | -1.54 |
| 1368 | n-Decyl alcohol | 80.90 | 79.40 | 79.30 | 1.60 |
| 1369 | O,O-Diethyl phosphorodithioate | 67.70 | 66.10 |  | 1.60 |
| 1370 | 1,1-Dichloroheptane | 53.50 | 52.00 | 51.90 | 1.60 |
| 1371 | 2-Methyl-2-pentyl-1,3-dioxolane | 54.00 | 52.40 | 52.40 | 1.60 |
| 1372 | 3,3-Dimethyloctane | 48.50 | 47.10 | 46.90 | 1.60 |
| 1373 | Methyl isobutyl ketone | 41.00 | 39.60 | 39.40 | 1.60 |
| 1374 | Cyclobutanol | 54.00 | 52.20 | 52.40 | 1.60 |
| 1375 | Methyl 2-octynoate | 64.50 | 63.10 | 62.90 | 1.60 |
| 1376 | 2,5-Dimethyl-3-ethylhexane | 46.40 | 44.80 | 44.80 | 1.60 |
| 1377 | 2-Chloronitrobenzene | 60.40 | 58.90 | 58.80 | 1.60 |
| 1378 | 3-Isopropyl-2-methylhexane | 46.40 | 44.80 | 44.80 | 1.60 |
| 1379 | 2,2’-Diaminodiethylamine | 63.40 | 61.90 | 61.80 | 1.60 |
| 1380 | 2,4,5-Trimethylacetophenone | 63.20 | 64.90 | 64.80 | -1.60 |
| 1381 | 2-(Diethylamino)pentanenitrile | 58.80 | 56.70 | 57.20 | 1.60 |
| 1382 | 3,3-Dimethyl-2-butanol | 53.80 | 52.30 | 52.20 | 1.60 |
| 1383 | 4-Ethyl-m-xylene | 53.30 | 51.60 | 51.70 | 1.60 |
| 1384 | Diethylmethylamine | 31.80 | 29.90 | 30.20 | 1.60 |
| 1385 | Ethyl t-octyl ether | 45.30 | 43.80 | 43.70 | 1.60 |
| 1386 | Isopropylthioacetate | 42.30 | 41.00 | 40.70 | 1.60 |
| 1387 | N,N-Dimethylaniline | 52.80 | 52.00 | 51.20 | 1.60 |
| 1388 | (Z) 5-Tetradecen-1-ol | 100.30 | 98.80 | 98.70 | 1.60 |
| 1389 | (Z) 9-Tridecen-1-ol | 95.80 | 94.10 | 94.20 | 1.60 |
| 1390 | Cetene | 80.30 | 78.50 | 78.70 | 1.60 |
| 1391 | Heneicosane | 106.80 | 105.40 | 105.20 | 1.60 |
| 1392 | Indole | 65.30 | 66.70 | 63.70 | 1.60 |
| 1393 | Propylacetamide | 69.80 | 67.80 | 68.20 | 1.60 |
| 1394 | 3,4-Dichloronitrobenzene | 65.20 | 63.70 | 63.60 | 1.60 |
| 1395 | 1,2,4,5-Tetrafluorobenzene | 37.20 | 35.70 | 35.60 | 1.60 |
| 1396 | 2,2-Dichlorotrifluoroethyl 1,1,3-trichlorotetrafluoropropyl ether | 50.70 | 49.80 | 49.10 | 1.60 |
| 1397 | 3,6,9-Triethyl-3,6,9-trimethyl-1,2,4,5,7,8-hexaoxacyclononane | 59.20 | 58.00 | 57.60 | 1.60 |
| 1398 | Cyclohexyl azide | 49.20 | 47.80 | 47.60 | 1.60 |
| 1399 | 1,5-Diaminopentane | 58.70 | 57.20 | 57.10 | 1.60 |
| 1400 | Diisopropyl oxalate | 60.20 | 58.70 | 58.60 | 1.60 |
| 1401 | 3-Methylbenzaldehyde | 53.70 | 52.30 | 52.10 | 1.60 |
| 1402 | n-Butylethylether | 36.30 | 37.80 | 37.90 | -1.60 |
| 1403 | (E) 7-Tridecen-1-ol | 95.60 | 94.10 | 94.00 | 1.60 |
| 1404 | (Z) 8-Decen-1-ol | 81.60 | 80.10 | 80.00 | 1.60 |
| 1405 | 1,1-Dimethylbutyl 2-methylpropanoate | 51.40 | 53.00 | 53.00 | -1.60 |
| 1406 | 4-Methylcyclohexyl propanoate | 58.90 | 60.50 | 60.50 | -1.60 |
| 1407 | 4-Phenylpyridine | 68.40 | 70.00 | 70.00 | -1.60 |
| 1408 | cis-1-Methyl-1,2-dicyclopropylcyclopropane | 46.90 | 48.50 | 48.50 | -1.60 |
| 1409 | Dicyclopropylacetylene | 47.60 | 45.90 | 46.00 | 1.60 |
| 1410 | Diphenylamine | 79.90 | 81.50 | 81.50 | -1.60 |
| 1411 | Ethyldiphenylsilane | 66.10 | 65.20 | 64.50 | 1.60 |
| 1412 | n-Valeraldehyde | 38.60 | 37.10 | 37.00 | 1.60 |
| 1413 | Tetradecanal | 77.40 | 79.20 | 79.00 | -1.60 |
| 1414 | (E) 6-Decenal | 59.50 | 61.20 | 61.10 | -1.60 |
| 1415 | 1,2-Difluoro-1,1,2-trichloroethane | 33.00 | 34.60 | 34.60 | -1.60 |
| 1416 | 2-Methyl-1,3-dioxane | 39.00 | 37.60 | 37.40 | 1.60 |
| 1417 | 2-Methylpiperidine | 40.50 | 42.00 | 42.10 | -1.60 |
| 1418 | Ethyl hexanoate | 51.50 | 53.10 | 53.10 | -1.60 |
| 1419 | Nonane | 46.50 | 48.20 | 48.10 | -1.60 |
| 1420 | 2-Methoxy-1-propene | 28.30 | 29.80 | 29.90 | -1.60 |
| 1421 | (E) 11-Tetradecenyl acetate | 89.60 | 91.20 | 91.20 | -1.60 |
| 1422 | Pentadecanonitrile | 88.10 | 89.80 | 89.70 | -1.60 |
| 1423 | Phenazine | 76.60 | 77.90 | 78.20 | -1.60 |
| 1424 | Trimethoxy((2-propenylthio)methyl)silane | 38.60 | 41.00 | 40.20 | -1.60 |
| 1425 | 1-Nonene | 44.70 | 46.30 | 46.30 | -1.60 |
| 1426 | 2,3-Dihydrothiophene | 37.70 | 39.30 | 39.30 | -1.60 |
| 1427 | Cumylamine | 56.70 | 58.10 | 58.30 | -1.60 |
| 1428 | cis-3-Methyl-2-heptene | 39.70 | 41.60 | 41.30 | -1.60 |
| 1429 | cis-Bicyclo[3.3.0]octane | 42.70 | 44.40 | 44.30 | -1.60 |
| 1430 | Isobutyl 3-chloropropionate | 55.20 | 56.80 | 56.80 | -1.60 |
| 1431 | trans-3-Methyl-3-heptene | 39.70 | 41.60 | 41.30 | -1.60 |
| 1432 | trans-4-Methyl-3-heptene | 39.70 | 41.60 | 41.30 | -1.60 |
| 1433 | trans-Bicyclo[3.3.0]octane | 42.70 | 44.40 | 44.30 | -1.60 |
| 1434 | (E) 7-Tridecenyl acetate | 84.80 | 86.50 | 86.40 | -1.60 |
| 1435 | 1-Butyl-2-methylimidazole | 67.80 | 68.80 | 69.40 | -1.60 |
| 1436 | Phorate | 70.80 | 72.40 |  | -1.60 |
| 1437 | 1,2-Dihydronaphthalene | 54.80 | 56.40 | 56.40 | -1.60 |
| 1438 | 2,5-Dioxanonane | 47.80 | 49.40 | 49.40 | -1.60 |
| 1439 | Indene | 50.30 | 51.80 | 51.90 | -1.60 |
| 1440 | Methyl 4-methylbenzoate | 59.80 | 61.40 | 61.40 | -1.60 |
| 1441 | Methyl isobutanoate | 37.30 | 38.90 | 38.90 | -1.60 |
| 1442 | 1-Methyl-2-piperidone | 60.30 | 63.10 | 61.90 | -1.60 |
| 1443 | Caryophyllene | 65.50 | 66.90 | 67.10 | -1.60 |
| 1444 | (E) 7-Dodecenyl acetate | 80.20 | 81.90 | 81.80 | -1.60 |
| 1445 | Methyl (Z) 9-octadecenoate | 106.20 | 107.70 | 107.80 | -1.60 |
| 1446 | 1-Bromodecane | 67.12 | 65.70 | 65.50 | 1.62 |
| 1447 | (E) 6-Tetradecen-1-ol | 100.50 | 98.80 | 98.80 | 1.70 |
| 1448 | N-Methylcarbazole | 79.50 | 77.90 | 77.80 | 1.70 |
| 1449 | 2,5-Dichlorobiphenyl | 76.80 | 75.20 | 75.10 | 1.70 |
| 1450 | 2-Ethyl-1-hexanoic acid | 76.30 | 75.20 | 74.60 | 1.70 |
| 1451 | Di-t-butyl malonate | 65.80 | 63.90 | 64.10 | 1.70 |
| 1452 | Diethyl glutarate | 69.80 | 68.20 | 68.10 | 1.70 |
| 1453 | (Z) 9-Dodecen-1-ol | 91.10 | 89.40 | 89.40 | 1.70 |
| 1454 | 2,2-Diacetoxypropane | 53.60 | 51.90 | 51.90 | 1.70 |
| 1455 | 2-Chlorobiphenyl | 72.10 | 70.40 | 70.40 | 1.70 |
| 1456 | 2-Norbornanone | 49.60 | 48.00 | 47.90 | 1.70 |
| 1457 | Propyl t-octyl ether | 50.10 | 48.40 | 48.40 | 1.70 |
| 1458 | 2,2,5-Trimethylheptane | 46.00 | 44.30 | 44.30 | 1.70 |
| 1459 | 2,4-Lutidine | 47.50 | 45.90 | 45.80 | 1.70 |
| 1460 | 2-Chloro-1,1,2-trifluoroethyl ethyl ether | 37.50 | 35.90 | 35.80 | 1.70 |
| 1461 | 2-Thiopheneacetonitrile | 60.50 | 58.90 | 58.80 | 1.70 |
| 1462 | 3-Methyl-2-thiapentane | 38.50 | 37.00 | 36.80 | 1.70 |
| 1463 | Pyridazine | 53.50 | 52.10 | 51.80 | 1.70 |
| 1464 | trans-2,2,5,5-Tetramethyl-3-hexene | 42.00 | 40.20 | 40.30 | 1.70 |
| 1465 | 1,5-Diamino-2-methylpentane | 60.90 | 59.20 | 59.20 | 1.70 |
| 1466 | 2,4,6-Trimethylheptane | 46.40 | 44.80 | 44.70 | 1.70 |
| 1467 | 2-Methoxybenzamide | 89.40 | 87.30 | 87.70 | 1.70 |
| 1468 | 2-Methyl-1-penten-4-one | 41.90 | 40.50 | 40.20 | 1.70 |
| 1469 | 4-t-Butylpyridine | 54.40 | 52.70 | 52.70 | 1.70 |
| 1470 | Diphenylmethane | 67.60 | 69.30 | 69.30 | -1.70 |
| 1471 | Fluorene | 72.40 | 70.60 | 70.70 | 1.70 |
| 1472 | Ethylacetamide | 64.90 | 63.20 | 63.20 | 1.70 |
| 1473 | Tetrakis(3-methylphenoxy)silane | 73.60 | 72.90 | 75.30 | -1.70 |
| 1474 | 1,1-Dichloropentane | 44.30 | 42.60 | 42.60 | 1.70 |
| 1475 | 2,6-Lutidine | 45.30 | 43.40 | 43.60 | 1.70 |
| 1476 | Aniline | 55.80 | 54.30 | 54.10 | 1.70 |
| 1477 | Methylphosphonic acid dimethyl ester | 52.80 | 51.10 |  | 1.70 |
| 1478 | Ethyl 3-methyl-2-butenoate | 49.30 | 47.80 | 47.60 | 1.70 |
| 1479 | trans-2-Hexene | 32.20 | 33.70 | 33.90 | -1.70 |
| 1480 | 1-Fluoro-2-methylbutane | 30.70 | 29.20 | 29.00 | 1.70 |
| 1481 | Diisobutylene | 35.70 | 34.40 | 34.00 | 1.70 |
| 1482 | 3,3,4,4-Tetramethyl-1,2-dioxetane | 29.70 | 27.80 | 28.00 | 1.70 |
| 1483 | Diethylaminoacetone | 47.70 | 45.90 | 46.00 | 1.70 |
| 1484 | N,N-Dibutylaminotriethylsilane | 56.30 | 57.10 | 58.00 | -1.70 |
| 1485 | 1-Iodobutane | 40.30 | 41.90 | 42.00 | -1.70 |
| 1486 | Benzaldehyde diethylacetal | 62.80 | 64.50 | 64.50 | -1.70 |
| 1487 | Tetrahydropyran | 38.20 | 36.30 | 36.50 | 1.70 |
| 1488 | trans-1-Methyl-1,2-dicyclopropylcyclopropane | 47.30 | 48.50 | 49.00 | -1.70 |
| 1489 | 1,2-Butanediamine | 46.90 | 48.60 | 48.60 | -1.70 |
| 1490 | 1-Bromopentane | 40.90 | 42.40 | 42.60 | -1.70 |
| 1491 | 1-Chlorooctane | 52.40 | 53.80 | 54.10 | -1.70 |
| 1492 | Pentyl t-octyl ether | 55.90 | 57.80 | 57.60 | -1.70 |
| 1493 | 2,3',4,4'-Tetrabromodiphenyl ether | 93.50 | 95.30 | 95.20 | -1.70 |
| 1494 | 2-Fluoroaniline | 52.00 | 54.10 | 53.70 | -1.70 |
| 1495 | 4-Propylheptane | 48.50 | 50.30 | 50.20 | -1.70 |
| 1496 | Isopentyl 2-butenoate | 54.00 | 55.80 | 55.70 | -1.70 |
| 1497 | 1-Chlorobutane | 33.50 | 35.10 | 35.20 | -1.70 |
| 1498 | n-Hexylformate | 50.00 | 51.70 | 51.70 | -1.70 |
| 1499 | 3-Hexanone | 40.60 | 42.30 | 42.30 | -1.70 |
| 1500 | 4-Methylcyclohexyl acetate | 54.10 | 55.90 | 55.80 | -1.70 |
| 1501 | 1,1-Dimethylazoethane | 39.10 | 40.50 | 40.80 | -1.70 |
| 1502 | 1-Pentanethiol | 41.10 | 42.70 | 42.80 | -1.70 |
| 1503 | Perfluoromethylcyclohexane | 36.60 | 38.30 | 38.30 | -1.70 |
| 1504 | trans-3-Hexen-1-ol | 59.60 | 61.40 | 61.30 | -1.70 |
| 1505 | Bis(piperidino)methane | 62.20 | 63.90 |  | -1.70 |
| 1506 | Cumyl ethyl ether | 54.70 | 56.40 | 56.40 | -1.70 |
| 1507 | Acenaphthene | 67.20 | 68.90 | 68.90 | -1.70 |
| 1508 | N-Methylnicotinamide | 88.20 | 90.60 | 89.90 | -1.70 |
| 1509 | Nonylcyclopentane | 70.70 | 72.50 | 72.40 | -1.70 |
| 1510 | 2,4'-Bipyridine | 70.90 | 72.10 | 72.60 | -1.70 |
| 1511 | 2,5,8,11,14-Pentaoxopentadecane | 76.90 | 79.10 | 78.60 | -1.70 |
| 1512 | (E) 6-Hexadecenyl acetate | 98.60 | 100.50 | 100.30 | -1.70 |
| 1513 | o-Cumenol | 73.01 | 71.20 | 71.30 | 1.71 |
| 1514 | Piperidine | 39.25 | 41.30 | 41.00 | -1.75 |
| 1515 | 2,7-Diiodofluorene | 103.10 | 101.20 | 101.30 | 1.80 |
| 1516 | 1-Heptadecene | 84.90 | 83.10 | 83.10 | 1.80 |
| 1517 | 3,5,5-Trimethyl-1-hexanol | 67.90 | 66.00 | 66.10 | 1.80 |
| 1518 | 3-Isopropylbiphenyl | 76.40 | 74.90 | 74.60 | 1.80 |
| 1519 | Dimethyl glutarate | 65.70 | 63.90 | 63.90 | 1.80 |
| 1520 | O,O-Diethyl O-2-dimethylaminoethyl thiophosphate | 72.70 | 70.90 | 70.90 | 1.80 |
| 1521 | 2-Azetidinone | 63.20 | 61.70 | 61.40 | 1.80 |
| 1522 | 3-Methyl-3-pentanol | 55.70 | 53.90 | 53.90 | 1.80 |
| 1523 | Ethyl thiophene-2-carboxylate | 61.20 | 59.60 | 59.40 | 1.80 |
| 1524 | 2,3,4,5,6-Pentafluorotoluene | 41.20 | 39.20 | 39.40 | 1.80 |
| 1525 | 2-Picoline | 43.60 | 42.20 | 41.80 | 1.80 |
| 1526 | Methyldi(2,2-dimethylpropyl)silane | 38.10 | 36.60 | 36.30 | 1.80 |
| 1527 | (E) 2-Dodecen-1-ol | 91.00 | 89.40 | 89.20 | 1.80 |
| 1528 | (Z) 8-Dodecen-1-ol | 91.00 | 89.40 | 89.20 | 1.80 |
| 1529 | 1-trans-5-trans-9-cis-Cyclododecatriene | 68.00 | 66.00 | 66.20 | 1.80 |
| 1530 | 2,5-Dimethylpyrrole | 56.50 | 53.10 | 54.70 | 1.80 |
| 1531 | 3,3,5-Trimethylheptane | 46.00 | 44.30 | 44.20 | 1.80 |
| 1532 | 3,4-Difluoronitrobenzene | 55.50 | 53.70 | 53.70 | 1.80 |
| 1533 | Dimethyl succinate | 61.00 | 59.20 | 59.20 | 1.80 |
| 1534 | Dipropyl succinate | 71.00 | 72.80 | 72.80 | -1.80 |
| 1535 | Isopropyl vinyl ether | 31.00 | 28.90 | 29.20 | 1.80 |
| 1536 | 1-Hexene | 30.60 | 32.50 | 32.40 | -1.80 |
| 1537 | Acetophenone | 55.40 | 53.80 | 53.60 | 1.80 |
| 1538 | Diisopropyldiazene | 35.90 | 34.50 | 34.10 | 1.80 |
| 1539 | trans-1,2-Dimethylcyclopentane | 34.60 | 36.50 | 36.40 | -1.80 |
| 1540 | 1-Chloro-4-fluorobenzene | 42.80 | 41.00 | 41.00 | 1.80 |
| 1541 | Neopentyl alcohol | 51.80 | 50.10 | 50.00 | 1.80 |
| 1542 | 2,6-Dimethyloctane | 49.30 | 47.50 | 47.50 | 1.80 |
| 1543 | Benzedrine | 58.20 | 59.80 | 60.00 | -1.80 |
| 1544 | cis-3-Methyl-3-heptene | 39.70 | 41.60 | 41.50 | -1.80 |
| 1545 | Cyclononane | 49.70 | 51.40 | 51.50 | -1.80 |
| 1546 | Cyclopentylamine | 40.20 | 42.30 | 42.00 | -1.80 |
| 1547 | 2-Butanone | 34.80 | 33.00 | 33.00 | 1.80 |
| 1548 | 3-Methyl-2-butanone | 36.80 | 35.00 | 35.00 | 1.80 |
| 1549 | 2-Chloro-6-(trichloromethyl)pyridine | 58.70 | 60.50 |  | -1.80 |
| 1550 | (Z) 9-Pentadecen-1-ol | 105.30 | 103.40 | 103.50 | 1.80 |
| 1551 | 2,6-Dimethyl-4-heptanol | 65.20 | 66.80 | 67.00 | -1.80 |
| 1552 | Dicyclohexano-18-crown-6 | 124.20 | 125.10 | 126.00 | -1.80 |
| 1553 | (Z) 9-Octadecen-1-ol | 119.30 | 117.50 | 117.50 | 1.80 |
| 1554 | (Z) 6-Decenal | 59.30 | 61.20 | 61.10 | -1.80 |
| 1555 | Cyclohexylacetate | 52.30 | 53.90 | 54.10 | -1.80 |
| 1556 | 2,2-Dichloro-1,1,1-trifluoroethane | 26.60 | 28.50 | 28.40 | -1.80 |
| 1557 | 1,1,3-Trimethylbutyl 2-methylpropanoate | 53.40 | 54.90 | 55.20 | -1.80 |
| 1558 | 1-Ethyl-1-methylcyclopentane | 38.90 | 40.70 | 40.70 | -1.80 |
| 1559 | 1-Heptanol | 67.10 | 65.40 | 65.30 | 1.80 |
| 1560 | 3-Fluorobenzotrifluoride | 37.90 | 39.90 | 39.70 | -1.80 |
| 1561 | 9,10-Dihydrophenanthrene | 76.60 | 75.20 | 74.80 | 1.80 |
| 1562 | Di-n-pentyl malonate | 84.90 | 86.80 | 86.70 | -1.80 |
| 1563 | Ethylbenzene | 42.40 | 44.30 | 44.20 | -1.80 |
| 1564 | 2-Methylpropylamine | 33.90 | 35.60 | 35.70 | -1.80 |
| 1565 | Freon 113 | 28.40 | 28.70 | 30.20 | -1.80 |
| 1566 | 1,2,2-Trimethylcyclopropene | 27.90 | 29.60 | 29.70 | -1.80 |
| 1567 | Ethyl-n-propylether | 31.40 | 33.10 | 33.20 | -1.80 |
| 1568 | 3,5-Dimethylisoxazole | 45.00 | 48.00 | 46.80 | -1.80 |
| 1569 | 5-Methyl-2,3-dihydrofuran | 35.00 | 36.40 | 36.80 | -1.80 |
| 1570 | Fluorobenzene | 34.50 | 36.30 | 36.30 | -1.80 |
| 1571 | 1-Methyl-1H-pyrazole | 48.00 | 49.80 |  | -1.80 |
| 1572 | (Z) 10-Pentadecenyl acetate | 94.10 | 95.90 | 95.90 | -1.80 |
| 1573 | 1,3,5-Triisopropylbenzene | 64.60 | 66.40 | 66.40 | -1.80 |
| 1574 | 2,6-Xylidine | 59.60 | 61.70 | 61.40 | -1.80 |
| 1575 | Diphenylmethylsilane | 64.60 | 66.00 | 66.40 | -1.80 |
| 1576 | (E) 5-Hexadecenyl acetate | 98.80 | 100.50 | 100.60 | -1.80 |
| 1577 | Methyl heptadecanoate | 100.80 | 102.30 | 102.60 | -1.80 |
| 1578 | Octylcyclopentane | 65.80 | 67.80 | 67.60 | -1.80 |
| 1579 | (E) 5-Dodecenyl acetate | 80.00 | 81.90 | 81.80 | -1.80 |
| 1580 | (E) 6-Dodecenyl acetate | 80.00 | 81.90 | 81.80 | -1.80 |
| 1581 | (Z) 3-Hexadecenyl acetate | 98.50 | 100.50 | 100.30 | -1.80 |
| 1582 | 3-Methylenecyclobutanenitrile | 44.56 | 46.30 | 46.40 | -1.84 |
| 1583 | 1-Methylnorcamphor | 47.61 | 49.40 | 49.50 | -1.89 |
| 1584 | (E) 7-Tetradecen-1-ol | 100.50 | 98.80 | 98.60 | 1.90 |
| 1585 | 1-(4-Bromophenyl)-1H-imidazole | 83.50 | 81.10 | 81.60 | 1.90 |
| 1586 | 1-Iodoeicosane | 118.50 | 116.60 | 116.60 | 1.90 |
| 1587 | 1-Methoxynaphthalene | 68.00 | 65.90 | 66.10 | 1.90 |
| 1588 | 1-Naphthalenecarboxylic acid | 99.50 | 97.90 | 97.60 | 1.90 |
| 1589 | 3-Chlorotoluene | 46.80 | 45.00 | 44.90 | 1.90 |
| 1590 | Methyl trichloroacetate | 48.30 | 46.70 | 46.40 | 1.90 |
| 1591 | Triethoxyphenylsilane | 58.30 | 56.50 | 56.40 | 1.90 |
| 1592 | (E) 5-Tetradecen-1-ol | 100.80 | 98.80 | 98.90 | 1.90 |
| 1593 | 1,2-Dibromopropane | 42.20 | 40.50 | 40.30 | 1.90 |
| 1594 | 2,2,3-Trimethylhexane | 41.70 | 39.60 | 39.80 | 1.90 |
| 1595 | N-Ethylmorpholine | 42.20 | 39.90 | 40.30 | 1.90 |
| 1596 | 1,1-Dichloropropane | 35.20 | 33.30 | 33.30 | 1.90 |
| 1597 | (Z) 9-Tetradecen-1-ol | 100.60 | 98.80 | 98.70 | 1.90 |
| 1598 | 1,3-Dimethyl-5-t-butylbenzene | 56.60 | 54.80 | 54.70 | 1.90 |
| 1599 | 1-Chloroundecane | 65.90 | 67.80 | 67.80 | -1.90 |
| 1600 | 2,6-Dichlorotoluene | 51.60 | 49.70 | 49.70 | 1.90 |
| 1601 | 3,5-Dimethyl-1-hexene | 38.10 | 36.20 | 36.20 | 1.90 |
| 1602 | 3,8,8-Trimethyl-4-oxatricyclo[5.1.0.03,5]octane | 50.10 | 48.40 | 48.20 | 1.90 |
| 1603 | 1,1-Dichloroethane | 30.60 | 28.60 | 28.70 | 1.90 |
| 1604 | 1,1,3-Trimethylcyclopentane | 36.00 | 38.00 | 37.90 | -1.90 |
| 1605 | 1,2-Dibromotetrafluoroethane | 28.50 | 26.60 |  | 1.90 |
| 1606 | 1-Chloro-3-fluorobenzene | 43.00 | 41.00 | 41.10 | 1.90 |
| 1607 | 2,2-Dimethyl-3-ethylhexane | 46.00 | 44.30 | 44.10 | 1.90 |
| 1608 | 2,5,5-Trimethylheptane | 46.00 | 44.30 | 44.10 | 1.90 |
| 1609 | Cyclobutylamine | 36.00 | 37.60 | 37.90 | -1.90 |
| 1610 | Tris(pentafluoroethyl)amine | 34.00 | 32.70 | 32.10 | 1.90 |
| 1611 | trans-1,3-Dimethylcyclopentane | 34.50 | 36.50 | 36.40 | -1.90 |
| 1612 | (E) 5-Decenyl acetate | 70.60 | 72.50 | 72.50 | -1.90 |
| 1613 | alpha,alpha-Dimethylbenzylcyanide | 60.60 | 62.50 | 62.50 | -1.90 |
| 1614 | 3-Thiophenecarbonitrile | 51.60 | 53.20 | 53.50 | -1.90 |
| 1615 | 8-Methylquinoline | 62.10 | 64.10 | 64.00 | -1.90 |
| 1616 | Camphor | 54.40 | 52.80 | 52.50 | 1.90 |
| 1617 | 2,4-Dimethyl-3-pentanone | 43.40 | 41.60 | 41.50 | 1.90 |
| 1618 | Octane | 41.60 | 43.50 | 43.50 | -1.90 |
| 1619 | Pentane-1,3-diamine | 54.90 | 53.30 | 53.00 | 1.90 |
| 1620 | (E) 5-Decenal | 59.20 | 61.20 | 61.10 | -1.90 |
| 1621 | (Z) 3-Decenal | 59.20 | 61.20 | 61.10 | -1.90 |
| 1622 | alpha-t-Butylstyrene | 53.20 | 54.80 | 55.10 | -1.90 |
| 1623 | Chlorocyclopentane | 38.80 | 37.00 | 36.90 | 1.90 |
| 1624 | Ethyl 4-chlorobutyrate | 52.70 | 54.80 | 54.60 | -1.90 |
| 1625 | (E) 4-Dodecenyl acetate | 79.80 | 81.90 | 81.70 | -1.90 |
| 1626 | (Z) 9-Octadecenyl acetate | 107.80 | 109.90 | 109.70 | -1.90 |
| 1627 | 1-(2-Bromophenyl)-1H-imidazole | 79.80 | 81.10 | 81.70 | -1.90 |
| 1628 | 2-(Dimethylamino)propiophenone | 64.80 | 67.20 | 66.70 | -1.90 |
| 1629 | (Z) 4-Decenal | 59.30 | 61.20 | 61.20 | -1.90 |
| 1630 | 1,1-Dimethylcyclohexane | 38.80 | 40.70 | 40.70 | -1.90 |
| 1631 | 1-Chlorohexane | 42.80 | 44.50 | 44.70 | -1.90 |
| 1632 | Benzene | 33.80 | 36.00 | 35.70 | -1.90 |
| 1633 | cis-1-Ethyl-3-methylcyclopentane | 39.30 | 41.20 | 41.20 | -1.90 |
| 1634 | Cumyl propyl ether | 59.30 | 61.10 | 61.20 | -1.90 |
| 1635 | trans-1-Ethyl-2-methylcyclopentane | 39.30 | 41.20 | 41.20 | -1.90 |
| 1636 | 1-Methylcyclohexyl acetate | 52.40 | 54.20 | 54.30 | -1.90 |
| 1637 | 2,2,3,3,3-Pentafluoro-1-propanol | 44.40 | 46.70 | 46.30 | -1.90 |
| 1638 | 2,2,6,6-Tetramethyl-4-heptanone | 52.90 | 53.80 | 54.80 | -1.90 |
| 1639 | Allylcyclopentane | 40.40 | 42.10 | 42.30 | -1.90 |
| 1640 | Bicyclo[2.2.2]octa-2,5,7-triene | 42.90 | 45.30 | 44.80 | -1.90 |
| 1641 | cis-1,2-Dimethylcyclohexane | 39.40 | 41.20 | 41.30 | -1.90 |
| 1642 | Ethyl 2-methylpentanoate | 48.40 | 50.40 | 50.30 | -1.90 |
| 1643 | S-t-Butyl thiolacetate | 42.90 | 44.20 | 44.80 | -1.90 |
| 1644 | 1,1,4-Trimethylcycloheptane | 45.50 | 47.50 | 47.40 | -1.90 |
| 1645 | 1,1-Di-p-tolylethane | 76.50 | 78.50 | 78.40 | -1.90 |
| 1646 | 1-Chloronaphthalene | 62.00 | 63.90 | 63.90 | -1.90 |
| 1647 | 1-Nitropentane | 51.00 | 52.70 | 52.90 | -1.90 |
| 1648 | Benzyl alcohol | 65.50 | 67.40 | 67.40 | -1.90 |
| 1649 | Dodecylcyclohexane | 89.50 | 91.60 | 91.40 | -1.90 |
| 1650 | Ethyl 4-pentenoate | 46.00 | 47.90 | 47.90 | -1.90 |
| 1651 | Nonanolactone | 59.00 | 61.00 | 60.90 | -1.90 |
| 1652 | 1-Isobutylimidazole | 62.20 | 64.40 | 64.10 | -1.90 |
| 1653 | (E) 3-Tetradecenyl acetate | 89.20 | 91.20 | 91.10 | -1.90 |
| 1654 | (E) 8-Tetradecenyl acetate | 89.20 | 91.20 | 91.10 | -1.90 |
| 1655 | (Z) 7-Decenyl acetate | 70.70 | 72.50 | 72.60 | -1.90 |
| 1656 | Di-n-pentyl succinate | 89.70 | 91.50 | 91.60 | -1.90 |
| 1657 | Empenthrin | 87.20 | 88.70 | 89.10 | -1.90 |
| 1658 | Ethylamine | 27.08 | 29.00 | 29.00 | -1.92 |
| 1659 | Ethylenedichloride | 35.15 | 36.90 | 37.10 | -1.95 |
| 1660 | Codlemone | 92.30 | 90.20 | 90.30 | 2.00 |
| 1661 | 2,6-Dimethylquinoline | 67.10 | 65.30 | 65.10 | 2.00 |
| 1662 | 3,4-Diethoxy-3-cyclobutene-1,2-dione | 73.60 | 72.00 | 71.60 | 2.00 |
| 1663 | 1-(4-Fluorophenyl)-1H-imidazole | 75.40 | 73.10 | 73.40 | 2.00 |
| 1664 | alpha-Methylstyrene | 48.90 | 47.00 | 46.90 | 2.00 |
| 1665 | N,N-Dimethyl-2,3-dimethyl-3-phenyl-2-butanamine | 66.40 | 64.70 | 64.40 | 2.00 |
| 1666 | 1,4-Dichlorobenzene | 47.80 | 46.00 | 45.80 | 2.00 |
| 1667 | 1-Ethylpyrazole | 53.30 | 52.20 | 51.30 | 2.00 |
| 1668 | 2-Ethylacrolein | 36.80 | 35.20 | 34.80 | 2.00 |
| 1669 | Diisopropyl disulfide | 49.30 | 47.40 | 47.30 | 2.00 |
| 1670 | Naphthalene | 60.30 | 58.40 | 58.30 | 2.00 |
| 1671 | (E) 4-Tetradecen-1-ol | 100.70 | 98.80 | 98.70 | 2.00 |
| 1672 | 1-(4-Methylphenyl)-1H-imidazole | 78.20 | 77.00 | 76.20 | 2.00 |
| 1673 | Benzo[e]pyrene | 118.20 | 116.20 | 116.20 | 2.00 |
| 1674 | 1,2,5-Trimethylpyrrole | 50.70 | 48.20 | 48.70 | 2.00 |
| 1675 | 1,3-Dichlorobutane | 42.20 | 40.10 | 40.20 | 2.00 |
| 1676 | Di-t-butylmethanol | 62.70 | 60.40 | 60.70 | 2.00 |
| 1677 | Trimethoxy(2-(methylthio)ethyl)silane | 45.20 | 43.60 | 43.20 | 2.00 |
| 1678 | 2,3,3-Trimethylhexane | 42.10 | 39.60 | 40.10 | 2.00 |
| 1679 | 2-Nitrotoluene | 59.60 | 57.80 | 57.60 | 2.00 |
| 1680 | Ethyl isopropyl sulfide | 38.10 | 36.40 | 36.10 | 2.00 |
| 1681 | Isopropyl methyl sulfide | 34.10 | 32.30 | 32.10 | 2.00 |
| 1682 | 2-Chloro-1,1,1,2-tetrafluoropropane | 24.60 | 23.40 | 22.60 | 2.00 |
| 1683 | (Z) 10-Hexadecenyl acetate | 98.50 | 100.50 | 100.50 | -2.00 |
| 1684 | (Z) 2-Dodecenal | 72.50 | 70.50 | 70.50 | 2.00 |
| 1685 | (Z) 7-Hexadecenyl acetate | 98.50 | 100.50 | 100.50 | -2.00 |
| 1686 | 1,1-Dimethylcyclopentane | 34.00 | 36.00 | 36.00 | -2.00 |
| 1687 | 2,2-Dimethyloctane | 49.00 | 47.10 | 47.00 | 2.00 |
| 1688 | 2-Ethylhexyl laurate | 104.50 | 106.40 | 106.50 | -2.00 |
| 1689 | 4-Methylcyclopentene | 32.00 | 34.20 | 34.00 | -2.00 |
| 1690 | Bicyclo(2.1.0)pentane | 28.00 | 30.10 | 30.00 | -2.00 |
| 1691 | Ethyl trichloroacetate | 51.00 | 48.80 | 49.00 | 2.00 |
| 1692 | Hexabromobenzene | 85.00 | 83.50 | 83.00 | 2.00 |
| 1693 | Trichloroethylene | 34.50 | 36.50 | 36.50 | -2.00 |
| 1694 | 2-Fluorobenzotrifluoride | 38.10 | 39.90 | 40.10 | -2.00 |
| 1695 | 1-n-Propylimidazole | 61.10 | 62.40 | 63.10 | -2.00 |
| 1696 | 2-Ethylaniline | 60.60 | 62.70 | 62.60 | -2.00 |
| 1697 | Heptane | 36.60 | 38.70 | 38.60 | -2.00 |
| 1698 | Butane | 22.40 | 24.40 | 24.40 | -2.00 |
| 1699 | 1,4-Dihydronaphthalene | 54.20 | 56.40 | 56.20 | -2.00 |
| 1700 | 1,4-Pentadiene | 25.20 | 27.30 | 27.20 | -2.00 |
| 1701 | 2-Methyl-2-heptene | 39.70 | 41.60 | 41.70 | -2.00 |
| 1702 | cis-3-Ethyl-2-hexene | 39.70 | 41.60 | 41.70 | -2.00 |
| 1703 | cis-4-Methyl-3-heptene | 39.70 | 41.60 | 41.70 | -2.00 |
| 1704 | trans-3-Ethyl-2-hexene | 39.70 | 41.60 | 41.70 | -2.00 |
| 1705 | 2-Ethylnaphthalene | 64.70 | 66.60 | 66.70 | -2.00 |
| 1706 | 8-Hexyl-8-pentylhexadecane | 125.70 | 128.00 | 127.70 | -2.00 |
| 1707 | Dipropyl malonate | 66.20 | 68.20 | 68.20 | -2.00 |
| 1708 | 4-Isopropyl-2-methylphenol | 73.20 | 74.90 | 75.20 | -2.00 |
| 1709 | Methyl (Z) 10-pentadecenoate | 91.70 | 93.70 | 93.70 | -2.00 |
| 1710 | Methyllaurate | 77.20 | 79.00 | 79.20 | -2.00 |
| 1711 | 1,2-Dibutoxyethane | 58.80 | 60.90 | 60.80 | -2.00 |
| 1712 | 3-Phenylpropene | 46.30 | 48.30 | 48.30 | -2.00 |
| 1713 | cis-1,3-Dimethylcyclopentane | 34.30 | 36.50 | 36.30 | -2.00 |
| 1714 | Diethylamine | 31.30 | 33.40 | 33.30 | -2.00 |
| 1715 | 1,1-Diphenylethane | 68.90 | 71.20 | 70.90 | -2.00 |
| 1716 | 2-Nitro-m-xylene | 59.40 | 61.50 | 61.40 | -2.00 |
| 1717 | Propan-1,3-diol | 72.40 | 74.00 | 74.40 | -2.00 |
| 1718 | N-Ethyloctahydrocarbazole | 81.90 | 82.30 | 83.90 | -2.00 |
| 1719 | (E) 6-Decenyl acetate | 70.60 | 72.50 | 72.60 | -2.00 |
| 1720 | (E) 8-Hexadecenyl acetate | 98.60 | 100.50 | 100.60 | -2.00 |
| 1721 | Heptyl butyrate | 65.10 | 67.10 | 67.10 | -2.00 |
| 1722 | Methyl (Z) 9-tetradecenoate | 87.10 | 89.10 | 89.10 | -2.00 |
| 1723 | Undecylcyclohexane | 84.60 | 86.80 | 86.60 | -2.00 |
| 1724 | (E) 3-Dodecenyl acetate | 79.80 | 81.90 | 81.80 | -2.00 |
| 1725 | Ethyl dodecanoate | 79.30 | 81.20 | 81.30 | -2.00 |
| 1726 | Methyl (Z) 10-heptadecenoate | 100.80 | 103.10 | 102.80 | -2.00 |
| 1727 | N-Methylmorpholine | 39.31 | 37.50 | 37.30 | 2.01 |
| 1728 | 3-Chlorobiphenyl | 72.29 | 70.40 | 70.20 | 2.09 |
| 1729 | 10-Hexyl-10-methyleicosane | 129.90 | 128.00 | 127.80 | 2.10 |
| 1730 | (Z) 7-Hexadecen-1-ol | 110.20 | 108.10 | 108.10 | 2.10 |
| 1731 | 3-Fluorobenzamide | 83.20 | 80.30 | 81.10 | 2.10 |
| 1732 | Menthol | 74.20 | 72.00 | 72.10 | 2.10 |
| 1733 | 1,10-Phenanthroline | 86.20 | 84.30 | 84.10 | 2.10 |
| 1734 | 1,2-Dibromohexane | 56.50 | 54.50 | 54.40 | 2.10 |
| 1735 | 2,2',4,4',5,6'-Hexabromodiphenyl ether | 113.00 | 111.00 | 110.90 | 2.10 |
| 1736 | Ethylene glycol monobutyl ether acetate | 59.50 | 57.50 | 57.40 | 2.10 |
| 1737 | trans-Citral | 61.00 | 59.20 | 58.90 | 2.10 |
| 1738 | N-Cyclohexylpiperidine | 60.50 | 58.20 | 58.40 | 2.10 |
| 1739 | 2,4-Dimethyl-4-ethylhexane | 46.40 | 44.30 | 44.30 | 2.10 |
| 1740 | 2-Cyanobutane | 42.40 | 40.30 | 40.30 | 2.10 |
| 1741 | Butyl propanoate | 50.40 | 48.50 | 48.30 | 2.10 |
| 1742 | 2-Methylthiolane | 41.80 | 39.60 | 39.70 | 2.10 |
| 1743 | Thietane | 35.80 | 33.80 | 33.70 | 2.10 |
| 1744 | 1-Iodooctadecane | 109.30 | 107.30 | 107.20 | 2.10 |
| 1745 | 3,3-Dimethyl-1-pentene | 33.50 | 31.10 | 31.40 | 2.10 |
| 1746 | 3-Chloronitrobenzene | 60.20 | 58.90 | 58.10 | 2.10 |
| 1747 | Methacrolein | 32.20 | 30.60 | 30.10 | 2.10 |
| 1748 | Propanal | 29.70 | 27.80 | 27.60 | 2.10 |
| 1749 | Triethylphosphate | 55.70 | 53.20 | 53.60 | 2.10 |
| 1750 | (E) 6-Tetradecenyl acetate | 88.90 | 91.20 | 91.00 | -2.10 |
| 1751 | (Z) 11-Octadecen-1-ol | 119.60 | 117.50 | 117.50 | 2.10 |
| 1752 | Prehnitene | 52.60 | 50.60 | 50.50 | 2.10 |
| 1753 | 2,2-Dimethoxypropane | 37.60 | 35.60 | 35.50 | 2.10 |
| 1754 | 2-Methyl-1,3-dioxolane | 35.10 | 32.90 | 33.00 | 2.10 |
| 1755 | 3-Ethoxyacetanilide | 94.10 | 91.20 | 92.00 | 2.10 |
| 1756 | Methyl palmitoleate | 96.40 | 98.40 | 98.50 | -2.10 |
| 1757 | N-Cyclopentylpiperidine | 55.10 | 53.50 | 53.00 | 2.10 |
| 1758 | Amylamine | 40.90 | 43.00 | 43.00 | -2.10 |
| 1759 | Pyrimidine | 44.10 | 41.90 | 42.00 | 2.10 |
| 1760 | Isopropyl methyl ether | 26.40 | 28.60 | 28.50 | -2.10 |
| 1761 | 1,2,3,4-Tetrafluorobenzene | 37.50 | 35.70 | 35.40 | 2.10 |
| 1762 | 4-Methylpentyl t-octyl ether | 57.50 | 59.70 | 59.60 | -2.10 |
| 1763 | Indane | 49.00 | 51.10 | 51.10 | -2.10 |
| 1764 | Methanol | 38.00 | 39.90 | 40.10 | -2.10 |
| 1765 | Methyl pentafluoropropanoate | 35.00 | 36.90 | 37.10 | -2.10 |
| 1766 | t-Butylamine | 30.50 | 32.70 | 32.60 | -2.10 |
| 1767 | (E) 5-Tetradecenyl acetate | 89.10 | 91.20 | 91.20 | -2.10 |
| 1768 | (Z) 2-Tetradecenyl acetate | 89.10 | 91.20 | 91.20 | -2.10 |
| 1769 | 4-Ethyloctane | 48.10 | 50.30 | 50.20 | -2.10 |
| 1770 | Di-n-propylsulfate | 66.90 | 65.40 | 64.80 | 2.10 |
| 1771 | Spiro[5,5]undecane | 56.10 | 58.20 | 58.20 | -2.10 |
| 1772 | trans-1,3-Dimethylcyclohexane | 39.10 | 41.20 | 41.20 | -2.10 |
| 1773 | 1,1,1,2,2,3,3,4,4-Nonafluorononane | 45.70 | 47.60 | 47.80 | -2.10 |
| 1774 | 4-Octyne | 42.70 | 45.00 | 44.80 | -2.10 |
| 1775 | Allyl hexanoate | 55.20 | 57.30 | 57.30 | -2.10 |
| 1776 | Dimethylacetamide | 50.70 | 52.80 | 52.80 | -2.10 |
| 1777 | Methyl cyclobutanecarboxylate | 44.70 | 46.80 | 46.80 | -2.10 |
| 1778 | Perfluoro-1-decene | 45.20 | 47.30 |  | -2.10 |
| 1779 | Dibutyl malonate | 75.30 | 77.50 | 77.40 | -2.10 |
| 1780 | 1,1-Dimethylbutyl 2,2-dimethylpropanoate | 52.30 | 54.40 | 54.40 | -2.10 |
| 1781 | 3-Methylbutyl chloroacetate | 54.80 | 56.80 | 56.90 | -2.10 |
| 1782 | Butyl cumyl ether | 63.80 | 65.80 | 65.90 | -2.10 |
| 1783 | Cyclohexylbenzene | 60.80 | 63.10 | 62.90 | -2.10 |
| 1784 | Heptylcyclopentane | 60.80 | 63.00 | 62.90 | -2.10 |
| 1785 | Perfluoroheptane | 36.30 | 38.30 | 38.40 | -2.10 |
| 1786 | 2,3:7,8-Dibenzocycloocta-2,7-dien-1-one | 92.00 | 94.50 | 94.10 | -2.10 |
| 1787 | (Z) 2-Dodecenyl acetate | 79.70 | 81.90 | 81.80 | -2.10 |
| 1788 | 15-Crown-5 | 75.70 | 77.20 | 77.80 | -2.10 |
| 1789 | Decylcyclohexane | 79.70 | 82.00 | 81.80 | -2.10 |
| 1790 | 1,4-Dioxatetralin | 50.42 | 48.20 | 48.30 | 2.12 |
| 1791 | 4-Chlorobiphenyl | 72.54 | 70.40 | 70.40 | 2.14 |
| 1792 | Butyl propyl ether | 40.26 | 42.50 | 42.40 | -2.14 |
| 1793 | Trimethylamine | 22.85 | 25.10 | 25.00 | -2.15 |
| 1794 | 2,2,2-Trifluoroethanol | 43.97 | 41.90 | 41.80 | 2.17 |
| 1795 | 1-Iodononadecane | 113.80 | 112.00 | 111.60 | 2.20 |
| 1796 | Di-n-butylsulfite | 67.80 | 66.10 | 65.60 | 2.20 |
| 1797 | O,O-Dimethyl-S-[2-(ethylthio)ethyl]dithiophosphate | 76.80 | 75.40 | 74.60 | 2.20 |
| 1798 | (E) 2-Dodecenal | 72.60 | 70.50 | 70.40 | 2.20 |
| 1799 | (Z) 2-Tetradecen-1-ol | 101.10 | 98.80 | 98.90 | 2.20 |
| 1800 | 2-Tetradecanone | 82.10 | 79.70 | 79.90 | 2.20 |
| 1801 | Ethyl dichloroacetate | 50.60 | 48.30 | 48.40 | 2.20 |
| 1802 | Heptyl t-butyl ether | 56.60 | 54.40 | 54.40 | 2.20 |
| 1803 | Myrcene | 50.60 | 48.30 | 48.40 | 2.20 |
| 1804 | Hemimellitene | 49.00 | 47.00 | 46.80 | 2.20 |
| 1805 | 2,2,4,4-Tetramethylpentane | 38.50 | 36.30 | 36.30 | 2.20 |
| 1806 | 2-Acetylfuran | 53.00 | 51.00 | 50.80 | 2.20 |
| 1807 | cis-1,2-Dichloroethylene | 30.90 | 29.40 | 28.70 | 2.20 |
| 1808 | 2,2',4,5'-Tetrachlorobiphenyl | 87.40 | 84.70 | 85.20 | 2.20 |
| 1809 | 2,2,6-Trimethylheptane | 46.40 | 44.30 | 44.20 | 2.20 |
| 1810 | 2,4-Dimethyl-3-ethylhexane | 46.90 | 44.80 | 44.70 | 2.20 |
| 1811 | 3,3-Dimethyl-4-ethylhexane | 46.40 | 44.30 | 44.20 | 2.20 |
| 1812 | 3,4,4-Trimethylheptane | 46.40 | 44.30 | 44.20 | 2.20 |
| 1813 | 3,4-Dimethyl-1-pentene | 33.90 | 31.60 | 31.70 | 2.20 |
| 1814 | Cellosolve acetate | 63.90 | 61.70 | 61.70 | 2.20 |
| 1815 | Ethyl t-butyl ether | 33.10 | 31.00 | 30.90 | 2.20 |
| 1816 | 3-Bromochlorobenzene | 51.30 | 49.10 | 49.10 | 2.20 |
| 1817 | 2,3,4-Trimethyl-2-pentene | 39.30 | 37.50 | 37.10 | 2.20 |
| 1818 | Pinacolone | 38.30 | 36.40 | 36.10 | 2.20 |
| 1819 | 4-Fluoro-2-methylaniline | 59.80 | 57.80 | 57.60 | 2.20 |
| 1820 | 1,2-Dibromobenzene | 54.30 | 52.10 | 52.10 | 2.20 |
| 1821 | 2,3-Dimethyl-1,3-butadiene | 31.00 | 29.20 | 28.80 | 2.20 |
| 1822 | 2-Bromofluorene | 81.70 | 79.40 | 79.50 | 2.20 |
| 1823 | tri-n-Amylphosphate | 92.30 | 95.20 | 94.50 | -2.20 |
| 1824 | 2,2-Dimethyl-3-ethylpentane | 41.70 | 39.60 | 39.50 | 2.20 |
| 1825 | 3-Ethyl-3-hexene | 39.30 | 41.60 | 41.50 | -2.20 |
| 1826 | 3-Methylcyclohexyl propanoate | 58.30 | 60.50 | 60.50 | -2.20 |
| 1827 | 3-Methylthiolane | 42.70 | 40.40 | 40.50 | 2.20 |
| 1828 | 5,5-Dimethyl-1-hexene | 37.70 | 35.70 | 35.50 | 2.20 |
| 1829 | Phenol | 58.80 | 60.80 | 61.00 | -2.20 |
| 1830 | 1,3-Dihydroisobenzofuran | 53.70 | 51.50 | 51.50 | 2.20 |
| 1831 | 1,1-Dimethylindane | 51.90 | 54.40 | 54.10 | -2.20 |
| 1832 | 1,3,5-Trifluorobenzene | 33.90 | 35.90 | 36.10 | -2.20 |
| 1833 | 1-Iodopentane | 44.40 | 46.60 | 46.60 | -2.20 |
| 1834 | 4-Tolunitrile | 55.90 | 58.20 | 58.10 | -2.20 |
| 1835 | Cyclohexanethiol | 44.90 | 47.10 | 47.10 | -2.20 |
| 1836 | Diisopropyl sulfide | 39.60 | 37.60 | 37.40 | 2.20 |
| 1837 | Hexylcyclopentane | 55.90 | 58.20 | 58.10 | -2.20 |
| 1838 | Neopentyl pivalate | 48.90 | 50.60 | 51.10 | -2.20 |
| 1839 | Methylenecyclobutane | 27.70 | 29.70 | 29.90 | -2.20 |
| 1840 | (E) 4-Tetradecenyl acetate | 89.00 | 91.20 | 91.20 | -2.20 |
| 1841 | (E) 7-Tetradecenyl acetate | 89.00 | 91.20 | 91.20 | -2.20 |
| 1842 | 1-Butylamine | 36.00 | 38.30 | 38.20 | -2.20 |
| 1843 | 1-Pentene | 25.50 | 27.90 | 27.70 | -2.20 |
| 1844 | cis-1,4-Dimethylcyclohexane | 39.00 | 41.20 | 41.20 | -2.20 |
| 1845 | cis-2-Hexene | 31.50 | 33.70 | 33.70 | -2.20 |
| 1846 | Dimethylamine | 26.50 | 28.60 | 28.70 | -2.20 |
| 1847 | Ethyl methyl sulfite | 43.50 | 45.20 | 45.70 | -2.20 |
| 1848 | Methyl nonadecanoate | 109.50 | 111.70 | 111.70 | -2.20 |
| 1849 | trans-2-Heptene | 36.00 | 38.30 | 38.20 | -2.20 |
| 1850 | Tributylamine | 58.00 | 60.30 | 60.20 | -2.20 |
| 1851 | trans-3-Hexene | 31.60 | 33.70 | 33.80 | -2.20 |
| 1852 | 1-Chloro-1,2,2-trifluoro-2-(difluoromethoxy)ethane | 32.60 | 34.10 | 34.80 | -2.20 |
| 1853 | 1,1,3-Trimethylbutyl 2,2-dimethylpropanoate | 54.20 | 56.30 | 56.40 | -2.20 |
| 1854 | Divinyl sulfoxide | 51.20 | 51.90 | 53.40 | -2.20 |
| 1855 | Vinylcyclohexane | 39.70 | 42.10 | 41.90 | -2.20 |
| 1856 | 3,4-Dibromodiphenyl ether | 77.40 | 79.60 | 79.60 | -2.20 |
| 1857 | Heptylbenzene | 64.90 | 67.30 | 67.10 | -2.20 |
| 1858 | (Z) 9-Pentadecenyl acetate | 93.60 | 95.90 | 95.80 | -2.20 |
| 1859 | (Z) 9-Tetradecenyl acetate | 89.10 | 91.20 | 91.30 | -2.20 |
| 1860 | Methyl tetradecanoate | 86.10 | 88.30 | 88.30 | -2.20 |
| 1861 | 1,4-Dimethylpiperazine | 40.91 | 38.90 | 38.70 | 2.21 |
| 1862 | Isopropylnitrate | 38.79 | 40.90 | 41.00 | -2.21 |
| 1863 | Methyl pentacosanoate | 142.00 | 139.70 | 139.70 | 2.30 |
| 1864 | 2,2',4,5',6-Pentachlorobiphenyl | 91.60 | 89.50 | 89.30 | 2.30 |
| 1865 | (E) 9-Tridecen-1-ol | 96.40 | 94.10 | 94.10 | 2.30 |
| 1866 | (Z) 8-Hexadecen-1-ol | 110.40 | 108.10 | 108.10 | 2.30 |
| 1867 | 1-Iodoheptadecane | 104.70 | 102.60 | 102.40 | 2.30 |
| 1868 | 2-Pyrrolidone | 68.70 | 66.40 | 66.40 | 2.30 |
| 1869 | 1,3,5-Trimethylhexahydro-s-triazine | 51.20 | 48.90 |  | 2.30 |
| 1870 | Ethane-1,2-dithiol | 44.70 | 42.60 | 42.40 | 2.30 |
| 1871 | 1,8-Cineole | 48.70 | 46.60 | 46.40 | 2.30 |
| 1872 | n-Heptaldehyde | 48.70 | 46.50 | 46.40 | 2.30 |
| 1873 | N,N-Dimethylaminoacetone | 43.60 | 41.10 | 41.30 | 2.30 |
| 1874 | 1-Octadecene | 90.00 | 87.70 | 87.70 | 2.30 |
| 1875 | 2-Bromopentane | 38.50 | 36.20 | 36.20 | 2.30 |
| 1876 | 3-Chlorophenol | 63.50 | 65.60 | 65.80 | -2.30 |
| 1877 | 3-Ethyl-4-methyl-1-pentene | 38.50 | 36.20 | 36.20 | 2.30 |
| 1878 | 4-Iodoacetophenone | 71.00 | 68.60 | 68.70 | 2.30 |
| 1879 | Methyl 3-aminobenzoate | 78.50 | 75.60 | 76.20 | 2.30 |
| 1880 | Propanenitrile | 36.00 | 33.70 | 33.70 | 2.30 |
| 1881 | 2-Chlorobutane | 31.50 | 29.10 | 29.20 | 2.30 |
| 1882 | 2-Methyl-2-butanol | 51.50 | 49.20 | 49.20 | 2.30 |
| 1883 | 1-Ethyl-2-methylimidazole | 60.40 | 59.50 | 58.10 | 2.30 |
| 1884 | 2,3-Dimethyl-4-ethylhexane | 46.90 | 44.80 | 44.60 | 2.30 |
| 1885 | 2,4,5-Trimethylheptane | 46.90 | 44.80 | 44.60 | 2.30 |
| 1886 | 2,6-Dimethoxypyridine | 53.90 | 51.60 |  | 2.30 |
| 1887 | t-Amyl mercaptan | 35.60 | 37.70 | 37.90 | -2.30 |
| 1888 | 2-Methyl-2-propenol | 51.90 | 49.50 | 49.60 | 2.30 |
| 1889 | 3,4-Dimethyl-4-ethylhexane | 46.40 | 44.30 | 44.10 | 2.30 |
| 1890 | Cyclobutanone | 38.40 | 36.20 | 36.10 | 2.30 |
| 1891 | Quadricyclane | 37.90 | 35.10 | 35.60 | 2.30 |
| 1892 | trans-4-Methylcyclohexanol | 65.90 | 63.40 | 63.60 | 2.30 |
| 1893 | Benzoyl chloride | 54.80 | 52.50 | 52.50 | 2.30 |
| 1894 | Dipropyl ether | 35.70 | 37.80 | 38.00 | -2.30 |
| 1895 | Isopentyl alcohol | 55.30 | 53.30 | 53.00 | 2.30 |
| 1896 | N,N-Diethyl-2-(1-methylhydrazino)ethanamine | 61.80 | 59.50 |  | 2.30 |
| 1897 | (Z) 9-Hexadecenyl acetate | 98.20 | 100.50 | 100.50 | -2.30 |
| 1898 | Methyl pentadecanoate | 90.70 | 93.00 | 93.00 | -2.30 |
| 1899 | Hexadecyl acetate | 102.30 | 99.80 | 100.00 | 2.30 |
| 1900 | Spiro[2.2]pentane | 27.50 | 29.70 | 29.80 | -2.30 |
| 1901 | Azidocyclopentane | 41.30 | 43.10 | 43.60 | -2.30 |
| 1902 | cis-2-Pentene | 26.80 | 29.10 | 29.10 | -2.30 |
| 1903 | 1-Butylimidazole | 63.90 | 67.10 | 66.20 | -2.30 |
| 1904 | 1-Ethylbenzimidazole | 76.10 | 70.80 | 73.80 | 2.30 |
| 1905 | 2-Chloroaniline | 56.40 | 59.10 | 58.70 | -2.30 |
| 1906 | Acetophenone dimethyl ketal | 58.90 | 61.00 | 61.20 | -2.30 |
| 1907 | N-Methylcyclohexylamine | 46.40 | 49.00 | 48.70 | -2.30 |
| 1908 | trans-1-Ethyl-3-methylcyclopentane | 38.90 | 41.20 | 41.20 | -2.30 |
| 1909 | Norpempidine | 47.00 | 48.80 | 49.30 | -2.30 |
| 1910 | Pentylcyclopentane | 51.00 | 53.50 | 53.30 | -2.30 |
| 1911 | (E) 4-Decenyl acetate | 70.10 | 72.50 | 72.40 | -2.30 |
| 1912 | (Z) 5-Decenyl acetate | 70.10 | 72.50 | 72.40 | -2.30 |
| 1913 | 2-Methyl-1,3-propanediol | 73.60 | 75.90 | 75.90 | -2.30 |
| 1914 | 3-Methylcyclohexyl acetate | 53.60 | 55.90 | 55.90 | -2.30 |
| 1915 | Azacyclotridecan-2-one | 101.60 | 103.70 | 103.90 | -2.30 |
| 1916 | Phenylacetone | 56.10 | 58.40 | 58.40 | -2.30 |
| 1917 | Butylmethylamine | 38.10 | 40.30 | 40.40 | -2.30 |
| 1918 | Di-n-hexylether | 63.60 | 65.80 | 65.90 | -2.30 |
| 1919 | Diisopropyl methylphosphonate | 57.60 | 59.90 |  | -2.30 |
| 1920 | Hexafluoro-2-propanol | 41.60 | 44.10 | 43.90 | -2.30 |
| 1921 | Methyl benzoate | 55.60 | 57.70 | 57.90 | -2.30 |
| 1922 | Nonylbenzene | 74.10 | 76.50 | 76.40 | -2.30 |
| 1923 | (Z) 7-tridecenyl acetate | 84.30 | 86.50 | 86.60 | -2.30 |
| 1924 | 1-Methylindole | 61.80 | 61.80 | 64.10 | -2.30 |
| 1925 | (E) 9-Tetradecen-1-ol | 101.00 | 98.80 | 98.60 | 2.40 |
| 1926 | 4-Acetanisidine | 92.00 | 89.10 | 89.60 | 2.40 |
| 1927 | Methyl docosanoate | 127.50 | 125.70 | 125.10 | 2.40 |
| 1928 | Methyl linolenate | 110.50 | 109.20 | 108.10 | 2.40 |
| 1929 | Methyl p-tolyl sulfone | 90.00 | 87.20 | 87.60 | 2.40 |
| 1930 | 2,3,6-Trimethylheptane | 47.30 | 44.80 | 44.90 | 2.40 |
| 1931 | 2-Ethyl-1-butanol | 60.30 | 58.00 | 57.90 | 2.40 |
| 1932 | 12-Crown-4 | 65.70 | 63.50 | 63.30 | 2.40 |
| 1933 | 3,3,4-Trimethylhexane | 42.20 | 39.60 | 39.80 | 2.40 |
| 1934 | (Z) 10-Tetradecen-1-ol | 101.10 | 98.80 | 98.70 | 2.40 |
| 1935 | endo-Norbornene oxide | 44.10 | 41.80 | 41.70 | 2.40 |
| 1936 | 1,3-Dimethylcyclopentene | 35.00 | 37.40 | 37.40 | -2.40 |
| 1937 | 2,3-Dimethyl-2-butanol | 54.00 | 51.20 | 51.60 | 2.40 |
| 1938 | 2-Chloropentane | 36.00 | 33.70 | 33.60 | 2.40 |
| 1939 | 4,5-Dimethyl-1-hexene | 38.50 | 36.20 | 36.10 | 2.40 |
| 1940 | Propanone | 30.50 | 28.30 | 28.10 | 2.40 |
| 1941 | Ethyl 2-chloropropionate | 46.50 | 44.10 | 44.10 | 2.40 |
| 1942 | Cryofluorane | 23.20 | 21.90 | 20.80 | 2.40 |
| 1943 | (E) 10-Dodecen-1-ol | 91.90 | 89.40 | 89.50 | 2.40 |
| 1944 | (E) 3-Decenyl acetate | 70.10 | 72.50 | 72.50 | -2.40 |
| 1945 | (Z) 6-Decenyl acetate | 70.10 | 72.50 | 72.50 | -2.40 |
| 1946 | 1-Butylbenzimidazole | 83.40 | 80.20 | 81.00 | 2.40 |
| 1947 | 1-Methyl-1-vinylsilacyclobutane | 33.10 | 35.50 |  | -2.40 |
| 1948 | 2,3-Dimethyl-3-ethylhexane | 46.90 | 44.30 | 44.50 | 2.40 |
| 1949 | Dipentyl disulfide | 71.10 | 73.10 | 73.50 | -2.40 |
| 1950 | N,N-Diisobutylaminotriethylsilane | 51.40 | 49.30 | 49.00 | 2.40 |
| 1951 | Octyl t-butyl ether | 61.40 | 59.10 | 59.00 | 2.40 |
| 1952 | Phenanthrene | 78.60 | 80.70 | 81.00 | -2.40 |
| 1953 | 2-Phenylethylamine | 56.60 | 59.10 | 59.00 | -2.40 |
| 1954 | 2-Ethylpyridine | 44.70 | 46.80 | 47.10 | -2.40 |
| 1955 | Methyl dimethylmalonate | 55.70 | 57.90 | 58.10 | -2.40 |
| 1956 | (Z) 8-Dodecenyl acetate | 79.30 | 81.90 | 81.70 | -2.40 |
| 1957 | Tritane | 93.80 | 96.30 | 96.20 | -2.40 |
| 1958 | s-Triazine | 38.80 | 39.40 | 41.20 | -2.40 |
| 1959 | 1-Propylamine | 31.30 | 33.60 | 33.70 | -2.40 |
| 1960 | 2-Isopropylaniline | 61.80 | 64.60 | 64.20 | -2.40 |
| 1961 | 2-Methoxyethylamine | 38.30 | 40.60 | 40.70 | -2.40 |
| 1962 | 4-Methylcyclohexene | 36.30 | 38.80 | 38.70 | -2.40 |
| 1963 | Hexyl t-octyl ether | 59.80 | 62.40 | 62.20 | -2.40 |
| 1964 | t-Butylmercaptan | 30.80 | 33.00 | 33.20 | -2.40 |
| 1965 | 3-Iodotoluene | 52.90 | 55.00 | 55.30 | -2.40 |
| 1966 | 1-Aminoadamantane | 59.90 | 62.10 | 62.30 | -2.40 |
| 1967 | Hexane | 31.40 | 34.00 | 33.80 | -2.40 |
| 1968 | (Z) 5-Hexadecenyl acetate | 98.00 | 100.50 | 100.40 | -2.40 |
| 1969 | 3-(Dimethylamino)-1-propanol | 57.00 | 59.40 | 59.40 | -2.40 |
| 1970 | 3-Ethylcyclopentene | 36.50 | 38.80 | 38.90 | -2.40 |
| 1971 | 4,4-Dipropylundecane | 78.00 | 80.40 | 80.40 | -2.40 |
| 1972 | 4-Ethylcyclopentene | 36.50 | 38.80 | 38.90 | -2.40 |
| 1973 | Bicyclo[4.4.1]undeca-1,3,5,7,9-pentaene | 61.00 | 63.30 | 63.40 | -2.40 |
| 1974 | Isopentylbenzene | 53.00 | 55.30 | 55.40 | -2.40 |
| 1975 | Naproxen | 109.00 | 111.30 | 111.40 | -2.40 |
| 1976 | (Z) 8-Tetradecenyl acetate | 88.70 | 91.20 | 91.10 | -2.40 |
| 1977 | Azetidine | 34.12 | 31.90 | 31.70 | 2.42 |
| 1978 | 1,4-Dioxane | 38.33 | 36.10 | 35.90 | 2.43 |
| 1979 | 2',3,3',4,4',5,6-Heptabromodiphenyl ether | 121.15 | 118.90 | 118.70 | 2.45 |
| 1980 | 3-Isopropyl-1,2-dihydroxybenzene | 97.80 | 95.50 | 95.30 | 2.50 |
| 1981 | 4-Fluorobenzamide | 83.30 | 80.30 | 80.80 | 2.50 |
| 1982 | Methyl Z,Z,Z,Z,Z,Z-4,7,10,13,16,19-docosahexaeneoate | 131.80 | 130.00 | 129.30 | 2.50 |
| 1983 | 2-Chlorofluorene | 79.10 | 76.30 | 76.60 | 2.50 |
| 1984 | (E) 9-Pentadecen-1-ol | 105.90 | 103.40 | 103.40 | 2.50 |
| 1985 | 1,4-Di-t-butylbenzene | 61.40 | 59.00 | 58.90 | 2.50 |
| 1986 | 2-Ethyl-m-xylene | 53.90 | 51.60 | 51.40 | 2.50 |
| 1987 | 3-Chloropyridine | 47.90 | 45.70 | 45.40 | 2.50 |
| 1988 | 4-Ethyl-o-xylene | 53.90 | 51.60 | 51.40 | 2.50 |
| 1989 | cis-4-Methylcyclohexanol | 65.90 | 63.40 | 63.40 | 2.50 |
| 1990 | Sabinene | 46.90 | 44.50 | 44.40 | 2.50 |
| 1991 | Tetracosane | 121.90 | 119.60 | 119.40 | 2.50 |
| 1992 | 1,3-Diaminopropane | 50.30 | 47.90 | 47.80 | 2.50 |
| 1993 | 2,3,4-Trimethylheptane | 47.30 | 44.80 | 44.80 | 2.50 |
| 1994 | 4-Pentenoic acid | 65.80 | 63.30 | 63.30 | 2.50 |
| 1995 | (E) 9-Dodecen-1-ol | 91.70 | 89.40 | 89.20 | 2.50 |
| 1996 | 1-Chlorododecane | 70.30 | 72.50 | 72.80 | -2.50 |
| 1997 | 3-Bromoquinoline | 70.70 | 68.20 | 68.20 | 2.50 |
| 1998 | cis-5-Octenyl propionate | 65.30 | 67.90 | 67.80 | -2.50 |
| 1999 | Methyltris(4-methylphenoxy)silane | 70.30 | 72.40 | 72.80 | -2.50 |
| 2000 | Tetrakis(2-methylphenoxy)silane | 76.20 | 72.90 | 73.70 | 2.50 |
| 2001 | Ethyl t-amyl ether | 38.20 | 35.70 | 35.70 | 2.50 |
| 2002 | Triptane | 32.40 | 30.00 | 29.90 | 2.50 |
| 2003 | 3-Carene | 48.10 | 45.80 | 45.60 | 2.50 |
| 2004 | Di-n-propylsulfite | 58.60 | 56.70 | 56.10 | 2.50 |
| 2005 | N-Isopropylaminotriethylsilane | 38.60 | 36.90 | 36.10 | 2.50 |
| 2006 | (Z) 6-Hexadecen-1-ol | 110.50 | 108.10 | 108.00 | 2.50 |
| 2007 | 2-Bromobutane | 34.50 | 31.50 | 32.00 | 2.50 |
| 2008 | Allyl cyanide | 40.00 | 37.90 | 37.50 | 2.50 |
| 2009 | Dipropyldiethoxysilane | 46.50 | 48.70 | 49.00 | -2.50 |
| 2010 | n-Butylformate | 40.10 | 42.40 | 42.60 | -2.50 |
| 2011 | Di-n-butylether | 44.70 | 47.20 | 47.20 | -2.50 |
| 2012 | Formaldehyde diethylacetal | 35.70 | 38.00 | 38.20 | -2.50 |
| 2013 | Hexylbenzene | 60.20 | 62.70 | 62.70 | -2.50 |
| 2014 | (Z) 5-Dodecenyl acetate | 79.20 | 81.90 | 81.70 | -2.50 |
| 2015 | Phenethyl alcohol | 69.70 | 72.10 | 72.20 | -2.50 |
| 2016 | Nonylcyclohexane | 74.70 | 77.30 | 77.20 | -2.50 |
| 2017 | 4-Fluorobenzonitrile | 51.80 | 54.30 | 54.30 | -2.50 |
| 2018 | Butylbenzene | 50.80 | 53.50 | 53.30 | -2.50 |
| 2019 | cis-3-Hexene | 31.30 | 33.70 | 33.80 | -2.50 |
| 2020 | Octyl propyl ether | 58.80 | 61.20 | 61.30 | -2.50 |
| 2021 | (1-Isopropoxyethyl)benzene | 55.40 | 58.40 | 57.90 | -2.50 |
| 2022 | 1-Chlorononane | 55.90 | 58.50 | 58.40 | -2.50 |
| 2023 | 2-Benzylpyridine | 69.90 | 72.30 | 72.40 | -2.50 |
| 2024 | 2,4,6-Tribromodiphenyl ether | 85.10 | 87.50 | 87.60 | -2.50 |
| 2025 | 4-Chlorododecane | 64.10 | 66.40 | 66.60 | -2.50 |
| 2026 | (Z) 3-Dodecenyl acetate | 79.30 | 81.90 | 81.80 | -2.50 |
| 2027 | (Z) 6-Dodecenyl acetate | 79.30 | 81.90 | 81.80 | -2.50 |
| 2028 | 1-Octyl-2-methylimidazole | 83.80 | 87.50 | 86.30 | -2.50 |
| 2029 | (Z) 7-Hexadecenyl acetate | 97.80 | 100.50 | 100.30 | -2.50 |
| 2030 | 2-Methylpropane | 19.12 | 21.70 | 21.70 | -2.58 |
| 2031 | (Z) 2-Tetradecenal | 82.50 | 79.90 | 79.90 | 2.60 |
| 2032 | beta-Farnesene | 72.50 | 70.70 | 69.90 | 2.60 |
| 2033 | O,O-Diethyl O-2-diethylaminoethyl thiophosphate | 78.50 | 75.70 | 75.90 | 2.60 |
| 2034 | 2,3,3-Trimethylheptane | 46.90 | 44.30 | 44.30 | 2.60 |
| 2035 | 2,3,5-Trimethylheptane | 47.30 | 44.80 | 44.70 | 2.60 |
| 2036 | 3,4,5-Trimethylheptane | 47.30 | 44.80 | 44.70 | 2.60 |
| 2037 | 2-Methylpropanal | 32.30 | 29.70 | 29.70 | 2.60 |
| 2038 | 3,3,6,6-Tetraethyloctane | 72.30 | 69.70 | 69.70 | 2.60 |
| 2039 | Decanedioic acid | 124.80 | 122.30 | 122.20 | 2.60 |
| 2040 | Perfluoro(isopropylcyclohexane) | 46.70 | 44.20 | 44.10 | 2.60 |
| 2041 | (E) 9-Octadecen-1-ol | 120.10 | 117.50 | 117.50 | 2.60 |
| 2042 | (Z) 4-Hexadecen-1-ol | 110.60 | 108.10 | 108.00 | 2.60 |
| 2043 | (Z) 9-Hexadecen-1-ol | 110.60 | 108.10 | 108.00 | 2.60 |
| 2044 | Enantholactam | 77.90 | 80.40 | 80.50 | -2.60 |
| 2045 | 3-Chloroaniline | 61.10 | 59.10 | 58.50 | 2.60 |
| 2046 | Methylcaprylate | 57.90 | 60.30 | 60.50 | -2.60 |
| 2047 | Benzyl mercaptan | 56.60 | 54.10 | 54.00 | 2.60 |
| 2048 | trans-3-Methyl-2-heptene | 38.90 | 41.60 | 41.50 | -2.60 |
| 2049 | trans-2-Pentene | 26.70 | 29.10 | 29.30 | -2.60 |
| 2050 | (Z) 5-Decenal | 58.50 | 61.20 | 61.10 | -2.60 |
| 2051 | 1-Methylcyclohexene | 37.50 | 40.20 | 40.10 | -2.60 |
| 2052 | 2-Methyl-2-pentanethiol | 40.00 | 42.30 | 42.60 | -2.60 |
| 2053 | 4-Iodotoluene | 52.50 | 55.00 | 55.10 | -2.60 |
| 2054 | Butylcyclopentane | 46.00 | 48.70 | 48.60 | -2.60 |
| 2055 | Perfluorocyclohexene | 31.00 | 33.00 | 33.60 | -2.60 |
| 2056 | Methyl 3-methylbutyrate | 41.00 | 43.60 | 43.60 | -2.60 |
| 2057 | n-Hexylamine | 45.00 | 47.70 | 47.60 | -2.60 |
| 2058 | cis-2-Butene | 22.10 | 24.50 | 24.70 | -2.60 |
| 2059 | 1,1-Dimethoxybutane | 41.70 | 44.10 | 44.30 | -2.60 |
| 2060 | Methylenanthate | 53.20 | 55.60 | 55.80 | -2.60 |
| 2061 | 1-Hexen-3-ol | 55.20 | 57.80 | 57.80 | -2.60 |
| 2062 | Butylethylamine | 40.20 | 42.70 | 42.80 | -2.60 |
| 2063 | Cyclohexyl isobutyrate | 57.70 | 60.50 | 60.30 | -2.60 |
| 2064 | alpha,alpha-Dimethylphenethylamine | 60.20 | 62.80 | 62.80 | -2.60 |
| 2065 | (Z) 8-Hexadecenyl acetate | 97.80 | 100.50 | 100.40 | -2.60 |
| 2066 | Phthalazine | 72.80 | 74.70 | 75.40 | -2.60 |
| 2067 | 3-Thiacyclohexanone | 49.30 | 52.00 | 51.90 | -2.60 |
| 2068 | 1-Hexanethiol | 44.80 | 47.30 | 47.40 | -2.60 |
| 2069 | Perfluoro-isobutylcyclohexane | 46.30 | 48.90 | 48.90 | -2.60 |
| 2070 | (Z) 3-Tetradecenyl acetate | 88.50 | 91.20 | 91.10 | -2.60 |
| 2071 | 2:3,6:7-Dibenzobicyclo[3.2.2]nona-2,6-dien-4-one | 94.50 | 96.90 | 97.10 | -2.60 |
| 2072 | 3,4'-Dibromodiphenyl ether | 77.00 | 79.60 | 79.60 | -2.60 |
| 2073 | Methyl tridecanoate | 80.70 | 83.70 | 83.30 | -2.60 |
| 2074 | Isobutyl nitrate | 42.18 | 45.30 | 44.80 | -2.62 |
| 2075 | N,N-Dimethylformamide | 47.57 | 48.80 | 50.20 | -2.63 |
| 2076 | (E) 2-Tetradecen-1-ol | 101.50 | 98.80 | 98.80 | 2.70 |
| 2077 | 1-Methylanthracene | 87.00 | 84.40 | 84.30 | 2.70 |
| 2078 | 2,2',3,5',6-Pentachlorobiphenyl | 92.30 | 89.50 | 89.60 | 2.70 |
| 2079 | Butyl 4-hydroxybenzoate | 95.80 | 93.60 | 93.10 | 2.70 |
| 2080 | Pentacosane | 126.80 | 124.40 | 124.10 | 2.70 |
| 2081 | 2-Propoxyethylacetate | 55.60 | 52.80 | 52.90 | 2.70 |
| 2082 | alpha-Methyl-4-isopropylbenzenepropanal | 72.60 | 70.20 | 69.90 | 2.70 |
| 2083 | Thiolan-3-one | 50.10 | 47.40 | 47.40 | 2.70 |
| 2084 | 1,2-Dichlorobenzene | 48.50 | 46.00 | 45.80 | 2.70 |
| 2085 | 2,2,4,4-Tetramethylhexane | 43.50 | 41.10 | 40.80 | 2.70 |
| 2086 | 2,2,5,5-Tetramethylhexane | 43.50 | 41.10 | 40.80 | 2.70 |
| 2087 | (E) 8-Tetradecen-1-ol | 101.40 | 98.80 | 98.70 | 2.70 |
| 2088 | 2,2,3-Trimethylheptane | 46.90 | 44.30 | 44.20 | 2.70 |
| 2089 | 2-Ethylanthracene | 91.40 | 89.00 | 88.70 | 2.70 |
| 2090 | 3,3,4-Trimethylheptane | 46.90 | 44.30 | 44.20 | 2.70 |
| 2091 | 3-Phenoxyphenol | 90.40 | 88.30 | 87.70 | 2.70 |
| 2092 | Oxetane | 29.80 | 27.00 | 27.10 | 2.70 |
| 2093 | (Z) 3-Hexadecen-1-ol | 110.70 | 108.10 | 108.00 | 2.70 |
| 2094 | 1-Methyl-2-piperidinemethanol | 68.20 | 65.80 | 65.50 | 2.70 |
| 2095 | Dibutyl phthalate | 99.30 | 102.00 | 102.00 | -2.70 |
| 2096 | Octylcyclohexane | 69.80 | 72.50 | 72.50 | -2.70 |
| 2097 | Propane-1,3-dithiol | 49.70 | 47.30 | 47.00 | 2.70 |
| 2098 | 1-Ethyladamantane | 55.30 | 58.00 | 58.00 | -2.70 |
| 2099 | 2,3,4-Trimethylpentane | 37.70 | 35.30 | 35.00 | 2.70 |
| 2100 | 2-Iodotoluene | 52.30 | 55.00 | 55.00 | -2.70 |
| 2101 | Isobutyl dichloroacetate | 52.30 | 54.90 | 55.00 | -2.70 |
| 2102 | 1,4-Diaminobutane | 55.20 | 52.50 | 52.50 | 2.70 |
| 2103 | 1,1,3-Trimethylbutyl 3,3-dimethylbutanoate | 58.40 | 61.00 | 61.10 | -2.70 |
| 2104 | Perfluoro-cis-decahydronaphthalene | 50.40 | 52.50 | 53.10 | -2.70 |
| 2105 | Methyl formate | 28.40 | 30.90 | 31.10 | -2.70 |
| 2106 | Isobutylbenzene | 48.00 | 50.70 | 50.70 | -2.70 |
| 2107 | Allylcyclohexane | 44.00 | 46.70 | 46.70 | -2.70 |
| 2108 | Butylacetamide | 75.00 | 72.50 | 72.30 | 2.70 |
| 2109 | Ethylcyclopentane | 36.50 | 39.20 | 39.20 | -2.70 |
| 2110 | Ethylidenecyclohexane | 42.00 | 44.80 | 44.70 | -2.70 |
| 2111 | N-Methyl-3-methyl-3-phenyl-2-butaneamine | 67.50 | 65.20 | 64.80 | 2.70 |
| 2112 | Methylcyclopentane | 31.60 | 34.40 | 34.30 | -2.70 |
| 2113 | 1,4-Cyclohexadiene | 35.10 | 37.60 | 37.80 | -2.70 |
| 2114 | 4-Methylpiperidine | 40.60 | 43.20 | 43.30 | -2.70 |
| 2115 | Methylnitrate | 34.10 | 36.50 | 36.80 | -2.70 |
| 2116 | Benzotrifluoride | 37.10 | 40.10 | 39.80 | -2.70 |
| 2117 | 1,1-Dimethoxy-2-phenylcyclopropane | 63.70 | 66.20 | 66.40 | -2.70 |
| 2118 | cis-3-Hexenyl propionate | 55.70 | 58.50 | 58.40 | -2.70 |
| 2119 | 2,3-Butandione | 38.70 | 41.90 | 41.40 | -2.70 |
| 2120 | Isocumene | 46.20 | 48.90 | 48.90 | -2.70 |
| 2121 | Pyrazinecarbonitrile | 58.70 | 60.90 | 61.40 | -2.70 |
| 2122 | 1,1,5-Trimethylhexyl 3,3-dimethylbutanoate | 67.40 | 70.40 | 70.10 | -2.70 |
| 2123 | 2,3,7,8-Tetrachlorodibenzo-p-dioxin | 79.90 | 82.70 | 82.60 | -2.70 |
| 2124 | 3-Menthene | 45.61 | 48.50 | 48.40 | -2.79 |
| 2125 | Glutaric acid | 101.60 | 99.00 | 98.80 | 2.80 |
| 2126 | (Z) 5-Hexadecen-1-ol | 110.90 | 108.10 | 108.10 | 2.80 |
| 2127 | 2-Methyl-2,4-pentanediol | 68.90 | 66.30 | 66.10 | 2.80 |
| 2128 | (E) 10-Pentadecen-1-ol | 106.20 | 103.40 | 103.40 | 2.80 |
| 2129 | 1,6-Hexanedioic acid | 105.20 | 103.60 | 102.40 | 2.80 |
| 2130 | Perfluorododecane | 64.20 | 62.00 | 61.40 | 2.80 |
| 2131 | 2,3,4-Trimethylhexane | 42.70 | 40.10 | 39.90 | 2.80 |
| 2132 | 4-Methylacetophenone | 60.70 | 57.50 | 57.90 | 2.80 |
| 2133 | Diethoxydimethylsilane | 43.10 | 40.90 | 40.30 | 2.80 |
| 2134 | 1-s-Butylimidazole | 64.60 | 63.20 | 61.80 | 2.80 |
| 2135 | (E) 10-Tetradecen-1-ol | 101.50 | 98.80 | 98.70 | 2.80 |
| 2136 | (E) 3-Octadecen-1-ol | 120.00 | 117.50 | 117.20 | 2.80 |
| 2137 | 1-Ethoxy-1,1,2,2-tetrafluoroethane | 33.00 | 30.40 | 30.20 | 2.80 |
| 2138 | 3-Methoxythiophene | 48.50 | 46.50 | 45.70 | 2.80 |
| 2139 | Cumyl bromide | 58.00 | 55.20 |  | 2.80 |
| 2140 | Cyclododecane | 63.00 | 65.70 | 65.80 | -2.80 |
| 2141 | Dimethyl oxalate | 52.50 | 49.80 | 49.70 | 2.80 |
| 2142 | Methylpelargonate | 62.00 | 65.00 | 64.80 | -2.80 |
| 2143 | 2,2,4,5-Tetramethylhexane | 44.40 | 41.60 | 41.60 | 2.80 |
| 2144 | 1,3-Dibromobenzene | 54.90 | 52.10 | 52.10 | 2.80 |
| 2145 | N,N,N',N'-Tetramethyl-1,3-butanediamine | 47.90 | 45.60 | 45.10 | 2.80 |
| 2146 | 4-Methylcyclohexyl isobutyrate | 59.70 | 62.50 | 62.50 | -2.80 |
| 2147 | Mustard gas | 59.80 | 57.40 | 57.00 | 2.80 |
| 2148 | cis-2-Octene | 40.20 | 42.90 | 43.00 | -2.80 |
| 2149 | 3-Methylbutyl trichloroacetate | 57.70 | 60.10 | 60.50 | -2.80 |
| 2150 | trans-2-Octene | 40.20 | 42.90 | 43.00 | -2.80 |
| 2151 | Methylvalerate | 43.70 | 46.30 | 46.50 | -2.80 |
| 2152 | (E) 3-Hexadecen-1-ol | 110.80 | 108.10 | 108.00 | 2.80 |
| 2153 | (E) 6-Dodecenal | 67.70 | 70.50 | 70.50 | -2.80 |
| 2154 | 2,6-Di-t-butylpyridine | 57.30 | 59.60 | 60.10 | -2.80 |
| 2155 | Azulene | 52.80 | 55.60 | 55.60 | -2.80 |
| 2156 | Bis(nonafluorobutyl) ether | 40.30 | 43.00 | 43.10 | -2.80 |
| 2157 | t-Butylmethylamine | 32.30 | 34.70 | 35.10 | -2.80 |
| 2158 | (Z) 7-Tetradecenyl acetate | 88.40 | 91.20 | 91.20 | -2.80 |
| 2159 | Cyclohexylamine | 43.90 | 46.90 | 46.70 | -2.80 |
| 2160 | 1-Methoxyheptane | 46.90 | 49.70 | 49.70 | -2.80 |
| 2161 | Trifluorothioacetic acid S-(2-fluoroethyl) ester | 41.40 | 42.90 | 44.20 | -2.80 |
| 2162 | 1,1,2-Trimethylsilacyclobutane | 36.00 | 36.70 | 38.80 | -2.80 |
| 2163 | Ethylnitrate | 36.00 | 38.70 | 38.80 | -2.80 |
| 2164 | cis-3-Heptene | 35.60 | 38.30 | 38.40 | -2.80 |
| 2165 | Isobutyl trichloroacetate | 53.10 | 55.40 | 55.90 | -2.80 |
| 2166 | Methylenecyclohexane | 36.10 | 38.90 | 38.90 | -2.80 |
| 2167 | Propylcyclopentane | 41.10 | 44.00 | 43.90 | -2.80 |
| 2168 | trans-3-Heptene | 35.60 | 38.30 | 38.40 | -2.80 |
| 2169 | (Z) 6-Hexadecenyl acetate | 97.80 | 100.50 | 100.60 | -2.80 |
| 2170 | 2-Isopropyl-p-cresol | 77.74 | 74.90 | 74.90 | 2.84 |
| 2171 | 1,I-Dimethylazoxyethane | 45.94 | 43.10 |  | 2.84 |
| 2172 | Methylamine | 23.85 | 26.60 | 26.70 | -2.85 |
| 2173 | Perylene | 118.50 | 116.20 | 115.60 | 2.90 |
| 2174 | Diisopropyl succinate | 70.80 | 68.00 | 67.90 | 2.90 |
| 2175 | Propylenediamine | 46.70 | 44.00 | 43.80 | 2.90 |
| 2176 | 2,2-Diethoxypropane | 43.20 | 39.90 | 40.30 | 2.90 |
| 2177 | 3-Methyl-2-pentanol | 58.20 | 55.60 | 55.30 | 2.90 |
| 2178 | Butyl t-butyl ether | 43.20 | 40.40 | 40.30 | 2.90 |
| 2179 | (E) 2-Tetradecenal | 82.60 | 79.90 | 79.70 | 2.90 |
| 2180 | 1,1,4-Trimethylcyclohexane | 45.60 | 42.80 | 42.70 | 2.90 |
| 2181 | 1,3-Dioxane | 39.10 | 36.60 | 36.20 | 2.90 |
| 2182 | 1-Pentyl-2-methylimidazole | 71.40 | 73.50 | 74.30 | -2.90 |
| 2183 | 2-Naphthoic acid | 101.10 | 97.90 | 98.20 | 2.90 |
| 2184 | 2-Propynyl-(2E,4E)-3,7,11-trimethyl-2,4-dodecadienoate | 98.10 | 95.20 | 95.20 | 2.90 |
| 2185 | Bromo-2-chlorobenzene | 52.00 | 49.10 | 49.10 | 2.90 |
| 2186 | 1-Bromo-2-fluorobenzene | 47.00 | 44.10 | 44.10 | 2.90 |
| 2187 | 1-Chloro-2-fluorobenzene | 44.00 | 41.00 | 41.10 | 2.90 |
| 2188 | Perfluoropentane | 26.60 | 28.80 | 29.50 | -2.90 |
| 2189 | 1-Methoxyhexane | 42.10 | 45.00 | 45.00 | -2.90 |
| 2190 | 2,7-Dichlorodibenzo-p-dioxin | 76.40 | 73.20 | 73.50 | 2.90 |
| 2191 | 2-Nitropropane | 43.90 | 41.00 |  | 2.90 |
| 2192 | 3,4-Dimethyl-1-hexene | 38.90 | 36.20 | 36.00 | 2.90 |
| 2193 | Bis(2-methylphenoxy)dimethylsilane | 63.60 | 66.20 | 66.50 | -2.90 |
| 2194 | Cyclopropanecarbonitrile | 41.90 | 38.90 | 39.00 | 2.90 |
| 2195 | Dibutyl succinate | 79.10 | 82.20 | 82.00 | -2.90 |
| 2196 | N-(1-Methylethyl)ethanamide | 66.40 | 63.90 | 63.50 | 2.90 |
| 2197 | n-Butyl methyl ether | 32.60 | 35.70 | 35.50 | -2.90 |
| 2198 | 2,6-dimethylheptane-3,5-dione | 57.70 | 59.80 | 60.60 | -2.90 |
| 2199 | 3,5,7-Trioxanonane | 44.70 | 47.60 | 47.60 | -2.90 |
| 2200 | Benzocyclobutane | 43.70 | 46.50 | 46.60 | -2.90 |
| 2201 | trans-3-Octene | 40.20 | 42.90 | 43.10 | -2.90 |
| 2202 | trans-Perfluorobicyclo[4,3.0]nonane | 45.20 | 47.70 | 48.10 | -2.90 |
| 2203 | (Z) 5-Tetradecenyl acetate | 88.30 | 91.20 | 91.20 | -2.90 |
| 2204 | Fluorodiphenylmethane | 69.80 | 71.50 | 72.70 | -2.90 |
| 2205 | Levulinic acid | 70.30 | 72.80 | 73.20 | -2.90 |
| 2206 | Bis(2-hydroxyethyl) sulfide | 86.80 | 89.80 | 89.70 | -2.90 |
| 2207 | 2-Vinyl-5-norbornene | 42.30 | 45.20 | 45.20 | -2.90 |
| 2208 | Ethyl 2-thiopheneacetate | 61.80 | 64.30 | 64.70 | -2.90 |
| 2209 | Isobutyl 3-chlorobutyrate | 52.30 | 55.40 | 55.20 | -2.90 |
| 2210 | n-Heptylformate | 53.80 | 56.40 | 56.70 | -2.90 |
| 2211 | Perfluorononane | 45.30 | 47.80 | 48.20 | -2.90 |
| 2212 | 1,3-Benzodioxole | 41.40 | 44.10 | 44.30 | -2.90 |
| 2213 | 2,3,4,6-Tetrafluoroaniline | 50.40 | 53.50 | 53.30 | -2.90 |
| 2214 | 2,6-Dimethyl-2-heptyl propanoate | 59.40 | 62.30 | 62.30 | -2.90 |
| 2215 | Trimethyl orthobenzoate | 59.90 | 62.10 | 62.80 | -2.90 |
| 2216 | Iodobenzene | 48.50 | 51.30 | 51.40 | -2.90 |
| 2217 | (Z) 4-Hexadecenyl acetate | 97.70 | 100.50 | 100.60 | -2.90 |
| 2218 | Decylbenzene | 78.20 | 81.10 | 81.10 | -2.90 |
| 2219 | n-Propylnitrate | 40.58 | 43.30 | 43.50 | -2.92 |
| 2220 | trans-Decahydroquinoline | 53.28 | 56.50 | 56.20 | -2.92 |
| 2221 | Pyrocatechol trimethylene ether | 55.56 | 52.90 | 52.60 | 2.96 |
| 2222 | Chlorfenvinphos | 96.34 | 99.30 |  | -2.96 |
| 2223 | (S)-Ethyl-(2E,4E)-3,7,11-trimethyl-2,4-dodecadienoate | 92.40 | 89.80 | 89.40 | 3.00 |
| 2224 | (Z) 10-Dodecen-1-ol | 92.40 | 89.40 | 89.40 | 3.00 |
| 2225 | Muscalure | 114.40 | 111.90 | 111.40 | 3.00 |
| 2226 | 1,1,1-Trichloro-3,3,3-trifluoropropane | 36.80 | 33.70 | 33.80 | 3.00 |
| 2227 | (Z) 10-Pentadecen-1-ol | 106.20 | 103.40 | 103.20 | 3.00 |
| 2228 | (Z) 11-Tetradecen-1-ol | 101.70 | 98.80 | 98.70 | 3.00 |
| 2229 | Anabasine | 75.20 | 71.90 | 72.20 | 3.00 |
| 2230 | 1,1,1-Trimethoxyethane | 39.20 | 36.70 | 36.20 | 3.00 |
| 2231 | 2,2,3-Trimethylpentane | 37.70 | 34.80 | 34.70 | 3.00 |
| 2232 | 3-Acetylthiophene | 55.20 | 52.50 | 52.20 | 3.00 |
| 2233 | 3-Chloropentane | 36.70 | 33.70 | 33.70 | 3.00 |
| 2234 | Diethyl malonate | 61.70 | 58.80 | 58.70 | 3.00 |
| 2235 | N,N-Dimethyl-p-toluidine | 57.20 | 55.70 | 54.20 | 3.00 |
| 2236 | 3,3-Dichloro-1,1,1,3-tetrafluoropropane | 31.90 | 29.60 | 28.90 | 3.00 |
| 2237 | 2,3,3-Trimethylpentane | 37.60 | 34.80 | 34.60 | 3.00 |
| 2238 | isobutyl 2-chloropropionate | 53.60 | 50.70 | 50.60 | 3.00 |
| 2239 | 2-Chloropropane | 27.60 | 24.40 | 24.60 | 3.00 |
| 2240 | (E) 6-Hexadecen-1-ol | 111.00 | 108.10 | 108.00 | 3.00 |
| 2241 | (Z) 10-Hexadecen-1-ol | 111.00 | 108.10 | 108.00 | 3.00 |
| 2242 | (Z) 3-Octadecen-1-ol | 120.50 | 117.50 | 117.50 | 3.00 |
| 2243 | 1-Nonadecene | 95.00 | 92.30 | 92.00 | 3.00 |
| 2244 | 2-Carene | 48.50 | 45.80 | 45.50 | 3.00 |
| 2245 | Dimethyl malonate | 57.50 | 54.50 | 54.50 | 3.00 |
| 2246 | Ibuprofen | 94.00 | 96.90 | 97.00 | -3.00 |
| 2247 | 1-Butene | 20.10 | 23.30 | 23.10 | -3.00 |
| 2248 | 1-Methylcyclopentene | 32.60 | 35.60 | 35.60 | -3.00 |
| 2249 | 2,5-Di-t-butylfuran | 56.10 | 58.90 | 59.10 | -3.00 |
| 2250 | cis-Hydrindan | 46.10 | 49.20 | 49.10 | -3.00 |
| 2251 | Amylbenzene | 55.10 | 58.10 | 58.10 | -3.00 |
| 2252 | trans-3,4,4-Trimethyl-2-pentene | 38.90 | 35.60 | 35.90 | 3.00 |
| 2253 | Pentane | 26.40 | 29.20 | 29.40 | -3.00 |
| 2254 | Methyldibutylsilane | 36.20 | 39.10 | 39.20 | -3.00 |
| 2255 | cis-Stilbene | 71.20 | 74.70 | 74.20 | -3.00 |
| 2256 | N-Cyclohexyl-2,4,6-trimethylbenzaldehyde imine | 78.70 | 82.70 | 81.70 | -3.00 |
| 2257 | cis-3-Hexen-1-ol | 58.30 | 61.40 | 61.30 | -3.00 |
| 2258 | Di-s-butylamine | 41.30 | 44.30 | 44.30 | -3.00 |
| 2259 | trans-1,2-Dimethylcyclohexane | 38.30 | 41.20 | 41.30 | -3.00 |
| 2260 | 1,1,1,2,2,3,3,4,4-Nonafluorodecane | 49.40 | 52.20 | 52.40 | -3.00 |
| 2261 | 4-Methylcyclohexyl pivalate | 60.90 | 63.90 | 63.90 | -3.00 |
| 2262 | Propyl 3-chloropropanoate | 51.90 | 54.80 | 54.90 | -3.00 |
| 2263 | 1-Decyl-1-undecylcyclohexane | 133.60 | 131.20 | 130.60 | 3.00 |
| 2264 | Methyl hexacosanoate | 147.10 | 144.40 | 144.10 | 3.00 |
| 2265 | 7,7-Dihexyltridecane | 115.30 | 118.50 | 118.30 | -3.00 |
| 2266 | 9-Fluorenylmethanol | 97.80 | 100.80 | 100.80 | -3.00 |
| 2267 | O,O'-Dibutyl dithiophosphate | 81.80 | 84.80 |  | -3.00 |
| 2268 | 2,5-Dimethyl-2,4-hexadiene | 43.92 | 40.90 | 40.90 | 3.02 |
| 2269 | (E) 11-Tridecen-1-ol | 97.20 | 94.10 | 94.10 | 3.10 |
| 2270 | (E) 11-Pentadecen-1-ol | 106.50 | 103.40 | 103.40 | 3.10 |
| 2271 | 4-Fluoroacetophenone | 56.50 | 53.60 | 53.40 | 3.10 |
| 2272 | 3-Ethyl-3-methyl-1-pentene | 38.90 | 35.70 | 35.80 | 3.10 |
| 2273 | Triethylamine | 35.40 | 32.30 | 32.30 | 3.10 |
| 2274 | 2-t-Butylaniline | 62.70 | 66.10 | 65.80 | -3.10 |
| 2275 | 3,3-Diethyl-2-methylpentane | 47.30 | 44.30 | 44.20 | 3.10 |
| 2276 | (E) 11-Tetradecen-1-ol | 101.80 | 98.80 | 98.70 | 3.10 |
| 2277 | (Z) 11-Pentadecen-1-ol | 106.30 | 103.40 | 103.20 | 3.10 |
| 2278 | 3-Methyl-4-nitrophenol | 85.80 | 82.20 | 82.70 | 3.10 |
| 2279 | (E) 8-Hexadecen-1-ol | 111.10 | 108.10 | 108.00 | 3.10 |
| 2280 | (Z) 2-Ethylhexyl 4-methoxycinnamate | 98.90 | 102.00 | 102.00 | -3.10 |
| 2281 | 1,2,3,4-Tetrachlorodibenzo-p-dioxin | 85.60 | 82.70 | 82.50 | 3.10 |
| 2282 | 1-(2-Methoxyphenyl)-1H-imidazole | 83.10 | 80.10 | 80.00 | 3.10 |
| 2283 | 1-Bromonaphthalene | 63.90 | 66.90 | 67.00 | -3.10 |
| 2284 | 3-Methyl-2-thiophenecarbonitrile | 54.40 | 57.50 | 57.50 | -3.10 |
| 2285 | 3-Methylbenzophenone | 85.60 | 82.90 | 82.50 | 3.10 |
| 2286 | Trichlorotoluene | 57.60 | 54.50 | 54.50 | 3.10 |
| 2287 | d-Verbenone | 58.60 | 55.40 | 55.50 | 3.10 |
| 2288 | Di-n-octylamine | 92.60 | 89.40 | 89.50 | 3.10 |
| 2289 | Methyl 2,4,4-trimethylpentanoate | 48.40 | 51.60 | 51.50 | -3.10 |
| 2290 | Trimethoxy((phenylthio)methyl)silane | 56.40 | 58.80 | 59.50 | -3.10 |
| 2291 | Cyclohexyl pivalate | 59.00 | 62.00 | 62.10 | -3.10 |
| 2292 | Ethyl cis-2-pentenoate | 46.00 | 49.20 | 49.10 | -3.10 |
| 2293 | (Z) 6-Tetradecenyl acetate | 88.10 | 91.20 | 91.20 | -3.10 |
| 2294 | 1,1,1-Trifluoro-2,2-diphenylethane | 69.10 | 72.10 | 72.20 | -3.10 |
| 2295 | 1-Pentylimidazole | 69.10 | 71.80 | 72.20 | -3.10 |
| 2296 | 2,2,3-Trichloro-1,1,1,3,4,4,4-heptafluorobutane | 35.60 | 36.60 | 38.70 | -3.10 |
| 2297 | cis-1,3-Dimethylcyclohexane | 38.10 | 41.20 | 41.20 | -3.10 |
| 2298 | Hydroxyacetic acid | 74.60 | 77.10 | 77.70 | -3.10 |
| 2299 | 2,2,6-Trimethylheptane-3,5-dione | 57.70 | 61.30 | 60.80 | -3.10 |
| 2300 | Butyl heptyl ether | 58.20 | 61.20 | 61.30 | -3.10 |
| 2301 | Diisopropenyldiacetylene | 50.20 | 53.10 | 53.30 | -3.10 |
| 2302 | Ethylcyclobutane | 31.20 | 34.40 | 34.30 | -3.10 |
| 2303 | Quinoxaline | 58.70 | 61.60 | 61.80 | -3.10 |
| 2304 | 1,1-Diphenylpropane | 72.80 | 75.80 | 75.90 | -3.10 |
| 2305 | 2-Phenyltoluene | 65.30 | 68.40 | 68.40 | -3.10 |
| 2306 | Cyclohexadecane | 81.80 | 84.80 | 84.90 | -3.10 |
| 2307 | Hexacosane | 131.70 | 129.20 | 128.60 | 3.10 |
| 2308 | Acetylmesitylene | 62.30 | 64.90 | 65.40 | -3.10 |
| 2309 | Dibutyldiazene | 49.30 | 51.60 | 52.40 | -3.10 |
| 2310 | Ethylpyrazine | 48.80 | 51.30 | 51.90 | -3.10 |
| 2311 | Isobutyl 2-chlorobutyrate | 52.30 | 55.40 | 55.40 | -3.10 |
| 2312 | (Z) 3-Decenyl acetate | 69.50 | 72.50 | 72.60 | -3.10 |
| 2313 | Dibutyl adipate | 88.50 | 91.50 | 91.60 | -3.10 |
| 2314 | n-Butylmethylsulfone | 76.00 | 78.80 | 79.10 | -3.10 |
| 2315 | 2,3,3',4,4',5'-Hexabromodiphenyl ether | 114.06 | 111.00 | 110.90 | 3.16 |
| 2316 | (E) 9-Hexadecen-1-ol | 111.30 | 108.10 | 108.10 | 3.20 |
| 2317 | (Z) 11-Tridecen-1-ol | 97.10 | 94.10 | 93.90 | 3.20 |
| 2318 | Diphenyl ether | 67.10 | 63.90 | 63.90 | 3.20 |
| 2319 | 1,1-Dinitropropane | 62.50 | 59.30 |  | 3.20 |
| 2320 | 2-Cyclohexene-1-one | 49.50 | 46.30 | 46.30 | 3.20 |
| 2321 | (E) 11-Octadecen-1-ol | 120.40 | 117.50 | 117.20 | 3.20 |
| 2322 | cis-3,4,4-Trimethyl-2-pentene | 38.90 | 35.60 | 35.70 | 3.20 |
| 2323 | 1,5-Pentanedithiol | 59.30 | 56.60 | 56.10 | 3.20 |
| 2324 | 1-Bromo-4-chlorobenzene | 52.30 | 49.10 | 49.10 | 3.20 |
| 2325 | 1-Methylpiperazine | 45.80 | 42.40 | 42.60 | 3.20 |
| 2326 | Bicyclopropyl | 31.70 | 34.90 | 34.90 | -3.20 |
| 2327 | 1-Methylfluorene | 77.20 | 74.30 | 74.00 | 3.20 |
| 2328 | Hydroxycitronellal | 75.30 | 78.20 | 78.50 | -3.20 |
| 2329 | Methyl heptacosanoate | 152.20 | 149.10 | 149.00 | 3.20 |
| 2330 | 1,1,1,2,3,3-Hexafluoro-3-(2,2,3,3-tetrafluoropropoxy)propane | 43.20 | 41.00 | 40.00 | 3.20 |
| 2331 | 1-Chloro-2-methyl-1-propene | 33.20 | 30.40 | 30.00 | 3.20 |
| 2332 | 2,3-Dimethyl-3-ethylpentane | 42.70 | 39.60 | 39.50 | 3.20 |
| 2333 | Ethyl methyl ketone oxime | 57.70 | 54.50 |  | 3.20 |
| 2334 | Isopentyl 2-chloropropanoate | 52.30 | 55.40 | 55.50 | -3.20 |
| 2335 | 3-Methylcyclohexyl isobutyrate | 59.30 | 62.50 | 62.50 | -3.20 |
| 2336 | 4-Chloroacetophenone | 62.20 | 58.50 | 59.00 | 3.20 |
| 2337 | 4-Ethylpyridine | 46.30 | 49.30 | 49.50 | -3.20 |
| 2338 | 4-Vinylcyclohexene | 39.80 | 42.90 | 43.00 | -3.20 |
| 2339 | N-Ethylaniline | 58.30 | 62.40 | 61.50 | -3.20 |
| 2340 | trans-2-Butene | 21.30 | 24.50 | 24.50 | -3.20 |
| 2341 | Vinyl acetate | 37.20 | 34.10 | 34.00 | 3.20 |
| 2342 | Dipropyldiazene | 39.90 | 42.30 | 43.10 | -3.20 |
| 2343 | Ethyldiisopropylsilane | 38.10 | 36.20 | 34.90 | 3.20 |
| 2344 | 1,2,7,8-Tetrachlorodibenzofuran | 81.50 | 84.40 | 84.70 | -3.20 |
| 2345 | 3-Methylcyclopentene | 31.00 | 34.20 | 34.20 | -3.20 |
| 2346 | 4-Iodophenol | 73.00 | 75.70 | 76.20 | -3.20 |
| 2347 | Cyclohexyl butanoate | 60.00 | 63.30 | 63.20 | -3.20 |
| 2348 | Dicyclohexylsulfide | 69.00 | 72.00 | 72.20 | -3.20 |
| 2349 | Dimethyl sulfate | 48.50 | 51.70 |  | -3.20 |
| 2350 | Butyl hexyl ether | 53.20 | 56.50 | 56.40 | -3.20 |
| 2351 | Pervitin | 58.70 | 61.80 | 61.90 | -3.20 |
| 2352 | n-Octylformate | 58.20 | 61.10 | 61.40 | -3.20 |
| 2353 | 1,1-Diphenylbutane | 77.20 | 80.40 | 80.40 | -3.20 |
| 2354 | 2,4'-Dibromodiphenyl ether | 76.40 | 79.60 | 79.60 | -3.20 |
| 2355 | 4,5-Methylenephenanthrene | 83.40 | 86.60 | 86.60 | -3.20 |
| 2356 | 1,2,3,4,7,8-Hexachlorodibenzo-p-dioxin | 89.10 | 92.30 | 92.30 | -3.20 |
| 2357 | 2-(Biphen-3-yl)naphthalene | 118.60 | 115.80 | 115.30 | 3.30 |
| 2358 | Tetracene | 106.20 | 103.00 | 102.90 | 3.30 |
| 2359 | 2,4,6-Collidine | 50.20 | 47.20 | 46.90 | 3.30 |
| 2360 | 3-Methyl-2-isopropyl-1-butene | 38.10 | 34.80 | 34.80 | 3.30 |
| 2361 | 3,3-Dimethyl-1-butanol | 58.00 | 54.70 | 54.70 | 3.30 |
| 2362 | 1,1-Dimethyl-4-penten-2-yn-1-ylhydroperoxide | 63.50 | 61.20 | 60.20 | 3.30 |
| 2363 | Glycidyl isopropyl ether | 43.50 | 40.60 | 40.20 | 3.30 |
| 2364 | 1,2-Dimethylimidazole | 58.90 | 57.10 | 55.60 | 3.30 |
| 2365 | 1,4-Butanedithiol | 54.90 | 51.90 | 51.60 | 3.30 |
| 2366 | (1-Chloroethyl)benzene | 52.80 | 49.80 | 49.50 | 3.30 |
| 2367 | 2,2,3,4-Tetramethylpentane | 40.80 | 36.80 | 37.50 | 3.30 |
| 2368 | 2,4-Dimethyl-3-ethylpentane | 43.30 | 40.10 | 40.00 | 3.30 |
| 2369 | 2-Propyl-1,3-dioxolane | 45.30 | 42.30 | 42.00 | 3.30 |
| 2370 | 3-Ethyl-2,2,4-trimethylpentane | 44.80 | 41.60 | 41.50 | 3.30 |
| 2371 | cis-4-Octene | 39.70 | 42.90 | 43.00 | -3.30 |
| 2372 | Methyl pivalate | 37.70 | 40.40 | 41.00 | -3.30 |
| 2373 | Pentyl t-butyl ether | 48.30 | 45.00 | 45.00 | 3.30 |
| 2374 | s-Butyl-t-butylether | 41.30 | 38.00 | 38.00 | 3.30 |
| 2375 | (Z) 11-Hexadecen-1-ol | 111.30 | 108.10 | 108.00 | 3.30 |
| 2376 | (Z) 13-Octadecen-1-ol | 120.80 | 117.50 | 117.50 | 3.30 |
| 2377 | Benz[a]anthracene | 105.80 | 103.00 | 102.50 | 3.30 |
| 2378 | 3,3,3-Trifluoropropene | 22.00 | 18.80 | 18.70 | 3.30 |
| 2379 | Cyclohexylformate | 49.30 | 52.50 | 52.60 | -3.30 |
| 2380 | Vinyltripropoxysilane | 52.30 | 55.60 |  | -3.30 |
| 2381 | 3-Methylpentyl t-octyl ether | 56.40 | 59.70 | 59.70 | -3.30 |
| 2382 | cis 3-Hexenyl butyrate | 59.90 | 63.20 | 63.20 | -3.30 |
| 2383 | Isopropylcyclopentane | 37.90 | 41.20 | 41.20 | -3.30 |
| 2384 | Perfluoro-n-octane | 39.90 | 43.00 | 43.20 | -3.30 |
| 2385 | Cycloheptane | 38.50 | 41.90 | 41.80 | -3.30 |
| 2386 | Isobutylcyclohexane | 47.50 | 50.70 | 50.80 | -3.30 |
| 2387 | (Z) 4-Dodecenyl acetate | 78.60 | 81.90 | 81.90 | -3.30 |
| 2388 | 2,4-Xylenol | 64.60 | 68.30 | 67.90 | -3.30 |
| 2389 | (Z) 4-Tetradecenyl acetate | 87.80 | 91.20 | 91.10 | -3.30 |
| 2390 | Methyl caprate | 66.30 | 69.70 | 69.60 | -3.30 |
| 2391 | beta-Phenylethylacetate | 61.30 | 64.50 | 64.60 | -3.30 |
| 2392 | Coronene | 139.50 | 142.60 | 142.80 | -3.30 |
| 2393 | 1,3-Dioxolane | 35.23 | 31.90 | 31.90 | 3.33 |
| 2394 | (E) 4-Hexadecen-1-ol | 111.50 | 108.10 | 108.10 | 3.40 |
| 2395 | 1-Ethyl-3-piperidinol | 68.50 | 65.10 | 65.10 | 3.40 |
| 2396 | n-Octanoic acid | 81.00 | 77.90 | 77.60 | 3.40 |
| 2397 | 1,3-Di-t-butyl-5-methylbenzene | 63.30 | 59.90 | 59.90 | 3.40 |
| 2398 | 1,5-Pentanediol | 86.80 | 83.30 | 83.40 | 3.40 |
| 2399 | 2',5'-Dichloroacetophenone | 66.30 | 63.30 | 62.90 | 3.40 |
| 2400 | Phenacetin | 94.80 | 91.20 | 91.40 | 3.40 |
| 2401 | 2-Methylanisole | 50.20 | 47.00 | 46.80 | 3.40 |
| 2402 | 2,2,3,3-Tetrafluoro-1-propanol | 53.60 | 50.80 | 50.20 | 3.40 |
| 2403 | 1,5-Diazabicyclo[4.3.0]non-5-ene | 62.60 | 60.40 | 59.20 | 3.40 |
| 2404 | Ethyl 2-cyanopropanoate | 58.60 | 55.40 | 55.20 | 3.40 |
| 2405 | 2,3-Dihydro-1H-indole | 60.90 | 64.60 | 64.30 | -3.40 |
| 2406 | Nitroethane | 41.60 | 38.70 | 38.20 | 3.40 |
| 2407 | 1-Chloro-3-isocyanatobenzene | 54.00 | 51.00 | 50.60 | 3.40 |
| 2408 | Cyclohexene | 33.50 | 36.90 | 36.90 | -3.40 |
| 2409 | (E) 5-Hexadecen-1-ol | 111.40 | 108.10 | 108.00 | 3.40 |
| 2410 | (E) 7-Hexadecen-1-ol | 111.40 | 108.10 | 108.00 | 3.40 |
| 2411 | 1,3-Butyleneglycol | 72.60 | 76.20 | 76.00 | -3.40 |
| 2412 | 2,3,4,7,8-Pentachlorodibenzofuran | 85.60 | 89.20 | 89.00 | -3.40 |
| 2413 | 2-Bromoacetophenone | 64.90 | 61.60 | 61.50 | 3.40 |
| 2414 | 4-Methylbenzoic acid | 82.40 | 79.00 | 79.00 | 3.40 |
| 2415 | Dodecahydro-N-ethylcarbazole | 68.40 | 66.00 | 65.00 | 3.40 |
| 2416 | 2-Methylpyrazine | 43.70 | 46.60 | 47.10 | -3.40 |
| 2417 | Acetylacetone | 43.20 | 46.60 | 46.60 | -3.40 |
| 2418 | Dimethyl sulfite | 40.20 | 43.10 | 43.60 | -3.40 |
| 2419 | 1-(2-Methylphenyl)-1H-imidazole | 73.80 | 77.00 | 77.20 | -3.40 |
| 2420 | 1-Naphthylamine | 73.30 | 76.90 | 76.70 | -3.40 |
| 2421 | 2-(Dimethylamino)acetophenone | 69.70 | 66.50 | 66.30 | 3.40 |
| 2422 | Ethan-1,2-diol | 65.80 | 69.30 | 69.20 | -3.40 |
| 2423 | Acetylchloride | 23.80 | 27.10 | 27.20 | -3.40 |
| 2424 | Cyclooctane | 43.30 | 46.70 | 46.70 | -3.40 |
| 2425 | Oxonan-2-one | 52.80 | 56.30 | 56.20 | -3.40 |
| 2426 | Methylcaproate | 47.40 | 51.00 | 50.80 | -3.40 |
| 2427 | Neral | 55.90 | 59.20 | 59.30 | -3.40 |
| 2428 | 2-Methylpropyl 4-pentenoate | 51.00 | 54.50 | 54.40 | -3.40 |
| 2429 | N,N-Dimethylbenzylamine | 49.50 | 52.90 | 52.90 | -3.40 |
| 2430 | Bis(2,2,3,3-tetrafluoropropyl)carbonate | 66.50 | 68.90 | 69.90 | -3.40 |
| 2431 | Butylcyclohexane | 50.00 | 53.50 | 53.40 | -3.40 |
| 2432 | Parathion | 89.92 | 92.20 | 93.40 | -3.48 |
| 2433 | 3,6-Dimethyl-3,6-diethyl-1,2,4,5-tetraoxacyclohexane | 45.09 | 41.50 | 41.60 | 3.49 |
| 2434 | (Dimethylamino)acetonitrile | 45.40 | 41.80 | 41.90 | 3.50 |
| 2435 | 3-Ethyl-o-xylene | 54.90 | 51.60 | 51.40 | 3.50 |
| 2436 | 1,3,6-Trioxocane | 48.80 | 45.60 | 45.30 | 3.50 |
| 2437 | 2-Methyl-2-propyl-1,3-dioxolane | 46.30 | 43.10 | 42.80 | 3.50 |
| 2438 | (Z) 12-Pentadecen-1-ol | 106.70 | 103.40 | 103.20 | 3.50 |
| 2439 | 3,3',4,4'-Tetrabromodiphenyl ether | 98.70 | 95.30 | 95.20 | 3.50 |
| 2440 | 6-Methylquinoline | 67.70 | 64.10 | 64.20 | 3.50 |
| 2441 | Methylundecylate | 70.80 | 74.30 | 74.30 | -3.50 |
| 2442 | 2-Bromopropane | 30.20 | 26.90 | 26.70 | 3.50 |
| 2443 | 3-Ethyl-3-(chloromethyl)oxetane | 49.70 | 46.10 | 46.20 | 3.50 |
| 2444 | Hexyl t-butyl ether | 53.20 | 49.70 | 49.70 | 3.50 |
| 2445 | 3,3-Dimethyloxetane | 33.90 | 30.40 | 30.40 | 3.50 |
| 2446 | 3-(Methylamino)propylamine | 53.10 | 49.90 | 49.60 | 3.50 |
| 2447 | 3-Furancarboxaldehyde | 48.10 | 43.70 | 44.60 | 3.50 |
| 2448 | (E) 10-Hexadecen-1-ol | 111.50 | 108.10 | 108.00 | 3.50 |
| 2449 | (Z) 4-Decenyl acetate | 69.00 | 72.50 | 72.50 | -3.50 |
| 2450 | Hexamethyleneglycol | 91.00 | 88.00 | 87.50 | 3.50 |
| 2451 | 1-Eicosene | 100.00 | 96.90 | 96.50 | 3.50 |
| 2452 | 2-Methylcyclohexanone | 44.00 | 47.50 | 47.50 | -3.50 |
| 2453 | 3-Methylcyclohexyl pivalate | 60.50 | 63.90 | 64.00 | -3.50 |
| 2454 | Butyl 2-pentenoate | 55.00 | 58.50 | 58.50 | -3.50 |
| 2455 | 1,1-Dimethoxycyclopentane | 44.50 | 48.20 | 48.00 | -3.50 |
| 2456 | Ethoxyacetonitrile | 46.50 | 42.80 | 43.00 | 3.50 |
| 2457 | Ethylcyclohexane | 40.50 | 44.00 | 44.00 | -3.50 |
| 2458 | Metasystox | 71.00 | 74.40 | 74.50 | -3.50 |
| 2459 | t-Butylhydroperoxide | 48.00 | 45.00 | 44.50 | 3.50 |
| 2460 | Methylpropylether | 27.60 | 31.00 | 31.10 | -3.50 |
| 2461 | 1-Bromoperfluorooctane | 45.60 | 49.10 |  | -3.50 |
| 2462 | 2-Tolunitrile | 54.80 | 58.20 | 58.30 | -3.50 |
| 2463 | 4-(3,6-Dimethylheptan-3-yl)phenol | 89.40 | 93.20 | 92.90 | -3.50 |
| 2464 | Cyclobutane | 23.92 | 27.60 | 27.50 | -3.58 |
| 2465 | Octacosane | 141.90 | 138.70 | 138.30 | 3.60 |
| 2466 | 9-Ethyl-9H-carbazole | 83.90 | 80.30 | 80.30 | 3.60 |
| 2467 | Suberic acid | 116.70 | 113.00 | 113.10 | 3.60 |
| 2468 | (E) 12-Pentadecen-1-ol | 107.00 | 103.40 | 103.40 | 3.60 |
| 2469 | 1,3,6,8-Tetrachlorodibenzo-p-dioxin | 86.50 | 82.70 | 82.90 | 3.60 |
| 2470 | Dioctyl sulfide | 95.00 | 91.30 | 91.40 | 3.60 |
| 2471 | (Diethylamino)acetonitrile | 49.90 | 46.60 | 46.30 | 3.60 |
| 2472 | 2,6-Dimethyl-1-heptene | 45.90 | 42.20 | 42.30 | 3.60 |
| 2473 | Ethoxytrimethylsilane | 38.40 | 34.00 | 34.80 | 3.60 |
| 2474 | Dimethyl t-butyl amine | 34.80 | 31.20 | 31.20 | 3.60 |
| 2475 | Propyl tert-amyl ether | 43.80 | 40.40 | 40.20 | 3.60 |
| 2476 | 2-(Dimethylamino)-2-methyl-1-phenylpropanone | 66.70 | 70.20 | 70.30 | -3.60 |
| 2477 | Dibenzo[b,d]thiophene | 78.30 | 75.80 | 74.70 | 3.60 |
| 2478 | Methylchloroacetate | 46.70 | 43.40 | 43.10 | 3.60 |
| 2479 | Methyloxiran | 27.90 | 24.60 | 24.30 | 3.60 |
| 2480 | 3-Thiopheneacetonitrile | 61.10 | 57.90 | 57.50 | 3.60 |
| 2481 | Bicyclopentyl | 50.40 | 53.90 | 54.00 | -3.60 |
| 2482 | Isopropyl t-amyl ether | 41.60 | 38.00 | 38.00 | 3.60 |
| 2483 | 2-Isopropyl-5-methylphenol | 70.90 | 74.90 | 74.50 | -3.60 |
| 2484 | Triphenylene | 106.10 | 103.00 | 102.50 | 3.60 |
| 2485 | Di-n-octyl phthalate | 135.90 | 139.30 | 139.50 | -3.60 |
| 2486 | 2,3,4-Trimethyl-1-pentene | 38.50 | 34.80 | 34.90 | 3.60 |
| 2487 | 2-Methylpropyl 3-Pentenoate | 52.00 | 55.80 | 55.60 | -3.60 |
| 2488 | 1,2,3,6,7,8-Hexachlorodibenzo-p-dioxin | 88.10 | 92.30 | 91.70 | -3.60 |
| 2489 | 1-Hexylimidazole | 73.10 | 76.40 | 76.70 | -3.60 |
| 2490 | Phenethyl propionate | 65.60 | 69.20 | 69.20 | -3.60 |
| 2491 | Propylcyclohexane | 45.10 | 48.70 | 48.70 | -3.60 |
| 2492 | trans-1,4-Dimethylcyclohexane | 37.60 | 41.20 | 41.20 | -3.60 |
| 2493 | 1-Methylcyclohexyl isobutyrate | 57.20 | 60.80 | 60.80 | -3.60 |
| 2494 | Nitrocyclohexane | 54.70 | 58.30 |  | -3.60 |
| 2495 | Perfluoro-2,4-dimethyl-3-ethyl-2-pentene | 38.70 | 41.60 | 42.30 | -3.60 |
| 2496 | 1,2,3,4-Tetrahydroquinoline | 65.30 | 69.30 | 68.90 | -3.60 |
| 2497 | 5-Ethylidene-2-norbornene | 44.30 | 47.80 | 47.90 | -3.60 |
| 2498 | 1-Methyl-4-benzylbenzene | 69.50 | 73.00 | 73.10 | -3.60 |
| 2499 | 2,2'-Bipyridine | 67.00 | 69.60 | 70.60 | -3.60 |
| 2500 | cis-3-Hexenyl 2-methylbutyrate | 61.50 | 65.10 | 65.10 | -3.60 |
| 2501 | Di-n-propylsulfone | 80.00 | 82.90 | 83.60 | -3.60 |
| 2502 | Triisobutylphosphate | 76.30 | 73.00 | 72.60 | 3.70 |
| 2503 | 2,4-Dimethyl-3-isopropylpentane | 45.60 | 42.10 | 41.90 | 3.70 |
| 2504 | 2-Acetyl-5-methylthiophene | 62.00 | 57.80 | 58.30 | 3.70 |
| 2505 | 3,6-Dithiaoctane | 59.50 | 55.70 | 55.80 | 3.70 |
| 2506 | Chloral | 41.00 | 37.50 | 37.30 | 3.70 |
| 2507 | Oxirane | 25.90 | 22.30 | 22.20 | 3.70 |
| 2508 | 4-t-Butylbiphenyl | 79.90 | 76.20 | 76.20 | 3.70 |
| 2509 | Methyl isonicotinate | 65.40 | 62.20 | 61.70 | 3.70 |
| 2510 | 2-Furanacrylonitrile | 65.20 | 61.70 | 61.50 | 3.70 |
| 2511 | Chrysene | 106.20 | 103.00 | 102.50 | 3.70 |
| 2512 | Di-t-butyl adipate | 74.30 | 77.90 | 78.00 | -3.70 |
| 2513 | Thioxanthene | 77.80 | 80.40 | 81.50 | -3.70 |
| 2514 | 1,1,5-Trimethylhexyl 2-methylpropanoate | 60.80 | 64.20 | 64.50 | -3.70 |
| 2515 | 1-Azepan-1-ylacetone | 62.20 | 58.40 | 58.50 | 3.70 |
| 2516 | 2,3,3,5-Tetramethylhexane | 45.20 | 41.60 | 41.50 | 3.70 |
| 2517 | 3-Methylisoxazole | 39.80 | 42.60 | 43.50 | -3.70 |
| 2518 | (Fluoromethyl)benzene | 46.20 | 43.30 | 42.50 | 3.70 |
| 2519 | Methylnitrite | 22.60 | 25.90 | 26.30 | -3.70 |
| 2520 | (Diethylamino)trimethylsilane | 37.90 | 40.80 | 41.60 | -3.70 |
| 2521 | 1,5-Cyclooctadiene | 43.40 | 46.80 | 47.10 | -3.70 |
| 2522 | N-(2-Methylpropylidene)-butylamine | 42.00 | 44.00 | 45.70 | -3.70 |
| 2523 | s-Butyl butyrate | 47.00 | 50.70 | 50.70 | -3.70 |
| 2524 | N,N-Dimethylaminotrimethylsilane | 33.60 | 35.90 | 37.30 | -3.70 |
| 2525 | 1-Piperidineethanol | 64.20 | 67.50 | 67.90 | -3.70 |
| 2526 | Di-n-propyl phthalate | 88.70 | 92.60 | 92.40 | -3.70 |
| 2527 | Dibutyl glutarate | 83.10 | 86.80 | 86.80 | -3.70 |
| 2528 | Morpholine | 44.45 | 41.00 | 40.70 | 3.75 |
| 2529 | 2,6-Diisopropylphenol | 85.60 | 81.50 | 81.80 | 3.80 |
| 2530 | 1,7-Heptanediol | 96.40 | 92.60 | 92.60 | 3.80 |
| 2531 | Xanthene | 73.40 | 69.90 | 69.60 | 3.80 |
| 2532 | m-Terphenyl | 97.20 | 93.50 | 93.40 | 3.80 |
| 2533 | 2,2,3,5-Tetramethylhexane | 45.20 | 41.60 | 41.40 | 3.80 |
| 2534 | 3-Methyl-1-pentanol | 61.70 | 58.00 | 57.90 | 3.80 |
| 2535 | 2-Phenyl-1,3-dioxolane | 62.10 | 58.30 | 58.30 | 3.80 |
| 2536 | 3-Methylpyridazine | 56.10 | 53.30 | 52.30 | 3.80 |
| 2537 | (E) 12-Tetradecen-1-ol | 102.50 | 98.80 | 98.70 | 3.80 |
| 2538 | (Z) 12-Tetradecen-1-ol | 102.50 | 98.80 | 98.70 | 3.80 |
| 2539 | 1-Benzyl-4-piperidone | 78.00 | 74.50 | 74.20 | 3.80 |
| 2540 | 2,4-Hexadiyne | 42.50 | 38.50 | 38.70 | 3.80 |
| 2541 | 2-Hydroxymethyl-1,3-dioxane | 69.00 | 64.90 | 65.20 | 3.80 |
| 2542 | Pentyl t-amyl ether | 53.50 | 49.70 | 49.70 | 3.80 |
| 2543 | Acetaldehyde | 26.90 | 23.10 | 23.10 | 3.80 |
| 2544 | (1,1,2-Trifluoro-2,2-dichloroethyl)(2,2,3,3-tetrafluoro-1,1,3-trichloropropyl) ether | 50.70 | 53.40 | 54.50 | -3.80 |
| 2545 | 2,2,4-Trimethyl-4-methoxypentane | 45.30 | 41.60 | 41.50 | 3.80 |
| 2546 | 2,4,4-Trimethyl-2-pentene | 39.30 | 35.60 | 35.50 | 3.80 |
| 2547 | 4-Chloroaniline | 62.30 | 59.10 | 58.50 | 3.80 |
| 2548 | Diethyl oxalate | 57.80 | 54.10 | 54.00 | 3.80 |
| 2549 | (E) 11-Hexadecen-1-ol | 111.80 | 108.10 | 108.00 | 3.80 |
| 2550 | (Z) 12-Hexadecen-1-ol | 111.80 | 108.10 | 108.00 | 3.80 |
| 2551 | (Z)-4-Tridecenyl acetate | 82.70 | 86.50 | 86.50 | -3.80 |
| 2552 | 1,2,4,7,8-Pentachlorodibenzofuran | 85.20 | 89.20 | 89.00 | -3.80 |
| 2553 | Dicyclohexyl malonate | 93.70 | 97.80 | 97.50 | -3.80 |
| 2554 | Ethyl methacrylate | 38.30 | 41.90 | 42.10 | -3.80 |
| 2555 | Trifluorothioacetic acid S-(2,2-difluoroethyl) ester | 39.30 | 43.80 | 43.10 | -3.80 |
| 2556 | 2-Cyclopentene-1-carbonitrile | 44.90 | 48.90 | 48.70 | -3.80 |
| 2557 | Benzylethylsulfide | 56.90 | 60.70 | 60.70 | -3.80 |
| 2558 | Methylcyclohexane | 35.40 | 39.20 | 39.20 | -3.80 |
| 2559 | Cyclopentane | 28.50 | 32.40 | 32.30 | -3.80 |
| 2560 | Isopropyl pentanoate | 47.00 | 50.70 | 50.80 | -3.80 |
| 2561 | (1-Ethoxyethyl)benzene | 52.60 | 56.20 | 56.40 | -3.80 |
| 2562 | Norbornene | 35.10 | 39.30 | 38.90 | -3.80 |
| 2563 | 1-Benzylpyrazole | 73.80 | 77.60 |  | -3.80 |
| 2564 | 2,5-Dimethyl-3-hexyne-2,5-diol | 82.80 | 86.50 | 86.60 | -3.80 |
| 2565 | 4,7-Phenanthroline | 80.80 | 84.30 | 84.60 | -3.80 |
| 2566 | 1,3-Dimethylnaphthalene | 69.58 | 65.70 | 65.70 | 3.88 |
| 2567 | Diisobutyl sulfone | 90.00 | 86.80 | 86.10 | 3.90 |
| 2568 | Pimelic acid | 112.00 | 108.30 | 108.10 | 3.90 |
| 2569 | 1-Chlorotridecane | 81.30 | 77.20 | 77.40 | 3.90 |
| 2570 | Methyl Z,Z,Z-11,14,17-eicosatrieneoate | 122.60 | 118.50 | 118.70 | 3.90 |
| 2571 | Methyltris(3-methylphenoxy)silane | 66.90 | 72.40 | 70.80 | -3.90 |
| 2572 | 1,1,1-Trichloroethane | 32.50 | 29.10 | 28.60 | 3.90 |
| 2573 | Dimethoxymethane | 29.60 | 33.70 | 33.50 | -3.90 |
| 2574 | N,N-Dimethyl-2-propylamine | 31.90 | 28.20 | 28.00 | 3.90 |
| 2575 | 1-Nonylimidazole | 85.60 | 90.40 | 89.50 | -3.90 |
| 2576 | 2,3,6-Trifluoroaniline | 50.10 | 53.70 | 54.00 | -3.90 |
| 2577 | Cyclohexane | 33.10 | 37.20 | 37.00 | -3.90 |
| 2578 | Hexaethylethane | 63.90 | 60.10 | 60.00 | 3.90 |
| 2579 | N-Methyl-2-pyrrolidone | 61.90 | 58.40 | 58.00 | 3.90 |
| 2580 | trans-Hydrindan | 45.10 | 49.20 | 49.00 | -3.90 |
| 2581 | 8-Oxabicyclo[5.1.0]octane | 45.20 | 48.70 | 49.10 | -3.90 |
| 2582 | 1-Heptylimidazole | 76.80 | 81.10 | 80.70 | -3.90 |
| 2583 | 2,3,7,8-Tetrachlorodibenzofuran | 80.30 | 84.40 | 84.20 | -3.90 |
| 2584 | (4-Methoxyphenyl)trimethylsilane | 56.90 | 60.30 | 60.80 | -3.90 |
| 2585 | (4S,5R)-3,4-Dimethyl-5-phenyl-1,3-oxazolidine | 52.40 | 55.60 | 56.30 | -3.90 |
| 2586 | 2-Ethylbutyryl chloride | 39.40 | 43.00 | 43.30 | -3.90 |
| 2587 | 1-Methylimidazole | 52.00 | 55.30 | 55.90 | -3.90 |
| 2588 | 2,2,6,6-Tetramethyl-3,5-heptanedione | 59.50 | 62.70 | 63.40 | -3.90 |
| 2589 | Triisovalerin | 106.00 | 110.00 | 109.90 | -3.90 |
| 2590 | Isobutyl valerate | 51.00 | 55.10 | 54.90 | -3.90 |
| 2591 | Disulfoton | 76.70 | 79.70 | 80.60 | -3.90 |
| 2592 | 1,1,2,2-Tetrachloroethane | 45.78 | 42.50 | 41.80 | 3.98 |
| 2593 | 1,2,4-Trichlorobenzene | 54.68 | 50.80 | 50.70 | 3.98 |
| 2594 | 1,2,4,7,8-Pentachlorodibenzo-p-dioxin | 91.80 | 87.50 | 87.80 | 4.00 |
| 2595 | 9-Methylanthracene | 88.10 | 84.40 | 84.10 | 4.00 |
| 2596 | Perfluorotridecane | 70.60 | 66.80 | 66.60 | 4.00 |
| 2597 | 2-Methyl-2-isopropyl-1,3-dioxolane | 43.90 | 40.40 | 39.90 | 4.00 |
| 2598 | 1,3,5-Trichlorotrifluorobenzene | 53.80 | 50.20 | 49.80 | 4.00 |
| 2599 | Isobutyl t-amyl ether | 46.30 | 42.30 | 42.30 | 4.00 |
| 2600 | (E) 13-Octadecen-1-ol | 121.20 | 117.50 | 117.20 | 4.00 |
| 2601 | m-Cresylacetate | 60.70 | 56.70 | 56.70 | 4.00 |
| 2602 | 2,2,3,4-Tetramethylhexane | 45.60 | 41.60 | 41.60 | 4.00 |
| 2603 | 2-Methoxy-2-methylpentanenitrile | 48.50 | 52.50 | 52.50 | -4.00 |
| 2604 | 3,3-Bis(chloromethyl)oxetane | 56.00 | 52.50 | 52.00 | 4.00 |
| 2605 | Acetonitrile | 33.00 | 29.10 | 29.00 | 4.00 |
| 2606 | Diethyldipropylsilane | 41.50 | 37.70 | 37.50 | 4.00 |
| 2607 | Diethylene glycol dimethyl ether | 48.00 | 51.60 | 52.00 | -4.00 |
| 2608 | Chloroacetic acid ethylester | 49.50 | 45.50 | 45.50 | 4.00 |
| 2609 | N-Methylaniline | 55.00 | 60.00 | 59.00 | -4.00 |
| 2610 | Octadecyl acetate | 113.50 | 109.20 | 109.50 | 4.00 |
| 2611 | Heptamethylenimine | 46.50 | 50.60 | 50.50 | -4.00 |
| 2612 | Spiro[2.2]nonane | 44.50 | 48.70 | 48.50 | -4.00 |
| 2613 | t-Butylnitrite | 34.00 | 30.60 | 30.00 | 4.00 |
| 2614 | 1,1,1,2,2,3,3,4,4-Nonafluorododecane | 57.60 | 61.60 | 61.60 | -4.00 |
| 2615 | Isopentyl dichloroacetate | 55.60 | 59.60 | 59.60 | -4.00 |
| 2616 | (1-Propoxyethyl)benzene | 56.70 | 60.80 | 60.70 | -4.00 |
| 2617 | 1-Hexyl-2-methylimidazole | 74.70 | 78.20 | 78.70 | -4.00 |
| 2618 | 2,5-Aldehydine | 46.30 | 50.60 | 50.30 | -4.00 |
| 2619 | 4-t-Butyl-2-methylphenol | 72.10 | 76.30 | 76.10 | -4.00 |
| 2620 | 2,4',6-Tribromodiphenyl ether | 83.30 | 87.50 | 87.30 | -4.00 |
| 2621 | Cyclopentene | 28.37 | 32.30 | 32.40 | -4.03 |
| 2622 | 2-Bromophenyl isobutyl ether | 64.00 | 59.90 | 59.90 | 4.10 |
| 2623 | Methyl octacosanoate | 157.50 | 153.70 | 153.40 | 4.10 |
| 2624 | Phenethyl butyrate | 69.70 | 73.90 | 73.80 | -4.10 |
| 2625 | Undecanolactone | 66.20 | 70.30 | 70.30 | -4.10 |
| 2626 | (E) 12-Hexadecen-1-ol | 112.10 | 108.10 | 108.00 | 4.10 |
| 2627 | 1-Iodonaphthalene | 69.90 | 73.90 | 74.00 | -4.10 |
| 2628 | 2-Bromo-7-iodofluorene | 98.10 | 94.20 | 94.00 | 4.10 |
| 2629 | 3,5-Lupetidine | 49.10 | 45.20 | 45.00 | 4.10 |
| 2630 | Benzyl butyl phthalate | 113.90 | 118.00 | 118.00 | -4.10 |
| 2631 | Ethyl 2-bromo-2-methylpropanoate | 45.40 | 49.50 |  | -4.10 |
| 2632 | Ethylene glycol dipropionate | 67.60 | 63.50 | 63.50 | 4.10 |
| 2633 | 4-Methylquinoline | 67.60 | 64.10 | 63.50 | 4.10 |
| 2634 | Methyl butyldiazene | 36.40 | 39.90 | 40.50 | -4.10 |
| 2635 | 1,2-Dichloro-3,3,4,4,5,5-hexafluorocyclopentene | 36.50 | 34.50 | 32.40 | 4.10 |
| 2636 | Benzo[c]cinnoline | 88.60 | 91.00 | 92.70 | -4.10 |
| 2637 | 1-Methoxycyclohexene | 43.70 | 48.30 | 47.80 | -4.10 |
| 2638 | Furan | 28.20 | 32.00 | 32.30 | -4.10 |
| 2639 | Dipropyl glutarate | 73.50 | 77.50 | 77.60 | -4.10 |
| 2640 | 2,3,4,5-Tetramethylhexane | 46.00 | 42.10 | 41.80 | 4.20 |
| 2641 | (1-Bromoethyl)benzene | 56.40 | 52.30 | 52.20 | 4.20 |
| 2642 | 2-Methyl-5-nitrophenol | 85.90 | 82.20 | 81.70 | 4.20 |
| 2643 | 3-Octylthiophene | 67.60 | 71.60 | 71.80 | -4.20 |
| 2644 | 4,4'-Bipyridine | 71.10 | 74.50 | 75.30 | -4.20 |
| 2645 | Crotonaldehyde | 37.30 | 33.20 | 33.10 | 4.20 |
| 2646 | s-Butyl t-amyl ether | 46.80 | 42.60 | 42.60 | 4.20 |
| 2647 | 2,2,3,3-Tetramethylhexane | 45.20 | 41.10 | 41.00 | 4.20 |
| 2648 | Di-t-butyl ether | 37.70 | 33.60 | 33.50 | 4.20 |
| 2649 | Methyl o-toluate | 57.30 | 61.40 | 61.50 | -4.20 |
| 2650 | Ethyl cis-3-pentenoate | 45.00 | 49.20 | 49.20 | -4.20 |
| 2651 | ((Butylthio)methyl)trimethoxysilane | 41.60 | 46.20 | 45.80 | -4.20 |
| 2652 | Pentylcyclohexane | 54.10 | 58.20 | 58.30 | -4.20 |
| 2653 | 2,5-Dimethylthiophene | 40.20 | 44.20 | 44.40 | -4.20 |
| 2654 | Diethylacetamide | 53.70 | 57.60 | 57.90 | -4.20 |
| 2655 | Isoxazole | 37.20 | 40.10 | 41.40 | -4.20 |
| 2656 | 2-Methyl-1,1-diphenylpropane | 73.20 | 77.70 | 77.40 | -4.20 |
| 2657 | 1,1-Dinitropentane | 64.40 | 68.60 |  | -4.20 |
| 2658 | 2,4-Dibromodiphenyl ether | 75.40 | 79.60 | 79.60 | -4.20 |
| 2659 | 2,2',3,3',4-Pentabromodiphenyl ether | 99.10 | 103.20 | 103.30 | -4.20 |
| 2660 | 2,3:6,7-Dibenzocycloocta-2,6-dien-1-one | 90.60 | 94.50 | 94.80 | -4.20 |
| 2661 | cis- Cyclooctene | 41.57 | 46.10 | 45.80 | -4.23 |
| 2662 | 1,1-Dimethylhydrazine | 35.22 | 39.50 |  | -4.28 |
| 2663 | (E) 13-Pentadecen-1-ol | 107.70 | 103.40 | 103.40 | 4.30 |
| 2664 | 1-Propylbenzimidazole | 80.20 | 75.50 | 75.90 | 4.30 |
| 2665 | 2-Acetoxyacetophenone | 74.20 | 70.30 | 69.90 | 4.30 |
| 2666 | Ethyl(phenyl)diethoxysilane | 65.20 | 62.10 | 60.90 | 4.30 |
| 2667 | Hexyl t-amyl ether | 58.60 | 54.40 | 54.30 | 4.30 |
| 2668 | Methyl 2-aminobenzoate | 67.60 | 63.10 | 63.30 | 4.30 |
| 2669 | Piperazine | 50.10 | 45.90 | 45.80 | 4.30 |
| 2670 | 2,3,3-Trimethyl-1-pentene | 38.50 | 34.40 | 34.20 | 4.30 |
| 2671 | Phenyl vinyl ether | 49.00 | 45.10 | 44.70 | 4.30 |
| 2672 | 1,1,1,2,2,3,4,5,5,5-Decafluoropentane | 33.40 | 31.20 | 29.10 | 4.30 |
| 2673 | 1,4-Dichloropentane | 48.90 | 44.80 | 44.60 | 4.30 |
| 2674 | 2,3-Dichlorobutane | 38.40 | 34.10 | 34.10 | 4.30 |
| 2675 | 2-Methoxyethyl acetate | 50.30 | 46.00 | 46.00 | 4.30 |
| 2676 | (Z) 13-Hexadecen-1-ol | 112.30 | 108.10 | 108.00 | 4.30 |
| 2677 | 2,3-Dichlorodibenzofuran | 78.80 | 74.80 | 74.50 | 4.30 |
| 2678 | 2-(2-N,N-Dimethylaminoethyl-N-methylamino)ethanol | 65.20 | 69.90 | 69.50 | -4.30 |
| 2679 | 1-Heptyl-2-methylimidazole | 79.40 | 82.80 | 83.70 | -4.30 |
| 2680 | 2-Methyl-bicyclo[2.2.2]-oct-2-ene | 43.50 | 47.80 | 47.80 | -4.30 |
| 2681 | s-Butyl pentanoate | 51.00 | 55.40 | 55.30 | -4.30 |
| 2682 | 1,1-Dimethoxycyclohexane | 48.60 | 52.80 | 52.90 | -4.30 |
| 2683 | 2-Chloropyrazine | 45.10 | 49.40 |  | -4.30 |
| 2684 | 1,3-Diphenylacetone | 84.60 | 88.50 | 88.90 | -4.30 |
| 2685 | Pentyl butyrate | 53.60 | 57.80 | 57.90 | -4.30 |
| 2686 | Perfluoroethylcyclohexane | 38.60 | 43.00 | 42.90 | -4.30 |
| 2687 | 2,2',4,4',5-Pentabromodiphenyl ether | 108.00 | 103.20 | 103.60 | 4.40 |
| 2688 | Phenoxathiin | 77.30 | 74.10 | 72.90 | 4.40 |
| 2689 | Cumyl chloride | 54.70 | 51.00 | 50.30 | 4.40 |
| 2690 | 1,1,1,3,3,3-Hexafluoro-2-methoxypropane | 32.60 | 28.40 | 28.20 | 4.40 |
| 2691 | 1,2,3,4-Tetrachlorobenzene | 60.10 | 55.60 | 55.70 | 4.40 |
| 2692 | 1-Adamantanecarboxylic acid | 91.10 | 87.30 | 86.70 | 4.40 |
| 2693 | 3,3-Dimethyl-2-ethyl-1-butene | 38.50 | 34.40 | 34.10 | 4.40 |
| 2694 | Di-t-butyl oxalate | 63.50 | 59.30 | 59.10 | 4.40 |
| 2695 | Acetylbromide | 29.60 | 34.00 |  | -4.40 |
| 2696 | (3-Methoxyphenyl)trimethylsilane | 56.10 | 60.30 | 60.50 | -4.40 |
| 2697 | 1-Cyclopropyl-1,3-pentadiyne | 51.90 | 48.20 | 47.50 | 4.40 |
| 2698 | Butylnitrate | 43.60 | 48.00 | 48.00 | -4.40 |
| 2699 | p-Menthadiene | 45.10 | 49.30 | 49.50 | -4.40 |
| 2700 | p-Menthane | 43.60 | 48.00 | 48.00 | -4.40 |
| 2701 | Pentachloroethane | 47.40 | 43.00 | 43.00 | 4.40 |
| 2702 | Pentafluorophenylacetate | 48.10 | 52.00 | 52.50 | -4.40 |
| 2703 | (1-s-Butoxyethyl)benzene | 58.70 | 63.10 | 63.10 | -4.40 |
| 2704 | 1-Methylbenzimidazole | 73.70 | 68.40 | 69.30 | 4.40 |
| 2705 | 2-Iodonaphthalene | 69.80 | 73.90 | 74.20 | -4.40 |
| 2706 | N,N-Dimethyldodecylamine | 69.30 | 74.20 | 73.70 | -4.40 |
| 2707 | Phenethyl hexanoate | 78.80 | 83.20 | 83.20 | -4.40 |
| 2708 | cis-Bicyclo[4.3.0]nona-3,7-diene | 44.90 | 49.20 | 49.30 | -4.40 |
| 2709 | Tricyclo[4.2.2.02,5]deca-3,7,9-triene | 47.90 | 52.20 | 52.30 | -4.40 |
| 2710 | 1-Octylimidazole | 81.00 | 85.80 | 85.40 | -4.40 |
| 2711 | 2,6-Dimethyl-2-heptyl butanoate | 62.50 | 67.00 | 66.90 | -4.40 |
| 2712 | Benzyl acetate | 55.50 | 59.90 | 59.90 | -4.40 |
| 2713 | Isoquinoline | 59.50 | 63.50 | 63.90 | -4.40 |
| 2714 | Tri-s-butyl phosphate | 70.50 | 74.00 | 74.90 | -4.40 |
| 2715 | 2-t-Butylphenol | 77.03 | 72.60 | 72.60 | 4.43 |
| 2716 | 1,3-Dibromobutane | 49.40 | 45.20 | 44.90 | 4.50 |
| 2717 | 4-Methoxybenzaldehyde | 60.40 | 55.40 | 55.90 | 4.50 |
| 2718 | (Z) 13-Pentadecen-1-ol | 107.70 | 103.40 | 103.20 | 4.50 |
| 2719 | N,N-Dimethyl-3-toluidine | 58.20 | 55.70 | 53.70 | 4.50 |
| 2720 | Ethylepoxide | 33.40 | 29.30 | 28.90 | 4.50 |
| 2721 | Colamine | 60.60 | 56.20 | 56.10 | 4.50 |
| 2722 | 1,1,2,2-Tetrafluoro-1-(2,2,2-trifluoroethoxy)ethane | 34.00 | 30.20 | 29.50 | 4.50 |
| 2723 | 3-Ethyl-2,2,3-trimethylpentane | 46.00 | 41.10 | 41.50 | 4.50 |
| 2724 | cis-1-Methyl-4-isopropylcyclohexane | 43.60 | 48.00 | 48.10 | -4.50 |
| 2725 | Tetrapropylsilane | 42.20 | 47.00 | 46.70 | -4.50 |
| 2726 | 2,3-Dichlorodibenzo-p-dioxin | 77.80 | 73.20 | 73.30 | 4.50 |
| 2727 | 3,6-Dichlorodibenzofuran | 70.70 | 74.80 | 75.20 | -4.50 |
| 2728 | 2,3-Difluoroaniline | 49.30 | 53.90 | 53.80 | -4.50 |
| 2729 | 1-Methylcyclohexyl pivalate | 57.90 | 62.30 | 62.40 | -4.50 |
| 2730 | O,O-Diethyl-S-[2-(ethylthio)ethyl] thiophosphate | 76.40 | 79.70 | 80.90 | -4.50 |
| 2731 | Tributylsilane | 42.90 | 47.70 | 47.40 | -4.50 |
| 2732 | 2,3',4,6-Tetrabromodiphenyl ether | 91.10 | 95.30 | 95.60 | -4.50 |
| 2733 | Trihexyl phosphate | 104.80 | 109.20 | 109.30 | -4.50 |
| 2734 | 2,5-Diisopropylphenol | 86.11 | 81.50 | 81.60 | 4.51 |
| 2735 | N,N-Dimethylpropionamide | 52.89 | 57.40 | 57.40 | -4.51 |
| 2736 | Cyclopropane | 18.11 | 22.90 | 22.70 | -4.59 |
| 2737 | 2,4,6-Trichlorodibenzofuran | 84.20 | 79.60 | 79.60 | 4.60 |
| 2738 | Tetradecanoic acid | 110.70 | 105.90 | 106.10 | 4.60 |
| 2739 | 2,3,4,4-Tetramethylhexane | 46.00 | 41.60 | 41.40 | 4.60 |
| 2740 | Benzo[b]fluorene | 97.50 | 93.00 | 92.90 | 4.60 |
| 2741 | Glycerol trinitrate | 92.00 | 88.70 | 87.40 | 4.60 |
| 2742 | 3,6-Dimethyl-3,6-di-t-butyl-1,2,4,5-tetraoxyacyclohexane | 53.70 | 48.30 | 49.10 | 4.60 |
| 2743 | 4,5-Dimethyl-2-furaldehyde | 57.70 | 54.40 | 53.10 | 4.60 |
| 2744 | (E) 13-Hexadecen-1-ol | 112.60 | 108.10 | 108.00 | 4.60 |
| 2745 | 1,5-Dimethyltetrazole | 87.10 | 82.50 |  | 4.60 |
| 2746 | Pentafluorodimethylether | 15.60 | 17.50 | 20.20 | -4.60 |
| 2747 | 1-Decylimidazole | 89.60 | 95.10 | 94.20 | -4.60 |
| 2748 | 4-Methylcyclohexyl 3,3-dimethylbutanoate | 64.10 | 68.60 | 68.70 | -4.60 |
| 2749 | 1,3,5-Cycloheptatriene | 38.70 | 43.00 | 43.30 | -4.60 |
| 2750 | 1,3-Cyclohexadiene | 33.20 | 37.60 | 37.80 | -4.60 |
| 2751 | 2-Ethylpiperidine | 42.20 | 46.70 | 46.80 | -4.60 |
| 2752 | Perfluorodipropyl ether | 31.20 | 35.60 | 35.80 | -4.60 |
| 2753 | 1,2,3,4,6,7,8-Heptachlorodibenzo-p-dioxin | 92.50 | 97.10 | 97.10 | -4.60 |
| 2754 | Tridecanolactone | 75.00 | 79.70 | 79.60 | -4.60 |
| 2755 | Dicyclohexyl succinate | 98.00 | 102.40 | 102.60 | -4.60 |
| 2756 | Dodecanolactone | 70.50 | 75.00 | 75.10 | -4.60 |
| 2757 | m-Toluamide | 89.50 | 84.20 | 84.80 | 4.70 |
| 2758 | Demeton-S-methyl | 78.80 | 75.40 | 74.10 | 4.70 |
| 2759 | 1-Chlorotetradecane | 86.60 | 81.80 | 81.90 | 4.70 |
| 2760 | Anethole | 61.90 | 57.00 | 57.20 | 4.70 |
| 2761 | 1,1-Dimethylsilacyclobutane | 33.00 | 28.50 | 28.30 | 4.70 |
| 2762 | Cycloundecanone | 64.30 | 68.90 | 69.00 | -4.70 |
| 2763 | Triethylenetetramine | 84.70 | 80.50 | 80.00 | 4.70 |
| 2764 | 2-(2-(Dimethylamino)ethoxy)ethanol | 63.80 | 68.50 | 68.50 | -4.70 |
| 2765 | 2-Ethoxyethylacetate | 52.70 | 48.20 | 48.00 | 4.70 |
| 2766 | 3-Fluoropyridine | 45.20 | 40.70 | 40.50 | 4.70 |
| 2767 | 3,4-Dihydro-2H-1-benzopyran | 56.70 | 52.30 | 52.00 | 4.70 |
| 2768 | N,N-Diethylformamide | 50.30 | 53.60 | 55.00 | -4.70 |
| 2769 | 1H-Perfluorooctane | 43.40 | 47.20 | 48.10 | -4.70 |
| 2770 | 2,6-Diisopropylaniline | 69.50 | 75.00 | 74.20 | -4.70 |
| 2771 | 3-Hexylthiophene | 58.50 | 62.30 | 63.20 | -4.70 |
| 2772 | 4,4-Dimethyl-1-hexene | 31.00 | 35.70 | 35.70 | -4.70 |
| 2773 | Butyl pentanoate | 53.00 | 57.80 | 57.70 | -4.70 |
| 2774 | trans-1,2-Dibromocycloheptane | 52.00 | 56.20 | 56.70 | -4.70 |
| 2775 | n-Butyltrichloroacetate | 53.60 | 58.10 | 58.30 | -4.70 |
| 2776 | Perfluoro(propylcyclohexane) | 43.10 | 47.80 | 47.80 | -4.70 |
| 2777 | N-Nitropiperidine | 52.70 | 56.20 | 57.40 | -4.70 |
| 2778 | 1-Benzylimidazole | 79.20 | 83.20 | 83.90 | -4.70 |
| 2779 | 1-Nonyl-2-methylimidazole | 87.40 | 92.20 | 92.10 | -4.70 |
| 2780 | trans-Cyclooctene | 41.57 | 46.10 | 46.30 | -4.73 |
| 2781 | 2,7-Dichlorofluorene | 86.10 | 81.10 | 81.30 | 4.80 |
| 2782 | 3-Fluoroanisole | 48.10 | 43.10 | 43.30 | 4.80 |
| 2783 | DTBP | 38.90 | 33.90 | 34.10 | 4.80 |
| 2784 | Pyrrolizidine | 44.40 | 39.50 | 39.60 | 4.80 |
| 2785 | 1,3,5-Trimethyladamantane | 51.70 | 56.30 | 56.50 | -4.80 |
| 2786 | 2,3,3,4-Tetramethylpentane | 41.80 | 36.80 | 37.00 | 4.80 |
| 2787 | 2,3,3-Trimethyl-1-butene | 34.30 | 29.80 | 29.50 | 4.80 |
| 2788 | Cycloheptene | 36.70 | 41.50 | 41.50 | -4.80 |
| 2789 | 2-Phenylindene | 84.30 | 80.20 | 79.50 | 4.80 |
| 2790 | 5-Azidomethyl-3-nitrooxazolidine | 67.80 | 63.00 |  | 4.80 |
| 2791 | 1-Methylcyclohexyl propanoate | 55.80 | 60.50 | 60.60 | -4.80 |
| 2792 | 2-Chloroethanol | 48.30 | 53.10 | 53.10 | -4.80 |
| 2793 | Methyl methoxyacetate | 39.30 | 43.90 | 44.10 | -4.80 |
| 2794 | N,N-Diethylaminotriethylsilane | 42.20 | 38.40 | 37.40 | 4.80 |
| 2795 | Azidobenzene | 44.80 | 49.40 | 49.60 | -4.80 |
| 2796 | 4-t-Butylphenol | 67.90 | 72.60 | 72.70 | -4.80 |
| 2797 | 2-Methylfuran | 33.50 | 37.40 | 38.30 | -4.80 |
| 2798 | N-Cyclohexylmethylimidazole | 77.50 | 81.60 | 82.30 | -4.80 |
| 2799 | N-Methyl-2,3-dimethyl-3-phenyl-2-butaneamine | 72.50 | 68.20 | 67.60 | 4.90 |
| 2800 | Perfluoro-2,7-dimethyloctane | 48.80 | 45.30 | 43.90 | 4.90 |
| 2801 | Methyl 4-methoxybenzoate | 69.30 | 64.50 | 64.40 | 4.90 |
| 2802 | 2,3-Dimethylpyrazine | 52.60 | 47.90 | 47.70 | 4.90 |
| 2803 | 2,6-Diethylaniline | 65.90 | 71.10 | 70.80 | -4.90 |
| 2804 | 1,1,1-Trinitrobutane | 66.50 | 61.60 |  | 4.90 |
| 2805 | 1-(2-Pyridinyl)ethanone | 60.50 | 55.80 | 55.60 | 4.90 |
| 2806 | Hydrazine | 44.50 | 47.10 | 39.60 | 4.90 |
| 2807 | ((Ethylthio)methyl)trimethoxysilane | 41.40 | 36.90 | 36.50 | 4.90 |
| 2808 | 2,3,3,4-Tetramethylhexane | 46.40 | 41.60 | 41.50 | 4.90 |
| 2809 | 3,3,6,6-Tetrapropyl-1,2,4,5-tetraoxacyclohexane | 65.10 | 69.50 | 70.00 | -4.90 |
| 2810 | 9-Hydroxyfluorene | 91.60 | 96.40 | 96.50 | -4.90 |
| 2811 | Cyclohexyl 3,3-dimethylbutanoate | 62.10 | 66.70 | 67.00 | -4.90 |
| 2812 | Cyclooctatetraene | 43.10 | 48.30 | 48.00 | -4.90 |
| 2813 | 2-Ethyloxazoline | 44.20 | 46.20 | 49.10 | -4.90 |
| 2814 | Methyl phenylacetate | 57.40 | 62.40 | 62.30 | -4.90 |
| 2815 | Cyclopentadiene | 28.40 | 33.00 | 33.30 | -4.90 |
| 2816 | Phlorol | 64.50 | 69.20 | 69.40 | -4.90 |
| 2817 | 1-Hydroxycumene | 65.00 | 70.00 | 69.90 | -4.90 |
| 2818 | 2-Thiophenecarbonitrile | 49.50 | 54.20 | 54.40 | -4.90 |
| 2819 | Di-n-propylsulfoxide | 74.00 | 77.70 | 78.90 | -4.90 |
| 2820 | Linalool | 66.20 | 71.40 | 71.10 | -4.90 |
| 2821 | 2-Bromo-7-chlorofluorene | 89.60 | 84.20 | 84.60 | 5.00 |
| 2822 | 5-Hydroxymethylene-5H-6,7-dihydrodibenzo[a.c]cyclohepten-6-one | 116.10 | 109.20 | 111.10 | 5.00 |
| 2823 | 4-Bromoacetophenone | 66.40 | 61.60 | 61.40 | 5.00 |
| 2824 | trans-Stilbene | 79.70 | 74.70 | 74.70 | 5.00 |
| 2825 | 1-Fluorooctane | 49.70 | 45.90 | 44.70 | 5.00 |
| 2826 | 2-Chloro-2-methylpropane | 28.60 | 25.60 | 23.60 | 5.00 |
| 2827 | 2,4-Difluoronitrobenzene | 58.50 | 53.70 | 53.50 | 5.00 |
| 2828 | 3-Iodoanisole | 62.50 | 58.10 | 57.50 | 5.00 |
| 2829 | 3-Methylcyclohexyl 3,3-dimethylbutanoate | 63.50 | 68.60 | 68.50 | -5.00 |
| 2830 | 3-Phenylisoxazole | 64.00 | 68.00 | 69.00 | -5.00 |
| 2831 | Ethyl bis(isopropyl)cyanoacetate | 65.00 | 70.00 | 70.00 | -5.00 |
| 2832 | n-Butyl-3-chlorobutanoate | 53.10 | 58.10 | 58.10 | -5.00 |
| 2833 | 1,3-Dimethyladamantane | 49.70 | 54.80 | 54.70 | -5.00 |
| 2834 | 4-Nitrobenzamide | 93.70 | 98.20 | 98.70 | -5.00 |
| 2835 | 1,2-Dibromoheptane | 54.40 | 59.20 | 59.40 | -5.00 |
| 2836 | alpha-Pentylcinnamaldehyde | 75.30 | 80.60 | 80.30 | -5.00 |
| 2837 | 3-Chlorodibenzofuran | 75.20 | 70.00 | 70.10 | 5.10 |
| 2838 | 2,2,3,3-Tetramethylpentane | 41.20 | 36.30 | 36.10 | 5.10 |
| 2839 | 3-Methyl-2-thiophenecarboxyaldehyde | 56.20 | 51.60 | 51.10 | 5.10 |
| 2840 | Glycidyl butyrate | 58.70 | 53.60 | 53.60 | 5.10 |
| 2841 | Isobutyroylbromide | 45.70 | 40.60 |  | 5.10 |
| 2842 | Methylcyanoacetate | 56.20 | 51.30 | 51.10 | 5.10 |
| 2843 | 1-Bromo-2-(2-(2-methoxyethoxy)ethoxy)benzene | 83.10 | 78.60 | 78.00 | 5.10 |
| 2844 | Ethyl 4-methylpentanoate | 45.40 | 50.40 | 50.50 | -5.10 |
| 2845 | Isobutyl acetate | 35.90 | 41.10 | 41.00 | -5.10 |
| 2846 | N-(Ethoxymethyl)diethylamine | 39.60 | 35.70 | 34.50 | 5.10 |
| 2847 | Nonafluorocyclopentane | 29.40 | 33.60 | 34.50 | -5.10 |
| 2848 | (1-Methoxyethyl)benzene | 49.10 | 54.00 | 54.20 | -5.10 |
| 2849 | 2-Ethylthiophene | 39.70 | 44.60 | 44.80 | -5.10 |
| 2850 | 1,1,5-Trimethylhexyl 2,2-dimethylpropanoate | 61.30 | 65.70 | 66.40 | -5.10 |
| 2851 | Dotriacontane | 162.50 | 157.70 | 157.30 | 5.20 |
| 2852 | 3-Bromoacetophenone | 66.60 | 61.60 | 61.40 | 5.20 |
| 2853 | 3-Chloroanisole | 53.60 | 48.10 | 48.40 | 5.20 |
| 2854 | 2,2,3,4,4-Pentamethylpentane | 43.50 | 38.40 | 38.30 | 5.20 |
| 2855 | 3-Ethyl-2,3,4-trimethylpentane | 46.50 | 41.60 | 41.30 | 5.20 |
| 2856 | Ethyl(phenyl)dimethoxysilane | 62.50 | 57.80 | 57.30 | 5.20 |
| 2857 | Dimethylmaleate | 64.40 | 59.90 | 59.20 | 5.20 |
| 2858 | 2,2-Dichlorobutane | 36.30 | 32.80 | 31.10 | 5.20 |
| 2859 | 2-Chlorodibenzofuran | 75.20 | 70.00 | 70.00 | 5.20 |
| 2860 | Dicyclohexyl adipate | 106.30 | 111.80 | 111.50 | -5.20 |
| 2861 | Isopropoxytrimethylsilane | 31.80 | 36.30 | 37.00 | -5.20 |
| 2862 | Ethoxybenzene | 50.70 | 45.40 | 45.50 | 5.20 |
| 2863 | Propoxytrimethylsilane | 34.30 | 38.70 | 39.50 | -5.20 |
| 2864 | N-Ethyl-m-toluidine | 60.00 | 66.10 | 65.20 | -5.20 |
| 2865 | Trimethoxy(3-(methylthio)propyl)silane | 43.50 | 48.30 | 48.70 | -5.20 |
| 2866 | Methyltris(2-methylphenoxy)silane | 68.20 | 72.40 | 73.40 | -5.20 |
| 2867 | 1,2,3,5-Tetrachlorobenzene | 60.70 | 55.60 | 55.40 | 5.30 |
| 2868 | 2,5-Difluoronitrobenzene | 58.70 | 53.70 | 53.40 | 5.30 |
| 2869 | 2-Azidoethanol | 62.20 | 57.10 | 56.90 | 5.30 |
| 2870 | 5-Methyl-2-thiophenecarboxyaldehyde | 57.70 | 52.60 | 52.40 | 5.30 |
| 2871 | 3,3,4-Trimethyl-1-pentene | 38.10 | 33.00 | 32.80 | 5.30 |
| 2872 | 3-Chloroacetophenone | 64.00 | 58.50 | 58.70 | 5.30 |
| 2873 | 2-Chloro-3-methylbutane | 35.90 | 31.00 | 30.60 | 5.30 |
| 2874 | 4-Methylene-2-oxetanone | 42.90 | 38.00 | 37.60 | 5.30 |
| 2875 | 3-Bromophenyl isobutyl ether | 65.30 | 59.90 | 60.00 | 5.30 |
| 2876 | Dibenzyl carbonate | 96.70 | 102.20 | 102.00 | -5.30 |
| 2877 | 6,10-Dimethyl-2-undecanone | 75.10 | 69.60 | 69.80 | 5.30 |
| 2878 | n-Propylacrylate | 37.90 | 43.20 | 43.20 | -5.30 |
| 2879 | Hexafluorobenzene | 31.40 | 35.30 | 36.70 | -5.30 |
| 2880 | Pyrazine | 40.50 | 45.40 | 45.80 | -5.30 |
| 2881 | 2-Methyloxazoline | 39.10 | 41.50 | 44.40 | -5.30 |
| 2882 | 2,2',4,4',5,5'-Hexachlorobiphenyl | 99.30 | 94.30 | 93.90 | 5.40 |
| 2883 | 4-Methoxypyridine | 52.70 | 47.70 | 47.30 | 5.40 |
| 2884 | cis-1,2-Cyclohexanediamine | 62.20 | 57.30 | 56.80 | 5.40 |
| 2885 | Bis(dimethylamino)methane | 33.10 | 38.50 |  | -5.40 |
| 2886 | Fumaric acid dichloride | 45.60 | 49.50 | 51.00 | -5.40 |
| 2887 | Trimethoxy(((2-methylpropyl)thio)methyl)silane | 38.70 | 43.50 | 44.10 | -5.40 |
| 2888 | 1H,1H-Perfluorotetradecan-1-ol | 93.80 | 98.90 | 99.20 | -5.40 |
| 2889 | 2,5-Norbornadiene | 34.80 | 40.00 | 40.20 | -5.40 |
| 2890 | n-Butyldichloroacetate | 52.30 | 57.70 | 57.70 | -5.40 |
| 2891 | 2-Methoxyisobutyronitrile | 37.40 | 43.20 | 42.80 | -5.40 |
| 2892 | Bicyclohexyl | 58.00 | 63.50 | 63.40 | -5.40 |
| 2893 | 1-Butoxy-2,3-epoxypropane | 53.33 | 47.70 | 47.90 | 5.43 |
| 2894 | Chlordane | 77.22 | 80.20 | 82.70 | -5.48 |
| 2895 | Tritriacontane | 167.60 | 162.50 | 162.10 | 5.50 |
| 2896 | 2-Chloropropanoic acid | 64.90 | 59.50 | 59.40 | 5.50 |
| 2897 | Tetrahydro-4H-pyran-4-one | 50.70 | 45.30 | 45.20 | 5.50 |
| 2898 | Bis(3-methylphenoxy)dimethylsilane | 61.50 | 66.20 | 67.00 | -5.50 |
| 2899 | 3,5,7,9-Tetraoxaundecane | 53.60 | 57.20 | 59.10 | -5.50 |
| 2900 | Butyl 2-chlorobutyrate | 52.70 | 58.10 | 58.20 | -5.50 |
| 2901 | 1,4,7,10,13,16-Hexaoxacyclooctadecane | 86.10 | 90.90 | 91.60 | -5.50 |
| 2902 | 9-Phenylanthracene | 103.80 | 109.40 | 109.30 | -5.50 |
| 2903 | Benzamide | 86.50 | 80.50 | 80.90 | 5.60 |
| 2904 | Dibenzo-p-dioxin | 68.40 | 63.60 | 62.80 | 5.60 |
| 2905 | 1,2,4,5-Tetrachlorobenzene | 60.70 | 55.60 | 55.10 | 5.60 |
| 2906 | 1-Methylcyclohexyl 3,3-dimethylbutanoate | 61.40 | 66.90 | 67.00 | -5.60 |
| 2907 | 2,2-Dichloropropane | 32.10 | 28.10 | 26.50 | 5.60 |
| 2908 | 3-Thiophenecarboxyaldehyde | 52.60 | 47.30 | 47.00 | 5.60 |
| 2909 | t-Butyl phenyl carbonate | 67.60 | 72.40 | 73.20 | -5.60 |
| 2910 | 1-Methylpyrene | 92.30 | 97.60 | 97.90 | -5.60 |
| 2911 | 2-Methoxytetrahydropyran | 39.30 | 44.80 | 44.90 | -5.60 |
| 2912 | Dimethylpropiolactone | 42.43 | 36.40 | 36.80 | 5.63 |
| 2913 | 5-Hydroxymethylfurfural | 83.40 | 78.40 | 77.70 | 5.70 |
| 2914 | (1-Butoxyethyl)benzene | 59.80 | 65.50 | 65.50 | -5.70 |
| 2915 | cis-3-Octene | 37.30 | 42.90 | 43.00 | -5.70 |
| 2916 | Furfural | 50.70 | 45.70 | 45.00 | 5.70 |
| 2917 | 1,1,1-Trinitropentane | 72.00 | 66.30 |  | 5.70 |
| 2918 | Dimethylether | 18.50 | 24.20 | 24.20 | -5.70 |
| 2919 | N,N-Dimethylbenzamide | 72.50 | 78.20 | 78.20 | -5.70 |
| 2920 | N,N,2,2-Tetramethylpropionamide | 55.10 | 60.80 | 60.80 | -5.70 |
| 2921 | Orthoformic acid trimethyl ester | 38.10 | 42.50 | 43.80 | -5.70 |
| 2922 | 2-n-Propylthiophene | 43.70 | 49.30 | 49.40 | -5.70 |
| 2923 | 2,3-Dihydrobenzofuran | 53.20 | 47.70 | 47.40 | 5.80 |
| 2924 | Bis(cyclohexyl)amine | 64.00 | 69.30 | 69.80 | -5.80 |
| 2925 | N-Benzylbenzaldehyde imine | 85.00 | 78.90 | 79.20 | 5.80 |
| 2926 | 1-(Difluoromethoxy)-1,1,2-trifluoroethane | 31.70 | 27.70 | 25.90 | 5.80 |
| 2927 | Pyrrole | 45.20 | 50.60 | 51.00 | -5.80 |
| 2928 | Carbazole | 87.30 | 82.80 | 81.50 | 5.80 |
| 2929 | Benzo[c]isoxazole | 55.30 | 59.90 | 61.10 | -5.80 |
| 2930 | 1,2-Butanediol | 70.40 | 76.20 | 76.20 | -5.80 |
| 2931 | Cyclodecanone | 58.40 | 64.20 | 64.20 | -5.80 |
| 2932 | Ethyl 3-hydroxyhexanoate | 61.90 | 65.50 | 67.70 | -5.80 |
| 2933 | N-Methylformanilide | 70.30 | 73.40 | 76.10 | -5.80 |
| 2934 | Corannulene | 115.50 | 120.30 | 121.30 | -5.80 |
| 2935 | (6Z)-Octadecenoic acid | 131.20 | 125.30 | 125.30 | 5.90 |
| 2936 | 2-Isopropyl-6-tert-butylphenol | 89.00 | 82.90 | 83.10 | 5.90 |
| 2937 | 1,2,3,4,7-Pentachlorodibenzo-p-dioxin | 92.80 | 87.50 | 86.90 | 5.90 |
| 2938 | 1,1,3-Trimethylsilacyclobutane | 35.50 | 30.40 | 29.60 | 5.90 |
| 2939 | 2,3,5-Trimethylpyrazine | 53.90 | 49.20 | 48.00 | 5.90 |
| 2940 | Propyl 4-chlorobutanoate | 53.60 | 59.50 | 59.50 | -5.90 |
| 2941 | Tetramethylsilane | 26.00 | 31.50 | 31.90 | -5.90 |
| 2942 | Hexacyclopropylethane | 85.80 | 91.50 | 91.70 | -5.90 |
| 2943 | 2,2',3,3',4,4',6-Heptachlorobiphenyl | 104.14 | 99.10 | 98.20 | 5.94 |
| 2944 | Glycidyl propyl ether | 48.54 | 43.00 | 42.60 | 5.94 |
| 2945 | Dibenz[ah]anthracene | 131.10 | 125.40 | 125.10 | 6.00 |
| 2946 | Dicyclopropyl ketone | 53.70 | 48.00 | 47.70 | 6.00 |
| 2947 | 2,2-Dimethyloxirane | 30.90 | 24.90 | 24.90 | 6.00 |
| 2948 | 1,2-Difluoro-3-fluoromethylbenzene | 37.50 | 42.90 | 43.50 | -6.00 |
| 2949 | 2-Fluoronitrobenzene | 59.50 | 53.90 | 53.50 | 6.00 |
| 2950 | 3,3'-Dichlorobiphenyl | 81.00 | 75.20 | 75.00 | 6.00 |
| 2951 | Propyl isovalerate | 44.30 | 50.40 | 50.30 | -6.00 |
| 2952 | 3-Methylbutyl 3-chlorobutyrate | 54.40 | 60.10 | 60.40 | -6.00 |
| 2953 | N,N-Dimethylnicotinamide | 76.10 | 82.70 | 82.10 | -6.00 |
| 2954 | N-Octylaminotriethylsilane | 59.10 | 64.10 | 65.10 | -6.00 |
| 2955 | 1,2,4-Trichlorodibenzo-p-dioxin | 83.90 | 78.00 | 77.80 | 6.10 |
| 2956 | 2-Chloropyridine | 51.00 | 44.90 |  | 6.10 |
| 2957 | 1,2-Difluorotetranitroethane | 62.80 | 56.70 |  | 6.10 |
| 2958 | 5-Ethyl-2-thiophenecarboxaldehyde | 62.20 | 57.30 | 56.10 | 6.10 |
| 2959 | 5-Methyl-2-acetylfuran | 61.70 | 56.30 | 55.60 | 6.10 |
| 2960 | Di-n-propyldiazene N-oxide | 51.70 | 45.60 |  | 6.10 |
| 2961 | (2,2',4,6'-tetrachloro-5-methyldiphenyl)methane | 98.60 | 93.10 | 92.50 | 6.10 |
| 2962 | 2,7-Dibromofluorene | 93.10 | 87.30 | 87.00 | 6.10 |
| 2963 | 1,2,3,7,8-Pentachlorodibenzofuran | 83.60 | 89.20 | 89.70 | -6.10 |
| 2964 | 1,2-Pentanediol | 74.60 | 80.90 | 80.70 | -6.10 |
| 2965 | 2,5-Dihydro-2,5-dimethoxyfuran | 44.20 | 49.30 | 50.30 | -6.10 |
| 2966 | Ethyldipentylsilane | 41.20 | 47.70 | 47.30 | -6.10 |
| 2967 | 1-Hexacosanol | 148.00 | 154.10 | 154.10 | -6.10 |
| 2968 | 5-Ethyl-5-methyltetracosane | 133.80 | 128.00 | 127.60 | 6.20 |
| 2969 | Benzo[b]fluoranthene | 116.80 | 110.70 | 110.60 | 6.20 |
| 2970 | 1-Pentadecanoic acid | 116.60 | 110.60 | 110.40 | 6.20 |
| 2971 | 2,3-Lutidine | 52.00 | 45.90 | 45.80 | 6.20 |
| 2972 | 4-Methylbenzoyl chloride | 61.50 | 56.20 | 55.30 | 6.20 |
| 2973 | Dimethyl-(2,2-dichlorovinyl) phosphate | 68.00 | 61.80 |  | 6.20 |
| 2974 | Perfluoro-2,3-dimethylbutane | 31.60 | 26.30 | 25.40 | 6.20 |
| 2975 | 3-Methoxytoluene | 52.80 | 47.00 | 46.60 | 6.20 |
| 2976 | Trimethoxymethylsilane | 34.30 | 28.00 | 28.10 | 6.20 |
| 2977 | o-Toluamide | 90.70 | 84.20 | 84.50 | 6.20 |
| 2978 | Perfluoropentadecane | 82.70 | 76.30 | 76.50 | 6.20 |
| 2979 | 4-Fluoroanisole | 48.70 | 43.10 | 42.50 | 6.20 |
| 2980 | Dihydro-2(3H)-thiophenone | 38.20 | 43.10 | 44.40 | -6.20 |
| 2981 | Triphenylamine | 90.20 | 94.50 | 96.40 | -6.20 |
| 2982 | 2-Methoxyphenol | 61.40 | 67.70 | 67.60 | -6.20 |
| 2983 | Dicyclohexyl glutarate | 101.10 | 107.10 | 107.30 | -6.20 |
| 2984 | 2-Acetyl-4-methylthiophene | 63.00 | 56.80 | 56.70 | 6.30 |
| 2985 | Dipropylmethylsilane | 35.90 | 29.80 | 29.60 | 6.30 |
| 2986 | trans-2,3-Epoxybutane | 33.10 | 26.90 | 26.80 | 6.30 |
| 2987 | Bis(undecafluoropentyl)ether | 47.30 | 52.50 | 53.60 | -6.30 |
| 2988 | 2,6-Difluoroaniline | 47.50 | 53.90 | 53.80 | -6.30 |
| 2989 | 3-Methylbutyl 2-chlorobutanoate | 54.00 | 60.10 | 60.30 | -6.30 |
| 2990 | 5-Methylisoxazole | 42.00 | 45.50 | 48.30 | -6.30 |
| 2991 | N,N-Dimethyltetradecylamine | 77.30 | 83.50 | 83.60 | -6.30 |
| 2992 | N-(Hydroxyethyl)piperazine | 78.80 | 72.10 | 72.40 | 6.40 |
| 2993 | 2-Methoxyacetophenone | 66.70 | 60.60 | 60.30 | 6.40 |
| 2994 | 1-Chloropentadecane | 92.60 | 86.50 | 86.20 | 6.40 |
| 2995 | 3-Bromodiphenyl ether | 65.40 | 71.80 | 71.80 | -6.40 |
| 2996 | Thiamorpholine | 54.00 | 47.80 | 47.60 | 6.40 |
| 2997 | 4,4'-Dichlorobiphenyl | 81.40 | 75.20 | 75.00 | 6.40 |
| 2998 | Endrin | 76.60 | 80.10 | 83.00 | -6.40 |
| 2999 | Ethylbenzene-2,4-diisocyanate | 61.10 | 64.40 | 67.50 | -6.40 |
| 3000 | 4-Methoxyphenol | 73.70 | 67.70 | 67.30 | 6.40 |
| 3001 | Vinylene carbonate | 41.40 | 46.40 | 47.80 | -6.40 |
| 3002 | 3-Nitro-3-(4-nitrophenyl)pentane | 90.50 | 94.30 | 96.90 | -6.40 |
| 3003 | 6-Undecanone | 59.00 | 65.70 | 65.40 | -6.40 |
| 3004 | Methyl 1-methyl-2-pyrrolecarboxylate | 59.20 | 64.50 | 65.60 | -6.40 |
| 3005 | Palmitic acid | 121.30 | 115.20 | 114.80 | 6.50 |
| 3006 | (E) 2-Ethylhexyl 4-methoxycinnamate | 108.90 | 102.00 | 102.40 | 6.50 |
| 3007 | 4-Methylanisole | 53.30 | 47.00 | 46.80 | 6.50 |
| 3008 | 4-Dimethylaminopyridine | 61.30 | 56.40 | 54.80 | 6.50 |
| 3009 | 1,2,3-Trichlorobenzene | 57.20 | 50.80 | 50.70 | 6.50 |
| 3010 | Methylisocyanate | 28.70 | 24.20 | 22.20 | 6.50 |
| 3011 | 1,2-Dimethylsilacyclobutane | 33.10 | 27.20 | 26.60 | 6.50 |
| 3012 | 2-Pentylthiophene | 52.00 | 58.60 | 58.50 | -6.50 |
| 3013 | 9,10-Dimethylanthracene | 94.50 | 88.00 | 88.00 | 6.50 |
| 3014 | Cyclononanone | 53.10 | 59.60 | 59.60 | -6.50 |
| 3015 | Isopentyl propionate | 44.10 | 50.40 | 50.60 | -6.50 |
| 3016 | N,N-Dimethyl-1-naphthylamine | 66.90 | 74.60 | 73.40 | -6.50 |
| 3017 | Tri(pentylthio)borane | 104.60 | 109.50 | 111.10 | -6.50 |
| 3018 | 2-Thiophenecarboxaldehyde | 54.90 | 48.30 | 48.30 | 6.60 |
| 3019 | Ethyldiisobutylsilane | 39.80 | 32.90 | 33.20 | 6.60 |
| 3020 | Tripropylsilane | 39.10 | 33.60 | 32.50 | 6.60 |
| 3021 | Furfuranol | 58.60 | 64.70 | 65.20 | -6.60 |
| 3022 | 2',3,4-Tribromodiphenyl ether | 81.00 | 87.50 | 87.60 | -6.60 |
| 3023 | 2-Naphthol | 76.20 | 83.40 | 82.80 | -6.60 |
| 3024 | Aziridine | 33.78 | 27.30 | 27.10 | 6.68 |
| 3025 | 1-Chlorohexadecane | 97.90 | 91.20 | 91.20 | 6.70 |
| 3026 | 3-Methoxyphenol | 74.40 | 67.70 | 67.70 | 6.70 |
| 3027 | 2-Ethyl-2-methyl-1,3-dioxolane | 44.80 | 38.40 | 38.10 | 6.70 |
| 3028 | Phenylacetaldehyde | 59.80 | 53.20 | 53.10 | 6.70 |
| 3029 | 1,1,1-Trifluoro-2-(2,2,2-trifluoroethoxy)ethane | 35.00 | 28.20 | 28.30 | 6.70 |
| 3030 | 4-Ethylbenzoic acid | 90.20 | 83.70 | 83.50 | 6.70 |
| 3031 | 1,10-Decanediol | 113.70 | 106.60 | 107.00 | 6.70 |
| 3032 | 1-Methylpyrrole | 40.30 | 45.70 | 47.00 | -6.70 |
| 3033 | 4-Methoxy-1-methyl-2-pyrimidinone | 81.00 | 74.30 |  | 6.70 |
| 3034 | 2,2-Diethyl-1,3-propanediol | 80.20 | 86.70 | 86.90 | -6.70 |
| 3035 | 2,6-Dibromodiphenyl ether | 73.10 | 79.60 | 79.80 | -6.70 |
| 3036 | N,N-Dimethylbutyramide | 55.19 | 62.10 | 61.90 | -6.71 |
| 3037 | (2,2',4,6'-Tetrachloro-3-methyldiphenyl)methane | 100.10 | 93.10 | 93.30 | 6.80 |
| 3038 | 1,3,7,8-Tetrachlorodibenzo-p-dioxin | 88.90 | 82.70 | 82.10 | 6.80 |
| 3039 | 1-(4-Methoxyphenyl)-1H-imidazole | 86.40 | 80.10 | 79.60 | 6.80 |
| 3040 | p-Toluamide | 91.40 | 84.20 | 84.60 | 6.80 |
| 3041 | 2-Chloro-2-methylbutane | 35.00 | 30.30 | 28.20 | 6.80 |
| 3042 | Dimethyldiphenoxysilane | 64.40 | 58.80 | 57.60 | 6.80 |
| 3043 | 4-Bromophenyl isobutyl ether | 66.80 | 59.90 | 60.00 | 6.80 |
| 3044 | 5-Isopropyl-2-methylphenol | 68.20 | 74.90 | 75.00 | -6.80 |
| 3045 | Perfluorotetradecane | 77.80 | 71.50 | 70.90 | 6.90 |
| 3046 | 1-Nitro-2,6-diisopropylbenzene | 68.40 | 74.80 | 75.30 | -6.90 |
| 3047 | Tetrakis(4-methylphenoxy)silane | 77.10 | 72.90 | 70.20 | 6.90 |
| 3048 | 1-Chloro-2,3,4,5,5-pentafluoro-1,3-cyclopentadiene | 31.00 | 27.30 | 24.10 | 6.90 |
| 3049 | Methyl picolinate | 67.00 | 59.70 | 60.10 | 6.90 |
| 3050 | Benzylazide | 48.10 | 55.30 | 55.00 | -6.90 |
| 3051 | Diacetoxymethane | 56.40 | 50.00 | 49.50 | 6.90 |
| 3052 | Trimethoxy(((phenylmethyl)thio)methyl)silane | 56.10 | 62.30 | 63.00 | -6.90 |
| 3053 | 1,2-Propanediol | 64.50 | 71.60 | 71.40 | -6.90 |
| 3054 | 4,5-Dicyano-1-methylimidazole | 86.90 | 86.50 | 93.80 | -6.90 |
| 3055 | Benzo[k]fluoranthene | 117.40 | 110.70 | 110.40 | 7.00 |
| 3056 | 4-Chloroanisole | 54.80 | 48.10 | 47.80 | 7.00 |
| 3057 | 2,2,3,3,4-Pentamethylpentane | 45.20 | 38.40 | 38.20 | 7.00 |
| 3058 | 2-Chloroanisole | 55.00 | 48.10 | 48.00 | 7.00 |
| 3059 | Methylhydroperoxide | 35.00 | 40.30 | 42.00 | -7.00 |
| 3060 | 2,4-Dimethoxypyrimidine | 48.30 | 55.30 |  | -7.00 |
| 3061 | 1,1,1,2,2,3,3,4,4,5,5,6,6-Tridecafluorododecane | 54.90 | 61.70 | 61.90 | -7.00 |
| 3062 | cis-Perfluorobicyclo[4.3.0]nonane | 41.40 | 47.70 | 48.40 | -7.00 |
| 3063 | Benzal diacetate | 69.90 | 76.40 | 76.90 | -7.00 |
| 3064 | trans-1,2-Dibromocyclooctane | 54.54 | 60.90 | 61.60 | -7.06 |
| 3065 | Propyl chloroformate | 40.70 | 33.60 |  | 7.10 |
| 3066 | Chloroacetyl chloride | 44.10 | 38.10 | 37.00 | 7.10 |
| 3067 | Hexylcyclohexane | 55.90 | 63.00 | 63.00 | -7.10 |
| 3068 | o-Terphenyl | 86.40 | 93.50 | 93.50 | -7.10 |
| 3069 | 1,3-Dihydroxybenzene | 78.40 | 85.20 | 85.50 | -7.10 |
| 3070 | Perfluorocyclopentane | 25.60 | 32.40 | 32.70 | -7.10 |
| 3071 | 2,3-Butyleneglycol | 66.60 | 73.80 | 73.70 | -7.10 |
| 3072 | 1,2-Hexanediol | 78.70 | 85.60 | 85.80 | -7.10 |
| 3073 | Margaric acid | 127.30 | 119.90 | 120.10 | 7.20 |
| 3074 | 1-Phenyl-4,7-dioxaspiro[2.4]heptane | 71.30 | 64.30 | 64.10 | 7.20 |
| 3075 | 4-Methyl-2-bromophenyl isobutyl ether | 71.00 | 63.60 | 63.80 | 7.20 |
| 3076 | cis-2,3-Epoxybutane | 33.90 | 26.90 | 26.70 | 7.20 |
| 3077 | 4-Bromoanisole | 58.30 | 51.10 | 51.10 | 7.20 |
| 3078 | Methyl hydroxyacetate | 52.50 | 59.60 | 59.70 | -7.20 |
| 3079 | Tri-s-pentyl phosphate | 81.50 | 88.00 | 88.70 | -7.20 |
| 3080 | (9Z)-Octadecenoic acid | 132.60 | 125.30 | 125.30 | 7.30 |
| 3081 | 1-Methyl-4-piperidone | 54.20 | 46.70 | 46.90 | 7.30 |
| 3082 | Cubane | 44.60 | 37.60 | 37.30 | 7.30 |
| 3083 | 3-Bromoanisole | 58.00 | 51.10 | 50.70 | 7.30 |
| 3084 | Dipropyl carbonate | 53.20 | 60.70 | 60.50 | -7.30 |
| 3085 | Methyl cellosolve | 46.30 | 53.60 | 53.60 | -7.30 |
| 3086 | Diisobutylamine | 39.30 | 46.60 | 46.60 | -7.30 |
| 3087 | (2-(Ethylthio)ethyl)trimethoxysilane | 41.40 | 47.70 | 48.70 | -7.30 |
| 3088 | 1,2,3,4,7,8-Hexachlorodibenzofuran | 86.50 | 94.00 | 93.80 | -7.30 |
| 3089 | 1-Ethyl-4-piperidone | 56.70 | 49.10 | 49.30 | 7.40 |
| 3090 | Ethanediol diacetate | 61.40 | 54.10 | 54.00 | 7.40 |
| 3091 | 1-Methylsilacyclobutane | 25.10 | 19.00 | 17.70 | 7.40 |
| 3092 | Benzyl benzoate | 77.80 | 85.30 | 85.20 | -7.40 |
| 3093 | 2-Hexylthiophene | 56.40 | 63.30 | 63.80 | -7.40 |
| 3094 | 3-Methylbutyl 3-chloropropionate | 54.00 | 61.50 | 61.40 | -7.40 |
| 3095 | Dimethoate | 95.00 | 100.90 | 102.40 | -7.40 |
| 3096 | 2,6-Xylenol | 75.31 | 68.30 | 67.90 | 7.41 |
| 3097 | Benzo[b]triphenylene | 132.30 | 125.40 | 124.80 | 7.50 |
| 3098 | 3-Nitrophenol | 85.90 | 78.50 | 78.40 | 7.50 |
| 3099 | 5-Methyl-1,3-benzodioxole | 54.90 | 47.80 | 47.40 | 7.50 |
| 3100 | 3-Methoxyacetophenone | 67.80 | 60.60 | 60.30 | 7.50 |
| 3101 | Oxalylchloride | 32.00 | 39.40 | 39.50 | -7.50 |
| 3102 | Propyltriethylsilane | 40.00 | 33.00 | 32.50 | 7.50 |
| 3103 | 2-Octylthiophene | 65.40 | 72.60 | 72.90 | -7.50 |
| 3104 | Triethoxy(2-(ethylthio)ethyl)silane | 46.90 | 54.20 | 54.40 | -7.50 |
| 3105 | 1-Chlorodibenzo-p-dioxin | 76.20 | 68.40 | 68.60 | 7.60 |
| 3106 | p-Terphenyl | 101.20 | 93.50 | 93.60 | 7.60 |
| 3107 | Tri(propylthio)borane | 87.00 | 81.50 | 79.40 | 7.60 |
| 3108 | 1,1,1-Trifluoro-2-(1,1,2-trifluoroethoxy)ethane | 35.40 | 29.30 | 27.80 | 7.60 |
| 3109 | Dibutyl carbonate | 62.90 | 70.00 | 70.50 | -7.60 |
| 3110 | (4R,5R)-3,4-Dimethyl-5-phenyl-1,3-oxazolidine | 50.00 | 55.60 | 57.60 | -7.60 |
| 3111 | 2-Diethylaminoethanol | 52.50 | 59.60 | 60.10 | -7.60 |
| 3112 | 2-t-Butylfuran | 38.10 | 45.40 | 45.70 | -7.60 |
| 3113 | Glyceryltriacetate | 83.90 | 76.10 | 76.30 | 7.60 |
| 3114 | 4-Amino-N-(4-ethylphenyl)benzenesulfonamide | 118.80 | 112.40 | 111.10 | 7.70 |
| 3115 | trans-Azobenzene | 72.80 | 72.10 | 65.10 | 7.70 |
| 3116 | E,Z-Nepetalactone | 68.00 | 60.30 | 60.30 | 7.70 |
| 3117 | 2-Methoxy-1,3-dioxolane | 46.40 | 40.70 | 38.70 | 7.70 |
| 3118 | Diisopropymethylsilane | 32.40 | 37.00 | 40.10 | -7.70 |
| 3119 | 8-Oxatricyclo[3,2,1,0(1,5)]octane | 36.50 | 44.10 | 44.20 | -7.70 |
| 3120 | alpha-Phenyl-1-piperidinoacetonitrile | 73.20 | 80.70 | 80.90 | -7.70 |
| 3121 | Methyl 4-iodobenzoate | 65.20 | 72.60 | 72.90 | -7.70 |
| 3122 | 11-Decylheneicosane | 142.00 | 150.20 | 149.70 | -7.70 |
| 3123 | Benzo-12-crown-4 | 82.70 | 75.70 | 74.90 | 7.80 |
| 3124 | Pentachlorobenzene | 67.70 | 60.40 | 59.90 | 7.80 |
| 3125 | (2,2',4,5'-Tetrachloro-5-methyldiphenyl)methane | 101.00 | 93.10 | 93.20 | 7.80 |
| 3126 | 2-Iodoanisole | 65.30 | 58.10 | 57.50 | 7.80 |
| 3127 | Isobutyl 4-chlorobutyrate | 54.00 | 61.50 | 61.80 | -7.80 |
| 3128 | 1,2,4,6,7,8-Hexachlorodibenzofuran | 86.80 | 94.00 | 94.60 | -7.80 |
| 3129 | (9Z)-hexadecenoic acid | 123.00 | 116.00 | 115.10 | 7.90 |
| 3130 | 3-Acetylpyridine | 66.10 | 58.20 | 58.20 | 7.90 |
| 3131 | 4-Methoxy-alpha-methylstyrene | 62.10 | 54.40 | 54.20 | 7.90 |
| 3132 | Hexachloroethane | 51.00 | 43.50 | 43.10 | 7.90 |
| 3133 | 1,2,4-Trimethyl-5-isopropylbenzene | 64.90 | 57.10 | 57.00 | 7.90 |
| 3134 | Perfluoro-2-methyl-2-pentene | 23.40 | 30.20 | 31.30 | -7.90 |
| 3135 | 2-Furancarbonitrile | 44.80 | 51.70 | 52.70 | -7.90 |
| 3136 | 2,4,4'-Tribromodiphenyl ether | 79.70 | 87.50 | 87.60 | -7.90 |
| 3137 | 2,4,6-Trichlorophenol | 67.20 | 75.20 | 75.10 | -7.90 |
| 3138 | (2',3,4,6'-Tetrachloro-6-methyldiphenyl)methane | 101.10 | 93.10 | 93.10 | 8.00 |
| 3139 | 1,2,3,7-Tetrachlorodibenzo-p-dioxin | 90.90 | 82.70 | 82.90 | 8.00 |
| 3140 | 2-Methoxy-3,3-dimethylbutanenitrile | 58.80 | 51.00 | 50.80 | 8.00 |
| 3141 | cis-3,5-Dimethylpyrrolizidine | 47.70 | 41.00 | 39.70 | 8.00 |
| 3142 | 2-Tetralone | 73.00 | 65.30 | 65.00 | 8.00 |
| 3143 | Dibutylperoxide | 40.00 | 47.50 | 48.00 | -8.00 |
| 3144 | 1,2,3,6,7,8-Hexachlorodibenzofuran | 86.10 | 94.00 | 94.10 | -8.00 |
| 3145 | Elaidic acid | 133.00 | 125.30 | 124.90 | 8.10 |
| 3146 | Octamethylcyclotetrasiloxane | 57.00 | 52.00 | 48.90 | 8.10 |
| 3147 | 2-Bromodiphenyl ether | 63.70 | 71.80 | 71.80 | -8.10 |
| 3148 | alpha-Pinene oxide | 53.60 | 45.50 | 45.50 | 8.10 |
| 3149 | 2-Fluorobenzamide | 73.60 | 80.30 | 81.70 | -8.10 |
| 3150 | Perfluoro(1-methyl-4-t-butylcyclohexane) | 54.10 | 47.20 | 45.90 | 8.20 |
| 3151 | 6,7-Dihydro-4(5H)-benzofuranone | 69.40 | 62.10 | 61.20 | 8.20 |
| 3152 | Perfluorohexadecane | 88.40 | 81.00 | 80.20 | 8.20 |
| 3153 | Hexachloropropylene | 54.80 | 49.40 | 46.60 | 8.20 |
| 3154 | 3-Isobutoxybenzoic acid | 99.20 | 90.90 | 91.00 | 8.20 |
| 3155 | 4-t-Butylbenzoic acid | 95.20 | 87.10 | 87.00 | 8.20 |
| 3156 | Trimethoxy((methylthio)methyl)silane | 40.20 | 32.80 | 32.00 | 8.20 |
| 3157 | N,N-Dimethylhexadecylamine | 85.00 | 92.90 | 93.20 | -8.20 |
| 3158 | N,N-Dimethylethanolamine | 46.70 | 54.70 | 54.90 | -8.20 |
| 3159 | Triethanolamine | 105.90 | 114.10 | 114.10 | -8.20 |
| 3160 | 1-Chloroheptadecane | 103.60 | 95.90 | 95.30 | 8.30 |
| 3161 | Linoleic acid | 134.20 | 126.00 | 125.90 | 8.30 |
| 3162 | 3,3,6,6-Tetramethyl-4,5-dihydropyridazine | 50.10 | 43.60 | 41.80 | 8.30 |
| 3163 | 2,4-Pentanediol | 72.50 | 66.00 | 64.20 | 8.30 |
| 3164 | 3-Acetoxyacetophenone | 78.50 | 70.30 | 70.20 | 8.30 |
| 3165 | Cyclododecanone | 65.50 | 73.60 | 73.80 | -8.30 |
| 3166 | 1,1-Ethanediol diacetate | 58.90 | 51.00 | 50.60 | 8.30 |
| 3167 | (2,2',4,4'-Tetrachloro-5-methyldiphenyl)methane | 101.30 | 93.10 | 93.00 | 8.30 |
| 3168 | 2-Fluorophenol | 52.30 | 60.60 | 60.60 | -8.30 |
| 3169 | Butylisobutyl amine | 41.00 | 49.40 | 49.30 | -8.30 |
| 3170 | 2-Isopropoxyethanol | 50.10 | 58.00 | 58.40 | -8.30 |
| 3171 | Tetrachloroethylene | 39.61 | 43.60 | 48.00 | -8.39 |
| 3172 | Methyl 4-dimethylaminobenzoate | 81.00 | 73.30 | 72.60 | 8.40 |
| 3173 | 1-(2-Aminoethyl)-4-methylpiperazine | 64.00 | 55.60 | 55.60 | 8.40 |
| 3174 | 2-Propoxyethanol | 52.10 | 60.40 | 60.50 | -8.40 |
| 3175 | Iodopentafluorobenzene | 57.90 | 50.30 | 49.50 | 8.40 |
| 3176 | (1-Cyclohexyloxyethyl)benzene | 69.80 | 78.00 | 78.20 | -8.40 |
| 3177 | 1,1,2-Triphenylethane | 92.20 | 100.90 | 100.60 | -8.40 |
| 3178 | t-Butyl(phenylacetylenyl)ketone | 65.70 | 73.90 | 74.10 | -8.40 |
| 3179 | Diphenylcarbonate | 80.90 | 88.40 | 89.30 | -8.40 |
| 3180 | 1,8-Octanediol | 105.40 | 97.30 | 96.90 | 8.50 |
| 3181 | Z,E-Nepetalactone | 69.40 | 60.30 | 60.90 | 8.50 |
| 3182 | (2,2',5,5'-Tetrachloro-4-methyldiphenyl)methane | 101.20 | 93.10 | 92.70 | 8.50 |
| 3183 | 5-Methyl-2-nitrophenol | 62.80 | 69.70 | 71.30 | -8.50 |
| 3184 | 1,3,5-Trichlorobenzene | 59.00 | 50.80 | 50.50 | 8.50 |
| 3185 | 3-Nitrobenzamide | 90.50 | 98.20 | 99.00 | -8.50 |
| 3186 | O,O-Diethyl O-2-diethylaminoethyl phosphate | 79.00 | 70.80 | 70.50 | 8.50 |
| 3187 | Di-t-Butylperoxydimethylsilane | 54.60 | 59.80 | 63.10 | -8.50 |
| 3188 | Dinitrofluorophenylethane | 52.70 | 61.20 |  | -8.50 |
| 3189 | Pentafluorophenol | 52.20 | 59.90 | 60.70 | -8.50 |
| 3190 | 2,3,4,6,7,8-Hexachlorodibenzofuran | 85.70 | 94.00 | 94.20 | -8.50 |
| 3191 | 1,2,3-Propanetriol | 90.70 | 98.80 | 99.20 | -8.50 |
| 3192 | (5Z,8Z,11Z,14Z)-Eicosatetraenoic acid | 145.30 | 136.80 | 136.70 | 8.60 |
| 3193 | 1,3,7-Trichlorodibenzo-p-dioxin | 86.40 | 78.00 | 77.80 | 8.60 |
| 3194 | 1-Nonadecanoic acid | 138.00 | 129.20 | 129.40 | 8.60 |
| 3195 | Methyl 4-aminobenzoate | 84.00 | 75.60 | 75.40 | 8.60 |
| 3196 | 1-Chlorooctadecane | 108.80 | 100.50 | 100.20 | 8.60 |
| 3197 | 2-(2-Propoxyethoxy)ethanol | 65.70 | 74.10 | 74.30 | -8.60 |
| 3198 | Fenpropidin | 80.90 | 88.70 | 89.50 | -8.60 |
| 3199 | Stearic acid | 132.60 | 124.60 | 124.00 | 8.60 |
| 3200 | Piperonylonitrile | 70.10 | 62.10 | 61.50 | 8.60 |
| 3201 | 2-Methyl-2-((nitrooxy)methyl)-1,3-propanediol dinitrate | 88.20 | 94.50 | 96.80 | -8.60 |
| 3202 | 1-Methyl-4-piperidinol | 71.50 | 62.60 | 62.80 | 8.70 |
| 3203 | 1-(4-Pyridinyl)ethanone | 66.50 | 58.20 | 57.80 | 8.70 |
| 3204 | Butylcellosolve | 56.60 | 65.10 | 65.30 | -8.70 |
| 3205 | 1,2,3,4,6,8-Hexachlorodibenzofuran | 86.00 | 94.00 | 94.70 | -8.70 |
| 3206 | Dibutoxymethane | 48.10 | 56.70 | 56.80 | -8.70 |
| 3207 | 2-t-Butyl-5-methylphenol | 67.20 | 76.30 | 75.90 | -8.70 |
| 3208 | Hexachlorobenzene | 74.40 | 65.20 | 65.60 | 8.80 |
| 3209 | 4,7,7-Trimethylbicyclo[4.1.0]heptan-3-ol | 78.00 | 69.20 | 69.20 | 8.80 |
| 3210 | 5-Methyl-2-furaldehyde | 59.40 | 51.10 | 50.60 | 8.80 |
| 3211 | Benzphetamine | 77.20 | 86.20 | 86.00 | -8.80 |
| 3212 | Dicyclohexyl phthalate | 113.40 | 122.20 | 122.20 | -8.80 |
| 3213 | Ethyl chloroformate | 37.80 | 28.90 |  | 8.90 |
| 3214 | Ethyldipropylsilane | 37.90 | 29.00 | 29.00 | 8.90 |
| 3215 | Sulfolane | 69.10 | 76.70 | 78.00 | -8.90 |
| 3216 | Trifluoroacetic acid | 41.00 | 49.70 | 49.90 | -8.90 |
| 3217 | 2,3,3',4,4',5-Hexachlorobiphenyl | 102.92 | 94.30 | 94.00 | 8.92 |
| 3218 | 5-Chloro-2-trichloromethyl pyridine | 52.51 | 61.30 | 61.50 | -8.99 |
| 3219 | 4-Methoxybenzoic acid | 90.70 | 82.10 | 81.70 | 9.00 |
| 3220 | N-Methylindoline | 64.20 | 56.70 | 55.20 | 9.00 |
| 3221 | Divinyl sulfide | 38.30 | 29.90 | 29.30 | 9.00 |
| 3222 | 1-Pentylbenzimidazole | 77.00 | 84.80 | 86.00 | -9.00 |
| 3223 | 2,4,6-Trinitrotoluene | 87.00 | 93.10 | 96.00 | -9.00 |
| 3224 | 4-Acetoxyacetophenone | 79.00 | 70.30 | 70.00 | 9.00 |
| 3225 | 2-Ethoxyethanol | 46.90 | 55.70 | 55.90 | -9.00 |
| 3226 | Dibutyldiazene N-oxide | 45.90 | 54.90 |  | -9.00 |
| 3227 | 2,3,8-Trichlorodibenzofuran | 70.80 | 79.60 | 79.80 | -9.00 |
| 3228 | 1,9-Nonanediol | 111.40 | 102.00 | 102.30 | 9.10 |
| 3229 | N-(4-Nitrophenyl)benzenesulfonamide | 111.70 | 103.80 | 102.60 | 9.10 |
| 3230 | 1,2-Ethanediol dinitrate | 70.50 | 62.60 | 61.40 | 9.10 |
| 3231 | (11Z)-Eicosenoic acid | 143.50 | 134.60 | 134.40 | 9.10 |
| 3232 | (2,2',4,4'-Tetrachloro-3-methyldiphenyl)methane | 101.80 | 93.10 | 92.70 | 9.10 |
| 3233 | 2-Nitrophenol | 58.40 | 66.00 | 67.50 | -9.10 |
| 3234 | Tetrabutoxysilane | 52.00 | 59.70 | 61.10 | -9.10 |
| 3235 | 1,4-Dinitrobutane | 77.40 | 72.10 | 68.30 | 9.10 |
| 3236 | Glycerol tri(t-butanoate) | 91.60 | 100.30 | 100.70 | -9.10 |
| 3237 | 2-Fluoroanisole | 52.10 | 43.10 | 42.90 | 9.20 |
| 3238 | 1,2-Dimethylbenzimidazole | 79.00 | 70.20 | 69.80 | 9.20 |
| 3239 | Vitamin B3 | 93.90 | 85.00 | 84.60 | 9.30 |
| 3240 | 1,15-Pentadecanediol | 139.20 | 130.00 | 129.90 | 9.30 |
| 3241 | Acetoin | 48.70 | 57.90 | 58.00 | -9.30 |
| 3242 | 2-Hydroxyacetophenone | 58.30 | 65.70 | 67.60 | -9.30 |
| 3243 | 4-Methoxybenzamide | 97.60 | 87.30 | 88.20 | 9.40 |
| 3244 | Phencyclidine | 77.60 | 86.60 | 87.00 | -9.40 |
| 3245 | t-Butylperoxytrimethylsilane | 34.10 | 43.50 |  | -9.40 |
| 3246 | Methyldipentylsilane | 40.30 | 48.50 | 49.70 | -9.40 |
| 3247 | t-Butyl glycidyl ether | 50.22 | 40.90 | 40.80 | 9.42 |
| 3248 | 2,2,3,3,4,4,5,5-Octamethylhexane | 56.90 | 48.20 | 47.40 | 9.50 |
| 3249 | Imidazo[1,2-a]pyrazine | 67.90 | 67.00 | 58.40 | 9.50 |
| 3250 | 1-Chlorononadecane | 114.70 | 105.20 | 105.20 | 9.50 |
| 3251 | Glycidyl methacrylate | 61.20 | 51.70 | 51.70 | 9.50 |
| 3252 | 1,2,4,6,8,9-Hexachlorodibenzofuran | 85.20 | 94.00 | 94.70 | -9.50 |
| 3253 | Benzo-15-crown-5 | 98.90 | 89.40 | 89.30 | 9.60 |
| 3254 | (2',3,4,4'-Tetrachloro-6-methyldiphenyl)methane | 103.00 | 93.10 | 93.40 | 9.60 |
| 3255 | Diethyldimethylsilane | 38.90 | 29.90 | 29.30 | 9.60 |
| 3256 | 3-Methyl-2-phenylbutane-2-ol | 67.40 | 76.60 | 77.00 | -9.60 |
| 3257 | Amiton | 83.60 | 76.80 | 74.00 | 9.60 |
| 3258 | 1,1,1-Trifluoroacetylacetone | 37.20 | 46.50 | 46.80 | -9.60 |
| 3259 | 2-Hydroxybenzaldehyde | 53.30 | 60.50 | 62.90 | -9.60 |
| 3260 | Isoniazid | 73.10 | 82.80 |  | -9.70 |
| 3261 | Isopropyl chloroacetate | 38.00 | 47.80 | 47.70 | -9.70 |
| 3262 | Diethyl 2-(dimethoxyphosphorylsulfanyl)butanedioate | 93.40 | 100.20 | 103.10 | -9.70 |
| 3263 | Phenylhydrazine | 61.90 | 52.10 |  | 9.80 |
| 3264 | 1,2,3,4,6,8,9-Heptachlorodibenzofuran | 89.20 | 98.80 | 99.00 | -9.80 |
| 3265 | Triisopentylsilane | 43.80 | 53.50 | 53.60 | -9.80 |
| 3266 | 1,16-Hexadecanolide | 83.80 | 93.70 | 93.60 | -9.80 |
| 3267 | Cyclotetradecane | 65.30 | 75.20 | 75.10 | -9.80 |
| 3268 | Arachidic acid | 143.20 | 133.90 | 133.30 | 9.90 |
| 3269 | gamma-Butyrolactone | 47.60 | 37.60 | 37.70 | 9.90 |
| 3270 | 3,3,6,6,9,9-Hexaethyl-1,2,4,5,7,8-hexaoxacyclononane | 63.60 | 72.00 | 73.50 | -9.90 |
| 3271 | 1-Piperazineethanamine | 68.70 | 59.10 | 58.70 | 10.00 |
| 3272 | 1,3-Dimethoxybenzene | 59.70 | 50.10 | 49.70 | 10.00 |
| 3273 | 4-Amino-N-(5-chloro-2-methylphenyl)benzenesulfonamide | 104.00 | 112.60 | 114.00 | -10.00 |
| 3274 | 1-Methoxy-2-propanol | 46.20 | 55.90 | 56.20 | -10.00 |
| 3275 | 3,4-Dihydro-2H-1-benzopyran-2-one | 69.90 | 59.90 | 59.80 | 10.10 |
| 3276 | Decamethylcyclopentasiloxane | 59.00 | 62.90 | 69.10 | -10.10 |
| 3277 | Dimethyl carbonate | 38.00 | 47.00 | 48.20 | -10.20 |
| 3278 | 2-Ethylacrylic acid | 52.10 | 62.00 | 62.30 | -10.20 |
| 3279 | Methyl 1-pyrrolecarboxylate | 52.60 | 62.90 |  | -10.30 |
| 3280 | Ethyl methyl sulphone | 78.00 | 69.40 | 67.60 | 10.40 |
| 3281 | (2,3',4,4'-Tetrachloro-5-methyldiphenyl)methane | 103.80 | 93.10 | 93.40 | 10.40 |
| 3282 | DMSO | 53.30 | 60.10 | 63.70 | -10.40 |
| 3283 | Benzoin | 98.50 | 108.70 | 108.90 | -10.40 |
| 3284 | Methacrylic acid | 47.50 | 57.30 | 57.90 | -10.40 |
| 3285 | Formamide | 60.15 | 51.10 | 49.70 | 10.45 |
| 3286 | 2-Chlorodibenzo-p-dioxin | 78.20 | 68.40 | 67.70 | 10.50 |
| 3287 | Methylacetylacetone (enolform) | 48.50 | 57.70 | 59.00 | -10.50 |
| 3288 | 3-Methylbutyl 4-chlorobutyrate | 55.60 | 66.10 | 66.10 | -10.50 |
| 3289 | alpha-Linolenic acid | 136.90 | 126.70 | 126.30 | 10.60 |
| 3290 | 2,3,7-Trichlorodibenzo-p-dioxin | 88.20 | 78.00 | 77.60 | 10.60 |
| 3291 | m-Anisic acid | 92.00 | 82.10 | 81.40 | 10.60 |
| 3292 | 2-Ethoxy-1,1,1-trinitropropane | 57.70 | 68.30 |  | -10.60 |
| 3293 | 4-Octanol | 57.20 | 67.60 | 67.80 | -10.60 |
| 3294 | 1,2,3,4,7,8,9-Heptachlorodibenzofuran | 88.60 | 98.80 | 99.20 | -10.60 |
| 3295 | 2,8-Dichlorodibenzo-p-dioxin | 84.10 | 73.20 | 73.40 | 10.70 |
| 3296 | 2-(Phenoxymethyl)oxirane | 65.60 | 55.30 | 54.90 | 10.70 |
| 3297 | gamma-Linolenic acid | 135.90 | 126.70 | 125.20 | 10.70 |
| 3298 | Vinyltriethoxysilane | 48.30 | 41.60 | 37.60 | 10.70 |
| 3299 | 1-Chloroeicosane | 120.20 | 109.90 | 109.50 | 10.70 |
| 3300 | Methyl 2-furoate | 45.20 | 54.90 | 55.90 | -10.70 |
| 3301 | 2-t-Butyl-4-methylphenol | 65.70 | 76.30 | 76.40 | -10.70 |
| 3302 | Lactic acid | 69.10 | 79.40 | 79.80 | -10.70 |
| 3303 | 3,5-Dichloro-2-(trichloromethyl)pyridine | 56.27 | 66.10 | 67.00 | -10.73 |
| 3304 | Erucic acid | 154.50 | 144.00 | 143.70 | 10.80 |
| 3305 | 4-Ethoxybenzoic acid | 95.20 | 84.20 | 84.40 | 10.80 |
| 3306 | Heneicosanoic acid | 149.20 | 138.60 | 138.40 | 10.80 |
| 3307 | 2-Bromoanisole | 61.80 | 51.10 | 51.00 | 10.80 |
| 3308 | Butylene carbonate | 63.20 | 52.10 | 52.30 | 10.90 |
| 3309 | 2,2,3,3-Tetramethylbutane | 42.91 | 31.60 | 32.00 | 10.91 |
| 3310 | (((1,1-Dimethylethyl)thio)methyl)trimethoxysilane | 50.60 | 41.20 | 39.60 | 11.00 |
| 3311 | DEHP | 122.80 | 133.90 | 133.80 | -11.00 |
| 3312 | 3-Methoxybenzamide | 98.30 | 87.30 | 87.20 | 11.10 |
| 3313 | Tetraethylsilane | 39.00 | 28.40 | 27.90 | 11.10 |
| 3314 | 2,6-Dichlorophenol | 59.60 | 70.40 | 70.70 | -11.10 |
| 3315 | 2-Biphenyl methanol | 85.60 | 96.60 | 96.70 | -11.10 |
| 3316 | Bis(2-ethylhexyl)terephthalate | 123.30 | 133.90 | 134.40 | -11.10 |
| 3317 | Pyracene | 68.70 | 79.40 | 79.80 | -11.10 |
| 3318 | 4-Nitrophenyl azide | 74.50 | 67.10 | 63.30 | 11.20 |
| 3319 | Ethyldihexylsilane | 44.80 | 57.00 | 56.00 | -11.20 |
| 3320 | Nitrophenylmethane | 54.00 | 64.20 | 65.20 | -11.20 |
| 3321 | 1,3,5-Triphenylbenzene | 133.40 | 122.20 | 122.10 | 11.30 |
| 3322 | gamma-Dodecanolactone | 83.90 | 72.60 | 72.60 | 11.30 |
| 3323 | Mintlactone | 73.20 | 61.70 | 61.90 | 11.30 |
| 3324 | Hexamethylbenzene | 68.60 | 57.90 | 57.30 | 11.30 |
| 3325 | Di-t-butyl-isopropylmethanol | 57.00 | 67.30 | 68.30 | -11.30 |
| 3326 | 6-t-Butyl-2,4-dimethylphenol | 68.40 | 80.00 | 79.70 | -11.30 |
| 3327 | Triphenylethylene | 91.80 | 103.00 | 103.10 | -11.30 |
| 3328 | 1-Docosanoic acid | 154.70 | 143.30 | 143.30 | 11.40 |
| 3329 | 1,3,5-Trimethoxybenzene | 68.20 | 56.90 | 56.80 | 11.40 |
| 3330 | 1,4-Dimethoxybenzene | 61.60 | 50.10 | 50.20 | 11.40 |
| 3331 | 6-Methoxyquinoline | 78.10 | 67.20 | 66.70 | 11.40 |
| 3332 | 3-Chlorosyringol | 68.60 | 79.30 | 80.00 | -11.40 |
| 3333 | Dimethyl perfluoroglutarate | 52.30 | 64.10 | 63.70 | -11.40 |
| 3334 | 2,4-Dichlorophenol | 59.00 | 70.40 | 70.40 | -11.40 |
| 3335 | n-Butylcarbamate | 76.60 | 65.10 |  | 11.50 |
| 3336 | 4-(Hydroxymethyl)-1,3-dioxolan-2-one | 85.40 | 74.70 | 73.90 | 11.50 |
| 3337 | 4-Amino-N-(4-nitrophenyl)benzenesulfonamide | 112.50 | 121.70 | 124.00 | -11.50 |
| 3338 | alpha-Terpineol | 60.70 | 72.40 | 72.20 | -11.50 |
| 3339 | trans-3-Methyl-4-octanolide | 67.50 | 55.90 | 55.90 | 11.60 |
| 3340 | gamma-Undecanolactone | 79.40 | 67.90 | 67.80 | 11.60 |
| 3341 | Flufenamic acid | 101.60 | 111.40 | 113.20 | -11.60 |
| 3342 | exo-Norborneol | 52.50 | 63.90 | 64.10 | -11.60 |
| 3343 | Veratrole | 61.10 | 50.10 | 49.40 | 11.70 |
| 3344 | N-(4-(1-Methylethyl)phenyl)-1-thia-3-azaspiro[5.5]undec-2-en-2-amine | 101.70 | 111.20 | 113.40 | -11.70 |
| 3345 | Triethoxymethylsilane | 45.10 | 34.50 | 33.30 | 11.80 |
| 3346 | gamma-Nonanolactone | 70.30 | 58.60 | 58.50 | 11.80 |
| 3347 | (3-(Ethylthio)propyl)trimethoxysilane | 41.80 | 52.40 | 53.60 | -11.80 |
| 3348 | 1,2,3,4,6,7,8-Heptachlorodibenzofuran | 87.80 | 98.80 | 99.60 | -11.80 |
| 3349 | 3-Methyl-5-phenylisoxazole | 84.10 | 73.40 | 72.20 | 11.90 |
| 3350 | 2-(Benzylamino)ethanol | 74.50 | 86.10 | 86.40 | -11.90 |
| 3351 | delta-Dodecanolactone | 84.60 | 72.60 | 72.60 | 12.00 |
| 3352 | 2,5-Dimethylfuran | 31.80 | 42.70 | 43.80 | -12.00 |
| 3353 | 1-Fluoro-3,3,3-triphenylpropane | 95.90 | 106.90 | 107.90 | -12.00 |
| 3354 | Glycerol triformate | 78.50 | 71.90 | 66.40 | 12.10 |
| 3355 | Isobutyl chloroacetate | 40.00 | 52.10 | 52.10 | -12.10 |
| 3356 | 1,8-Diamino-3,6-dioxaoctane | 58.80 | 70.70 | 70.90 | -12.10 |
| 3357 | delta-Undecanolactone | 80.10 | 67.90 | 67.90 | 12.20 |
| 3358 | gamma-Octanoic lactone | 66.00 | 53.90 | 53.80 | 12.20 |
| 3359 | delta-Nonalactone | 70.70 | 58.60 | 58.50 | 12.20 |
| 3360 | Tetraphenoxysilane | 48.50 | 58.00 | 60.70 | -12.20 |
| 3361 | Methyltriethylsilane | 40.50 | 29.20 | 28.20 | 12.30 |
| 3362 | 1,13-Tridecanediol | 132.80 | 120.70 | 120.50 | 12.30 |
| 3363 | 1,8-Bis(dimethylamino)naphthaline | 76.70 | 90.10 | 89.00 | -12.30 |
| 3364 | 4-t-Pentylphenol | 65.30 | 77.30 | 77.60 | -12.30 |
| 3365 | gamma-Ethylbutyrolactone | 57.20 | 44.60 | 44.80 | 12.40 |
| 3366 | Methyltriphenoxysilane | 71.50 | 61.20 | 59.10 | 12.40 |
| 3367 | Diphenylmethanol | 83.00 | 95.10 | 95.40 | -12.40 |
| 3368 | 2,4-Di-t-butylphenol | 72.40 | 84.40 | 84.80 | -12.40 |
| 3369 | gamma-Decalactone | 75.60 | 63.30 | 63.10 | 12.50 |
| 3370 | 4-Hexylbenzoic acid | 114.70 | 102.30 | 102.10 | 12.60 |
| 3371 | cis-3-Methyl-4-octanolide | 68.40 | 55.90 | 55.80 | 12.60 |
| 3372 | beta-Citronellol | 63.50 | 76.00 | 76.10 | -12.60 |
| 3373 | O,O-Diethyl S-2-diethylaminoethyl dithiophosphate | 86.30 | 76.80 | 73.60 | 12.70 |
| 3374 | delta-Octanolactone | 66.60 | 53.90 | 53.80 | 12.80 |
| 3375 | trans-1,3,5-Hexatriene | 20.50 | 32.60 | 33.30 | -12.80 |
| 3376 | 2-t-Butyl-6-methylphenol | 63.80 | 76.30 | 76.60 | -12.80 |
| 3377 | N-(4-Methylphenyl)-1-thia-3-azaspiro[5.5]undec-2-en-2-amine | 114.80 | 104.60 | 101.90 | 12.90 |
| 3378 | Ethyl 3-hydroxybutyrate | 55.90 | 68.70 | 68.80 | -12.90 |
| 3379 | Alverine | 89.30 | 101.80 | 102.20 | -12.90 |
| 3380 | Perfluorobicyclo[4.4.0]dec-1,6-ene | 45.30 | 57.10 | 58.20 | -12.90 |
| 3381 | Isomintlactone | 74.20 | 61.70 | 61.20 | 13.00 |
| 3382 | 2-Ethyl-1,3-hexanediol | 79.50 | 92.20 | 92.50 | -13.00 |
| 3383 | 1,16-Hexadecanediol | 147.50 | 134.70 | 134.40 | 13.10 |
| 3384 | 4-Heptanolide | 62.30 | 49.30 | 49.20 | 13.10 |
| 3385 | 1,1,2,2-Tetra-t-butylethane | 50.80 | 61.80 | 63.90 | -13.10 |
| 3386 | 2,3-Dichlorophenol | 57.30 | 70.40 | 70.40 | -13.10 |
| 3387 | 2-Bromophenol | 55.50 | 68.70 | 68.70 | -13.20 |
| 3388 | Propyl dichloroacetate | 40.00 | 53.00 | 53.20 | -13.20 |
| 3389 | 2-(Trichloromethyl)pyridine | 43.60 | 56.50 | 56.80 | -13.20 |
| 3390 | 2-Chlorosyringaldehyde | 77.70 | 91.30 | 90.90 | -13.20 |
| 3391 | Tetrapropyl silicate | 49.80 | 41.00 | 36.50 | 13.30 |
| 3392 | 2-Chlorophenol | 52.30 | 65.60 | 65.60 | -13.30 |
| 3393 | Caprolactone | 60.50 | 47.00 | 46.90 | 13.60 |
| 3394 | Tri-t-Butylperoxymethylsilane | 70.40 | 62.80 | 56.80 | 13.60 |
| 3395 | 4-Acetylanisole | 73.80 | 60.60 | 60.20 | 13.60 |
| 3396 | N-(4-Methoxyphenyl)sulfanilamide | 99.40 | 110.90 | 113.00 | -13.60 |
| 3397 | 3-Methyl-4-hydroxybenzaldehyde | 66.90 | 79.70 | 80.50 | -13.60 |
| 3398 | 5-Methyl-3-phenylisoxazole | 84.90 | 73.40 | 71.30 | 13.60 |
| 3399 | N-Benzyl-pivalophenone imine | 81.60 | 89.50 | 95.20 | -13.60 |
| 3400 | 2-(5H)-Thiophenone | 56.00 | 43.80 | 42.30 | 13.70 |
| 3401 | 2,5-Dichlorophenol | 56.70 | 70.40 | 70.40 | -13.70 |
| 3402 | 1,2-Dihydroxybenzene | 71.90 | 85.20 | 85.60 | -13.70 |
| 3403 | N-(4-Ethylphenyl)-1-thia-3-azaspiro[5.5]undec-2-en-2-amine | 120.20 | 109.20 | 106.40 | 13.80 |
| 3404 | delta-Hexalactone | 58.10 | 44.60 | 44.30 | 13.80 |
| 3405 | Propylenecarbonate | 60.00 | 47.50 | 46.20 | 13.80 |
| 3406 | 3,5-Dichlorosyringol | 70.40 | 84.00 | 84.20 | -13.80 |
| 3407 | 1-Adamantanol | 60.80 | 74.00 | 74.60 | -13.80 |
| 3408 | t-Butylperoxymethyloxirane | 53.85 | 41.20 | 40.00 | 13.85 |
| 3409 | 2-Oxetanone | 47.00 | 33.00 | 33.10 | 13.90 |
| 3410 | Hydrocinnamyl alcohol | 62.80 | 76.80 | 76.70 | -13.90 |
| 3411 | 1,4-Cubanedicarboxylic acid | 122.50 | 110.00 | 108.50 | 14.00 |
| 3412 | n-Butylchloroacetate | 41.00 | 54.80 | 55.00 | -14.00 |
| 3413 | Octachlorodibenzofuran | 90.00 | 103.60 | 104.00 | -14.00 |
| 3414 | Cholest-5-en-3-ol | 153.70 | 140.20 | 139.50 | 14.20 |
| 3415 | Dimethyl cubane-1,4-dicarboxylate | 88.50 | 74.90 | 74.30 | 14.20 |
| 3416 | 4-Valerolactone | 53.90 | 39.90 | 39.60 | 14.30 |
| 3417 | t-Pentylnitrate | 59.80 | 45.90 | 45.50 | 14.30 |
| 3418 | 4-Allyl-2-methoxyphenol | 66.30 | 80.20 | 80.60 | -14.30 |
| 3419 | p-Quaterphenyl | 136.10 | 122.20 | 121.70 | 14.40 |
| 3420 | 4-t-Octylphenol | 70.70 | 85.30 | 85.20 | -14.50 |
| 3421 | 1,14-Tetradecanedioic acid | 127.40 | 141.00 | 141.90 | -14.50 |
| 3422 | 1,12-Dodecanediol | 130.50 | 116.00 | 115.90 | 14.60 |
| 3423 | Ethyl 4-aminobenzoate | 91.80 | 77.70 | 77.20 | 14.60 |
| 3424 | 4-Octylbenzoic acid | 126.10 | 111.70 | 111.40 | 14.70 |
| 3425 | Tripentylsilane | 48.10 | 61.70 | 62.80 | -14.70 |
| 3426 | 2,6-Dichlorosyringaldehyde | 82.20 | 96.10 | 97.00 | -14.80 |
| 3427 | Imidazole | 71.30 | 60.20 | 56.50 | 14.80 |
| 3428 | p-Anisylchloride | 74.10 | 59.30 | 59.20 | 14.90 |
| 3429 | 1,3-Dioxolan-2-one | 60.80 | 45.20 | 45.80 | 15.00 |
| 3430 | 3-Dimethylaminopyridine | 70.70 | 56.40 | 55.70 | 15.00 |
| 3431 | Methyldiethylsilane | 34.60 | 20.40 | 19.30 | 15.30 |
| 3432 | Dipropyldibutylsilane | 44.00 | 56.40 | 59.30 | -15.30 |
| 3433 | Dimethyl 2,6-cuneanedicarboxylate | 89.70 | 74.90 | 74.30 | 15.40 |
| 3434 | 4-Nitrobenzylideneaniline | 101.40 | 90.70 | 85.70 | 15.70 |
| 3435 | Perfluoroeicosane | 113.70 | 100.00 | 98.00 | 15.70 |
| 3436 | Methyldihexylsilane | 42.60 | 57.80 | 58.30 | -15.70 |
| 3437 | cis-2,2,4,6,6-Pentamethyl-3-heptene | 63.20 | 48.10 | 47.40 | 15.80 |
| 3438 | Tribenzylamine | 92.60 | 108.60 | 108.50 | -15.90 |
| 3439 | Tetrahydro-2H-pyran-2-one | 58.20 | 42.30 | 42.20 | 16.00 |
| 3440 | N-(3-Chloro-4-methylphenyl)-1-thia-3-azaspiro[5.5]undec-2-en-2-amine | 96.60 | 109.40 | 112.60 | -16.00 |
| 3441 | Hexacosanoic acid | 177.20 | 161.90 | 161.10 | 16.10 |
| 3442 | Tri-n-octylamine | 100.10 | 116.40 | 116.40 | -16.30 |
| 3443 | Octachlorodibenzo-p-dioxin | 86.70 | 101.90 | 103.20 | -16.50 |
| 3444 | 1,14-Tetradecanediol | 141.70 | 125.30 | 125.10 | 16.60 |
| 3445 | Allyl ethyl sulfoxide | 83.70 | 72.50 | 67.10 | 16.60 |
| 3446 | cis-Verbenol | 54.90 | 71.30 | 71.60 | -16.70 |
| 3447 | 4-Amino-N-(2,5-dichlorophenyl)benzene sulfonamide | 127.80 | 113.60 | 111.00 | 16.80 |
| 3448 | 2-Methylnaphthalene | 45.31 | 62.00 | 62.20 | -16.89 |
| 3449 | 1,8-Diaminonaphthalene | 79.60 | 94.80 | 96.50 | -16.90 |
| 3450 | Flurbiprofen | 127.50 | 110.80 | 110.50 | 17.00 |
| 3451 | 3-Phenylcyclobutanone | 46.40 | 63.60 | 63.50 | -17.10 |
| 3452 | Terpinen-4-ol | 55.50 | 72.40 | 72.60 | -17.10 |
| 3453 | Phenoxytrimethylsilane | 56.90 | 43.00 | 39.70 | 17.20 |
| 3454 | Bis(trifluoroacetyl)methane | 30.60 | 46.40 | 47.90 | -17.30 |
| 3455 | 1-Phenyl-1,3-butanedione | 55.60 | 72.00 | 72.90 | -17.30 |
| 3456 | Oxacyclohexadecan-2-one | 71.80 | 89.00 | 89.20 | -17.40 |
| 3457 | (15Z)-Tetracosenoic acid | 170.60 | 153.30 | 152.70 | 17.90 |
| 3458 | (4Z,7Z,10Z,13Z,16Z,19Z)-Docosahexenoic acid | 162.90 | 147.60 | 145.00 | 17.90 |
| 3459 | o-Anisic acid | 86.00 | 69.60 | 68.00 | 18.00 |
| 3460 | Triethylsilane | 37.40 | 19.60 | 19.20 | 18.20 |
| 3461 | 2-Isobutoxybenzoic acid | 95.60 | 78.40 | 77.20 | 18.40 |
| 3462 | trans-2,2,4,6,6-Pentamethyl-3-heptene | 65.90 | 48.10 | 47.40 | 18.50 |
| 3463 | Tetracosanoic acid | 170.70 | 152.60 | 152.10 | 18.60 |
| 3464 | 2,6-Di-t-butylphenol | 66.00 | 84.40 | 84.70 | -18.70 |
| 3465 | 1,4,5,6-Tetrahydropyrimidine | 75.60 | 61.00 | 56.80 | 18.80 |
| 3466 | Propyltributylsilane | 45.00 | 61.10 | 64.00 | -19.00 |
| 3467 | 2,3,3',4,4',5'-Hexachlorobiphenyl | 112.60 | 94.30 | 93.50 | 19.10 |
