# Supplementary material for: Calculation of Five Thermodynamic Molecular Descriptors by Means of a General Computer Algorithm Based on the Group-Additivity Method: Standard Enthalpies of Vaporization, Sublimation and Solvation, and Entropy of Fusion of Ordinary Organic Molecules and Total Phase-Change Entropy of Liquid Crystals
Source: Molecules. 2017 Jun 25;22(7):1059. doi: 10.3390/molecules22071059 (PMC6152037; doi:10.3390/molecules22071059)
Supplement: Supplementary file 1 [file molecules-22-01059-s001.zip › S4. Experimental and Calculated Heat-of-Sublimation Data Table.doc]

|  | Molecules sorted by deviation |  |  |  |  |
| --- | --- | --- | --- | --- | --- |
| No. | Molecule name | ΔH°(sub) exp | ΔH°(sub) calc | ΔH°(sub) test | Deviation |
|  |  |  |  |  |  |
| 1 | 3-Acetylthiophene | 74.60 | 73.60 | 74.60 | 0.00 |
| 2 | N,N-Dimethylformamide | 56.50 | 56.50 |  | 0.00 |
| 3 | 2,5-Di-t-butyl-1,4-dihydroxybenzene | 122.40 | 122.70 | 122.40 | 0.00 |
| 4 | Peroxyundecanoic acid | 125.90 | 125.80 | 125.90 | 0.00 |
| 5 | 1,2,3-Triphenylbenzene | 134.10 | 133.80 | 134.10 | 0.00 |
| 6 | Glutaric anhydride | 86.10 | 85.70 | 86.10 | 0.00 |
| 7 | Protoadamantane | 65.60 | 66.10 | 65.60 | 0.00 |
| 8 | 2-Phenylbenzoic acid | 121.30 | 120.90 | 121.30 | 0.00 |
| 9 | Dimethylnitramine | 69.87 | 69.60 | 69.80 | 0.07 |
| 10 | Gentisic acid | 130.40 | 131.10 | 130.30 | 0.10 |
| 11 | 2,4,5-Trioxoimidazolidine | 119.40 | 119.70 | 119.30 | 0.10 |
| 12 | t-Butylacetamide | 77.90 | 77.00 | 77.80 | 0.10 |
| 13 | Ethyl 4-aminobenzoate | 112.90 | 112.50 | 112.80 | 0.10 |
| 14 | Diphenylcarbonate | 107.60 | 108.80 | 107.70 | -0.10 |
| 15 | Methyl hexadecanoate | 154.30 | 154.60 | 154.20 | 0.10 |
| 16 | Thioxanthene | 101.70 | 101.70 | 101.60 | 0.10 |
| 17 | 2,2-Dimethylglutaric acid | 126.80 | 127.00 | 126.70 | 0.10 |
| 18 | 3-Iodobenzoic acid | 109.10 | 108.50 | 109.00 | 0.10 |
| 19 | 2-Imidazolidinone | 96.60 | 97.50 | 96.50 | 0.10 |
| 20 | 9,10-Dihydrophenanthrene | 88.80 | 88.70 | 88.70 | 0.10 |
| 21 | 3-Methylbenzoic acid | 97.00 | 96.90 | 97.10 | -0.10 |
| 22 | 2-Isopropyl-5-methylphenol | 91.50 | 92.20 | 91.60 | -0.10 |
| 23 | 2,2'-Biphenyldicarboxylic anhydride | 120.70 | 120.40 | 120.80 | -0.10 |
| 24 | 9-Xanthenol | 115.20 | 115.80 | 115.30 | -0.10 |
| 25 | Myristyl alcohol | 143.90 | 143.70 | 143.70 | 0.20 |
| 26 | Benzoic acid | 93.30 | 93.20 | 93.50 | -0.20 |
| 27 | Theophylline | 144.00 | 147.00 | 143.80 | 0.20 |
| 28 | 2,3,4,5-Tetrafluoroaniline | 74.30 | 72.80 | 74.10 | 0.20 |
| 29 | 2-Hydroxybiphenyl | 104.30 | 104.20 | 104.10 | 0.20 |
| 30 | Ethylmalonic acid | 112.80 | 112.10 | 112.60 | 0.20 |
| 31 | Indole-2-carboxylic acid | 121.90 | 121.90 | 121.70 | 0.20 |
| 32 | 2,2',4,4',6,6'-Hexamethylazobenzene N,N'-dioxide | 107.00 | 101.70 | 107.20 | -0.20 |
| 33 | 4-Fluoronitrobenzene | 70.90 | 71.20 | 71.10 | -0.20 |
| 34 | Diphenylmethane | 86.60 | 86.40 | 86.40 | 0.20 |
| 35 | Xanthene | 94.10 | 93.90 | 93.90 | 0.20 |
| 36 | 1,1,1-Trifluoro-4-(2-thienyl)-butane-2,4-dione | 86.20 | 86.50 | 86.40 | -0.20 |
| 37 | Skatole | 90.40 | 90.40 | 90.60 | -0.20 |
| 38 | 5-Methyl-2-thiophenecarboxylic acid | 101.90 | 102.80 | 102.10 | -0.20 |
| 39 | Pyrazine N,N'-dioxide | 116.90 | 119.90 | 117.10 | -0.20 |
| 40 | trans-Diphenyldiazene N-oxide | 98.60 | 98.80 |  | -0.20 |
| 41 | 1,2,3,6,7,8-Hexachlorodibenzo-p-dioxin | 140.00 | 140.30 | 140.20 | -0.20 |
| 42 | 1,2,4,5-Tetraphenylbenzene | 161.40 | 160.90 | 161.10 | 0.30 |
| 43 | 5-Methoxybenzofurazan | 89.20 | 89.90 | 88.90 | 0.30 |
| 44 | Benzenecarbothioamide | 97.20 | 97.20 | 96.90 | 0.30 |
| 45 | 2,3-Xylenol | 84.00 | 84.00 | 83.70 | 0.30 |
| 46 | 2,8-Dichlorodibenzofuran | 101.40 | 101.20 | 101.10 | 0.30 |
| 47 | 4,5-Dicyano-1-methylimidazole | 96.40 | 96.50 | 96.10 | 0.30 |
| 48 | 4-Carboxyphenol | 117.00 | 116.50 | 116.70 | 0.30 |
| 49 | 4-Methoxyphenol | 88.40 | 88.80 | 88.10 | 0.30 |
| 50 | 2,6-Dichloro-4-nitroaniline | 109.20 | 109.60 | 109.50 | -0.30 |
| 51 | 1,10-Phenanthroline | 105.60 | 106.60 | 105.90 | -0.30 |
| 52 | Oxalamide | 113.00 | 113.10 | 112.70 | 0.30 |
| 53 | 1,3-Dihydroxybenzene | 99.70 | 99.80 | 100.00 | -0.30 |
| 54 | 2-Fluoro-2-methyl-1,3-diphenylpropane | 102.20 | 102.50 |  | -0.30 |
| 55 | 3,3-Diphenylpropionic acid | 132.30 | 132.30 | 132.00 | 0.30 |
| 56 | 4,4,4-Trifluoro-1-(2-naphthyl)-butan-1,3-dione | 108.70 | 109.80 | 109.00 | -0.30 |
| 57 | 2,3,6,7-Tetrachloroquinoxaline | 105.90 | 106.10 | 106.20 | -0.30 |
| 58 | Pyrimidine | 58.30 | 58.00 |  | 0.30 |
| 59 | 2,3:7,8-Dibenzocycloocta-2,7-dien-1-one | 112.80 | 112.10 | 112.50 | 0.30 |
| 60 | Suberic acid | 147.80 | 146.80 | 147.50 | 0.30 |
| 61 | 1,6-Hexanedioic acid | 133.60 | 133.10 | 133.20 | 0.40 |
| 62 | 2-Methylbutanedioic acid | 121.70 | 120.70 | 121.30 | 0.40 |
| 63 | 1-Indanone | 78.70 | 77.60 | 78.30 | 0.40 |
| 64 | 2,3-Dimethyl-2,3-dinitrobutane | 75.30 | 73.10 | 74.90 | 0.40 |
| 65 | 2-Amino-4,6-dimethylpyrimidine | 89.50 | 82.00 | 89.10 | 0.40 |
| 66 | 3-Aminoquinoline | 103.10 | 103.10 | 102.70 | 0.40 |
| 67 | N-Methylsuccinimide | 80.10 | 80.30 | 79.70 | 0.40 |
| 68 | Phenanthrene-9,10-dione | 108.10 | 106.60 | 107.70 | 0.40 |
| 69 | 1,2-Bis(methylnitramino)ethane | 110.90 | 108.20 | 110.50 | 0.40 |
| 70 | 2-Chloro-4-nitroaniline | 102.60 | 102.50 | 103.00 | -0.40 |
| 71 | 3,5-Dimethylpyrazole | 83.40 | 83.90 | 83.00 | 0.40 |
| 72 | 2,2,3,3-Tetramethylbutane | 42.90 | 44.20 | 43.30 | -0.40 |
| 73 | 1,4-Dichlorobenzene | 67.10 | 67.40 | 66.70 | 0.40 |
| 74 | 2-Naphthoic acid | 114.90 | 114.90 | 115.30 | -0.40 |
| 75 | 3,5-Dichloroanisole | 79.00 | 79.70 | 79.40 | -0.40 |
| 76 | 4-Chloroaniline | 83.40 | 83.50 | 83.80 | -0.40 |
| 77 | Pimelic acid | 139.90 | 139.90 | 139.50 | 0.40 |
| 78 | Simetryn | 144.00 | 144.40 |  | -0.40 |
| 79 | 4-Cumylphenol | 88.07 | 88.50 | 88.50 | -0.43 |
| 80 | 2,6-Diisopropylnaphthalene | 97.96 | 97.40 | 97.50 | 0.46 |
| 81 | 3,5-Dimethoxyphenol | 101.10 | 101.00 | 100.60 | 0.50 |
| 82 | 2,3,6-Trimethylbenzoic acid | 104.40 | 104.40 | 103.90 | 0.50 |
| 83 | 4-(Methylamino)benzoic acid | 123.90 | 123.50 | 123.40 | 0.50 |
| 84 | Oxamic acid | 108.90 | 109.30 | 108.40 | 0.50 |
| 85 | 1,1-Bis(4-tolyl)ethylene | 101.00 | 98.70 | 100.50 | 0.50 |
| 86 | 1,2,3-Trichlorobenzene | 75.10 | 74.50 | 74.60 | 0.50 |
| 87 | Hexamethyleneglycol | 116.50 | 116.80 | 116.00 | 0.50 |
| 88 | 2-Methylglutaric acid | 126.50 | 127.60 | 127.00 | -0.50 |
| 89 | 3H-1,2-Benzodithiole-3-thione | 107.00 | 106.50 |  | 0.50 |
| 90 | 4-Fluoroacetophenone | 71.60 | 71.10 | 72.10 | -0.50 |
| 91 | 4-t-Butylbenzoic acid | 104.40 | 104.70 | 104.90 | -0.50 |
| 92 | Dibenzo-18-crown-6 | 178.80 | 178.90 | 179.30 | -0.50 |
| 93 | Dimethylmalononitrile | 62.00 | 61.70 | 62.50 | -0.50 |
| 94 | Quinazoline | 77.60 | 78.50 | 78.10 | -0.50 |
| 95 | 3-Quinolinecarbonitrile | 93.20 | 94.20 | 93.70 | -0.50 |
| 96 | Mesitoic acid | 103.60 | 104.40 | 104.10 | -0.50 |
| 97 | N,N'-Dimethylthiourea | 111.80 | 114.60 | 112.30 | -0.50 |
| 98 | 1,2,3,4,7,8-Hexachlorodibenzo-p-dioxin | 140.80 | 140.30 | 140.20 | 0.60 |
| 99 | 1,2,3,7,8-Pentachlorodibenzo-p-dioxin | 134.10 | 133.20 | 133.50 | 0.60 |
| 100 | 5-Iodo-8-quinolinol | 119.00 | 118.50 | 118.40 | 0.60 |
| 101 | 9-Methyl-9H-fluorene | 83.70 | 83.30 | 83.10 | 0.60 |
| 102 | Acenaphthene | 83.40 | 83.00 | 82.80 | 0.60 |
| 103 | Paracetamol | 117.90 | 118.90 | 118.50 | -0.60 |
| 104 | cis-Heptacyclene | 126.50 | 126.60 | 125.90 | 0.60 |
| 105 | Fentanyl | 144.60 | 141.50 | 144.00 | 0.60 |
| 106 | (L)-Methionine | 164.00 | 163.20 | 163.40 | 0.60 |
| 107 | 4-Nitrobenzylideneaniline | 126.00 | 121.60 | 125.40 | 0.60 |
| 108 | 5-Aminoquinoline | 103.30 | 103.10 | 102.70 | 0.60 |
| 109 | 1,3,5-Trimethyluracil | 94.90 | 95.30 | 95.50 | -0.60 |
| 110 | 5,6-Dihydrouracil | 114.40 | 115.50 | 115.00 | -0.60 |
| 111 | 2-Chloropyrimidine | 70.10 | 70.70 |  | -0.60 |
| 112 | Tricyclo{4.3.1.08,10]decane | 65.60 | 66.10 | 66.20 | -0.60 |
| 113 | Thionaphthene-2-carboxylic acid | 116.80 | 116.90 | 117.40 | -0.60 |
| 114 | 2-Iodonaphthalene | 90.80 | 90.20 | 90.20 | 0.60 |
| 115 | 3-Chlorobenzoic acid | 100.60 | 100.30 | 100.00 | 0.60 |
| 116 | 4-Phenylbutyric acid | 113.00 | 113.80 | 113.60 | -0.60 |
| 117 | Benzyl 4-ethoxyphenyl sulfide | 112.50 | 113.80 | 113.10 | -0.60 |
| 118 | 1-Dodecanol | 129.30 | 129.90 | 130.00 | -0.70 |
| 119 | 3,4-Dichloronitrobenzene | 85.80 | 86.40 | 86.50 | -0.70 |
| 120 | 2-Methyl-2-adamantanol | 91.40 | 90.20 | 90.70 | 0.70 |
| 121 | 9-Nitroanthracene | 115.40 | 115.60 | 114.70 | 0.70 |
| 122 | 2,4,5-Trifluoroaniline | 73.70 | 73.70 | 73.00 | 0.70 |
| 123 | 2,4,6-Trinitrotoluene | 113.20 | 113.70 | 113.90 | -0.70 |
| 124 | 2-Fluorobenzamide | 95.40 | 96.10 | 96.10 | -0.70 |
| 125 | 2-Naphthalenemethanol | 106.00 | 106.80 | 106.70 | -0.70 |
| 126 | 2-Hydroxybenzylalcohol | 99.50 | 99.70 | 100.20 | -0.70 |
| 127 | 1,1-Difluoro-1,2-diphenylethane | 94.70 | 94.80 | 95.40 | -0.70 |
| 128 | Ethyl 4-methoxybenzoate | 100.90 | 101.60 | 101.60 | -0.70 |
| 129 | 5-Methylresorcinol | 102.90 | 103.50 | 103.60 | -0.70 |
| 130 | Alpha-caran-3-ol | 79.41 | 79.60 | 78.70 | 0.71 |
| 131 | 2,6-Diethylnaphthalene | 93.63 | 94.80 | 94.40 | -0.77 |
| 132 | 6-Heptadecanol | 157.60 | 158.10 | 158.40 | -0.80 |
| 133 | 1,3-Dimethyluracil | 92.00 | 90.50 | 91.20 | 0.80 |
| 134 | 1-Acetyladamantane | 84.20 | 86.00 | 85.00 | -0.80 |
| 135 | 2,4'-Bipyridine | 87.90 | 88.60 | 88.70 | -0.80 |
| 136 | 2-Chlorobenzoic acid | 101.40 | 100.30 | 100.60 | 0.80 |
| 137 | 2-Nitrobenzonitrile | 88.10 | 86.40 | 87.30 | 0.80 |
| 138 | 2,2'-Dipyridyl N-oxide | 105.10 | 105.70 | 105.90 | -0.80 |
| 139 | 2-Amino-5-methoxybenzoic acid | 119.30 | 120.10 | 120.10 | -0.80 |
| 140 | 3,5-Dibromophenol | 94.30 | 95.10 | 95.10 | -0.80 |
| 141 | N-Theonylthiocarbamic-O-butyl ester | 147.50 | 149.80 | 148.30 | -0.80 |
| 142 | Tetracyclopropylsuccinonitrile | 110.00 | 109.70 | 110.80 | -0.80 |
| 143 | Pentaerythritol | 143.90 | 144.60 | 144.70 | -0.80 |
| 144 | 5-Isopropyl-m-cresol | 91.13 | 92.20 | 92.00 | -0.87 |
| 145 | 3-Iodonitrobenzene | 88.80 | 87.40 | 87.90 | 0.90 |
| 146 | N-Benzoyl-N',N'-diethylurea | 132.20 | 132.10 | 133.10 | -0.90 |
| 147 | 2-Methoxy-1-naphthaldehyde | 107.40 | 105.70 | 106.50 | 0.90 |
| 148 | 3-Bromobenzonitrile | 77.90 | 76.70 | 77.00 | 0.90 |
| 149 | 3-Isopropylbenzoic acid | 104.10 | 105.20 | 105.00 | -0.90 |
| 150 | Vanillyl alcohol | 121.40 | 120.60 | 120.50 | 0.90 |
| 151 | Pentacyclo[5.4.02,603,1005,9]undecane | 54.90 | 54.10 | 54.00 | 0.90 |
| 152 | 2,3-Dichloroquinoxaline | 91.80 | 91.90 | 92.70 | -0.90 |
| 153 | 4-Fluorobenzoic acid | 91.30 | 92.30 | 92.20 | -0.90 |
| 154 | 1,8-Octanediamine | 116.70 | 116.30 | 117.60 | -0.90 |
| 155 | Hexabromobenzene | 109.60 | 109.00 | 108.70 | 0.90 |
| 156 | Methyl 4-dimethylaminobenzoate | 100.10 | 99.10 | 99.20 | 0.90 |
| 157 | Phenylacetic acid | 99.00 | 100.10 | 99.90 | -0.90 |
| 158 | Butanedioic acid | 118.00 | 119.30 | 118.90 | -0.90 |
| 159 | 4-Chloro-2,6-dinitroaniline | 105.20 | 104.10 | 106.10 | -0.90 |
| 160 | Pyracyclene | 83.20 | 85.00 | 84.10 | -0.90 |
| 161 | 2.5-Dichloronitrobenzene | 87.40 | 86.40 | 86.40 | 1.00 |
| 162 | 2,2,6,6-Tetramethyl-4-piperidone | 60.80 | 61.80 | 59.80 | 1.00 |
| 163 | Pentamethylbenzene | 71.70 | 70.80 | 70.70 | 1.00 |
| 164 | 1,2,4-Trihydroxybenzene | 124.20 | 123.10 | 123.20 | 1.00 |
| 165 | 1,3-Diiodobenzene | 82.60 | 83.80 | 83.60 | -1.00 |
| 166 | 1,3-Propylthiourea | 132.50 | 130.30 | 131.50 | 1.00 |
| 167 | 1-Naphthalenecarboxylic acid | 113.60 | 114.90 | 114.60 | -1.00 |
| 168 | 1,4,7,10,13,16-Hexaoxacyclooctadecane | 139.70 | 141.10 | 140.70 | -1.00 |
| 169 | 2-Indanone | 78.30 | 77.60 | 77.30 | 1.00 |
| 170 | 4-Methylbenzoic acid | 97.80 | 96.90 | 96.80 | 1.00 |
| 171 | Phenyl vinyl sulfone | 82.00 | 83.10 | 83.00 | -1.00 |
| 172 | 1,8-Diphenylnaphthalene | 126.40 | 128.10 | 127.40 | -1.00 |
| 173 | 9-Xanthenecarboxylic acid | 130.80 | 131.50 | 131.80 | -1.00 |
| 174 | Phthalimide | 106.30 | 107.90 | 107.30 | -1.00 |
| 175 | 1,1,2-Triphenylethane | 116.60 | 117.90 | 117.70 | -1.10 |
| 176 | 1,8-Dimethylnaphthalene | 82.70 | 81.20 | 81.60 | 1.10 |
| 177 | 2-Hydroxymethyl-1,4-benzodioxan | 106.90 | 108.70 | 108.00 | -1.10 |
| 178 | Protocatechuic acid | 139.00 | 139.80 | 140.10 | -1.10 |
| 179 | 4-Bromobiphenyl | 91.00 | 90.20 | 89.90 | 1.10 |
| 180 | 4-Chloroacetophenone | 77.90 | 79.20 | 79.00 | -1.10 |
| 181 | Benzo[b]fluoranthene | 119.20 | 117.90 | 118.10 | 1.10 |
| 182 | Homocubane-4-carboxylic acid | 82.00 | 81.90 | 80.90 | 1.10 |
| 183 | 1,4-Dihydroxynaphthalene | 120.40 | 121.50 | 121.50 | -1.10 |
| 184 | 3,4,5-Trifluoroaniline | 74.60 | 73.70 | 73.50 | 1.10 |
| 185 | 3-Acetyl-2-methyl-5-phenylthiophene | 108.90 | 110.40 | 110.00 | -1.10 |
| 186 | 1-Hydroxyisoquinoline | 113.60 | 112.70 | 112.50 | 1.10 |
| 187 | 2,3,7-Trichlorodibenzo-p-dioxin | 119.80 | 119.00 | 118.70 | 1.10 |
| 188 | 2-Ethylanthracene | 107.60 | 105.70 | 106.50 | 1.10 |
| 189 | Phenazine | 102.80 | 104.00 | 101.70 | 1.10 |
| 190 | 3-(Acetylamino)benzoic acid | 137.00 | 135.60 | 135.80 | 1.20 |
| 191 | 3-Fluoronitrobenzene | 72.30 | 71.20 | 71.10 | 1.20 |
| 192 | p-Anthranilic acid | 118.00 | 116.40 | 116.80 | 1.20 |
| 193 | 4-Pentyloxybenzoic acid | 134.50 | 133.20 | 133.30 | 1.20 |
| 194 | 2-Chloroquinoline | 84.30 | 83.40 | 83.10 | 1.20 |
| 195 | 1-Nitronaphthalene | 95.10 | 93.90 | 93.90 | 1.20 |
| 196 | 2,2-Dinitroadamantane | 90.60 | 90.60 | 89.40 | 1.20 |
| 197 | 4-Hexyloxybenzoic acid | 139.40 | 140.10 | 140.60 | -1.20 |
| 198 | 4-Methyl-2,6,7-trioxabicyclo[2.2.2]octane | 67.40 | 66.20 |  | 1.20 |
| 199 | 1-(2-Nitrophenyl)pyrrole | 101.30 | 103.50 | 102.50 | -1.20 |
| 200 | 2-Methyladamantane | 68.20 | 67.00 | 67.00 | 1.20 |
| 201 | t-Butylurea | 98.20 | 97.30 | 97.00 | 1.20 |
| 202 | 2-Amino-5-methylbenzoic acid | 110.60 | 111.60 | 111.80 | -1.20 |
| 203 | 2-Furoic acid | 88.40 | 88.40 | 87.20 | 1.20 |
| 204 | 3-Amino-4-methylbenzoic acid | 119.40 | 120.20 | 120.60 | -1.20 |
| 205 | 4-Fluorobenzonitrile | 67.20 | 66.50 | 66.00 | 1.20 |
| 206 | Fenchone | 67.30 | 68.50 | 68.50 | -1.20 |
| 207 | Homopiperonylic acid | 122.90 | 121.00 | 121.70 | 1.20 |
| 208 | 2-Methylbenzoic acid | 95.90 | 96.90 | 97.10 | -1.20 |
| 209 | Pentachlorobenzene | 87.10 | 88.80 | 88.30 | -1.20 |
| 210 | 1-Norbornylisocyanide | 60.60 | 61.80 |  | -1.20 |
| 211 | Coprostane | 133.80 | 133.60 | 132.60 | 1.20 |
| 212 | 4-Cyanopyridine | 73.20 | 73.70 | 74.40 | -1.20 |
| 213 | 1-Cyanonaphthalene | 88.10 | 89.20 | 89.30 | -1.20 |
| 214 | Methyl octadecanoate | 167.70 | 168.30 | 169.00 | -1.30 |
| 215 | 2-(Acetylamino)benzoic acid | 127.60 | 127.00 | 126.30 | 1.30 |
| 216 | 2-Naphthalenecarbonitrile | 90.70 | 89.20 | 89.40 | 1.30 |
| 217 | 2-Pyridinecarbonitrile | 70.70 | 68.90 | 69.40 | 1.30 |
| 218 | 1,5-Naphthalenediamine | 122.50 | 121.40 | 121.20 | 1.30 |
| 219 | 2,5-Xylenol | 85.00 | 84.00 | 83.70 | 1.30 |
| 220 | 2-Acetoxyacetophenone | 104.50 | 103.10 | 103.20 | 1.30 |
| 221 | 2-Methoxy-5-nitrophenol | 106.20 | 107.70 | 107.50 | -1.30 |
| 222 | 3-Thiopheneacetic acid | 100.90 | 101.70 | 102.20 | -1.30 |
| 223 | 4-Bromoiodobenzene | 76.70 | 77.80 | 78.00 | -1.30 |
| 224 | 4-Bromophenol | 84.20 | 85.80 | 85.50 | -1.30 |
| 225 | Chloroacetic acid | 82.20 | 80.90 |  | 1.30 |
| 226 | Fluorene | 80.20 | 82.00 | 81.50 | -1.30 |
| 227 | 1-((5-Phenylamino)-1,2,4-thiadiazol-3-yl)-2-propanol | 123.80 | 122.70 | 122.50 | 1.30 |
| 228 | 3,4-Difluorophenol | 72.90 | 74.70 | 74.20 | -1.30 |
| 229 | n-Butylmalonic acid | 124.60 | 125.80 | 125.90 | -1.30 |
| 230 | 3-Pyridinecarbonitrile | 72.10 | 73.70 | 73.40 | -1.30 |
| 231 | 2-Chlorodibenzofuran | 95.30 | 94.10 | 94.00 | 1.30 |
| 232 | 3,4,5-Trimethoxybenzoic acid | 131.20 | 130.00 | 129.90 | 1.30 |
| 233 | Lauric peroxyacid | 131.40 | 132.70 |  | -1.30 |
| 234 | Imidodicarbonic diamide | 126.70 | 124.20 | 125.30 | 1.40 |
| 235 | 4-Amino-3-methylbenzoic acid | 122.00 | 120.20 | 120.60 | 1.40 |
| 236 | 2-Cyanophenyl benzyl sulfide | 117.80 | 115.70 | 116.40 | 1.40 |
| 237 | 3-Cyanopyridine N-oxide | 101.90 | 100.80 | 100.50 | 1.40 |
| 238 | 2-Carbamoyl-3-methylquinoxaline N-oxide | 138.00 | 135.60 | 136.60 | 1.40 |
| 239 | 3-Chlorodibenzofuran | 95.30 | 94.10 | 93.90 | 1.40 |
| 240 | 4-Iodophenol | 90.20 | 91.80 | 91.60 | -1.40 |
| 241 | Methyl 4-aminobenzoate | 104.20 | 105.40 | 105.60 | -1.40 |
| 242 | Dibenzoylmethane (keto form) | 115.70 | 117.90 | 117.10 | -1.40 |
| 243 | 2,4-Dichloronitrobenzene | 87.80 | 86.40 | 86.30 | 1.50 |
| 244 | 4-Iodoanisole | 82.40 | 80.80 | 80.90 | 1.50 |
| 245 | 2,2'-Dichlorobiphenyl | 96.20 | 95.10 | 94.70 | 1.50 |
| 246 | 1,1-Diphenylethanol | 105.00 | 106.50 | 106.50 | -1.50 |
| 247 | 1-Hydroxycumene | 82.30 | 82.60 | 83.80 | -1.50 |
| 248 | cis-Verbenol | 81.20 | 84.00 | 82.70 | -1.50 |
| 249 | 4-Methoxy-alpha-methylstyrene | 81.20 | 81.10 | 79.70 | 1.50 |
| 250 | Phenothiazine | 114.50 | 112.90 | 113.00 | 1.50 |
| 251 | 1,1,1,3,5,5,5-Heptanitropentane | 114.20 | 112.90 | 115.70 | -1.50 |
| 252 | 2,3,6-Trimethylphenol | 86.70 | 87.70 | 88.20 | -1.50 |
| 253 | 3,5-Xylenol | 82.90 | 84.00 | 84.40 | -1.50 |
| 254 | 5-Phenylvaleric acid | 119.40 | 120.70 | 120.90 | -1.50 |
| 255 | Isopropylurea | 98.10 | 99.70 | 99.60 | -1.50 |
| 256 | N-Acetyl-L-alanine amide | 118.10 | 119.50 | 119.60 | -1.50 |
| 257 | 5-hydroxynicotinic acid | 149.80 | 148.80 | 148.20 | 1.60 |
| 258 | 1-(4-Iodophenyl)pyrrole | 100.70 | 99.90 | 99.10 | 1.60 |
| 259 | 3-Chlorobenzonitrile | 76.70 | 74.60 | 75.10 | 1.60 |
| 260 | 1,4-Dicyanobenzene di-N-oxide | 73.00 | 71.40 |  | 1.60 |
| 261 | 3-Hydroxybenzamide | 122.00 | 120.30 | 120.40 | 1.60 |
| 262 | 4-(Acetylamino)benzoic acid | 137.00 | 135.60 | 135.40 | 1.60 |
| 263 | p-Toluidine | 78.80 | 80.20 | 80.40 | -1.60 |
| 264 | 1,3-Benzoxazine-2,4-dione | 114.20 | 114.30 | 112.60 | 1.60 |
| 265 | Dimethyl sulfone | 77.00 | 76.90 | 75.40 | 1.60 |
| 266 | 3-Fluorobenzoic acid | 93.30 | 92.30 | 91.70 | 1.60 |
| 267 | Indole-3-carboxylic acid | 123.90 | 126.60 | 125.50 | -1.60 |
| 268 | (3,4-Dichlorophenyl)-(1-aza-3-thiabicyclo[3.3.1]non-2-ylidene)amine | 125.00 | 126.60 | 126.60 | -1.60 |
| 269 | Benzyl methyl sulfone | 99.00 | 100.00 | 100.60 | -1.60 |
| 270 | 1,3-Diphenylacetone | 111.60 | 109.70 | 110.00 | 1.60 |
| 271 | Hexachlorobenzene | 96.80 | 95.90 | 95.20 | 1.60 |
| 272 | Phenanthridine | 100.00 | 101.70 | 101.60 | -1.60 |
| 273 | Sorbitol | 202.00 | 204.30 | 203.60 | -1.60 |
| 274 | 2-Methyl-2-propenyl p-tolyl sulphone | 107.00 | 104.80 | 105.30 | 1.70 |
| 275 | 3-Aminopyridine | 84.00 | 82.70 | 82.30 | 1.70 |
| 276 | Thioxanthone | 114.80 | 109.90 | 113.10 | 1.70 |
| 277 | 2,3,4,6-Tetramethylbenzoic acid | 109.70 | 108.20 | 108.00 | 1.70 |
| 278 | 5-Amino-1-methyltetrazole | 120.20 | 118.50 |  | 1.70 |
| 279 | 1,3,7,8-Tetrachlorodibenzo-p-dioxin | 127.80 | 126.10 | 126.10 | 1.70 |
| 280 | 1-Benzylimidazole | 102.10 | 100.50 | 100.40 | 1.70 |
| 281 | 2-Chloro-5-nitroaniline | 101.00 | 102.50 | 102.70 | -1.70 |
| 282 | 2-Furanacrolein | 76.00 | 77.10 | 77.70 | -1.70 |
| 283 | 4-Nitrobenzamide | 114.20 | 115.90 | 115.90 | -1.70 |
| 284 | Azulene | 68.00 | 69.20 | 69.70 | -1.70 |
| 285 | 3,4-Xylenol | 85.73 | 84.00 | 84.00 | 1.73 |
| 286 | (11Z)-Eicosenoic acid | 192.90 | 189.60 | 191.10 | 1.80 |
| 287 | Benzofurazan N-oxide | 79.60 | 77.90 | 77.80 | 1.80 |
| 288 | 1,4-Diiodobenzene | 85.40 | 83.80 | 83.60 | 1.80 |
| 289 | 2,2-diphenyl-1,3-dioxolane | 99.70 | 101.40 | 101.50 | -1.80 |
| 290 | 2-Nitroaniline | 89.00 | 86.70 | 87.20 | 1.80 |
| 291 | 3-Aminophenol | 101.60 | 99.70 | 99.80 | 1.80 |
| 292 | 3-Nitroaniline | 96.50 | 95.40 | 94.70 | 1.80 |
| 293 | 4-Fluorophenol | 74.10 | 75.60 | 75.90 | -1.80 |
| 294 | Diphenylamine | 99.70 | 98.90 | 97.90 | 1.80 |
| 295 | 1,2-Diphenylethane | 91.40 | 93.20 | 93.20 | -1.80 |
| 296 | 1,2,4-Triazole | 84.10 | 87.80 | 85.90 | -1.80 |
| 297 | 1H-Indazole | 91.10 | 91.80 | 92.90 | -1.80 |
| 298 | 4-Chromanone | 84.60 | 86.30 | 86.40 | -1.80 |
| 299 | 4-Propylbenzoic acid | 109.10 | 110.70 | 110.90 | -1.80 |
| 300 | Peroxydecanoic acid | 117.10 | 118.90 |  | -1.80 |
| 301 | 4-Phenoxyphenol | 112.80 | 114.80 | 114.60 | -1.80 |
| 302 | 1-(3,5-Dichlorophenyl)-2,5-dimethylpyrrole | 97.50 | 98.80 | 99.30 | -1.80 |
| 303 | 2,4,5,6-Tetrachloropyrimidine | 83.00 | 84.80 |  | -1.80 |
| 304 | 1-Bromo-4-chlorobenzene | 68.10 | 69.60 | 70.00 | -1.90 |
| 305 | 2-Bromonaphthalene | 81.60 | 84.20 | 83.50 | -1.90 |
| 306 | m-Anisic acid | 107.50 | 105.50 | 105.60 | 1.90 |
| 307 | Methyl 4-bromobenzoate | 89.30 | 91.50 | 91.20 | -1.90 |
| 308 | alpha-Methyl-5-((3-methylphenyl)amino)-1,2,4-thiadiazole-3-ethanol | 137.30 | 135.80 | 135.40 | 1.90 |
| 309 | 9-Anthroic acid | 139.40 | 136.70 | 137.50 | 1.90 |
| 310 | 2,6-Dimethylbenzoic acid | 99.10 | 100.70 | 101.00 | -1.90 |
| 311 | 2-Propylbenzimidazole | 109.40 | 108.30 | 107.50 | 1.90 |
| 312 | 3-(2-Methoxyphenyl)propionic acid | 117.80 | 119.20 | 119.70 | -1.90 |
| 313 | Hexanamide | 98.70 | 100.60 | 100.60 | -1.90 |
| 314 | Pyrene | 104.50 | 106.10 | 106.40 | -1.90 |
| 315 | 2-Acetylbenzofuran | 88.20 | 90.30 | 90.10 | -1.90 |
| 316 | Mephenamic acid | 136.30 | 137.80 | 138.20 | -1.90 |
| 317 | 1,4-Benzodioxan-2-carboxylic acid | 117.80 | 116.90 | 115.80 | 2.00 |
| 318 | Mesitylenic acid | 102.30 | 100.70 | 100.30 | 2.00 |
| 319 | 1,2,3,4-Tetrahydro-N-methylcarbazole | 94.10 | 92.20 | 92.10 | 2.00 |
| 320 | 2-Fluorobenzoic acid | 94.40 | 92.30 | 92.40 | 2.00 |
| 321 | Benzo[a]fluorene | 105.40 | 103.30 | 103.40 | 2.00 |
| 322 | 2,2-Dimethylsuccinic acid | 122.70 | 120.10 | 120.70 | 2.00 |
| 323 | [2.2]Paracyclophane-1,9-diene | 93.10 | 95.20 | 95.10 | -2.00 |
| 324 | 2,4,6-Tri-t-butylnitrosobenzene | 91.00 | 92.40 | 93.00 | -2.00 |
| 325 | 2-Chloronitrobenzene | 80.90 | 79.20 | 78.90 | 2.00 |
| 326 | 2-Methoxybenzamide | 111.00 | 109.30 | 109.00 | 2.00 |
| 327 | Piperonylonitrile | 90.90 | 88.30 | 88.90 | 2.00 |
| 328 | Succinonitrile | 70.00 | 67.80 | 68.00 | 2.00 |
| 329 | Tritane | 108.40 | 111.20 | 110.40 | -2.00 |
| 330 | N,N-Diethylbenzamide | 91.40 | 92.70 | 93.40 | -2.00 |
| 331 | 1,3,5-Trinitrobenzene | 107.30 | 110.00 | 109.30 | -2.00 |
| 332 | 5-Methoxy-3,4-dihydro-2H-naphthalen-1-one | 97.90 | 96.70 | 95.80 | 2.10 |
| 333 | 4-Chlorobenzonitrile | 76.70 | 74.60 | 74.60 | 2.10 |
| 334 | Succinyl anhydride | 80.70 | 78.90 | 78.60 | 2.10 |
| 335 | 1,2,3,5-Tetrachlorobenzene | 79.60 | 81.70 | 81.70 | -2.10 |
| 336 | 1,2,4,5-Tetrachlorobenzene | 83.20 | 81.70 | 81.10 | 2.10 |
| 337 | 3,4-Dinitrobenzoic acid | 129.00 | 131.10 | 131.10 | -2.10 |
| 338 | 4-Chlorobenzoic acid | 102.50 | 100.30 | 100.40 | 2.10 |
| 339 | Biphenyl | 77.90 | 79.70 | 80.00 | -2.10 |
| 340 | Dibenzofuran | 84.80 | 87.00 | 86.90 | -2.10 |
| 341 | Ethyl 4-hydroxybenzoate | 110.40 | 112.60 | 112.50 | -2.10 |
| 342 | 1,4-Diaminobutane | 87.10 | 88.80 | 89.20 | -2.10 |
| 343 | 2-Chlorobenzonitrile | 72.30 | 74.60 | 74.40 | -2.10 |
| 344 | 4-Isopropylbenzylidine-t-butylamine N-oxide | 101.80 | 103.40 | 103.90 | -2.10 |
| 345 | Phthalhydrazide | 139.80 | 141.50 | 141.90 | -2.10 |
| 346 | 1,3,5-Trithiane | 93.20 | 95.30 |  | -2.10 |
| 347 | 2,3-Bis(bromomethyl)quinoxaline | 114.00 | 116.20 | 116.10 | -2.10 |
| 348 | 2,7-Dichlorodibenzo-p-dioxin | 113.80 | 111.90 | 111.70 | 2.10 |
| 349 | 3-Nitrobenzoic acid | 110.00 | 112.10 | 112.10 | -2.10 |
| 350 | 4-Isobutoxybenzoic acid | 119.00 | 120.90 | 121.10 | -2.10 |
| 351 | Perylene | 125.50 | 127.40 | 127.60 | -2.10 |
| 352 | 2-Aminothiazole | 85.20 | 86.30 | 87.30 | -2.10 |
| 353 | Ethylenethiourea | 107.40 | 104.60 | 105.20 | 2.20 |
| 354 | 1,4-Benzodioxan-6-carboxaldehyde | 98.20 | 98.50 | 96.00 | 2.20 |
| 355 | 3,5-Difluorophenol | 72.80 | 74.70 | 75.00 | -2.20 |
| 356 | 1,4-Dimethoxybenzene | 80.30 | 77.70 | 78.10 | 2.20 |
| 357 | n-Decyl alcohol | 114.60 | 116.20 | 116.80 | -2.20 |
| 358 | 1-Methylindole | 73.90 | 71.80 | 71.70 | 2.20 |
| 359 | 1-Thiaindan | 73.90 | 76.20 | 76.10 | -2.20 |
| 360 | 2-Nitrobenzamide | 117.90 | 115.90 | 115.70 | 2.20 |
| 361 | Monuron | 115.00 | 116.60 | 117.20 | -2.20 |
| 362 | 1,4-Dibromobenzene | 73.70 | 71.80 | 71.50 | 2.20 |
| 363 | Pyracene | 89.60 | 92.10 | 91.80 | -2.20 |
| 364 | 2,3-Dihydro-6-nitro-1,4-benzodioxin | 100.60 | 98.90 | 98.30 | 2.30 |
| 365 | 1,2,4-Trichlorodibenzo-p-dioxin | 121.00 | 119.00 | 118.70 | 2.30 |
| 366 | 2,4-Dichloropyrimidine | 76.50 | 74.20 |  | 2.30 |
| 367 | 2,6-Dimethylquinoline | 84.50 | 82.60 | 82.20 | 2.30 |
| 368 | 3-Acetoxyacetophenone | 100.60 | 103.10 | 102.90 | -2.30 |
| 369 | Decane | 80.30 | 82.70 | 82.60 | -2.30 |
| 370 | 1,4-Dihydroxybenzene | 102.00 | 99.80 | 99.70 | 2.30 |
| 371 | Imidazole | 85.40 | 89.30 | 87.70 | -2.30 |
| 372 | trans-Decahydroquinoline | 77.90 | 77.00 | 80.20 | -2.30 |
| 373 | Urea | 95.50 | 93.40 | 93.20 | 2.30 |
| 374 | 3-Anilino-1-phenyl-2-butene-1-one | 126.80 | 124.20 | 124.50 | 2.30 |
| 375 | 6-Chloro-2-pyridone | 91.70 | 94.00 |  | -2.30 |
| 376 | 4-Hydroxybenzamide | 117.80 | 120.30 | 120.10 | -2.30 |
| 377 | 3-t-Butylphenol | 86.00 | 88.00 | 88.30 | -2.30 |
| 378 | 2,3,7,8-Tetrachlorodibenzo-p-dioxin | 124.00 | 126.10 | 126.30 | -2.30 |
| 379 | HMX | 176.10 | 175.40 | 173.70 | 2.40 |
| 380 | 1-Thia-3-azaspiro[5.5]undec-2-en-2-ylamine | 100.70 | 100.10 | 98.30 | 2.40 |
| 381 | 3-Amino-4-methoxybenzoic acid | 130.70 | 128.70 | 128.30 | 2.40 |
| 382 | 1,4-Naphthoquinone | 91.00 | 89.10 | 88.60 | 2.40 |
| 383 | 2,2'-Bipyridine | 81.80 | 83.80 | 84.20 | -2.40 |
| 384 | Fluoranthene | 99.20 | 96.50 | 96.80 | 2.40 |
| 385 | Quinazolin-2,4-dione | 128.30 | 126.80 | 125.90 | 2.40 |
| 386 | 2-Phenylbicyclo[1,1,1]pentane-2-ol | 87.10 | 88.90 | 89.50 | -2.40 |
| 387 | 4-t-Butylphenol | 85.90 | 88.00 | 88.30 | -2.40 |
| 388 | 9-Phenylanthracene | 120.50 | 122.40 | 122.90 | -2.40 |
| 389 | N,N-Diethylthiobenzamide | 91.40 | 92.80 | 93.80 | -2.40 |
| 390 | 9,9'-Bifluorenyl | 132.60 | 133.70 | 135.10 | -2.50 |
| 391 | Pentamethylbenzoic acid | 113.40 | 111.90 | 110.90 | 2.50 |
| 392 | 6-Aminoquinoline | 105.70 | 103.10 | 103.20 | 2.50 |
| 393 | 1,3,7-Trichlorodibenzo-p-dioxin | 116.20 | 119.00 | 118.70 | -2.50 |
| 394 | 2,4-Dichlorophenoxyacetic acid | 125.00 | 123.10 | 122.50 | 2.50 |
| 395 | 3,5-Di-t-butyltoluene | 81.80 | 78.40 | 79.30 | 2.50 |
| 396 | 4-Amino-N-(4-ethylphenyl)benzenesulfonamide | 143.60 | 141.10 | 141.10 | 2.50 |
| 397 | 4-Ethylbenzoic acid | 101.20 | 103.80 | 103.70 | -2.50 |
| 398 | 4-Phenyltoluene | 80.60 | 83.30 | 83.10 | -2.50 |
| 399 | alpha-Phenyl-1-piperidinoacetonitrile | 92.90 | 95.20 | 95.40 | -2.50 |
| 400 | Di-p-tolyl sulfone | 110.00 | 110.50 | 112.50 | -2.50 |
| 401 | Erucic acid | 207.60 | 203.30 | 205.10 | 2.50 |
| 402 | Monomethyl terephthalate | 124.10 | 122.20 | 121.60 | 2.50 |
| 403 | 3,5-Di-t-butylphenol | 97.70 | 99.40 | 100.20 | -2.50 |
| 404 | 1,4-Diaminobenzene | 96.80 | 99.70 | 99.30 | -2.50 |
| 405 | 1,3,5-Trinitroso-1,3,5-triazacyclohexane | 134.30 | 131.70 |  | 2.60 |
| 406 | 1,2,3,7,8,9-Hexachlorodibenzo-p-dioxin | 142.90 | 140.30 | 140.30 | 2.60 |
| 407 | 1-Octanol | 100.40 | 102.40 | 103.00 | -2.60 |
| 408 | Aspirin | 118.70 | 115.70 | 116.10 | 2.60 |
| 409 | Diphenylsulfone | 106.00 | 103.00 | 103.40 | 2.60 |
| 410 | Ethylurea | 99.30 | 101.40 | 101.90 | -2.60 |
| 411 | 1,3-Dithiolan-2-thione | 82.90 | 85.30 | 85.50 | -2.60 |
| 412 | Benzyl phenyl sulfide | 98.40 | 101.50 | 101.00 | -2.60 |
| 413 | 2-Ethylbenzoic acid | 101.10 | 103.80 | 103.70 | -2.60 |
| 414 | 2-Pyridone | 86.60 | 88.70 | 89.20 | -2.60 |
| 415 | Trifluorothymine | 110.80 | 112.00 | 113.40 | -2.60 |
| 416 | Triphenylacetic acid | 152.20 | 148.80 | 149.60 | 2.60 |
| 417 | 1-Phenyl-1,3-butanedione | 91.00 | 94.00 | 93.60 | -2.60 |
| 418 | 3-Carboxyphenol | 118.30 | 116.50 | 115.70 | 2.60 |
| 419 | 1-Adamantylcarboxamide | 108.20 | 110.90 | 110.80 | -2.60 |
| 420 | 2-Picoline N-oxide | 78.20 | 80.30 | 80.80 | -2.60 |
| 421 | 1-Pentadecanoic acid | 156.00 | 158.70 | 158.60 | -2.60 |
| 422 | 1-Acetylvinyl 4-nitrobenzoate | 131.00 | 131.00 | 128.30 | 2.70 |
| 423 | 2,4-Dimethylbenzoic acid | 103.50 | 100.70 | 100.80 | 2.70 |
| 424 | 2-Chlorofluorene | 92.50 | 90.40 | 89.80 | 2.70 |
| 425 | 2-Mercapto-2-thiazoline | 100.50 | 97.80 |  | 2.70 |
| 426 | 6-Chloro-2,4-dinitroaniline | 115.00 | 112.80 | 112.30 | 2.70 |
| 427 | 4-Methylbenzophenone | 97.30 | 99.70 | 100.00 | -2.70 |
| 428 | Anthracene | 98.20 | 95.30 | 95.50 | 2.70 |
| 429 | Flufenamic acid | 121.20 | 123.40 | 123.90 | -2.70 |
| 430 | 9H-Fluoren-9-one | 94.70 | 91.50 | 92.00 | 2.70 |
| 431 | N-Methylnicotinamide | 107.40 | 110.40 | 110.10 | -2.70 |
| 432 | Triptycene | 105.00 | 106.60 | 107.70 | -2.70 |
| 433 | 4-Amino-N-(3-chloro-2-methoxyphenyl)benzenesulfonamide | 147.20 | 149.90 | 149.90 | -2.70 |
| 434 | 1,3,7,9-Tetrachlorodibenzo-p-dioxin | 123.60 | 126.10 | 126.30 | -2.70 |
| 435 | 4-Nitrocatechol | 121.10 | 118.70 | 118.30 | 2.80 |
| 436 | 2,3,5-Trimethylbenzoic acid | 106.70 | 104.40 | 103.90 | 2.80 |
| 437 | 2-Hydroxynicotinic acid | 128.30 | 130.30 | 131.10 | -2.80 |
| 438 | 2,4-Difluoronitrobenzene | 72.80 | 70.30 | 70.00 | 2.80 |
| 439 | 2,8-Dichlorodibenzo-p-dioxin | 109.10 | 111.90 | 111.90 | -2.80 |
| 440 | 2-Hydroxy-6-methylpyridine | 92.00 | 89.80 | 89.20 | 2.80 |
| 441 | Dibenzo[b,d]thiophene | 97.50 | 94.80 | 94.70 | 2.80 |
| 442 | Methyl 4-methylbenzoate | 83.30 | 85.90 | 86.10 | -2.80 |
| 443 | Phenanthrene | 91.60 | 95.30 | 94.40 | -2.80 |
| 444 | 3-Acetylpyrrole | 94.70 | 96.60 | 97.50 | -2.80 |
| 445 | 4-Thianone | 73.40 | 73.80 | 76.20 | -2.80 |
| 446 | 1,1,1-Triphenylethane | 108.60 | 110.50 | 111.40 | -2.80 |
| 447 | 1-(2-(Vinyloxy)ethyl)-1-nitrosohydrazine | 112.10 | 114.90 |  | -2.80 |
| 448 | 2,5-Dichloropyridine | 67.10 | 70.10 | 69.90 | -2.80 |
| 449 | 1,3,5-Trichlorobenzene | 76.60 | 74.50 | 73.80 | 2.80 |
| 450 | 1,3-Benzothiazole-2-thione | 111.00 | 113.80 |  | -2.80 |
| 451 | 9-Methylanthracene | 101.80 | 98.90 | 99.00 | 2.80 |
| 452 | Bromoacetic acid | 83.50 | 86.00 | 86.30 | -2.80 |
| 453 | 1,6-Hexanediamine | 103.30 | 102.60 | 100.50 | 2.80 |
| 454 | 2-Chloro-3-hydroxypyridine | 89.20 | 86.30 | 86.30 | 2.90 |
| 455 | 1,3-Benzodioxole-5-carboxylic acid | 117.20 | 114.10 | 114.30 | 2.90 |
| 456 | 1-Methyladamantane | 67.80 | 65.10 | 64.90 | 2.90 |
| 457 | 1,4-Dioxane-2,5-dione | 87.80 | 85.70 | 84.90 | 2.90 |
| 458 | 4-Methyl-3-nitrobenzoic acid | 118.60 | 115.90 | 115.70 | 2.90 |
| 459 | Benzophenone | 93.10 | 96.00 | 96.00 | -2.90 |
| 460 | (-)-Isopinocampheol | 80.50 | 84.10 | 83.40 | -2.90 |
| 461 | 2,2'-Bithiophene | 85.20 | 87.30 | 88.10 | -2.90 |
| 462 | 2,2'-Dipyridyl N,N'-oxide | 125.00 | 127.60 | 127.90 | -2.90 |
| 463 | 4-Bromoacetophenone | 83.40 | 81.30 | 80.50 | 2.90 |
| 464 | Naphthalene | 76.70 | 73.90 | 73.80 | 2.90 |
| 465 | Propyl 4-hydroxybenzoate | 116.70 | 119.50 | 119.60 | -2.90 |
| 466 | 2,3,5-Trichloropyridine | 74.40 | 77.20 | 77.30 | -2.90 |
| 467 | 2,6-Dimethoxyphenol | 98.40 | 101.00 | 101.30 | -2.90 |
| 468 | 3,5-Diisopropylphenol | 98.24 | 100.50 | 101.20 | -2.96 |
| 469 | 1,3,5,7-Tetroxane | 79.60 | 77.50 | 76.60 | 3.00 |
| 470 | 2-Naphthaleneacetic acid | 124.60 | 121.80 | 121.60 | 3.00 |
| 471 | 2-Phenylquinoline | 105.40 | 102.70 | 102.40 | 3.00 |
| 472 | 2-Iodofluorene | 102.00 | 98.60 | 99.00 | 3.00 |
| 473 | Piperonyl alcohol | 103.70 | 105.90 | 106.70 | -3.00 |
| 474 | 6-Aminopurine | 110.00 | 107.00 |  | 3.00 |
| 475 | Methyl nonadecanoate | 173.30 | 175.20 | 176.30 | -3.00 |
| 476 | Methyl 4-hydroxybenzoate | 108.40 | 105.50 | 105.40 | 3.00 |
| 477 | Naproxen | 132.40 | 135.50 | 135.40 | -3.00 |
| 478 | 2-Chlorobenzophenone | 100.20 | 103.10 | 103.20 | -3.00 |
| 479 | 4-Butoxybenzoic acid | 123.20 | 126.30 | 126.20 | -3.00 |
| 480 | 2,3,5,6-Tetramethylbenzoic acid | 106.10 | 108.20 | 109.10 | -3.00 |
| 481 | 3-Acetoxybenzoic acid | 121.10 | 124.30 | 124.10 | -3.00 |
| 482 | 1-Benzopyran-4-one | 81.29 | 79.10 | 78.20 | 3.09 |
| 483 | Bisphenol A | 137.90 | 135.20 | 134.80 | 3.10 |
| 484 | 1-Piperidinecarboxamide | 100.20 | 97.70 | 97.10 | 3.10 |
| 485 | 2-Phenylbenzimidazole | 123.00 | 118.40 | 119.90 | 3.10 |
| 486 | 2,5-Dimethyl-1-(4-nitrophenyl)pyrrole | 105.80 | 103.50 | 102.70 | 3.10 |
| 487 | 6-Propylthiouracil | 139.70 | 138.80 | 136.60 | 3.10 |
| 488 | Bicyclo[2.2.1]heptane-7-one 2,2-dimethylpropylene ketal | 84.00 | 81.30 | 87.10 | -3.10 |
| 489 | n-Octanoic acid | 113.30 | 110.50 | 110.20 | 3.10 |
| 490 | Heptadecane | 125.10 | 127.20 | 128.20 | -3.10 |
| 491 | Monomethyl isophthalate | 125.60 | 122.20 | 122.50 | 3.10 |
| 492 | N-(2-Chloro-3-pyridinyl)benzene sulfonamide | 115.00 | 117.10 | 118.10 | -3.10 |
| 493 | Triphenylene | 120.10 | 116.60 | 117.00 | 3.10 |
| 494 | Meso-3,4-dicyclohexyl-2,5-dimethylhexan | 112.70 | 116.90 | 115.80 | -3.10 |
| 495 | 1,2,4,6,7,9-Hexachlorodibenzo-p-dioxin | 143.20 | 140.30 | 140.10 | 3.10 |
| 496 | 3-Chloronitrobenzene | 82.50 | 79.20 | 79.30 | 3.20 |
| 497 | 4-Amino-3-methoxybenzoic acid | 132.00 | 128.70 | 128.80 | 3.20 |
| 498 | 4-Methyl-2,5-diisopropylphenol | 108.30 | 104.30 | 105.10 | 3.20 |
| 499 | 2-Pyrazinecarboxylic acid | 103.60 | 101.00 | 100.40 | 3.20 |
| 500 | 3,4-Dinitro-1-fluoropyrazole | 88.30 | 91.50 |  | -3.20 |
| 501 | 2,3,8-Trichlorodibenzofuran | 111.80 | 108.40 | 108.60 | 3.20 |
| 502 | 4-Iodobenzoic acid | 111.20 | 108.50 | 108.00 | 3.20 |
| 503 | Diphenylmethanol | 105.70 | 109.60 | 108.90 | -3.20 |
| 504 | 3,5-Dichloronitrobenzene | 83.20 | 86.40 | 86.40 | -3.20 |
| 505 | Dibenzo-30-crown-10 | 255.70 | 259.00 | 259.00 | -3.30 |
| 506 | 2-Methylpyrazine-5-carboxylic acid | 100.90 | 99.80 | 97.60 | 3.30 |
| 507 | 2-Methylindole | 88.70 | 85.60 | 85.40 | 3.30 |
| 508 | [2,2]Paracyclophane-1-ene | 94.40 | 98.70 | 97.70 | -3.30 |
| 509 | 1,2,3,4-Tetrachlorobenzene | 78.80 | 81.70 | 82.10 | -3.30 |
| 510 | 5-Amino-2-methyltetrazole | 92.00 | 88.70 |  | 3.30 |
| 511 | 2-Hydroxybenzamide | 106.60 | 111.60 | 109.90 | -3.30 |
| 512 | 1-Nitroadamantane | 81.20 | 83.20 | 84.50 | -3.30 |
| 513 | 2,4-Diaminopyrimidine | 106.30 | 102.20 | 103.00 | 3.30 |
| 514 | 3-Methyl-4-nitrobenzoic acid | 119.30 | 115.90 | 116.00 | 3.30 |
| 515 | N-(2-Bromoethyl)phthalimide | 108.70 | 112.00 |  | -3.30 |
| 516 | Benzofuran-2-carboxylic acid | 108.40 | 111.40 | 111.70 | -3.30 |
| 517 | Coumarin | 83.10 | 86.20 | 86.40 | -3.30 |
| 518 | 1,2,3,4,6,7,8-Heptachlorodibenzo-p-dioxin | 149.80 | 147.50 | 146.50 | 3.30 |
| 519 | Di-n-butylsulfone | 100.00 | 102.80 | 103.30 | -3.30 |
| 520 | 1,3-Dinitrobenzene | 87.00 | 91.10 | 90.30 | -3.30 |
| 521 | N,N-Dimethylnicotinamide | 94.00 | 96.90 | 97.30 | -3.30 |
| 522 | N-Methylbenzamide | 101.00 | 104.10 | 104.30 | -3.30 |
| 523 | Tridecanoic acid | 141.00 | 144.90 | 144.30 | -3.30 |
| 524 | Undecanedioic acid | 171.00 | 167.50 | 167.60 | 3.40 |
| 525 | Benz[a]anthracene | 119.50 | 116.60 | 116.10 | 3.40 |
| 526 | 2,3-Dichlorodibenzo-p-dioxin | 108.60 | 111.90 | 112.00 | -3.40 |
| 527 | 2-Aminopyridine | 76.50 | 77.40 | 73.10 | 3.40 |
| 528 | 3,4-Difluoronitrobenzene | 73.80 | 70.30 | 70.40 | 3.40 |
| 529 | Pentafluorophenol | 67.40 | 71.90 | 70.80 | -3.40 |
| 530 | 3',4'-Dichloroacetophenone | 90.80 | 86.30 | 87.40 | 3.40 |
| 531 | 4-t-Butyldiphenyl ether | 100.10 | 103.00 | 103.50 | -3.40 |
| 532 | 1-(4-Chlorophenyl)pyrrole | 88.50 | 91.70 | 91.90 | -3.40 |
| 533 | Nitrofungin | 99.00 | 102.50 | 102.40 | -3.40 |
| 534 | 4-Acetoxyacetophenone | 99.70 | 103.10 | 103.10 | -3.40 |
| 535 | trans-Cinnamic acid | 107.10 | 103.40 | 103.70 | 3.40 |
| 536 | Phthalic acid | 129.80 | 133.20 | 133.20 | -3.40 |
| 537 | 2,4-Dihydroxytoluene | 107.30 | 103.50 | 103.80 | 3.50 |
| 538 | 4-Bromobenzonitrile | 80.80 | 76.70 | 77.30 | 3.50 |
| 539 | 2,5-Dimethyl-1-phenyl-3-pyrrolecarboxaldehyde | 107.60 | 104.10 | 104.10 | 3.50 |
| 540 | 2,3-Dichloroanisole | 83.60 | 79.70 | 80.10 | 3.50 |
| 541 | 2,3-Dichloropyridine | 73.50 | 70.10 | 70.00 | 3.50 |
| 542 | 3-Methyl-2-thiophenecarboxylic acid | 98.00 | 101.20 | 101.50 | -3.50 |
| 543 | 4-Nitrophenol | 92.40 | 95.40 | 95.90 | -3.50 |
| 544 | Azoadamantane N,N'-dioxide | 97.50 | 101.40 | 101.00 | -3.50 |
| 545 | Ibuprofen | 117.10 | 120.40 | 120.60 | -3.50 |
| 546 | p-Tolyl vinyl sulfone | 82.00 | 86.80 | 85.50 | -3.50 |
| 547 | 5,5'-Bis(1-fluoro-3-nitro-1,2,4-triazolyl) | 105.40 | 108.90 |  | -3.50 |
| 548 | N-Methylphthalimide | 91.10 | 94.20 | 94.60 | -3.50 |
| 549 | 1-Piperidinocyclohexanecarbonitrile | 87.80 | 91.30 | 91.30 | -3.50 |
| 550 | 1,2,3,7-Tetrachlorodibenzo-p-dioxin | 129.40 | 126.10 | 125.80 | 3.60 |
| 551 | 2,3-Dichlorodibenzofuran | 105.00 | 101.20 | 101.40 | 3.60 |
| 552 | 4,4'-Di-t-butylbiphenyl | 106.00 | 101.80 | 102.40 | 3.60 |
| 553 | 6-Acetyl-1,4-benzodioxane | 102.50 | 98.80 | 98.90 | 3.60 |
| 554 | 2,6-Difluorophenol | 77.80 | 74.70 | 74.20 | 3.60 |
| 555 | 2,6-Dinitrotoluene | 98.30 | 94.80 | 94.70 | 3.60 |
| 556 | 5-Methyluracil | 126.80 | 122.70 | 123.20 | 3.60 |
| 557 | 4-Nitrotoluene | 79.10 | 75.90 | 75.50 | 3.60 |
| 558 | 2,5-Dihydroxytoluene | 100.40 | 103.50 | 104.00 | -3.60 |
| 559 | 1,3-Dimethyladamantane | 60.60 | 64.00 | 64.20 | -3.60 |
| 560 | 3,5-Dihydroxybenzoic acid | 143.20 | 139.80 | 139.60 | 3.60 |
| 561 | Glutaric acid | 122.70 | 126.20 | 126.30 | -3.60 |
| 562 | 3,5-Bis(trifluoromethyl)-1,2,4-triazole | 74.70 | 76.40 | 78.30 | -3.60 |
| 563 | 3-Acetyl-2-oxazolidinone | 82.36 | 78.90 | 78.70 | 3.66 |
| 564 | 4,4'-(1-Phenylethylidene) biphenol | 162.80 | 159.10 | 159.10 | 3.70 |
| 565 | 1,4-Diphenylnaphthalene | 132.50 | 128.10 | 128.80 | 3.70 |
| 566 | Stearic acid | 183.50 | 179.30 | 179.80 | 3.70 |
| 567 | Xanthone | 105.80 | 102.10 | 102.10 | 3.70 |
| 568 | Benzoyltrifluoroacetone | 87.10 | 83.30 | 83.40 | 3.70 |
| 569 | 4-Amino-N-(3-chloro-4-methylphenyl)benzenesulfonamide | 144.60 | 141.30 | 140.90 | 3.70 |
| 570 | 4-Nitropyridine N-oxide | 108.90 | 105.50 | 105.20 | 3.70 |
| 571 | 9,10-Anthraquinone | 110.90 | 106.60 | 107.20 | 3.70 |
| 572 | N,N-Diethyl-N'-isobutanoylthiourea | 120.80 | 123.50 | 124.50 | -3.70 |
| 573 | 3-Bromobenzoic acid | 99.20 | 102.50 | 102.90 | -3.70 |
| 574 | 3-Thiophenecarboxylic acid | 91.90 | 94.80 | 95.60 | -3.70 |
| 575 | 1,4-Dinitrobenzene | 94.30 | 91.10 | 90.60 | 3.70 |
| 576 | Ethyl trans-2-phenylcyclopropanecarboxylate | 96.90 | 100.70 | 100.60 | -3.70 |
| 577 | o-Anthranilic acid | 111.60 | 107.80 | 107.80 | 3.80 |
| 578 | 2-Iodobenzoic acid | 111.40 | 108.50 | 107.60 | 3.80 |
| 579 | 4,5-Tetramethylene-1,3-dithiolan-2-thione | 103.90 | 101.40 | 100.10 | 3.80 |
| 580 | 2,7-Dimethylphenanthrene | 107.00 | 102.60 | 103.20 | 3.80 |
| 581 | 2-Amino-6-methylbenzoic acid | 116.10 | 111.60 | 112.30 | 3.80 |
| 582 | 2-Methyl-3-nitrobenzoic acid | 119.50 | 115.90 | 115.70 | 3.80 |
| 583 | 3-Methyl-4-nitropyridine-1-oxide | 106.70 | 109.20 | 110.50 | -3.80 |
| 584 | Trimethylacetic acid | 73.20 | 77.00 | 77.00 | -3.80 |
| 585 | 4-Amino-N-(4-chlorophenyl)benzene sulfonamide | 134.10 | 137.60 | 137.90 | -3.80 |
| 586 | Phenylalanine | 160.60 | 162.50 | 164.40 | -3.80 |
| 587 | 1-Phenylpyrrole | 80.80 | 84.60 | 84.60 | -3.80 |
| 588 | o-Terphenyl | 103.00 | 106.70 | 106.80 | -3.80 |
| 589 | Phenoxazine | 101.50 | 105.10 | 105.30 | -3.80 |
| 590 | beta-Resorcylic acid | 127.90 | 131.10 | 131.70 | -3.80 |
| 591 | 2-Bromonitrobenzene | 85.20 | 81.40 | 81.30 | 3.90 |
| 592 | Cyclohexanone oxime | 75.80 | 71.90 |  | 3.90 |
| 593 | Pentafluorobenzoic acid | 91.50 | 88.70 | 87.60 | 3.90 |
| 594 | 2-Iodonitrobenzene | 90.60 | 87.40 | 86.70 | 3.90 |
| 595 | 4-Chloroiodobenzene | 71.90 | 75.60 | 75.80 | -3.90 |
| 596 | 1-Naphthalenemethanol | 102.30 | 106.80 | 106.20 | -3.90 |
| 597 | Tri-t-butylmethane | 55.40 | 57.60 | 59.30 | -3.90 |
| 598 | 1,2,3,4,7-Pentachlorodibenzo-p-dioxin | 137.40 | 133.20 | 133.50 | 3.90 |
| 599 | 2,4-Dinitrobenzoic acid | 135.00 | 131.10 | 131.10 | 3.90 |
| 600 | 2-Naphthol | 94.20 | 98.20 | 98.10 | -3.90 |
| 601 | 5-Phenyltetrazole | 115.00 | 118.40 | 118.90 | -3.90 |
| 602 | Acetanilide | 100.40 | 95.60 | 96.50 | 3.90 |
| 603 | Dibenz[ah]anthracene | 142.00 | 138.00 | 138.10 | 3.90 |
| 604 | Flavanone | 107.20 | 110.90 | 111.10 | -3.90 |
| 605 | 3-Iodophenol | 88.40 | 91.80 | 92.30 | -3.90 |
| 606 | 3-Methyl-3-pyrazoline-5-one | 111.40 | 112.00 | 115.30 | -3.90 |
| 607 | Peroxytridecanoic acid | 142.70 | 139.60 | 138.70 | 4.00 |
| 608 | 5-Methyltetrazole | 96.30 | 94.40 | 92.30 | 4.00 |
| 609 | 2-Thioxo-4-imidazolidinone | 119.10 | 115.70 | 115.10 | 4.00 |
| 610 | N,N-Dimethyl-3-nitroaniline | 92.70 | 89.00 | 88.70 | 4.00 |
| 611 | 2,3,7,8-Tetrachlorodibenzofuran | 119.70 | 115.50 | 115.70 | 4.00 |
| 612 | 2,4,6-Trimethylbenzonitrile N-oxide | 77.50 | 73.50 |  | 4.00 |
| 613 | 2-Methyl-6-nitrobenzoic acid | 120.00 | 115.90 | 116.00 | 4.00 |
| 614 | Carbazole | 97.70 | 94.50 | 93.70 | 4.00 |
| 615 | 2,3,5,6-Tetramethyl-1,4-benzoquinone | 93.20 | 90.80 | 89.20 | 4.00 |
| 616 | Propanamide | 75.00 | 79.90 | 79.00 | -4.00 |
| 617 | 1-Methyl-2-pyrrolecarboxylic acid | 96.20 | 99.20 | 100.20 | -4.00 |
| 618 | 5-Nitroquinoline | 94.20 | 98.90 | 98.20 | -4.00 |
| 619 | 2,6-Dichloropyrazine | 69.90 | 72.80 | 73.90 | -4.00 |
| 620 | Di-t-butylcarbonate | 65.40 | 66.10 | 69.40 | -4.00 |
| 621 | 1,4-Benzoxazin-3-one | 106.40 | 103.00 | 102.30 | 4.10 |
| 622 | 3,4-Dimethylbenzophenone | 107.90 | 103.50 | 103.80 | 4.10 |
| 623 | 2,4,5,7-Tetramethylphenanthrene | 114.00 | 109.90 | 109.90 | 4.10 |
| 624 | 4-Bromotoluene | 62.40 | 66.20 | 66.50 | -4.10 |
| 625 | 2,2,5,7,8-Pentamethylchroman-6-ol | 107.40 | 110.20 | 111.50 | -4.10 |
| 626 | 4-Aminophenol | 103.60 | 99.70 | 99.50 | 4.10 |
| 627 | 2-Coumaranone | 78.70 | 82.90 | 82.80 | -4.10 |
| 628 | 2,6-Di-t-butylnaphthalene | 99.14 | 96.10 | 95.00 | 4.14 |
| 629 | 6-Methyl-2-pyridone | 92.00 | 88.70 | 87.80 | 4.20 |
| 630 | 1-Phenyldodecane | 135.10 | 130.80 | 130.90 | 4.20 |
| 631 | Nonadecane | 143.60 | 139.90 | 139.40 | 4.20 |
| 632 | 2-Furanacrylic acid | 103.30 | 98.60 | 99.10 | 4.20 |
| 633 | 2,3-Dimethylindole | 86.80 | 90.40 | 91.00 | -4.20 |
| 634 | 2-Bromofluorene | 97.20 | 92.60 | 93.00 | 4.20 |
| 635 | Perfluorotridecane | 94.30 | 89.40 | 90.10 | 4.20 |
| 636 | Diethanolamine | 105.90 | 109.40 | 110.10 | -4.20 |
| 637 | 3-Phenylpropanoic acid | 102.40 | 107.00 | 106.60 | -4.20 |
| 638 | epsilon-Aminocaproic acid | 155.00 | 159.70 | 159.20 | -4.20 |
| 639 | 2-Benzoyl-3-phenylquinoxaline N,N'-dioxide | 167.40 | 171.40 | 171.60 | -4.20 |
| 640 | Hemellitic acid | 104.60 | 100.70 | 100.30 | 4.30 |
| 641 | 2,4,6-Trimethylbenzonitrile | 82.90 | 78.70 | 78.60 | 4.30 |
| 642 | RDX | 134.30 | 136.80 | 138.60 | -4.30 |
| 643 | 2,2-Diphenylpropionic acid | 129.00 | 124.90 | 124.70 | 4.30 |
| 644 | 2,3:6,7-Dibenzocycloocta-2,6-dien-1-one | 107.80 | 112.10 | 112.10 | -4.30 |
| 645 | Butyl 4-hydroxybenzoate | 122.40 | 126.30 | 126.70 | -4.30 |
| 646 | 1,2,3-Benzotriazin-4(3H)-one | 111.30 | 108.30 | 107.00 | 4.30 |
| 647 | 3-Ethylbenzoic acid | 99.70 | 103.80 | 104.00 | -4.30 |
| 648 | 5-Chlorobenzofurazan N-oxide | 81.20 | 83.80 | 85.50 | -4.30 |
| 649 | 1,3,5-Trimethylbarbituric acid | 96.10 | 100.50 | 100.40 | -4.30 |
| 650 | Methyl carbamate | 73.60 | 77.40 | 77.90 | -4.30 |
| 651 | N-Methylmaleimide | 73.30 | 76.70 | 77.60 | -4.30 |
| 652 | 2,4,6-Tribromoaniline | 101.00 | 104.30 | 105.30 | -4.30 |
| 653 | 2,4,8-Trichlorodibenzofuran | 112.30 | 108.40 | 108.00 | 4.30 |
| 654 | 2-Amino-3-methoxybenzoic acid | 115.50 | 120.10 | 119.80 | -4.30 |
| 655 | N,N-Dimethylbenzamide | 94.80 | 90.60 | 90.50 | 4.30 |
| 656 | Chlorohydroquinone | 102.93 | 106.90 | 107.30 | -4.37 |
| 657 | Methyl heneicosanoate | 193.50 | 189.00 | 189.10 | 4.40 |
| 658 | 1,3-Diphenyl-3-thioxopropan-1-one | 125.50 | 121.10 |  | 4.40 |
| 659 | Isoxylic acid | 105.00 | 100.70 | 100.60 | 4.40 |
| 660 | 9-Hydroxyfluorene | 109.00 | 105.10 | 104.60 | 4.40 |
| 661 | Valeramide | 89.30 | 93.70 | 93.70 | -4.40 |
| 662 | 4-Pyridinamine | 87.10 | 82.70 | 82.70 | 4.40 |
| 663 | 2-Quinolinecarbonitrile | 94.40 | 89.30 | 90.00 | 4.40 |
| 664 | Acetamide | 77.20 | 73.10 | 72.80 | 4.40 |
| 665 | 2-Isopropylbenzoic acid | 101.00 | 105.20 | 105.40 | -4.40 |
| 666 | 8-Aminocaprylic acid | 170.00 | 173.40 | 174.40 | -4.40 |
| 667 | N-(4-Nitrophenyl)benzenesulfonamide | 132.50 | 126.20 | 128.10 | 4.40 |
| 668 | N-Propylphthalimide | 98.20 | 102.10 | 102.60 | -4.40 |
| 669 | 2,3,4-Trimethylbenzoic acid | 109.30 | 104.40 | 104.80 | 4.50 |
| 670 | 3-Aminobenzamide | 125.30 | 120.20 | 120.80 | 4.50 |
| 671 | (Z)-1-Methyl-4-(2-phenylethenyl)sulphonylbenzene | 116.00 | 113.90 | 111.50 | 4.50 |
| 672 | 1,14-Tetradecanedioic acid | 183.90 | 188.10 | 188.40 | -4.50 |
| 673 | 1,8-Bis((biphenyl)-4-yl)naphthalene | 178.50 | 182.20 | 183.00 | -4.50 |
| 674 | 1-Adamantanecarboxylic acid | 102.50 | 107.10 | 107.00 | -4.50 |
| 675 | Isobutyramide | 86.00 | 81.30 | 81.50 | 4.50 |
| 676 | 4-Amino-N-(2,3-dichlorophenyl)benzene sulfonamide | 141.10 | 144.70 | 145.60 | -4.50 |
| 677 | 5-nitro-1,3-Benzodioxole | 97.40 | 93.00 | 92.90 | 4.50 |
| 678 | 9,9-Dimethylxanthene | 90.00 | 94.70 | 94.50 | -4.50 |
| 679 | Fenbufen | 154.90 | 149.70 | 150.40 | 4.50 |
| 680 | 2,5-Furandione | 70.00 | 75.30 | 74.50 | -4.50 |
| 681 | Monomethyl phthalate | 117.90 | 122.20 | 122.40 | -4.50 |
| 682 | 1,2-Hydrazinedicarboxaldehyde | 100.80 | 105.30 |  | -4.50 |
| 683 | 5-Aminoindazole | 118.40 | 115.10 | 113.80 | 4.60 |
| 684 | 2-Piperidinemethanol | 93.00 | 88.20 | 88.40 | 4.60 |
| 685 | 4-Octylbenzoic acid | 140.70 | 145.10 | 145.30 | -4.60 |
| 686 | Methylurea | 97.10 | 100.40 | 101.70 | -4.60 |
| 687 | 1,3,5,7,9-Pentoxecane | 87.90 | 91.60 | 92.50 | -4.60 |
| 688 | Dibenzo[b,e]pyridine | 97.10 | 100.30 | 101.70 | -4.60 |
| 689 | 3,3,3-Triphenylpropanoic acid | 151.80 | 155.70 | 156.40 | -4.60 |
| 690 | PBN | 86.80 | 91.40 | 91.40 | -4.60 |
| 691 | 4,4'-Dichlorobiphenyl | 91.20 | 95.10 | 95.80 | -4.60 |
| 692 | N,N-(Dimethyl)thiobenzamide | 94.80 | 90.80 | 90.20 | 4.60 |
| 693 | 2,6-Dihydroxytoluene | 99.20 | 103.50 | 103.80 | -4.60 |
| 694 | 1,1,2,2-Tetra-t-butylethane | 74.30 | 70.90 | 69.60 | 4.70 |
| 695 | 1,7-Heptanediamine | 105.30 | 109.40 | 110.00 | -4.70 |
| 696 | Lactide | 91.60 | 87.00 | 86.90 | 4.70 |
| 697 | N,N'-Diethylthiourea | 120.20 | 116.60 | 115.50 | 4.70 |
| 698 | 9,10-Dihydroanthracene | 94.20 | 88.70 | 89.50 | 4.70 |
| 699 | 1,1,1-Trifluoro-4-(2-thienyl)-4-hydroxy-3-buten-2-one | 86.20 | 91.30 | 90.90 | -4.70 |
| 700 | 3,4,5-Trichloroaniline | 92.90 | 97.80 | 97.60 | -4.70 |
| 701 | N-Phenylanthranilic acid | 126.00 | 130.30 | 130.70 | -4.70 |
| 702 | Acetophenone ethylene glycol ketal | 81.88 | 77.40 | 77.10 | 4.78 |
| 703 | Durylic acid | 109.60 | 104.40 | 104.80 | 4.80 |
| 704 | 2,4-Dinitrotoluene | 99.60 | 94.80 | 94.80 | 4.80 |
| 705 | trans-4-Coumaric acid | 130.90 | 126.70 | 126.10 | 4.80 |
| 706 | 1,1,2,2-Tetraphenylethane | 136.80 | 142.70 | 141.60 | -4.80 |
| 707 | 3-Trifluoromethyl-5-methylpyrazole | 78.20 | 73.20 | 73.40 | 4.80 |
| 708 | 4,5-Dichloro-1-methylimidazole | 81.40 | 86.20 |  | -4.80 |
| 709 | 4-Chloronitrobenzene | 74.70 | 79.20 | 79.50 | -4.80 |
| 710 | 4-Chlorophenol | 78.90 | 83.60 | 83.70 | -4.80 |
| 711 | 5-Methoxybenzofurazan N-oxide | 96.00 | 92.00 | 91.20 | 4.80 |
| 712 | Hydroxyacetic acid | 92.60 | 97.40 | 97.40 | -4.80 |
| 713 | 1,2-Dinitrobenzene | 95.50 | 91.10 | 90.70 | 4.80 |
| 714 | Pyridine N-oxide | 81.90 | 86.50 | 86.70 | -4.80 |
| 715 | Quinaldine | 75.10 | 78.80 | 79.90 | -4.80 |
| 716 | 2-Bromobenzonitrile | 81.80 | 76.70 | 77.00 | 4.80 |
| 717 | 6-Nitroquinoline | 103.80 | 98.90 | 99.00 | 4.80 |
| 718 | alpha-Terthienyl | 114.70 | 120.80 | 119.50 | -4.80 |
| 719 | 1-(4-Fluorophenyl)pyrrole | 78.90 | 83.70 | 83.70 | -4.80 |
| 720 | N-Acetyl valinamide | 133.10 | 127.80 | 128.20 | 4.90 |
| 721 | 2-Amino-3-methylbenzoic acid | 107.30 | 111.60 | 112.20 | -4.90 |
| 722 | Dimethylene urethane | 84.20 | 80.60 | 79.30 | 4.90 |
| 723 | Dodecane | 100.20 | 95.40 | 95.30 | 4.90 |
| 724 | 2,5-Dichloro-4-nitroaniline | 114.30 | 109.60 | 109.40 | 4.90 |
| 725 | 3-Bromonitrobenzene | 86.80 | 81.40 | 81.90 | 4.90 |
| 726 | 5,6-Dihydro-6-methyluracil | 118.80 | 113.80 | 113.90 | 4.90 |
| 727 | N-Methylthiourea | 112.90 | 107.50 | 108.00 | 4.90 |
| 728 | [2.2]Paracyclophane | 98.00 | 102.30 | 102.90 | -4.90 |
| 729 | Butanamide | 82.00 | 86.80 | 86.90 | -4.90 |
| 730 | Perfluorotetradecane | 102.40 | 96.40 | 97.50 | 4.90 |
| 731 | Tetramethylthiourea | 84.50 | 87.70 | 89.40 | -4.90 |
| 732 | 4-Cyanopyridine N-oxide | 104.40 | 100.80 | 99.40 | 5.00 |
| 733 | trans-2-Butenedinitrile | 68.60 | 64.20 | 63.60 | 5.00 |
| 734 | 1,3-Diaminobenzene | 95.50 | 99.70 | 100.50 | -5.00 |
| 735 | 2:3,6:7-Dibenzobicyclo[3.2.2]nona-2,6-dien-4-one | 105.40 | 109.80 | 110.40 | -5.00 |
| 736 | 2-Aminophenol | 103.90 | 99.70 | 98.90 | 5.00 |
| 737 | 4-Hydroxybenzaldehyde | 98.20 | 95.00 | 93.20 | 5.00 |
| 738 | 4-Nitroacetophenone | 97.00 | 91.00 | 92.00 | 5.00 |
| 739 | 5-Aminotetrazole | 116.00 | 121.00 |  | -5.00 |
| 740 | Dodecahydrocarbazole | 84.00 | 85.40 | 89.00 | -5.00 |
| 741 | 1,9-Nonanediamine | 119.50 | 123.20 | 124.50 | -5.00 |
| 742 | Caprylamide | 110.00 | 114.30 | 115.00 | -5.00 |
| 743 | Tetraphenylmethane | 140.00 | 134.00 | 135.00 | 5.00 |
| 744 | Triphenylethylene | 112.20 | 117.80 | 117.20 | -5.00 |
| 745 | 3,5-Dimethyl-1-phenyl-4-nitrosopyrazole | 89.70 | 94.70 |  | -5.00 |
| 746 | 2,2,2-Trinitro-1-phenylethane | 84.10 | 88.60 | 89.10 | -5.00 |
| 747 | 5,7-Dibromo-8-hydroxyquinoline | 117.30 | 121.80 | 122.30 | -5.00 |
| 748 | 3-(4-Methoxyphenyl)propionic acid | 124.50 | 119.20 | 119.40 | 5.10 |
| 749 | 4,4'-Dibromobiphenyl | 105.50 | 99.50 | 100.40 | 5.10 |
| 750 | 4-Biphenylmethanol | 107.30 | 112.70 | 112.40 | -5.10 |
| 751 | Benzamide | 102.00 | 97.00 | 96.90 | 5.10 |
| 752 | Methyl 4-chlorobenzoate | 83.40 | 89.30 | 88.50 | -5.10 |
| 753 | Methyl arachidate | 188.00 | 182.10 | 182.90 | 5.10 |
| 754 | Lauric acid | 133.00 | 138.10 | 138.10 | -5.10 |
| 755 | 2-Aminofluorene | 112.30 | 106.50 | 107.20 | 5.10 |
| 756 | n-Decanoic acid | 129.60 | 124.30 | 124.50 | 5.10 |
| 757 | 2-Aminobenzamide | 106.80 | 111.60 | 111.90 | -5.10 |
| 758 | 4-Nitrobenzonitrile | 91.10 | 86.40 | 86.00 | 5.10 |
| 759 | 4-Iodotoluene | 68.00 | 72.30 | 73.10 | -5.10 |
| 760 | 2,5-Dimethyl-p-benzoquinone | 76.99 | 81.20 | 82.10 | -5.11 |
| 761 | 2-Hydroxy-3-methylquinoxaline | 123.00 | 115.20 | 117.80 | 5.20 |
| 762 | 3-Hydroxybiphenyl | 109.00 | 104.20 | 103.80 | 5.20 |
| 763 | Dodecanedioic acid | 179.80 | 174.30 | 174.60 | 5.20 |
| 764 | 3-Benzylphenol | 115.80 | 111.00 | 110.60 | 5.20 |
| 765 | 9(10H)-Anthracenone | 103.30 | 98.40 | 98.10 | 5.20 |
| 766 | 4-Bromonitrobenzene | 86.60 | 81.40 | 81.40 | 5.20 |
| 767 | 5-Hydroxyisoquinoline | 109.60 | 104.50 | 104.40 | 5.20 |
| 768 | N-(4-Isopropylphenylmethylene)benzenamine N-oxide | 127.20 | 122.50 | 122.00 | 5.20 |
| 769 | 1-Cyanoadamantane | 77.00 | 81.40 | 82.20 | -5.20 |
| 770 | 4,7-Dichloroquinoline | 89.50 | 94.20 | 94.70 | -5.20 |
| 771 | 4-t-Octylphenol | 97.90 | 102.60 | 103.10 | -5.20 |
| 772 | 1,3-Dithian-2-thione | 91.40 | 92.20 | 96.60 | -5.20 |
| 773 | 2,3,4-Trichloroaniline | 92.40 | 97.80 | 97.60 | -5.20 |
| 774 | 3,3-Tetramethyleneglutaric acid anhydride | 96.40 | 102.10 | 101.60 | -5.20 |
| 775 | 3-Piperidinemethanol | 95.90 | 91.30 | 90.60 | 5.30 |
| 776 | 1-Methyltetrazole | 88.20 | 86.90 | 82.90 | 5.30 |
| 777 | 1,2,4,5-Tetramethylbenzene | 71.70 | 67.20 | 66.40 | 5.30 |
| 778 | 1-Ethyladamantane | 66.60 | 71.40 | 71.90 | -5.30 |
| 779 | 1-Methylfluorene | 91.20 | 85.60 | 85.90 | 5.30 |
| 780 | 2-Thiophenecarboxylic acid | 91.20 | 96.40 | 96.50 | -5.30 |
| 781 | 4-Bromobenzoic acid | 107.60 | 102.50 | 102.30 | 5.30 |
| 782 | o-Toluamide | 106.10 | 100.70 | 100.80 | 5.30 |
| 783 | trans-1,3,5,7-Tetraoxadecalin | 81.50 | 78.60 | 76.20 | 5.30 |
| 784 | 4-Nitrobenzylidene-t-butylamine | 91.10 | 96.40 | 96.40 | -5.30 |
| 785 | (E)-(2-Phenylethenyl)-sulphonylbenzene | 105.00 | 110.10 | 110.30 | -5.30 |
| 786 | 1,4-Cyclohexanedione | 75.00 | 78.70 | 80.30 | -5.30 |
| 787 | Pentacosane | 174.00 | 178.00 | 179.30 | -5.30 |
| 788 | Carbic anhydride | 97.00 | 91.30 | 91.60 | 5.40 |
| 789 | Quinoxalin-2(1H)-one | 118.50 | 109.20 | 113.10 | 5.40 |
| 790 | Dibenzosuberone | 109.30 | 105.20 | 103.90 | 5.40 |
| 791 | 4-Cyanobenzoic acid | 112.80 | 107.40 | 107.40 | 5.40 |
| 792 | 3,2':5',3-Terthiophene | 122.60 | 117.60 | 117.20 | 5.40 |
| 793 | s-Triazine | 54.20 | 48.80 |  | 5.40 |
| 794 | 2,4,10-Trioxaadamantane | 74.40 | 69.00 |  | 5.40 |
| 795 | 3,4-Dimethylbenzoic acid | 106.40 | 100.70 | 101.00 | 5.40 |
| 796 | 6-Azauracil | 118.10 | 124.20 | 123.50 | -5.40 |
| 797 | Mesitol | 82.80 | 87.70 | 88.20 | -5.40 |
| 798 | Ethyl-(1-thia-3-azaspiro[5.5]undec-2-en-2-yl)amine | 100.80 | 108.10 | 106.20 | -5.40 |
| 799 | N-Ethylphthalimide | 90.90 | 95.20 | 96.30 | -5.40 |
| 800 | Dibenzo[a,h]pyrene | 144.30 | 148.80 | 149.70 | -5.40 |
| 801 | 1,1,1-Trifluoro-4-(2-thienyl)-4-thioxobutane-2-one | 95.10 | 89.60 |  | 5.50 |
| 802 | Phthalonitrile | 86.90 | 81.70 | 81.40 | 5.50 |
| 803 | 2-Methyl-3,5-diisopropylphenol | 109.20 | 104.30 | 103.70 | 5.50 |
| 804 | 4-Chlorobenzophenone | 108.20 | 103.10 | 102.70 | 5.50 |
| 805 | (E)-1-Methyl-4-(2-phenylethenyl)sulphonylbenzene | 108.00 | 113.90 | 113.50 | -5.50 |
| 806 | 2,7-dimethylquinoline | 87.50 | 82.60 | 82.00 | 5.50 |
| 807 | 2-Thiopheneacetic acid | 97.50 | 103.30 | 103.00 | -5.50 |
| 808 | 4,7-Phenanthroline | 111.00 | 106.60 | 105.50 | 5.50 |
| 809 | 4-Acetylanisole | 89.70 | 84.30 | 84.20 | 5.50 |
| 810 | 7-Chloro-5-iodo-8-quinolinol | 131.00 | 125.60 | 125.50 | 5.50 |
| 811 | Nonanedioic acid | 159.90 | 153.70 | 154.40 | 5.50 |
| 812 | Benzo[ghi]perylene | 132.90 | 138.20 | 138.40 | -5.50 |
| 813 | Dimethylacetamide | 60.90 | 66.70 | 66.40 | -5.50 |
| 814 | Diphenylnitrone | 115.00 | 110.40 | 109.50 | 5.50 |
| 815 | Phenacetin | 120.00 | 115.00 | 114.50 | 5.50 |
| 816 | 1,11-Undecanediamine | 132.80 | 137.00 | 138.30 | -5.50 |
| 817 | 6-Chloro-2-hydroxypyridine | 91.70 | 94.40 | 97.20 | -5.50 |
| 818 | N-(4-Methoxyphenyl)-3-aza-1-thiaspiro[5.5]undec-2-en-2-amine | 139.10 | 134.90 | 133.60 | 5.50 |
| 819 | N-Benzoyl-N-methylbenzamide | 120.10 | 126.40 | 125.60 | -5.50 |
| 820 | Benzo[e]pyrene | 123.00 | 127.40 | 128.60 | -5.60 |
| 821 | 1,4-Dioxane-2,6-dione | 90.50 | 85.10 | 84.90 | 5.60 |
| 822 | Dimethyl pyridine-2,6-dicarboxylate | 113.50 | 107.80 | 107.90 | 5.60 |
| 823 | 2,3-Dimethylquinoxaline | 87.80 | 82.70 | 82.20 | 5.60 |
| 824 | 1,4-Dithiane | 68.90 | 68.80 | 74.50 | -5.60 |
| 825 | 2-Benzoxazolone | 97.60 | 93.80 | 92.00 | 5.60 |
| 826 | Dimethyl fumarate | 88.40 | 93.70 | 94.00 | -5.60 |
| 827 | o-Anisic acid | 91.70 | 96.90 | 97.30 | -5.60 |
| 828 | 1,3-Dihydroxynaphthalene | 116.00 | 121.50 | 121.60 | -5.60 |
| 829 | 1,3-Benzodioxol-5-ol | 92.20 | 97.40 | 97.80 | -5.60 |
| 830 | Tetrazole | 85.00 | 89.50 | 90.60 | -5.60 |
| 831 | 1,3,5-Tri-t-butylbenzene | 81.20 | 85.80 | 86.80 | -5.60 |
| 832 | Methyl 2-thiopheneacetate | 98.00 | 92.30 | 92.30 | 5.70 |
| 833 | Isonicotinamide N-oxide | 125.30 | 130.30 | 131.00 | -5.70 |
| 834 | 2-Hydroxydiphenylmethane | 116.60 | 111.00 | 110.90 | 5.70 |
| 835 | 2-Furancarboxylic acid hydrazide | 99.00 | 110.50 | 104.70 | -5.70 |
| 836 | 2,4-Dinitro-2,4-diazapentane | 102.50 | 108.20 | 108.20 | -5.70 |
| 837 | 1,1,1,2,2-Pentanitropropane | 77.40 | 83.80 | 83.10 | -5.70 |
| 838 | 2-Biphenyl methanol | 107.10 | 112.70 | 112.80 | -5.70 |
| 839 | 1,3,5-Trimethyl-2-nitrobenzene | 78.60 | 83.40 | 84.30 | -5.70 |
| 840 | 9-Xanthenecarboxamide | 130.00 | 135.30 | 135.70 | -5.70 |
| 841 | 9-Heptadecanol | 151.70 | 158.10 | 157.50 | -5.80 |
| 842 | Methacrylamide | 89.40 | 84.90 | 83.60 | 5.80 |
| 843 | 2,6-Dichloropyridine | 72.00 | 66.50 | 66.20 | 5.80 |
| 844 | 2,7-Dichlorofluorene | 102.50 | 97.50 | 96.70 | 5.80 |
| 845 | 3-Furancarboxylic acid | 87.10 | 82.00 | 81.30 | 5.80 |
| 846 | 3-Methoxy-4-nitrobenzoic acid | 131.00 | 124.40 | 125.20 | 5.80 |
| 847 | 4-Phenylpyridine | 81.40 | 87.20 | 87.20 | -5.80 |
| 848 | Benzo[k]fluoranthene | 124.20 | 117.90 | 118.40 | 5.80 |
| 849 | beta-Cyanopropiophenone | 102.00 | 96.30 | 96.20 | 5.80 |
| 850 | p-Bromoacetanilide | 110.00 | 104.90 | 104.20 | 5.80 |
| 851 | Piperazine | 66.80 | 61.30 | 61.00 | 5.80 |
| 852 | 3-Hydroxypyridine | 88.30 | 82.80 | 82.50 | 5.80 |
| 853 | Flavone | 108.20 | 114.20 | 114.00 | -5.80 |
| 854 | 4-Methylphenanthrene | 93.40 | 98.90 | 99.20 | -5.80 |
| 855 | 3-Hydroxy-2-pyridone | 109.10 | 113.90 | 114.90 | -5.80 |
| 856 | Picolinamide | 93.10 | 98.40 | 98.90 | -5.80 |
| 857 | 2-Benzyl-2-fluoro-1,3-diphenylpropane | 127.50 | 133.30 |  | -5.80 |
| 858 | Oxindole | 99.70 | 94.20 | 93.80 | 5.90 |
| 859 | Vanillin isoniazid | 182.20 | 177.80 | 176.30 | 5.90 |
| 860 | 3,4,5-Trimethylbenzoic acid | 111.00 | 104.40 | 105.10 | 5.90 |
| 861 | 3-Hydroxybenzaldehyde | 100.10 | 95.00 | 94.20 | 5.90 |
| 862 | 2-Phenylindole | 114.40 | 109.50 | 108.50 | 5.90 |
| 863 | 4-Methylpyridine N-oxide | 85.30 | 90.30 | 91.20 | -5.90 |
| 864 | 1,2,7,8-Tetrachlorodibenzofuran | 120.60 | 115.50 | 114.70 | 5.90 |
| 865 | Inositol | 181.00 | 185.00 | 186.90 | -5.90 |
| 866 | 1,4-Benzoquinone | 66.70 | 71.60 | 72.60 | -5.90 |
| 867 | 7-Bromo-5-chloro-8-hydroxyquinoline | 113.20 | 119.60 | 119.10 | -5.90 |
| 868 | 4-Hydroxybiphenyl | 109.80 | 104.20 | 103.80 | 6.00 |
| 869 | Isatoic anhydride | 115.60 | 110.50 | 109.60 | 6.00 |
| 870 | Diphenylacetic acid | 131.70 | 125.40 | 125.70 | 6.00 |
| 871 | 1-Methyl-4-(1-methylethenylsulphonyl)benzene | 89.00 | 93.20 | 95.00 | -6.00 |
| 872 | 5-Acetyl-2-thiophenecarboxylic acid | 123.50 | 117.90 | 117.50 | 6.00 |
| 873 | N,N'-Dibutylthiourea | 141.00 | 144.10 | 147.00 | -6.00 |
| 874 | Propylurea | 101.40 | 108.30 | 107.40 | -6.00 |
| 875 | 6-Chloroquinoline | 80.80 | 87.00 | 86.80 | -6.00 |
| 876 | 2-Methylnaphthalene | 71.30 | 77.60 | 77.30 | -6.00 |
| 877 | 2,2,4,4-Tetramethyl-1,3-cyclobutanedione | 72.20 | 66.70 | 66.10 | 6.10 |
| 878 | 2-Amino-4-methylpyrimidine | 88.50 | 83.20 | 82.40 | 6.10 |
| 879 | 2-Bromobenzoic acid | 108.50 | 102.50 | 102.40 | 6.10 |
| 880 | 2-Fluoro-1,2,3-triphenylpropane | 132.50 | 126.40 |  | 6.10 |
| 881 | beta-Alanine | 134.00 | 139.00 | 140.10 | -6.10 |
| 882 | Benzil | 106.30 | 111.10 | 112.40 | -6.10 |
| 883 | N,N'-Dinitrosopiperazine | 101.00 | 94.90 |  | 6.10 |
| 884 | Nicotinic acid | 105.20 | 99.50 | 99.10 | 6.10 |
| 885 | Pivalamide | 86.60 | 80.80 | 80.50 | 6.10 |
| 886 | Bicyclo[2.2.2]oct-5-ene-2,2,3,3-tetracarbonitrile | 112.00 | 107.00 | 105.80 | 6.20 |
| 887 | 3,5-Dichloropyridine | 67.30 | 73.70 | 73.50 | -6.20 |
| 888 | 1,4-Dinitropiperazine | 102.90 | 98.20 | 96.70 | 6.20 |
| 889 | 2-Chlorodibenzo-p-dioxin | 98.10 | 104.70 | 104.30 | -6.20 |
| 890 | 3-Chlorophenol | 76.90 | 83.60 | 83.10 | -6.20 |
| 891 | Acenaphthylene | 73.00 | 79.40 | 79.20 | -6.20 |
| 892 | Diphenyldisulfide | 94.90 | 94.90 | 88.70 | 6.20 |
| 893 | Hexamethylbenzene | 81.40 | 74.50 | 75.20 | 6.20 |
| 894 | 1-Phthalazinone | 107.40 | 112.40 | 113.60 | -6.20 |
| 895 | 2,3-Dihydroxypyridine | 109.10 | 114.20 | 115.30 | -6.20 |
| 896 | Pyrazinamide | 112.60 | 104.70 | 106.30 | 6.30 |
| 897 | 1,3,7,9-Tetrachlorodibenzofuran | 122.00 | 115.50 | 115.70 | 6.30 |
| 898 | 2-Butenyl p-tolyl sulphone | 108.00 | 103.10 | 101.70 | 6.30 |
| 899 | 4-Benzylphenol | 117.50 | 111.00 | 111.20 | 6.30 |
| 900 | Phenol | 69.70 | 76.50 | 76.00 | -6.30 |
| 901 | 1,1,1,4,4-Pentanitro-2,2-dimethylpentane | 103.80 | 98.40 | 97.50 | 6.30 |
| 902 | Benzoyl(cyclohexyloxy)-carbonylperoxide | 96.20 | 99.40 | 102.50 | -6.30 |
| 903 | 2-Methylimidazole | 88.40 | 94.20 | 94.70 | -6.30 |
| 904 | 4-Bromoaniline | 79.30 | 85.70 | 85.70 | -6.40 |
| 905 | 4-Chloro-2-nitrophenol | 87.60 | 93.90 | 94.00 | -6.40 |
| 906 | N-Methylphenothiazine | 103.60 | 99.20 | 97.20 | 6.40 |
| 907 | 4-Nitroaniline | 101.40 | 95.40 | 95.00 | 6.40 |
| 908 | Benzanilide | 125.40 | 119.50 | 119.00 | 6.40 |
| 909 | 1,2,4,7,8-Pentachlorodibenzofuran | 128.40 | 122.60 | 122.00 | 6.40 |
| 910 | 2,5-Dimethyl-3-furancarboxylic acid | 99.00 | 104.40 | 105.40 | -6.40 |
| 911 | 2-Adamantanol | 99.90 | 93.30 | 93.50 | 6.40 |
| 912 | 4-Isopropylbenzoic acid | 99.00 | 105.20 | 105.40 | -6.40 |
| 913 | 4-Aminobutyric acid | 140.00 | 145.90 | 146.40 | -6.40 |
| 914 | 1,2,3-Benzenetriol | 116.90 | 123.10 | 123.30 | -6.40 |
| 915 | 2,3-Difluorophenol | 68.20 | 74.70 | 74.60 | -6.40 |
| 916 | N-Methyl-1,8-Naphthalimide | 109.70 | 115.90 | 116.10 | -6.40 |
| 917 | Adamantane 1,3-dinitrate | 105.90 | 109.20 | 112.30 | -6.40 |
| 918 | t-Butylhydroquinone | 104.40 | 111.20 | 110.80 | -6.40 |
| 919 | Triphenylmethanol | 124.30 | 130.50 | 130.70 | -6.40 |
| 920 | Dibenzo[ae]pyrene | 155.20 | 148.80 | 148.70 | 6.50 |
| 921 | 1,3-Dinitroadamantane | 103.80 | 95.00 | 97.30 | 6.50 |
| 922 | 2,5-Dimethoxybenzoic acid | 116.10 | 109.10 | 109.60 | 6.50 |
| 923 | 2,7-Di-t-butyl-9,9-dimethylxanthene | 123.90 | 117.70 | 117.40 | 6.50 |
| 924 | cis-2-Methoxycinnamic acid | 121.70 | 115.70 | 115.20 | 6.50 |
| 925 | Heptacosane | 196.00 | 190.70 | 189.50 | 6.50 |
| 926 | 2-Methyl-3-(phenylmethyl)quinoxaline-1,4-dioxide | 146.60 | 139.20 | 140.10 | 6.50 |
| 927 | Glycine | 138.10 | 132.10 | 131.60 | 6.50 |
| 928 | 2-Hydroxypyridine | 86.60 | 90.90 | 93.10 | -6.50 |
| 929 | Chromone-3-carboxylic acid | 113.80 | 120.10 | 120.30 | -6.50 |
| 930 | 4-Methyldiadamantane | 79.41 | 74.00 | 72.90 | 6.51 |
| 931 | Bisphenol F | 140.80 | 134.30 | 134.20 | 6.60 |
| 932 | 2,2-Diphenyl-dimethoxymethane | 103.90 | 99.20 | 97.30 | 6.60 |
| 933 | 3-Chlorobenzophenone | 110.40 | 103.10 | 103.80 | 6.60 |
| 934 | 4-Methoxybenzoic acid | 112.50 | 105.50 | 105.90 | 6.60 |
| 935 | 4-Propoxybenzoic acid | 126.00 | 119.50 | 119.40 | 6.60 |
| 936 | Dimethylterephthalate | 104.90 | 111.20 | 111.50 | -6.60 |
| 937 | 1-Naphthol | 91.50 | 98.20 | 98.10 | -6.60 |
| 938 | 2,5-Difluorophenol | 68.00 | 74.70 | 74.60 | -6.60 |
| 939 | Methyl 4-methoxybenzoate | 87.70 | 94.50 | 94.30 | -6.60 |
| 940 | 2,4-Dichloroaniline | 84.70 | 90.70 | 91.30 | -6.60 |
| 941 | 4-Pentylbenzoic acid | 118.20 | 124.50 | 124.80 | -6.60 |
| 942 | 1,2-Dihydronaphthalene | 65.30 | 71.70 | 72.00 | -6.70 |
| 943 | 2-Chloronaphthalene | 75.70 | 82.00 | 82.40 | -6.70 |
| 944 | o-Cresol | 73.70 | 80.20 | 80.40 | -6.70 |
| 945 | 3-Nitrobenzonitrile | 92.80 | 86.40 | 86.10 | 6.70 |
| 946 | n-Butylcarbamate | 92.90 | 98.20 | 99.60 | -6.70 |
| 947 | 1,3-Dithiane | 69.90 | 76.60 |  | -6.70 |
| 948 | 2,2',2''-Tripyridine | 97.10 | 108.00 | 103.80 | -6.70 |
| 949 | 2,4-Dinitroimidazole | 112.10 | 118.80 |  | -6.70 |
| 950 | Coronene | 142.60 | 149.10 | 149.30 | -6.70 |
| 951 | Tris(2,2,2-trinitroethyl)orthoformate | 150.00 | 156.70 |  | -6.70 |
| 952 | 1-Nonadecanoic acid | 192.00 | 186.20 | 185.20 | 6.80 |
| 953 | 2-Cyanobenzoic acid | 114.60 | 107.40 | 107.80 | 6.80 |
| 954 | 3,5-Dimethylpyridine N-oxide | 100.90 | 94.00 | 94.10 | 6.80 |
| 955 | 3-Amino-1-phenyl-2-buten-1-one | 109.40 | 101.70 | 102.60 | 6.80 |
| 956 | 8-Nitroquinoline | 106.70 | 98.90 | 99.90 | 6.80 |
| 957 | 2,3-Dihydrothiophene 1,1-dioxide | 61.50 | 67.30 | 68.30 | -6.80 |
| 958 | 2-Phenylnaphthalene | 107.60 | 101.00 | 100.80 | 6.80 |
| 959 | 4-t-Pentylphenol | 88.30 | 94.80 | 95.10 | -6.80 |
| 960 | 4-t-Butylbiphenyl | 98.00 | 90.70 | 91.20 | 6.80 |
| 961 | Malonamide | 126.40 | 120.00 | 119.60 | 6.80 |
| 962 | 4-Diphenylcarboxylic acid | 127.50 | 120.90 | 120.70 | 6.80 |
| 963 | Pentadecane | 107.80 | 114.50 | 114.60 | -6.80 |
| 964 | 1,5-Dimethyl-2-pyrrolecarbonitrile | 78.30 | 73.50 | 71.50 | 6.80 |
| 965 | 2,5-Dimethylhydroquinone | 100.80 | 107.30 | 107.60 | -6.80 |
| 966 | 2,4-Dibromoaniline | 88.00 | 95.00 | 94.80 | -6.80 |
| 967 | 2,6-Pyridinedicarboxylic acid | 137.10 | 129.80 | 130.20 | 6.90 |
| 968 | 4-Methoxy-3-nitrobenzoic acid | 131.20 | 124.40 | 124.30 | 6.90 |
| 969 | 5,5-Dimethyl-1,3-cyclohexanedione | 99.80 | 93.00 | 92.90 | 6.90 |
| 970 | 5-Chloro-6-hydroxynicotinic acid | 151.30 | 146.00 | 144.40 | 6.90 |
| 971 | N,N'-Trimethyleneurea | 113.40 | 104.40 | 106.50 | 6.90 |
| 972 | 1,3-Dimethylbarbituric acid | 92.30 | 99.10 | 99.20 | -6.90 |
| 973 | 1,2,3,4-Tetrahydrodibenzothiophene | 107.30 | 101.00 | 100.40 | 6.90 |
| 974 | 2,3,4,6,7,8-Hexachlorodibenzofuran | 136.80 | 129.70 | 129.90 | 6.90 |
| 975 | 2,4-Di-t-butylphenol | 92.90 | 99.40 | 99.80 | -6.90 |
| 976 | 3,4-Dinitropyrazole | 112.50 | 119.40 |  | -6.90 |
| 977 | 4-Iodoacetophenone | 92.80 | 87.40 | 85.90 | 6.90 |
| 978 | 2,4,6-Trichloronitrobenzene | 86.70 | 93.50 | 93.60 | -6.90 |
| 979 | 4-((4-Nitrophenyl)amino)pent-3-ene-2-one | 121.90 | 127.90 | 128.80 | -6.90 |
| 980 | sec-Butylurea | 101.90 | 106.60 | 108.80 | -6.90 |
| 981 | 1,1,2,2-Tetrafluoro-1,2-diphenylethane | 101.80 | 95.00 | 94.80 | 7.00 |
| 982 | 2,4'-Dichloroacetophenone | 97.80 | 90.80 |  | 7.00 |
| 983 | (2-Pyridyl)acetamide | 103.80 | 96.70 | 96.80 | 7.00 |
| 984 | 3-Coumaranone | 85.80 | 79.40 | 78.80 | 7.00 |
| 985 | 5-Phenyl-1,2-dithiole-3-thione | 123.30 | 116.30 |  | 7.00 |
| 986 | Thiourea | 107.60 | 100.40 | 100.60 | 7.00 |
| 987 | (E)-1-Methyl-4-(1-propenyl-sulphonyl)-benzene | 84.00 | 89.90 | 91.00 | -7.00 |
| 988 | 1,2,3,4,6,8-Hexachlorodibenzofuran | 137.00 | 129.70 | 130.00 | 7.00 |
| 989 | 1,3,5-Trimethyl-4-nitrosopyrazole | 88.00 | 91.00 | 95.00 | -7.00 |
| 990 | 2,5-Dibromonitrobenzene | 97.00 | 90.70 | 90.00 | 7.00 |
| 991 | 4-Iodoaniline | 84.80 | 91.70 | 91.80 | -7.00 |
| 992 | 5-Nitroindole | 110.50 | 104.50 | 103.50 | 7.00 |
| 993 | 9-Fluorenylmethanol | 118.80 | 112.80 | 111.80 | 7.00 |
| 994 | Benzimidazole | 94.30 | 89.50 | 87.30 | 7.00 |
| 995 | Octachlorodibenzofuran | 149.40 | 144.00 | 142.40 | 7.00 |
| 996 | 2,4,6-Trimethylphenyl isocyanide | 66.40 | 73.40 |  | -7.00 |
| 997 | 4-Ethylphenol | 80.33 | 87.10 | 87.40 | -7.07 |
| 998 | 4-Methoxy-2-nitrophenol | 90.80 | 99.10 | 97.90 | -7.10 |
| 999 | 6-Nitroindazole | 117.00 | 110.70 | 109.90 | 7.10 |
| 1000 | 4-Methyl-3,5-diisopropylphenol | 110.80 | 104.30 | 103.70 | 7.10 |
| 1001 | 1,2,4,6,8,9-Hexachlorodibenzofuran | 136.50 | 129.70 | 129.30 | 7.20 |
| 1002 | 5,6-Dihydro-5-methyluracil | 124.00 | 116.90 | 116.80 | 7.20 |
| 1003 | epsilon-Caprolactam | 89.80 | 96.70 | 97.00 | -7.20 |
| 1004 | p-Terphenyl | 113.00 | 106.70 | 105.80 | 7.20 |
| 1005 | 4-Nitrobenzophenone | 122.30 | 114.90 | 115.10 | 7.20 |
| 1006 | 2,2-Dimethyladamantane | 73.60 | 65.90 | 66.40 | 7.20 |
| 1007 | 4-Iodonitrobenzene | 94.60 | 87.40 | 87.40 | 7.20 |
| 1008 | Adamantane 1,3,5,7-tetranitrate | 150.20 | 147.00 | 143.00 | 7.20 |
| 1009 | 4-Butylbenzoic acid | 110.50 | 117.60 | 117.70 | -7.20 |
| 1010 | Crotonic acid | 72.20 | 79.50 | 79.40 | -7.20 |
| 1011 | 2,4,6-Trimethoxybenzonitrile N-oxide | 91.90 | 99.10 |  | -7.20 |
| 1012 | Ferulic acid | 131.80 | 139.00 | 139.10 | -7.30 |
| 1013 | 7-Methoxy-3,4-dihyro-1(2H)-naphthalenone | 103.10 | 96.70 | 95.80 | 7.30 |
| 1014 | 3-Amino-5-methoxybenzoic acid | 136.10 | 128.70 | 128.80 | 7.30 |
| 1015 | 2,3-Pyrazinedicarbonitrile | 89.10 | 84.60 | 81.80 | 7.30 |
| 1016 | 2-Fluoronitrobenzene | 78.40 | 71.20 | 71.10 | 7.30 |
| 1017 | 2-Methyl-2,3,3-trinitropentane | 92.00 | 86.00 | 84.70 | 7.30 |
| 1018 | 2-Tetradecanone | 130.90 | 123.80 | 123.60 | 7.30 |
| 1019 | p-Cresol | 73.10 | 80.20 | 80.40 | -7.30 |
| 1020 | 4-Dimethylaminopyridine | 82.90 | 76.40 | 75.60 | 7.30 |
| 1021 | Chrysene | 123.40 | 116.60 | 116.10 | 7.30 |
| 1022 | 1,2,3,4-Tetraphenylbenzene | 154.10 | 160.90 | 161.40 | -7.30 |
| 1023 | 4-Nitrobenzoic acid | 119.70 | 112.10 | 112.30 | 7.40 |
| 1024 | 1,2,3,7,8-Pentachlorodibenzofuran | 129.00 | 122.60 | 121.60 | 7.40 |
| 1025 | 1,2,3-Trimethoxybenzene | 98.00 | 90.00 | 90.60 | 7.40 |
| 1026 | 2-Methyl-3-hydroxypyridine | 89.30 | 81.70 | 81.90 | 7.40 |
| 1027 | Nortricyclene | 39.20 | 47.10 | 46.60 | -7.40 |
| 1028 | Basketene | 55.30 | 48.50 | 47.90 | 7.40 |
| 1029 | 2-Cyanoadamantane | 75.80 | 83.30 | 83.20 | -7.40 |
| 1030 | 4,5-Tetramethylene-1,2-dithiole-3-thione | 105.30 | 112.70 |  | -7.40 |
| 1031 | 2,3-Diazabicyclo[2.2.1]hept-2-ene | 55.30 | 61.20 | 62.70 | -7.40 |
| 1032 | 4-Fluorobenzamide | 102.80 | 96.10 | 95.40 | 7.40 |
| 1033 | 6,6'-Diphenylfulvene | 100.00 | 107.30 | 107.40 | -7.40 |
| 1034 | 4-N,N-diethylamino-7-nitrobenzofurazan | 139.20 | 131.70 |  | 7.50 |
| 1035 | Dimethylmalonic acid | 111.70 | 104.60 | 104.20 | 7.50 |
| 1036 | 1,2,3,4,6,7,8-Heptachlorodibenzofuran | 144.60 | 136.80 | 137.10 | 7.50 |
| 1037 | 1,2,3,6,7,8-Hexachlorodibenzofuran | 137.10 | 129.70 | 129.60 | 7.50 |
| 1038 | 1,3,6,8-Tetrachlorodibenzofuran | 122.40 | 115.50 | 114.90 | 7.50 |
| 1039 | 2,3,4,7,8-Pentachlorodibenzofuran | 130.40 | 122.60 | 122.90 | 7.50 |
| 1040 | 3-Hydroxybenzylalcohol | 116.00 | 108.40 | 108.50 | 7.50 |
| 1041 | 4-Dimethylaminonitrosobenzene | 82.00 | 74.90 | 74.50 | 7.50 |
| 1042 | 8-Quinolinol | 96.00 | 103.20 | 103.50 | -7.50 |
| 1043 | 2-Phenylglycine | 165.00 | 155.60 | 157.50 | 7.50 |
| 1044 | Benzo[a]pyrene | 120.50 | 127.40 | 128.00 | -7.50 |
| 1045 | 2-(2,4-Dichlorophenoxy)propanoic acid | 116.00 | 123.70 | 123.50 | -7.50 |
| 1046 | N-Benzyl-pivalophenone imine | 109.70 | 114.30 | 117.20 | -7.50 |
| 1047 | Ethylcarbamate | 77.70 | 84.50 | 85.20 | -7.50 |
| 1048 | 1,7-Phenanthroline | 99.90 | 106.60 | 107.40 | -7.50 |
| 1049 | 1-(2-Aminophenyl)pyrrole | 98.80 | 107.80 | 106.30 | -7.50 |
| 1050 | Oxalic acid | 97.91 | 105.50 | 105.50 | -7.59 |
| 1051 | 4-Octyloxybenzoic acid | 161.40 | 153.90 | 153.80 | 7.60 |
| 1052 | 1,3,5,6-Tetramethyluracil | 101.70 | 95.30 | 94.10 | 7.60 |
| 1053 | 2,5-Norbornadiene | 44.90 | 52.20 | 52.50 | -7.60 |
| 1054 | 6-Aminoindazole | 122.00 | 115.10 | 114.40 | 7.60 |
| 1055 | 2,11-Dicyclohexyldodecane | 147.30 | 153.20 | 154.90 | -7.60 |
| 1056 | 1-Methylcyclopentanol | 67.00 | 74.20 | 74.60 | -7.60 |
| 1057 | m-Toluamide | 107.80 | 100.70 | 100.20 | 7.60 |
| 1058 | Ethyl(1,1-dimethylpropyl)malononitrile | 76.20 | 83.20 | 83.80 | -7.60 |
| 1059 | 4,4'-Dichlorobenzophenone | 117.50 | 110.20 | 109.80 | 7.70 |
| 1060 | 4-Piperidinemethanol | 98.30 | 91.30 | 90.60 | 7.70 |
| 1061 | 5-Nitroindoline | 109.80 | 102.10 | 102.10 | 7.70 |
| 1062 | 4,4'-Dimethylbiphenyl | 95.10 | 87.00 | 87.40 | 7.70 |
| 1063 | 2-Fluorobenzonitrile | 59.10 | 66.50 | 66.80 | -7.70 |
| 1064 | 1,3,5-Trioxane | 56.60 | 63.40 | 64.30 | -7.70 |
| 1065 | 1-(4-Hydroxyphenyl)butan-1-one | 116.70 | 109.10 | 109.00 | 7.70 |
| 1066 | 3,5-Di-t-butylbenzoic acid | 108.30 | 116.20 | 116.00 | -7.70 |
| 1067 | 3-Amino-2-methylbenzoic acid | 127.80 | 120.20 | 120.10 | 7.70 |
| 1068 | Biphenylene | 83.80 | 75.20 | 76.10 | 7.70 |
| 1069 | Dimethyl oxalate | 75.30 | 83.50 | 83.00 | -7.70 |
| 1070 | Isoborneol | 74.70 | 82.20 | 82.40 | -7.70 |
| 1071 | 5-Aminouracil | 138.10 | 146.00 | 145.80 | -7.70 |
| 1072 | 3-Methyl-2-nitrobenzoic acid | 124.40 | 115.90 | 116.60 | 7.80 |
| 1073 | 3,3'-Bithiophene | 89.20 | 84.10 | 81.40 | 7.80 |
| 1074 | 4-N,N-Dimethylamino-7-nitrobenzofurazan | 134.20 | 129.70 | 126.40 | 7.80 |
| 1075 | 1,2,3,7-Tetrachlorodibenzofuran | 122.90 | 115.50 | 115.10 | 7.80 |
| 1076 | 3-Nitrobenzamide | 108.80 | 115.90 | 116.60 | -7.80 |
| 1077 | Benzene | 44.40 | 52.60 | 52.20 | -7.80 |
| 1078 | Dibenzoylmethane | 115.70 | 122.70 | 123.50 | -7.80 |
| 1079 | N,N'-Dimethylurea | 98.30 | 107.50 | 106.10 | -7.80 |
| 1080 | 1,2,3,4,7,8-Hexachlorodibenzofuran | 137.40 | 129.70 | 129.60 | 7.80 |
| 1081 | 4-Nitrophthalic anhydride | 103.50 | 111.70 | 111.30 | -7.80 |
| 1082 | 1,3,6,8-Tetrachlorodibenzo-p-dioxin | 118.60 | 126.10 | 126.50 | -7.90 |
| 1083 | 5-Nitro-8-hydroxyquinoline | 114.10 | 122.10 | 122.00 | -7.90 |
| 1084 | 3-Methoxycarbonyl-2-methoxyquinoxaline 1-oxide | 129.20 | 129.50 | 137.10 | -7.90 |
| 1085 | N,N-Diisobutyl-N'-furoylthiourea | 141.70 | 150.90 | 149.60 | -7.90 |
| 1086 | 1-Norbornyl cyanide | 61.90 | 69.90 | 69.80 | -7.90 |
| 1087 | 2,4,6-Trichlorodibenzofuran | 116.10 | 108.40 | 108.20 | 7.90 |
| 1088 | 2-Pentadecanone | 139.30 | 130.70 | 131.40 | 7.90 |
| 1089 | 3-Fluorobenzamide | 104.10 | 96.10 | 96.20 | 7.90 |
| 1090 | (L)-Alanine | 138.00 | 131.70 | 130.10 | 7.90 |
| 1091 | N,N-Dimethyl-1-adamantylcarboxamide | 97.50 | 104.60 | 105.40 | -7.90 |
| 1092 | Pindolol | 146.00 | 152.10 | 153.90 | -7.90 |
| 1093 | Terbutryn | 132.40 | 140.30 |  | -7.90 |
| 1094 | 2,5-Dichloroaniline | 83.40 | 90.70 | 91.30 | -7.90 |
| 1095 | 1,4-Benzenedicarbonitrile | 88.80 | 81.70 | 80.80 | 8.00 |
| 1096 | 2,3-Dimethoxybenzoic acid | 116.60 | 109.10 | 108.60 | 8.00 |
| 1097 | 2,4,6-Trimethoxybenzonitrile | 112.60 | 104.30 | 104.60 | 8.00 |
| 1098 | Benzo[b]chrysene | 144.40 | 138.00 | 136.40 | 8.00 |
| 1099 | 1,3-Dinitro-1,3-diazacyclopentane | 105.40 | 98.20 | 97.40 | 8.00 |
| 1100 | 2,4-Dimethylbenzonitrile | 83.90 | 74.90 | 75.90 | 8.00 |
| 1101 | 6-Methoxyquinoline N-oxide | 117.90 | 110.20 | 109.90 | 8.00 |
| 1102 | 4,4'-Dimethyl-2,2'-bipyridyl | 99.70 | 91.30 | 91.70 | 8.00 |
| 1103 | 2,4,6-Trifluoroaniline | 66.00 | 73.70 | 74.00 | -8.00 |
| 1104 | 5-Ethyl-1,3-dimethyluracil | 110.00 | 102.10 | 102.00 | 8.00 |
| 1105 | 2-Ethoxycarbonyl-3-methylquinoxaline 1,4-dioxide | 133.40 | 140.80 | 141.40 | -8.00 |
| 1106 | 2-Methyl-2-butanol | 58.10 | 65.50 | 66.10 | -8.00 |
| 1107 | 4-Acetoxybenzoic acid | 116.10 | 124.30 | 124.10 | -8.00 |
| 1108 | Levofloxacin | 152.20 | 160.60 | 160.20 | -8.00 |
| 1109 | 2,3-Dihydroxyquinoxaline | 156.30 | 147.80 | 148.20 | 8.10 |
| 1110 | 3-Phenylpropiolic acid | 105.00 | 99.70 | 96.90 | 8.10 |
| 1111 | 1-Hexadecanol | 165.40 | 157.40 | 157.30 | 8.10 |
| 1112 | Dibenzo-p-dioxin | 89.60 | 97.60 | 97.70 | -8.10 |
| 1113 | Octadecane | 142.30 | 133.60 | 134.20 | 8.10 |
| 1114 | Uracil | 126.50 | 117.90 | 118.40 | 8.10 |
| 1115 | 3-Nitroacetophenone | 99.10 | 91.00 | 91.00 | 8.10 |
| 1116 | Dibenzosuberane | 103.60 | 95.50 | 95.50 | 8.10 |
| 1117 | Isoquinolin-1(2H)-one | 113.60 | 106.20 | 105.50 | 8.10 |
| 1118 | 3,5-Dichlorophenol | 82.80 | 90.70 | 90.90 | -8.10 |
| 1119 | 1,2,3,4-Tetrachlorodibenzofuran | 122.80 | 115.50 | 114.70 | 8.10 |
| 1120 | 1,4-Di-t-butylbenzene | 82.80 | 74.70 | 74.70 | 8.10 |
| 1121 | Adamantane | 58.00 | 66.10 | 66.10 | -8.10 |
| 1122 | Tropolone | 83.70 | 91.90 | 91.80 | -8.10 |
| 1123 | 4-Chloro-3-nitrophenol | 111.00 | 102.50 | 102.80 | 8.20 |
| 1124 | Tetracene | 125.00 | 116.60 | 116.80 | 8.20 |
| 1125 | 2,4-Imidazolidinedione | 116.30 | 108.60 | 108.10 | 8.20 |
| 1126 | 4,5-Dichloro-2-nitroaniline | 109.40 | 101.00 | 101.20 | 8.20 |
| 1127 | 5,7-Diiodo-8-hydroxyquinoline | 126.80 | 133.80 | 135.00 | -8.20 |
| 1128 | N,N-Diethylurea | 95.70 | 89.00 | 87.50 | 8.20 |
| 1129 | 2-Acetyl-3-methylquinoxaline N,N'-dioxide | 117.00 | 123.50 | 125.20 | -8.20 |
| 1130 | 4-Acetylphenol | 103.20 | 95.30 | 95.00 | 8.20 |
| 1131 | Malonic acid | 111.40 | 103.80 | 103.20 | 8.20 |
| 1132 | 4-Heptylbenzoic acid | 130.00 | 138.20 | 138.20 | -8.20 |
| 1133 | 1-Adamantyl isocyanide | 81.42 | 73.20 |  | 8.22 |
| 1134 | 1,1'-Diphenyl-1,1'-bicyclooctyl | 173.90 | 165.90 | 165.60 | 8.30 |
| 1135 | 1,2,4,7,8-Pentachlorodibenzo-p-dioxin | 125.30 | 133.20 | 133.60 | -8.30 |
| 1136 | Ametryn | 151.00 | 142.70 |  | 8.30 |
| 1137 | 2,3,4,5-Tetramethylbenzoic acid | 115.90 | 108.20 | 107.60 | 8.30 |
| 1138 | 2-Isopropylbenzimidazole | 109.90 | 102.80 | 101.60 | 8.30 |
| 1139 | N-Phenylglycine | 128.00 | 120.80 | 119.70 | 8.30 |
| 1140 | 2,3-Dichloroaniline | 82.20 | 90.70 | 90.50 | -8.30 |
| 1141 | Glutarimide | 94.10 | 100.90 | 102.40 | -8.30 |
| 1142 | 1,3 Diphenylisobenzofuran | 122.00 | 129.20 | 130.30 | -8.30 |
| 1143 | 2-Methylquinoxaline N,N'-dioxide | 107.00 | 114.70 | 115.30 | -8.30 |
| 1144 | 1-Methyldiadamantane | 80.63 | 72.10 | 72.30 | 8.33 |
| 1145 | Methylmalonic acid | 113.20 | 105.20 | 104.80 | 8.40 |
| 1146 | 1-(Cyanoacetyl)piperidine | 103.50 | 94.80 | 95.10 | 8.40 |
| 1147 | Azacyclononan-2-one | 102.30 | 110.40 | 110.70 | -8.40 |
| 1148 | 5-Methyl-2-thiouracil | 137.30 | 129.80 | 128.90 | 8.40 |
| 1149 | Hexamethylenetetramine | 79.60 | 80.50 | 88.00 | -8.40 |
| 1150 | Dimethyl isophthalate | 101.70 | 111.20 | 110.10 | -8.40 |
| 1151 | Perfluoropentadecane | 109.40 | 103.50 | 101.00 | 8.40 |
| 1152 | N-(2-Chloroethyl)phthalimide | 98.40 | 106.80 |  | -8.40 |
| 1153 | 4-Hexylbenzoic acid | 122.60 | 131.30 | 131.10 | -8.50 |
| 1154 | Hydrazinecarbothioamide | 125.80 | 118.70 | 117.30 | 8.50 |
| 1155 | Neopentyl alcohol | 60.30 | 68.90 | 68.80 | -8.50 |
| 1156 | Dimethyl-2,6-naphthalinedicarboxylate | 141.40 | 132.90 | 132.90 | 8.50 |
| 1157 | 2,2'-Bi-4-picoline | 99.70 | 91.30 | 91.20 | 8.50 |
| 1158 | 2,6-Xylenol | 75.60 | 84.00 | 84.10 | -8.50 |
| 1159 | 4-Nitroguaiacol | 99.40 | 107.70 | 107.90 | -8.50 |
| 1160 | 3-Methyl-1-pyrazolecarboxamide | 93.50 | 102.00 |  | -8.50 |
| 1161 | Isopropyl t-butyl ether | 42.90 | 51.20 | 51.40 | -8.50 |
| 1162 | 1,5,5-Trimethylbarbituric acid | 106.20 | 113.70 | 114.70 | -8.50 |
| 1163 | Thianaphthene | 65.70 | 74.30 | 74.20 | -8.50 |
| 1164 | 1,4-Dihydronaphthalene | 63.60 | 71.70 | 72.10 | -8.50 |
| 1165 | 3-Fluorobenzonitrile | 57.60 | 66.50 | 66.10 | -8.50 |
| 1166 | 2-Trifluoroacetylpyrrole | 72.80 | 81.10 | 81.30 | -8.50 |
| 1167 | 4-Nitrobenzylidine-t-butylamine N-oxide | 116.50 | 110.30 | 107.90 | 8.60 |
| 1168 | Cyanuric acid | 133.00 | 139.40 | 141.60 | -8.60 |
| 1169 | 2-Acetamidopyridine | 103.80 | 96.60 | 95.20 | 8.60 |
| 1170 | 7-Methoxycoumarin | 107.30 | 98.50 | 98.70 | 8.60 |
| 1171 | N,N-Diethyl-N'-pivaloylthiourea | 114.90 | 123.00 | 123.50 | -8.60 |
| 1172 | N,N'-Ethylenebis(4-aminopent-3-ene-2-one) | 131.60 | 128.30 | 123.00 | 8.60 |
| 1173 | 2,6-Dimethylbenzonitrile | 83.90 | 74.90 | 75.30 | 8.60 |
| 1174 | 4-Chloroquinoline | 78.60 | 87.00 | 87.20 | -8.60 |
| 1175 | 5,7-Dichloro-8-hydroxyquinoline | 109.30 | 117.40 | 117.90 | -8.60 |
| 1176 | 1,5-Diaminopentane | 88.50 | 95.70 | 97.10 | -8.60 |
| 1177 | 3-Butenyl 4-tolyl sulphone | 113.00 | 106.90 | 104.30 | 8.70 |
| 1178 | Hexacosane | 177.20 | 184.30 | 185.90 | -8.70 |
| 1179 | 3-Nitrobenzophenone | 123.30 | 114.90 | 114.60 | 8.70 |
| 1180 | 1-(4-Hydroxyphenyl)hexan-1-one | 132.10 | 122.90 | 123.40 | 8.70 |
| 1181 | 5,12-Dihydronaphthacene | 118.90 | 110.10 | 110.20 | 8.70 |
| 1182 | 3,4-Dihydroxytoluene | 94.90 | 103.50 | 103.60 | -8.70 |
| 1183 | cis-4-Hydroxycyclohexanecarboxylic lactone | 69.60 | 77.80 | 78.30 | -8.70 |
| 1184 | 2,2'-Biphenyldicarboxylic acid | 151.90 | 160.90 | 160.60 | -8.70 |
| 1185 | Dibenzo[e,l]pyrene | 157.20 | 148.80 | 148.40 | 8.80 |
| 1186 | 2-Fluorofluorene | 91.40 | 82.40 | 82.60 | 8.80 |
| 1187 | 3,3-Dimethyl-1-butanol | 67.50 | 75.70 | 76.30 | -8.80 |
| 1188 | Norbornane | 47.30 | 56.30 | 56.10 | -8.80 |
| 1189 | 2-Benzoyl-3-methylquinoxaline N,N'-dioxide | 153.80 | 147.50 | 145.00 | 8.80 |
| 1190 | 6-Methoxy-alpha-tetralone | 104.70 | 96.70 | 95.80 | 8.90 |
| 1191 | N,N-Dimethylurea | 93.50 | 87.00 | 84.60 | 8.90 |
| 1192 | 4-t-Butyl-2,6-xylenol | 104.10 | 95.50 | 95.20 | 8.90 |
| 1193 | Dimethyl carbonate | 49.60 | 56.80 | 58.50 | -8.90 |
| 1194 | Formamide | 71.70 | 62.80 |  | 8.90 |
| 1195 | 5-Aminovaleric acid | 144.00 | 152.80 | 152.90 | -8.90 |
| 1196 | 1,10-Decanediamine | 138.00 | 130.10 | 129.10 | 8.90 |
| 1197 | Isophthalic acid | 142.00 | 133.20 | 133.10 | 8.90 |
| 1198 | 9-Fluorenecarboxylic acid | 130.40 | 120.90 | 121.40 | 9.00 |
| 1199 | 1,2,3,4,7,8,9-Heptachlorodibenzofuran | 145.20 | 136.80 | 136.20 | 9.00 |
| 1200 | 1,2,4,6,7,8-Hexachlorodibenzofuran | 137.60 | 129.70 | 128.60 | 9.00 |
| 1201 | 2-Chloroacetophenone | 87.70 | 79.20 | 78.70 | 9.00 |
| 1202 | 4-Ethoxybenzoic acid | 121.60 | 112.60 | 112.60 | 9.00 |
| 1203 | trans-3-Methoxycinnamic acid | 124.00 | 115.70 | 115.00 | 9.00 |
| 1204 | 1-Phenyl-3-(methylamino)-2-butene-1-one | 99.20 | 108.80 | 108.20 | -9.00 |
| 1205 | 2,6-Di-t-butylphenol | 108.00 | 99.40 | 98.90 | 9.10 |
| 1206 | 3-Nitrophenol | 104.00 | 95.40 | 94.90 | 9.10 |
| 1207 | Indole | 77.60 | 85.60 | 86.70 | -9.10 |
| 1208 | 2-Ethyl-3-hydroxy-4-pyrone | 91.40 | 99.30 | 100.50 | -9.10 |
| 1209 | 1,1-Diethoxyethane | 50.60 | 59.70 |  | -9.10 |
| 1210 | N-Acetylurea | 103.10 | 112.50 | 112.20 | -9.10 |
| 1211 | Tricyclo[3,2,1,0(2,4)]oct-6-ene | 44.10 | 53.30 | 53.20 | -9.10 |
| 1212 | 1-Heptanol | 85.50 | 95.50 | 94.60 | -9.10 |
| 1213 | 3-Bromoacetophenone | 72.30 | 81.30 | 81.40 | -9.10 |
| 1214 | 4,4,4-Trifluoro-1-(2-furanyl)-butane-1,3-dione | 70.00 | 78.50 | 79.10 | -9.10 |
| 1215 | (9Z)-hexadecenoic acid | 152.10 | 162.00 | 161.20 | -9.10 |
| 1216 | 1,2,3,4,6,8,9-Heptachlorodibenzofuran | 145.60 | 136.80 | 136.40 | 9.20 |
| 1217 | 3,4-Dichlorophenol | 81.30 | 90.70 | 90.50 | -9.20 |
| 1218 | Benzoylacetonitrile | 98.50 | 89.40 | 89.30 | 9.20 |
| 1219 | 3,5-Dimethoxybenzoic acid | 127.10 | 117.80 | 117.90 | 9.20 |
| 1220 | 3-Acetylbenzonitrile | 94.70 | 86.30 | 85.50 | 9.20 |
| 1221 | 5-(1-Adamantyl)tetrazole | 126.80 | 132.30 | 136.00 | -9.20 |
| 1222 | Quinuclidine | 50.80 | 58.80 | 60.00 | -9.20 |
| 1223 | N-Theonylthiocarbamic-O-ethyl ester | 143.20 | 136.00 | 134.00 | 9.20 |
| 1224 | 8-Aminoquinoline | 93.33 | 103.10 | 102.60 | -9.27 |
| 1225 | Behenyl alcohol | 207.00 | 198.70 | 197.70 | 9.30 |
| 1226 | 1,3-Benzenedicarbonitrile | 90.10 | 81.70 | 80.80 | 9.30 |
| 1227 | 2-Carboxyadamantane | 99.80 | 109.10 | 109.10 | -9.30 |
| 1228 | 2-t-Butyl-4-methylphenol | 82.90 | 91.70 | 92.20 | -9.30 |
| 1229 | 3,5-Dibromobenzoic acid | 120.30 | 111.80 | 111.00 | 9.30 |
| 1230 | Cyclobutane-1,3-dione | 73.60 | 65.00 | 64.30 | 9.30 |
| 1231 | Methylacetamide | 70.80 | 80.10 | 80.10 | -9.30 |
| 1232 | 9-Methoxy-1,4-anthraquinone | 131.50 | 123.10 | 122.10 | 9.40 |
| 1233 | 3-Isochromanone | 97.30 | 87.90 | 87.90 | 9.40 |
| 1234 | 1-Naphthaleneacetic acid | 112.30 | 121.80 | 121.70 | -9.40 |
| 1235 | 1,8-Octanediol | 139.30 | 130.50 | 129.90 | 9.40 |
| 1236 | 4-Methoxybenzamide | 118.90 | 109.30 | 109.50 | 9.40 |
| 1237 | Corannulene | 119.50 | 127.70 | 128.90 | -9.40 |
| 1238 | 3-Picoline N-oxide | 82.20 | 90.30 | 91.60 | -9.40 |
| 1239 | N-(Diethylaminothiocarbonyl)-N'-phenylbenzamidine | 159.40 | 170.30 | 168.80 | -9.40 |
| 1240 | 1,3-Benzoxazole-2-thione | 107.50 | 99.00 | 98.00 | 9.50 |
| 1241 | 2-Bromoacetophenone | 90.90 | 81.30 | 81.40 | 9.50 |
| 1242 | 4-Chlorobiphenyl | 79.20 | 88.00 | 88.70 | -9.50 |
| 1243 | Di-t-butyl oxalate | 84.80 | 92.80 | 94.30 | -9.50 |
| 1244 | Benzo[b]triphenylene | 145.90 | 138.00 | 136.40 | 9.50 |
| 1245 | N-Theonylthiocarbamic-O-pentyl ester | 165.60 | 156.70 | 156.10 | 9.50 |
| 1246 | p-Toluamide | 109.60 | 100.70 | 100.10 | 9.50 |
| 1247 | Phenyl benzoate | 99.00 | 108.20 | 108.50 | -9.50 |
| 1248 | Perhydrotriquinacene | 56.70 | 66.10 | 66.20 | -9.50 |
| 1249 | Tetrahydrocarbazole | 97.20 | 105.90 | 106.70 | -9.50 |
| 1250 | Vat Blue 1 | 148.20 | 158.20 | 157.70 | -9.50 |
| 1251 | 1-(4-Hydroxyphenyl)pentan-1-one | 125.90 | 116.00 | 116.30 | 9.60 |
| 1252 | 3,6-Dichlorodibenzofuran | 110.90 | 101.20 | 101.30 | 9.60 |
| 1253 | 3-Cyanobenzoic acid | 116.60 | 107.40 | 107.00 | 9.60 |
| 1254 | Tetradecane | 117.60 | 108.10 | 108.00 | 9.60 |
| 1255 | 6-Hydroxynicotinic acid | 146.40 | 138.90 | 136.70 | 9.70 |
| 1256 | 2-(Biphen-3-yl)naphthalene | 137.10 | 128.10 | 127.40 | 9.70 |
| 1257 | Octachlorodibenzo-p-dioxin | 145.70 | 154.60 | 155.40 | -9.70 |
| 1258 | Picramide | 125.30 | 115.90 | 115.60 | 9.70 |
| 1259 | 2,6-Dimethyl-4-pyrone | 86.80 | 78.40 | 77.10 | 9.70 |
| 1260 | Indazolinone | 127.60 | 120.90 | 117.90 | 9.70 |
| 1261 | 4-Carboxypyridine N-oxide | 136.10 | 126.50 | 126.40 | 9.70 |
| 1262 | 5,6-Dimethyluracil | 132.40 | 122.80 | 122.70 | 9.70 |
| 1263 | 2-Naphthyl acetate | 96.30 | 106.00 | 106.00 | -9.70 |
| 1264 | 4-Heptyloxybenzoic acid | 157.20 | 147.00 | 147.50 | 9.70 |
| 1265 | 1-Chlorodibenzo-p-dioxin | 95.20 | 104.70 | 104.90 | -9.70 |
| 1266 | 2,6-Dihydroxybenzoic acid | 113.50 | 122.50 | 123.20 | -9.70 |
| 1267 | 2-Chloro-2,5-cyclohexadiene-1,4-dione | 69.00 | 77.50 | 78.70 | -9.70 |
| 1268 | Pentafluoroaniline | 63.70 | 71.90 | 73.40 | -9.70 |
| 1269 | Coumarin-3-carboxylic acid | 118.20 | 127.30 | 127.90 | -9.70 |
| 1270 | 3,5-Dimethyl-4-nitrosopyrazole | 102.90 | 93.60 | 93.10 | 9.80 |
| 1271 | trans-Stilbene | 99.60 | 89.70 | 89.80 | 9.80 |
| 1272 | 4-(Dimethylamino)-benzoic acid | 120.30 | 110.10 | 110.50 | 9.80 |
| 1273 | Hexanitroethane | 70.70 | 82.50 | 80.50 | -9.80 |
| 1274 | 2,3,5,6-Tetrachloronitrobenzene | 91.30 | 100.60 | 101.10 | -9.80 |
| 1275 | 1,3-Diaminopropane | 73.00 | 81.90 | 82.80 | -9.80 |
| 1276 | 1-Methyl-4-(2-methyl-1-propenylsulphonyl)-benzene | 102.00 | 94.70 | 92.10 | 9.90 |
| 1277 | 6-Hydroxy-alpha-tetralone | 117.50 | 107.70 | 107.60 | 9.90 |
| 1278 | n-Butylurea | 105.80 | 115.20 | 115.70 | -9.90 |
| 1279 | 1-(4-Nitrophenyl)pyrrole | 112.40 | 103.50 | 102.50 | 9.90 |
| 1280 | 5,7-Dihydro-6H-dibenzo[a,c]cyclohepten-6-one | 95.60 | 105.20 | 105.50 | -9.90 |
| 1281 | (1R) (-)-Thiocamphor | 61.70 | 71.60 |  | -9.90 |
| 1282 | 2,5-Dibromoaniline | 85.70 | 95.00 | 95.60 | -9.90 |
| 1283 | Phenoxathiin | 96.70 | 105.40 | 106.60 | -9.90 |
| 1284 | 2-Acetylpyrrole | 81.20 | 91.80 | 91.10 | -9.90 |
| 1285 | 1,1'-(1,2-Ethanediyl)bis(1-nitrosohydrazine) | 172.40 | 162.40 |  | 10.00 |
| 1286 | 1,1,1,2-Tetraphenylethane | 132.60 | 140.70 | 142.60 | -10.00 |
| 1287 | 2-Hydroxyquinoline | 119.40 | 111.30 | 109.40 | 10.00 |
| 1288 | 1-Methylimidazole | 66.90 | 75.50 | 76.90 | -10.00 |
| 1289 | N-Theonylthiocarbamic-O-propyl ester | 136.50 | 142.90 | 146.50 | -10.00 |
| 1290 | Dibenzo[g,p]chrysene | 150.90 | 159.30 | 160.90 | -10.00 |
| 1291 | Benzalaniline | 98.10 | 102.70 | 108.10 | -10.00 |
| 1292 | Niflumic acid | 130.20 | 124.40 | 120.20 | 10.00 |
| 1293 | 1-(4-Hydroxyphenyl)heptan-1-one | 139.30 | 129.70 | 129.20 | 10.10 |
| 1294 | 2-Methyl-4-nitro-1,2,3-triazole | 74.70 | 64.60 |  | 10.10 |
| 1295 | 1-Naphthylamine | 88.10 | 98.20 | 98.20 | -10.10 |
| 1296 | 9,10-Dimethylanthracene | 113.00 | 102.60 | 102.90 | 10.10 |
| 1297 | trans-Azobenzene N,N'-dioxide | 87.00 | 79.20 | 76.90 | 10.10 |
| 1298 | Tartronic acid | 116.40 | 125.70 | 126.50 | -10.10 |
| 1299 | n-Undecanoic acid | 121.30 | 131.20 | 131.40 | -10.10 |
| 1300 | t-Butylmalononitrile | 59.80 | 70.00 | 69.90 | -10.10 |
| 1301 | Benzoylformic acid | 98.50 | 108.30 | 108.60 | -10.10 |
| 1302 | Dimethylamino-2,4-pentadiene-5-al | 99.04 | 89.90 | 88.90 | 10.14 |
| 1303 | omega-Bromoacetophenone | 99.00 | 88.80 |  | 10.20 |
| 1304 | Dibenzoylperoxide | 97.90 | 94.70 | 87.70 | 10.20 |
| 1305 | 3-Methoxybenzamide | 120.20 | 109.30 | 110.00 | 10.20 |
| 1306 | Methyl docosanoate | 205.70 | 195.90 | 195.50 | 10.20 |
| 1307 | Tetracosane | 162.00 | 171.60 | 172.20 | -10.20 |
| 1308 | 3,5-Di-t-butyl-o-benzoquinone | 106.10 | 96.70 | 95.80 | 10.30 |
| 1309 | Thioacetamide | 82.80 | 73.20 | 72.50 | 10.30 |
| 1310 | 1,2-Diaminobenzene | 89.80 | 99.70 | 100.10 | -10.30 |
| 1311 | [2,2]Metacyclophane | 92.00 | 102.30 | 102.30 | -10.30 |
| 1312 | 2,7-Difluorofluorene | 91.80 | 81.40 | 81.50 | 10.30 |
| 1313 | 2-Aminobenzothiazole | 104.70 | 98.30 | 94.30 | 10.40 |
| 1314 | 1,4-Oxathiane 4,4-dioxide | 92.00 | 83.40 | 81.60 | 10.40 |
| 1315 | 3-Chloro-5-hydroxypyridine | 99.50 | 89.90 | 89.10 | 10.40 |
| 1316 | 6,6-Dimethyl-1-phenyl-4,8-dioxaspiro[2.5]octane | 97.50 | 88.30 | 87.10 | 10.40 |
| 1317 | Hexacyclopropylethane | 109.00 | 98.20 | 98.60 | 10.40 |
| 1318 | 1,3-Cyclohexanedione | 89.80 | 78.70 | 79.40 | 10.40 |
| 1319 | 2-Thiophenecarboxylic acid hydrazide | 113.30 | 118.50 | 123.70 | -10.40 |
| 1320 | 1,3,5-Trimethoxybenzene | 100.60 | 90.00 | 90.20 | 10.40 |
| 1321 | 1,3-Dithiane sulfone | 103.60 | 95.00 | 93.20 | 10.40 |
| 1322 | 1,10-Decanediol | 155.80 | 144.30 | 145.40 | 10.40 |
| 1323 | 6-Hydroxy-2,5,7,8-tetramethylchroman-2-carboxylic acid | 136.90 | 146.40 | 147.30 | -10.40 |
| 1324 | 1,4-Benzothiazin-3-one | 117.90 | 108.00 | 107.40 | 10.50 |
| 1325 | Heneicosane | 142.00 | 152.60 | 152.50 | -10.50 |
| 1326 | Methyl 3-aminobenzoate | 94.60 | 105.40 | 105.10 | -10.50 |
| 1327 | N-Phenyldiacetamide | 90.00 | 100.10 | 100.50 | -10.50 |
| 1328 | 1-Pyrenecarboxaldehyde | 117.40 | 126.20 | 127.90 | -10.50 |
| 1329 | 3,3-Tetramethyleneglutarimide | 106.80 | 117.30 | 117.30 | -10.50 |
| 1330 | N,N-Diethyl-N'-isovaleroylthiourea | 121.50 | 130.40 | 132.10 | -10.60 |
| 1331 | Pyrazine | 56.40 | 65.80 | 67.00 | -10.60 |
| 1332 | 4-Hydroxynicotinic acid | 148.10 | 140.10 | 137.50 | 10.60 |
| 1333 | 1H-Indene-1,3(2H)-dione | 97.30 | 85.80 | 86.70 | 10.60 |
| 1334 | Dibenzylsulfone | 114.00 | 123.20 | 124.60 | -10.60 |
| 1335 | 2,3-Dihydroxytoluene | 93.20 | 103.50 | 103.80 | -10.60 |
| 1336 | 4-Benzoylphenol | 130.30 | 119.30 | 119.60 | 10.70 |
| 1337 | Tolan | 95.30 | 85.90 | 84.60 | 10.70 |
| 1338 | 2-Bromoaniline | 75.00 | 85.70 | 85.70 | -10.70 |
| 1339 | Barbituric acid | 115.10 | 126.60 | 125.80 | -10.70 |
| 1340 | Bicyclopentyl | 64.10 | 75.30 | 74.90 | -10.80 |
| 1341 | 2-Hydroxyquinoxaline | 125.80 | 116.30 | 115.00 | 10.80 |
| 1342 | Adamantane 1-nitrate | 79.90 | 90.30 | 90.70 | -10.80 |
| 1343 | 1,3-Diethyl-2-thiobarbituric acid | 98.30 | 108.20 | 109.10 | -10.80 |
| 1344 | 2-Iodoaniline | 81.30 | 91.70 | 92.20 | -10.90 |
| 1345 | N-(2-Nitrophenyl)benzenesulfonamide | 126.80 | 117.60 | 115.90 | 10.90 |
| 1346 | 1,5-Bis(dimethylamino)naphthalene | 98.60 | 108.70 | 109.50 | -10.90 |
| 1347 | 4-Methyldibenzothiophene | 90.30 | 98.60 | 101.20 | -10.90 |
| 1348 | 1-Methylbenzimidazole | 86.10 | 75.80 | 75.20 | 10.90 |
| 1349 | beta-Hydroxynaphthaldehyde | 98.00 | 108.10 | 108.90 | -10.90 |
| 1350 | 1,2-Cyclohexanedione dioxime | 62.00 | 72.90 |  | -10.90 |
| 1351 | Adamantylideneadamantane | 102.20 | 113.90 | 113.10 | -10.90 |
| 1352 | Benzo[h]quinoline | 90.20 | 101.70 | 101.10 | -10.90 |
| 1353 | 2,4,6-Tri-t-butylnitrobenzene | 96.40 | 106.60 | 107.30 | -10.90 |
| 1354 | 4-Phenylaminopent-3-ene-2-one | 89.90 | 100.30 | 100.80 | -10.90 |
| 1355 | N-(4-Methoxyphenylmethylene)benzenamine N-oxide | 130.60 | 122.70 | 119.60 | 11.00 |
| 1356 | 5-Hydroxy-1-tetralone | 118.50 | 107.70 | 107.50 | 11.00 |
| 1357 | Tetramethylbutanedinitrile | 81.00 | 69.50 | 70.00 | 11.00 |
| 1358 | 2,5-Pyrrolidinedione | 83.60 | 94.00 | 94.60 | -11.00 |
| 1359 | Clioquinol | 114.80 | 125.60 | 125.80 | -11.00 |
| 1360 | N-Ethylthiourea | 118.80 | 108.50 | 107.70 | 11.10 |
| 1361 | 3,3,5,5-Tetramethyl-1-pyrazoline | 61.60 | 54.50 | 50.50 | 11.10 |
| 1362 | 2,2,3,3-Tetranitrobutane | 79.50 | 85.10 | 90.60 | -11.10 |
| 1363 | 3,9-Dimethylbenzo[a]anthracene | 113.00 | 123.90 | 124.10 | -11.10 |
| 1364 | 8-Methylquinoline | 72.80 | 83.70 | 83.90 | -11.10 |
| 1365 | 4-Benzyloxyphenol | 131.00 | 119.80 | 119.80 | 11.20 |
| 1366 | trans-2,5-Dimethoxycinnamic acid | 138.80 | 128.00 | 127.60 | 11.20 |
| 1367 | 2,3,5,6-Tetrafluoroaniline | 62.50 | 72.80 | 73.70 | -11.20 |
| 1368 | 2-Bromo-7-iodofluorene | 118.70 | 107.90 | 107.50 | 11.20 |
| 1369 | Benzal diacetate | 97.20 | 108.40 |  | -11.20 |
| 1370 | Tridecane | 91.40 | 101.80 | 102.60 | -11.20 |
| 1371 | 2-Formylpyrrole | 78.60 | 91.50 | 89.80 | -11.20 |
| 1372 | N-Benzoyl-N',N'-diisobutylurea | 137.50 | 148.70 | 148.70 | -11.20 |
| 1373 | 2,2'-Biquinoline | 134.70 | 124.60 | 123.40 | 11.30 |
| 1374 | 4-Aminobenzamide | 131.20 | 120.20 | 119.90 | 11.30 |
| 1375 | Isobutyl t-butylether | 47.80 | 58.80 | 59.10 | -11.30 |
| 1376 | 4,4'-Diethoxyazoxybenzene | 126.20 | 137.50 |  | -11.30 |
| 1377 | t-Butyl propyl ether | 46.50 | 57.40 | 57.80 | -11.30 |
| 1378 | 1,1,1-Triphenylbutane | 114.30 | 124.10 | 125.60 | -11.30 |
| 1379 | 1H,1H-Perfluorododecan-1-ol | 110.50 | 121.10 | 121.80 | -11.30 |
| 1380 | 4-Amino-N-(2,5-dichlorophenyl)benzene sulfonamide | 155.40 | 144.70 | 144.10 | 11.30 |
| 1381 | 1-(Trinitromethyl)-3-nitrateadamantane | 125.00 | 114.50 | 113.60 | 11.40 |
| 1382 | Benzofurazan | 64.40 | 75.80 |  | -11.40 |
| 1383 | 1-Naphthyl acetate | 95.10 | 106.00 | 106.50 | -11.40 |
| 1384 | PCNB | 96.30 | 107.70 | 107.70 | -11.40 |
| 1385 | 1-Octadecanol | 183.40 | 171.20 | 172.00 | 11.40 |
| 1386 | 2,3-Dihydroxybenzoic acid | 119.80 | 131.10 | 131.20 | -11.40 |
| 1387 | p-Azoxyanisole | 134.80 | 123.30 |  | 11.50 |
| 1388 | Pyrazole | 74.00 | 82.90 | 85.50 | -11.50 |
| 1389 | 2-Bromo-7-chlorofluorene | 110.40 | 99.70 | 98.90 | 11.50 |
| 1390 | 2-Nitrophenol | 75.40 | 86.80 | 86.90 | -11.50 |
| 1391 | 2-Piperidone | 78.70 | 89.80 | 90.20 | -11.50 |
| 1392 | 4-t-Butylcatechol | 99.30 | 111.20 | 110.80 | -11.50 |
| 1393 | Salicylic acid | 96.30 | 107.90 | 107.80 | -11.50 |
| 1394 | exo-Norbornene oxide | 44.10 | 55.50 | 55.60 | -11.50 |
| 1395 | 2-Methyl-8-hydroxyquinoline | 90.40 | 102.10 | 101.90 | -11.50 |
| 1396 | 3-Methyl-2-quinoxalone | 119.70 | 110.10 | 108.10 | 11.60 |
| 1397 | Acetopiperone | 104.40 | 92.90 | 92.80 | 11.60 |
| 1398 | Camphor | 56.90 | 68.50 | 68.50 | -11.60 |
| 1399 | m-Terphenyl | 118.00 | 106.70 | 106.40 | 11.60 |
| 1400 | Thianthrene | 106.40 | 113.30 | 118.00 | -11.60 |
| 1401 | 2,4,5-Trichloroaniline | 86.30 | 97.80 | 97.90 | -11.60 |
| 1402 | Cyclobutane-1,1-dicarboxylic acid | 111.20 | 121.90 | 122.80 | -11.60 |
| 1403 | Chloranil | 98.74 | 95.30 | 87.10 | 11.64 |
| 1404 | 1,4-Bis-(2-hydroxyethyl)piperazine | 130.50 | 119.00 | 118.80 | 11.70 |
| 1405 | 2,6-Dichlorophenol | 79.30 | 90.70 | 91.00 | -11.70 |
| 1406 | 1,2-Dihydroxybenzene | 88.30 | 99.80 | 100.00 | -11.70 |
| 1407 | 1,2,4-Trichlorobenzene | 62.30 | 74.50 | 74.00 | -11.70 |
| 1408 | 2,7-Dibromofluorene | 114.10 | 101.90 | 102.40 | 11.70 |
| 1409 | 6-Aminouracil | 150.40 | 156.50 | 162.10 | -11.70 |
| 1410 | 1-(4-Biphenylyl)naphthalene | 138.90 | 128.10 | 127.10 | 11.80 |
| 1411 | Pyromellitic dianhydride | 122.30 | 132.30 | 134.10 | -11.80 |
| 1412 | 2-t-Butyl-5-methylphenol | 79.70 | 91.70 | 91.50 | -11.80 |
| 1413 | m-Anthranilic acid | 128.00 | 116.40 | 116.20 | 11.80 |
| 1414 | 1,4,5,8-Tetramethylnaphthalene | 99.80 | 88.50 | 88.00 | 11.80 |
| 1415 | 5,5-Dimethylbarbituric acid | 115.80 | 127.40 | 127.60 | -11.80 |
| 1416 | Decanedioic acid | 171.80 | 160.60 | 160.00 | 11.80 |
| 1417 | 2-Methyl-1,1,1,3-tetranitropropane | 91.20 | 83.10 | 79.30 | 11.90 |
| 1418 | 5-Nitroindazole | 121.20 | 110.70 | 109.30 | 11.90 |
| 1419 | 6-Chlorouracil | 135.20 | 123.30 |  | 11.90 |
| 1420 | 1H,1H-Perfluorotetradecan-1-ol | 126.20 | 135.30 | 138.10 | -11.90 |
| 1421 | N,N-Dimethyl-4-nitroaniline | 101.30 | 89.00 | 89.40 | 11.90 |
| 1422 | Cholest-5-en-3-ol | 180.20 | 169.60 | 168.30 | 11.90 |
| 1423 | 3-Methyl-4-isopropylphenol | 104.40 | 92.20 | 92.40 | 12.00 |
| 1424 | trans-Decalin | 64.30 | 75.30 | 76.30 | -12.00 |
| 1425 | 2-Methyl-2-piperidinopropionitrile | 80.30 | 68.90 | 68.30 | 12.00 |
| 1426 | Trimethyl isocyanurate | 88.20 | 98.20 | 100.20 | -12.00 |
| 1427 | 3-(3,4-Dimethoxyphenyl)propionic acid | 143.60 | 131.50 | 131.60 | 12.00 |
| 1428 | 3-Hydroxybenzophenone | 131.70 | 119.30 | 119.60 | 12.10 |
| 1429 | Tricyclo[4.2.2.02,5]deca-3,7,9-triene | 49.40 | 59.80 | 61.50 | -12.10 |
| 1430 | 2,6-Di-t-butyl-p-cresol | 91.90 | 103.20 | 104.00 | -12.10 |
| 1431 | 2-Hydroxybenzophenone | 97.90 | 110.60 | 110.00 | -12.10 |
| 1432 | 2,3-Naphthalenediol | 109.60 | 121.50 | 121.70 | -12.10 |
| 1433 | Benzophenone anilide | 119.70 | 127.60 | 131.80 | -12.10 |
| 1434 | Pycene | 149.40 | 138.00 | 137.20 | 12.20 |
| 1435 | N,N,N',N'-Tetraphenylbiphenyl-4,4'-diamine | 198.50 | 188.10 | 186.30 | 12.20 |
| 1436 | Benzanthrone | 124.60 | 113.20 | 112.40 | 12.20 |
| 1437 | Diuron | 133.90 | 123.80 | 121.70 | 12.20 |
| 1438 | 1,2-Dihydroxynaphthalene | 109.30 | 121.50 | 121.50 | -12.20 |
| 1439 | 2-Benzylbenzimidazole | 136.20 | 125.30 | 124.00 | 12.20 |
| 1440 | 1-Chloro-4-fluorobenzene | 71.90 | 59.40 | 59.70 | 12.20 |
| 1441 | Nicotinamide N-oxide | 119.20 | 130.30 | 131.40 | -12.20 |
| 1442 | 3-Nitrophthalic anhydride | 100.40 | 111.70 | 112.60 | -12.20 |
| 1443 | 5-Methylbenzofuroxan | 92.20 | 82.70 | 79.90 | 12.30 |
| 1444 | 3-Piperidinecarboxamide | 112.50 | 103.20 | 100.20 | 12.30 |
| 1445 | 1,3,5-Trihydroxybenzene | 135.50 | 123.10 | 123.20 | 12.30 |
| 1446 | 2,6-Diphenylphenol | 119.10 | 131.80 | 131.40 | -12.30 |
| 1447 | Tetracyanoethylene | 84.30 | 94.80 | 96.60 | -12.30 |
| 1448 | 3-Hydroxypyridine N-oxide | 121.80 | 109.80 | 109.40 | 12.40 |
| 1449 | Bullvalene | 71.80 | 59.80 | 59.40 | 12.40 |
| 1450 | 5-Nitrouracil | 161.40 | 150.10 | 149.00 | 12.40 |
| 1451 | trans-3,5-Dimethoxycinnamic acid | 141.40 | 128.00 | 129.00 | 12.40 |
| 1452 | 4-Amino-N-(5-chloro-2-methylphenyl)benzenesulfonamide | 130.00 | 141.30 | 142.40 | -12.40 |
| 1453 | 5-Chloro-8-hydroxyquinoline | 98.70 | 110.30 | 111.10 | -12.40 |
| 1454 | Isonicotinic acid | 111.30 | 99.50 | 98.80 | 12.50 |
| 1455 | 1,1'-Diphenyl-1,1'-bicyclohexyl | 150.00 | 138.80 | 137.50 | 12.50 |
| 1456 | (15Z)-Tetracosenoic acid | 228.90 | 217.10 | 216.40 | 12.50 |
| 1457 | 2-Pyridinecarbothioamide | 87.30 | 98.60 | 99.80 | -12.50 |
| 1458 | Methyl pentacosanoate | 229.70 | 216.50 | 217.10 | 12.60 |
| 1459 | Octacosane | 208.90 | 197.10 | 196.30 | 12.60 |
| 1460 | 2,4,6-Trichloroaniline | 85.30 | 97.80 | 97.90 | -12.60 |
| 1461 | Quinoxaline N,N'-dioxide | 112.00 | 120.90 | 124.60 | -12.60 |
| 1462 | 1,3-Di-t-butyl urea | 91.70 | 101.30 | 104.30 | -12.60 |
| 1463 | 4'-Hydroxypropiophenone | 114.50 | 102.20 | 101.80 | 12.70 |
| 1464 | 3-Sulfolene | 62.80 | 73.50 | 75.50 | -12.70 |
| 1465 | 2,6-Dibromophenol | 83.40 | 95.10 | 96.10 | -12.70 |
| 1466 | 1,6-Bis(methylnitramino)hexane | 125.50 | 135.70 | 138.20 | -12.70 |
| 1467 | 5-Isopropylbarbituric acid | 125.00 | 136.30 | 137.70 | -12.70 |
| 1468 | Tryptophane | 186.60 | 196.00 | 199.40 | -12.80 |
| 1469 | Quinizarin | 124.10 | 135.90 | 136.90 | -12.80 |
| 1470 | 3',5'-Diisopropyl-4,4-dimethyl-3-phenyl-1,2-benzocyclobuten-3-ol | 117.90 | 130.10 | 130.70 | -12.80 |
| 1471 | 4,4'-Difluorobiphenyl | 91.20 | 79.10 | 78.30 | 12.90 |
| 1472 | 3,4-Dimethoxybenzoic acid | 129.80 | 117.80 | 116.90 | 12.90 |
| 1473 | 4-(2,4-Dichlorophenoxy)butyric acid | 124.00 | 136.80 | 136.90 | -12.90 |
| 1474 | 2,4-Dichlorophenol | 78.00 | 90.70 | 90.90 | -12.90 |
| 1475 | Methyl tetracosanoate | 222.60 | 209.60 | 209.70 | 12.90 |
| 1476 | 3-Diphenylmethyl-3-methyl-2,4-pentandione | 114.40 | 127.10 | 127.30 | -12.90 |
| 1477 | N-acetyl-isoleucine amide | 147.40 | 134.60 | 134.40 | 13.00 |
| 1478 | [18]Annulene | 120.00 | 111.00 | 107.00 | 13.00 |
| 1479 | Xylitol | 161.00 | 149.60 | 148.00 | 13.00 |
| 1480 | 1,3-Dithiole-2-thione | 75.40 | 85.00 | 88.40 | -13.00 |
| 1481 | Terephthalic acid | 146.60 | 133.20 | 133.60 | 13.00 |
| 1482 | Pyrrole-2-carboxylic acid | 100.80 | 113.00 | 113.80 | -13.00 |
| 1483 | 2-(4-Biphenylyl)naphthalene | 140.20 | 128.10 | 127.20 | 13.00 |
| 1484 | 2,6-Dimethoxybenzoic acid | 121.70 | 109.10 | 108.60 | 13.10 |
| 1485 | 1,2-Dimethylbenzimidazole | 94.00 | 80.80 | 80.90 | 13.10 |
| 1486 | Cyclohexadecane | 135.10 | 122.60 | 122.00 | 13.10 |
| 1487 | 2-Phenylaniline | 90.60 | 104.10 | 103.70 | -13.10 |
| 1488 | 3,4,5-Trimethoxyphenol | 125.30 | 113.30 | 112.20 | 13.10 |
| 1489 | 3,5-Di-t-butyl-2-hydroxybenzaldehyde | 96.00 | 109.30 | 109.10 | -13.10 |
| 1490 | 4,4'-Bipyridine | 106.30 | 93.50 | 93.20 | 13.10 |
| 1491 | trans-2-Methoxycinnamic acid | 128.80 | 115.70 | 115.70 | 13.10 |
| 1492 | Benzo-15-crown-5 | 128.10 | 140.00 | 141.20 | -13.10 |
| 1493 | 2,5-Dichlorophenol | 77.30 | 90.70 | 90.50 | -13.20 |
| 1494 | Tolfenamic acid | 128.40 | 141.20 | 141.60 | -13.20 |
| 1495 | 2-Nitrofluorene | 114.20 | 102.20 | 101.00 | 13.20 |
| 1496 | 1-Fluoro-4-nitro-1,2,3-triazole | 79.50 | 83.00 | 92.70 | -13.20 |
| 1497 | 1-Adamantanol | 75.40 | 88.80 | 88.60 | -13.20 |
| 1498 | 1-Phenyl-4,7-dioxaspiro[2.4]heptane | 91.80 | 80.60 | 78.60 | 13.20 |
| 1499 | Tetrahydrodicyclopentadiene | 52.90 | 66.10 | 66.10 | -13.20 |
| 1500 | 5,5'-Spirobi-1,3-dioxane | 73.00 | 85.00 | 86.20 | -13.20 |
| 1501 | 9,9'-Dimethyl-9,9'-bifluorenyl | 119.70 | 132.40 | 132.90 | -13.20 |
| 1502 | 1,3-Dimethylxanthine | 135.00 | 147.00 | 148.20 | -13.20 |
| 1503 | 2,3-Butyleneglycol | 77.40 | 90.50 | 90.70 | -13.30 |
| 1504 | 6-Methyluracil | 131.00 | 118.00 | 117.70 | 13.30 |
| 1505 | 1-((5-(4-Ethoxyphenyl)amino)-1,2,4-thiadiazol-3-yl)-2-propanol | 152.80 | 142.10 | 139.50 | 13.30 |
| 1506 | 1-Phenyltetrazole | 104.00 | 90.60 |  | 13.40 |
| 1507 | 1,1,1-Trifluoro-2,2,2-triphenylethane | 112.30 | 101.80 | 98.90 | 13.40 |
| 1508 | Methylbenzene | 43.10 | 56.20 | 56.50 | -13.40 |
| 1509 | 2,5-Dimethyl-2H-tetrazole | 48.70 | 62.10 |  | -13.40 |
| 1510 | 1,12-Dodecanediamine | 156.30 | 143.80 | 142.90 | 13.40 |
| 1511 | Tricyclo[8.2.2.24,7]hexadecane | 91.60 | 104.20 | 105.10 | -13.50 |
| 1512 | Ciprofloxacin | 159.90 | 146.30 |  | 13.60 |
| 1513 | Methyl adamantyl-1-carboxylate | 83.10 | 96.10 | 96.70 | -13.60 |
| 1514 | Methyl heptadecanoate | 148.90 | 161.50 | 162.50 | -13.60 |
| 1515 | 2,2'-Biphenol | 114.40 | 127.40 | 128.00 | -13.60 |
| 1516 | N,N'-Diethylurea | 95.40 | 109.50 | 109.00 | -13.60 |
| 1517 | [3,3]Paracyclophane | 103.30 | 115.90 | 116.90 | -13.60 |
| 1518 | Dibenzylsulfide | 94.00 | 104.80 | 107.60 | -13.60 |
| 1519 | Norfloxacin | 132.60 | 146.20 |  | -13.60 |
| 1520 | trans-2,3-Dimethoxycinnamic acid | 141.00 | 128.00 | 127.30 | 13.70 |
| 1521 | Dithiooxamide | 105.40 | 113.50 | 119.10 | -13.70 |
| 1522 | 1,3,5,5-Tetramethylbarbituric acid | 87.70 | 100.00 | 101.40 | -13.70 |
| 1523 | cis-Decalin | 62.50 | 75.30 | 76.30 | -13.80 |
| 1524 | 1,2,4-Triphenylbenzene | 147.80 | 133.80 | 134.00 | 13.80 |
| 1525 | 1,2-Ethanediol dinitrate | 103.80 | 92.30 | 90.00 | 13.80 |
| 1526 | Isonicotinamide | 117.00 | 103.30 | 103.10 | 13.90 |
| 1527 | Piracetam | 123.80 | 110.60 | 109.90 | 13.90 |
| 1528 | 2-Ethylimidazole | 89.60 | 101.10 | 103.50 | -13.90 |
| 1529 | 2,4-Dibromophenol | 81.30 | 95.10 | 95.20 | -13.90 |
| 1530 | Diamantan-3-ol | 116.10 | 102.40 | 102.10 | 14.00 |
| 1531 | 3,3'-Dinitrobenzophenone | 147.40 | 133.80 | 133.40 | 14.00 |
| 1532 | 6-Aza-2-thiothymine | 120.10 | 132.20 | 134.10 | -14.00 |
| 1533 | 2,3-Dichlorophenol | 76.90 | 90.70 | 90.90 | -14.00 |
| 1534 | alpha-Methyl-5-((4-methoxyphenyl)amino)-1,2,4-thiadiazole-3-ethanol | 129.00 | 135.70 | 143.00 | -14.00 |
| 1535 | Bicyclo[2.2.2]octane | 48.10 | 62.60 | 62.10 | -14.00 |
| 1536 | 1,3,5-Trimethyladamantane | 77.80 | 62.90 | 63.80 | 14.00 |
| 1537 | 2,4-Dimethoxybenzoic acid | 123.40 | 109.10 | 109.30 | 14.10 |
| 1538 | Cetane | 135.10 | 120.80 | 121.00 | 14.10 |
| 1539 | 2-Phenylcarbamoyl-3-methylquinoxaline N-oxide | 145.10 | 158.10 | 159.20 | -14.10 |
| 1540 | Enantholactam | 89.60 | 103.60 | 103.80 | -14.20 |
| 1541 | 4,4,4-Trifluoro-1-(2-furanyl)-1-hydroxybutane-3-one | 70.00 | 83.30 | 84.40 | -14.40 |
| 1542 | Phenazine N-oxide | 100.00 | 113.00 | 114.40 | -14.40 |
| 1543 | Nitrosulfathiazole | 144.00 | 136.10 | 129.50 | 14.50 |
| 1544 | 3-Methyl-N-(2-methylphenyl)-2-quinoxalinecarboxamide N,N'-dioxide | 154.80 | 166.10 | 169.30 | -14.50 |
| 1545 | Meso-2,3-diethyl-2,3-dimethylsuccinonitrile | 97.65 | 83.20 | 83.10 | 14.55 |
| 1546 | 3-Hydroxy-6-methylpyridine | 96.20 | 81.70 | 81.60 | 14.60 |
| 1547 | 2,7-Diiodofluorene | 126.20 | 113.90 | 111.60 | 14.60 |
| 1548 | trans-3-Phenyl-2-propen-1-ol | 109.00 | 95.30 | 94.40 | 14.60 |
| 1549 | 4-Aminoquinaldine | 115.30 | 102.00 | 100.70 | 14.60 |
| 1550 | 1,4-Dimethyl-2,5-piperazinedione | 103.60 | 90.40 | 89.00 | 14.60 |
| 1551 | Tetraphenylethylene | 133.40 | 145.90 | 148.00 | -14.60 |
| 1552 | 2-Chloroaniline | 68.80 | 83.50 | 83.40 | -14.60 |
| 1553 | 2-Hydroxy-4-methylquinoline | 128.10 | 115.10 | 113.40 | 14.70 |
| 1554 | 2,4,6-Triaminopyrimidine | 125.40 | 120.10 | 110.60 | 14.80 |
| 1555 | 2,6-Dibromoaniline | 80.70 | 95.00 | 95.50 | -14.80 |
| 1556 | 8-Nitroquinaldine | 111.00 | 97.70 | 96.20 | 14.80 |
| 1557 | Butylcyclohexane | 64.20 | 79.00 | 79.00 | -14.80 |
| 1558 | Cyclotetradecane | 95.60 | 109.90 | 110.40 | -14.80 |
| 1559 | 1,2,3,4-Tetrachlorodibenzo-p-dioxin | 111.30 | 126.10 | 126.20 | -14.90 |
| 1560 | 3-(5-(4-Methoxyphenyl)-2-furyl)acrylic acid | 160.00 | 146.00 | 145.10 | 14.90 |
| 1561 | 2-Carboxypyridine N-oxide | 94.40 | 107.90 | 109.30 | -14.90 |
| 1562 | DABCO | 61.90 | 50.80 | 46.90 | 15.00 |
| 1563 | [2,2]Metaparacyclophane | 87.50 | 102.30 | 102.50 | -15.00 |
| 1564 | Methyl(1,1,2-trimethylpropyl)malononitrile | 62.00 | 77.70 | 77.00 | -15.00 |
| 1565 | 9-Ethyl-9H-carbazole | 97.10 | 81.70 | 82.10 | 15.00 |
| 1566 | 2-Nitrobenzoic acid | 118.70 | 103.50 | 103.60 | 15.10 |
| 1567 | 1,3,5-Triphenylbenzene | 147.80 | 133.80 | 132.70 | 15.10 |
| 1568 | 2,4,6-Trichlorophenol | 82.30 | 97.80 | 97.50 | -15.20 |
| 1569 | cis-1,2-Cyclohexanediol | 91.30 | 106.10 | 106.60 | -15.30 |
| 1570 | 1-Nitro-2,6-diisopropylbenzene | 80.60 | 96.20 | 96.00 | -15.40 |
| 1571 | 2-Thiouracil | 138.50 | 125.00 | 123.10 | 15.40 |
| 1572 | 5-Hydroxymethylene-5H-6,7-dihydrodibenzo[a.c]cyclohepten-6-one | 133.00 | 118.70 | 117.60 | 15.40 |
| 1573 | Methyl-(1-thia-3-azaspiro{5.5]undec-2-en-2-yl)amine | 95.10 | 107.10 | 110.50 | -15.40 |
| 1574 | Diethylcarbonate | 59.20 | 71.00 | 74.60 | -15.40 |
| 1575 | N-(1-Aza-3-thiabicyclo[3.3.1]non-2-ylidene)-4-(trifluoromethyl)aniline | 89.00 | 105.40 | 104.40 | -15.40 |
| 1576 | 4-Amino-N-(2-bromo-4-nitrophenyl)benzenesulfonamide | 142.80 | 158.70 | 158.20 | -15.40 |
| 1577 | p-Acetotoluide | 115.10 | 99.30 | 99.60 | 15.50 |
| 1578 | l,1'-Diadamantyl ketone | 109.00 | 123.90 | 124.50 | -15.50 |
| 1579 | N-Methylcarbazole | 95.50 | 80.70 | 80.00 | 15.50 |
| 1580 | N-(Diethylaminothiocarbony)-N-ethylbenzamide | 141.20 | 126.40 | 125.60 | 15.60 |
| 1581 | 4,4'-Dihydroxybiphenyl | 143.00 | 127.40 | 127.40 | 15.60 |
| 1582 | 3,3'-Dimethylbiphenyl | 71.90 | 87.00 | 87.50 | -15.60 |
| 1583 | Tetrakis(p-tolyl)porphyrin | 260.40 | 275.00 | 276.00 | -15.60 |
| 1584 | 1,1,1-Trinitroethane | 72.00 | 57.80 | 56.20 | 15.80 |
| 1585 | 1,8-Bis(dimethylamino)naphthaline | 94.70 | 108.70 | 110.50 | -15.80 |
| 1586 | 1-Pentanol | 65.90 | 81.80 | 81.70 | -15.80 |
| 1587 | 1,1'-Diphenyl-1,1'-bicyclopentyl | 141.00 | 125.20 | 125.10 | 15.90 |
| 1588 | 1-Methyl-2-phenylindole | 111.10 | 95.80 | 95.20 | 15.90 |
| 1589 | 2-t-Butoxycarbonyl-3-methylquinoxaline N-oxide | 140.80 | 125.40 | 124.80 | 16.00 |
| 1590 | 1-Propanol | 52.80 | 68.00 | 68.80 | -16.00 |
| 1591 | 2,7-Di-t-butylfluorene | 119.90 | 104.10 | 103.90 | 16.00 |
| 1592 | Propylenecarbonate | 69.00 | 59.60 | 53.00 | 16.00 |
| 1593 | 2,6-Dichloroaniline | 74.20 | 90.70 | 90.20 | -16.00 |
| 1594 | 2-Iodophenol | 76.10 | 91.80 | 92.10 | -16.00 |
| 1595 | 1,2,3,4,5,6,7,8-Octahydroanthracene | 82.30 | 97.90 | 98.30 | -16.00 |
| 1596 | 2,2',6,6'-Tetramethylazobenzene N,N'-dioxide | 107.20 | 94.20 | 91.10 | 16.10 |
| 1597 | 1,1'-Biadamantane | 113.80 | 98.20 | 97.70 | 16.10 |
| 1598 | 3,3-Tetramethyleneglutaric acid | 126.90 | 142.60 | 143.00 | -16.10 |
| 1599 | 1-Heptadecanol | 149.50 | 164.30 | 165.60 | -16.10 |
| 1600 | Nitropenta | 152.00 | 159.30 | 168.10 | -16.10 |
| 1601 | Ribitol | 161.00 | 175.50 | 177.10 | -16.10 |
| 1602 | 2,2'-Difluorobiphenyl | 95.00 | 79.10 | 78.80 | 16.20 |
| 1603 | trans-1,2-Cyclohexanediol | 90.40 | 106.10 | 106.60 | -16.20 |
| 1604 | Arachic alcohol | 169.50 | 185.00 | 185.80 | -16.30 |
| 1605 | Erythritol | 130.20 | 146.80 | 146.60 | -16.40 |
| 1606 | 1-Fluoro-3-nitro-1,2,4-triazole | 79.70 | 66.00 | 63.30 | 16.40 |
| 1607 | Vitamin B3 | 119.40 | 103.30 | 103.00 | 16.40 |
| 1608 | Propylcyclohexane | 55.70 | 72.70 | 72.20 | -16.50 |
| 1609 | 2-Methyl-8-quinolinol | 90.40 | 107.00 | 106.90 | -16.50 |
| 1610 | N-Acetyl-D-leucine amide | 120.40 | 134.60 | 137.00 | -16.60 |
| 1611 | Norbornene | 38.70 | 55.70 | 55.30 | -16.60 |
| 1612 | Thiobarbituric acid | 118.30 | 133.60 | 134.90 | -16.60 |
| 1613 | Isatin | 118.80 | 102.50 | 102.20 | 16.60 |
| 1614 | Benzo-12-crown-4 | 104.30 | 120.00 | 121.00 | -16.70 |
| 1615 | Arabitol | 161.00 | 175.50 | 177.80 | -16.80 |
| 1616 | 1,4-Dithiane sulfone | 99.90 | 87.20 | 83.00 | 16.90 |
| 1617 | Tetradecanoic acid | 168.60 | 151.80 | 151.70 | 16.90 |
| 1618 | 1-[4-(1-Thia-3-azaspiro[5.5]undec-2-en-2-ylamino)phenyl]ethanone | 156.00 | 141.40 | 139.10 | 16.90 |
| 1619 | 2-Methoxycarbonyl-3-methylquinoxaline N,N'-dioxide | 118.30 | 133.70 | 135.20 | -16.90 |
| 1620 | Indole-3-carboxyaldehyde | 120.30 | 105.10 | 103.30 | 17.00 |
| 1621 | 3,3,4,4-Tetramethyl-1,2-diazetine | 62.30 | 47.70 | 45.30 | 17.00 |
| 1622 | 2,6-Di-t-butyl-4-phenylphenol | 143.20 | 127.10 | 126.20 | 17.00 |
| 1623 | 9,9'-Bianthracene | 148.00 | 165.00 | 165.00 | -17.00 |
| 1624 | 2,6-Dichlorobenzoquinone | 69.90 | 83.50 | 86.90 | -17.00 |
| 1625 | Tetrahydro-2H-1,3-oxazine-2-thione | 108.90 | 92.70 | 91.80 | 17.10 |
| 1626 | (4-Methylphenyl)-(1-aza-3-thiabicyclo[3.3.1]non-2-ylidene)amine | 101.10 | 116.10 | 118.20 | -17.10 |
| 1627 | Ethylcyclohexane | 49.00 | 66.30 | 66.10 | -17.10 |
| 1628 | 4,5-Benzo-1,3-dithiole-2-thione | 118.80 | 108.60 | 101.60 | 17.20 |
| 1629 | 1-Methyl-1-nitrosohydrazine | 79.50 | 96.70 |  | -17.20 |
| 1630 | 2,5-Di-t-butylphenol | 116.70 | 99.40 | 99.40 | 17.30 |
| 1631 | Methylthiouracil | 140.70 | 125.00 | 123.40 | 17.30 |
| 1632 | 9,10-Dimethylphenanthrene | 119.50 | 102.60 | 102.10 | 17.40 |
| 1633 | Trifluoroacetamide | 77.70 | 62.40 | 60.30 | 17.40 |
| 1634 | 2,5-Dihydro-3-methylthiophene 1,1-dioxide | 64.00 | 78.30 | 81.40 | -17.40 |
| 1635 | 9-Hydroxy-1,4-anthraquinone | 109.50 | 125.50 | 126.90 | -17.40 |
| 1636 | 4-Pyridinecarbothioamide | 118.80 | 103.50 | 101.30 | 17.50 |
| 1637 | 3,5-Difluoroaniline | 91.70 | 74.60 | 74.20 | 17.50 |
| 1638 | 2,4,6-Triisopropylbenzophenone | 116.00 | 132.00 | 133.50 | -17.50 |
| 1639 | 2-Adamantanone | 63.00 | 79.70 | 80.50 | -17.50 |
| 1640 | trans-4-Methoxycinnamic acid | 134.00 | 115.70 | 116.50 | 17.50 |
| 1641 | 1-Piperidinoacetonitrile | 56.02 | 73.00 | 73.60 | -17.58 |
| 1642 | 3-Methoxy-2-nitrobenzoic acid | 141.90 | 124.40 | 124.30 | 17.60 |
| 1643 | 3-Furanacrylic acid | 106.80 | 92.20 | 89.20 | 17.60 |
| 1644 | 1-Methylisatin | 105.60 | 88.70 | 88.00 | 17.60 |
| 1645 | 3,5-Di-t-butylcatechol | 104.70 | 122.70 | 122.30 | -17.60 |
| 1646 | 3-(Diphenylmethyl)-3-propyl-2,4-pentanedione | 124.70 | 140.80 | 142.30 | -17.60 |
| 1647 | Glycine anhydride | 134.00 | 117.30 | 116.30 | 17.70 |
| 1648 | 4-Hydroxycoumarin | 128.50 | 111.40 | 110.70 | 17.80 |
| 1649 | Pentacyclo[5.4.02,603,1005,9]undecane-8,11-dione | 92.60 | 76.40 | 74.80 | 17.80 |
| 1650 | 6-Azathymine | 109.10 | 125.10 | 126.90 | -17.80 |
| 1651 | Bicyclo[3.3.1]nonane | 51.00 | 69.00 | 68.90 | -17.90 |
| 1652 | Tri-t-butylmethanol | 63.20 | 81.90 | 81.10 | -17.90 |
| 1653 | Methyl 2-pyrrolecarboxylate | 85.40 | 102.00 | 103.30 | -17.90 |
| 1654 | Pentaphenylbenzene | 170.20 | 188.00 | 188.10 | -17.90 |
| 1655 | 5-Nitroimidazole | 122.90 | 112.70 | 104.90 | 18.00 |
| 1656 | 2,5-Dimethyl-3-hexyne-2,5-diol | 106.70 | 90.80 | 88.70 | 18.00 |
| 1657 | Salicylaldoxime | 105.20 | 87.10 |  | 18.10 |
| 1658 | Bicyclo[3.3.2]decane | 58.20 | 75.30 | 76.30 | -18.10 |
| 1659 | Bicyclo[3.3.3]undecane | 63.60 | 81.70 | 81.70 | -18.10 |
| 1660 | Saccharin | 112.60 | 95.70 | 94.30 | 18.30 |
| 1661 | (3-Methylphenyl)-(1-aza-3-thiabicyclo[3.3.1]non-2-ylidene)amine | 131.00 | 116.10 | 112.60 | 18.40 |
| 1662 | 1-((5-p-Tolylamino)-1,2,4-thiadiazol-3-yl)-2-propanol | 142.00 | 126.40 | 123.60 | 18.40 |
| 1663 | Dicyanomethane | 78.20 | 60.90 | 59.80 | 18.40 |
| 1664 | N-(4-Methoxyphenyl)sulfanilamide | 124.00 | 142.80 | 142.40 | -18.40 |
| 1665 | Enrofloxacin | 138.90 | 156.00 | 157.30 | -18.40 |
| 1666 | Tetraphenylporphyrin | 244.20 | 260.00 | 262.60 | -18.40 |
| 1667 | 2-Hydroxypyridine N-oxide | 89.40 | 100.00 | 107.90 | -18.50 |
| 1668 | Benzo[f]quinoline | 83.10 | 101.70 | 101.70 | -18.60 |
| 1669 | 6-Amino-1-methyluracil | 158.20 | 142.70 | 139.60 | 18.60 |
| 1670 | 2-Phenylcarbamoyl-3-methylquinoxaline N,N'-dioxide | 145.10 | 162.40 | 163.70 | -18.60 |
| 1671 | 1-Ethyl-2-phenylindole | 115.00 | 96.80 | 96.30 | 18.70 |
| 1672 | cis-1,3,5,7-Tetraoxadecalin | 94.90 | 78.60 | 76.20 | 18.70 |
| 1673 | N-Theonylthiocarbamic-O-hexyl ester | 180.10 | 163.60 | 161.30 | 18.80 |
| 1674 | Dulcitol | 205.00 | 187.00 | 186.10 | 18.90 |
| 1675 | Methyl heptacosanoate | 247.80 | 230.30 | 228.90 | 18.90 |
| 1676 | N-Cyclohexyl-2,4,6-trimethylbenzaldehyde imine | 104.90 | 113.60 | 123.80 | -18.90 |
| 1677 | N-Acetylglycinamide | 140.20 | 121.20 | 121.20 | 19.00 |
| 1678 | 2,3,5,6-Tetrachloroaniline | 86.00 | 104.90 | 105.00 | -19.00 |
| 1679 | Caffeine | 119.00 | 133.20 | 138.00 | -19.00 |
| 1680 | 1,2-Dihydroxy-3-isopropyl-6-methylbenzene | 96.60 | 115.50 | 115.60 | -19.00 |
| 1681 | 3-Azabicyclo[3.2.2]nonane | 57.80 | 73.20 | 76.80 | -19.00 |
| 1682 | 2,4,6-Trinitroresorcinol | 120.80 | 139.30 | 139.90 | -19.10 |
| 1683 | Benzo[b]fluorene | 122.00 | 103.30 | 102.80 | 19.20 |
| 1684 | 3-Methyl-4-hydroxybenzaldehyde | 89.30 | 107.30 | 108.50 | -19.20 |
| 1685 | all-trans-1,5,9-Cyclododecatriene | 74.68 | 91.70 | 93.90 | -19.22 |
| 1686 | cis-3,6-Dimethylpiperazine-2,5-dione | 130.40 | 113.90 | 111.10 | 19.30 |
| 1687 | Camphene | 47.00 | 64.80 | 66.30 | -19.30 |
| 1688 | 2-Methyl-2-propanol | 41.00 | 58.70 | 60.30 | -19.30 |
| 1689 | 1-Pentadecanol | 132.10 | 150.60 | 151.40 | -19.30 |
| 1690 | exo-4-Hydroxy-endo-endo-tetracyclo[6.2.1.1.3,6.02,7]dodecane | 79.00 | 97.90 | 98.30 | -19.30 |
| 1691 | (4-Chlorophenyl)-(1-aza-3-thiabicyclo[3.3.1]non-2-ylidene)amine | 136.70 | 119.40 | 117.30 | 19.40 |
| 1692 | 1,1,1,4-Tetranitrobutane | 102.50 | 88.60 | 83.10 | 19.40 |
| 1693 | Benzimidazole-2-thione | 130.90 | 113.30 | 111.50 | 19.40 |
| 1694 | 2,4,6-Tri-t-butylaniline | 92.50 | 110.90 | 111.90 | -19.40 |
| 1695 | Tyrosine | 202.40 | 185.80 | 182.90 | 19.50 |
| 1696 | 1H-Benzotriazole | 98.20 | 87.70 | 78.70 | 19.50 |
| 1697 | Bicyclo[2.2.2]oct-2-ene | 43.40 | 62.50 | 62.90 | -19.50 |
| 1698 | Bicyclo[4.2.1]non-3-ene | 49.70 | 69.30 | 69.30 | -19.60 |
| 1699 | N,N,2,2-Tetramethylpropionamide | 55.10 | 74.40 | 74.70 | -19.60 |
| 1700 | Dimedone | 99.80 | 79.60 | 80.00 | 19.80 |
| 1701 | 9-Methylpoxanthine | 84.00 | 102.80 | 103.80 | -19.80 |
| 1702 | 2,3-Dimethylquinoxaline 1,4-dioxide | 124.40 | 108.40 | 104.50 | 19.90 |
| 1703 | 2,4,6-Trinitrophenol | 105.10 | 124.60 | 125.00 | -19.90 |
| 1704 | Diphenylmethylene diacetate | 123.60 | 138.00 | 143.60 | -20.00 |
| 1705 | N-Nitrosodiisobutylamine | 66.50 | 86.50 |  | -20.00 |
| 1706 | 3-Methylpicric acid | 111.20 | 128.40 | 131.20 | -20.00 |
| 1707 | 4,4-Dimethyl-1,3-cyclohexanedione | 99.20 | 79.60 | 79.10 | 20.10 |
| 1708 | Perhydroanthracene | 72.70 | 91.50 | 92.80 | -20.10 |
| 1709 | Pentacene | 158.00 | 138.00 | 137.80 | 20.20 |
| 1710 | 3-(5-Phenyl-2-furyl)acrylic acid | 152.60 | 133.70 | 132.40 | 20.20 |
| 1711 | 1,6-Anhydro-beta-D-glucopyranose | 125.70 | 105.40 |  | 20.30 |
| 1712 | N-Acetylacetamide | 73.20 | 92.20 | 93.50 | -20.30 |
| 1713 | N-(2-Hydroxyphenylmethylene)benzenamine N-oxide | 116.50 | 133.70 | 136.80 | -20.30 |
| 1714 | (4-Fluorophenyl)-(1-aza-3-thiabicyclo[3.3.1]non-2-ylidene)amine | 92.60 | 111.40 | 113.00 | -20.40 |
| 1715 | 3-Oxabicyclo[3.2.2]nonane | 53.30 | 73.10 | 73.70 | -20.40 |
| 1716 | 4-Amino-N-(4-nitrophenyl)benzenesulfonamide | 131.40 | 149.40 | 151.80 | -20.40 |
| 1717 | 3-Hydroxycoumarin | 91.90 | 111.40 | 112.30 | -20.40 |
| 1718 | N-Benzoylthiocarbamic O-hexyl ester | 139.70 | 160.30 | 160.10 | -20.40 |
| 1719 | Anthrarufin | 117.60 | 135.90 | 138.10 | -20.50 |
| 1720 | 1,3,5,7-Tetramethyladamantane | 81.10 | 61.80 | 60.60 | 20.50 |
| 1721 | Quinoxaline | 66.60 | 84.90 | 87.20 | -20.60 |
| 1722 | Diamantan-4-ol | 117.80 | 97.90 | 97.10 | 20.70 |
| 1723 | 1-Aminoadamantane | 61.70 | 80.00 | 82.40 | -20.70 |
| 1724 | Pentachlorophenol | 91.60 | 112.10 | 112.30 | -20.70 |
| 1725 | 5,12-Naphthacenedione | 109.00 | 128.30 | 129.70 | -20.70 |
| 1726 | 4-Hydroxypyridine | 103.80 | 82.80 | 83.00 | 20.80 |
| 1727 | 1,3-Oxazolidine-2-thione | 104.30 | 85.80 | 83.50 | 20.80 |
| 1728 | trans-2,4-Dimethoxycinnamic acid | 149.20 | 128.00 | 128.30 | 20.90 |
| 1729 | 1,3-Dioxolan-2-one | 73.80 | 59.00 | 52.90 | 20.90 |
| 1730 | 4-Methyl-2-pyrazoline-5-one | 111.80 | 93.00 | 90.90 | 20.90 |
| 1731 | Cubane | 55.20 | 35.00 | 34.30 | 20.90 |
| 1732 | Cyclododecane | 76.40 | 97.20 | 97.30 | -20.90 |
| 1733 | Dimethyl malonate | 111.70 | 90.40 | 90.70 | 21.00 |
| 1734 | 7-Norbornanone | 47.20 | 68.20 | 68.30 | -21.10 |
| 1735 | 2-Methyl-4-pyridone | 113.00 | 91.80 | 91.80 | 21.20 |
| 1736 | 2-Benzimidazolone | 126.40 | 106.30 | 105.20 | 21.20 |
| 1737 | Diamantan-1-ol | 117.90 | 97.90 | 96.70 | 21.20 |
| 1738 | Bicyclo[3.2.2]non-6-ene | 48.00 | 69.30 | 69.20 | -21.20 |
| 1739 | cis-8-Methyl-2-hydrindanone | 60.90 | 81.40 | 82.20 | -21.30 |
| 1740 | 1,3,5-Tris(diphenylamino)benzene | 200.80 | 214.00 | 222.10 | -21.30 |
| 1741 | Methyl hexacosanoate | 243.50 | 223.40 | 222.10 | 21.40 |
| 1742 | 1,2,3,6,7,8-Hexahydropyrene | 84.80 | 105.60 | 106.20 | -21.40 |
| 1743 | Bicyclo[3.3.1]non-2-ene | 48.20 | 69.30 | 69.60 | -21.40 |
| 1744 | Tetra(3-pyridyl)porphyrin | 270.40 | 285.30 | 291.90 | -21.50 |
| 1745 | 4-Hydroxybenzylalcohol | 129.30 | 108.40 | 107.70 | 21.60 |
| 1746 | 7-Heptadecanol | 137.00 | 158.10 | 158.60 | -21.60 |
| 1747 | Trimethyl 1,3,5-benzenetricarboxylate | 118.90 | 140.20 | 140.60 | -21.70 |
| 1748 | trans-Heptacyclene | 149.00 | 126.60 | 127.20 | 21.80 |
| 1749 | Diethoxydiphenylmethane | 97.10 | 113.40 | 118.90 | -21.80 |
| 1750 | Ethyl 2-pyrrolecarboxylate | 88.00 | 109.10 | 109.80 | -21.80 |
| 1751 | 3,4-Dihydro-2H-1-benzopyran | 56.70 | 78.10 | 78.70 | -22.00 |
| 1752 | 2,6-Dichlorohydroquinone | 92.05 | 114.00 | 114.10 | -22.05 |
| 1753 | 7,7,8,8-Tetracyanoquinodimethane | 105.00 | 123.00 | 127.20 | -22.20 |
| 1754 | p-Quaterphenyl | 155.00 | 133.80 | 132.70 | 22.30 |
| 1755 | Carbostyril | 119.40 | 97.60 | 97.10 | 22.30 |
| 1756 | N-Benzoylthiocarbamic O-ethylester | 112.20 | 132.80 | 134.60 | -22.40 |
| 1757 | trans-3,4-Dimethoxycinnamic acid | 149.90 | 128.00 | 127.30 | 22.60 |
| 1758 | 3,4,5,6-Tetramethylphenanthrene | 133.00 | 109.90 | 110.40 | 22.60 |
| 1759 | 2,5-Dibromobenzoic acid | 133.60 | 111.80 | 110.80 | 22.80 |
| 1760 | Cycloheptanol | 67.40 | 91.10 | 90.20 | -22.80 |
| 1761 | Tetracyanomethane | 61.10 | 82.70 | 83.90 | -22.80 |
| 1762 | trans-Cyclohexyl-1,4-diamine | 105.00 | 87.20 | 82.10 | 22.90 |
| 1763 | 2,3-5,6-Dibenzoxalene | 129.40 | 105.90 | 106.50 | 22.90 |
| 1764 | 5-Amino-6-nitroquinoline | 136.40 | 113.40 | 113.40 | 23.00 |
| 1765 | 1,8-Diaminonaphthalene | 99.00 | 121.40 | 122.10 | -23.10 |
| 1766 | 4-Piperidinecarboxamide | 123.60 | 103.20 | 100.20 | 23.40 |
| 1767 | Di-t-butyl-isopropylmethanol | 59.30 | 82.40 | 82.70 | -23.40 |
| 1768 | 3-Phenylcyclobutenone | 58.60 | 81.90 | 82.00 | -23.40 |
| 1769 | Hexacosanoic acid | 257.80 | 234.40 | 234.40 | 23.40 |
| 1770 | 2,4,6-Tri-t-butylphenol | 87.50 | 110.90 | 111.10 | -23.60 |
| 1771 | 1,4-Bis(diphenylamino)benzene | 178.80 | 160.40 | 155.10 | 23.70 |
| 1772 | 3,4,4'-Trichlorocarbanilide | 182.20 | 159.80 | 158.50 | 23.70 |
| 1773 | (4-Ethylphenyl)-(1-aza-3-thiabicyclo[3.3.1]non-2-ylidene)amine | 101.90 | 122.90 | 125.70 | -23.80 |
| 1774 | 6-Amino-1,3-dimethyluracil | 148.70 | 129.00 | 124.70 | 24.00 |
| 1775 | 5,8-Dihydroxy-1,4-naphthalenedione | 96.70 | 118.40 | 120.70 | -24.00 |
| 1776 | 3,4-Diethyl-3,4-bis(4-t-butylphenyl)-hexane | 167.70 | 145.70 | 143.50 | 24.20 |
| 1777 | 8-Oxabicyclo[5.1.0]octane | 47.40 | 71.60 | 71.70 | -24.30 |
| 1778 | Heptacyclo[6.6.02,6.03,13.04,11.05,9.08,1.O10,14]tetradecane | 79.30 | 54.70 | 55.00 | 24.30 |
| 1779 | Eicosane | 169.80 | 146.20 | 145.50 | 24.30 |
| 1780 | 4-Nitrobenzofuroxan | 97.30 | 110.00 | 121.70 | -24.40 |
| 1781 | Dimethyl 2,6-cuneanedicarboxylate | 113.10 | 88.70 | 88.70 | 24.40 |
| 1782 | Tetraisobutylsucccinonitrile | 152.70 | 130.10 | 128.20 | 24.50 |
| 1783 | 2-Naphthylamine | 73.90 | 98.20 | 98.40 | -24.50 |
| 1784 | 4-Amino-N-(4-cyanophenyl)benzenesulfonamide | 168.30 | 144.70 | 143.70 | 24.60 |
| 1785 | 1,4-Anthraquinone | 134.50 | 110.90 | 109.90 | 24.60 |
| 1786 | 7-Hydroxycoumarin | 133.60 | 109.50 | 109.00 | 24.60 |
| 1787 | Methyl octacosanoate | 261.30 | 237.10 | 236.60 | 24.70 |
| 1788 | 3-(4-Dimethylaminophenyl)acrylic acid | 144.50 | 120.30 | 119.60 | 24.90 |
| 1789 | Barbital | 117.30 | 141.20 | 142.30 | -25.00 |
| 1790 | N-(Diethylaminothiocarbonyl)benzamidine | 126.00 | 147.70 | 151.10 | -25.10 |
| 1791 | 4-Amino-N-(3,4-dichlorophenyl)benzene sulfonamide | 167.50 | 144.70 | 142.30 | 25.20 |
| 1792 | 1,12-Dodecanediol | 181.70 | 158.10 | 156.50 | 25.20 |
| 1793 | 9-Oxatetracyclo[5.4.0.0(3,10).0(4,8)]undeca-5-en-2-one | 96.00 | 71.60 | 70.60 | 25.40 |
| 1794 | Tetrakis(o-aminophenyl)porphyrin | 331.88 | 353.00 | 357.40 | -25.52 |
| 1795 | 2-Cyano-2-nitroadamantane | 70.00 | 95.10 | 95.70 | -25.70 |
| 1796 | alpha,alpha'-Dihydroxy-4-diisopropylbenzene | 136.80 | 112.00 | 110.90 | 25.90 |
| 1797 | N-(Diethylaminothiocarbonyl)-N',N'-diethylbenzamidine | 122.20 | 143.40 | 148.10 | -25.90 |
| 1798 | [1,8]Paracyclophane | 110.90 | 136.20 | 136.80 | -25.90 |
| 1799 | Nitrosobenzene | 80.80 | 58.00 | 54.80 | 26.00 |
| 1800 | Isoniazid | 101.00 | 121.60 | 127.00 | -26.00 |
| 1801 | Tetramethylpyromellitate | 143.30 | 169.20 | 169.60 | -26.30 |
| 1802 | 4-Methylcarbostyril | 128.10 | 102.40 | 101.70 | 26.40 |
| 1803 | 5-Chlorouracil | 148.30 | 123.90 | 121.60 | 26.70 |
| 1804 | 2-Methyl-5-pyrazinecarboxylic acid | 100.90 | 120.70 | 127.60 | -26.70 |
| 1805 | 1,3-Diamino-2,4,6-trinitrobenzene | 143.50 | 121.90 | 116.50 | 27.00 |
| 1806 | Nicotinic acid N-oxide | 152.30 | 126.50 | 125.10 | 27.20 |
| 1807 | 1,3-Diphenylbutane | 73.60 | 101.30 | 101.00 | -27.40 |
| 1808 | Flurbiprofen | 155.60 | 128.20 | 128.20 | 27.40 |
| 1809 | Acridone | 136.20 | 109.60 | 108.70 | 27.50 |
| 1810 | 1-Nitro-2-nitrosobenzene | 54.00 | 76.90 | 81.60 | -27.60 |
| 1811 | 2-Butynedinitrile | 44.25 | 60.50 | 72.00 | -27.75 |
| 1812 | Azacyclotridecan-2-one | 110.60 | 138.00 | 138.40 | -27.80 |
| 1813 | 4-Pyridone | 118.60 | 91.80 | 90.70 | 27.90 |
| 1814 | Dibenzo-24-crown-8-ether | 241.70 | 218.90 | 213.60 | 28.10 |
| 1815 | 1,8-Cyclotetradecadiyne | 94.30 | 101.40 | 122.90 | -28.60 |
| 1816 | 2,2'-Dinitroazobenzene N,N'-dioxide | 95.00 | 117.10 | 123.70 | -28.70 |
| 1817 | Dimethyl cubane-1,4-dicarboxylate | 117.20 | 88.70 | 88.50 | 28.70 |
| 1818 | N,N'-Bis(salicylaldehydo)tetramethylenediimine | 165.10 | 173.30 | 193.90 | -28.80 |
| 1819 | Palmitic acid | 194.00 | 165.60 | 165.10 | 28.90 |
| 1820 | Dulcose | 205.00 | 178.40 | 176.00 | 29.00 |
| 1821 | (3-Chloro-4-methylphenyl)-(1-aza-3-thiabicyclo[3.3.1]non-2-ylidene)amine | 150.50 | 123.20 | 121.40 | 29.10 |
| 1822 | Carbonothioic dihydrazide | 152.10 | 137.00 | 122.90 | 29.20 |
| 1823 | Benzoic anhydride | 96.20 | 124.90 | 125.50 | -29.30 |
| 1824 | Pyridine-2,5-dicarboxylic acid | 163.60 | 134.70 | 134.20 | 29.40 |
| 1825 | 5-((3-Chloro-4-methylphenyl)amino)-alpha-methyl-1,2,4-thiadiazole-3-ethanol | 120.70 | 142.90 | 150.10 | -29.40 |
| 1826 | Cyclododecanone | 83.20 | 111.80 | 112.60 | -29.40 |
| 1827 | 4,5-Dipropyl-4,5-bis-(4-t-butylphenyl)octane | 198.00 | 170.90 | 168.50 | 29.50 |
| 1828 | N-Benzoylthiocarbamic O-butyl ester | 120.70 | 146.60 | 150.50 | -29.80 |
| 1829 | Tetrakis(p-nitrophenyl)porphyrin | 312.44 | 335.80 | 342.50 | -30.06 |
| 1830 | 1,2,3,4-Tetraphenylnaphthalene | 154.20 | 182.20 | 184.40 | -30.20 |
| 1831 | 3-Methylquinoxaline-2-carboxamide 1,4-dioxide | 166.60 | 139.80 | 136.30 | 30.30 |
| 1832 | N-Methyl-2,3-5,6-dibenzazalene | 132.00 | 102.60 | 101.70 | 30.30 |
| 1833 | 2-t-Butoxycarbonyl-3-methylquinoxaline N,N'-dioxide | 164.10 | 138.30 | 133.70 | 30.40 |
| 1834 | trans-Azobenzene | 94.10 | 110.00 | 124.50 | -30.40 |
| 1835 | Dicyclohexylsulfide | 84.70 | 102.30 | 115.10 | -30.40 |
| 1836 | Quinoxaline-2,3-dione | 156.30 | 126.00 | 125.70 | 30.60 |
| 1837 | Cytosine | 155.00 | 126.50 | 124.20 | 30.80 |
| 1838 | Melamine | 120.00 | 127.50 | 151.30 | -31.30 |
| 1839 | 4-Hydroxy-2-methylpyridine | 113.00 | 81.70 | 81.60 | 31.40 |
| 1840 | 3-Methyldiadamantane | 103.10 | 72.10 | 70.90 | 32.20 |
| 1841 | 4-Quinolinol | 135.10 | 103.20 | 102.70 | 32.40 |
| 1842 | Squaric acid | 84.00 | 111.80 | 116.70 | -32.70 |
| 1843 | N,N'-Ethylenebis(benzoylacetonimine) | 198.80 | 176.20 | 166.00 | 32.80 |
| 1844 | 9-Fluorenyl-tris(methoxycarbonyl)methane | 136.00 | 168.10 | 169.10 | -33.10 |
| 1845 | Dimethyl phthalate | 77.70 | 111.20 | 110.90 | -33.20 |
| 1846 | Tetracosanoic acid | 253.00 | 220.60 | 219.30 | 33.70 |
| 1847 | 4,5,9,10-Tetrahydropyrene | 65.00 | 97.80 | 98.90 | -33.90 |
| 1848 | 6,13-Pentacenedione | 116.30 | 150.10 | 150.20 | -33.90 |
| 1849 | Atenolol | 140.00 | 170.40 | 174.10 | -34.10 |
| 1850 | Dimethylamino-2,4,6-heptatrienal | 131.00 | 100.10 | 96.80 | 34.20 |
| 1851 | Sulfamethopyrazine | 130.40 | 158.20 | 164.60 | -34.20 |
| 1852 | 4-Diethylaminonitrosobenzene | 107.90 | 76.90 | 72.00 | 35.90 |
| 1853 | 2-Nitroadamantane | 58.00 | 81.20 | 94.10 | -36.10 |
| 1854 | Phenylcyclobutenedione | 54.40 | 90.20 | 90.60 | -36.20 |
| 1855 | Tetrahydro-2,6-diphenyl-4H-thiopyran-4-one | 144.00 | 122.20 | 107.40 | 36.60 |
| 1856 | 2-Methyl-4-quinolinol | 139.00 | 102.10 | 102.10 | 36.90 |
| 1857 | 1,1,1-Tris(methoxycarbonyl)-2,2-diphenylethane | 136.00 | 172.60 | 172.90 | -36.90 |
| 1858 | Pentamethoxycarbonylbenzene | 165.10 | 198.20 | 202.20 | -37.10 |
| 1859 | 4,4'-Biphenyldicarboxylic acid | 196.40 | 160.90 | 159.10 | 37.30 |
| 1860 | Tetramethylpyrazine | 94.60 | 61.40 | 57.00 | 37.60 |
| 1861 | N,N’-Bis(salicylaldehydo)ethylenediimine | 141.30 | 159.60 | 180.10 | -38.80 |
| 1862 | 5,6,11,12-Tetraphenyltetracene | 262.00 | 224.90 | 222.90 | 39.10 |
| 1863 | 1-Methylcytosine | 149.10 | 112.80 | 110.00 | 39.10 |
| 1864 | 4,5,6-Trimethyl-2,3-benzoxalene | 139.50 | 103.60 | 97.50 | 42.00 |
| 1865 | Hexaphenylbenzene | 175.50 | 215.10 | 218.30 | -42.80 |
| 1866 | 2-Nitroso-1-naphthol | 56.50 | 94.40 | 100.00 | -43.50 |
