# Supplementary material for: Calculation of Five Thermodynamic Molecular Descriptors by Means of a General Computer Algorithm Based on the Group-Additivity Method: Standard Enthalpies of Vaporization, Sublimation and Solvation, and Entropy of Fusion of Ordinary Organic Molecules and Total Phase-Change Entropy of Liquid Crystals
Source: Molecules. 2017 Jun 25;22(7):1059. doi: 10.3390/molecules22071059 (PMC6152037; doi:10.3390/molecules22071059)
Supplement: Supplementary file 1 [file molecules-22-01059-s001.zip › S7. Experimental and Calculated Heat-of-Fusion Data Table.doc]

|  | **Molecules sorted by deviation** |  |  |
| --- | --- | --- | --- |
| **Nr** | **Molecule name** | **ΔH°(fus) exp** | **ΔH°(fus) calc** |
|  |  |  |  |
| 1 | 4-Nitroguaiacol | 21.69 | 22.40 |
| 2 | 2-Oxoglutaric acid | 28.59 | 27.20 |
| 3 | p-Cresol | 12.60 | 16.00 |
| 4 | 4-Hydroxybenzaldehyde | 21.60 | 22.40 |
| 5 | 1-Naphthalenecarboxylic acid | 14.10 | 17.00 |
| 6 | Ibuprofen | 23.10 | 23.50 |
| 7 | 2-Naphthoic acid | 13.80 | 17.00 |
| 8 | 4-Methylbenzoic acid | 15.40 | 17.90 |
| 9 | 2-Amino-3-methylbenzoic acid | 27.30 | 26.20 |
| 10 | 1,4-Dinitrobenzene | 17.58 | 19.40 |
| 11 | 4-Methoxybenzamide | 21.30 | 22.00 |
| 12 | 2,8-Dichlorodibenzofuran | 27.20 | 26.40 |
| 13 | Acenaphthylene | 3.90 | 9.80 |
| 14 | 2-Methoxy-5-nitrophenol | 21.43 | 22.40 |
| 15 | 4-Phenyltoluene | 11.20 | 14.90 |
| 16 | 2-Thiophenecarboxylic acid | 21.00 | 22.10 |
| 17 | Succinonitrile | 9.84 | 14.30 |
| 18 | Saprorthoquinone | 23.10 | 23.60 |
| 19 | Ethyl 4,7-dihydro-4,7-ethano-2H-isoindole-1-carboxylate | 45.40 | 39.20 |
| 20 | 2,4-Dichlorophenol | 19.00 | 20.30 |
| 21 | 1,12-Dodecanediamine | 67.10 | 53.90 |
| 22 | Ambroxol | 31.50 | 29.00 |
| 23 | 2-Amino-6-methylbenzoic acid | 27.50 | 26.20 |
| 24 | p-Toluidine | 21.00 | 22.20 |
| 25 | 3-Acetylthiophene | 19.40 | 21.10 |
| 26 | 4-Fluoroacetophenone | 15.10 | 17.50 |
| 27 | 2-Methoxybenzamide | 21.60 | 22.00 |
| 28 | 2,6-Dichloro-4-nitroaniline | 29.48 | 28.20 |
| 29 | 2,6-Diphenyl-1,4-dithiin | 24.60 | 24.80 |
| 30 | N-(2,3-Dichlorophenyl)benzenesulfonamide | 27.20 | 25.90 |
| 31 | 4-Bromoiodobenzene | 16.90 | 18.70 |
| 32 | 2-Amino-5-methylbenzoic acid | 27.63 | 26.20 |
| 33 | 1,14-Tetradecanedioic acid | 56.50 | 47.10 |
| 34 | 4-Fluoronitrobenzene | 14.90 | 17.30 |
| 35 | Thymoquinone | 18.20 | 19.60 |
| 36 | 3-Chlorophenylacetic acid | 22.00 | 23.00 |
| 37 | 1,3-Dimethyl-2,4-dinitro-5-t-butylbenzene | 16.70 | 19.30 |
| 38 | 4-Bromotoluene | 15.10 | 18.20 |
| 39 | Hexamethylbenzene | 12.80 | 16.60 |
| 40 | Perfluorotetradecane | 24.60 | 24.90 |
| 41 | Isoproturon | 21.30 | 22.60 |
| 42 | p-Anthranilic acid | 24.50 | 23.90 |
| 43 | Hydroxyacetic acid | 18.00 | 20.30 |
| 44 | Dimethylacetamide | 10.20 | 13.90 |
| 45 | 1-Acetyladamantane | 18.50 | 19.70 |
| 46 | Ethyl methyl carbonate | 11.24 | 14.60 |
| 47 | Chrysanthemic acid | 14.50 | 17.90 |
| 48 | Phenazine | 26.20 | 26.10 |
| 49 | Dodecanedioic acid | 49.80 | 42.60 |
| 50 | 3-Methoxybenzamide | 21.90 | 22.00 |
| 51 | 4-Bromoacetophenone | 17.00 | 19.70 |
| 52 | 2-t-Butyl-5-methylphenol | 12.50 | 15.40 |
| 53 | Naproxen | 23.40 | 24.20 |
| 54 | Benzo[e]pyrene | 4.80 | 11.20 |
| 55 | N-Methylbenzamide | 16.10 | 17.90 |
| 56 | N-(4-Nitrophenyl)benzenesulfonamide | 20.80 | 22.40 |
| 57 | N,N'-Bis(3-methoxyphenyl)urea | 36.76 | 32.30 |
| 58 | 2,4-Difluoronitrobenzene | 14.30 | 16.60 |
| 59 | 2,3-Dichlorophenol | 19.60 | 20.30 |
| 60 | Nonanedioic acid | 40.20 | 36.00 |
| 61 | 1,4-Dichlorobenzene | 19.30 | 21.40 |
| 62 | Isobutyramide | 19.20 | 20.00 |
| 63 | Tricyclo[4.2.2.02,5]deca-3,7,9-triene | 1.50 | 7.60 |
| 64 | 1-Octadecanol | 66.60 | 54.50 |
| 65 | 1,2-Dihydronaphthalene | 10.50 | 15.30 |
| 66 | cis-1,2-Cyclohexanediol | 20.27 | 20.70 |
| 67 | 2,6-Dichlorophenol | 19.70 | 20.30 |
| 68 | 2-Methylbenzimidazole | 21.08 | 22.70 |
| 69 | Adiponitrile | 18.00 | 19.10 |
| 70 | 4-Chlorophenylacetic acid | 23.60 | 23.00 |
| 71 | 2,4,6-Trichlorodibenzofuran | 31.90 | 28.80 |
| 72 | N-(4-Bromophenyl)-4H-3,1-benzothiazin-2-amine | 24.50 | 23.60 |
| 73 | 2-Bromonaphthalene | 15.50 | 17.30 |
| 74 | 4-Iodotoluene | 15.50 | 17.30 |
| 75 | 1-Thiaindan | 14.80 | 16.80 |
| 76 | 1,2-Ethanediol dinitrate | 33.30 | 29.70 |
| 77 | Methyl 4-methylbenzoate | 23.50 | 24.50 |
| 78 | Phenol | 10.90 | 15.70 |
| 79 | 2,3-Dichlorodibenzofuran | 26.20 | 26.40 |
| 80 | 2,6-Dioxaadamantane | 9.60 | 14.80 |
| 81 | 1-Nonadecanol | 72.40 | 58.70 |
| 82 | trans-3-Methoxycinnamic acid | 22.60 | 23.90 |
| 83 | Dimethylene urethane | 17.30 | 20.20 |
| 84 | N-Methylnicotinamide | 19.20 | 19.80 |
| 85 | 4-Phenylpyridine | 13.00 | 17.20 |
| 86 | Salicylsalicylic acid | 29.00 | 28.40 |
| 87 | Octadecane | 51.70 | 42.50 |
| 88 | 4-Methoxybenzoic acid | 21.80 | 23.40 |
| 89 | 2-Hydroxy-4-methoxybenzophenone | 21.80 | 23.40 |
| 90 | 1,3,5-Tribromobenzene | 21.72 | 21.50 |
| 91 | 1,12-Dodecanediol | 51.20 | 42.10 |
| 92 | Perylene | 7.00 | 11.20 |
| 93 | 2-Methylbenzoic acid | 16.60 | 17.90 |
| 94 | Phenylacetic acid | 19.90 | 20.20 |
| 95 | Dodecane | 38.10 | 32.90 |
| 96 | 4-Bromophenol | 15.50 | 17.10 |
| 97 | Dibenzofuran | 19.40 | 21.80 |
| 98 | 2,2-diphenyl-1,3-dioxolane | 15.10 | 16.80 |
| 99 | N-(4-Methyl-2-nitrophenyl)acetamide | 20.97 | 20.90 |
| 100 | 1,10-Decanediol | 42.10 | 37.70 |
| 101 | Tetradecanedinitrile | 41.94 | 37.60 |
| 102 | N-(4-Methylphenyl)-4H-3,1-benzothiazin-2-amine | 19.90 | 22.20 |
| 103 | 3,4-Dichloronitrobenzene | 20.60 | 22.70 |
| 104 | Hexabromobenzene | 24.60 | 25.50 |
| 105 | Phenacetin | 25.20 | 23.80 |
| 106 | Pyrimidine | 14.20 | 16.10 |
| 107 | 1,3-Diiodobenzene | 16.50 | 17.70 |
| 108 | Suberonitrile | 21.97 | 23.70 |
| 109 | 2-Imidazolidinone | 15.10 | 16.70 |
| 110 | 2-Aminophenol | 21.70 | 21.30 |
| 111 | 5-Hydroxymethyl-5,6-dihydro-7H-dibenzo[a,c]cycloheptane | 16.00 | 17.30 |
| 112 | Pentanedinitrile | 12.03 | 16.80 |
| 113 | 2,4,6-Tribromophenol | 20.90 | 20.70 |
| 114 | Musk xylene | 20.80 | 20.60 |
| 115 | 1,3,5,7-Tetrachloronaphthalene | 27.10 | 25.00 |
| 116 | 1,2,4,5-Tetrabromobenzene | 24.40 | 23.10 |
| 117 | 1,2,3,5,7-Pentachloronaphthalene | 27.10 | 27.40 |
| 118 | Perfluorotridecane | 23.70 | 22.60 |
| 119 | Quinaldine | 12.50 | 17.20 |
| 120 | trans-1,2-Cyclohexanediol | 21.00 | 20.70 |
| 121 | 1,8-Bis(dimethylamino)naphthaline | 18.00 | 18.60 |
| 122 | 2-Chlorophenylacetic acid | 24.30 | 23.00 |
| 123 | Benzo[ghi]perylene | 4.00 | 8.80 |
| 124 | 2,9-Dimethyl-1,10-phenanthroline | 17.60 | 18.30 |
| 125 | Butanedioic acid | 23.60 | 25.00 |
| 126 | 3-Thiophenecarboxylic acid | 18.30 | 21.30 |
| 127 | 2-Chloronitrobenzene | 20.50 | 20.30 |
| 128 | Perfluoropentadecane | 26.70 | 27.20 |
| 129 | Ethylcarbamate | 26.70 | 27.20 |
| 130 | Benzo[a]pyrene | 3.80 | 11.20 |
| 131 | Fluorene | 7.80 | 11.40 |
| 132 | 4-Chloro-2-nitrophenol | 22.69 | 21.80 |
| 133 | 3-Aminophenol | 22.00 | 21.30 |
| 134 | Biphenyl | 13.00 | 15.00 |
| 135 | Vioxx | 12.00 | 14.30 |
| 136 | Lophine | 35.90 | 31.00 |
| 137 | Sebaconitrile | 28.20 | 28.30 |
| 138 | 2,5-Dichlorophenol | 20.60 | 20.30 |
| 139 | 6-Methyl-1,2,3,4-tetrahydro-1-oxocarbazole | 26.90 | 24.70 |
| 140 | 4-(Phenylmethoxy)benzeneacetic acid | 29.30 | 29.10 |
| 141 | 2-Pentadecanone | 53.90 | 46.30 |
| 142 | 3-Chlorophenol | 13.40 | 18.00 |
| 143 | 1,2,3,4-Tetrachloronaphthalene | 23.40 | 25.00 |
| 144 | 2-(4-Nitrophenyl)-1-(4-(4-butylcyclohexyl)phenyl)ethanone | 36.40 | 34.10 |
| 145 | Propylenecarbonate | 9.00 | 12.10 |
| 146 | 1-Phenyldodecane | 45.50 | 40.50 |
| 147 | 1,4-Dihydronaphthalene | 9.40 | 15.30 |
| 148 | 2-Chloronaphthalene | 13.40 | 18.10 |
| 149 | 1,10-Phenanthroline | 19.40 | 22.30 |
| 150 | o-Toluamide | 15.40 | 16.50 |
| 151 | 4-Chlorophenol | 13.20 | 18.00 |
| 152 | Methyl 4-hydroxybenzoate | 25.30 | 23.40 |
| 153 | 3,4,5-Trimethoxyphenol | 31.90 | 31.10 |
| 154 | 2,2,4-Trimethyl-1,3-pentanediol | 24.20 | 22.60 |
| 155 | Benzoic acid | 17.50 | 17.90 |
| 156 | Benzamide | 15.50 | 16.50 |
| 157 | 4-Methoxy-2-nitrophenol | 22.42 | 24.50 |
| 158 | 1,4,7,10-Tetrathiacyclododecane | 31.00 | 30.50 |
| 159 | 1,4-Dinitropiperazine | 20.80 | 20.20 |
| 160 | 4-Iodoaniline | 19.98 | 22.80 |
| 161 | o-Cresol | 14.80 | 16.00 |
| 162 | 1,4-Dibromobenzene | 20.39 | 19.90 |
| 163 | 1,3-Dinitrobenzene | 19.68 | 19.40 |
| 164 | Nortricyclene | 1.20 | 9.70 |
| 165 | 4-Amino-3-methylbenzoic acid | 21.80 | 24.10 |
| 166 | 1,4,7-Trithiacyclononane | 29.00 | 25.90 |
| 167 | epsilon-Caprolactam | 16.20 | 20.20 |
| 168 | 18-Hexylpentatriacontane | 107.00 | 80.40 |
| 169 | 1,4-Dimethylnaphthalene | 10.60 | 16.30 |
| 170 | 2,4,6-Tribromoaniline | 25.75 | 26.90 |
| 171 | Dibenzo[b,d]thiophene | 19.20 | 19.00 |
| 172 | 2,3:6,7-Dibenzocycloocta-2,6-dien-1-one | 17.20 | 17.60 |
| 173 | 2-Nitrophenol | 17.00 | 20.80 |
| 174 | Dodecanedinitrile | 34.30 | 32.90 |
| 175 | N-(4-Chlorophenyl)benzenesulfonamide | 25.80 | 23.60 |
| 176 | Benzo[b]fluoranthene | 2.40 | 7.20 |
| 177 | 4-(4-Propylcyclohexyl)benzonitrile | 21.50 | 24.00 |
| 178 | Phenanthrene | 13.00 | 14.60 |
| 179 | 1,2,4-Tribromobenzene | 17.90 | 21.50 |
| 180 | 1-Ethyladamantane | 11.30 | 13.40 |
| 181 | 4-Chloronitrobenzene | 16.17 | 20.30 |
| 182 | Khellin | 32.30 | 31.60 |
| 183 | Decanedioic acid | 47.00 | 38.30 |
| 184 | 2-Nitrodiphenylamine | 26.14 | 23.70 |
| 185 | Tetradecanoic acid | 57.90 | 45.90 |
| 186 | 1,10-Decanediamine | 57.80 | 49.50 |
| 187 | 2-Ethylanthracene | 16.20 | 16.70 |
| 188 | 2,3,5,6-Tetramethyl-1,4-benzoquinone | 18.40 | 22.00 |
| 189 | 2,3-Butyleneglycol | 10.80 | 16.70 |
| 190 | 2-(2-Benzofuryl)-2-imidazoline | 26.00 | 23.50 |
| 191 | Ethyl 8-(hydroxymethyl)-6-phenyl-2-oxo-2H-1-benzopyran-3-carboxylate | 43.10 | 39.30 |
| 192 | 2,4,8-Trichlorodibenzofuran | 33.60 | 28.80 |
| 193 | Benzene | 10.60 | 16.60 |
| 194 | 3-Amino-4-methylbenzoic acid | 26.90 | 24.10 |
| 195 | 2-Indanone | 16.90 | 17.10 |
| 196 | 2:3,6:7-Dibenzobicyclo[3.2.2]nona-2,6-dien-4-one | 10.90 | 12.90 |
| 197 | 2-Iodoaniline | 19.38 | 22.80 |
| 198 | 2-(4-Nitrophenyl)-1-(4-pentylphenyl)ethanone | 29.50 | 29.90 |
| 199 | Pyrazine | 15.90 | 20.40 |
| 200 | Chloroacetic acid | 16.30 | 20.70 |
| 201 | 1-(4-Nitrophenylethynyl)-4-pentylbenzene | 21.50 | 20.20 |
| 202 | N-Benzyl-pivalophenone imine | 28.10 | 24.80 |
| 203 | 4-Fluorophenol | 14.10 | 15.00 |
| 204 | Dicyanomethane | 11.10 | 12.90 |
| 205 | 2,5-Norbornadiene | 10.10 | 12.20 |
| 206 | 2,6-Dimethylquinoline | 17.40 | 17.30 |
| 207 | 3-t-Butylphenol | 14.70 | 15.40 |
| 208 | 1,2,4,5,6,8-Hexachloronaphthalene | 28.90 | 29.60 |
| 209 | 2-Fluorobenzonitrile | 4.00 | 12.20 |
| 210 | 3-Fluorobenzonitrile | 4.00 | 12.20 |
| 211 | N-(4-Chlorophenyl)-4H-3,1-benzothiazin-2-amine | 27.50 | 24.30 |
| 212 | 3-Fluoronitrobenzene | 17.50 | 17.30 |
| 213 | 2-(2,4-Dichlorophenoxy)propanoic acid | 32.00 | 31.80 |
| 214 | 2,3-Dinitrophenol | 22.67 | 20.90 |
| 215 | 4-Acetylphenol | 18.10 | 17.70 |
| 216 | 1-Bromo-4-chlorobenzene | 15.80 | 20.50 |
| 217 | Butanamide | 19.20 | 22.90 |
| 218 | Nitrofungin | 20.88 | 19.60 |
| 219 | Decane | 28.90 | 29.70 |
| 220 | 1,8-Octanediol | 33.90 | 33.20 |
| 221 | Dibenz[ah]anthracene | 10.90 | 12.60 |
| 222 | 9,10-Dihydrophenanthrene | 12.20 | 13.50 |
| 223 | Methyl 2-thiopheneacetate | 36.10 | 30.20 |
| 224 | Xanthene | 20.70 | 24.00 |
| 225 | p-Terphenyl | 11.80 | 13.20 |
| 226 | 2,7-Dichlorofluorene | 16.40 | 16.40 |
| 227 | 9-Methylanthracene | 13.70 | 14.50 |
| 228 | 3-Nitrobenzamide | 18.30 | 17.70 |
| 229 | 4-Dimethylaminopyridine | 21.60 | 20.00 |
| 230 | trans-4-Methoxycinnamic acid | 27.20 | 23.90 |
| 231 | 2,6-Dinitrophenol | 22.91 | 20.90 |
| 232 | 4-Nitrophenol | 11.00 | 17.30 |
| 233 | Fenchone | 15.50 | 15.70 |
| 234 | Dodecanamide | 46.00 | 41.80 |
| 235 | 1,6-Hexanedioic acid | 28.40 | 29.50 |
| 236 | 4-Chloroaniline | 21.10 | 24.40 |
| 237 | 1-Hexadecanol | 57.70 | 50.00 |
| 238 | Methyl 4-bromobenzoate | 23.20 | 25.90 |
| 239 | 1-Indanone | 17.60 | 17.10 |
| 240 | 4-Chloroacetophenone | 15.70 | 20.70 |
| 241 | 1,2,3-Trichloronaphthalene | 18.40 | 22.60 |
| 242 | 2-Chlorofluorene | 13.40 | 14.10 |
| 243 | 2-Hydroxydiphenylmethane | 17.00 | 16.60 |
| 244 | 1,3-Dioxolan-2-one | 13.00 | 13.80 |
| 245 | 3-Chloronitrobenzene | 22.30 | 20.30 |
| 246 | 1-Methylimidazole | 14.90 | 20.20 |
| 247 | Tritane | 14.60 | 14.90 |
| 248 | 4-Aminophenol | 23.80 | 21.30 |
| 249 | Quinazoline | 18.00 | 17.20 |
| 250 | 2,4'-Bipyridine | 17.00 | 16.50 |
| 251 | Pentabromophenol | 27.60 | 23.90 |
| 252 | Corannulene | 4.00 | 7.40 |
| 253 | 1-Adamantanol | 14.60 | 14.80 |
| 254 | Thioxanthene | 23.90 | 21.30 |
| 255 | 4-Ethoxybenzoic acid | 26.40 | 28.40 |
| 256 | Ethyl 4-hydroxybenzoate | 26.40 | 28.40 |
| 257 | 3,5-Dichloroanisole | 23.68 | 26.50 |
| 258 | 1-(4-Nitrophenylethynyl)-4-butoxybenzene | 21.80 | 25.20 |
| 259 | 2,6-Dibromoaniline | 21.79 | 25.20 |
| 260 | 2-(4-Nitrophenyl)-1-(4-(4-propylcyclohexyl)phenyl)ethanone | 38.90 | 31.70 |
| 261 | 2-Tetradecanone | 48.80 | 44.10 |
| 262 | Adamantane | 6.30 | 14.40 |
| 263 | n-Octanoic acid | 32.30 | 32.60 |
| 264 | (2-Furyl)oxoacetamide | 17.80 | 16.90 |
| 265 | 2-Chlorodibenzofuran | 20.10 | 24.10 |
| 266 | 3-Chlorodibenzofuran | 20.10 | 24.10 |
| 267 | Carbazole | 10.40 | 11.70 |
| 268 | 2-Hydroxymethyl-1,4-benzodioxan | 28.80 | 30.20 |
| 269 | Hexanamide | 24.60 | 27.30 |
| 270 | 4-Formylbenzoic acid | 21.30 | 25.00 |
| 271 | 1,4,8,11-Tetrathiacyclotetradecane | 35.70 | 35.10 |
| 272 | Malonic acid | 23.10 | 26.30 |
| 273 | 3-Chlorobenzoic acid | 23.70 | 20.90 |
| 274 | Coronene | 3.10 | 6.50 |
| 275 | 18-Butylpentatriacontane | 104.00 | 77.00 |
| 276 | Musk ketone | 23.80 | 20.90 |
| 277 | 2-(4-Nitrophenyl)-1-(4-propoxyphenyl)ethanone | 32.00 | 32.60 |
| 278 | 1,2,3,4-Tetrachlorodibenzofuran | 38.40 | 31.10 |
| 279 | 3-Nitrophenol | 18.10 | 16.90 |
| 280 | 2,4-Dibromoaniline | 21.37 | 25.20 |
| 281 | N-Palmitoyl-pyrazinamide | 51.80 | 46.50 |
| 282 | Caprylamide | 30.48 | 31.60 |
| 283 | Indole | 12.30 | 18.90 |
| 284 | 1,2,3,7-Tetrachlorodibenzofuran | 38.50 | 31.10 |
| 285 | Lifibrol | 38.10 | 30.80 |
| 286 | 1,2,3,4,6,7,8-Heptachlorodibenzo-p-dioxin | 57.30 | 50.40 |
| 287 | 1,6-Dimethylnaphthalene | 8.50 | 16.30 |
| 288 | 3-Thiopheneacetic acid | 18.80 | 23.50 |
| 289 | 1,2,4,5-Tetrachlorobenzene | 22.50 | 26.10 |
| 290 | 1,3,6,8-Tetrachlorodibenzofuran | 38.60 | 31.10 |
| 291 | 1,3-Benzodioxole-5-carboxylic acid | 30.50 | 31.70 |
| 292 | 2-Fluoronitrobenzene | 18.90 | 17.30 |
| 293 | 4-Iodophenol | 17.20 | 16.10 |
| 294 | 2-Hydroxybenzylalcohol | 21.50 | 19.10 |
| 295 | Valeramide | 21.00 | 25.10 |
| 296 | Oxalamide | 11.70 | 18.60 |
| 297 | N-(2-Cyanoethyl)aniline | 20.38 | 18.30 |
| 298 | 1,3,7,9-Tetrachlorodibenzofuran | 38.70 | 31.10 |
| 299 | 1,3,5-Trimethoxybenzene | 32.40 | 33.10 |
| 300 | Naphthalene | 16.40 | 15.50 |
| 301 | Benz[a]anthracene | 13.70 | 13.60 |
| 302 | Tetracosanoic acid | 82.30 | 68.00 |
| 303 | 1-(4-Nitrophenylethynyl)-4-pentyloxybenzene | 33.60 | 27.50 |
| 304 | 2-Nitrobenzamide | 19.60 | 17.70 |
| 305 | 4-Amino-N-(5-chloro-2-methylphenyl)benzenesulfonamide | 26.00 | 28.70 |
| 306 | Tetradecane | 46.00 | 36.10 |
| 307 | 1,2,4,5,6-Pentachloronaphthalene | 24.10 | 27.40 |
| 308 | Undecanedioic acid | 42.80 | 40.50 |
| 309 | 2-(4-Nitrophenyl)-1-(4-ethoxyphenyl)ethanone | 28.20 | 30.30 |
| 310 | 2,2'-Bipyridine | 14.80 | 14.20 |
| 311 | 1-Octanol | 31.10 | 32.40 |
| 312 | 2,2'-Bithiophene | 16.50 | 22.20 |
| 313 | Diphenylmethane | 19.00 | 17.10 |
| 314 | Triphenylene | 14.00 | 13.60 |
| 315 | 3,4-Difluoronitrobenzene | 18.30 | 16.60 |
| 316 | Nonadecane | 47.80 | 44.10 |
| 317 | Ferulic acid | 17.90 | 23.20 |
| 318 | p-Toluamide | 18.20 | 16.50 |
| 319 | 1,2,7,8-Tetrachlorodibenzofuran | 39.10 | 31.10 |
| 320 | 2,4-Dichlorophenoxyacetic acid | 32.00 | 33.10 |
| 321 | Pyrene | 12.10 | 12.20 |
| 322 | 1,2,3,5,8-Pentachloronaphthalene | 23.80 | 27.40 |
| 323 | Piperazine | 16.70 | 15.40 |
| 324 | m-Toluamide | 18.30 | 16.50 |
| 325 | 4-Methoxyphenol | 14.70 | 21.10 |
| 326 | 2,2'-Dihydroxybenzophenone | 20.10 | 17.70 |
| 327 | Methyl octacosanoate | 103.80 | 83.40 |
| 328 | 1,5,9-Trithiacyclododecane | 31.40 | 32.80 |
| 329 | Cholest-5-en-3-ol | 26.50 | 29.40 |
| 330 | 2,5-Dibromoaniline | 20.47 | 25.20 |
| 331 | 1,2,4,5-Tetraisopropylbenzene | 19.60 | 24.60 |
| 332 | Formamide | 11.60 | 11.70 |
| 333 | Pimelonitrile | 15.00 | 21.40 |
| 334 | 4-Iodoacetophenone | 21.80 | 18.80 |
| 335 | Diphenylamine | 19.80 | 17.40 |
| 336 | 2,3,7,8-Tetrachlorodibenzofuran | 39.40 | 31.10 |
| 337 | 4-Methylbenzylalcohol | 21.11 | 18.30 |
| 338 | 1-Naphthylamine | 14.80 | 21.30 |
| 339 | Hexamethyleneglycol | 25.50 | 28.80 |
| 340 | Tetrahydrodicyclopentadiene | 4.32 | 14.00 |
| 341 | 4-Ethoxyisonitrosoacetanilide | 7.60 | 8.80 |
| 342 | 1,4-Benzoquinone | 18.40 | 16.30 |
| 343 | 1-Methyl-3,5-diphenylpyrazole | 17.50 | 23.30 |
| 344 | 2,4-Dinitrobenzaldehyde | 21.20 | 25.90 |
| 345 | 2,3-Dichloroanisole | 22.04 | 26.50 |
| 346 | 2-t-Butyl-4-methylphenol | 17.20 | 15.40 |
| 347 | 4-Nitrobenzamide | 20.50 | 17.70 |
| 348 | trans-Cyclohexyl-1,4-diamine | 27.00 | 30.00 |
| 349 | 4,5-Dicyano-1-methylimidazole | 9.50 | 10.00 |
| 350 | 8-Quinolinol | 22.10 | 18.80 |
| 351 | 2-(4-Nitrophenyl)-1-(4-(4-ethylcyclohexyl)phenyl)ethanone | 37.30 | 29.40 |
| 352 | 4-t-Butylbenzoic acid | 9.20 | 17.60 |
| 353 | trans-Decahydroquinoline | 24.60 | 20.50 |
| 354 | 1,2,3-Trichlorobenzene | 17.90 | 23.70 |
| 355 | Monomethyl phthalate | 21.60 | 26.30 |
| 356 | Vitavax | 28.20 | 23.00 |
| 357 | trans-Cinnamic acid | 22.20 | 18.80 |
| 358 | N,N,N-Tris(3-methylphenyl)amine | 13.07 | 12.40 |
| 359 | 2-Chlorobenzoic acid | 25.30 | 20.90 |
| 360 | m-Cresol | 6.90 | 16.10 |
| 361 | 1,2,4,7,8-Pentachlorodibenzofuran | 43.20 | 33.40 |
| 362 | Ethriol | 17.30 | 23.40 |
| 363 | 3,4-Dichloroaniline | 21.69 | 26.50 |
| 364 | 1,2-Dihydroxybenzene | 16.40 | 14.60 |
| 365 | 4-Amino-N-(4-ethylphenyl)benzenesulfonamide | 24.80 | 28.70 |
| 366 | 5-nitro-1,3-Benzodioxole | 28.20 | 31.10 |
| 367 | 1,2,3,6,7-Pentachloronaphthalene | 22.90 | 27.40 |
| 368 | 1,3,5-Trichlorobenzene | 17.60 | 23.70 |
| 369 | Benzo[b]triphenylene | 13.60 | 12.60 |
| 370 | 3,5-Di-t-butylsalicylic acid | 22.92 | 19.10 |
| 371 | 2,3:7,8-Dibenzocycloocta-2,7-dien-1-one | 20.80 | 17.60 |
| 372 | Methylacetamide | 11.40 | 19.40 |
| 373 | Octachlorodibenzo-p-dioxin | 59.00 | 52.70 |
| 374 | Hydrocortisone hemisuccinate | 41.30 | 31.90 |
| 375 | 2,2-Dimethoxybutane | 9.32 | 18.00 |
| 376 | 2-(4-Nitrophenyl)-1-(4-(4-propylcyclohexylethyl)phenyl)ethanone | 35.60 | 36.40 |
| 377 | Azulene | 15.20 | 13.60 |
| 378 | 5-Nitro-8-hydroxyquinoline | 24.70 | 20.20 |
| 379 | Octacosane | 67.00 | 58.40 |
| 380 | Propanamide | 12.90 | 20.60 |
| 381 | Propyl 4-hydroxybenzoate | 27.20 | 30.60 |
| 382 | 2-Isopropyl-5-methylphenol | 20.60 | 17.30 |
| 383 | 1,2,4,6,7-Pentachloronaphthalene | 22.60 | 27.40 |
| 384 | Methyl hexacosanoate | 96.40 | 79.00 |
| 385 | Cannabinol | 17.00 | 23.50 |
| 386 | Salicylic acid | 24.50 | 20.00 |
| 387 | 2-Acetoxyacetophenone | 30.30 | 32.80 |
| 388 | 2-Bromobenzoic acid | 24.80 | 20.20 |
| 389 | Piperonylonitrile | 20.80 | 26.20 |
| 390 | 4-t-Butylphenol | 18.00 | 15.40 |
| 391 | Silybin | 44.90 | 34.20 |
| 392 | 2-Methyl-2-butanol | 6.60 | 16.30 |
| 393 | 3-Bromobenzoic acid | 24.90 | 20.20 |
| 394 | Acenaphthene | 16.20 | 14.10 |
| 395 | 1,2,3,6,7,8-Hexachlorodibenzo-p-dioxin | 51.90 | 48.00 |
| 396 | 3,5-Di-t-butyltoluene | 18.50 | 15.70 |
| 397 | 1,8-Dimethylnaphthalene | 18.53 | 15.70 |
| 398 | 1,2,3,4-Tetrahydrodibenzothiophene | 32.00 | 25.10 |
| 399 | 18-Methylpentatriacontane | 99.00 | 71.90 |
| 400 | 4-Amino-N-(2,5-dichlorophenyl)benzene sulfonamide | 27.60 | 31.10 |
| 401 | Bicyclopentyl | 13.70 | 21.40 |
| 402 | 1-Adamantanecarboxylic acid | 11.40 | 19.80 |
| 403 | 1,2,3,4,7,8-Hexachlorodibenzo-p-dioxin | 51.70 | 48.00 |
| 404 | 4-Pyridinemethanol | 11.78 | 20.10 |
| 405 | 4-Bromoaniline | 16.75 | 23.60 |
| 406 | 2-Oxaadamantane | 8.10 | 17.60 |
| 407 | 3-Methyl-4-hydroxybenzaldehyde | 22.40 | 27.60 |
| 408 | 16-Hentriacontanone | 117.10 | 84.40 |
| 409 | Isopropyl t-butyl ether | 8.50 | 17.90 |
| 410 | Benzil | 22.90 | 18.50 |
| 411 | Sulfacetamide | 29.80 | 23.30 |
| 412 | Xylitol | 33.26 | 25.70 |
| 413 | 2,4,6-Trichlorophenol | 15.10 | 22.60 |
| 414 | Benzo[k]fluoranthene | 6.80 | 7.20 |
| 415 | 4-Bromo-4'-(3-butenyloxy)biphenyl | 29.10 | 32.40 |
| 416 | Suberic acid | 31.10 | 33.80 |
| 417 | Monuron | 29.30 | 22.90 |
| 418 | Hexadecanamide | 55.80 | 51.10 |
| 419 | Pimelic acid | 27.90 | 31.60 |
| 420 | 8-Methylquinoline | 10.70 | 19.60 |
| 421 | Methyl 4-chlorobenzoate | 21.00 | 26.80 |
| 422 | 2-Bromofluorene | 15.50 | 13.20 |
| 423 | Fenofibrate | 32.40 | 34.80 |
| 424 | 1,3-Diphenylacetone | 27.00 | 21.20 |
| 425 | 1,13-Tridecanedioic acid | 49.40 | 46.70 |
| 426 | 1,5-Dimethylnaphthalene | 20.00 | 16.30 |
| 427 | 1-(4-Nitrophenylethynyl)-4-ethoxybenzene | 26.02 | 20.50 |
| 428 | 1,4-Dihydroxybenzene | 17.60 | 14.60 |
| 429 | 2,4,6-Trinitrotoluene | 26.20 | 20.60 |
| 430 | 1,2,3,7,8,9-Hexachlorodibenzo-p-dioxin | 51.20 | 48.00 |
| 431 | 4-t-Butylcatechol | 2.80 | 14.20 |
| 432 | N-(4-Acetylphenyl)-4H-3,1-benzothiazin-2-amine | 16.10 | 23.50 |
| 433 | 2,2'-Dinitrodiphenylamine | 18.36 | 25.10 |
| 434 | N-(2-Methyl-4-chlorophenyl)-4H-3,1-benzothiazin-2-amine | 17.50 | 24.50 |
| 435 | 2-Naphthol | 18.00 | 14.80 |
| 436 | 1,2,4,6,7,9-Hexachlorodibenzo-p-dioxin | 51.10 | 48.00 |
| 437 | 2-(4-Nitrophenyl)-1-(4-(4-pentylcyclohexyl)phenyl)ethanone | 34.50 | 36.40 |
| 438 | 2-Bromoaniline | 16.14 | 23.60 |
| 439 | Ethyl 5-phenylsulfonyl-4,7-dihydro-4,7-ethano-2H-isoindole-1-carboxylate | 29.80 | 23.00 |
| 440 | 4-Fluorobenzamide | 19.50 | 15.80 |
| 441 | 9-Methyl-9H-fluorene | 12.40 | 10.80 |
| 442 | 2-Methylacetanilide | 21.70 | 17.30 |
| 443 | 4-Amino-N-(2,3-dichlorophenyl)benzene sulfonamide | 26.80 | 31.10 |
| 444 | 4-Iodobenzoic acid | 23.30 | 18.40 |
| 445 | 2,3,4,7,8-Pentachlorodibenzofuran | 44.80 | 33.40 |
| 446 | 1,2-Dihydro-1,6-dimethylphenanthro[1,2-b]furan-10,11-dione | 22.20 | 17.60 |
| 447 | 4-Ethylbenzoic acid | 11.00 | 20.10 |
| 448 | Undecanedinitrile | 26.00 | 30.60 |
| 449 | Methyl 4-dimethylaminobenzoate | 19.10 | 25.80 |
| 450 | Glutaric acid | 21.10 | 27.20 |
| 451 | 2-Iodofluorene | 14.60 | 12.20 |
| 452 | 1-Phenyl-4,7-dioxaspiro[2.4]heptane | 20.50 | 16.30 |
| 453 | Amitraz | 19.50 | 15.60 |
| 454 | Cannabidiol | 28.40 | 32.40 |
| 455 | 2,7-Dichlorodibenzo-p-dioxin | 37.40 | 38.70 |
| 456 | m-Anisic acid | 15.50 | 23.40 |
| 457 | 2,3,7,8-Tetrachlorodibenzo-p-dioxin | 44.10 | 43.40 |
| 458 | 4-Acetylanisole | 15.90 | 23.70 |
| 459 | N-Cyclohexyl-2,4,6-trimethylbenzaldehyde imine | 26.20 | 30.90 |
| 460 | 1,4,7,10,13-Pentathiacyclopentadecane | 32.20 | 35.10 |
| 461 | 1,6-Hexanediamine | 40.20 | 40.70 |
| 462 | 2-Benzoylpyridine | 20.91 | 16.50 |
| 463 | 4-t-Butylbiphenyl | 18.10 | 14.50 |
| 464 | 2-Adamantanol | 22.40 | 17.50 |
| 465 | 2-Nitrobenzonitrile | 17.30 | 13.90 |
| 466 | Octyl urea | 36.20 | 38.00 |
| 467 | 1,4,7,10,13,16-Hexaoxacyclooctadecane | 53.60 | 50.20 |
| 468 | Anthracene | 18.40 | 14.60 |
| 469 | Diphenylcarbonate | 26.70 | 20.40 |
| 470 | Dimethyl carbonate | 11.60 | 9.80 |
| 471 | N,N-Dimethylnicotinamide | 17.90 | 14.20 |
| 472 | 9,10-Dimethylanthracene | 18.50 | 14.60 |
| 473 | 1,2,3,7,8-Pentachlorodibenzofuran | 45.40 | 33.40 |
| 474 | Methyl trans-cinnamate | 33.10 | 24.80 |
| 475 | Decanamide | 35.00 | 37.30 |
| 476 | Neopentyl alcohol | 8.50 | 18.80 |
| 477 | 2-Iodonaphthalene | 21.00 | 16.30 |
| 478 | 1-Nitro-2,6-diisopropylbenzene | 12.20 | 21.40 |
| 479 | 1,2,3,5-Tetrachlorobenzene | 18.90 | 26.10 |
| 480 | o-Terphenyl | 16.60 | 13.20 |
| 481 | 3,4,5-Trimethoxybenzoic acid | 29.90 | 33.80 |
| 482 | 1,1-Diethoxyethane | 11.00 | 20.60 |
| 483 | 1,3-Dimethyladamantane | 10.90 | 9.20 |
| 484 | Chrysene | 17.20 | 13.60 |
| 485 | 4-Chlorobiphenyl | 6.70 | 17.60 |
| 486 | Benzal diacetate | 27.30 | 32.00 |
| 487 | Azelanitrile | 18.70 | 26.00 |
| 488 | Methylbenzene | 5.10 | 16.50 |
| 489 | 1-Methylindole | 12.10 | 10.00 |
| 490 | 1,3,5-Triphenylbenzene | 14.40 | 11.60 |
| 491 | 3,4-Dichlorophenol | 10.50 | 20.30 |
| 492 | 2-Bromoacetophenone | 26.00 | 19.70 |
| 493 | Cetane | 53.90 | 39.20 |
| 494 | 1-Methylfluorene | 14.00 | 11.30 |
| 495 | N,N-Dimethylformamide | 8.90 | 7.70 |
| 496 | 1,2,3,4-Tetrachlorobenzene | 18.70 | 26.10 |
| 497 | 4-(2,4-Dichlorophenoxy)butyric acid | 35.00 | 37.50 |
| 498 | 1,8-Diaminonaphthalene | 19.40 | 26.60 |
| 499 | 4-Chloro-3-nitrophenol | 25.97 | 19.60 |
| 500 | 4,4'-Dichlorobiphenyl | 9.80 | 19.90 |
| 501 | 4-Fluorobenzonitrile | 15.40 | 12.20 |
| 502 | 2-Piperidone | 8.20 | 18.80 |
| 503 | 4-t-Pentylphenol | 23.00 | 17.50 |
| 504 | 2-Chloroacetophenone | 27.60 | 20.70 |
| 505 | 2-Furoic acid | 22.60 | 17.20 |
| 506 | [2,2]Paracyclophane-1-ene | 17.60 | 13.70 |
| 507 | 2-Phenylnaphthalene | 17.90 | 13.90 |
| 508 | Nonyl urea | 38.90 | 40.30 |
| 509 | Pentafluorophenol | 15.20 | 12.00 |
| 510 | Phenylcarbamate | 22.12 | 28.60 |
| 511 | 9-Phenylanthracene | 16.70 | 13.00 |
| 512 | Hesperetin | 35.90 | 26.40 |
| 513 | Acetopiperone | 26.20 | 31.50 |
| 514 | 1-Benzylimidazole | 22.90 | 17.30 |
| 515 | 1H-Indene-1,3(2H)-dione | 21.80 | 16.50 |
| 516 | n-Decyl alcohol | 33.70 | 36.80 |
| 517 | Diethylcarbonate | 9.20 | 19.70 |
| 518 | Bromoacetic acid | 13.90 | 23.00 |
| 519 | 3-Fluorobenzamide | 20.90 | 15.80 |
| 520 | 4,7-Phenanthroline | 30.20 | 22.30 |
| 521 | trans-2-Methoxycinnamic acid | 32.50 | 23.90 |
| 522 | Pivalamide | 24.10 | 18.00 |
| 523 | 9H-Fluoren-9-one | 14.10 | 11.00 |
| 524 | 4-Methoxy-alpha-methylstyrene | 19.10 | 26.70 |
| 525 | Norbornane | 4.45 | 16.50 |
| 526 | trans-Stilbene | 19.90 | 15.00 |
| 527 | 2-Biphenyl methanol | 21.50 | 16.10 |
| 528 | 5H-10,11-Dihydrodibenzo[a,d]cyclohexane-5-ol | 19.00 | 14.30 |
| 529 | Di-t-butyl-isopropylmethanol | 2.30 | 15.10 |
| 530 | Azacyclononan-2-one | 17.00 | 25.40 |
| 531 | Cyclotetradecane | 30.30 | 34.70 |
| 532 | 2-Bromo-7-chlorofluorene | 20.80 | 15.50 |
| 533 | Tamoxifen | 34.00 | 37.30 |
| 534 | 1,2,4,6,8-Pentachloronaphthalene | 19.80 | 27.40 |
| 535 | Heptadecane | 39.10 | 40.90 |
| 536 | Behenyl alcohol | 71.10 | 63.30 |
| 537 | 2-(4-Nitrophenyl)-1-(4-(4-butylcyclohexylethyl)phenyl)ethanone | 35.90 | 38.70 |
| 538 | p-Bromoacetanilide | 25.80 | 18.90 |
| 539 | 1-(4-Nitrophenylethynyl)-4-propoxybenzene | 31.40 | 22.80 |
| 540 | 1,8-Octanediamine | 45.00 | 45.10 |
| 541 | Glibenclamide | 46.30 | 33.20 |
| 542 | 2,4-Dichlorobenzaldehyde | 20.50 | 28.00 |
| 543 | 1,4,8,11-Tetrathiacyclohexadecane | 37.20 | 39.70 |
| 544 | 4-Nitrobenzylideneaniline | 24.60 | 30.90 |
| 545 | Monomethyl isophthalate | 36.50 | 26.30 |
| 546 | 2,7-Dimethylnaphthalene | 22.20 | 16.30 |
| 547 | 1-Dodecanol | 39.30 | 41.20 |
| 548 | Nicotinic acid | 28.38 | 20.60 |
| 549 | 1,4-Di-t-butylbenzene | 21.40 | 15.70 |
| 550 | 1H,1H-Perfluorotetradecan-1-ol | 32.40 | 36.40 |
| 551 | alpha-Phenyl-1-piperidinoacetonitrile | 19.70 | 14.50 |
| 552 | Estra-1,3,5(10)-triene-3,17-diol(17beta) 3-benzoate | 41.80 | 29.90 |
| 553 | Dimethylterephthalate | 26.30 | 32.20 |
| 554 | Dodecyl urea | 47.90 | 47.30 |
| 555 | 2,4-Di-t-butylphenol | 20.50 | 15.00 |
| 556 | Atenolol | 38.70 | 27.70 |
| 557 | Methyl tetracosanoate | 86.90 | 74.60 |
| 558 | Norbornene | 3.60 | 16.40 |
| 559 | Acetanilide | 18.30 | 13.40 |
| 560 | 1,2,6,7,8,9-Hexahydro-1,6,6-trimethylphenanthro[1,2-b]furan-10,11-dione | 26.50 | 19.10 |
| 561 | 2-Fluorobenzamide | 21.80 | 15.80 |
| 562 | Cyclohexadecane | 53.30 | 37.80 |
| 563 | Triphenylethylene | 20.40 | 14.80 |
| 564 | Camphor | 2.50 | 15.70 |
| 565 | 2-(1'-Hydroxycyclohexyl)cyclohexanone | 20.80 | 28.50 |
| 566 | 1,2,3,7,8-Pentachlorodibenzo-p-dioxin | 45.40 | 45.70 |
| 567 | 1-Hydroxycumene | 17.30 | 12.60 |
| 568 | 2,4,5-Trimethoxybenzoic acid | 31.20 | 35.80 |
| 569 | 1,4-Diaminobutane | 31.90 | 36.30 |
| 570 | 4-Nitrobenzonitrile | 19.20 | 13.90 |
| 571 | Vitamin B3 | 25.50 | 18.30 |
| 572 | 1,4-D-Mannonolactone | 36.10 | 25.70 |
| 573 | Tetracene | 18.80 | 13.60 |
| 574 | 3-Chlorothiophene | 9.39 | 20.60 |
| 575 | 4,4'-Thiodiphenol | 31.00 | 22.10 |
| 576 | Butyl 4-hydroxybenzoate | 26.60 | 32.70 |
| 577 | Myristyl alcohol | 45.00 | 45.60 |
| 578 | m-Anthranilic acid | 33.70 | 23.90 |
| 579 | 6-Heptadecanol | 49.00 | 48.40 |
| 580 | 4-Hydroxyphenylacetic acid | 28.00 | 19.90 |
| 581 | 4,4'-Di-t-butylbiphenyl | 19.80 | 14.10 |
| 582 | 2-Chloro-N,N-dimethylnicotinamide | 21.39 | 15.20 |
| 583 | 2,4-Dinitrophenol | 26.19 | 18.50 |
| 584 | 1,2,4,6,7,8-Hexachlorodibenzofuran | 50.80 | 35.70 |
| 585 | N-(2-Chlorophenyl)benzenesulfonamide | 33.50 | 23.60 |
| 586 | 2,3,8-Trichlorodibenzofuran | 41.00 | 28.80 |
| 587 | 2-Oxa-6-thiaadamantane | 12.23 | 22.90 |
| 588 | 3,3-Dimethyl-1-butanol | 9.50 | 21.00 |
| 589 | 1,2,3,4,7,8-Hexachlorodibenzofuran | 50.90 | 35.70 |
| 590 | Methyl heptacosanoate | 95.60 | 81.20 |
| 591 | 1,2-Dimethylbenzimidazole | 15.00 | 10.60 |
| 592 | Tetradecyl urea | 53.60 | 51.90 |
| 593 | Tridecyl urea | 50.30 | 49.60 |
| 594 | 1,2,3,4,6,8-Hexachlorodibenzofuran | 51.00 | 35.70 |
| 595 | 1,2,3,6,7,8-Hexachlorodibenzofuran | 51.00 | 35.70 |
| 596 | 2-(4-Nitrophenyl)-1-(4-(4-pentylcyclohexylethyl)phenyl)ethanone | 37.95 | 41.00 |
| 597 | 4-Amino-N-(4-nitrophenyl)benzenesulfonamide | 18.90 | 27.70 |
| 598 | Torasemide | 37.20 | 26.00 |
| 599 | 2,3,4,6,7,8-Hexachlorodibenzofuran | 51.10 | 35.70 |
| 600 | 2-Thiopheneacetic acid | 14.00 | 24.30 |
| 601 | N-Propionylpyrazinecarboxamide | 23.50 | 16.40 |
| 602 | 1,2,3,4,7-Pentachlorodibenzo-p-dioxin | 44.60 | 45.70 |
| 603 | 1,4-Dimethoxybenzene | 18.70 | 27.60 |
| 604 | Monomethyl terephthalate | 37.70 | 26.30 |
| 605 | 1,1,2-Triphenylethane | 24.40 | 17.00 |
| 606 | 2,7-Dibromofluorene | 21.00 | 14.60 |
| 607 | N,N,N',N'-Tetramethyloxamide | 18.00 | 12.50 |
| 608 | 1,2,4,6,8,9-Hexachlorodibenzofuran | 51.30 | 35.70 |
| 609 | 2-Butyl-2-ethyl-1,3-propanediol | 20.80 | 29.20 |
| 610 | Pentyl urea | 23.50 | 31.10 |
| 611 | N-(4-Methoxyphenyl)sulfanilamide | 24.60 | 31.90 |
| 612 | Propyl Naltrexone-3-O-carbonate | 21.00 | 14.40 |
| 613 | t-Butyl propyl ether | 9.90 | 21.70 |
| 614 | 1,3-Dihydroxybenzene | 21.30 | 14.60 |
| 615 | 1,2,7,8-Tetrachloronaphthalene | 14.60 | 25.00 |
| 616 | Methyl naltrexone-3-O-carbonate | 10.90 | 7.30 |
| 617 | Pentachlorobenzene | 19.40 | 28.40 |
| 618 | Fluoranthene | 12.10 | 8.10 |
| 619 | Palmitic acid | 72.70 | 50.40 |
| 620 | Decyl urea | 39.60 | 42.60 |
| 621 | 3-Nitrobenzonitrile | 20.50 | 13.90 |
| 622 | Guajen | 23.97 | 16.30 |
| 623 | Pimelamide | 44.60 | 30.70 |
| 624 | Enantholactam | 11.70 | 23.20 |
| 625 | Hexachlorobenzene | 22.40 | 30.70 |
| 626 | 2-(Chloromethyl)benzonitrile | 20.53 | 13.80 |
| 627 | Monacolin K | 43.14 | 45.20 |
| 628 | 1,2,4-Trichlorodibenzo-p-dioxin | 37.10 | 41.00 |
| 629 | Aspirin | 29.20 | 35.50 |
| 630 | 1,2,3,7,8-Pentachloronaphthalene | 17.60 | 27.40 |
| 631 | Isobutyl t-butylether | 8.70 | 21.20 |
| 632 | 2-(Biphen-3-yl)naphthalene | 18.50 | 12.30 |
| 633 | Glutaramide | 38.40 | 26.20 |
| 634 | 2-Bromo-7-iodofluorene | 20.60 | 13.70 |
| 635 | 4-Chlorobenzoic acid | 30.91 | 20.90 |
| 636 | 5,5-Dimethyltetrahydro-1,3-oxazin-2-one | 28.50 | 19.20 |
| 637 | Pentadecane | 32.10 | 37.70 |
| 638 | Penconazole | 33.58 | 22.70 |
| 639 | 1-(4-Nitrophenylethynyl)-4-propylbenzene | 23.30 | 15.50 |
| 640 | Basketene | 2.70 | 1.10 |
| 641 | 6-Hydroxymethyl-5,6-dihydro-7H-dibenzo[a,c]cycloheptane | 25.90 | 17.30 |
| 642 | Fenpropathrin | 18.60 | 28.30 |
| 643 | Methyl arachidate | 72.00 | 65.70 |
| 644 | 1,3,5-Trithiane | 32.30 | 38.00 |
| 645 | Heptyl urea | 29.00 | 35.70 |
| 646 | N,N,N',N'-Tetrapropyloxamide | 21.00 | 13.70 |
| 647 | 2,6-Xylenol | 0.30 | 15.70 |
| 648 | Methyl pentacosanoate | 87.70 | 76.80 |
| 649 | Methyl docosanoate | 78.20 | 70.20 |
| 650 | Butylcyclohexane | 14.20 | 25.50 |
| 651 | 4-(Trifluoromethyl)-7-(N,N-dimethylamino)coumarin | 26.30 | 17.30 |
| 652 | Cholestanole | 22.60 | 31.40 |
| 653 | Octachlorodibenzofuran | 59.40 | 40.40 |
| 654 | 4-Methoxycinnamaldehyde | 19.00 | 28.90 |
| 655 | N-(4-Methoxyphenyl)-4H-3,1-benzothiazin-2-amine | 16.10 | 26.90 |
| 656 | Ethyl naltrexone-3-O-carbonate | 19.00 | 12.10 |
| 657 | Methyl 4-aminobenzoate | 20.20 | 29.80 |
| 658 | 3-Bromoacetophenone | 5.70 | 19.70 |
| 659 | 3-Bromocinnamic acid | 31.10 | 20.50 |
| 660 | 2-Chloroaniline | 12.40 | 24.40 |
| 661 | Alemcinal | 36.70 | 24.40 |
| 662 | n-Butylurea | 17.80 | 28.20 |
| 663 | 1-Methylbenzimidazole | 12.40 | 7.40 |
| 664 | Cyclodecane | 19.00 | 29.10 |
| 665 | 2-Cyano-4'-methylbiphenyl | 18.79 | 11.80 |
| 666 | m-Terphenyl | 20.80 | 13.20 |
| 667 | s-Triazine | 15.40 | 9.40 |
| 668 | Octadecanamide | 57.00 | 55.70 |
| 669 | Diphenylmethanol | 22.70 | 14.50 |
| 670 | 2,6-Dimethylnaphthalene | 25.30 | 16.30 |
| 671 | Malonamide | 31.74 | 20.80 |
| 672 | Propanolamine | 16.90 | 27.70 |
| 673 | Tolfenamic acid | 41.20 | 27.40 |
| 674 | 4-Bromobenzoic acid | 30.90 | 20.20 |
| 675 | 1,2,3,4,6,8,9-Heptachlorodibenzofuran | 56.40 | 38.00 |
| 676 | 4-Trifluoromethyl-7-aminocoumarin | 31.70 | 20.70 |
| 677 | Carvedilol | 57.60 | 38.80 |
| 678 | Propylurea | 14.30 | 26.00 |
| 679 | Tri-p-tolylamine | 19.95 | 12.40 |
| 680 | 1,2,3,4,7,8,9-Heptachlorodibenzofuran | 56.60 | 38.00 |
| 681 | 1,7-Heptanediamine | 38.20 | 42.80 |
| 682 | p-Quaterphenyl | 18.90 | 11.60 |
| 683 | Ethyl vanillin | 23.10 | 32.30 |
| 684 | 2-Ethylhexyl-alpha-D-glucoside | 37.00 | 24.20 |
| 685 | Hexacosanoic acid | 80.60 | 72.50 |
| 686 | 3,6-Dichlorodibenzofuran | 40.20 | 26.40 |
| 687 | Flufenamic acid | 19.60 | 12.00 |
| 688 | 4-Methoxyisonitrosoacetanilide | 8.30 | 4.10 |
| 689 | 1,2,3,4,6,7,8-Heptachlorodibenzofuran | 56.80 | 38.00 |
| 690 | 4-t-Octylphenol | 27.20 | 17.30 |
| 691 | 1,3,7,8-Tetrachlorodibenzo-p-dioxin | 38.90 | 43.40 |
| 692 | N-Caproyl-pyrazinamide | 35.95 | 23.40 |
| 693 | Ethyl 8-(hydroxymethyl)-6-chloro-2-oxo-2H-1-benzopyran-3-carboxylate | 38.00 | 42.80 |
| 694 | Methylurea | 12.50 | 25.00 |
| 695 | 4-Bromo-4'-(5-hexenyloxy)biphenyl | 29.80 | 37.10 |
| 696 | Methyl hexadecanoate | 58.10 | 56.90 |
| 697 | Erythritol | 29.10 | 18.50 |
| 698 | Ethylcyclohexane | 8.50 | 22.30 |
| 699 | 3-Furancarboxylic acid | 21.30 | 13.00 |
| 700 | Pyracene | 20.90 | 12.70 |
| 701 | 3,5-Di-t-butyl-o-quinone | 26.50 | 16.60 |
| 702 | Paracetamol | 26.50 | 16.60 |
| 703 | 1-Chloro-4-fluorobenzene | 29.10 | 18.40 |
| 704 | Niclosamide | 36.00 | 23.20 |
| 705 | Metazachlor | 19.70 | 11.80 |
| 706 | Dimethyl malonate | 54.20 | 35.90 |
| 707 | 1,2,3,7-Tetrachlorodibenzo-p-dioxin | 38.50 | 43.40 |
| 708 | Homopiperonylic acid | 24.90 | 33.90 |
| 709 | Sorbitol | 30.35 | 19.20 |
| 710 | 1,9-Nonanediamine | 44.00 | 47.30 |
| 711 | 2-Hydroxybenzamide | 29.00 | 18.20 |
| 712 | Propylcyclohexane | 10.60 | 24.00 |
| 713 | Hexadecanedioic acid | 52.20 | 53.10 |
| 714 | Dimethyl isophthalate | 22.30 | 32.20 |
| 715 | 1-Aminoadamantane | 1.80 | 17.90 |
| 716 | 4-(Trifluoromethyl)-7-(N-ethylamino)coumarin | 30.50 | 19.10 |
| 717 | Flurbiprofen | 28.10 | 17.40 |
| 718 | 2-Phenylsuccinic acid | 37.40 | 23.90 |
| 719 | Indometacin | 32.90 | 20.70 |
| 720 | 5-Hydroxymethylene-5H-6,7-dihydrodibenzo[a.c]cyclohepten-6-one | 16.90 | 9.50 |
| 721 | 6,7,8,9-Tetrahydro-6-(hydroxymethyl)-1,6-dimethylphenanthro[1,2-b]furan-10,11-dione | 23.70 | 14.20 |
| 722 | N,N’-Bis(salicylaldehydo)ethylenediimine | 34.10 | 40.70 |
| 723 | 4-(1-Methylheptyloxy)-4'-cyanobiphenyl | 20.80 | 31.50 |
| 724 | Dimethyl 2,6-cuneanedicarboxylate | 23.40 | 13.80 |
| 725 | 2-Methylnaphthalene | 26.00 | 15.60 |
| 726 | 1,2,3,4,5,6,7-Heptafluoronaphthalene | 17.30 | 9.50 |
| 727 | Ethylurea | 9.60 | 23.80 |
| 728 | 1,3-Benzoxazine-2,4-dione | 28.63 | 17.40 |
| 729 | 2-Fluorofluorene | 19.50 | 11.00 |
| 730 | Eicosane | 69.00 | 45.60 |
| 731 | Methyl 4-methoxybenzoate | 18.40 | 30.00 |
| 732 | 1,3-Benzodioxol-5-ol | 16.96 | 29.00 |
| 733 | N-Decanoylpyrazinamide | 50.58 | 32.70 |
| 734 | 4-Biphenylmethanol | 27.00 | 16.20 |
| 735 | Nonadecylcyclohexane | 77.79 | 51.70 |
| 736 | 2,3-Dichlorodibenzo-p-dioxin | 30.80 | 38.70 |
| 737 | 9-Fluorenylmethanol | 21.00 | 12.00 |
| 738 | Tridecane | 24.70 | 34.50 |
| 739 | 1-Phenyl-1,3-butanedione | 35.40 | 22.00 |
| 740 | 1-Heptanol | 18.40 | 30.10 |
| 741 | Methyl heneicosanoate | 72.60 | 68.00 |
| 742 | N-(3-Methylphenyl)-N,N-bis(4-methylphenyl)amine | 21.71 | 12.40 |
| 743 | 3-Amino-2-methylbenzoic acid | 38.50 | 24.10 |
| 744 | 3-Acetoxyacetophenone | 22.10 | 32.80 |
| 745 | 2,7-Difluorofluorene | 18.80 | 10.20 |
| 746 | Biphenylene | 17.40 | 9.20 |
| 747 | 4-Trifluoromethyl-7-(diethylamino)coumarin | 23.30 | 13.30 |
| 748 | 2-(4-Nitrophenyl)-1-(4-butoxyphenyl)ethanone | 24.90 | 34.90 |
| 749 | 1-Propanol | 5.40 | 21.30 |
| 750 | Azelamide | 55.00 | 35.30 |
| 751 | 1H,1H-Perfluorododecan-1-ol | 20.10 | 31.70 |
| 752 | 1,5-Diaminopentane | 29.80 | 38.50 |
| 753 | N,N-Dimethylbenzamide | 22.30 | 12.40 |
| 754 | Phenoxathiin | 19.40 | 31.30 |
| 755 | Dimethyl oxalate | 22.80 | 33.70 |
| 756 | Quinoxaline | 7.90 | 23.30 |
| 757 | Naltrexone | 15.60 | 7.60 |
| 758 | Hexyl urea | 22.40 | 33.50 |
| 759 | 4-Hydroxy-2-pyrrolidone | 23.40 | 13.00 |
| 760 | 2,7-Diiodofluorene | 23.10 | 12.70 |
| 761 | 2-Carboxyadamantane | 4.50 | 21.10 |
| 762 | 9-Hydroxyfluorene | 17.40 | 8.70 |
| 763 | Ethyl 8-(hydroxymethyl)-6-methyl-2-oxo-2H-1-benzopyran-3-carboxylate | 32.50 | 40.70 |
| 764 | 1,2,3-Benzenetriol | 25.90 | 14.60 |
| 765 | Sulfathiazole | 30.30 | 39.20 |
| 766 | Piperonyl alcohol | 18.10 | 30.70 |
| 767 | Barbituric acid | 22.17 | 11.90 |
| 768 | 1,3-Diaminopropane | 22.70 | 34.00 |
| 769 | 4-Carboxyphenol | 30.85 | 17.90 |
| 770 | Undecyl urea | 38.40 | 45.00 |
| 771 | 1,11-Undecanediamine | 48.10 | 51.80 |
| 772 | Salol | 19.20 | 31.60 |
| 773 | 9-Heptadecanol | 43.20 | 48.40 |
| 774 | 1,6-Dimethylphenanthro[1,2-b]furan-10,11-dione | 22.10 | 11.70 |
| 775 | Methyl octadecanoate | 61.60 | 61.30 |
| 776 | 2-(4-Nitrophenyl)-1-(4-pentyloxyphenyl)ethanone | 27.10 | 37.20 |
| 777 | 4-Acetoxyacetophenone | 20.70 | 32.80 |
| 778 | Tranilast | 48.90 | 30.30 |
| 779 | 1,2,4-Trichlorobenzene | 7.60 | 23.70 |
| 780 | 2-Adamantanone | 2.30 | 20.00 |
| 781 | Snoutene | 7.87 | 1.60 |
| 782 | 1-Pentanol | 10.50 | 25.80 |
| 783 | 3-Aminophenylacetic acid | 42.70 | 25.80 |
| 784 | 2-Thiaadamantane | 10.10 | 25.60 |
| 785 | Methyl 3-aminobenzoate | 16.10 | 29.80 |
| 786 | Cyclohexanone oxime | 12.70 | 4.80 |
| 787 | Di-t-butyl oxalate | 21.30 | 33.50 |
| 788 | Camphene | 2.30 | 20.30 |
| 789 | 19-Oxaheptatriacontane | 116.70 | 100.30 |
| 790 | Methyl nonadecanoate | 63.80 | 63.50 |
| 791 | 2,3,7-Trichlorodibenzo-p-dioxin | 31.60 | 41.00 |
| 792 | 17-Methyltestosterone | 27.80 | 15.00 |
| 793 | 3-Octyloxybenzoic acid | 33.12 | 42.10 |
| 794 | Estrone propionate | 23.00 | 35.10 |
| 795 | 3,4-Dimethoxybenzaldehyde | 20.30 | 33.30 |
| 796 | Stearic acid | 50.90 | 54.70 |
| 797 | Dibenzo-p-dioxin | 21.20 | 34.00 |
| 798 | Imidazole | 14.10 | 29.10 |
| 799 | 1-Nonadecanoic acid | 54.00 | 57.00 |
| 800 | Isoniazid | 27.90 | 38.80 |
| 801 | Dicyclohexylsulfide | 15.70 | 30.30 |
| 802 | Chrysin | 39.20 | 22.40 |
| 803 | Undecanediamide | 64.40 | 40.00 |
| 804 | Cyclohexylacetic acid | 13.80 | 29.20 |
| 805 | 1,3-Dithiane sulfone | 22.00 | 10.30 |
| 806 | 2-(4-Biphenylyl)naphthalene | 25.10 | 12.30 |
| 807 | 7-Norbornanone | 0.60 | 20.20 |
| 808 | Tulobuterol | 25.40 | 12.40 |
| 809 | Ethyl 8-(hydroxymethyl)-6-methoxy-2-oxo-2H-1-benzopyran-3-carboxylate | 36.40 | 45.40 |
| 810 | 1,2,4-Trihydroxybenzene | 28.80 | 14.60 |
| 811 | B-Carotene | 56.00 | 33.60 |
| 812 | Ethyl 4-aminobenzoate | 21.10 | 34.80 |
| 813 | 2-Chloro-3-(trifluoromethyl)pyridine | 18.86 | 7.60 |
| 814 | Myclobutanil | 30.90 | 16.00 |
| 815 | 4,4'-Bipyridine | 35.20 | 19.00 |
| 816 | Methyl adamantyl-1-carboxylate | 7.70 | 25.50 |
| 817 | Fenbufen | 41.10 | 23.10 |
| 818 | 4-Methyl-4-cyclohexene-1,2-dicarboxylic anhydride | 17.70 | 32.50 |
| 819 | 1,3,7-Trichlorodibenzo-p-dioxin | 29.80 | 41.00 |
| 820 | trans-9,10-Bishydroxymethyl-9,10-dihydrophenanthrene | 30.30 | 15.50 |
| 821 | cis-Verbenol | 26.30 | 12.70 |
| 822 | N,N'-Bis(salicylaldehydo)tetramethylenediimine | 35.50 | 45.00 |
| 823 | 4-Butylphenyl-(6-(4-butylphenyl)aminomethylene-9-methyl-9H-carbazol-3-ylmethylene)-amine | 29.60 | 40.90 |
| 824 | 2,3-Dimethyl-2,3-dinitrobutane | 37.80 | 20.70 |
| 825 | Acridone | 32.50 | 16.90 |
| 826 | Cyclohexanecarboxylic acid | 9.20 | 26.80 |
| 827 | Propyl 2-cyano-6-methoxy-1(2H)-quinolinecarboxylate | 15.60 | 5.00 |
| 828 | 2,3-Pyrazinedicarbonitrile | 19.80 | 7.90 |
| 829 | (11Z)-Eicosenoic acid | 49.40 | 55.00 |
| 830 | N,N-Bis(3-methylphenyl)-N-(4-methylphenyl)amine | 26.39 | 12.40 |
| 831 | 1-Pentadecanoic acid | 39.40 | 48.10 |
| 832 | Pentacosane | 47.20 | 53.60 |
| 833 | Isatin | 27.80 | 13.30 |
| 834 | Docosanamide | 63.30 | 64.90 |
| 835 | Dimethyl cubane-1,4-dicarboxylate | 28.70 | 13.80 |
| 836 | Naringenin | 39.80 | 21.40 |
| 837 | 1,3,6,8-Tetrachlorodibenzo-p-dioxin | 32.10 | 43.40 |
| 838 | 2,8-Dichlorodibenzo-p-dioxin | 25.00 | 38.70 |
| 839 | Carbamazepine | 24.90 | 10.60 |
| 840 | Benzo[b]fluorene | 24.50 | 10.30 |
| 841 | 8-Oxabicyclo[5.1.0]octane | 2.20 | 22.90 |
| 842 | p-Acetotoluide | 28.90 | 13.30 |
| 843 | Oxolinic acid | 43.60 | 23.50 |
| 844 | 3-Hexyloxybenzoic acid | 22.70 | 37.40 |
| 845 | n-Butylcarbamate | 16.30 | 33.10 |
| 846 | Tetradecanediamide | 77.50 | 46.90 |
| 847 | 1,1,2,2-Tetra-t-butylethane | 23.50 | 9.10 |
| 848 | N,N,N',N'-Tetraethyloxamide | 17.00 | 4.50 |
| 849 | 6,7,8,9-Tetrahydro-1,6,6-trimethylphenanthro[1,2-b]furan-10,11-dione | 29.20 | 13.00 |
| 850 | trans-5-Hydroxymethyl-5,6-dihydro-7H-dibenzo[a,c]cycloheptan-6-ol | 31.80 | 14.80 |
| 851 | Butyl 4-aminobenzoate | 23.90 | 38.70 |
| 852 | 3-(Butanoyloxy)-estra-1,3,5(10)-trien-17-one | 22.00 | 37.40 |
| 853 | 3,3'-Di-naltrexonyl-carbonate | 17.50 | 4.70 |
| 854 | 9-Acetylcarbazole | 15.10 | 3.00 |
| 855 | 3-Carboxyphenol | 36.50 | 17.90 |
| 856 | Cyclododecane | 13.40 | 31.50 |
| 857 | Erucic acid | 53.10 | 59.30 |
| 858 | 3-(Pentoyloxy)-estra-1,3,5(10)-trien-17-one | 25.00 | 39.70 |
| 859 | Diphenylacetamide | 23.40 | 8.60 |
| 860 | Prednisolone acetate | 42.30 | 21.80 |
| 861 | 1,2,4,7,8-Pentachlorodibenzo-p-dioxin | 33.50 | 45.70 |
| 862 | Ketorolac | 28.62 | 12.20 |
| 863 | Estrone acetate | 15.00 | 32.80 |
| 864 | 9-Ethyl-9H-carbazole | 13.20 | 1.40 |
| 865 | Isopropyl Naltrexone-3-O-carbonate | 26.60 | 10.70 |
| 866 | Ethyl 8-(hydroxymethyl)-6-bromo-2-oxo-2H-1-benzopyran-3-carboxylate | 28.20 | 42.10 |
| 867 | 2-Chlorodibenzo-p-dioxin | 19.90 | 36.30 |
| 868 | Diflunisal | 35.80 | 17.10 |
| 869 | 19-Octatriacontene | 108.37 | 98.20 |
| 870 | Heneicosane | 35.20 | 47.20 |
| 871 | 4-Aminosalicylic acid | 47.90 | 25.20 |
| 872 | N-Methylcarbazole | 16.00 | 2.80 |
| 873 | 3-Decyloxybenzoic acid | 33.90 | 46.70 |
| 874 | trans-Azobenzene | 21.30 | 37.90 |
| 875 | Cycloheptanol | 2.70 | 24.90 |
| 876 | 1-Chlorodibenzo-p-dioxin | 19.00 | 36.30 |
| 877 | (15Z)-Tetracosenoic acid | 58.30 | 63.80 |
| 878 | Valium | 25.49 | 9.20 |
| 879 | 1,4-Oxathiane 4,4-dioxide | 20.20 | 5.50 |
| 880 | 4-Octylbenzoic acid | 14.60 | 33.40 |
| 881 | Hexacosane | 45.50 | 55.10 |
| 882 | 6-Dodecyloxyisoquinoline | 38.90 | 50.50 |
| 883 | o-Anisic acid | 5.70 | 27.30 |
| 884 | Dibenzo-30-crown-10 | 86.50 | 83.80 |
| 885 | 4-Hexylbenzoic acid | 7.90 | 29.00 |
| 886 | Saccharin | 29.90 | 11.80 |
| 887 | Pindolol | 58.00 | 31.30 |
| 888 | Cilostazol | 44.70 | 22.00 |
| 889 | 5-(1,1-Dimethylheptyl)-2-(5-hydroxy-2-(3-hydroxypropyl)-cyclohexyl)phenol | 18.40 | 36.80 |
| 890 | Ethyl eicosanoate | 68.60 | 71.90 |
| 891 | Tetracosane | 40.10 | 52.00 |
| 892 | Cholesteryl 2-methylpropionate | 25.20 | 41.60 |
| 893 | 1,3,5-Trihydroxybenzene | 34.50 | 14.60 |
| 894 | Hexacyclopropylethane | 23.20 | 6.70 |
| 895 | Adipamide | 54.30 | 28.40 |
| 896 | 4-Chlorophenyl 2-cyano-6-methoxy-1(2H)-quinolinecarboxylate | 22.40 | 6.10 |
| 897 | Methyl 2,4-dichlorophenoxyacetate | 20.00 | 38.40 |
| 898 | 2,5-Bis(biphenyl-4-yl)thiophene | 39.00 | 17.20 |
| 899 | Sebacamide | 68.70 | 37.70 |
| 900 | cis-Cyclohexane-1,2-dicarboxylic anhydride | 14.71 | 35.40 |
| 901 | 2-Ethoxyisonitrosoacetanilide | 27.80 | 8.80 |
| 902 | Cyclooctanol | 4.02 | 28.00 |
| 903 | 1,2,3,4-Tetrachlorodibenzo-p-dioxin | 25.70 | 43.40 |
| 904 | 4-Heptadecanol | 35.70 | 50.40 |
| 905 | Quercetin | 41.50 | 18.00 |
| 906 | Methyl heptadecanoate | 48.10 | 59.20 |
| 907 | 3-(Octyloxy)-estra-1,3,5(10)-trien-17-ol | 21.00 | 40.30 |
| 908 | Ethyl 8-(hydroxymethyl)-6-chloro-5,7-dimethyl-2-oxo-2H-1-benzopyran-3-carboxylate | 28.70 | 45.70 |
| 909 | (9Z)-hexadecenoic acid | 29.10 | 46.00 |
| 910 | Ethyl undecanoate | 36.20 | 51.10 |
| 911 | Cyclododecanone | 17.70 | 38.20 |
| 912 | 3-(Hexoyloxy)-estra-1,3,5(10)-trien-17-one | 23.00 | 42.00 |
| 913 | 1,3,5-Trimethyladamantane | 26.10 | 6.60 |
| 914 | Fenoxycarb | 26.98 | 45.00 |
| 915 | Suberamide | 63.90 | 33.00 |
| 916 | Hydrocortisone acetate | 53.64 | 25.80 |
| 917 | [2.2]Paracyclophane-1,9-diene | 30.70 | 9.70 |
| 918 | 1-Heptadecanol | 37.00 | 52.20 |
| 919 | 3,4,5,6-Tetrahydrophthalic anhydride | 11.90 | 34.70 |
| 920 | 2-Bromoethyl 2-cyano-6-nitro-1(2H)-quinolinecarboxylate | 19.80 | 1.90 |
| 921 | Prazepam | 27.70 | 7.30 |
| 922 | Phenyl 2-cyano-6-methoxy-1(2H)-quinolinecarboxylate | 22.90 | 3.70 |
| 923 | 2,5-Dimethyl-3-hexyne-2,5-diol | 23.90 | 4.30 |
| 924 | 3'-(Nitrooxymethyl)phenyl 2-acetoxybenzoate | 33.80 | 50.70 |
| 925 | Tristearin | 195.80 | 164.00 |
| 926 | 1-Pentadecanol | 29.60 | 47.90 |
| 927 | 2-Methylpropyl 2-cyano-6-methoxy-1(2H)-quinolinecarboxylate | 24.50 | 4.40 |
| 928 | Dodecanediamide | 78.80 | 42.30 |
| 929 | 1,1'-(1,12-Dodecanediyl)bisthymine | 43.95 | 17.90 |
| 930 | Ethyl tridecanoate | 40.70 | 55.80 |
| 931 | 2-Pyridinealdoxime | 19.98 | 1.10 |
| 932 | 2,10-Dimethylacridin-9(10H)-one | 22.40 | 2.60 |
| 933 | Ethyl 2-cyano-6-methoxy-1(2H)-quinolinecarboxylate | 22.40 | 2.60 |
| 934 | Simvastatin | 29.60 | 48.30 |
| 935 | Methyl 4-(2,4-dichlorophenoxy)butyrate | 22.00 | 43.10 |
| 936 | Butyl 2-cyano-1(2H)-quinolinecarboxylate | 22.50 | 2.30 |
| 937 | Azacyclotridecan-2-one | 9.00 | 34.30 |
| 938 | 3-(p-Terphenyl-4-yl)-5-phenylthiophene | 43.00 | 16.40 |
| 939 | Dibenzo-24-crown-8-ether | 62.50 | 71.90 |
| 940 | Isobutyl p-aminobenzoate | 10.70 | 35.70 |
| 941 | 3-(p-Terphenyl-4-yl)-4-phenylthiophene | 42.00 | 15.60 |
| 942 | 7-Heptadecanol | 28.80 | 48.40 |
| 943 | Benzimidazole | 47.80 | 19.60 |
| 944 | Salicaine | 26.80 | 47.10 |
| 945 | Arachic alcohol | 43.60 | 58.90 |
| 946 | Butyl 2-cyano-6-nitro-1(2H)-quinolinecarboxylate | 25.20 | 3.60 |
| 947 | 3-(Octyloxy)-estra-1,3,5(10)-trien-17-one | 19.00 | 41.80 |
| 948 | 2,6-Di-t-butylphenol | 42.00 | 15.00 |
| 949 | Ethyl beta-cyano-3,4-dimethoxy-alph,alpha-dimethyl-beta-(1-methylethyl)benzenepropanoate | 8.80 | 35.10 |
| 950 | Isoquercitrin | 49.80 | 20.30 |
| 951 | Benzo-12-crown-4 | 21.60 | 44.30 |
| 952 | Arcoxia | 30.40 | 6.50 |
| 953 | N-Methyl-(1-(1-(2-fluorophenethyl)piperidin-4-yl)-1H-indol-6-yl)acetamide | 35.20 | 9.80 |
| 954 | 17-Tetratriacontene | 84.93 | 88.80 |
| 955 | 3-(Heptoyloxy)-estra-1,3,5(10)-trien-17-one | 21.00 | 44.40 |
| 956 | 2-Methoxyisonitrosoacetanilide | 27.80 | 4.10 |
| 957 | 3-(Undecanoyloxy)-estra-1,3,5(10)-trien-17-one | 34.00 | 53.60 |
| 958 | 3-(Octoyloxy)-estra-1,3,5(10)-trien-17-one | 24.00 | 46.70 |
| 959 | Fentanyl | 30.10 | 5.60 |
| 960 | Benzo-15-crown-5 | 29.20 | 50.60 |
| 961 | 1,4-Dithiane sulfone | 26.90 | 3.00 |
| 962 | 2-Propenyl 2-cyano-6-nitro-1(2H)-quinolinecarboxylate | 26.90 | 2.70 |
| 963 | Propyl 2-cyano-6-nitro-1(2H)-quinolinecarboxylate | 25.00 | 1.30 |
| 964 | 1,1'-(1,10-Decanediyl)bisthymine | 42.60 | 13.40 |
| 965 | 3-(Decanoyloxy)-estra-1,3,5(10)-trien-17-one | 29.00 | 51.30 |
| 966 | Hexa-2,4-diyne-1,6-diyl-bis(4-hexyloxybenzoate) | 49.30 | 65.50 |
| 967 | Salicin | 55.50 | 22.00 |
| 968 | 4-Butoxyphenyl-(6-(4-butoxyphenyl)aminomethylene-9-methyl-9H-carbazol-3-ylmethylene)-amine | 34.40 | 55.40 |
| 969 | 1,4,5,8-Tetrakis(propoxy)-anthracene | 43.93 | 62.90 |
| 970 | 3-(Nonanoyloxy)-estra-1,3,5(10)-trien-17-one | 24.00 | 49.00 |
| 971 | Felodipine | 31.50 | 54.70 |
| 972 | 10-Methylacridin-9(10H)-one | 29.70 | 2.40 |
| 973 | 10-Ethylacridin-9(10H)-one | 27.50 | 0.30 |
| 974 | 4-Chloro-2'-hydroxy-4'-propoxyazobenzene | 29.80 | 54.40 |
| 975 | 2,4-Dinitrophenylhydrazine | 18.89 | 46.80 |
| 976 | 2-Methylpropyl 2-cyano-6-nitro-1(2H)-quinolinecarboxylate | 28.30 | 0.60 |
| 977 | 2-Chloroisonitrosoacetanilide | 29.70 | 1.40 |
| 978 | 17beta-4-Heptyloxybenzoyloxy testosterone | 22.00 | 49.30 |
| 979 | 3-(Dodecanoyloxy)-estra-1,3,5(10)-trien-17-one | 31.00 | 55.90 |
| 980 | Dibenzo-18-crown-6 | 41.80 | 63.60 |
| 981 | 10-Phenylacridin-9(10H)-one | 38.90 | 7.30 |
| 982 | Maltitol | 55.10 | 18.30 |
| 983 | Glimepiride | 53.30 | 17.00 |
| 984 | 4,7-Dichloro-2-(4-n-tridecafluorohexylphenyl)isoindole-1,3-dione | 38.30 | 6.50 |
| 985 | 4,5,6,7-Tetrachloro-2-(4-n-tridecafluorohexylphenyl)isoindole-1,3-dione | 45.10 | 11.10 |
| 986 | 4-Octylphenyl-(6-(4-Octylphenyl)aminomethylene-9-methyl-9H-carbazol-3-ylmethylene)-amine | 33.10 | 59.50 |
| 987 | 5,6-Dichloro-2-(4-n-tridecafluorohexylphenyl)isoindole-1,3-dione | 40.70 | 6.50 |
| 988 | 10-Methyl-2-nitroacridin-9(10H)-one | 37.60 | 3.60 |
| 989 | 1,4,5,8-Tetrakis(propoxy)-9,10-anthraquinone | 28.60 | 61.00 |
| 990 | Trielaidin | 157.10 | 151.80 |
