# Supplementary material for: Calculation of Five Thermodynamic Molecular Descriptors by Means of a General Computer Algorithm Based on the Group-Additivity Method: Standard Enthalpies of Vaporization, Sublimation and Solvation, and Entropy of Fusion of Ordinary Organic Molecules and Total Phase-Change Entropy of Liquid Crystals
Source: Molecules. 2017 Jun 25;22(7):1059. doi: 10.3390/molecules22071059 (PMC6152037; doi:10.3390/molecules22071059)
Supplement: Supplementary file 1 [file molecules-22-01059-s001.zip › S10. Experimental and Calculated Heat-of-Solvation Data Table.doc]

|  | **Molecules sorted by deviation** |  |  |  |  |
| --- | --- | --- | --- | --- | --- |
| **No.** | **Molecule name** | **ΔH°(solv) exp** | **ΔH°(solv) calc** | **ΔH°(solv) test** | **Deviation** |
|  |  |  |  |  |  |
| 1 | Hexane | -31.90 | -31.90 | -31.90 | 0.00 |
| 2 | Isobutyl propanoate | -54.70 | -54.60 | -54.70 | 0.00 |
| 3 | n-Propylformate | -40.51 | -40.40 | -40.60 | 0.09 |
| 4 | 2-Heptanone | -54.90 | -55.30 | -54.80 | -0.10 |
| 5 | 1-Butylamine | -59.20 | -59.20 | -59.10 | -0.10 |
| 6 | 2,6-Xylidine | -60.50 | -60.60 | -60.60 | 0.10 |
| 7 | Propanone | -39.70 | -39.90 | -39.80 | 0.10 |
| 8 | N-(1-Methylethyl)ethanamide | -83.61 | -84.00 | -83.50 | -0.11 |
| 9 | 3-Aminopyridine | -70.36 | -69.70 | -70.50 | 0.14 |
| 10 | Amyl acetate | -55.34 | -55.30 | -55.50 | 0.16 |
| 11 | 6-Amino-1,3-dimethyluracil | -128.54 | -128.70 |  | 0.16 |
| 12 | 1-Propylamine | -55.98 | -55.30 | -55.80 | -0.18 |
| 13 | Propyl propanoate | -51.18 | -51.50 | -51.00 | -0.18 |
| 14 | Dipropyl sulfide | -47.60 | -47.40 |  | -0.20 |
| 15 | 1,1,2-Trichloroethane | -32.50 | -32.90 | -32.30 | -0.20 |
| 16 | 2,4-Dimethyl-3-pentanone | -54.00 | -53.80 | -53.80 | -0.20 |
| 17 | 1,3-Butyleneglycol | -86.40 | -86.30 | -86.20 | -0.20 |
| 18 | 1-Pentanol | -61.90 | -61.80 | -61.70 | -0.20 |
| 19 | 2,5,8,11-Tetraoxododecane | -102.40 | -101.20 | -102.20 | -0.20 |
| 20 | m-Cresol | -58.70 | -58.80 | -58.50 | -0.20 |
| 21 | 2-Methylpentane | -30.50 | -31.10 | -30.70 | 0.20 |
| 22 | 3-Hydroxybenzaldehyde | -70.70 | -70.80 | -70.90 | 0.20 |
| 23 | 2-Chloroaniline | -55.19 | -55.10 | -54.90 | -0.29 |
| 24 | 2-Ethylpyridine | -55.70 | -54.70 | -55.40 | -0.30 |
| 25 | 1,2,3,4-Tetrachlorobenzene | -35.00 | -34.60 | -34.70 | -0.30 |
| 26 | Pentafluoroethane | -21.50 | -21.20 |  | -0.30 |
| 27 | Methanethiol | -24.40 | -24.10 |  | -0.30 |
| 28 | 5-Nonanone | -62.80 | -63.00 | -63.10 | 0.30 |
| 29 | 1,4-Dimethylbenzene | -34.80 | -35.00 | -35.10 | 0.30 |
| 30 | Di-n-butylamine | -75.90 | -75.90 | -76.20 | 0.30 |
| 31 | Pinacolone | -47.50 | -48.30 | -47.80 | 0.30 |
| 32 | MTBE | -48.68 | -49.70 | -49.00 | 0.32 |
| 33 | Ethylene glycol dibutyrate | -80.47 | -80.70 | -80.80 | 0.33 |
| 34 | Propylurea | -92.75 | -92.40 | -92.40 | -0.35 |
| 35 | Butyric acid methylester | -47.50 | -47.10 | -47.10 | -0.40 |
| 36 | 2-Picoline | -50.30 | -50.90 | -50.70 | 0.40 |
| 37 | Pentane | -28.28 | -29.00 | -28.70 | 0.42 |
| 38 | Urea | -80.27 | -80.90 | -80.70 | 0.43 |
| 39 | Hemimellitene | -37.36 | -38.10 | -37.80 | 0.44 |
| 40 | Ethylurea | -88.97 | -88.50 | -88.50 | -0.47 |
| 41 | 6-Aminouracil | -132.02 | -132.50 |  | 0.48 |
| 42 | 1,3,5-Trichlorobenzene | -34.20 | -33.60 | -33.70 | -0.50 |
| 43 | Diethyl sulphide | -40.20 | -39.70 |  | -0.50 |
| 44 | Ethanol | -50.60 | -50.30 | -50.10 | -0.50 |
| 45 | Isopropylamine | -55.00 | -54.60 | -54.50 | -0.50 |
| 46 | Amyl formate | -48.10 | -48.10 | -48.60 | 0.50 |
| 47 | Acetic acid | -52.80 | -53.30 | -53.30 | 0.50 |
| 48 | Propyl butyrate | -54.90 | -55.30 | -55.40 | 0.50 |
| 49 | Propanenitrile | -39.52 | -39.30 | -39.00 | -0.52 |
| 50 | 1-Octanol | -74.14 | -73.40 | -73.60 | -0.54 |
| 51 | Diisopropyl ether | -51.74 | -52.40 | -52.30 | 0.56 |
| 52 | Ethyl 2-methylbutanoate | -55.40 | -54.60 | -54.80 | -0.60 |
| 53 | Ethanethiol | -28.30 | -27.80 | -27.70 | -0.60 |
| 54 | Isobutylene | -22.70 | -22.90 | -22.10 | -0.60 |
| 55 | Isopentyl formate | -47.70 | -47.30 | -47.10 | -0.60 |
| 56 | Methylvalerate | -50.40 | -51.00 | -51.00 | 0.60 |
| 57 | Ethan-1,2-diol | -77.30 | -78.30 | -77.90 | 0.60 |
| 58 | Cyclohexanone | -49.80 | -50.30 | -50.40 | 0.60 |
| 59 | 2-Methylpropane | -24.18 | -25.30 | -24.80 | 0.62 |
| 60 | 6-Amino-1-methyluracil | -131.25 | -130.60 |  | -0.65 |
| 61 | Propylene | -21.63 | -22.10 | -22.30 | 0.67 |
| 62 | Amylamine | -62.13 | -63.00 | -62.80 | 0.67 |
| 63 | Methylbenzene | -32.40 | -31.90 | -31.70 | -0.70 |
| 64 | Chlorobenzene | -30.60 | -31.40 | -31.30 | 0.70 |
| 65 | Isobutyl formate | -43.00 | -43.50 | -43.70 | 0.70 |
| 66 | Methyl pivalate | -46.20 | -47.90 | -46.90 | 0.70 |
| 67 | Methyl benzoate | -50.25 | -50.90 | -51.00 | 0.75 |
| 68 | Cyanoethylene | -35.02 | -36.10 | -35.80 | 0.78 |
| 69 | N,2-Dimethylpropanamide | -82.91 | -83.60 | -83.70 | 0.79 |
| 70 | 1,1,1-Trifluoro-2-propanol | -53.50 | -52.80 | -52.70 | -0.80 |
| 71 | Methyl formate | -32.70 | -32.20 | -31.90 | -0.80 |
| 72 | Ethyl isobutyrate | -51.30 | -50.70 | -50.50 | -0.80 |
| 73 | Cyclohexane | -30.00 | -30.70 | -30.80 | 0.80 |
| 74 | Nopinene | -37.00 | -37.50 | -37.80 | 0.80 |
| 75 | Dimethyl sulfide | -31.50 | -32.30 |  | 0.80 |
| 76 | Pentanoic acid | -64.10 | -64.90 | -64.90 | 0.80 |
| 77 | 1-Amino-3-methoxypropane | -72.30 | -72.90 | -73.10 | 0.80 |
| 78 | N-Methylpropionamide | -79.75 | -80.50 | -80.60 | 0.85 |
| 79 | Isoamyl acetate | -53.83 | -54.60 | -54.70 | 0.87 |
| 80 | Heptane | -34.01 | -34.80 | -34.90 | 0.89 |
| 81 | 1-Butanethiol | -36.29 | -35.50 | -35.40 | -0.89 |
| 82 | 3-Methyl-2-butanone | -47.60 | -46.80 | -46.70 | -0.90 |
| 83 | 2,5-Xylidine | -61.50 | -60.60 | -60.60 | -0.90 |
| 84 | N-Methylthiourea | -90.10 | -90.80 | -91.00 | 0.90 |
| 85 | Methylcaproate | -54.70 | -54.90 | -55.60 | 0.90 |
| 86 | 2-Octanone | -58.30 | -59.20 | -59.20 | 0.90 |
| 87 | Propanoic acid | -56.50 | -57.20 | -57.40 | 0.90 |
| 88 | 1-Butoxy-2-methoxypropane | -72.49 | -73.60 | -73.40 | 0.91 |
| 89 | Propylacetamide | -85.52 | -84.70 | -84.60 | -0.92 |
| 90 | Cyclohexylamine | -65.32 | -65.00 | -64.40 | -0.92 |
| 91 | o-Toluidine | -56.76 | -57.30 | -57.70 | 0.94 |
| 92 | Anisole | -41.42 | -41.60 | -42.40 | 0.98 |
| 93 | 2,5-Lutidine | -54.90 | -54.20 | -53.90 | -1.00 |
| 94 | Ethyl ether | -45.30 | -44.10 | -44.30 | -1.00 |
| 95 | Ethylcyclohexane | -36.80 | -35.80 | -35.80 | -1.00 |
| 96 | Decane | -42.50 | -43.50 | -43.50 | 1.00 |
| 97 | Butane | -24.80 | -26.10 | -25.80 | 1.00 |
| 98 | Ethyl t-butyl ether | -53.40 | -54.00 | -54.40 | 1.00 |
| 99 | n-Butyl acetate | -52.63 | -51.50 | -51.60 | -1.03 |
| 100 | Ethylacetamide | -80.37 | -80.90 | -81.40 | 1.03 |
| 101 | 3-Cyanophenol | -70.71 | -72.00 | -71.80 | 1.09 |
| 102 | N-Ethylthiourea | -94.40 | -95.00 | -95.50 | 1.10 |
| 103 | cis-1,2-Dichloroethylene | -26.90 | -28.00 |  | 1.10 |
| 104 | Methyl isobutanoate | -46.00 | -46.40 | -47.10 | 1.10 |
| 105 | Benzene | -28.10 | -28.90 | -29.20 | 1.10 |
| 106 | 3-Chloropyridine | -46.20 | -47.20 | -47.30 | 1.10 |
| 107 | Ethyl acetate | -44.96 | -43.70 | -43.80 | -1.16 |
| 108 | Butanal | -43.60 | -41.90 | -42.40 | -1.20 |
| 109 | Ethyl valerate | -56.50 | -55.30 | -55.30 | -1.20 |
| 110 | 2-Pentanol | -63.30 | -62.10 | -62.10 | -1.20 |
| 111 | trans-1,2-Dimethylcyclohexane | -36.10 | -35.00 | -34.90 | -1.20 |
| 112 | 1,2,4-Trimethylbenzene | -36.60 | -38.10 | -37.80 | 1.20 |
| 113 | Fluorene | -42.70 | -43.50 | -43.90 | 1.20 |
| 114 | Butylacetamide | -89.71 | -88.60 | -88.50 | -1.21 |
| 115 | Isopropylacetate | -46.79 | -47.90 | -48.00 | 1.21 |
| 116 | 2-Methoxyethyl acetate | -62.65 | -61.30 | -61.40 | -1.25 |
| 117 | Ethyl butanoate | -52.70 | -51.50 | -51.40 | -1.30 |
| 118 | Ethyl isovalerate | -56.00 | -54.60 | -54.70 | -1.30 |
| 119 | Methyl propanoate | -44.50 | -43.30 | -43.20 | -1.30 |
| 120 | Caprylene | -39.20 | -40.20 | -40.50 | 1.30 |
| 121 | trans-1,2-Dichloroethylene | -29.30 | -28.00 |  | -1.30 |
| 122 | 3-Methyl-2-butanol | -59.90 | -61.30 | -61.20 | 1.30 |
| 123 | s-Butylamine | -57.10 | -58.50 | -58.40 | 1.30 |
| 124 | Methyl acetate | -38.10 | -39.40 | -39.40 | 1.30 |
| 125 | 1,3,5-Trimethylbenzene | -39.12 | -38.10 | -37.80 | -1.32 |
| 126 | N,N'-Dimethylurea | -88.25 | -87.70 | -86.90 | -1.35 |
| 127 | Pentanenitrile | -45.64 | -47.00 | -47.00 | 1.36 |
| 128 | Pyrrole | -42.63 | -42.10 | -44.00 | 1.37 |
| 129 | Ethyl hexanoate | -60.20 | -59.20 | -58.80 | -1.40 |
| 130 | Propan-1,3-diol | -81.10 | -82.20 | -82.50 | 1.40 |
| 131 | 2,3-Dichlorobiphenyl | -45.60 | -47.30 | -47.00 | 1.40 |
| 132 | 4-Pyridinamine | -70.90 | -69.70 | -69.50 | -1.40 |
| 133 | 1,1,2,2-Tetrachloroethane | -34.80 | -35.40 | -36.20 | 1.40 |
| 134 | N,N'-Diethylurea | -94.70 | -96.10 | -96.10 | 1.40 |
| 135 | Trimethylethylene | -26.61 | -26.30 | -25.20 | -1.41 |
| 136 | n-Hexanoic acid | -67.28 | -68.70 | -68.70 | 1.42 |
| 137 | n-Hexylamine | -65.93 | -66.90 | -67.40 | 1.47 |
| 138 | Glutaric acid | -94.71 | -96.00 | -96.20 | 1.49 |
| 139 | n-Propyl acetate | -49.40 | -47.60 | -47.90 | -1.50 |
| 140 | 1,1-Dichloroethane | -30.30 | -28.80 |  | -1.50 |
| 141 | 1,2,3-Trichlorobenzene | -32.60 | -33.60 | -34.10 | 1.50 |
| 142 | 1-Nonanol | -75.90 | -77.30 | -77.40 | 1.50 |
| 143 | 1-Methylpyrrole | -39.66 | -40.20 | -41.20 | 1.54 |
| 144 | Methylurea | -85.67 | -84.30 | -84.10 | -1.57 |
| 145 | 2-Methylpropanal | -40.00 | -41.20 | -41.60 | 1.60 |
| 146 | 1-Hexanol | -67.40 | -65.70 | -65.80 | -1.60 |
| 147 | 3,5-Lutidine | -51.30 | -52.70 | -52.90 | 1.60 |
| 148 | Glyceryl tributanoate | -112.70 | -114.10 | -114.30 | 1.60 |
| 149 | Ethanediol diacetate | -67.06 | -65.30 | -65.40 | -1.66 |
| 150 | 1,2-Dihydroxybenzene | -81.97 | -80.50 | -80.30 | -1.67 |
| 151 | 1,3-Diaminopropane | -85.60 | -84.60 | -83.90 | -1.70 |
| 152 | Propanal | -39.40 | -38.10 | -37.70 | -1.70 |
| 153 | Glyme | -59.30 | -57.40 | -57.60 | -1.70 |
| 154 | n-Hexylacetate | -60.80 | -59.20 | -59.10 | -1.70 |
| 155 | Butanoic acid | -59.50 | -61.00 | -61.20 | 1.70 |
| 156 | 2,5-Dichlorobiphenyl | -45.60 | -47.30 | -47.30 | 1.70 |
| 157 | 1,3-Dimethyl-5-propyluracil | -101.10 | -102.60 | -102.80 | 1.70 |
| 158 | Benzylamine | -64.46 | -62.90 | -62.70 | -1.76 |
| 159 | Tetrahydropyran | -48.87 | -46.80 | -47.10 | -1.77 |
| 160 | 4-Chlorotoluene | -33.30 | -34.80 | -35.10 | 1.80 |
| 161 | Ethyl propanoate | -49.52 | -47.60 | -47.70 | -1.82 |
| 162 | Dipropyl ether | -49.88 | -51.80 | -51.70 | 1.82 |
| 163 | Dimethylether | -33.97 | -35.50 | -35.80 | 1.83 |
| 164 | 1-Butene | -24.14 | -25.70 | -26.00 | 1.86 |
| 165 | Isobutyl acetate | -49.02 | -50.70 | -50.90 | 1.88 |
| 166 | 2-Ethylaniline | -59.70 | -61.20 | -61.60 | 1.90 |
| 167 | Enanthic acid | -70.96 | -72.60 | -72.90 | 1.94 |
| 168 | n-Butylurea | -94.71 | -96.20 | -96.70 | 1.99 |
| 169 | 2-Heptanol | -72.60 | -69.80 | -70.60 | -2.00 |
| 170 | cis-1,2-Cyclohexanediamine | -94.10 | -93.50 | -92.10 | -2.00 |
| 171 | Phenethyl alcohol | -67.40 | -65.60 | -65.40 | -2.00 |
| 172 | Methyl t-pentyl ether | -55.20 | -53.50 | -53.20 | -2.00 |
| 173 | 2,3-Dimethylbutane | -32.40 | -30.30 | -30.40 | -2.00 |
| 174 | Octane | -36.00 | -37.70 | -38.00 | 2.00 |
| 175 | 1-Propanethiol | -30.20 | -31.70 | -32.20 | 2.00 |
| 176 | 2-Butanone | -41.90 | -43.70 | -43.90 | 2.00 |
| 177 | Hexylbenzene | -52.72 | -50.00 | -50.70 | -2.02 |
| 178 | Diethylketone | -49.45 | -47.60 | -47.40 | -2.05 |
| 179 | Benzonitrile | -48.50 | -46.90 | -46.40 | -2.10 |
| 180 | 1,1-Difluoroethane | -20.70 | -22.80 |  | 2.10 |
| 181 | 3-Pentanol | -59.60 | -62.10 | -61.70 | 2.10 |
| 182 | Isobutylurea | -93.95 | -95.50 | -96.10 | 2.15 |
| 183 | Methyl-n-propylketone | -45.31 | -47.60 | -47.50 | 2.19 |
| 184 | 2-Methylnaphthalene | -44.90 | -43.10 | -42.70 | -2.20 |
| 185 | n-Valeraldehyde | -47.80 | -45.80 | -45.60 | -2.20 |
| 186 | alpha-Pinene | -36.90 | -37.30 | -39.10 | 2.20 |
| 187 | 2-Phenylethylamine | -68.81 | -66.80 | -66.60 | -2.21 |
| 188 | 4-Hydroxybenzonitrile | -70.29 | -72.00 | -72.50 | 2.21 |
| 189 | Methylamine | -45.27 | -47.20 | -47.50 | 2.23 |
| 190 | 1-Methylnaphthalene | -45.00 | -43.10 | -42.70 | -2.30 |
| 191 | Acetophenone | -53.30 | -51.30 | -51.00 | -2.30 |
| 192 | o-Xylene | -37.70 | -35.00 | -35.40 | -2.30 |
| 193 | Cyclopentanol | -58.50 | -60.90 | -60.80 | 2.30 |
| 194 | t-Butylacetamide | -80.50 | -82.80 |  | 2.30 |
| 195 | Butyl propanoate | -57.80 | -55.30 | -55.40 | -2.40 |
| 196 | 2-Nonanone | -65.30 | -63.00 | -62.90 | -2.40 |
| 197 | 3-Chlorotoluene | -37.00 | -34.80 | -34.60 | -2.40 |
| 198 | Cyclooctane | -39.00 | -36.50 | -36.60 | -2.40 |
| 199 | 2,4-Xylidine | -58.70 | -60.60 | -61.10 | 2.40 |
| 200 | Acetonitrile | -33.20 | -35.40 | -35.60 | 2.40 |
| 201 | Ethyl pivalate | -50.30 | -52.20 | -52.70 | 2.40 |
| 202 | 1-Hexene | -30.40 | -32.90 | -32.80 | 2.40 |
| 203 | n-Octanoic acid | -74.27 | -76.50 | -76.70 | 2.43 |
| 204 | Ethylamine | -53.68 | -51.50 | -51.20 | -2.48 |
| 205 | 3-Nitrophenol | -67.70 | -65.60 | -65.20 | -2.50 |
| 206 | Phenol | -57.70 | -55.40 | -55.20 | -2.50 |
| 207 | Isobutyronitrile | -40.00 | -42.40 | -42.50 | 2.50 |
| 208 | Ethane | -17.90 | -20.30 | -20.40 | 2.50 |
| 209 | 3,4-Lutidine | -50.50 | -52.70 | -53.10 | 2.60 |
| 210 | Glyceryltriacetate | -89.30 | -90.90 | -91.90 | 2.60 |
| 211 | Cyclopentane | -30.33 | -27.80 | -27.70 | -2.63 |
| 212 | p-Cresol | -61.30 | -58.80 | -58.60 | -2.70 |
| 213 | Isobutyl isobutyrate | -55.30 | -57.60 | -58.00 | 2.70 |
| 214 | 1-Heptanol | -72.13 | -69.60 | -69.40 | -2.73 |
| 215 | 1,3-Dichlorobenzene | -35.30 | -32.50 | -32.50 | -2.80 |
| 216 | 4-Nitrophenol | -68.60 | -65.60 | -65.80 | -2.80 |
| 217 | 1,2-Dipropoxyethane | -76.80 | -73.80 | -73.90 | -2.90 |
| 218 | 2,2-Dimethylpropane | -23.40 | -25.90 | -26.30 | 2.90 |
| 219 | Propane | -20.40 | -23.20 | -23.30 | 2.90 |
| 220 | 2,5-Dimethylhexane | -32.70 | -36.20 | -35.60 | 2.90 |
| 221 | Trimethylamine | -52.71 | -50.40 | -49.80 | -2.91 |
| 222 | Piperidine | -65.41 | -63.20 | -62.50 | -2.91 |
| 223 | 2-Methyl-1-propanol | -60.16 | -57.20 | -57.20 | -2.96 |
| 224 | 1,4-Diaminobutane | -91.60 | -88.40 | -88.60 | -3.00 |
| 225 | Succinonitrile | -58.20 | -56.40 | -55.20 | -3.00 |
| 226 | N,N-Dimethylaniline | -45.70 | -48.40 | -48.70 | 3.00 |
| 227 | 1,2-Butanediol | -83.20 | -86.30 | -86.20 | 3.00 |
| 228 | Cyclopentanone | -44.30 | -46.40 | -47.30 | 3.00 |
| 229 | Ethylene glycol dipropionate | -75.21 | -73.00 | -72.20 | -3.01 |
| 230 | n-Heptaldehyde | -56.40 | -53.50 | -53.30 | -3.10 |
| 231 | Nitrobenzene | -43.80 | -40.50 | -40.70 | -3.10 |
| 232 | 2,5,8,11,14-Pentaoxopentadecane | -125.80 | -123.20 | -122.70 | -3.10 |
| 233 | 2-Hexanone | -48.90 | -51.40 | -52.00 | 3.10 |
| 234 | Methylacetamide | -73.71 | -76.70 | -76.90 | 3.19 |
| 235 | Naphthalene | -42.80 | -40.00 | -39.60 | -3.20 |
| 236 | 2-Chlorobiphenyl | -42.80 | -46.20 | -46.00 | 3.20 |
| 237 | Malonic acid | -83.97 | -88.20 | -87.20 | 3.23 |
| 238 | 2-Pyrrolidone | -82.65 | -79.70 | -79.40 | -3.25 |
| 239 | 4-Heptanone | -58.10 | -55.30 | -54.80 | -3.30 |
| 240 | Aniline | -56.50 | -54.00 | -53.20 | -3.30 |
| 241 | 2-Methyl-2-propanol | -62.90 | -60.10 | -59.60 | -3.30 |
| 242 | t-Butylurea | -87.20 | -90.50 |  | 3.30 |
| 243 | Isooctane | -31.00 | -33.80 | -34.30 | 3.30 |
| 244 | 1,5-Diaminopentane | -95.50 | -92.30 | -92.10 | -3.40 |
| 245 | N,N'-Diethylthiourea | -106.30 | -102.60 | -102.90 | -3.40 |
| 246 | Hexafluoro-2-propanol | -57.10 | -51.20 | -53.70 | -3.40 |
| 247 | 4-Ethylaniline | -65.00 | -61.20 | -61.60 | -3.40 |
| 248 | Isopropyl formate | -43.00 | -40.60 | -39.60 | -3.40 |
| 249 | 2-Nitrotoluene | -46.40 | -43.80 | -43.00 | -3.40 |
| 250 | Ethyl formate | -33.60 | -36.50 | -37.00 | 3.40 |
| 251 | Isocumene | -36.40 | -39.20 | -39.80 | 3.40 |
| 252 | 2-Hexanol | -69.24 | -66.00 | -65.80 | -3.44 |
| 253 | 3-Hexanol | -69.58 | -66.00 | -66.10 | -3.48 |
| 254 | 1-Chloropropane | -27.00 | -30.10 | -30.50 | 3.50 |
| 255 | 2,4-Lutidine | -51.10 | -54.20 | -54.60 | 3.50 |
| 256 | 2,4'-Dichlorobiphenyl | -44.20 | -47.30 | -47.70 | 3.50 |
| 257 | N-Methylmorpholine | -68.70 | -71.80 | -72.20 | 3.50 |
| 258 | Acetamide | -70.66 | -73.20 | -74.20 | 3.54 |
| 259 | Adiponitrile | -66.60 | -64.10 | -63.00 | -3.60 |
| 260 | 1-Butanol | -61.71 | -58.00 | -58.10 | -3.61 |
| 261 | Amylbenzene | -49.45 | -46.40 | -45.80 | -3.65 |
| 262 | Ethylenedichloride | -32.75 | -30.40 | -29.10 | -3.65 |
| 263 | 2-Chlorotoluene | -38.30 | -34.80 | -34.60 | -3.70 |
| 264 | 1-Chloropentane | -34.10 | -37.90 | -37.80 | 3.70 |
| 265 | Di-n-propylamine | -65.20 | -68.20 | -68.90 | 3.70 |
| 266 | n-Butylethylether | -48.43 | -51.80 | -52.20 | 3.77 |
| 267 | cis-1,2-Dimethylcyclohexane | -38.30 | -35.00 | -34.50 | -3.80 |
| 268 | Chloroethane | -22.00 | -26.30 | -25.80 | 3.80 |
| 269 | 2,5-Dioxaoctane | -69.10 | -65.60 | -65.30 | -3.80 |
| 270 | Styrene | -28.40 | -32.30 | -32.20 | 3.80 |
| 271 | Methyl methacrylate | -44.15 | -41.10 | -40.30 | -3.85 |
| 272 | 1,4-Dichlorobutane | -41.16 | -38.10 | -37.30 | -3.86 |
| 273 | 1,3-Dimethylbenzene | -38.60 | -35.00 | -34.70 | -3.90 |
| 274 | 2,3,4-Trimethylpentane | -38.50 | -35.40 | -34.60 | -3.90 |
| 275 | 3-Ethylpyridine | -49.10 | -53.30 | -53.00 | 3.90 |
| 276 | Di-n-butylether | -55.80 | -59.60 | -59.70 | 3.90 |
| 277 | 1,3-Dichloropropane | -37.38 | -34.20 | -33.40 | -3.98 |
| 278 | 2,5-Dioxaheptane | -66.10 | -61.70 | -62.10 | -4.00 |
| 279 | Benzaldehyde | -42.10 | -45.70 | -46.10 | 4.00 |
| 280 | 1,5-Dichloropentane | -44.78 | -41.90 | -40.70 | -4.08 |
| 281 | N,N-Diethylformamide | -68.29 | -65.30 | -64.20 | -4.09 |
| 282 | Dimethylamine | -53.09 | -52.10 | -49.00 | -4.09 |
| 283 | Pentanedinitrile | -63.50 | -60.20 | -59.40 | -4.10 |
| 284 | 1,4-Diethylbenzene | -46.40 | -42.20 | -42.30 | -4.10 |
| 285 | Propanamide | -73.40 | -77.10 | -77.50 | 4.10 |
| 286 | 2-Methyltetrahydrofuran | -42.60 | -47.10 | -46.70 | 4.10 |
| 287 | 4-Ethylpyridine | -50.20 | -53.30 | -54.30 | 4.10 |
| 288 | Uracil | -101.31 | -97.70 | -97.20 | -4.11 |
| 289 | N-Methylpiperidine | -65.77 | -61.50 | -61.60 | -4.17 |
| 290 | Tetramethylene glycol | -89.60 | -86.00 | -85.40 | -4.20 |
| 291 | 2-Propanol | -58.44 | -54.40 | -54.20 | -4.24 |
| 292 | 2-Ethoxyethylacetate | -68.67 | -65.70 | -64.40 | -4.27 |
| 293 | Butyl butanoate | -63.50 | -59.20 | -59.20 | -4.30 |
| 294 | s-Butylacetate | -47.99 | -51.70 | -52.30 | 4.31 |
| 295 | Butanedioic acid | -94.39 | -92.10 | -90.00 | -4.39 |
| 296 | 5-Ethyl-1,3-dimethyluracil | -101.30 | -98.70 | -96.90 | -4.40 |
| 297 | N,N'-Dimethylthiourea | -97.80 | -94.20 | -93.40 | -4.40 |
| 298 | Ethylbenzene | -39.40 | -35.50 | -35.00 | -4.40 |
| 299 | 4-t-Butylphenol | -63.80 | -67.20 | -68.20 | 4.40 |
| 300 | 1,4-Dichlorobenzene | -28.40 | -32.50 | -32.80 | 4.40 |
| 301 | Tetrahydrofuran | -47.32 | -43.00 | -42.80 | -4.52 |
| 302 | 1,4-Dihydroxybenzene | -84.22 | -80.50 | -79.70 | -4.52 |
| 303 | 2,4-Dichlorobiphenyl | -43.00 | -47.30 | -47.60 | 4.60 |
| 304 | 2-Butanol | -62.72 | -58.20 | -58.10 | -4.62 |
| 305 | 4-Heptanol | -75.30 | -69.80 | -70.60 | -4.70 |
| 306 | 1,2-Diaminoethane | -76.10 | -80.70 | -80.80 | 4.70 |
| 307 | 15-Crown-5 | -119.28 | -122.90 | -124.00 | 4.72 |
| 308 | Butylbenzene | -38.50 | -42.80 | -43.30 | 4.80 |
| 309 | 1,2-Dichlorobenzene | -37.30 | -32.50 | -32.40 | -4.90 |
| 310 | Diethylamine | -64.30 | -60.50 | -59.30 | -5.00 |
| 311 | 2,2,2-Trifluoroethanol | -53.10 | -48.70 | -48.10 | -5.00 |
| 312 | Morpholine | -69.50 | -73.50 | -74.50 | 5.00 |
| 313 | 2-Methoxyphenol | -62.60 | -66.70 | -67.70 | 5.10 |
| 314 | 4-Methyl-2-pentanol | -69.90 | -65.20 | -64.70 | -5.20 |
| 315 | Isopentyl alcohol | -65.80 | -61.10 | -60.60 | -5.20 |
| 316 | Hexamethyleneglycol | -98.70 | -93.70 | -93.50 | -5.20 |
| 317 | Pyridine | -42.10 | -46.10 | -47.30 | 5.20 |
| 318 | Cumene | -33.70 | -38.40 | -38.90 | 5.20 |
| 319 | Butanamide | -76.63 | -81.00 | -82.00 | 5.37 |
| 320 | 1,6-Hexanedioic acid | -95.40 | -99.80 | -100.80 | 5.40 |
| 321 | 3-Methylpyridine | -54.28 | -49.40 | -48.80 | -5.48 |
| 322 | 2-Methoxyethylamine | -63.51 | -69.10 | -69.10 | 5.59 |
| 323 | t-Butylamine | -59.00 | -53.40 |  | -5.60 |
| 324 | 2-Methyl-2-butanol | -68.44 | -64.00 | -62.80 | -5.64 |
| 325 | 4-Methylpyridine | -54.78 | -49.40 | -49.10 | -5.68 |
| 326 | Thiourea | -89.30 | -87.40 | -83.60 | -5.70 |
| 327 | Diethylacetamide | -78.19 | -76.10 | -72.40 | -5.79 |
| 328 | 3-Hexanone | -46.00 | -51.40 | -51.80 | 5.80 |
| 329 | Methylpropylether | -38.00 | -43.70 | -43.80 | 5.80 |
| 330 | Benzyl alcohol | -66.94 | -61.70 | -61.10 | -5.84 |
| 331 | Biphenyl | -47.20 | -42.20 | -41.30 | -5.90 |
| 332 | Pentachlorobenzene | -39.90 | -35.70 | -34.00 | -5.90 |
| 333 | 1-Propanol | -59.90 | -54.10 | -53.90 | -6.00 |
| 334 | Cyclohexene | -27.30 | -31.60 | -33.30 | 6.00 |
| 335 | 2-Ethoxyethanol | -66.40 | -72.20 | -72.40 | 6.00 |
| 336 | Methanol | -52.00 | -45.90 | -45.90 | -6.10 |
| 337 | 2,5-Dimethyltetrahydrofuran | -56.30 | -51.20 | -50.20 | -6.10 |
| 338 | 3-Methylpentane | -36.80 | -31.10 | -30.70 | -6.10 |
| 339 | 1-Chlorobutane | -28.20 | -34.00 | -34.40 | 6.20 |
| 340 | Cyclohexanol | -70.70 | -64.80 | -64.50 | -6.20 |
| 341 | Caproic aldehyde | -55.20 | -49.70 | -49.00 | -6.20 |
| 342 | o-Cresol | -64.80 | -58.80 | -58.50 | -6.30 |
| 343 | Cycloheptanol | -74.60 | -68.70 | -68.30 | -6.30 |
| 344 | Formamide | -58.07 | -62.50 | -64.40 | 6.33 |
| 345 | 3-Nitrotoluene | -38.50 | -43.80 | -45.00 | 6.50 |
| 346 | cis- Cyclooctene | -34.50 | -38.80 | -41.00 | 6.50 |
| 347 | 1,5-Pentanediol | -97.39 | -89.90 | -90.80 | -6.59 |
| 348 | 3-Chlorophenol | -50.30 | -56.50 | -57.00 | 6.70 |
| 349 | Dimethylacetamide | -71.65 | -67.60 | -64.80 | -6.85 |
| 350 | Methyl isobutyl ketone | -44.60 | -50.70 | -51.50 | 6.90 |
| 351 | Cyclopropane | -15.40 | -22.00 | -22.30 | 6.90 |
| 352 | Ethene | -13.70 | -18.70 | -20.70 | 7.00 |
| 353 | Butylcellosolve | -73.60 | -79.90 | -80.80 | 7.20 |
| 354 | 2-Propoxyethanol | -69.60 | -76.00 | -76.90 | 7.30 |
| 355 | N,N-Dimethylurea | -80.88 | -75.30 | -73.50 | -7.38 |
| 356 | 12-Crown-4 | -94.65 | -101.00 | -102.10 | 7.45 |
| 357 | 1-Chlorohexane | -34.50 | -41.70 | -42.00 | 7.50 |
| 358 | p-Cymene | -34.60 | -41.50 | -42.10 | 7.50 |
| 359 | Diethyl cellosolve | -71.90 | -66.00 | -64.30 | -7.60 |
| 360 | Butyronitrile | -36.20 | -43.10 | -43.80 | 7.60 |
| 361 | Acenaphthene | -52.10 | -45.00 | -44.30 | -7.80 |
| 362 | 1-Phenyl-1-propanone | -61.90 | -55.20 | -54.10 | -7.80 |
| 363 | 1,2,3-Propanetriol | -103.50 | -110.50 | -111.30 | 7.80 |
| 364 | Methyl cellosolve | -60.40 | -67.90 | -68.40 | 8.00 |
| 365 | 1-Dodecanol | -81.90 | -88.90 | -90.10 | 8.20 |
| 366 | N-Methylformamide | -61.38 | -65.90 | -69.60 | 8.22 |
| 367 | 1,2-Propanediol | -74.96 | -82.40 | -83.30 | 8.34 |
| 368 | Colamine | -71.36 | -79.50 | -79.90 | 8.54 |
| 369 | N,N-Dimethylformamide | -62.90 | -56.90 | -54.30 | -8.60 |
| 370 | 3,5,5-Trimethyl-2-cyclohexenone | -59.10 | -52.50 | -50.40 | -8.70 |
| 371 | 2,6-Lutidine | -60.48 | -55.60 | -51.50 | -8.98 |
| 372 | Triethylamine | -69.60 | -63.00 | -60.60 | -9.00 |
| 373 | 2,3-Lutidine | -46.20 | -54.20 | -55.20 | 9.00 |
| 374 | t-Butylacetate | -46.22 | -53.60 | -55.40 | 9.18 |
| 375 | 1,4-Dioxane | -48.40 | -57.20 | -57.60 | 9.20 |
| 376 | 1,4,7,10,13,16-Hexaoxacyclooctadecane | -149.51 | -144.80 | -140.30 | -9.21 |
| 377 | Caprylic aldehyde | -48.80 | -57.40 | -58.60 | 9.80 |
| 378 | Pivalamide | -89.00 | -81.70 | -78.70 | -10.30 |
| 379 | Diethyleneglycol diethyl ether | -96.20 | -88.00 | -85.50 | -10.70 |
| 380 | 4-Dimethylaminopyridine | -72.86 | -64.10 | -60.60 | -12.26 |
| 381 | cis-1,2-Cyclohexanediol | -82.40 | -93.10 | -94.90 | 12.50 |
| 382 | N,N-Dimethylethanolamine | -72.51 | -82.60 | -85.80 | 13.29 |
| 383 | s-trans-1,3-Butadiene | -31.40 | -22.50 | -18.00 | -13.40 |
| 384 | Anthracene | -44.30 | -51.10 | -58.10 | 13.80 |
| 385 | Tetramethylthiourea | -68.30 | -76.10 | -83.50 | 15.20 |
| 386 | N,N-Diethylaniline | -45.70 | -56.80 | -61.50 | 15.80 |
| 387 | 2-Nitrophenol | -49.80 | -65.60 | -65.80 | 16.00 |
| 388 | Trifluorothymine | -87.23 | -97.10 | -104.50 | 17.27 |
