# Supplementary material for: Calculation of Five Thermodynamic Molecular Descriptors by Means of a General Computer Algorithm Based on the Group-Additivity Method: Standard Enthalpies of Vaporization, Sublimation and Solvation, and Entropy of Fusion of Ordinary Organic Molecules and Total Phase-Change Entropy of Liquid Crystals
Source: Molecules. 2017 Jun 25;22(7):1059. doi: 10.3390/molecules22071059 (PMC6152037; doi:10.3390/molecules22071059)
Supplement: Supplementary file 1 [file molecules-22-01059-s001.zip › S12. Experimental and Calculated Entropy-of-Fusion Data Table.doc]

|  | **Molecules sorted by deviation** |  |  |  |  |
| --- | --- | --- | --- | --- | --- |
| **No.** | **Molecule name** | **ΔS(fus) exp** | **ΔS(fus) calc** | **ΔS(fus) test** | **Deviation** |
|  |  |  |  |  |  |
| 1 | 2-Chlorodibenzofuran | 52.00 | 52.30 | 52.00 | 0.00 |
| 2 | 3-Chlorodibenzofuran | 52.00 | 52.30 | 52.00 | 0.00 |
| 3 | Tris(pentafluoroethyl)amine | 29.80 | 29.80 |  | 0.00 |
| 4 | Ethyl naltrexone-3-O-carbonate | 46.80 | 50.90 | 46.80 | 0.00 |
| 5 | Decyl methacrylate | 121.90 | 122.90 | 121.90 | 0.00 |
| 6 | Pentacosofluorooctacosane | 163.80 | 162.30 | 163.80 | 0.00 |
| 7 | 2-Butanone | 45.59 | 45.00 | 45.60 | -0.01 |
| 8 | 2,5-Dinitrophenol | 62.28 | 62.80 | 62.30 | -0.02 |
| 9 | Dibenzo[a,i]pyrene | 50.05 | 51.10 | 50.10 | -0.05 |
| 10 | 1,2,3,4-Tetra(methoxycarbonyl)naphthalene | 84.47 | 85.40 | 84.40 | 0.07 |
| 11 | Ethyl propyl sulfide | 67.78 | 66.80 | 67.70 | 0.08 |
| 12 | Octanoylamide Deferoxamine | 287.10 | 288.70 | 287.20 | -0.10 |
| 13 | 4-Methoxy-N-(3-methoxyphenylmethylaminothioxomethyl)benzamide | 79.70 | 80.50 | 79.60 | 0.10 |
| 14 | 3-Fluorotoluene | 45.00 | 45.00 | 44.90 | 0.10 |
| 15 | N-2-(4-Picolyl)-N'-(4-chlorophenyl) thiourea | 76.50 | 73.60 | 76.40 | 0.10 |
| 16 | 3-Benzoyl-1,2,2-trimethylcyclopentanecarboxylic acid | 52.50 | 53.30 | 52.60 | -0.10 |
| 17 | 4-Bromophenol | 52.00 | 52.30 | 52.10 | -0.10 |
| 18 | 4-Chloro-2-nitrophenol | 63.00 | 62.70 | 63.10 | -0.10 |
| 19 | Naringenin | 76.10 | 75.20 | 76.20 | -0.10 |
| 20 | trans-Decalin | 51.10 | 51.50 | 51.20 | -0.10 |
| 21 | 4-Ethynyl-1-(4-ethynylphenylmethoxy)benzene | 57.11 | 60.20 | 57.00 | 0.11 |
| 22 | Triphenylene | 52.53 | 52.40 | 52.40 | 0.13 |
| 23 | Perfluidone | 75.97 | 76.60 | 76.10 | -0.13 |
| 24 | Butyl pentanoate | 92.94 | 91.70 | 92.80 | 0.14 |
| 25 | Guajen | 42.06 | 42.00 | 42.20 | -0.14 |
| 26 | Pyrolan | 65.96 | 66.00 | 66.10 | -0.14 |
| 27 | 1,5-Bis(methoxycarbonyl)naphthalene | 67.35 | 66.90 | 67.20 | 0.15 |
| 28 | Piperidine | 56.64 | 58.00 | 56.80 | -0.16 |
| 29 | Ethofumesate | 76.28 | 76.10 |  | 0.18 |
| 30 | 1,4-Dioxane | 53.98 | 53.30 | 53.80 | 0.18 |
| 31 | 2-Cyclohexyl-4,6-dinitrophenol | 74.02 | 74.00 | 74.20 | -0.18 |
| 32 | Nonyl urea | 102.30 | 101.70 | 102.10 | 0.20 |
| 33 | Methanol | 44.10 | 43.50 | 43.90 | 0.20 |
| 34 | Suberone | 61.80 | 61.40 | 61.60 | 0.20 |
| 35 | 4-Trifluoromethyl-7-(diethylamino)coumarin | 64.70 | 63.90 | 64.50 | 0.20 |
| 36 | Decaethylcyclopentasilane | 67.20 | 62.80 | 67.00 | 0.20 |
| 37 | Vinyl acetate | 46.80 | 46.40 | 47.00 | -0.20 |
| 38 | 1-Aminoanthraquinone | 54.90 | 55.70 | 55.10 | -0.20 |
| 39 | Ethyltrichlorosilane | 42.09 | 46.20 | 42.30 | -0.21 |
| 40 | 6,9-Dimethyl-8-ethyladenine | 68.22 | 68.00 |  | 0.22 |
| 41 | Perfluoropropane | 39.58 | 37.80 | 39.80 | -0.22 |
| 42 | Dinoseb | 69.54 | 70.50 | 69.80 | -0.26 |
| 43 | 4-Chlorophenylacetic acid | 62.37 | 62.00 | 62.10 | 0.27 |
| 44 | 2-(2-Chloro-3-methylphenoxy)propanoic acid | 61.68 | 62.50 | 61.40 | 0.28 |
| 45 | trans,trans-2,6-Octadiene-1,8-dioic acid | 76.50 | 76.00 | 76.20 | 0.30 |
| 46 | Hemellitic acid | 43.80 | 44.00 | 43.50 | 0.30 |
| 47 | Codeine | 55.30 | 53.80 | 55.00 | 0.30 |
| 48 | Xylitol | 102.30 | 101.00 | 102.00 | 0.30 |
| 49 | 1,2,3,5-Tetra(methoxycarbonyl)benzene | 83.89 | 83.50 | 84.20 | -0.31 |
| 50 | 2-Phenoxypropionic acid | 62.93 | 62.70 | 62.60 | 0.33 |
| 51 | Metobromuron | 66.36 | 66.00 |  | 0.36 |
| 52 | (1E,2E)-Bis(4-methoxybenzylidene)hydrazine | 67.30 | 66.90 | 66.90 | 0.40 |
| 53 | Dodecyl urea | 127.60 | 127.10 | 127.20 | 0.40 |
| 54 | 4-(Trifluoromethyl)-7-(N,N-dimethylamino)coumarin | 62.40 | 61.60 | 62.00 | 0.40 |
| 55 | Pentacosafluoroeicosane | 97.60 | 94.60 | 98.00 | -0.40 |
| 56 | 1,3-Dihydroxybenzene | 53.80 | 53.80 | 54.20 | -0.40 |
| 57 | Acenaphthylene | 42.40 | 43.10 | 42.80 | -0.40 |
| 58 | 3,4,5-Trimethoxyphenol | 76.00 | 77.00 | 76.40 | -0.40 |
| 59 | Diphenylacetamide | 62.50 | 62.80 | 62.90 | -0.40 |
| 60 | 4-Methylheptane | 71.22 | 71.30 | 70.80 | 0.42 |
| 61 | Methyl-n-propylketone | 54.14 | 53.40 | 53.70 | 0.44 |
| 62 | Ethylbenzene | 51.43 | 51.70 | 51.90 | -0.47 |
| 63 | N-2-(6-Picolyl)-N'-(4-chlorophenyl) thiourea | 86.40 | 83.10 | 85.90 | 0.50 |
| 64 | 4,4'-Thiodiphenol | 71.70 | 73.20 | 71.20 | 0.50 |
| 65 | 4-Acetylanisole | 54.10 | 53.80 | 53.60 | 0.50 |
| 66 | 1,2,3,4-Tetrachlorodibenzo-p-dioxin | 68.00 | 69.30 | 68.50 | -0.50 |
| 67 | 2,8-Dichlorodibenzofuran | 55.00 | 55.50 | 55.50 | -0.50 |
| 68 | 2-Fluorotoluene | 46.00 | 45.00 | 45.50 | 0.50 |
| 69 | Isobutyl t-butylether | 53.30 | 55.70 | 53.80 | -0.50 |
| 70 | 2-Chloro-2-methylpropane | 45.02 | 46.10 | 44.50 | 0.52 |
| 71 | 1-Piperazinyl-3,5-bis(dimethylamino)-s-triazine | 60.24 | 61.10 | 59.70 | 0.54 |
| 72 | 2,2,3,3-Tetramethylpentane | 50.85 | 50.50 | 51.40 | -0.55 |
| 73 | 8-Methylquinoline | 43.46 | 43.00 | 42.90 | 0.56 |
| 74 | 1-Butanethiol | 66.43 | 66.40 | 67.00 | -0.57 |
| 75 | 2,3,6-TBA | 59.23 | 59.80 | 59.80 | -0.57 |
| 76 | 4-Ethoxybenzoic acid | 62.18 | 61.40 | 61.60 | 0.58 |
| 77 | o-Chloromandelic acid | 62.89 | 62.20 | 62.30 | 0.59 |
| 78 | trans-1,2-Cyclohexanediol | 54.89 | 55.70 | 54.30 | 0.59 |
| 79 | m-Fluoromandelic acid | 61.59 | 60.80 | 61.00 | 0.59 |
| 80 | 3-Nitrophenol | 53.90 | 53.30 | 53.30 | 0.60 |
| 81 | 1,1,1-Trifluoroethane | 38.30 | 36.40 | 37.70 | 0.60 |
| 82 | Ethyne | 37.30 | 36.80 | 36.70 | 0.60 |
| 83 | 1-(4-Nitrophenylethynyl)-4-propylbenzene | 66.20 | 67.40 | 66.80 | -0.60 |
| 84 | Acridone | 50.80 | 52.10 | 51.40 | -0.60 |
| 85 | Testosterone valerate | 81.45 | 80.40 | 80.80 | 0.65 |
| 86 | Isopentane | 45.23 | 45.90 | 45.90 | -0.67 |
| 87 | 1-Bromo-2-chloroethane | 54.68 | 53.60 | 54.00 | 0.68 |
| 88 | Ethyl dodecanoate | 132.02 | 134.00 | 132.70 | -0.68 |
| 89 | Griseofulvin | 79.60 | 79.40 | 78.90 | 0.70 |
| 90 | 2,4,6-Triazido-s-triazine | 60.50 | 59.80 |  | 0.70 |
| 91 | Monomethyl phthalate | 60.50 | 59.70 | 59.80 | 0.70 |
| 92 | 3-Methyl-4-hydroxybenzaldehyde | 63.00 | 62.30 | 62.30 | 0.70 |
| 93 | TME | 76.90 | 76.70 | 76.20 | 0.70 |
| 94 | Butyl 4-hydroxybenzoate | 77.80 | 78.30 | 78.50 | -0.70 |
| 95 | 3-(4-Ethylbenzoyl)-1,2,2-trimethylcyclopentanecarboxylic acid | 58.20 | 57.90 | 57.50 | 0.70 |
| 96 | Pyrazine | 49.20 | 46.70 | 48.50 | 0.70 |
| 97 | 2-Cyclohexylcyclohexanone | 65.00 | 65.90 | 65.70 | -0.70 |
| 98 | 4-Nitrobenzonitrile | 46.10 | 47.20 | 46.80 | -0.70 |
| 99 | Butyl butanoate | 82.20 | 83.20 | 82.90 | -0.70 |
| 100 | N,N’-Bis(salicylaldehydo)ethylenediimine | 85.70 | 85.10 | 86.40 | -0.70 |
| 101 | 8-Propyltheophylline | 62.32 | 62.10 | 61.60 | 0.72 |
| 102 | 3-(3-Chlorophenyl)-3-hydroxypropanoic acid | 76.18 | 77.00 | 76.90 | -0.72 |
| 103 | 1-Chlorodibenzo-p-dioxin | 61.34 | 60.00 | 60.60 | 0.74 |
| 104 | Tetrahydroanthracene | 58.86 | 58.80 | 58.10 | 0.76 |
| 105 | 4-(cis-3-Hexenyloxy)-3',4'-difluorodiphenyldiacetylene | 84.94 | 85.70 | 85.70 | -0.76 |
| 106 | 1,1,1,3,5,5,5-Heptamethyl-3-phenyltrisiloxane | 80.70 | 80.00 | 79.90 | 0.80 |
| 107 | N-(2-Trimethylsilylethyl)ethylenimine | 60.20 | 60.80 | 59.40 | 0.80 |
| 108 | 1,2-Dimethylhydrazine | 51.60 | 50.80 |  | 0.80 |
| 109 | 1,2-Dimethoxytetrafluoroethane | 42.70 | 43.50 |  | -0.80 |
| 110 | trans-Cinnamic acid | 54.70 | 55.40 | 55.50 | -0.80 |
| 111 | N-Methyl-N-(4-chlorophenyl)nitramine | 60.30 | 60.80 | 61.10 | -0.80 |
| 112 | 1-Phenyl-1-methylsilacyclobutane | 58.50 | 58.80 | 59.30 | -0.80 |
| 113 | N-Propionylpyrazinecarboxamide | 64.09 | 67.30 | 64.90 | -0.81 |
| 114 | 2-Methylnonane | 87.97 | 88.20 | 88.80 | -0.83 |
| 115 | Dibenzo[a,l]pyrene | 49.24 | 51.10 | 50.10 | -0.86 |
| 116 | DDE | 65.33 | 63.50 | 66.20 | -0.87 |
| 117 | 1-Pentadecanoic acid | 152.97 | 152.10 | 152.10 | 0.87 |
| 118 | 2,3-Dimethyl-2,3-dinitrobutane | 67.98 | 67.30 | 67.10 | 0.88 |
| 119 | Triamcinolone diacetate | 75.42 | 82.20 | 76.30 | -0.88 |
| 120 | Spiro[2.2]pentane | 38.72 | 41.50 | 39.60 | -0.88 |
| 121 | 1,3,5,7-Tetrachloronaphthalene | 59.91 | 60.90 | 60.80 | -0.89 |
| 122 | 1,6-Anhydro-beta-D-altropyranose | 52.80 | 52.60 | 51.90 | 0.90 |
| 123 | 2,2,11,11-Tetramethyl-1,3,10,12-tetraoxacyclooctadecane | 94.10 | 93.10 | 93.20 | 0.90 |
| 124 | Methylurea | 42.10 | 41.30 | 41.20 | 0.90 |
| 125 | p-Dioxanone | 53.50 | 52.30 | 52.60 | 0.90 |
| 126 | 1,2,3-Trichlorobenzene | 56.90 | 55.90 | 56.00 | 0.90 |
| 127 | 1,2-Dihydro-1,6-dimethylphenanthro[1,2-b]furan-10,11-dione | 45.30 | 46.70 | 46.20 | -0.90 |
| 128 | Perfluorotoluene | 55.32 | 53.10 | 54.40 | 0.92 |
| 129 | 2-Bromoaniline | 53.08 | 53.60 | 54.00 | -0.92 |
| 130 | Tridecanoic acid | 135.52 | 135.10 | 134.60 | 0.92 |
| 131 | 4-Ethoxy-4'-fluorodiphenylacetylene | 64.33 | 63.20 | 63.40 | 0.93 |
| 132 | 5-Methylnonane | 89.13 | 88.20 | 88.20 | 0.93 |
| 133 | 1,3,5-Trimethyladamantane | 34.43 | 34.50 | 33.50 | 0.93 |
| 134 | Phenylcarbamate | 52.97 | 52.00 | 53.90 | -0.93 |
| 135 | 2-Methylthiophene | 45.57 | 44.80 | 44.60 | 0.97 |
| 136 | Pentacene | 55.22 | 54.30 | 56.20 | -0.98 |
| 137 | Pycene | 55.22 | 54.30 | 56.20 | -0.98 |
| 138 | 2,3-Dinitrophenol | 62.92 | 62.80 | 63.90 | -0.98 |
| 139 | Allobarbital | 73.00 | 70.90 | 72.00 | 1.00 |
| 140 | Tetradecanedinitrile | 136.50 | 138.00 | 137.50 | -1.00 |
| 141 | 4-Nitrophenol | 52.30 | 53.30 | 53.30 | -1.00 |
| 142 | 2-Methylfuran | 47.01 | 45.90 | 46.00 | 1.01 |
| 143 | Pentafluoroaniline | 60.22 | 59.80 | 59.20 | 1.02 |
| 144 | 1,2-Difluorobenzene | 48.87 | 49.90 | 49.90 | -1.03 |
| 145 | Thioxanthene | 64.96 | 64.10 | 63.90 | 1.06 |
| 146 | 3-(Diphenylmethyl)-3-propyl-2,4-pentanedione | 77.61 | 77.80 | 78.70 | -1.09 |
| 147 | 1-Octanol | 97.70 | 96.30 | 96.60 | 1.10 |
| 148 | Ethyl 2-cyano-1(2H)-quinolinecarboxylate | 59.00 | 58.10 | 57.90 | 1.10 |
| 149 | Saprorthoquinone | 62.50 | 61.70 | 61.40 | 1.10 |
| 150 | 4-Acetoxybenzoic acid | 56.40 | 55.20 | 55.30 | 1.10 |
| 151 | 2-Methylpropyl 2-cyano-6-methoxy-1(2H)-quinolinecarboxylate | 70.60 | 69.60 | 69.50 | 1.10 |
| 152 | 4-(4-Propylcyclohexyl)benzonitrile | 67.90 | 66.30 | 66.80 | 1.10 |
| 153 | Methyl N-(4,6-dimethoxypyrimidin-2-yl)carbamate | 73.60 | 73.70 | 74.70 | -1.10 |
| 154 | N-Methyl-N-(4-methoxyphenyl)nitramine | 66.30 | 66.70 | 67.40 | -1.10 |
| 155 | 2,5-Diheptyloxy-1,4-benzoquinone | 154.00 | 156.30 | 155.10 | -1.10 |
| 156 | Ancymidol | 69.51 | 68.40 |  | 1.11 |
| 157 | 2-Methyl-1-phenyl-2-(N-piperidinyl)-1-propanone | 53.97 | 58.20 | 55.10 | -1.13 |
| 158 | Devrinol | 71.16 | 72.60 | 72.30 | -1.14 |
| 159 | 1-Methyltetrazole | 49.85 | 51.60 | 48.70 | 1.15 |
| 160 | Stearic acid | 178.80 | 177.50 | 177.60 | 1.20 |
| 161 | 3-(3-Chloro-4-methoxybenzoyl)-1,2,2-trimethylcyclopentanecarboxylic acid | 66.10 | 65.20 | 64.90 | 1.20 |
| 162 | trans-1,2-Dimethylcyclopentane | 41.60 | 40.10 | 40.40 | 1.20 |
| 163 | 2,4,6-Tribromophenol | 56.90 | 56.70 | 55.70 | 1.20 |
| 164 | 2-Thiophenecarboxylic acid | 52.40 | 52.00 | 51.20 | 1.20 |
| 165 | 3-Aminophenol | 55.30 | 55.10 | 54.10 | 1.20 |
| 166 | 2-Ethoxyisonitrosoacetanilide | 56.80 | 59.00 | 58.00 | -1.20 |
| 167 | Chrysanthemic acid | 37.10 | 40.00 | 38.30 | -1.20 |
| 168 | 3-Tetradecylamino-1,2-propanediol | 182.20 | 183.70 | 183.40 | -1.20 |
| 169 | 1,9-Nonanediol | 113.89 | 115.40 | 115.10 | -1.21 |
| 170 | Testosterone propionate | 65.24 | 63.50 | 64.00 | 1.24 |
| 171 | Thioanisole | 57.85 | 58.60 | 59.10 | -1.25 |
| 172 | Nitrofen | 67.13 | 68.80 | 68.40 | -1.27 |
| 173 | N-Methylcarbazole | 47.32 | 48.70 | 48.60 | -1.28 |
| 174 | o-Cresol | 48.50 | 46.90 | 47.20 | 1.30 |
| 175 | 1,2,4,5-Tetrachlorobenzene | 60.40 | 59.00 | 59.10 | 1.30 |
| 176 | 4-Aminobenzenesulfonamide | 58.70 | 60.50 | 60.00 | -1.30 |
| 177 | 12-Crown-4 | 77.30 | 75.60 | 76.00 | 1.30 |
| 178 | (2-Hydroxyethyl)(3-(hydroxyiminomethyl)phenyl) carbamic acid | 56.90 | 56.10 | 58.20 | -1.30 |
| 179 | 1,5-Diaminopentane | 104.60 | 105.00 | 105.90 | -1.30 |
| 180 | Octyl methacrylate | 104.60 | 105.90 | 105.90 | -1.30 |
| 181 | Pentane | 58.59 | 60.30 | 59.90 | -1.31 |
| 182 | cis-Cyclohexane-1,2-dicarboxylic anhydride | 48.42 | 48.30 | 47.10 | 1.32 |
| 183 | Fenson | 65.53 | 64.20 |  | 1.33 |
| 184 | 2-Naphthoic acid | 51.15 | 52.40 | 52.50 | -1.35 |
| 185 | 1,1'-Diphenyl-1,1'-bicyclopentyl | 75.77 | 74.70 | 74.40 | 1.37 |
| 186 | 3-Octylamino-1,2-propanediol | 134.27 | 133.00 | 132.90 | 1.37 |
| 187 | 1,2-Pentadiene | 55.63 | 57.00 |  | -1.37 |
| 188 | Methyl propyl sulfide | 61.88 | 61.10 | 60.50 | 1.38 |
| 189 | 4-Iodotoluene | 48.79 | 47.00 | 47.40 | 1.39 |
| 190 | 4-Bromoiodobenzene | 54.00 | 52.40 | 52.60 | 1.40 |
| 191 | Methyl carbamate | 51.00 | 48.10 | 49.60 | 1.40 |
| 192 | 6-Methyl-1,2,3,4-tetrahydro-1-oxocarbazole | 57.40 | 55.60 | 56.00 | 1.40 |
| 193 | Xanthene | 55.20 | 57.60 | 56.60 | -1.40 |
| 194 | Nitrofungin | 54.90 | 56.40 | 56.30 | -1.40 |
| 195 | 1,1,1-Tris(4-cyanatophenyl)ethane | 75.00 | 75.60 | 76.40 | -1.40 |
| 196 | Butane | 53.62 | 51.80 | 52.20 | 1.42 |
| 197 | 1,2,4,5-Tetrafluorobenzene | 54.33 | 53.20 | 52.90 | 1.43 |
| 198 | Hexachlorocyclopropane | 49.47 | 50.40 | 50.90 | -1.43 |
| 199 | 1,1-Dimethylhydrazine | 46.64 | 45.20 |  | 1.44 |
| 200 | 3-Carboxyphenol | 55.15 | 54.10 | 53.70 | 1.45 |
| 201 | alpha-Methylstyrene | 47.55 | 49.10 | 49.00 | -1.45 |
| 202 | 4-Methylnonane | 86.94 | 88.20 | 88.40 | -1.46 |
| 203 | 2,3-Dihydro-2,2-dimethyl-7-benzofuranol-3-one | 49.47 | 45.40 | 48.00 | 1.47 |
| 204 | Tritane | 60.13 | 62.60 | 61.60 | -1.47 |
| 205 | Caprylic aldehyde | 89.73 | 92.10 | 91.20 | -1.47 |
| 206 | 2-Chloro-6-(trichloromethyl)pyridine | 60.19 | 60.00 | 58.70 | 1.49 |
| 207 | Proxyphylline | 60.51 | 59.60 | 62.00 | -1.49 |
| 208 | 4-(2,4-Dichlorophenoxy)butyric acid | 88.50 | 87.80 | 87.00 | 1.50 |
| 209 | 2-Deoxy-d-glucose | 68.00 | 66.00 | 66.50 | 1.50 |
| 210 | 1,2,4,5-Tetrabromobenzene | 53.70 | 55.20 | 55.20 | -1.50 |
| 211 | N,N,N',N'-Tetraethyl-1,2-benzenedicarbothiamide | 60.20 | 63.30 | 61.70 | -1.50 |
| 212 | 3-(3,4-Diethoxybenzoyl)-1,2,2-trimethylcyclopentanecarboxylic acid | 74.70 | 75.00 | 76.20 | -1.50 |
| 213 | 1,2-Dibromobenzene | 49.40 | 50.90 | 50.90 | -1.50 |
| 214 | 4-Butyl-3',4'-difluorodiphenylacetylene | 78.21 | 76.10 | 76.70 | 1.51 |
| 215 | 1,3,5-Trichlorotrifluorobenzene | 59.19 | 60.90 | 60.70 | -1.51 |
| 216 | 1,4-Diisocyanoatobutane | 89.79 | 91.30 |  | -1.51 |
| 217 | Trifluoroacetonitrile | 38.62 | 37.20 | 37.10 | 1.52 |
| 218 | 1,1-Dichloro-2,2-bis(4-ethylphenyl)ethane | 70.38 | 71.70 | 71.90 | -1.52 |
| 219 | 3-Chloro-1,1,1-trifluoropropane | 54.64 | 53.60 | 53.10 | 1.54 |
| 220 | Chlorobenzilate | 75.64 | 76.90 | 77.20 | -1.56 |
| 221 | 1,8-Octanediol | 108.47 | 106.90 | 106.90 | 1.57 |
| 222 | Phenoxathiin | 61.63 | 63.40 | 63.20 | -1.57 |
| 223 | Methyloxiran | 40.52 | 41.90 | 42.10 | -1.58 |
| 224 | Carbazole | 52.19 | 50.50 | 50.60 | 1.59 |
| 225 | cis-1,2-Cyclohexanediol | 54.31 | 55.70 | 55.90 | -1.59 |
| 226 | 4-Chloro-2'-hydroxy-4'-methoxyazobenzene | 86.40 | 84.00 | 84.80 | 1.60 |
| 227 | 1,2,4-Trichlorodibenzo-p-dioxin | 67.00 | 66.20 | 65.40 | 1.60 |
| 228 | Ethylenedichloride | 53.00 | 52.50 | 51.40 | 1.60 |
| 229 | 1,4-Dibromobenzene | 52.00 | 50.90 | 50.40 | 1.60 |
| 230 | 6-Hydroxymethyl-5,6-dihydro-7H-dibenzo[a,c]cycloheptane | 63.80 | 62.60 | 62.20 | 1.60 |
| 231 | 1,4-Diiodobenzene | 55.60 | 54.00 | 54.00 | 1.60 |
| 232 | 2-t-Butyl-4-methoxymethyl-6-alpha-methylbenzylphenol | 79.10 | 77.30 | 77.50 | 1.60 |
| 233 | 3,4-Dichloronitrobenzene | 57.10 | 55.90 | 55.50 | 1.60 |
| 234 | 4-Methoxybenzoic acid | 61.10 | 59.40 | 59.50 | 1.60 |
| 235 | Chloropentafluorobenzene | 55.50 | 58.00 | 57.10 | -1.60 |
| 236 | Benzimidazole | 47.80 | 48.00 | 49.40 | -1.60 |
| 237 | 2,2,9,9-Tetramethyl-1,3,8,10-tetraoxacyclotetradecane | 74.50 | 77.00 | 76.10 | -1.60 |
| 238 | 1,11-Cycloeicosanedione bis(ethylene ketal) | 120.71 | 117.80 | 119.10 | 1.61 |
| 239 | Methoxychlor | 76.21 | 71.10 | 74.60 | 1.61 |
| 240 | 3-Chlorobenzoic acid | 55.32 | 53.60 | 53.70 | 1.62 |
| 241 | Tridecyl urea | 134.47 | 135.50 | 136.10 | -1.63 |
| 242 | Tetrahydrofuran | 51.83 | 50.30 | 50.20 | 1.63 |
| 243 | Cyanoethylene | 40.16 | 41.70 | 41.80 | -1.64 |
| 244 | Ovex | 65.64 | 67.30 |  | -1.66 |
| 245 | N-Octylleucine | 94.88 | 93.40 | 93.20 | 1.68 |
| 246 | N-Propylstearamide | 187.40 | 184.40 | 185.70 | 1.70 |
| 247 | Fentanyl | 84.00 | 81.70 | 82.30 | 1.70 |
| 248 | Benzophenone | 55.00 | 53.20 | 53.30 | 1.70 |
| 249 | Fluorene | 51.30 | 49.90 | 49.60 | 1.70 |
| 250 | Arcoxia | 74.80 | 76.10 | 76.50 | -1.70 |
| 251 | N-Acetyl-L-alanine amide | 50.30 | 52.30 | 52.00 | -1.70 |
| 252 | Trichloroethylene | 44.80 | 46.50 |  | -1.70 |
| 253 | Decanedioic acid | 115.50 | 116.90 | 117.20 | -1.70 |
| 254 | Methylcyclopentane | 43.60 | 45.40 | 45.30 | -1.70 |
| 255 | Piperonyl alcohol | 55.20 | 56.60 | 56.90 | -1.70 |
| 256 | 4-Acetylphenol | 47.20 | 48.50 | 48.90 | -1.70 |
| 257 | 4-Fluorotoluene | 43.19 | 45.00 | 44.90 | -1.71 |
| 258 | Dibenzo-p-dioxin | 58.63 | 56.90 | 56.90 | 1.73 |
| 259 | Benzyl methyl sulfone | 63.73 | 60.90 | 62.00 | 1.73 |
| 260 | Methyl 4-aminobenzoate | 58.56 | 60.70 | 60.30 | -1.74 |
| 261 | Benzonitrile | 42.16 | 44.00 | 43.90 | -1.74 |
| 262 | Cyclopentyl methyl sulfide | 59.75 | 56.10 | 58.00 | 1.75 |
| 263 | Vinylidenechloride | 43.26 | 41.90 | 41.50 | 1.76 |
| 264 | 1-(4-Nitrophenylethynyl)-4-ethoxybenzene | 67.00 | 64.70 | 65.20 | 1.80 |
| 265 | Methyl 4-hydroxybenzoate | 61.00 | 59.40 | 59.20 | 1.80 |
| 266 | Pipemidic acid | 62.00 | 57.80 | 60.20 | 1.80 |
| 267 | 3-Thiopheneacetic acid | 53.20 | 56.20 | 55.00 | -1.80 |
| 268 | N-(3-Methoxyphenylmethyl)-N'-dodecylthiourea | 146.20 | 147.50 | 148.00 | -1.80 |
| 269 | 4-Aminophenol | 52.30 | 55.10 | 54.10 | -1.80 |
| 270 | Bicyclo[3.3.1]nonan-9-one | 47.00 | 49.40 | 48.80 | -1.80 |
| 271 | epsilon-Caprolactam | 47.00 | 48.50 | 48.80 | -1.80 |
| 272 | 1,4,6-Tris(methoxycarbonyl)naphthalene | 73.60 | 76.20 | 75.40 | -1.80 |
| 273 | 2-Methylheptane | 72.62 | 71.30 | 70.80 | 1.82 |
| 274 | Tebufelone | 73.47 | 74.90 | 75.30 | -1.83 |
| 275 | Pyrazole | 41.43 | 36.40 | 39.60 | 1.83 |
| 276 | 3-Ethylpentane | 61.77 | 62.80 | 63.60 | -1.83 |
| 277 | p-Tolyl vinyl sulfone | 31.96 | 34.50 | 33.80 | -1.84 |
| 278 | Azulene | 48.15 | 46.50 | 46.30 | 1.85 |
| 279 | 3-Octylthio-1,2-propanediol | 129.85 | 128.40 | 128.00 | 1.85 |
| 280 | 3-Methyl-1,2-butadiene | 49.87 | 48.20 | 48.00 | 1.87 |
| 281 | Chloroneb | 68.23 | 70.70 | 70.10 | -1.87 |
| 282 | DDD | 71.48 | 67.50 | 69.60 | 1.88 |
| 283 | N-(4-Methyl-2-nitrophenyl)acetamide | 58.79 | 57.10 | 56.90 | 1.89 |
| 284 | Ethyl(1,1-dimethylpropyl)malononitrile | 62.59 | 60.50 | 60.70 | 1.89 |
| 285 | 2,6-Diisopropylphenol | 50.00 | 48.80 | 48.10 | 1.90 |
| 286 | 2,3-Dichlorodibenzo-p-dioxin | 61.00 | 63.10 | 62.90 | -1.90 |
| 287 | 4-Amino-3-methylbenzoic acid | 49.50 | 52.10 | 51.40 | -1.90 |
| 288 | 3-Decylamino-1,2-propanediol | 158.11 | 156.20 | 156.20 | 1.91 |
| 289 | Heptane | 78.81 | 77.20 | 76.90 | 1.91 |
| 290 | 2-Methylthiazole | 48.91 | 42.80 | 47.00 | 1.91 |
| 291 | N-Methyl-(1-(1-(2-fluorophenethyl)piperidin-4-yl)-1H-indol-6-yl)acetamide | 85.23 | 82.60 | 83.30 | 1.93 |
| 292 | 2,6-Di-t-butylphenol | 53.33 | 50.80 | 51.40 | 1.93 |
| 293 | 1,4,7,10,13,16,19,22-Octaoxacyclotetracosane | 118.07 | 120.00 | 120.00 | -1.93 |
| 294 | Metoxuron | 68.86 | 71.10 | 70.80 | -1.94 |
| 295 | Phosmet | 78.56 | 79.70 | 80.50 | -1.94 |
| 296 | Diethyl phthalate | 66.63 | 69.00 | 68.60 | -1.97 |
| 297 | 4-Chlorotoluene | 48.29 | 46.40 | 46.30 | 1.99 |
| 298 | 1,3,5-Tribromobenzene | 55.00 | 53.00 | 53.00 | 2.00 |
| 299 | 1-Thiaindan | 55.00 | 56.90 | 57.00 | -2.00 |
| 300 | 2-Nitrobenzonitrile | 45.70 | 47.20 | 47.70 | -2.00 |
| 301 | 4-Dimethylaminopyridine | 55.90 | 57.80 | 57.90 | -2.00 |
| 302 | 1,3-Dibromopropane | 61.40 | 63.20 | 63.40 | -2.00 |
| 303 | 3-Oxabicyclo[3.2.2]nonane | 48.71 | 46.40 | 46.70 | 2.01 |
| 304 | Diphenyl ether | 57.38 | 59.40 | 59.40 | -2.02 |
| 305 | 2-Methylpropane | 39.73 | 37.50 | 37.70 | 2.03 |
| 306 | Azidothymidine | 83.45 | 85.50 |  | -2.05 |
| 307 | Trimethylvinylsilane | 54.06 | 52.00 |  | 2.06 |
| 308 | 2-Trichlorosilylbiphenyl | 61.28 | 57.50 | 59.20 | 2.08 |
| 309 | Oryzalin | 92.78 | 92.20 | 90.70 | 2.08 |
| 310 | 2,2',3,3',4,4'-Hexachlorobiphenyl | 68.62 | 70.30 | 70.70 | -2.08 |
| 311 | Phenyl vinyl sulfone | 34.12 | 37.70 | 36.20 | -2.08 |
| 312 | 2-(4-Nitrophenyl)-1-(4-propoxyphenyl)ethanone | 85.90 | 84.20 | 83.80 | 2.10 |
| 313 | 3,3,7,7,11,11,15,15-Octamethylcyclohexadecane-1,9-dione | 81.00 | 80.10 | 78.90 | 2.10 |
| 314 | Tetrazole | 42.70 | 40.80 | 40.60 | 2.10 |
| 315 | Ethynylbenzene | 41.50 | 43.70 | 43.60 | -2.10 |
| 316 | 1,2,3-Propanetriol | 62.80 | 64.80 | 64.90 | -2.10 |
| 317 | 4-Propylbenzoic acid | 66.51 | 64.10 | 64.40 | 2.11 |
| 318 | Norbornene | 43.66 | 43.90 | 45.80 | -2.14 |
| 319 | 1,2,6,7-Tetra(methoxycarbonyl)naphthalene | 83.76 | 85.40 | 85.90 | -2.14 |
| 320 | Theophylline | 51.84 | 51.90 | 54.00 | -2.16 |
| 321 | 2-(1,3-Dioxolan-2-yl)phenyl methylcarbamate | 61.51 | 61.60 | 63.70 | -2.19 |
| 322 | trans-4-Octadecenoic acid | 167.80 | 165.50 | 165.60 | 2.20 |
| 323 | Deferoxamine | 256.10 | 254.70 | 253.90 | 2.20 |
| 324 | 1,2-Dichlorobenzene | 50.50 | 52.80 | 52.70 | -2.20 |
| 325 | Naphthalene | 46.50 | 48.50 | 48.70 | -2.20 |
| 326 | 1,3-Diaminopropane | 86.90 | 88.10 | 89.10 | -2.20 |
| 327 | Diphenylmethane | 62.34 | 60.10 | 60.10 | 2.24 |
| 328 | 1,2,4-Trichlorobenzene | 53.56 | 55.90 | 55.80 | -2.24 |
| 329 | Folbex | 70.53 | 73.00 | 72.80 | -2.27 |
| 330 | Oxycarboxin | 66.39 | 63.20 | 64.10 | 2.29 |
| 331 | 2-(4-Nitrophenyl)-1-(4-(4-propylcyclohexyl)phenyl)ethanone | 89.10 | 87.10 | 86.80 | 2.30 |
| 332 | 1,4-Diaminobutane | 95.10 | 96.50 | 92.80 | 2.30 |
| 333 | 2,3-Bis(trifluoromethyl)-4-methoxyphenyl-alpha,alpha-difluoro-4-propylcyclohexyl ether | 68.00 | 70.30 |  | -2.30 |
| 334 | 4-Hydroxybenzaldehyde | 55.40 | 53.30 | 53.10 | 2.30 |
| 335 | Methyl 3-((hydroxyimino)(4-methoxy-3-nitrophenyl)methyl)-1,2,2-trimethylcyclopentanecarboxylate | 73.90 | 75.90 | 76.20 | -2.30 |
| 336 | 1,3-Dichlorobenzene | 50.60 | 52.80 | 52.90 | -2.30 |
| 337 | 1,6-Anhydro-beta-D-glucopyranose | 64.80 | 65.20 | 67.10 | -2.30 |
| 338 | 4-Methyl-7-(dimethylamino)coumarin | 57.47 | 60.20 | 59.80 | -2.33 |
| 339 | Methyl benzoate | 53.26 | 55.80 | 55.60 | -2.34 |
| 340 | Propylcyclopentane | 64.45 | 62.40 | 62.10 | 2.35 |
| 341 | Chloroacetic acid | 48.74 | 50.90 | 51.10 | -2.36 |
| 342 | 2,3-Dinitrotoluene | 53.27 | 49.60 | 50.90 | 2.37 |
| 343 | 1,1-Dichloroethane | 44.67 | 42.00 | 42.30 | 2.37 |
| 344 | o-Fluoromandelic acid | 57.63 | 60.80 | 60.00 | -2.37 |
| 345 | Cyclohexanethiol | 52.73 | 56.40 | 55.10 | -2.37 |
| 346 | 2-Methylpentane | 52.43 | 54.40 | 54.80 | -2.37 |
| 347 | 2-Amino-5-nitropyridine | 63.29 | 62.30 | 60.90 | 2.39 |
| 348 | 1,7-Heptanediamine | 123.80 | 121.90 | 121.40 | 2.40 |
| 349 | 1,3-Dinitrobenzene | 54.60 | 52.80 | 52.20 | 2.40 |
| 350 | Triphenylmethyl chloride | 74.10 | 71.20 | 71.70 | 2.40 |
| 351 | N-(2,3-Dichlorophenyl)benzenesulfonamide | 70.30 | 73.60 | 72.70 | -2.40 |
| 352 | 2,7-Difluorofluorene | 51.90 | 53.30 | 54.30 | -2.40 |
| 353 | endo-2-Methylnorbornane | 36.72 | 34.70 | 34.30 | 2.42 |
| 354 | N-Hexadecyl-L-valine | 149.48 | 152.60 | 151.90 | -2.42 |
| 355 | n-Butylmethylsulfide | 71.03 | 69.50 | 68.60 | 2.43 |
| 356 | Cythioate | 76.13 | 75.70 | 73.70 | 2.43 |
| 357 | Benzo[a]fluorene | 49.26 | 51.90 | 51.70 | -2.44 |
| 358 | Ethylnitrate | 47.74 | 49.90 | 50.20 | -2.46 |
| 359 | 3-Thiophenecarboxylic acid | 44.32 | 47.80 | 46.80 | -2.48 |
| 360 | Nalidixic acid | 71.60 | 70.50 | 69.10 | 2.50 |
| 361 | 1,4-Dichlorobenzene | 55.30 | 52.80 | 52.80 | 2.50 |
| 362 | 2,2,4,4-Tetramethyl-1,3-dioxan | 43.50 | 43.90 | 46.00 | -2.50 |
| 363 | Dodecanamide | 127.50 | 126.10 | 125.00 | 2.50 |
| 364 | 5-Nitro-8-hydroxyquinoline | 54.50 | 53.10 | 52.00 | 2.50 |
| 365 | 1,2,3,5-Tetrachlorobenzene | 56.60 | 59.00 | 59.10 | -2.50 |
| 366 | 2,7-Dibromofluorene | 51.80 | 54.30 | 54.30 | -2.50 |
| 367 | 2,4,4-Trimethyl-2-pentene | 40.90 | 43.10 | 43.40 | -2.50 |
| 368 | 18-Methylpentatriacontane | 304.40 | 308.20 | 306.90 | -2.50 |
| 369 | 2-Chloro-alpha-(4-chlorophenyl)-alpha-(trichloromethyl)benzenemethanol | 63.61 | 61.40 | 61.10 | 2.51 |
| 370 | Butylcyclopentane | 68.49 | 71.40 | 71.00 | -2.51 |
| 371 | 4-Methylpyridine | 45.43 | 43.30 | 42.90 | 2.53 |
| 372 | 4,4-Dimethyl-1,8-diphenyl-2,3,5-trioxabicyclo[4.3.0]non-7-ene | 59.86 | 63.70 | 62.40 | -2.54 |
| 373 | 4-Hexyloxy-3',4'-difluorodiphenylacetylene | 102.29 | 98.70 | 99.70 | 2.59 |
| 374 | 2,7-Bis(methoxycarbonyl)naphthalene | 64.31 | 66.90 | 66.90 | -2.59 |
| 375 | Cycloundecanone | 80.50 | 77.50 | 77.90 | 2.60 |
| 376 | Monuron | 65.50 | 62.10 | 62.90 | 2.60 |
| 377 | 4-Bromo-4'-(3-butenyloxy)biphenyl | 80.80 | 79.30 | 78.20 | 2.60 |
| 378 | p-Quinquephenyl | 64.10 | 67.00 | 66.70 | -2.60 |
| 379 | Isoquinoline | 45.20 | 48.50 | 47.80 | -2.60 |
| 380 | 1,2,4,5,6,8-Hexachloronaphthalene | 64.30 | 67.10 | 66.90 | -2.60 |
| 381 | Silybin | 105.80 | 111.00 | 108.40 | -2.60 |
| 382 | Dexamethasone acetate | 75.00 | 78.90 | 77.60 | -2.60 |
| 383 | 2,3-Pentadiene | 44.80 | 47.40 |  | -2.60 |
| 384 | N,N'-Bis(3-methoxyphenyl)urea | 83.01 | 79.90 | 80.40 | 2.61 |
| 385 | 2-Hydroxybenzophenone | 59.88 | 63.20 | 62.50 | -2.62 |
| 386 | Pendimethalin | 76.92 | 72.40 | 74.30 | 2.62 |
| 387 | Formamide | 31.45 | 28.80 |  | 2.65 |
| 388 | Phosalone | 93.86 | 90.60 | 91.20 | 2.66 |
| 389 | Chlorbromuron | 71.79 | 69.10 |  | 2.69 |
| 390 | 2,3,4,5,6-Pentafluorotoluene | 54.49 | 51.60 | 51.80 | 2.69 |
| 391 | 1-Nitronaphthalene | 54.39 | 51.60 | 51.70 | 2.69 |
| 392 | Propyl 4-hydroxybenzoate | 72.30 | 69.90 | 69.60 | 2.70 |
| 393 | 2,6-Dichloro-4-nitroaniline | 63.10 | 60.80 | 60.40 | 2.70 |
| 394 | 1,3-Dibromobenzene | 53.40 | 50.90 | 50.70 | 2.70 |
| 395 | 1,4-Diaminobenzene | 59.80 | 56.40 | 57.10 | 2.70 |
| 396 | 2-Biphenyl methanol | 60.80 | 59.00 | 58.10 | 2.70 |
| 397 | Cyclopentanone | 51.30 | 53.50 | 54.00 | -2.70 |
| 398 | 1,2,7,8-tetrahydroxyoctane | 104.20 | 107.30 | 106.90 | -2.70 |
| 399 | Chrysin | 70.20 | 72.10 | 72.90 | -2.70 |
| 400 | 3-Chlorophenylacetic acid | 64.61 | 62.00 | 61.90 | 2.71 |
| 401 | 1,10-Decanediol | 120.69 | 123.80 | 123.40 | -2.71 |
| 402 | Methyl 4-dimethylaminobenzoate | 70.12 | 67.00 | 67.40 | 2.72 |
| 403 | 1,1-Dimethylcyclopentane | 49.52 | 47.00 | 46.80 | 2.72 |
| 404 | 2-Amino-3-methylbenzoic acid | 61.02 | 58.50 | 58.30 | 2.72 |
| 405 | DMSO | 49.26 | 46.50 |  | 2.76 |
| 406 | Tetrachloroethylene | 43.38 | 41.80 | 40.60 | 2.78 |
| 407 | 4-Methyl-4-cyclohexene-1,2-dicarboxylic anhydride | 52.70 | 49.30 | 49.90 | 2.80 |
| 408 | Anisole | 58.10 | 55.50 | 55.30 | 2.80 |
| 409 | s-Triazine | 41.20 | 44.00 |  | -2.80 |
| 410 | Biphenyl | 54.30 | 51.70 | 51.50 | 2.80 |
| 411 | Terbutryn | 55.80 | 57.90 | 58.60 | -2.80 |
| 412 | 1,2,3,5,7-Pentachloronaphthalene | 61.00 | 64.00 | 63.80 | -2.80 |
| 413 | Diethylketone | 49.50 | 53.40 | 52.30 | -2.80 |
| 414 | N-Methyl-N-(4-nitrophenyl)nitramine | 60.00 | 60.90 | 62.80 | -2.80 |
| 415 | 4-Octyloxy-N-(4-methoxybenzylidene)aniline | 112.10 | 116.40 | 114.90 | -2.80 |
| 416 | Chlorpyrifos | 81.99 | 87.50 | 84.80 | -2.81 |
| 417 | Acetophenone | 42.26 | 44.90 | 45.10 | -2.84 |
| 418 | Azinphos-ethyl | 78.26 | 82.40 | 81.10 | -2.84 |
| 419 | 1,3-Dithiane sulfoxide | 62.45 | 64.10 | 65.30 | -2.85 |
| 420 | 4-(4-Fluorophenyl)cyclohexyl trans-2-butenoate | 70.86 | 68.40 | 68.00 | 2.86 |
| 421 | 2,3,5-Triiodobenzoic acid | 63.97 | 61.60 | 61.10 | 2.87 |
| 422 | Bicyclopentyl | 57.87 | 57.10 | 55.00 | 2.87 |
| 423 | Prometryn | 63.38 | 64.30 | 60.50 | 2.88 |
| 424 | 1,4-Dimethyl-2,5-piperazinedione | 52.70 | 50.30 | 49.80 | 2.90 |
| 425 | 2-Phenyl-5,5-dimethyl-1,3-dioxane | 60.50 | 57.30 | 57.60 | 2.90 |
| 426 | 3-Bromochlorobenzene | 48.80 | 51.80 | 51.70 | -2.90 |
| 427 | 2,3,7,8-Tetrachlorodibenzo-p-dioxin | 69.00 | 69.30 | 71.90 | -2.90 |
| 428 | Bicyclo[2.2.2]oct-2-ene | 47.55 | 44.60 | 44.60 | 2.95 |
| 429 | Naproxen | 66.96 | 63.90 | 64.00 | 2.96 |
| 430 | 6,9-Dimethyl-8-butyladenine | 87.98 | 85.00 |  | 2.98 |
| 431 | Diethamine | 78.29 | 75.80 | 75.30 | 2.99 |
| 432 | 1-(4-Nitrophenylethynyl)-4-pentyloxybenzene | 93.30 | 90.10 | 90.30 | 3.00 |
| 433 | Suberic acid | 102.80 | 100.00 | 99.80 | 3.00 |
| 434 | Tetramethylpyromellitate | 85.40 | 83.50 | 82.40 | 3.00 |
| 435 | 3-(2,4-Dimethylbenzoyl)-1,2,2-trimethylcyclopentanecarboxylic acid | 48.60 | 46.20 | 45.60 | 3.00 |
| 436 | Ethyl 4-aminobenzoate | 60.60 | 62.70 | 63.60 | -3.00 |
| 437 | Methyl 3,4-dichlorophenylcarbamate | 60.80 | 63.60 | 63.80 | -3.00 |
| 438 | 4,4'-Di-t-butylbiphenyl | 49.90 | 52.30 | 52.90 | -3.00 |
| 439 | Fluorobenzene | 44.99 | 48.20 | 48.00 | -3.01 |
| 440 | 3-Diphenylmethyl-2,4-pentanedione (enol form) | 69.78 | 73.20 | 72.80 | -3.02 |
| 441 | 3-(3-Fluorophenyl)-3-hydroxypropanoic acid | 78.03 | 75.50 | 75.00 | 3.03 |
| 442 | 1,2-Butadiene | 50.84 | 48.50 | 47.80 | 3.04 |
| 443 | Phenyltrichlorosilane | 49.96 | 52.40 | 53.00 | -3.04 |
| 444 | 2-Fluorenyl-2-methyl-1,3-cyclopentanedione | 62.25 | 59.80 | 59.20 | 3.05 |
| 445 | Acetamide | 44.15 | 41.50 | 41.10 | 3.05 |
| 446 | 2-Acetyl-1-naphthol | 60.57 | 56.70 | 57.50 | 3.07 |
| 447 | cis-Hydrindan | 45.33 | 48.00 | 48.40 | -3.07 |
| 448 | 2-Methyl-2-nitro-1-propanol | 65.82 | 68.10 | 68.90 | -3.08 |
| 449 | 2,4,6-Tri-t-butylphenol | 48.01 | 51.10 | 51.10 | -3.09 |
| 450 | Flurenol-butyl | 74.31 | 77.40 | 77.40 | -3.09 |
| 451 | 1-Methyl-7-isopropylphenanthrene | 48.90 | 46.50 | 45.80 | 3.10 |
| 452 | N-Propylthalidomide | 66.70 | 64.90 | 63.60 | 3.10 |
| 453 | 2-(Phenoxymethyl)oxirane | 61.90 | 64.40 | 65.00 | -3.10 |
| 454 | Chloro-2,6-dinitrobenzene | 52.50 | 55.90 | 55.60 | -3.10 |
| 455 | 4-Propyl-3',4'-difluorodiphenyldiacetylene | 64.10 | 66.60 | 67.20 | -3.10 |
| 456 | D-Mannose | 63.10 | 66.20 | 66.20 | -3.10 |
| 457 | Benzo[c]fluorene | 49.20 | 51.90 | 52.30 | -3.10 |
| 458 | Dimethyl phthalate | 61.80 | 65.00 | 64.90 | -3.10 |
| 459 | 4-Nitrotoluene | 50.21 | 46.40 | 47.10 | 3.11 |
| 460 | 4-Butyl-3',4'-difluorodiphenyldiacetylene | 71.39 | 75.00 | 74.50 | -3.11 |
| 461 | Butanal | 61.11 | 58.20 | 58.00 | 3.11 |
| 462 | Cyclohexanecarbonitrile | 47.28 | 50.20 | 50.40 | -3.12 |
| 463 | Monolinuron | 63.78 | 66.90 |  | -3.12 |
| 464 | Tridiphane | 59.17 | 59.80 | 62.30 | -3.13 |
| 465 | 1,3-Dimethyladamantane | 37.06 | 33.50 | 33.90 | 3.16 |
| 466 | Cyclohexylbenzene | 54.44 | 57.80 | 57.60 | -3.16 |
| 467 | 1,2,5,6-Tetra(methoxycarbonyl)naphthalene | 89.37 | 85.40 | 86.20 | 3.17 |
| 468 | 1,6-Hexanedioic acid | 80.43 | 83.10 | 83.60 | -3.17 |
| 469 | 6,9-Dimethyl-8-propyladenine | 73.32 | 76.50 |  | -3.18 |
| 470 | 1,1-Dimethylcyclohexane | 47.42 | 50.50 | 50.60 | -3.18 |
| 471 | 2-Chloro-N,N-dimethylnicotinamide | 62.51 | 64.50 | 65.70 | -3.19 |
| 472 | Etonogestrel | 66.00 | 61.00 | 62.80 | 3.20 |
| 473 | 1,1,4,4-Tetramethylcyclohexadecane | 82.80 | 86.00 | 86.00 | -3.20 |
| 474 | Sulfamethoxazole | 76.70 | 74.20 | 73.50 | 3.20 |
| 475 | Pentabromophenol | 63.70 | 61.00 | 60.50 | 3.20 |
| 476 | trans-4-Methoxycinnamic acid | 60.80 | 64.30 | 64.00 | -3.20 |
| 477 | Cyclopentadiene | 45.40 | 49.50 | 48.60 | -3.20 |
| 478 | o-Toluidine | 45.10 | 48.20 | 48.30 | -3.20 |
| 479 | Butylcyclohexane | 71.70 | 74.80 | 74.90 | -3.20 |
| 480 | Dodecanedioic acid | 130.70 | 133.90 | 133.90 | -3.20 |
| 481 | 14-Octadecynoic acid | 156.50 | 159.50 | 159.70 | -3.20 |
| 482 | 2-Piperidone | 47.02 | 44.50 | 43.80 | 3.22 |
| 483 | 3-(2-Fluorophenyl)-3-hydroxypropanoic acid | 79.52 | 75.50 | 76.30 | 3.22 |
| 484 | 2-Iodonaphthalene | 48.96 | 52.20 | 52.20 | -3.24 |
| 485 | 2-Furoic acid | 56.15 | 53.10 | 52.90 | 3.25 |
| 486 | 4-Propyl-3',4'-difluorodiphenylacetylene | 64.95 | 67.60 | 68.20 | -3.25 |
| 487 | N-(2-Cyanoethyl)aniline | 63.17 | 59.70 | 59.90 | 3.27 |
| 488 | Ethane | 31.93 | 34.90 | 35.20 | -3.27 |
| 489 | Dibenzo[b,e]pyridine | 48.48 | 46.00 | 45.20 | 3.28 |
| 490 | 2-Bromoiodobenzene | 49.01 | 52.40 | 52.30 | -3.29 |
| 491 | N,N'-Diethylurea | 38.01 | 41.50 | 41.30 | -3.29 |
| 492 | Azinphos-methyl | 80.40 | 78.40 | 77.10 | 3.30 |
| 493 | 1,3-Dimethyl-2,4-dinitro-5-t-butylbenzene | 49.00 | 46.60 | 45.70 | 3.30 |
| 494 | 2-Hydroxyacetanilide | 58.30 | 54.50 | 55.00 | 3.30 |
| 495 | 3-(Biphenyl-4-ylcarbonyl)-1,2,2-trimethylcyclopentanecarboxylic acid | 62.30 | 58.10 | 59.00 | 3.30 |
| 496 | Dibenz[ah]anthracene | 57.30 | 54.30 | 54.00 | 3.30 |
| 497 | 1,3-Diiodobenzene | 51.90 | 54.00 | 55.20 | -3.30 |
| 498 | 1-Pentene | 55.00 | 57.90 | 58.30 | -3.30 |
| 499 | Ethylcyclopentane | 51.00 | 54.00 | 54.30 | -3.30 |
| 500 | 1,3,5-Trimethyluracil | 37.60 | 39.60 | 40.90 | -3.30 |
| 501 | 4-Heptanone | 67.30 | 70.30 | 70.60 | -3.30 |
| 502 | 4-Methylbenzoic acid | 50.21 | 47.20 | 46.90 | 3.31 |
| 503 | 1,2,4,5-Tetra(methoxycarbonyl)naphthalene | 82.89 | 85.40 | 86.20 | -3.31 |
| 504 | 2-Iodofluorene | 49.76 | 53.70 | 53.10 | -3.34 |
| 505 | Dibenzo[b,d]thiophene | 58.17 | 55.70 | 54.80 | 3.37 |
| 506 | 4-alpha-Cumylphenol | 62.58 | 58.90 | 59.20 | 3.38 |
| 507 | N,N,N',N'-Tetramethyldithiooxamide | 51.32 | 53.70 | 54.70 | -3.38 |
| 508 | 4-Propoxy-2',4'-difluorodiphenylacetylene | 77.09 | 73.40 | 73.70 | 3.39 |
| 509 | 2,5-Norbornadiene | 48.10 | 45.00 | 44.70 | 3.40 |
| 510 | 1,2-Dihydroxybenzene | 58.00 | 53.80 | 54.60 | 3.40 |
| 511 | Chrysene | 55.50 | 52.40 | 52.10 | 3.40 |
| 512 | 2-Amino-5-methylbenzoic acid | 61.40 | 58.50 | 58.00 | 3.40 |
| 513 | Acetylacetone (enol form) | 56.90 | 54.20 | 53.50 | 3.40 |
| 514 | Methylcyclohexane | 46.10 | 49.40 | 49.50 | -3.40 |
| 515 | Trans-1,4-bis(1,1-dimethylethyl)cyclohexane | 47.20 | 50.70 | 50.60 | -3.40 |
| 516 | Propyl 2-cyano-6-nitro-1(2H)-quinolinecarboxylate | 67.20 | 69.70 | 70.60 | -3.40 |
| 517 | 1,2,3,6,7,8-Hexahydropyrene | 57.69 | 61.00 | 61.10 | -3.41 |
| 518 | 2-Naphthylamine | 57.32 | 53.40 | 53.90 | 3.42 |
| 519 | Pyridine | 49.22 | 46.60 | 45.80 | 3.42 |
| 520 | MCPB | 85.73 | 81.40 | 82.30 | 3.43 |
| 521 | Niflumic acid | 79.83 | 77.00 | 76.40 | 3.43 |
| 522 | 2,2,4,4-Tetramethylpentane | 47.16 | 50.50 | 50.60 | -3.44 |
| 523 | Pyrene | 43.36 | 47.20 | 46.80 | -3.44 |
| 524 | Diisopropyl ether | 59.18 | 55.30 | 55.70 | 3.48 |
| 525 | Triamcinolone | 78.39 | 74.60 | 74.90 | 3.49 |
| 526 | 2-Benzoylpyridine | 66.10 | 62.70 | 62.60 | 3.50 |
| 527 | Enilconazole | 90.00 | 87.30 | 86.50 | 3.50 |
| 528 | Palmitic acid | 163.50 | 160.50 | 160.00 | 3.50 |
| 529 | 1-Methyl-9H-pyrido[3,4-b]indole | 53.30 | 56.70 | 56.80 | -3.50 |
| 530 | 1,6-Anhydro-beta-D-galactopyranose | 56.90 | 59.00 | 60.40 | -3.50 |
| 531 | Dimethyl sulfone | 47.91 | 46.80 | 44.40 | 3.51 |
| 532 | 2-Bromofluorene | 48.78 | 52.10 | 52.30 | -3.52 |
| 533 | 1,2-Diaminoethane | 79.43 | 79.60 | 75.90 | 3.53 |
| 534 | 4-Chloroaniline | 57.74 | 54.60 | 54.20 | 3.54 |
| 535 | 1,1-Dicyclohexyldodecane | 147.54 | 151.10 | 151.10 | -3.56 |
| 536 | 4-Propoxybenzoic acid | 65.94 | 69.90 | 69.50 | -3.56 |
| 537 | 1,2,4-Tribromobenzene | 56.50 | 53.00 | 52.90 | 3.60 |
| 538 | 1,6-Cyclodecanedione | 79.50 | 75.40 | 75.90 | 3.60 |
| 539 | 1-Bromo-4-chlorobenzene | 55.50 | 51.80 | 51.90 | 3.60 |
| 540 | 10-Octadecynoic acid | 164.00 | 159.50 | 160.40 | 3.60 |
| 541 | 3-Chlorothiophene | 43.80 | 42.10 | 40.20 | 3.60 |
| 542 | 1-Butyne | 40.90 | 43.80 | 44.50 | -3.60 |
| 543 | Fenpropathrin | 57.60 | 63.70 | 61.20 | -3.60 |
| 544 | 4-Methoxyphenol | 55.70 | 59.10 | 59.30 | -3.60 |
| 545 | Lifibrol | 91.80 | 95.70 | 95.40 | -3.60 |
| 546 | Dodecanedinitrile | 116.70 | 121.00 | 120.30 | -3.60 |
| 547 | Lenacil | 72.41 | 68.70 | 68.80 | 3.61 |
| 548 | Propham | 53.88 | 57.40 | 57.50 | -3.62 |
| 549 | 2,3-Lutidine | 52.13 | 49.50 | 48.50 | 3.63 |
| 550 | Benzamide | 45.96 | 49.90 | 49.60 | -3.64 |
| 551 | Acetanilide | 47.26 | 50.80 | 50.90 | -3.64 |
| 552 | 1,1-Dimethyl-3-phenylurea | 56.35 | 59.00 | 60.00 | -3.65 |
| 553 | 1-Dodecanol | 133.76 | 130.10 | 130.10 | 3.66 |
| 554 | 3-Dodecyloxy-1,2-propanediol | 159.13 | 163.10 | 162.80 | -3.67 |
| 555 | 1-Propanethiol | 62.18 | 58.00 | 58.50 | 3.68 |
| 556 | Pinacol | 46.49 | 44.00 | 42.80 | 3.69 |
| 557 | 3-Heptanone | 74.30 | 70.30 | 70.60 | 3.70 |
| 558 | 4'-Propylbiphenyl-4-carbonitrile | 67.00 | 62.80 | 63.30 | 3.70 |
| 559 | 5,5,8,8,16,16,19,19-Octamethylcyclodocosane-1,12-dione | 107.90 | 104.30 | 104.20 | 3.70 |
| 560 | N -(2-Trimethylsilylethyl)trimethylenimine | 64.60 | 69.30 | 68.30 | -3.70 |
| 561 | 1,3,5-Trichlorobenzene | 52.30 | 55.90 | 56.00 | -3.70 |
| 562 | 2,2-Dinitropropanol | 61.20 | 59.00 | 57.50 | 3.70 |
| 563 | Vitamin C | 79.50 | 81.50 | 83.20 | -3.70 |
| 564 | Bifenox | 74.00 | 78.00 | 77.70 | -3.70 |
| 565 | 12-Octadecynoic acid | 155.60 | 159.50 | 159.30 | -3.70 |
| 566 | Chlorothalonil | 57.01 | 54.00 | 53.30 | 3.71 |
| 567 | 1-(Ethylamino)-3,5-bis(dimethylamino)-s-triazine | 50.27 | 53.90 | 54.00 | -3.73 |
| 568 | 1-Piperidinyl-3,5-bis(dimethylamino)-s-triazine | 64.23 | 58.50 | 60.50 | 3.73 |
| 569 | 2-(Dimethylamino)-1,2-diphenylethanone | 66.97 | 70.50 | 70.70 | -3.73 |
| 570 | 2,4,5-Trichlorophenol | 63.44 | 59.50 | 59.70 | 3.74 |
| 571 | 3-Hydroxy-3-phenylvaleric acid | 81.69 | 78.20 | 77.90 | 3.79 |
| 572 | 4-Methylphenanthrene | 43.21 | 47.20 | 47.00 | -3.79 |
| 573 | Carbanilide | 66.50 | 62.00 | 62.70 | 3.80 |
| 574 | beta-Carboline | 54.00 | 50.50 | 50.20 | 3.80 |
| 575 | Sulfacetamide | 65.50 | 62.30 | 61.70 | 3.80 |
| 576 | Musk xylene | 53.80 | 49.80 | 50.00 | 3.80 |
| 577 | 2,4,6-Trinitroresorcinol | 73.60 | 75.90 | 77.40 | -3.80 |
| 578 | 3,4-Dimethoxybenzaldehyde | 64.10 | 67.60 | 67.90 | -3.80 |
| 579 | 2-(4-Nitrophenyl)-1-(4-ethoxyphenyl)ethanone | 72.30 | 75.80 | 76.10 | -3.80 |
| 580 | Bicyclo[2.2.2]octane | 46.71 | 43.40 | 42.90 | 3.81 |
| 581 | 1-Hexacosanol | 243.09 | 248.60 | 246.90 | -3.81 |
| 582 | 3,5-Dichlorophenol | 60.14 | 56.40 | 56.30 | 3.84 |
| 583 | Acetic acid | 38.36 | 42.10 | 42.20 | -3.84 |
| 584 | Cyanogen | 33.05 | 36.40 | 36.90 | -3.85 |
| 585 | 1-Chloronaphthalene | 47.65 | 51.60 | 51.50 | -3.85 |
| 586 | 4-Bromoaniline | 49.85 | 53.60 | 53.70 | -3.85 |
| 587 | Nitroethane | 53.64 | 53.60 | 57.50 | -3.86 |
| 588 | trans-Hydrindan | 51.08 | 48.00 | 47.20 | 3.88 |
| 589 | 4,7-Dimethylindane | 49.58 | 46.60 | 45.70 | 3.88 |
| 590 | 1,3-Benzodioxol-5-ol | 50.22 | 52.90 | 54.10 | -3.88 |
| 591 | p-Chloromandelic acid | 58.41 | 62.20 | 62.30 | -3.89 |
| 592 | 4-Hydroxyphenylacetic acid | 66.20 | 62.60 | 62.30 | 3.90 |
| 593 | Dimethylphenylvinylsilane | 64.40 | 60.50 |  | 3.90 |
| 594 | 2,3,5,6-Tetramethyl-1,4-benzoquinone | 47.90 | 43.10 | 44.00 | 3.90 |
| 595 | Glycerol trinitrate | 76.60 | 77.70 | 80.50 | -3.90 |
| 596 | 2,2'-Bithiophene | 54.20 | 54.70 | 58.10 | -3.90 |
| 597 | 2-Indanone | 50.70 | 55.00 | 54.60 | -3.90 |
| 598 | 3-((2,3-Dihydro-1H-inden-5-yl)carbonyl)-1,2,2-trimethylcyclopentanecarboxylic acid | 55.70 | 59.40 | 59.60 | -3.90 |
| 599 | Salicylic acid | 56.70 | 60.40 | 60.60 | -3.90 |
| 600 | 2,3-Bis(trifluoromethyl)-4-methoxyphenyl-alpha,alpha-difluoro-4-propylbenzyl ether | 70.70 | 66.80 |  | 3.90 |
| 601 | Pentacosafluorodocosane | 103.30 | 111.60 | 107.20 | -3.90 |
| 602 | 4-Isobutyl-alpha-methylphenylacetic acid | 57.50 | 60.70 | 61.40 | -3.90 |
| 603 | Cholesteryl 2-bromopropionate | 87.20 | 91.10 |  | -3.90 |
| 604 | 1,11-Undecanediamine | 153.30 | 155.70 | 157.20 | -3.90 |
| 605 | 4-Pentyl-3',4'-difluorodiphenyldiacetylene | 86.91 | 83.50 | 83.00 | 3.91 |
| 606 | 1,10-Cyclooctadecanedione | 105.78 | 107.70 | 109.70 | -3.92 |
| 607 | Fenoxycarb | 82.68 | 84.60 | 86.60 | -3.92 |
| 608 | 4-Nitrophthalic anhydride | 44.15 | 47.80 | 48.10 | -3.95 |
| 609 | 2-Methyl-2-nitro-1,3-propanediol | 82.14 | 85.00 | 86.10 | -3.96 |
| 610 | 2,2-Dichloropropane | 41.42 | 45.20 | 45.40 | -3.98 |
| 611 | 2,2-Dimethyl-1,3-dioxane | 52.70 | 50.00 | 48.70 | 4.00 |
| 612 | N-(3-Methoxyphenylmethyl)-N'-octylthiourea | 108.20 | 113.60 | 112.20 | -4.00 |
| 613 | Ethyl-2-cyanoacrylate | 52.90 | 55.90 | 56.90 | -4.00 |
| 614 | Diphenamid | 62.47 | 67.10 | 66.50 | -4.03 |
| 615 | 1,4-Dimethylnaphthalene | 37.87 | 42.00 | 41.90 | -4.03 |
| 616 | Methyl acrylate | 49.26 | 53.40 | 53.30 | -4.04 |
| 617 | Benzylaniline | 54.84 | 58.90 | 58.90 | -4.06 |
| 618 | 4-Nitroguaiacol | 57.93 | 62.30 | 62.00 | -4.07 |
| 619 | Methylnitrate | 43.33 | 47.90 | 47.40 | -4.07 |
| 620 | 1,5-Dimethyltetrazole | 42.12 | 45.10 | 46.20 | -4.08 |
| 621 | Ethyl vanillin | 66.00 | 64.30 | 61.90 | 4.10 |
| 622 | 1,4-Benzoquinone | 47.80 | 44.10 | 43.70 | 4.10 |
| 623 | Phenylurea | 56.30 | 52.70 | 52.20 | 4.10 |
| 624 | trans-9,10-Bishydroxymethyl-9,10-dihydrophenanthrene | 67.20 | 63.30 | 63.10 | 4.10 |
| 625 | 1,4-Dioxane-2,5-dione | 47.40 | 51.20 | 51.50 | -4.10 |
| 626 | Tetradecyl urea | 140.40 | 144.00 | 144.50 | -4.10 |
| 627 | Naphthalic anhydride | 43.00 | 46.50 | 47.10 | -4.10 |
| 628 | N-Isopropylcarbazole | 51.60 | 55.00 | 55.70 | -4.10 |
| 629 | 2-Hydroxybenzylalcohol | 60.00 | 63.90 | 64.10 | -4.10 |
| 630 | Diisobutylene | 48.99 | 52.70 | 53.10 | -4.11 |
| 631 | 2-Amino-2-methyl-1,3-propanediol | 77.38 | 82.30 | 81.50 | -4.12 |
| 632 | Zectran | 50.78 | 54.70 | 54.90 | -4.12 |
| 633 | n-Undecanoic acid | 114.18 | 118.20 | 118.30 | -4.12 |
| 634 | Abate | 108.94 | 106.10 | 104.80 | 4.14 |
| 635 | 2,4-Dimethylpentane | 44.46 | 48.50 | 48.60 | -4.14 |
| 636 | Dicofol | 56.35 | 61.40 | 60.50 | -4.15 |
| 637 | Chloropropham | 56.55 | 60.50 | 60.70 | -4.15 |
| 638 | Benzo[c]phenanthrene | 48.75 | 52.40 | 52.90 | -4.15 |
| 639 | Benzyl benzoate | 69.76 | 66.10 | 65.60 | 4.16 |
| 640 | 1-Hexene | 70.10 | 66.40 | 65.90 | 4.20 |
| 641 | 2,5-Xylidine | 49.10 | 45.00 | 44.90 | 4.20 |
| 642 | 1-Methoxy-a-D-glucopyranoside | 88.60 | 84.20 | 84.40 | 4.20 |
| 643 | Diethylcarbonate | 46.60 | 42.90 | 42.40 | 4.20 |
| 644 | Dimethylaminoethyl methacrylate | 70.90 | 67.40 | 66.70 | 4.20 |
| 645 | N-2-(6-Picolyl)-N'-3-tolylthiourea | 72.10 | 76.70 | 76.30 | -4.20 |
| 646 | 1-Methylnaphthalene | 49.30 | 45.20 | 45.10 | 4.20 |
| 647 | 2-Chlorophenylacetic acid | 66.20 | 62.00 | 62.00 | 4.20 |
| 648 | Bromo-2-chlorobenzene | 47.50 | 51.80 | 51.70 | -4.20 |
| 649 | 2-Naphthol | 47.70 | 52.10 | 51.90 | -4.20 |
| 650 | Maltitol | 131.10 | 137.70 | 135.30 | -4.20 |
| 651 | Glibenclamide | 103.60 | 106.80 | 107.80 | -4.20 |
| 652 | Benzo[b]fluorene | 47.78 | 51.90 | 52.00 | -4.22 |
| 653 | 1,1-Dimethylindane | 52.73 | 48.20 | 48.50 | 4.23 |
| 654 | 2,4,5-Trichlorobiphenyl | 65.24 | 61.00 | 61.00 | 4.24 |
| 655 | 1,1,3,3-Tetraphenyl-1,3-dimethyldisiloxane | 82.55 | 85.50 | 86.80 | -4.25 |
| 656 | Methylparathion | 65.12 | 69.80 | 69.40 | -4.28 |
| 657 | Oxadiazon | 73.19 | 71.70 | 68.90 | 4.29 |
| 658 | Docosamethyl-1,11-diphenyl-undecasilane | 145.20 | 140.90 |  | 4.30 |
| 659 | 1-Methyl-4-isopropylcyclohexene | 57.10 | 53.00 | 52.80 | 4.30 |
| 660 | 2,2,2-Trinitroethanol | 65.50 | 61.60 | 61.20 | 4.30 |
| 661 | o-Terphenyl | 52.30 | 56.80 | 56.60 | -4.30 |
| 662 | 4,6-Dimethylindane | 50.21 | 46.60 | 45.90 | 4.31 |
| 663 | 2-Methylhexane | 59.29 | 62.80 | 63.60 | -4.31 |
| 664 | Benzylbromide | 48.57 | 53.20 | 52.90 | -4.33 |
| 665 | 3,3-Dimethylpentane | 51.16 | 55.40 | 55.50 | -4.34 |
| 666 | 1-(4-Methylpiperidinyl)-3,5-bis(dimethylamino)-s-triazine | 57.65 | 52.60 | 53.30 | 4.35 |
| 667 | p-Quaterphenyl | 66.15 | 61.90 | 61.80 | 4.35 |
| 668 | 3,4-Dihydro-1H-2-benzopyran | 60.35 | 55.80 | 56.00 | 4.35 |
| 669 | 1-Methylphenanthrene | 43.44 | 47.20 | 47.80 | -4.36 |
| 670 | 1-Chloroadamantane | 35.62 | 40.00 | 40.00 | -4.38 |
| 671 | 1,2,6,7,8,9-Hexahydro-1,6,6-trimethylphenanthro[1,2-b]furan-10,11-dione | 57.00 | 53.40 | 52.60 | 4.40 |
| 672 | 1H,1H-Perfluorododecan-1-ol | 50.50 | 46.10 |  | 4.40 |
| 673 | 2,4,4'-Trichlorobiphenyl | 56.60 | 61.00 | 61.00 | -4.40 |
| 674 | N,N'-Dimethylurea | 35.90 | 39.30 | 40.30 | -4.40 |
| 675 | 1-(4-Chlorobenzoyl)-1,2-dihydro-6-nitro-2-quinolinecarbonitrile | 59.50 | 64.20 | 63.90 | -4.40 |
| 676 | Ethyl heptadecanoate | 171.20 | 176.30 | 175.60 | -4.40 |
| 677 | Benzenesulfonamide | 59.11 | 55.60 | 54.70 | 4.41 |
| 678 | 2,2-Dimethyl-1,3-propanediol | 55.19 | 59.70 | 59.60 | -4.41 |
| 679 | Bis(4-chlorophenyl)acetic acid | 71.92 | 67.60 | 67.50 | 4.42 |
| 680 | 1-Methyladamantane | 27.68 | 32.40 | 32.10 | -4.42 |
| 681 | p-Cymene | 47.33 | 42.60 | 42.90 | 4.43 |
| 682 | Neohexane | 42.57 | 47.00 | 47.00 | -4.43 |
| 683 | Decacyclene | 38.14 | 36.80 | 33.70 | 4.44 |
| 684 | 4-Propoxy-4'-fluorodiphenylacetylene | 75.95 | 71.70 | 71.50 | 4.45 |
| 685 | 2-Iodobenzoic acid | 49.14 | 54.20 | 53.60 | -4.46 |
| 686 | Benz[a]anthracene | 49.23 | 52.40 | 53.70 | -4.47 |
| 687 | Butyl 6-(acetylamino)-2-cyano-1(2H)-quinolinecarboxylate | 83.60 | 79.40 | 79.10 | 4.50 |
| 688 | 2-Iodoaniline | 58.80 | 55.20 | 54.30 | 4.50 |
| 689 | Ethyl 2-cyano-6-methoxy-1(2H)-quinolinecarboxylate | 62.30 | 67.10 | 66.80 | -4.50 |
| 690 | 2,4-Dichlorobenzaldehyde | 59.00 | 55.90 | 54.50 | 4.50 |
| 691 | Isobutyramide | 48.00 | 44.10 | 43.50 | 4.50 |
| 692 | Cyclodecane | 67.00 | 71.50 | 71.50 | -4.50 |
| 693 | Propyl phenylcarbamate | 63.68 | 67.90 | 68.20 | -4.52 |
| 694 | 1-Bromobutane | 57.57 | 61.70 | 62.10 | -4.53 |
| 695 | 1-Bromodocosane | 217.95 | 214.00 | 213.40 | 4.55 |
| 696 | 4-(4-Bromophenyl)cyclohexyl trans-2-butenoate | 73.16 | 68.90 | 68.60 | 4.56 |
| 697 | 1-Chloro-2-[2,2,2-trichloro-1-(4-chlorophenyl)ethyl]benzene | 66.78 | 59.40 | 62.20 | 4.58 |
| 698 | 1,3,5,7-Tetramethyladamantane | 29.12 | 35.50 | 33.70 | -4.58 |
| 699 | 4-Ethyl-3',4'-difluorodiphenylacetylene | 55.11 | 59.20 | 59.70 | -4.59 |
| 700 | 2-Chlorobiphenyl | 50.21 | 54.80 | 54.80 | -4.59 |
| 701 | 4,4'-Dichlorodiphenyl sulphone | 57.80 | 53.50 | 53.20 | 4.60 |
| 702 | 1,2,4,5,6-Pentachloronaphthalene | 59.10 | 64.00 | 63.70 | -4.60 |
| 703 | Pentacosofluorooctadecane | 72.10 | 77.70 | 76.70 | -4.60 |
| 704 | 3,3,6,6-Tetramethyloctanedioic anhydride | 54.70 | 58.30 | 59.30 | -4.60 |
| 705 | 4-(Phenylmethoxy)benzeneacetic acid | 74.00 | 78.20 | 78.60 | -4.60 |
| 706 | 1-Benzopyran-4-one | 52.41 | 47.90 | 47.80 | 4.61 |
| 707 | Methyltrichlorosilane | 45.31 | 44.00 | 40.70 | 4.61 |
| 708 | Chlorocyclopentane | 48.59 | 53.80 | 53.20 | -4.61 |
| 709 | 2-Butanethiol | 48.69 | 52.90 | 53.30 | -4.61 |
| 710 | Hexane | 73.22 | 68.80 | 68.60 | 4.62 |
| 711 | 1-Iodonaphthalene | 56.82 | 52.20 | 52.20 | 4.62 |
| 712 | Quinoline | 41.58 | 46.30 | 46.20 | -4.62 |
| 713 | 1,3-Diphenylacetone | 65.78 | 70.20 | 70.40 | -4.62 |
| 714 | Styrene | 45.16 | 49.30 | 49.80 | -4.64 |
| 715 | 2-Methoxy-5-nitrophenol | 56.75 | 62.30 | 61.40 | -4.65 |
| 716 | 1,2,3,4-Tetrahydronaphthalene | 52.44 | 56.80 | 57.10 | -4.66 |
| 717 | Dipropyl sulfide | 71.24 | 75.30 | 75.90 | -4.66 |
| 718 | 1,2,3,4-Tetrahydrophenanthrene | 63.27 | 58.80 | 58.60 | 4.67 |
| 719 | 4,7-Phenanthroline | 49.70 | 46.00 | 45.00 | 4.70 |
| 720 | Quinaldine | 46.30 | 52.40 | 51.00 | -4.70 |
| 721 | Metalaxyl | 79.00 | 83.70 | 83.70 | -4.70 |
| 722 | 2-Diphenylmethyl-2-ethyl-1,3-cyclopentanedione | 73.78 | 78.40 | 78.50 | -4.72 |
| 723 | Isobutylene | 44.72 | 40.70 | 40.00 | 4.72 |
| 724 | 1,3-Difluorobenzene | 46.48 | 49.90 | 51.20 | -4.72 |
| 725 | 3-Chloro-N-phenylphthalimide | 62.53 | 57.30 | 57.80 | 4.73 |
| 726 | 2-Butyne | 38.33 | 33.90 | 33.60 | 4.73 |
| 727 | Tribenzo[a,e,i]pyrene | 47.37 | 53.00 | 52.10 | -4.73 |
| 728 | 2,2',4,5-Tetrachlorobiphenyl | 69.04 | 64.10 | 64.30 | 4.74 |
| 729 | 3-Furancarboxylic acid | 53.95 | 50.60 | 49.20 | 4.75 |
| 730 | 1,2,3,4-Tetrahydroquinoline | 40.75 | 47.10 | 45.50 | -4.75 |
| 731 | Cumene | 41.45 | 45.80 | 46.20 | -4.75 |
| 732 | Bromobenzene | 43.84 | 48.70 | 48.60 | -4.76 |
| 733 | 3,4-Dichlorophenol | 61.37 | 56.40 | 56.60 | 4.77 |
| 734 | Ethyl acetate | 55.27 | 49.40 | 50.50 | 4.77 |
| 735 | 3-Hexanone | 66.59 | 61.90 | 61.80 | 4.79 |
| 736 | Paracetamol | 58.99 | 54.50 | 54.20 | 4.79 |
| 737 | N,N'-Dibutylurea | 78.50 | 75.40 | 73.70 | 4.80 |
| 738 | Benzothiazole | 46.40 | 43.60 | 41.60 | 4.80 |
| 739 | 1-Methylisatin | 48.40 | 43.60 | 43.60 | 4.80 |
| 740 | Ethyl 8-(hydroxymethyl)-6-methoxy-2-oxo-2H-1-benzopyran-3-carboxylate | 84.30 | 79.70 | 79.50 | 4.80 |
| 741 | cis-1,3-Pentadiene | 42.60 | 45.90 | 47.40 | -4.80 |
| 742 | 1,6-Dimethylphenanthro[1,2-b]furan-10,11-dione | 44.60 | 48.50 | 49.40 | -4.80 |
| 743 | Tetracyclopropylsuccinonitrile | 57.18 | 59.70 | 62.00 | -4.82 |
| 744 | 1,2,4-Tris(methoxycarbonyl)naphthalene | 81.53 | 76.20 | 76.70 | 4.83 |
| 745 | 2-Methylbenzimidazole | 46.93 | 41.60 | 42.10 | 4.83 |
| 746 | 2-Undecanone | 99.07 | 104.20 | 103.90 | -4.83 |
| 747 | Butyl ethyl sulfide | 69.57 | 75.30 | 74.40 | -4.83 |
| 748 | 4-Propoxy-2',3',4'-trifluorodiphenylacetylene | 79.74 | 75.00 | 74.90 | 4.84 |
| 749 | Pronamide | 66.94 | 59.40 | 62.10 | 4.84 |
| 750 | Hexanamide | 70.66 | 75.40 | 75.50 | -4.84 |
| 751 | 3-Chlorophenol | 48.74 | 53.30 | 53.60 | -4.86 |
| 752 | 2,3-Bis(methoxycarbonyl)naphthalene | 62.31 | 66.90 | 67.20 | -4.89 |
| 753 | Isooctane | 54.70 | 49.50 | 49.80 | 4.90 |
| 754 | 1,3-Dioxolane | 56.10 | 51.40 | 51.20 | 4.90 |
| 755 | 4-Nitroaniline | 50.10 | 54.60 | 55.00 | -4.90 |
| 756 | N-Decyl-L-valine | 96.80 | 101.80 | 101.70 | -4.90 |
| 757 | 1,12-Dodecanediol | 145.50 | 140.80 | 140.60 | 4.90 |
| 758 | Heptylcyclohexane | 95.50 | 100.20 | 100.40 | -4.90 |
| 759 | Methyl 2-(2,4,5-trichlorophenoxy)butyrate | 91.22 | 85.80 | 86.30 | 4.92 |
| 760 | Adamantane | 36.32 | 31.40 | 31.40 | 4.92 |
| 761 | 3-Nitrophthalic anhydride | 42.18 | 47.80 | 47.10 | -4.92 |
| 762 | 1,1,4,7-Tetramethylindane | 45.93 | 41.70 | 41.00 | 4.93 |
| 763 | 4-Hexyloxy-2',3',4'-trifluorodiphenylacetylene | 95.65 | 100.40 | 100.60 | -4.95 |
| 764 | 1,8-Dimethylnaphthalene | 46.86 | 42.00 | 41.90 | 4.96 |
| 765 | 1-Bromonaphthalene | 55.86 | 50.60 | 50.90 | 4.96 |
| 766 | 2-Amino-9-((2-acetoxyethoxy)methyl)-1,9-dihydro-6H-purin-6-one | 96.86 | 84.20 | 91.90 | 4.96 |
| 767 | 2-Chlorodibenzo-p-dioxin | 63.78 | 60.00 | 58.80 | 4.98 |
| 768 | 3-Bromobenzoic acid | 57.90 | 52.60 | 52.90 | 5.00 |
| 769 | 4-Bromotoluene | 50.20 | 45.50 | 45.20 | 5.00 |
| 770 | Carvone | 46.60 | 53.80 | 51.60 | -5.00 |
| 771 | 3,6-Dichloro-5-hydroxy-2-methoxybenzoic acid | 70.70 | 75.60 | 75.70 | -5.00 |
| 772 | Ethyl 5-phenylsulfonyl-4,7-dihydro-4,7-ethano-2H-isoindole-1-carboxylate | 65.70 | 64.20 | 70.70 | -5.00 |
| 773 | N-(4-Chlorophenyl)benzenesulfonamide | 65.40 | 70.50 | 70.40 | -5.00 |
| 774 | 1,4-Bis(4-(4'-ethylbiphenyl))butane | 101.30 | 106.20 | 106.30 | -5.00 |
| 775 | 2-Hydroxydiphenylmethane | 58.99 | 63.80 | 64.00 | -5.01 |
| 776 | 2,2',4,5'-Tetrachlorobiphenyl | 69.01 | 64.10 | 64.00 | 5.01 |
| 777 | 1-Indanone | 56.03 | 50.60 | 51.00 | 5.03 |
| 778 | Di-n-butylsulfide | 98.05 | 92.20 | 93.00 | 5.05 |
| 779 | Camphor oxime | 39.35 | 44.30 | 44.40 | -5.05 |
| 780 | 9,10-Dihydroanthracene | 62.38 | 58.30 | 57.30 | 5.08 |
| 781 | 4-Iodoaniline | 50.42 | 55.20 | 55.50 | -5.08 |
| 782 | 3-(4-Chlorophenyl)-3-hydroxypropanoic acid | 72.81 | 77.00 | 77.90 | -5.09 |
| 783 | Arachidic acid | 198.70 | 194.40 | 193.60 | 5.10 |
| 784 | 1,2-Diaminobenzene | 62.00 | 56.40 | 56.90 | 5.10 |
| 785 | 2,3,7-Trichlorodibenzo-p-dioxin | 71.00 | 66.20 | 65.90 | 5.10 |
| 786 | Phenanthrene | 46.10 | 50.40 | 51.20 | -5.10 |
| 787 | 1,1,2-Trichloroethane | 45.70 | 50.80 | 50.80 | -5.10 |
| 788 | N-(3-Methylphenyl)-N,N-bis(4-methylphenyl)amine | 65.81 | 63.00 | 60.70 | 5.11 |
| 789 | 4-Pyridinamine | 46.68 | 51.50 | 51.80 | -5.12 |
| 790 | Sebaconitrile | 100.28 | 104.10 | 105.40 | -5.12 |
| 791 | N-(4,6-bis(dimethylamino)-1,3,5-triazin-2-yl)-N-methylglycine | 69.33 | 66.40 | 64.20 | 5.13 |
| 792 | Propoxur | 63.23 | 58.90 | 58.10 | 5.13 |
| 793 | Pentafluorophenol | 56.87 | 58.50 | 62.00 | -5.13 |
| 794 | 1-Butene | 43.84 | 49.40 | 49.00 | -5.16 |
| 795 | 1,3,5-Trimethylbenzene | 41.66 | 36.80 | 36.50 | 5.16 |
| 796 | cis- Cyclooctene | 58.53 | 64.70 | 63.70 | -5.17 |
| 797 | Caprylamide | 86.23 | 92.30 | 91.40 | -5.17 |
| 798 | Benzo[h]quinoline | 43.51 | 48.20 | 48.70 | -5.19 |
| 799 | Capraldehyde | 113.60 | 109.00 | 108.40 | 5.20 |
| 800 | 3,4-Dichloroaniline | 63.00 | 57.70 | 57.80 | 5.20 |
| 801 | 2,4-Dinitrobenzaldehyde | 61.40 | 56.00 | 56.20 | 5.20 |
| 802 | 10-Phenylacridin-9(10H)-one | 70.70 | 64.10 | 65.50 | 5.20 |
| 803 | 1-Chloro-4-fluorobenzene | 56.70 | 51.30 | 51.50 | 5.20 |
| 804 | Ethylurea | 37.90 | 42.50 | 43.10 | -5.20 |
| 805 | Hemimellitene | 41.81 | 36.80 | 36.60 | 5.21 |
| 806 | Pentachlorobenzene | 57.59 | 62.10 | 62.80 | -5.21 |
| 807 | 1,6-Cyclodecanedione bis(ethylene ketal) | 72.58 | 77.40 | 77.80 | -5.22 |
| 808 | Disperse Red 19 | 66.97 | 71.40 | 72.20 | -5.23 |
| 809 | Phthalazine | 36.54 | 31.30 |  | 5.24 |
| 810 | 1,3,8-Tris(methoxycarbonyl)naphthalene | 71.46 | 76.20 | 76.70 | -5.24 |
| 811 | Benzoic acid | 45.45 | 50.50 | 50.70 | -5.25 |
| 812 | Dimethylmalononitrile | 45.77 | 40.00 | 40.50 | 5.27 |
| 813 | 18-Butylpentatriacontane | 326.53 | 333.60 | 331.80 | -5.27 |
| 814 | 4,6-Dinitro-o-cresol | 54.02 | 59.50 | 59.30 | -5.28 |
| 815 | Methylhydrazine | 47.19 | 41.90 |  | 5.29 |
| 816 | 2-Propanethiol | 40.21 | 44.40 | 45.50 | -5.29 |
| 817 | Octyl urea | 98.60 | 93.20 | 93.30 | 5.30 |
| 818 | Nonane | 99.20 | 94.10 | 93.90 | 5.30 |
| 819 | 1,3,6,8-Tetrachlorodibenzo-p-dioxin | 74.00 | 69.30 | 68.70 | 5.30 |
| 820 | Norethindrone biphenyl-4-carboxylate | 68.40 | 64.00 | 63.10 | 5.30 |
| 821 | Linoleic acid | 157.40 | 153.50 | 152.10 | 5.30 |
| 822 | 4-Benzhydryl-5,6-dimethyl-2-(1'-naphthyl)-5-phenyl-1,3-dioxa-4-aza-2-boracyclohexane | 56.10 | 60.10 | 61.40 | -5.30 |
| 823 | Felodipine | 75.50 | 82.70 | 80.80 | -5.30 |
| 824 | Dicumyl | 65.11 | 58.80 | 59.80 | 5.31 |
| 825 | 1-Morpholinyl-3,5-bis(dimethylamino)-s-triazine | 62.13 | 57.50 | 56.80 | 5.33 |
| 826 | Isopropylnitrate | 52.43 | 47.90 | 47.10 | 5.33 |
| 827 | 2,3,4,5-Tetrachlorobiphenyl | 69.25 | 64.10 | 63.90 | 5.35 |
| 828 | 2-Oxoglutaric acid | 73.55 | 67.70 | 68.20 | 5.35 |
| 829 | Mefluidide | 82.35 | 77.00 |  | 5.35 |
| 830 | N-Acetylpyrazinamide | 64.36 | 58.80 | 59.00 | 5.36 |
| 831 | 1-Chloro-2-[2,2-dichloro-1-(4-chlorophenyl)vinyl]benzene | 68.17 | 63.50 | 62.80 | 5.37 |
| 832 | Methyl 2-(2,4,5-trichlorophenoxy)acetate | 84.18 | 79.30 | 78.80 | 5.38 |
| 833 | Endrin | 50.62 | 53.10 | 56.00 | -5.38 |
| 834 | Butanedioic acid | 72.09 | 66.20 | 66.70 | 5.39 |
| 835 | Cyclononanone | 64.90 | 69.40 | 70.30 | -5.40 |
| 836 | n-Butyl methyl ether | 68.90 | 74.20 | 74.30 | -5.40 |
| 837 | Ametryn | 55.00 | 59.20 | 60.40 | -5.40 |
| 838 | Ethyl N-benzoyl-N-(3,4-dichlorophenyl)-L-alaninate | 79.19 | 84.40 | 84.60 | -5.41 |
| 839 | s-trans-1,3-Butadiene | 48.62 | 47.00 | 43.20 | 5.42 |
| 840 | MTBE | 46.18 | 51.20 | 51.60 | -5.42 |
| 841 | p-Cresol | 41.26 | 46.90 | 46.70 | -5.44 |
| 842 | 2-Nitrophenol | 54.86 | 59.60 | 60.30 | -5.44 |
| 843 | Cyclopentane | 45.96 | 51.90 | 51.40 | -5.44 |
| 844 | 1,2,3,5-Tetrafluorobenzene | 47.01 | 53.20 | 52.50 | -5.49 |
| 845 | Pentyl urea | 63.01 | 67.90 | 68.50 | -5.49 |
| 846 | 1-Bromoundecane | 127.10 | 120.90 | 121.60 | 5.50 |
| 847 | Acomplia | 84.40 | 78.90 |  | 5.50 |
| 848 | 2,4-Dibromoaniline | 60.80 | 55.80 | 55.30 | 5.50 |
| 849 | Barbital | 54.00 | 58.80 | 59.50 | -5.50 |
| 850 | Barbituric acid | 42.20 | 46.80 | 47.70 | -5.50 |
| 851 | Chloroethylene | 41.20 | 46.70 |  | -5.50 |
| 852 | 4-(Bis(2-hydroxyethyl)aminoazobenzene | 73.61 | 68.30 | 68.10 | 5.51 |
| 853 | Heptachlor epoxide | 55.62 | 51.80 | 50.10 | 5.52 |
| 854 | Tetrahydrodicyclopentadiene | 26.17 | 33.30 | 31.70 | -5.53 |
| 855 | m-Terphenyl | 62.76 | 56.80 | 57.20 | 5.56 |
| 856 | p-Bromoacetanilide | 58.48 | 53.00 | 52.90 | 5.58 |
| 857 | Pentafluorobenzene | 48.22 | 54.90 | 53.80 | -5.58 |
| 858 | Rotenone | 81.39 | 75.90 | 75.80 | 5.59 |
| 859 | Isopropyl methyl sulfide | 54.50 | 47.60 | 48.90 | 5.60 |
| 860 | Nicotinic acid | 55.90 | 50.50 | 50.30 | 5.60 |
| 861 | 1,4-Dithiane | 56.20 | 51.50 | 50.60 | 5.60 |
| 862 | 2-(Perfluorooctyl)ethyl methacrylate | 59.40 | 64.50 | 65.00 | -5.60 |
| 863 | 3-Methylheptane | 76.60 | 71.30 | 71.00 | 5.60 |
| 864 | Ethylcyclohexane | 52.60 | 57.90 | 58.20 | -5.60 |
| 865 | Adiponitrile | 65.50 | 70.30 | 71.10 | -5.60 |
| 866 | Norfloxacin | 65.91 | 58.10 | 60.30 | 5.61 |
| 867 | 1-Methoxydecane | 130.31 | 125.00 | 124.70 | 5.61 |
| 868 | (2-Furyl)oxoacetamide | 48.47 | 54.10 | 54.10 | -5.63 |
| 869 | 2,6-Dinitrophenol | 58.26 | 62.80 | 63.90 | -5.64 |
| 870 | 1,2,3,4-Tetrafluorobenzene | 46.85 | 53.20 | 52.50 | -5.65 |
| 871 | 4-Ethoxyphenylacetic acid | 63.85 | 69.90 | 69.50 | -5.65 |
| 872 | 4-Methoxy-2-nitrophenol | 63.64 | 68.60 | 69.30 | -5.66 |
| 873 | 7-Oxabicyclo[4.1.0]heptane | 53.86 | 47.80 | 48.20 | 5.66 |
| 874 | Tetradecanoic acid | 137.92 | 143.60 | 143.60 | -5.68 |
| 875 | 2-Methylthiolane | 51.49 | 44.80 | 45.80 | 5.69 |
| 876 | Diphenylamine | 54.81 | 60.80 | 60.50 | -5.69 |
| 877 | Profluralin | 73.61 | 77.00 | 79.30 | -5.69 |
| 878 | 3-Nitrobenzonitrile | 52.60 | 47.20 | 46.90 | 5.70 |
| 879 | Dibenzofuran | 54.00 | 49.20 | 48.30 | 5.70 |
| 880 | 3-Bromo-N-(4-methoxyphenyl)benzenecarbothioamide | 60.60 | 66.80 | 66.30 | -5.70 |
| 881 | Glutaric acid | 69.10 | 74.60 | 74.80 | -5.70 |
| 882 | Diflunisal | 73.70 | 68.90 | 68.00 | 5.70 |
| 883 | 2-(Biphen-3-yl)naphthalene | 53.40 | 58.70 | 59.10 | -5.70 |
| 884 | 2-Picoline | 47.10 | 52.80 | 52.80 | -5.70 |
| 885 | N-(4-Chlorophenyl)-4H-3,1-benzothiazin-2-amine | 57.70 | 62.20 | 63.40 | -5.70 |
| 886 | 2,2'-Bis(trichlorosilyl)biphenyl | 61.09 | 63.40 | 66.80 | -5.71 |
| 887 | 1,2,3-Trichloronaphthalene | 51.99 | 57.80 | 57.70 | -5.71 |
| 888 | Isopropyl t-butyl ether | 45.78 | 51.20 | 51.50 | -5.72 |
| 889 | N-Theonylthiocarbamic-O-propyl ester | 70.52 | 65.40 | 64.80 | 5.72 |
| 890 | Isocinnamic acid | 49.68 | 55.40 | 55.40 | -5.72 |
| 891 | Pyrazon | 55.83 | 52.90 | 50.10 | 5.73 |
| 892 | 3-Methylbenzoic acid | 41.17 | 47.20 | 46.90 | -5.73 |
| 893 | 1,2-Dichloroacenaphthene | 60.46 | 54.80 | 54.70 | 5.76 |
| 894 | 1,2-Dibromotetrafluoroethane | 43.24 | 49.00 |  | -5.76 |
| 895 | 1,2,3,4-Tetrachlorobenzene | 53.14 | 59.00 | 58.90 | -5.76 |
| 896 | trans-Cyclooctene | 58.53 | 64.70 | 64.30 | -5.77 |
| 897 | 2-Phenylnaphthalene | 47.93 | 53.60 | 53.70 | -5.77 |
| 898 | 2-Naphthyl acetate | 58.59 | 53.30 | 52.80 | 5.79 |
| 899 | 2-Pentyl-6-octyldecalin | 138.50 | 132.70 | 132.70 | 5.80 |
| 900 | Decamethylcyclopentasiloxane | 90.10 | 86.60 | 84.30 | 5.80 |
| 901 | 5-Butyl-1,3-dimethyluracil | 70.50 | 65.00 | 64.70 | 5.80 |
| 902 | Estrone propionate | 56.20 | 61.70 | 62.00 | -5.80 |
| 903 | 2-Pyridinealdoxime | 51.50 | 56.30 | 57.30 | -5.80 |
| 904 | Indometacin | 77.30 | 82.80 | 83.10 | -5.80 |
| 905 | Mandelic acid | 64.92 | 59.10 | 59.10 | 5.82 |
| 906 | 4-Hexyloxy-4'-trifluoromethyldiphenyldiacetylene | 86.07 | 92.60 | 91.90 | -5.83 |
| 907 | 1,2,5-Tris(methoxycarbonyl)naphthalene | 70.25 | 76.20 | 76.10 | -5.85 |
| 908 | Trimethylamine | 41.93 | 46.00 | 47.80 | -5.87 |
| 909 | 3-Nitrotoluene | 51.88 | 46.40 | 46.00 | 5.88 |
| 910 | Salithion | 51.61 | 57.20 | 57.50 | -5.89 |
| 911 | 1,2,4-Triazole | 40.91 | 44.00 | 46.80 | -5.89 |
| 912 | 2-Oxa-6-thiaadamantane | 32.91 | 38.00 | 38.80 | -5.89 |
| 913 | trans-3-Octadecenoic acid | 171.10 | 165.50 | 165.20 | 5.90 |
| 914 | Caffeine | 52.30 | 50.00 | 46.40 | 5.90 |
| 915 | 1,2-Dinitrobenzene | 58.30 | 52.80 | 52.40 | 5.90 |
| 916 | 1,8-Bis(4-biphenyl)octane | 134.90 | 129.60 | 129.00 | 5.90 |
| 917 | Benzyldimethylvinylsilane | 56.70 | 62.60 |  | -5.90 |
| 918 | 2,2'-Dihydroxybenzophenone | 60.00 | 66.80 | 65.90 | -5.90 |
| 919 | N-Salicylidene-beta-alanine | 69.85 | 75.60 | 75.80 | -5.95 |
| 920 | 3-Methyl-2-butanethiol | 52.05 | 47.00 | 46.10 | 5.95 |
| 921 | 2-Hydroxybiphenyl | 49.04 | 55.30 | 55.00 | -5.96 |
| 922 | 2,5-Bis(biphenyl-4-yl)thiophene | 65.52 | 75.70 | 71.50 | -5.98 |
| 923 | 1-Naphthaleneacetic acid | 54.92 | 60.90 | 60.90 | -5.98 |
| 924 | Nitrobenzene | 43.51 | 49.70 | 49.50 | -5.99 |
| 925 | 2,6-Dibromoaniline | 61.30 | 55.80 | 55.30 | 6.00 |
| 926 | 2,2,4-Trimethyl-1,3-pentanediol | 73.70 | 66.60 | 67.70 | 6.00 |
| 927 | 1,2-Diiodobenzene | 47.50 | 54.00 | 53.50 | -6.00 |
| 928 | 1,2,3,5,8-Pentachloronaphthalene | 57.80 | 64.00 | 63.80 | -6.00 |
| 929 | Ambroxol | 99.90 | 104.20 | 105.90 | -6.00 |
| 930 | 3-Hydroxy-3-phenylbutyric acid | 63.29 | 69.80 | 69.30 | -6.01 |
| 931 | 2-(p-Terphenyl-4-yl)-5-phenyloxazole | 76.26 | 69.80 | 70.20 | 6.06 |
| 932 | 3-(4-Fluorophenyl)-3-hydroxypropanoic acid | 81.26 | 75.50 | 75.20 | 6.06 |
| 933 | Dicamba | 59.23 | 65.60 | 65.30 | -6.07 |
| 934 | 4-octadecynoic acid | 166.50 | 159.50 | 160.40 | 6.10 |
| 935 | 2-Pyrrolidone | 46.50 | 40.80 | 40.40 | 6.10 |
| 936 | 3-Nitroaniline | 61.30 | 54.60 | 55.20 | 6.10 |
| 937 | 4-Bromobenzoic acid | 58.70 | 52.60 | 52.60 | 6.10 |
| 938 | Caprolactone | 50.90 | 57.30 | 57.00 | -6.10 |
| 939 | trans-5-Hydroxymethyl-5,6-dihydro-7H-dibenzo[a,c]cycloheptan-6-ol | 69.10 | 62.80 | 63.00 | 6.10 |
| 940 | 2,4-Dichlorophenoxyacetic acid | 76.90 | 70.90 | 70.80 | 6.10 |
| 941 | 4-(5-Hexenyloxy)-3',4'-difluorodiphenyldiacetylene | 101.22 | 95.30 | 95.10 | 6.12 |
| 942 | Propanenitrile | 37.58 | 44.10 | 43.70 | -6.12 |
| 943 | Benzoic anhydride | 54.77 | 60.00 | 60.90 | -6.13 |
| 944 | Fluoranthene | 48.85 | 43.30 | 42.70 | 6.15 |
| 945 | Methyl methacrylate | 59.65 | 53.20 | 53.50 | 6.15 |
| 946 | 4-Bromo-2,5-dichlorophenol | 64.39 | 58.60 | 58.20 | 6.19 |
| 947 | 2-Methylbenzoic acid | 53.50 | 47.20 | 47.30 | 6.20 |
| 948 | Perfluoropentane | 46.00 | 37.60 | 39.80 | 6.20 |
| 949 | Acetonitrile | 29.20 | 35.70 | 35.40 | -6.20 |
| 950 | trans-3-Methoxycinnamic acid | 57.80 | 64.30 | 64.00 | -6.20 |
| 951 | Trimethylurea | 41.50 | 47.60 | 47.70 | -6.20 |
| 952 | Isopropylurea | 40.70 | 47.60 | 46.90 | -6.20 |
| 953 | Malonic acid | 56.70 | 64.00 | 62.90 | -6.20 |
| 954 | 2-Methoxy-4'-pentyloxy-trans-stilbene | 94.60 | 101.90 | 100.80 | -6.20 |
| 955 | Tamoxifen | 91.60 | 96.00 | 97.80 | -6.20 |
| 956 | Methylbenzene | 37.15 | 43.30 | 43.40 | -6.25 |
| 957 | n-Butylurea | 64.37 | 59.40 | 58.10 | 6.27 |
| 958 | Hexamethyldisiloxane | 58.18 | 51.60 | 51.90 | 6.28 |
| 959 | 1,4-Pentadiene | 49.41 | 55.50 | 55.70 | -6.29 |
| 960 | 3,3-Dimethylpentanedioic anhydride | 45.41 | 51.10 | 51.70 | -6.29 |
| 961 | 2-Chloronitrobenzene | 59.20 | 52.80 | 52.90 | 6.30 |
| 962 | 3-Chloronitrobenzene | 58.70 | 52.80 | 52.40 | 6.30 |
| 963 | Niclosamide | 71.60 | 78.50 | 77.90 | -6.30 |
| 964 | 2-(1'-Hydroxycyclohexyl)cyclohexanone | 67.80 | 74.20 | 74.10 | -6.30 |
| 965 | N,N'-Bis(salicylaldehydo)tetramethylenediimine | 98.00 | 102.00 | 104.30 | -6.30 |
| 966 | Propylene | 34.18 | 41.00 | 40.50 | -6.32 |
| 967 | Benzil | 61.65 | 54.80 | 55.30 | 6.35 |
| 968 | 1-Naphthylamine | 48.05 | 53.40 | 54.40 | -6.35 |
| 969 | 1,5-Cyclooctadiene | 58.03 | 66.00 | 64.40 | -6.37 |
| 970 | Aldicarb | 60.73 | 62.50 | 67.10 | -6.37 |
| 971 | Carbaryl | 58.88 | 51.90 | 52.50 | 6.38 |
| 972 | Quinoxaline | 38.61 | 44.10 | 45.00 | -6.39 |
| 973 | 2,10-Dimethylacridin-9(10H)-one | 52.60 | 47.10 | 46.20 | 6.40 |
| 974 | 1,1,1-Trinitroethane | 50.40 | 44.70 | 44.00 | 6.40 |
| 975 | 2-Bromobenzoic acid | 58.90 | 52.60 | 52.50 | 6.40 |
| 976 | 3,5-Dichlorobenzoic acid | 50.10 | 56.70 | 56.50 | -6.40 |
| 977 | Propylcyclohexane | 59.60 | 66.40 | 66.00 | -6.40 |
| 978 | Tartaric acid | 72.60 | 79.10 | 79.00 | -6.40 |
| 979 | 3-Bromoiodobenzene | 46.20 | 52.40 | 52.60 | -6.40 |
| 980 | N-Methyl-N-nitro-4-(phenylmethyl)benzenamine | 65.80 | 71.30 | 72.20 | -6.40 |
| 981 | Valeramide | 59.70 | 66.90 | 66.10 | -6.40 |
| 982 | Herban | 49.81 | 43.50 | 43.40 | 6.41 |
| 983 | 1,2,3,4,5,6,7,8-Octahydroanthracene | 60.69 | 67.10 | 67.10 | -6.41 |
| 984 | 3-Methylpentane | 48.17 | 54.40 | 54.60 | -6.43 |
| 985 | 4-Chloro-2'-hydroxy-4'-ethoxyazobenzene | 81.47 | 86.00 | 87.90 | -6.43 |
| 986 | 3,4-Dihydro-2H-1-benzopyran | 60.24 | 54.10 | 53.80 | 6.44 |
| 987 | Furfuranol | 50.75 | 56.50 | 57.20 | -6.45 |
| 988 | Phenothiazine | 58.75 | 64.80 | 65.20 | -6.45 |
| 989 | Chloroxuron | 81.88 | 75.00 | 75.40 | 6.48 |
| 990 | 1,2-Bis(chloromethyl)benzene | 64.78 | 57.60 | 58.30 | 6.48 |
| 991 | Diisopropyl sulfide | 53.39 | 48.20 | 46.90 | 6.49 |
| 992 | 1-Decene | 106.80 | 100.20 | 100.30 | 6.50 |
| 993 | Bis(4-cyanatophenyl)dimethyl silane | 82.10 | 76.70 | 75.60 | 6.50 |
| 994 | 2-(4-Nitrophenyl)-1-(4-(4-ethylcyclohexyl)phenyl)ethanone | 83.90 | 78.60 | 77.40 | 6.50 |
| 995 | 2,4-Dinitrophenol | 62.30 | 56.50 | 55.80 | 6.50 |
| 996 | 4-Pentoxy-4'-fluorodiphenylacetylene | 82.20 | 88.60 | 88.70 | -6.50 |
| 997 | Suberonitrile | 81.70 | 87.20 | 88.20 | -6.50 |
| 998 | Benzo[e]pyrene | 42.30 | 49.20 | 48.80 | -6.50 |
| 999 | 1,9-Cyclohexadecanedione bis(ethylene ketal) | 104.24 | 101.60 | 97.70 | 6.54 |
| 1000 | 1,3,7-Trichlorodibenzo-p-dioxin | 73.04 | 66.20 | 66.50 | 6.54 |
| 1001 | 3,4-Dinitrophenol | 62.35 | 56.50 | 55.80 | 6.55 |
| 1002 | 1,1,1,3-Tetrachloropropane | 54.16 | 51.20 | 47.60 | 6.56 |
| 1003 | 2,4-Dichlorophenol | 63.18 | 56.40 | 56.60 | 6.58 |
| 1004 | 3'-(Nitrooxymethyl)phenyl 2-acetoxybenzoate | 81.70 | 76.20 | 75.10 | 6.60 |
| 1005 | Amitraz | 56.50 | 51.00 | 49.90 | 6.60 |
| 1006 | Hexyl phenylcarbamate | 100.00 | 93.20 | 93.40 | 6.60 |
| 1007 | N-Vinylpyrrolidone | 53.40 | 46.70 | 46.80 | 6.60 |
| 1008 | Morphine | 54.70 | 48.50 | 48.10 | 6.60 |
| 1009 | 51-Crown-17 | 221.10 | 220.00 | 214.50 | 6.60 |
| 1010 | 4-Methoxycinnamaldehyde | 57.20 | 63.60 | 63.80 | -6.60 |
| 1011 | 4-Chlorobenzoic acid | 60.31 | 53.60 | 53.70 | 6.61 |
| 1012 | Thiane | 46.19 | 53.40 | 52.80 | -6.61 |
| 1013 | 2-Hexanone | 68.42 | 61.90 | 61.80 | 6.62 |
| 1014 | 4-Butoxy-4'-fluorodiphenylacetylene | 73.26 | 80.20 | 79.90 | -6.64 |
| 1015 | 1,1-Dimethylsilacyclobutane | 43.51 | 50.30 | 50.20 | -6.69 |
| 1016 | Dibutyl succinate | 119.60 | 114.60 | 112.90 | 6.70 |
| 1017 | Hydrocortisone hemisuccinate | 93.10 | 87.50 | 86.40 | 6.70 |
| 1018 | Ethyl 4-hydroxybenzoate | 67.90 | 61.40 | 61.20 | 6.70 |
| 1019 | Decyl urea | 103.80 | 110.10 | 110.50 | -6.70 |
| 1020 | 2,5-Dipentoxy-1,4-benzoquinone | 115.00 | 122.50 | 121.70 | -6.70 |
| 1021 | 2-Aminophenol | 48.50 | 55.10 | 55.20 | -6.70 |
| 1022 | 1-Naphthalenecarboxylic acid | 45.70 | 52.40 | 52.40 | -6.70 |
| 1023 | Testosterone butyrate | 66.20 | 72.00 | 72.90 | -6.70 |
| 1024 | Butylamide deferoxamine | 247.00 | 254.90 | 253.70 | -6.70 |
| 1025 | Cyclopropane | 37.39 | 44.90 | 44.10 | -6.71 |
| 1026 | Isocumene | 53.39 | 60.20 | 60.10 | -6.71 |
| 1027 | 1,4-Bis(methoxycarbonyl)naphthalene | 59.96 | 66.90 | 66.70 | -6.74 |
| 1028 | 3-Nitrobenzoic acid | 46.66 | 53.60 | 53.40 | -6.74 |
| 1029 | Coumarin | 55.95 | 49.20 | 49.20 | 6.75 |
| 1030 | Phthalonitrile | 48.26 | 41.60 | 41.50 | 6.76 |
| 1031 | 2-(2-Chlorophenoxy)propanoic acid | 72.57 | 65.80 | 65.80 | 6.77 |
| 1032 | Perhydrophenanthrene | 41.81 | 47.60 | 48.60 | -6.79 |
| 1033 | 1-Chloro-2,4-dinitrobenzene | 62.00 | 55.90 | 55.20 | 6.80 |
| 1034 | 4-Benzoyl-1-naphthol | 65.00 | 58.80 | 58.20 | 6.80 |
| 1035 | 6,7,8,9-Tetrahydro-1,6,6-trimethylphenanthro[1,2-b]furan-10,11-dione | 60.00 | 55.20 | 53.20 | 6.80 |
| 1036 | 1,4-Benzenedicarbonitrile | 48.30 | 41.60 | 41.50 | 6.80 |
| 1037 | N,N-Dimethylformamide | 41.80 | 35.00 |  | 6.80 |
| 1038 | Heneicosane | 202.80 | 195.70 | 196.00 | 6.80 |
| 1039 | Ethyl tridecanoate | 149.40 | 142.50 | 142.60 | 6.80 |
| 1040 | Pentacosofluoro-14-methyltricosane | 106.60 | 114.10 | 113.40 | -6.80 |
| 1041 | 2-Benzoyl-1-naphthol | 58.68 | 65.10 | 65.50 | -6.82 |
| 1042 | 2,4-Dimethylpyrrole | 35.75 | 42.90 | 42.60 | -6.85 |
| 1043 | 2,4,7-Trinitrofluorenone | 59.06 | 52.30 | 52.20 | 6.86 |
| 1044 | 6,6,14,14-Tetramethyl-1,3,9,11-tetraoxacyclohexadecane | 82.84 | 85.50 | 89.70 | -6.86 |
| 1045 | 1,4-Dihydroxybenzene | 60.67 | 53.80 | 53.80 | 6.87 |
| 1046 | Dibenzo[c,e][1,2]dithiine | 49.97 | 43.10 |  | 6.87 |
| 1047 | N-Hexadecylglycine | 170.73 | 177.10 | 177.60 | -6.87 |
| 1048 | N-(2-Chlorophenyl)thiourea | 53.01 | 59.90 | 59.90 | -6.89 |
| 1049 | Caproylamide deferoxamine | 264.80 | 271.80 | 271.70 | -6.90 |
| 1050 | Butyl 4-aminobenzoate | 72.30 | 79.60 | 79.20 | -6.90 |
| 1051 | 2-(4-Biphenylyl)naphthalene | 51.30 | 58.70 | 58.20 | -6.90 |
| 1052 | 2-Benzoyloxynaphthalene | 68.81 | 61.60 | 61.90 | 6.91 |
| 1053 | Bullvalene | 41.61 | 36.80 | 34.70 | 6.91 |
| 1054 | cis-1,4-Dimethylcyclohexane | 50.11 | 43.50 | 43.20 | 6.91 |
| 1055 | 4-Methoxyphenylacetic acid | 60.88 | 67.90 | 67.80 | -6.92 |
| 1056 | 2,4-Di-t-butylthiazole | 40.67 | 46.00 | 47.60 | -6.93 |
| 1057 | 3-Tetradecyloxy-1,2-propanediol | 187.44 | 180.10 | 180.50 | 6.94 |
| 1058 | 4-Methylcyclohexene | 43.16 | 50.70 | 50.10 | -6.94 |
| 1059 | Pivalaldehyde | 38.86 | 44.90 | 45.80 | -6.94 |
| 1060 | 1,2,3-Benzenetriol | 63.86 | 57.50 | 56.90 | 6.96 |
| 1061 | Benzaldehyde | 43.14 | 49.70 | 50.10 | -6.96 |
| 1062 | Trimethylhydrazine | 47.13 | 54.10 |  | -6.97 |
| 1063 | 2,2,2-Trinitroethyl 4,4,4-trinitrobutyrate | 89.79 | 85.80 | 82.80 | 6.99 |
| 1064 | 2,5-Dipropoxy-1,4-benzoquinone | 97.00 | 88.70 | 90.00 | 7.00 |
| 1065 | 2,5-Furandione | 37.60 | 44.50 | 44.60 | -7.00 |
| 1066 | 3-Thiopropionic acid | 58.20 | 65.10 | 65.20 | -7.00 |
| 1067 | Pivalonitrile | 31.80 | 39.20 | 38.80 | -7.00 |
| 1068 | Pentyl butyrate | 99.81 | 91.70 | 92.80 | 7.01 |
| 1069 | p-Toluidine | 54.81 | 48.20 | 47.80 | 7.01 |
| 1070 | N-Dodecyl-L-alanine | 105.59 | 116.20 | 112.60 | -7.01 |
| 1071 | Benzene hexa-n-hexanoate | 286.32 | 278.30 | 279.30 | 7.02 |
| 1072 | 1-Propylamine | 58.26 | 65.70 | 65.30 | -7.04 |
| 1073 | Cyclohexene | 49.85 | 56.60 | 56.90 | -7.05 |
| 1074 | N,N-Bis(3-methylphenyl)-N-(4-methylphenyl)amine | 72.76 | 63.00 | 65.70 | 7.06 |
| 1075 | trans-3-Phenyl-2-propen-1-ol | 51.04 | 58.80 | 58.10 | -7.06 |
| 1076 | 2-(4-Bromophenoxy)propanoic acid | 72.67 | 64.80 | 65.60 | 7.07 |
| 1077 | 1,1,1,5,5,5-Hexamethyl-3,3-diphenyltrisiloxane | 84.12 | 88.50 | 91.20 | -7.08 |
| 1078 | Methyl 3-((hydroxyimino)(3,4-dimethoxyphenyl)methyl)-1,2,2-trimethylcyclopentanecarboxylate | 92.10 | 82.30 | 85.00 | 7.10 |
| 1079 | 4-Formylbenzoic acid | 47.10 | 53.60 | 54.20 | -7.10 |
| 1080 | Dimethylmaleate | 58.00 | 64.80 | 65.10 | -7.10 |
| 1081 | Erythritol | 80.43 | 73.40 | 73.30 | 7.13 |
| 1082 | Margaric acid | 176.14 | 169.00 | 169.00 | 7.14 |
| 1083 | Benzo[b]triphenylene | 46.65 | 54.30 | 53.80 | -7.15 |
| 1084 | Buthidazole | 62.26 | 53.60 | 55.10 | 7.16 |
| 1085 | 7-Octadecynoic acid | 166.50 | 159.50 | 159.30 | 7.20 |
| 1086 | 1,2-Bis(methylsufinyl)ethane | 76.80 | 69.60 |  | 7.20 |
| 1087 | Vitavax | 77.20 | 70.40 | 70.00 | 7.20 |
| 1088 | N-Methyl-N-(4-biphenylyl)nitramine | 57.80 | 62.80 | 65.00 | -7.20 |
| 1089 | 4-Chloronitrobenzene | 45.40 | 52.80 | 52.60 | -7.20 |
| 1090 | Sulphadiazine | 58.00 | 66.00 | 65.20 | -7.20 |
| 1091 | 2-Hydroxy-4-methoxybenzophenone | 64.70 | 72.10 | 71.90 | -7.20 |
| 1092 | 6-Heptadecanol | 155.20 | 162.00 | 162.40 | -7.20 |
| 1093 | 1-Chloro-2,2-bis(4-chlorophenyl)ethylene | 75.53 | 68.30 |  | 7.23 |
| 1094 | 3-(3-Bromophenyl)-3-hydroxypropanoic acid | 68.14 | 76.00 | 75.40 | -7.26 |
| 1095 | Morniflumate | 98.57 | 92.70 | 91.30 | 7.27 |
| 1096 | 2-Methyl-2-nitropropyl 4,4,4-trinitrobutyrate | 86.41 | 92.30 | 93.70 | -7.29 |
| 1097 | 2,3:7,8-Dibenzocycloocta-2,7-dien-1-one | 66.20 | 59.50 | 58.90 | 7.30 |
| 1098 | trans-2-Methoxycinnamic acid | 70.90 | 64.30 | 63.60 | 7.30 |
| 1099 | Tetramethylurea | 49.20 | 56.00 | 56.50 | -7.30 |
| 1100 | 1-(Ethylmethylamino)-3,5-bis(dimethylamino)-s-triazine | 56.30 | 62.30 | 63.60 | -7.30 |
| 1101 | Cyclobutanone | 48.80 | 56.50 | 56.10 | -7.30 |
| 1102 | 2,2-Dimethylpropane | 31.10 | 38.50 | 38.40 | -7.30 |
| 1103 | 1,3-Benzodioxole-5-carboxylic acid | 60.81 | 53.10 | 53.50 | 7.31 |
| 1104 | Gloxazone | 80.39 | 79.70 | 87.70 | -7.31 |
| 1105 | Quinazoline | 52.82 | 45.50 |  | 7.32 |
| 1106 | 2-Chloronaphthalene | 44.28 | 51.60 | 51.60 | -7.32 |
| 1107 | cis-Decalin | 59.45 | 51.50 | 52.10 | 7.35 |
| 1108 | Tetrahydro-2H-pyran-2-one | 46.44 | 53.30 | 53.80 | -7.36 |
| 1109 | 2,3-Diazabicyclo(2,2,2)oct-2-ene N-oxide | 42.97 | 37.70 | 35.60 | 7.37 |
| 1110 | Propyl 4-aminobenzoate | 64.61 | 71.20 | 72.00 | -7.39 |
| 1111 | n-Decyl alcohol | 120.30 | 113.20 | 112.90 | 7.40 |
| 1112 | Tetraethylsilane | 68.70 | 60.70 | 61.30 | 7.40 |
| 1113 | 2,6-Diphenyl-1,4-dithiin | 62.10 | 65.40 | 69.50 | -7.40 |
| 1114 | Cycloheptene | 53.40 | 60.70 | 60.80 | -7.40 |
| 1115 | 2,8-Dichlorodibenzo-p-dioxin | 55.00 | 63.10 | 62.40 | -7.40 |
| 1116 | 6-Deoxy-D-glucopyranose | 55.50 | 61.80 | 62.90 | -7.40 |
| 1117 | t-Butyl propyl ether | 55.00 | 61.70 | 62.40 | -7.40 |
| 1118 | 2-Heptyl-6-butyldecalin | 107.70 | 115.80 | 115.10 | -7.40 |
| 1119 | Tetrachloro-o-xylene | 59.74 | 52.50 | 52.30 | 7.44 |
| 1120 | 2,3,6,7-Tetra(methoxycarbonyl)naphthalene | 91.88 | 85.40 | 84.40 | 7.48 |
| 1121 | Xanthone | 58.08 | 50.70 | 50.60 | 7.48 |
| 1122 | 1,3,3-Trinitroazetidine | 80.70 | 79.40 | 73.20 | 7.50 |
| 1123 | Diphenylsulfone | 54.70 | 47.20 | 47.20 | 7.50 |
| 1124 | 2-Chlorobenzoic acid | 61.00 | 53.60 | 53.50 | 7.50 |
| 1125 | Anthracene | 58.00 | 50.40 | 50.50 | 7.50 |
| 1126 | cis-3-Chloro-2-butenoic acid | 41.10 | 48.20 | 48.60 | -7.50 |
| 1127 | 3,3',4,4'-Tetrachlorobiphenyl | 56.60 | 64.10 | 64.10 | -7.50 |
| 1128 | Tridecanolactone | 92.55 | 85.60 | 85.00 | 7.55 |
| 1129 | 2,7-Dichlorofluorene | 48.84 | 56.20 | 56.40 | -7.56 |
| 1130 | 2-Methoxyisonitrosoacetanilide | 65.47 | 57.00 | 57.90 | 7.57 |
| 1131 | 1,3,7-Tris(methoxycarbonyl)naphthalene | 83.39 | 76.20 | 75.80 | 7.59 |
| 1132 | Mesitylenic acid | 51.00 | 44.00 | 43.40 | 7.60 |
| 1133 | beta-HCH | 72.00 | 66.90 | 64.40 | 7.60 |
| 1134 | Ferulic acid | 75.00 | 68.00 | 67.40 | 7.60 |
| 1135 | 1-(Dimethylamino)ethyl methacrylate | 70.90 | 63.00 | 63.30 | 7.60 |
| 1136 | 2,5-Dibromoaniline | 62.40 | 55.80 | 54.80 | 7.60 |
| 1137 | 1H-Indene-1,3(2H)-dione | 54.30 | 48.00 | 46.70 | 7.60 |
| 1138 | Sulfathiazole | 64.10 | 59.30 | 56.50 | 7.60 |
| 1139 | Phenazine | 46.47 | 41.50 | 38.80 | 7.67 |
| 1140 | Caprylene | 89.29 | 83.30 | 81.60 | 7.69 |
| 1141 | 2,5-Dimethylthiophene | 42.31 | 48.70 | 50.00 | -7.69 |
| 1142 | 2,5-Dihexadecyloxy-1,4-benzoquinone | 316.10 | 308.60 | 308.40 | 7.70 |
| 1143 | Bisphenol A | 69.60 | 62.50 | 61.90 | 7.70 |
| 1144 | 1,2,4,5-Tetraisopropylbenzene | 49.90 | 43.70 | 42.20 | 7.70 |
| 1145 | Norethindrone acetate | 56.90 | 50.30 | 49.20 | 7.70 |
| 1146 | 2-Nitrobenzoic acid | 66.80 | 59.90 | 59.10 | 7.70 |
| 1147 | 2,2-Dichloro-1,1,1-trifluoroethane | 37.80 | 43.50 | 45.50 | -7.70 |
| 1148 | 1-Heptene | 82.50 | 74.80 | 74.80 | 7.70 |
| 1149 | t-Butylbenzene | 39.10 | 46.90 | 46.80 | -7.70 |
| 1150 | Dibenzo[ae]pyrene | 58.63 | 51.10 | 50.90 | 7.73 |
| 1151 | Acenaphthene | 58.55 | 50.90 | 50.80 | 7.75 |
| 1152 | Diphenylmethanol | 67.95 | 60.30 | 60.20 | 7.75 |
| 1153 | 2,3,5,6-tetrachloro-p-xylene | 61.35 | 52.50 | 53.60 | 7.75 |
| 1154 | 1,6-Bis(methoxycarbonyl)naphthalene | 59.44 | 66.90 | 67.20 | -7.76 |
| 1155 | 8-Butyltheophylline | 63.43 | 70.60 | 71.20 | -7.77 |
| 1156 | Chlorobenzene | 41.93 | 49.60 | 49.70 | -7.77 |
| 1157 | 4-Propyl-4'-fluorodiphenylacetylene | 74.38 | 67.60 | 66.60 | 7.78 |
| 1158 | Octane | 93.19 | 85.70 | 85.40 | 7.79 |
| 1159 | Coumafuryl | 86.49 | 79.10 | 78.70 | 7.79 |
| 1160 | m-Anthranilic acid | 48.20 | 55.40 | 56.00 | -7.80 |
| 1161 | Dicyanomethane | 36.70 | 44.90 | 44.50 | -7.80 |
| 1162 | N-Theonylthiocarbamic-O-ethyl ester | 63.30 | 56.90 | 55.50 | 7.80 |
| 1163 | 1,3-Bis(propylsufinyl)propane | 98.70 | 106.50 |  | -7.80 |
| 1164 | 1,2,4,6,7-Pentachloronaphthalene | 55.90 | 64.00 | 63.70 | -7.80 |
| 1165 | Isoproturon | 49.90 | 58.30 | 57.70 | -7.80 |
| 1166 | Ritonavir | 146.40 | 148.70 | 154.20 | -7.80 |
| 1167 | 1,1,3,3,5,5-Hexamethyl-1,3,5-trisilacyclohexane | 61.42 | 53.60 |  | 7.82 |
| 1168 | 1,4-Bis(chloromethyl)benzene | 64.23 | 57.60 | 56.40 | 7.83 |
| 1169 | 1-Octanethiol | 108.35 | 100.30 | 100.50 | 7.85 |
| 1170 | 3-Methyl-1-butanethiol | 53.04 | 60.50 | 60.90 | -7.86 |
| 1171 | Piperonylonitrile | 56.80 | 46.70 | 48.90 | 7.90 |
| 1172 | Tridecane | 136.31 | 128.00 | 128.40 | 7.91 |
| 1173 | 2,5-Dimethylpyrrole | 33.09 | 39.20 | 41.00 | -7.91 |
| 1174 | Indeno[1,2,3-cd]pyrene | 49.41 | 42.00 | 41.50 | 7.91 |
| 1175 | 1,2-Diaceto-3-stearin | 218.72 | 211.70 | 210.80 | 7.92 |
| 1176 | N-Caproyl-pyrazinamide | 102.22 | 92.70 | 94.30 | 7.92 |
| 1177 | 9H-Fluoren-9-one | 50.84 | 42.90 | 42.90 | 7.94 |
| 1178 | Deoxycorticosterone acetate | 68.98 | 61.30 | 61.00 | 7.98 |
| 1179 | Undecane | 118.60 | 111.10 | 110.60 | 8.00 |
| 1180 | 2-Heptyl-6-hexyldecalin | 124.60 | 132.70 | 132.60 | -8.00 |
| 1181 | Ethyl 2-cyano-6-nitro-1(2H)-quinolinecarboxylate | 69.30 | 61.30 | 61.30 | 8.00 |
| 1182 | Contergan | 65.70 | 57.00 | 57.70 | 8.00 |
| 1183 | exo-Bicyclo[2.2.1]heptane-2-carbonitrile | 43.20 | 35.50 | 35.20 | 8.00 |
| 1184 | 2,5-Lutidine | 56.50 | 49.50 | 48.50 | 8.00 |
| 1185 | Dimethylacetamide | 40.10 | 47.80 | 48.10 | -8.00 |
| 1186 | Propyl Naltrexone-3-O-carbonate | 55.30 | 59.50 | 63.30 | -8.00 |
| 1187 | Ethyl 8-(hydroxymethyl)-6-bromo-2-oxo-2H-1-benzopyran-3-carboxylate | 64.90 | 72.90 | 72.90 | -8.00 |
| 1188 | N,N-Diethylurea | 59.51 | 51.90 | 51.50 | 8.01 |
| 1189 | 3,5-Lutidine | 49.12 | 40.10 | 41.10 | 8.02 |
| 1190 | Diethyl terephthalate | 77.82 | 69.00 | 69.80 | 8.02 |
| 1191 | 4-Cyanocyclohexyl trans-2-butenoate | 66.63 | 59.20 | 58.60 | 8.03 |
| 1192 | trans-1,3-Pentadiene | 38.47 | 45.90 | 46.50 | -8.03 |
| 1193 | 4-Hexyloxy-2',4'-difluorodiphenylacetylene | 106.26 | 98.70 | 98.20 | 8.06 |
| 1194 | 2-Methyl-1,2-propanediamine | 73.82 | 83.40 | 81.90 | -8.08 |
| 1195 | 3-Methyl-1-butene | 51.19 | 43.50 | 43.10 | 8.09 |
| 1196 | 4-Hexyloxybenzylideneaniline | 98.80 | 90.60 | 90.70 | 8.10 |
| 1197 | 4-Trifluoromethyl-7-aminocoumarin | 64.10 | 55.40 | 56.00 | 8.10 |
| 1198 | Chlordane | 56.40 | 61.30 | 64.50 | -8.10 |
| 1199 | Myclobutanil | 88.70 | 97.10 | 96.80 | -8.10 |
| 1200 | Dipropetryn | 63.39 | 70.10 | 71.50 | -8.11 |
| 1201 | 19-Octatriacontene | 320.07 | 327.50 | 328.20 | -8.13 |
| 1202 | 2,3-Dichlorophenol | 64.74 | 56.40 | 56.60 | 8.14 |
| 1203 | 5-Methylthiazole | 32.86 | 38.70 | 41.00 | -8.14 |
| 1204 | gamma-Butyrolactone | 41.56 | 49.60 | 49.70 | -8.14 |
| 1205 | Metribuzin | 45.06 | 51.90 | 53.20 | -8.14 |
| 1206 | 5-Methyltetrazole | 38.28 | 34.30 | 30.10 | 8.18 |
| 1207 | Ethyl nonadecanoate | 201.30 | 193.20 | 193.10 | 8.20 |
| 1208 | trans-13-Octadecenoic acid | 174.90 | 165.50 | 166.70 | 8.20 |
| 1209 | 2,3-Dichlorodibenzofuran | 63.00 | 55.50 | 54.80 | 8.20 |
| 1210 | (3-Chloro-4-methylphenyl)-(3-thia-1-aza-bicyclo[3.3.1]non-2-ylidene)-amine | 55.80 | 46.20 | 47.60 | 8.20 |
| 1211 | [2.2]Paracyclophane-1,9-diene | 60.70 | 51.20 | 52.50 | 8.20 |
| 1212 | N-Methyl-N-(4-methylsulfonylphenyl)nitramine | 52.30 | 58.20 | 60.50 | -8.20 |
| 1213 | 2,7-Dichlorodibenzo-p-dioxin | 55.00 | 63.10 | 63.20 | -8.20 |
| 1214 | 1,3-Bis(chloromethyl)benzene | 63.51 | 57.60 | 55.30 | 8.21 |
| 1215 | 3-(p-Terphenyl-4-yl)-5-phenylthiophene | 76.62 | 71.50 | 68.40 | 8.22 |
| 1216 | 4-Pentyl-4'-fluorodiphenylacetylene | 75.87 | 82.90 | 84.10 | -8.23 |
| 1217 | 3,4-Dinitrotoluene | 57.15 | 49.60 | 48.90 | 8.25 |
| 1218 | 4-(4-Heptyl-1-piperidinyl)benzonitrile | 88.95 | 97.40 | 97.20 | -8.25 |
| 1219 | Thiolane | 41.54 | 49.90 | 49.80 | -8.26 |
| 1220 | 1,3,7,8-Tetrachlorodibenzo-p-dioxin | 78.00 | 69.30 | 69.70 | 8.30 |
| 1221 | 2-Fluorofluorene | 60.00 | 51.60 | 51.70 | 8.30 |
| 1222 | Prehnitene | 42.31 | 33.50 | 34.00 | 8.31 |
| 1223 | Butylbenzene | 60.56 | 68.70 | 68.90 | -8.34 |
| 1224 | 1-Benzyoyl-1,2-dihydro-6-methoxy-2-quinolinecarbonitrile | 58.40 | 66.90 | 66.80 | -8.40 |
| 1225 | 2,2,8,8-Tetramethyl-1,3,7,9-tetraoxacyclododecane | 61.10 | 68.90 | 69.50 | -8.40 |
| 1226 | Butyl 2-cyano-6-nitro-1(2H)-quinolinecarboxylate | 70.10 | 78.20 | 78.50 | -8.40 |
| 1227 | Carbamazepine | 53.60 | 59.30 | 62.00 | -8.40 |
| 1228 | Urea | 36.40 | 43.40 | 44.80 | -8.40 |
| 1229 | 1-Bromopentane | 62.00 | 70.20 | 70.40 | -8.40 |
| 1230 | 2-Bromopropane | 35.50 | 43.90 |  | -8.40 |
| 1231 | (6Z)-Octadecenoic acid | 156.40 | 165.50 | 164.80 | -8.40 |
| 1232 | Propylenediamine | 77.89 | 84.70 | 86.30 | -8.41 |
| 1233 | 9-Methyl-9H-fluorene | 51.13 | 44.00 | 42.70 | 8.43 |
| 1234 | Monacolin K | 96.84 | 90.40 | 88.40 | 8.44 |
| 1235 | Methylpropylether | 57.25 | 65.70 | 65.70 | -8.45 |
| 1236 | Cyclotetradecane | 79.24 | 87.70 | 87.70 | -8.46 |
| 1237 | Pentanoic acid | 59.13 | 67.50 | 67.60 | -8.47 |
| 1238 | 2,4-Lutidine | 42.12 | 49.50 | 50.60 | -8.48 |
| 1239 | 5-Octadecynoic acid | 167.40 | 159.50 | 158.90 | 8.50 |
| 1240 | Ethyl 8-(hydroxymethyl)-6-methyl-2-oxo-2H-1-benzopyran-3-carboxylate | 75.90 | 67.50 | 67.40 | 8.50 |
| 1241 | exo-Dicyclopentadiene | 46.10 | 37.60 | 37.60 | 8.50 |
| 1242 | 2,3,8-Trichlorodibenzofuran | 67.00 | 58.60 | 58.50 | 8.50 |
| 1243 | 2,4,5-Trimethoxybenzoic acid | 74.50 | 83.60 | 83.00 | -8.50 |
| 1244 | 2,5-Diethoxy-1,4-benzoquinone | 62.50 | 71.70 | 71.00 | -8.50 |
| 1245 | 1,2,3,6,7-Pentachloronaphthalene | 55.10 | 64.00 | 63.60 | -8.50 |
| 1246 | Enantholactam | 44.40 | 52.60 | 52.90 | -8.50 |
| 1247 | 2,6-Dichlorophenol | 65.12 | 56.40 | 56.60 | 8.52 |
| 1248 | 1,5-Cyclooctanedione bis(ethylene ketal) | 60.88 | 69.30 | 69.40 | -8.52 |
| 1249 | DCPA | 70.01 | 77.40 | 78.60 | -8.59 |
| 1250 | 1,9-Nonanediamine | 143.40 | 138.80 | 134.80 | 8.60 |
| 1251 | 2-Methylacetanilide | 56.70 | 47.60 | 48.10 | 8.60 |
| 1252 | Permethric acid | 49.20 | 41.20 | 40.60 | 8.60 |
| 1253 | 1,6-Anhydro-beta-D-mannopyranose | 50.20 | 58.90 | 58.80 | -8.60 |
| 1254 | 1,14-Tetradecanedioic acid | 142.20 | 150.80 | 150.80 | -8.60 |
| 1255 | Fluoridamid | 88.81 | 80.20 |  | 8.61 |
| 1256 | 2-(4-Nitrophenoxy)propanoic acid | 57.79 | 65.80 | 66.40 | -8.61 |
| 1257 | Ovalene | 33.67 | 38.30 | 42.30 | -8.63 |
| 1258 | 1,4-Cyclohexanedione | 50.76 | 59.30 | 59.40 | -8.64 |
| 1259 | Perylene | 57.87 | 49.20 | 49.20 | 8.67 |
| 1260 | Tetramethylsuccinic acid | 49.02 | 56.40 | 57.70 | -8.68 |
| 1261 | Eicosamethyl-1,10-diphenyl-decasilane | 143.40 | 134.70 |  | 8.70 |
| 1262 | 2,4,6-Tribromoaniline | 65.50 | 58.00 | 56.80 | 8.70 |
| 1263 | 4-Chloro-3-nitrophenol | 65.00 | 56.40 | 56.30 | 8.70 |
| 1264 | 4-(4-Chlorophenyl)cyclohexyl trans-2-butenoate | 78.20 | 69.90 | 69.50 | 8.70 |
| 1265 | 3,5-Di-t-butylsalicylic acid | 52.40 | 61.10 | 61.10 | -8.70 |
| 1266 | Ethyl N-phenylcarbamate | 49.90 | 59.40 | 58.60 | -8.70 |
| 1267 | 1,2,7-Tris(methoxycarbonyl)naphthalene | 84.50 | 76.20 | 75.80 | 8.70 |
| 1268 | N,N,N',N'-Tetramethylmonothiooxamide | 48.50 | 57.20 | 57.20 | -8.70 |
| 1269 | 1,6-Dimethylnaphthalene | 33.08 | 42.00 | 41.80 | -8.72 |
| 1270 | 2,2,3,3-Tetramethylbutane | 33.27 | 42.10 | 42.00 | -8.73 |
| 1271 | DDT | 69.04 | 59.40 | 60.30 | 8.74 |
| 1272 | 3-Iodobenzoic acid | 62.34 | 54.20 | 53.60 | 8.74 |
| 1273 | Thiazole | 40.04 | 34.70 | 31.30 | 8.74 |
| 1274 | 1,3-Bis(bromomethyl)benzene | 67.65 | 59.80 | 58.90 | 8.75 |
| 1275 | Dimethyl isophthalate | 74.15 | 65.00 | 65.40 | 8.75 |
| 1276 | 2-Chloroaniline | 45.99 | 54.60 | 54.80 | -8.81 |
| 1277 | Fluchloralin | 72.49 | 81.70 | 81.30 | -8.81 |
| 1278 | Isoprene | 38.68 | 46.80 | 47.50 | -8.82 |
| 1279 | 4-Chlorophenol | 44.51 | 53.30 | 53.40 | -8.89 |
| 1280 | trans-14-Octadecenoic acid | 174.50 | 165.50 | 165.60 | 8.90 |
| 1281 | Bromoacetic acid | 43.50 | 52.00 | 52.40 | -8.90 |
| 1282 | Pimelamide | 99.73 | 90.50 | 90.80 | 8.93 |
| 1283 | Benzanilide | 67.84 | 59.20 | 58.90 | 8.94 |
| 1284 | Imidazole | 35.35 | 44.70 | 44.30 | -8.95 |
| 1285 | 2-(Diethylamino)ethyl methacrylate | 63.04 | 69.60 | 72.00 | -8.96 |
| 1286 | Cyanoacetamide | 59.46 | 50.80 | 50.50 | 8.96 |
| 1287 | Thianaphthene | 38.84 | 48.30 | 47.80 | -8.96 |
| 1288 | 4,5,9,10-Tetrahydropyrene | 47.53 | 56.10 | 56.50 | -8.97 |
| 1289 | 8-Pentyltheophylline | 70.43 | 79.10 | 79.40 | -8.97 |
| 1290 | Tolbutamide | 63.30 | 72.00 | 72.30 | -9.00 |
| 1291 | 4-Carboxyphenol | 63.10 | 54.10 | 54.10 | 9.00 |
| 1292 | 3-(5,6,7,8-Tetrahydro-2-naphthalenoyl)-1,2,2-trimethylcyclopentanecarboxylic acid | 54.50 | 63.00 | 63.50 | -9.00 |
| 1293 | Tetramethylene glycol | 63.70 | 73.10 | 72.70 | -9.00 |
| 1294 | Hexamethyleneglycol | 80.70 | 90.00 | 89.70 | -9.00 |
| 1295 | 9,10-Dimethylbenz[a]anthracene | 54.81 | 45.90 | 45.80 | 9.01 |
| 1296 | 3,5-Dinitrobenzoic acid | 47.47 | 56.70 | 56.50 | -9.03 |
| 1297 | 1,7-Cyclododecanedione bis(ethylene ketal) | 77.26 | 85.50 | 86.30 | -9.04 |
| 1298 | trans-1,4-Dimethylcyclohexane | 52.26 | 43.50 | 43.20 | 9.06 |
| 1299 | 2-Chlorophenol | 44.24 | 53.30 | 53.30 | -9.06 |
| 1300 | 1,2,8-Tris(methoxycarbonyl)naphthalene | 67.63 | 76.20 | 76.70 | -9.07 |
| 1301 | N,N,N',N'-Tetramethyloxamide | 51.11 | 60.70 | 60.20 | -9.09 |
| 1302 | Khellin | 75.80 | 67.90 | 66.70 | 9.10 |
| 1303 | Ethyl methyl carbonate | 51.20 | 40.90 | 42.10 | 9.10 |
| 1304 | 3-(4-Ethoxybenzoyl)-1,2,2-trimethylcyclopentanecarboxylic acid | 55.90 | 63.70 | 65.00 | -9.10 |
| 1305 | 1,3-Dithiane | 46.50 | 55.60 | 55.60 | -9.10 |
| 1306 | Menthol | 37.58 | 46.20 | 46.70 | -9.12 |
| 1307 | 4-(11-(1,1,3,3-Tetramethyldisiloxanyl)undecanoyloxy)phenyl 4-methoxybenzoate | 89.07 | 98.20 |  | -9.13 |
| 1308 | 1,2-Dibromoethane | 46.44 | 54.70 | 55.60 | -9.16 |
| 1309 | Dexamethasone | 77.97 | 68.50 | 68.80 | 9.17 |
| 1310 | Monalide | 64.71 | 74.40 | 73.90 | -9.19 |
| 1311 | Salol | 60.80 | 69.70 | 70.00 | -9.20 |
| 1312 | Sorbitol | 82.20 | 90.70 | 91.40 | -9.20 |
| 1313 | 7-Amino-4-methylcoumarin | 64.82 | 53.90 | 55.60 | 9.22 |
| 1314 | 4,4'-Dibutanoyloxydiphenyldiacetylene | 85.18 | 93.00 | 94.40 | -9.22 |
| 1315 | Methylcyclobutane | 41.56 | 50.80 | 50.80 | -9.24 |
| 1316 | m-Cresol | 37.53 | 46.90 | 46.80 | -9.27 |
| 1317 | Butralin | 61.52 | 70.70 | 70.80 | -9.28 |
| 1318 | Pentacosafluorohexadecane | 69.39 | 60.80 | 60.10 | 9.29 |
| 1319 | 2-Thiaadamantane | 22.81 | 31.20 | 32.10 | -9.29 |
| 1320 | Valeramide Deferoxamine | 271.40 | 263.40 | 262.10 | 9.30 |
| 1321 | trans-12-Octadecenoic acid | 174.50 | 165.50 | 165.20 | 9.30 |
| 1322 | 1,1-Diphenyldodecane | 148.20 | 138.80 | 138.90 | 9.30 |
| 1323 | Sulfapyridine | 87.50 | 80.00 | 78.20 | 9.30 |
| 1324 | Cyclodecanone | 82.30 | 73.50 | 73.00 | 9.30 |
| 1325 | L-Iditol | 87.60 | 97.00 | 96.90 | -9.30 |
| 1326 | 3-(4-(Dimethylamino)benzoyl)-1,2,2-trimethylcyclopentanecarboxylic acid | 56.30 | 64.00 | 65.60 | -9.30 |
| 1327 | 2,7-Diiodofluorene | 49.46 | 57.40 | 58.80 | -9.34 |
| 1328 | 2,11-Dicyclohexyldodecane | 146.15 | 136.70 | 136.80 | 9.35 |
| 1329 | Thianthrene | 64.13 | 70.00 | 73.50 | -9.37 |
| 1330 | trans-Diphenyldiazene N-oxide | 57.99 | 51.70 | 48.60 | 9.39 |
| 1331 | Tri-p-tolylamine | 51.31 | 63.00 | 60.70 | -9.39 |
| 1332 | (4-Cyanophenyl)-(3-thia-1-aza-bicyclo[3.3.1]non-2-ylidene)-amine | 52.00 | 43.90 | 42.60 | 9.40 |
| 1333 | 2-Propenyl 2-cyano-6-nitro-1(2H)-quinolinecarboxylate | 70.00 | 60.50 | 60.60 | 9.40 |
| 1334 | 4-Trichlorosilylbiphenyl | 49.80 | 57.50 | 59.20 | -9.40 |
| 1335 | Barban | 78.21 | 70.20 | 68.80 | 9.41 |
| 1336 | Altretamine | 51.78 | 61.20 | 61.20 | -9.42 |
| 1337 | Cyclohexane | 45.77 | 55.40 | 55.20 | -9.43 |
| 1338 | 4-(Dipropylamino)-N,N-dimethyl-3,5-dinitrobenzenesulfonamide | 78.75 | 88.20 |  | -9.45 |
| 1339 | Triptycene | 57.36 | 48.50 | 47.90 | 9.46 |
| 1340 | Ethyl-n-propylether | 57.64 | 67.70 | 67.10 | -9.46 |
| 1341 | t-Amyl mercaptan | 53.03 | 61.50 | 62.50 | -9.47 |
| 1342 | Famphur | 81.08 | 71.60 |  | 9.48 |
| 1343 | 2-(2,4-Dichlorophenoxy)propanoic acid | 78.18 | 68.90 | 68.70 | 9.48 |
| 1344 | Thioacetamide | 47.59 | 38.00 | 38.10 | 9.49 |
| 1345 | 2-Methylpropyl 2-cyano-6-nitro-1(2H)-quinolinecarboxylate | 72.70 | 63.80 | 63.20 | 9.50 |
| 1346 | 1,4-Dithiane sulfone | 54.80 | 60.20 | 64.30 | -9.50 |
| 1347 | Methyl nonadecanoate | 200.50 | 191.20 | 191.00 | 9.50 |
| 1348 | 9-Acetylcarbazole | 43.20 | 52.50 | 52.70 | -9.50 |
| 1349 | N-Methyl succinamic acid | 54.40 | 63.60 | 63.90 | -9.50 |
| 1350 | Phenyl 2-cyano-6-methoxy-1(2H)-quinolinecarboxylate | 59.60 | 69.00 | 69.10 | -9.50 |
| 1351 | Formamide deferoxamine | 215.70 | 225.20 |  | -9.50 |
| 1352 | 1-Tridecanol | 148.11 | 138.60 | 138.60 | 9.51 |
| 1353 | Pentamethylbenzene | 39.33 | 30.30 | 29.80 | 9.53 |
| 1354 | Thietane | 45.02 | 52.40 | 54.60 | -9.58 |
| 1355 | 19-Oxaheptatriacontane | 348.40 | 338.50 | 338.80 | 9.60 |
| 1356 | Decylcyclopentane | 132.00 | 122.10 | 122.40 | 9.60 |
| 1357 | 4-Benzhydryl-5,6-dimethyl-2,5-diphenyl-1,3-dioxa-4-aza-2-boracyclohexane | 65.90 | 58.10 | 56.30 | 9.60 |
| 1358 | Aspirin | 71.30 | 61.60 | 61.70 | 9.60 |
| 1359 | Butanamide | 49.60 | 58.50 | 59.20 | -9.60 |
| 1360 | 2-Ethylhexyl-alpha-D-glucoside | 107.30 | 116.30 | 116.90 | -9.60 |
| 1361 | Tricyclazole | 52.30 | 56.70 | 61.90 | -9.60 |
| 1362 | 4-Acetanisidine | 69.51 | 59.80 | 59.90 | 9.61 |
| 1363 | 2-(4-Nitrophenyl)-1-(4-(4-butylcyclohexyl)phenyl)ethanone | 85.27 | 95.60 | 94.90 | -9.63 |
| 1364 | Hexachlorophene | 76.01 | 86.10 | 85.70 | -9.69 |
| 1365 | 1-Hexanethiol | 93.51 | 83.30 | 83.80 | 9.71 |
| 1366 | N-(4-Ethoxybenzylidene)-4-butylaniline | 88.71 | 78.90 | 79.00 | 9.71 |
| 1367 | N-Methyl-diphenylacetamide | 68.73 | 58.70 | 59.00 | 9.73 |
| 1368 | Chlorophacinone | 82.94 | 72.90 | 73.20 | 9.74 |
| 1369 | 3-Undecylamino-1,2-propanediol | 166.86 | 158.40 | 157.10 | 9.76 |
| 1370 | Mecoprop | 72.16 | 62.50 | 62.40 | 9.76 |
| 1371 | N-Tetradecyl-L-alanine | 142.47 | 133.10 | 132.70 | 9.77 |
| 1372 | Desmedipham | 83.09 | 74.20 | 73.30 | 9.79 |
| 1373 | 1-Undecene | 118.20 | 108.60 | 108.40 | 9.80 |
| 1374 | Saccharin | 59.70 | 50.40 | 49.90 | 9.80 |
| 1375 | 1-Ethyladamantane | 50.00 | 40.90 | 40.20 | 9.80 |
| 1376 | Quinizarin Green SS | 74.50 | 66.50 | 64.70 | 9.80 |
| 1377 | Dibenzothiophene S,S-dioxide | 46.70 | 36.90 | 36.90 | 9.80 |
| 1378 | 3-Methylthiolane | 54.01 | 43.90 | 44.20 | 9.81 |
| 1379 | 1-Nonadecanoic acid | 195.93 | 185.90 | 186.10 | 9.83 |
| 1380 | Pelargonic acid | 92.05 | 101.30 | 101.90 | -9.85 |
| 1381 | Pentanedinitrile | 51.53 | 61.80 | 61.40 | -9.87 |
| 1382 | 11-Deoxycorticosterone | 67.59 | 57.40 | 57.70 | 9.89 |
| 1383 | 3-Amino-4-methylbenzoic acid | 61.30 | 52.10 | 51.40 | 9.90 |
| 1384 | 5-Ethyl-1,3-dimethyluracil | 54.80 | 48.10 | 44.90 | 9.90 |
| 1385 | Furfural | 61.10 | 52.30 | 51.20 | 9.90 |
| 1386 | N-(3-Methoxyphenylmethyl)-N'-(phenylmethyl)thiourea | 60.90 | 71.20 | 70.80 | -9.90 |
| 1387 | 2-(2-Benzofuryl)-2-imidazoline | 62.90 | 52.20 | 53.00 | 9.90 |
| 1388 | 1,10-Phenanthroline | 34.80 | 46.00 | 44.70 | -9.90 |
| 1389 | 5H-10,11-Dihydrodibenzo[a,d]cyclohexane-5-ol | 52.00 | 62.50 | 61.90 | -9.90 |
| 1390 | Hexadecanamide | 150.00 | 160.00 | 159.90 | -9.90 |
| 1391 | 1,2-Diphenylethane | 78.31 | 68.60 | 68.40 | 9.91 |
| 1392 | 2,4-Dinitrotoluene | 58.61 | 49.60 | 48.70 | 9.91 |
| 1393 | N-Methyl-N-(4-t-butylphenyl)nitramine | 66.63 | 58.10 | 56.70 | 9.93 |
| 1394 | Isopentene | 58.34 | 49.20 | 48.40 | 9.94 |
| 1395 | Phenylacetic acid | 48.95 | 58.90 | 58.90 | -9.95 |
| 1396 | 3-Phenyl-3-hydroxypropanoic acid | 83.47 | 73.90 | 73.50 | 9.97 |
| 1397 | Norethindrone 4-cyclohexylbenzoate | 80.08 | 70.10 | 70.10 | 9.98 |
| 1398 | 2-Nitro-1,1-bis(4-chlorophenyl)propane | 60.38 | 52.00 | 50.40 | 9.98 |
| 1399 | 3-(p-Terphenyl-4-yl)-4-phenylthiophene | 75.78 | 67.30 | 65.80 | 9.98 |
| 1400 | 1,4-Bis-(2-hydroxyethyl)piperazine | 64.00 | 54.70 | 54.00 | 10.00 |
| 1401 | n-Hexylacetate | 93.50 | 83.20 | 83.50 | 10.00 |
| 1402 | Penconazole | 81.00 | 89.90 | 91.00 | -10.00 |
| 1403 | 2-Chloro-5-(trichloromethyl)pyridine | 44.70 | 50.60 | 54.70 | -10.00 |
| 1404 | Pleconaril | 87.10 | 90.90 | 97.10 | -10.00 |
| 1405 | Tritriacontane | 305.20 | 297.20 | 295.20 | 10.00 |
| 1406 | 1,4,5-Tris(methoxycarbonyl)naphthalene | 65.77 | 76.20 | 75.80 | -10.03 |
| 1407 | 1-Pyrrolidinyl-3,5-bis(dimethylamino)-s-triazine | 63.53 | 54.70 | 53.50 | 10.03 |
| 1408 | 2,3,5,6-Tetrachloronitrobenzene | 52.13 | 62.10 | 62.20 | -10.07 |
| 1409 | Tranilast | 100.50 | 92.30 | 90.40 | 10.10 |
| 1410 | (6E)-5-Chloro-6-{[(methylcarbamoyl)oxy]imino}bicyclo[2.2.1]heptane-2-carbonitrile | 60.40 | 54.00 | 50.30 | 10.10 |
| 1411 | 2-Chloroethyl methacrylate | 72.30 | 64.00 | 62.20 | 10.10 |
| 1412 | p-Fluoromandelic acid | 71.70 | 60.80 | 61.60 | 10.10 |
| 1413 | (4-Ethylphenyl)-(3-thia-1-aza-bicyclo[3.3.1]non-2-ylidene)-amine | 41.60 | 51.60 | 51.70 | -10.10 |
| 1414 | Cyclohexadecane | 86.45 | 95.80 | 96.60 | -10.15 |
| 1415 | 4-Propoxy-4'-trifluoromethyldiphenyldiacetylene | 59.54 | 67.20 | 69.70 | -10.16 |
| 1416 | 6-Octadecynoic acid | 169.50 | 159.50 | 159.30 | 10.20 |
| 1417 | Dodecamethylcyclohexasiloxane | 106.30 | 97.70 | 96.10 | 10.20 |
| 1418 | Oxyfluorfen | 83.80 | 74.80 | 73.60 | 10.20 |
| 1419 | Eicosanomethylcyclodecasiloxane | 149.60 | 142.10 | 139.40 | 10.20 |
| 1420 | 2-Amino-6-methylbenzoic acid | 69.00 | 58.50 | 58.80 | 10.20 |
| 1421 | Isopropyl 2-cyano-6-nitro-1(2H)-quinolinecarboxylate | 69.00 | 59.30 | 58.80 | 10.20 |
| 1422 | N-Theonylthiocarbamic-O-butyl ester | 65.60 | 73.80 | 75.80 | -10.20 |
| 1423 | 4-t-Butylbenzoic acid | 40.70 | 50.80 | 50.90 | -10.20 |
| 1424 | Isoquercitrin | 105.70 | 120.10 | 115.90 | -10.20 |
| 1425 | Heptyl urea | 75.10 | 84.80 | 85.30 | -10.20 |
| 1426 | 1-Naphthyl acetate | 63.31 | 53.30 | 53.10 | 10.21 |
| 1427 | Triamcinolone acetonide | 80.02 | 69.40 | 69.80 | 10.22 |
| 1428 | 2,3-Dimethyl-2-butene | 50.33 | 39.30 | 40.10 | 10.23 |
| 1429 | t-Butylmercaptan | 44.87 | 53.10 | 55.10 | -10.23 |
| 1430 | Diethatyl-ethyl | 74.97 | 83.90 | 85.20 | -10.23 |
| 1431 | Diethylstilbestrol | 71.57 | 80.30 | 81.80 | -10.23 |
| 1432 | 1,2,6-Tris(methoxycarbonyl)naphthalene | 86.15 | 76.20 | 75.90 | 10.25 |
| 1433 | 1,2,3,4-Tetrachloronaphthalene | 51.34 | 60.90 | 61.60 | -10.26 |
| 1434 | trans-1,3-Dimethylcyclohexane | 53.89 | 43.50 | 43.60 | 10.29 |
| 1435 | Lauric acid | 137.00 | 126.70 | 126.70 | 10.30 |
| 1436 | Disperse Red 60 | 67.20 | 78.60 | 77.50 | -10.30 |
| 1437 | N-(4-Methylphenyl)-4H-3,1-benzothiazin-2-amine | 44.40 | 55.80 | 54.70 | -10.30 |
| 1438 | 2,6-Diphenyl-3,4,7,8-tetramethyl-perhydro-oxazino(3,2-b)oxazine | 48.50 | 54.90 | 58.80 | -10.30 |
| 1439 | Quinuclidine | 40.34 | 29.90 | 30.00 | 10.34 |
| 1440 | 1,2-Bis(methoxycarbonyl)naphthalene | 77.05 | 66.90 | 66.70 | 10.35 |
| 1441 | 1,2,3-Tris(methoxycarbonyl)naphthalene | 65.34 | 76.20 | 75.70 | -10.36 |
| 1442 | 3-Diphenylmethyl-3-methyl-2,4-pentandione | 71.27 | 60.90 | 60.90 | 10.37 |
| 1443 | Pentafluoronitrobenzene | 47.13 | 58.00 | 57.50 | -10.37 |
| 1444 | 3,5-Xylenol | 53.38 | 43.70 | 43.00 | 10.38 |
| 1445 | cis-2-Pentene | 58.39 | 48.30 | 48.00 | 10.39 |
| 1446 | Iodobenzene | 40.31 | 50.30 | 50.70 | -10.39 |
| 1447 | 3-Methylnonane | 99.20 | 88.20 | 88.80 | 10.40 |
| 1448 | 2-(1-Cyclohexenyl)cyclohexanone | 61.90 | 72.80 | 72.30 | -10.40 |
| 1449 | Acetohexamide | 89.90 | 80.10 | 79.50 | 10.40 |
| 1450 | 2,2-Dinitropropyl 4,4-dinitropentanoate | 86.53 | 80.60 | 76.10 | 10.43 |
| 1451 | 2,3,6-Tris(methoxycarbonyl)naphthalene | 86.27 | 76.20 | 75.80 | 10.47 |
| 1452 | Ethene | 32.23 | 42.10 | 42.70 | -10.47 |
| 1453 | Methyl 3,6-dichloro-2-methoxybenzoate | 60.72 | 70.90 | 71.20 | -10.48 |
| 1454 | 1-Naphthol | 62.90 | 52.10 | 52.40 | 10.50 |
| 1455 | alpha-D-Glucose | 75.90 | 66.20 | 65.40 | 10.50 |
| 1456 | Disperse Red 11 | 68.50 | 75.90 | 79.00 | -10.50 |
| 1457 | Estra-1,3,5(10)-triene-3-ol-17beta pentanoate | 70.00 | 79.70 | 80.50 | -10.50 |
| 1458 | Benzo[k]fluoranthene | 56.10 | 45.20 | 45.50 | 10.60 |
| 1459 | Propylurea | 40.40 | 50.90 | 51.00 | -10.60 |
| 1460 | 1,1-Diethoxyethane | 65.60 | 75.70 | 76.20 | -10.60 |
| 1461 | n-Butyl acrylate | 82.61 | 72.40 | 72.00 | 10.61 |
| 1462 | 1,4,5,8-Tetra(methoxycarbonyl)naphthalene | 75.69 | 85.40 | 86.30 | -10.61 |
| 1463 | Tetradifon | 68.92 | 59.70 | 58.30 | 10.62 |
| 1464 | 3-Dodecylamino-1,2-propanediol | 176.47 | 166.80 | 165.80 | 10.67 |
| 1465 | Methyl 4-amino-3,5,6-trichloro-2-picolinate | 67.91 | 84.60 | 78.60 | -10.69 |
| 1466 | 1-(4-Nitrophenylethynyl)-4-propoxybenzene | 83.30 | 73.20 | 72.60 | 10.70 |
| 1467 | Glutaramide | 84.60 | 73.50 | 73.90 | 10.70 |
| 1468 | N-Acetylsarcosinamide | 66.40 | 55.60 | 55.70 | 10.70 |
| 1469 | 5,5-Diphenylhydantoin | 63.20 | 54.30 | 52.50 | 10.70 |
| 1470 | 2,3-Pyrazinedicarbonitrile | 48.90 | 60.50 | 59.60 | -10.70 |
| 1471 | 4-Methyl-1-pentene | 41.50 | 52.00 | 52.20 | -10.70 |
| 1472 | 1,3,5,7-Tetroxane | 58.70 | 66.40 | 69.40 | -10.70 |
| 1473 | 4-Bromo-4'-(5-hexenyloxy)biphenyl | 85.40 | 96.20 | 96.10 | -10.70 |
| 1474 | Tetratriacontane | 316.90 | 305.60 | 306.20 | 10.70 |
| 1475 | Chlorprothixene | 75.10 | 84.10 | 85.80 | -10.70 |
| 1476 | 4-Methylumbelliferon | 63.42 | 52.60 | 52.70 | 10.72 |
| 1477 | Cyprazine | 65.13 | 72.90 | 75.90 | -10.77 |
| 1478 | Bis(trimethylsilyl)methane | 78.99 | 68.20 |  | 10.79 |
| 1479 | 1,4,7,10,13-Pentathiacyclopentadecane | 90.40 | 82.00 | 79.60 | 10.80 |
| 1480 | 1,4,8,11-Tetrathiacyclohexadecane | 97.90 | 88.00 | 87.10 | 10.80 |
| 1481 | 1,2,3,6,7,8-Hexachlorodibenzo-p-dioxin | 86.00 | 75.50 | 75.20 | 10.80 |
| 1482 | 1-Propenyl 2-cyano-6-nitro-1(2H)-quinolinecarboxylate | 67.50 | 57.20 | 56.70 | 10.80 |
| 1483 | 4-Iodobenzoic acid | 64.80 | 54.20 | 54.00 | 10.80 |
| 1484 | N-2-(4,6-Lutidyl)-N'-(2-chlorophenyl) thiourea | 90.30 | 79.80 | 79.50 | 10.80 |
| 1485 | 1,8-Diaminonaphthalene | 47.50 | 58.40 | 58.30 | -10.80 |
| 1486 | Butyl 2-cyano-1(2H)-quinolinecarboxylate | 64.80 | 75.00 | 75.60 | -10.80 |
| 1487 | Ethyl 8-(hydroxymethyl)-6-chloro-5,7-dimethyl-2-oxo-2H-1-benzopyran-3-carboxylate | 62.99 | 73.70 | 73.80 | -10.81 |
| 1488 | p-Anthranilic acid | 45.19 | 55.40 | 56.00 | -10.81 |
| 1489 | 3,3,7,7-Tetramethylnonanedioic anhydride | 51.75 | 62.40 | 62.60 | -10.85 |
| 1490 | 3,4-Xylenol | 54.27 | 43.70 | 43.40 | 10.87 |
| 1491 | 4-Methyl-7-(diethylamino)coumarin | 52.02 | 62.40 | 62.90 | -10.88 |
| 1492 | (4-Trifluoromethylphenyl)-(3-thia-1-aza-bicyclo[3.3.1]non-2-ylidene)-amine | 55.40 | 44.60 | 44.50 | 10.90 |
| 1493 | 3,4,5-Trimethoxybenzoic acid | 67.30 | 77.30 | 78.20 | -10.90 |
| 1494 | 4-Perfluorodecyl-1-butanol | 59.20 | 71.60 | 70.10 | -10.90 |
| 1495 | 3-(2,4,6-Trimethoxybenzoyl)-1,2,2-trimethylcyclopentanecarboxylic acid | 68.70 | 79.60 | 79.60 | -10.90 |
| 1496 | 2-Bromonaphthalene | 61.85 | 50.60 | 50.90 | 10.95 |
| 1497 | Hydrobenzoin | 79.85 | 68.90 | 68.90 | 10.95 |
| 1498 | Cyclobutane | 45.13 | 56.80 | 56.10 | -10.97 |
| 1499 | Furan | 33.93 | 43.70 | 44.90 | -10.97 |
| 1500 | N-Methyl-N-(3-methylsulfonylphenyl)nitramine | 69.10 | 58.20 | 58.10 | 11.00 |
| 1501 | 4,4,8,8,14,14,18,18-Octamethylcycloeicosane-1,11-dione | 88.00 | 96.30 | 99.00 | -11.00 |
| 1502 | N-2-Pyridyl-N'-(4-chlorophenyl) thiourea | 74.20 | 83.20 | 85.20 | -11.00 |
| 1503 | 17-Octadecynoic acid | 159.40 | 169.40 | 170.40 | -11.00 |
| 1504 | 9(10H)-Anthracenone | 62.47 | 51.40 | 51.40 | 11.07 |
| 1505 | 2,3-Dichloroanisole | 72.48 | 61.70 | 61.40 | 11.08 |
| 1506 | (3,4-Dichlorophenyl)-(3-thia-1-aza-bicyclo[3.3.1]non-2-ylidene)-amine | 63.40 | 52.60 | 52.30 | 11.10 |
| 1507 | Homopiperonylic acid | 73.40 | 61.60 | 62.30 | 11.10 |
| 1508 | 3-(3-Nitro-4-methoxybenzoyl)-1,2,2-trimethylcyclopentanecarboxylic acid | 75.80 | 64.80 | 64.70 | 11.10 |
| 1509 | Thymoquinone | 56.20 | 46.10 | 45.10 | 11.10 |
| 1510 | N,N,N',N'-Tetraethyloxamide | 54.80 | 65.20 | 65.90 | -11.10 |
| 1511 | 1-Heptanol | 76.50 | 87.80 | 87.60 | -11.10 |
| 1512 | Pentadecane | 156.02 | 144.90 | 144.90 | 11.12 |
| 1513 | 1-Naphthyl benzoate | 51.58 | 61.60 | 62.70 | -11.12 |
| 1514 | 1-Methylpyrrole | 36.07 | 45.40 | 47.20 | -11.13 |
| 1515 | 3,3'-Di-t-butyl-5,5'-dimethyl-2,2'-dihydroxydiphenylmethane | 72.65 | 61.50 | 61.50 | 11.15 |
| 1516 | Refrigerant 115 | 43.55 | 33.00 | 32.40 | 11.15 |
| 1517 | 2,3-Dimethylbutane | 52.96 | 40.00 | 41.80 | 11.16 |
| 1518 | 2-Methyl-1-propanol | 36.93 | 48.10 | 48.10 | -11.17 |
| 1519 | 2,5-Dichlorophenol | 67.78 | 56.40 | 56.60 | 11.18 |
| 1520 | Phenanthridine | 60.18 | 48.20 | 49.00 | 11.18 |
| 1521 | 1,3-Diaminobenzene | 45.92 | 56.40 | 57.10 | -11.18 |
| 1522 | Methyl 2-(2,4,5-trichlorphenoxy)propionate | 88.59 | 77.30 | 77.40 | 11.19 |
| 1523 | 4-t-Butylphenol | 38.90 | 50.50 | 50.10 | -11.20 |
| 1524 | Benzimidazole-2-thione | 40.90 | 50.60 | 52.10 | -11.20 |
| 1525 | Cortisone acetate | 75.50 | 65.20 | 64.30 | 11.20 |
| 1526 | cis-2-Hexene | 67.24 | 56.80 | 56.00 | 11.24 |
| 1527 | cis-1,3-Dimethylcyclohexane | 54.77 | 43.50 | 43.50 | 11.27 |
| 1528 | 2,6-Lutidine | 48.82 | 58.90 | 60.10 | -11.28 |
| 1529 | Chloroethane | 33.02 | 43.70 | 44.30 | -11.28 |
| 1530 | m-Toluidine | 36.41 | 48.20 | 47.70 | -11.29 |
| 1531 | 1,3-Dimethylbenzene | 51.40 | 40.00 | 40.10 | 11.30 |
| 1532 | 3,5-Diisopropylphenol | 37.20 | 48.80 | 48.50 | -11.30 |
| 1533 | 1,3-Dinitro-1,3-diazacyclohexane | 70.20 | 87.00 | 81.50 | -11.30 |
| 1534 | Benzene | 35.40 | 46.50 | 46.70 | -11.30 |
| 1535 | N-Theonylthiocarbamic-O-pentyl ester | 69.40 | 82.30 | 80.70 | -11.30 |
| 1536 | 2-(Chloromethyl)benzonitrile | 61.43 | 49.60 | 50.10 | 11.33 |
| 1537 | N-Benzyl-pivalophenone imine | 82.05 | 72.00 | 70.70 | 11.35 |
| 1538 | Carvoxime | 46.64 | 59.50 | 58.00 | -11.36 |
| 1539 | 1,2,3,7,8-Pentachlorodibenzo-p-dioxin | 83.00 | 72.40 | 71.60 | 11.40 |
| 1540 | (4-Isopropylphenyl)-(3-thia-1-aza-bicyclo[3.3.1]non-2-ylidene)-amine | 56.60 | 45.70 | 45.20 | 11.40 |
| 1541 | Cycloheptane | 47.60 | 59.40 | 59.00 | -11.40 |
| 1542 | cis-1,2-Dimethylcyclohexane | 55.22 | 43.50 | 43.80 | 11.42 |
| 1543 | Neburon | 72.75 | 83.30 | 84.20 | -11.45 |
| 1544 | Methyl 2,4-dichlorophenoxyacetate | 63.82 | 76.20 | 75.30 | -11.48 |
| 1545 | 1,2,4-Trihydroxybenzene | 69.70 | 57.50 | 58.20 | 11.50 |
| 1546 | 1,4-D-Mannonolactone | 84.70 | 73.50 | 73.20 | 11.50 |
| 1547 | Sulfadimidine | 66.00 | 78.30 | 77.50 | -11.50 |
| 1548 | Benzoylamide Deferoxamine | 235.80 | 246.40 | 247.30 | -11.50 |
| 1549 | 1-Bromoperfluorooctane | 54.42 | 42.90 |  | 11.52 |
| 1550 | 4-(cis-4-Hexenyloxy)-3',4'-difluorodiphenyldiacetylene | 96.93 | 85.70 | 85.40 | 11.53 |
| 1551 | Dimethylterephthalate | 76.44 | 65.00 | 64.90 | 11.54 |
| 1552 | 2,3,4-Trimethylpentane | 56.65 | 42.50 | 45.10 | 11.55 |
| 1553 | Ethyl cyanoacetate | 47.73 | 58.60 | 59.30 | -11.57 |
| 1554 | Benzo[a]pyrene | 59.90 | 49.20 | 48.30 | 11.60 |
| 1555 | Sulfamerazine | 61.20 | 72.20 | 72.80 | -11.60 |
| 1556 | N-Decylglycine | 108.90 | 126.40 | 120.50 | -11.60 |
| 1557 | 2,2'-((3-Chloro-4-(4-nitrophenylazo)phenyl)imino)bis-ethanol | 64.30 | 74.50 | 75.90 | -11.60 |
| 1558 | 2,4,6-Trichlorobiphenyl | 49.36 | 61.00 | 61.00 | -11.64 |
| 1559 | Pyrrolidine | 42.44 | 54.60 | 54.10 | -11.66 |
| 1560 | Lactic acid | 39.10 | 50.70 | 50.80 | -11.70 |
| 1561 | 3-Bromopentane | 50.20 | 60.80 | 61.90 | -11.70 |
| 1562 | Propachlor | 74.13 | 64.10 | 62.40 | 11.73 |
| 1563 | 1-Hexanol | 68.11 | 79.40 | 79.90 | -11.79 |
| 1564 | Dichlone | 58.00 | 46.70 | 46.20 | 11.80 |
| 1565 | 4-(4-Fluorophenylethyl)cyclohexyl trans-2-butenoate | 74.58 | 85.40 | 86.40 | -11.82 |
| 1566 | Ethan-1,2-diol | 44.57 | 56.20 | 56.40 | -11.83 |
| 1567 | 4-(3-Fluoro-4-cyanophenyl)cyclohexyl trans-2-butenoate | 53.66 | 65.90 | 65.50 | -11.84 |
| 1568 | Drazoxolon | 63.66 | 72.30 | 75.50 | -11.84 |
| 1569 | 9,10-Dihydrophenanthrene | 41.73 | 53.90 | 53.60 | -11.87 |
| 1570 | 3-Phenylpropanoic acid | 55.40 | 67.40 | 67.30 | -11.90 |
| 1571 | Vitamin B3 | 63.50 | 50.00 | 51.60 | 11.90 |
| 1572 | Salicylsalicylic acid | 67.40 | 79.70 | 79.30 | -11.90 |
| 1573 | 3-Methylpyridine | 55.61 | 43.30 | 43.70 | 11.91 |
| 1574 | 2-Hydroxybenzamide | 70.41 | 59.90 | 58.50 | 11.91 |
| 1575 | Aniline | 39.57 | 51.50 | 51.50 | -11.93 |
| 1576 | Pinacolone | 51.04 | 40.10 | 39.10 | 11.94 |
| 1577 | 2-(1-Naphthyl)acetamide | 72.05 | 60.30 | 60.10 | 11.95 |
| 1578 | Tetracyanoethylene | 52.77 | 42.40 | 40.80 | 11.97 |
| 1579 | Fluometuron | 68.69 | 57.20 | 56.70 | 11.99 |
| 1580 | 1,3,5-Trithiane | 65.90 | 61.90 | 53.90 | 12.00 |
| 1581 | 2,3,7,8-Tetrachlorodibenzofuran | 73.20 | 61.70 | 61.20 | 12.00 |
| 1582 | Butyl octanoate | 129.20 | 117.10 | 117.20 | 12.00 |
| 1583 | (4-Acetylphenyl)-(3-thia-1-aza-bicyclo[3.3.1]non-2-ylidene)-amine | 56.50 | 44.70 | 44.50 | 12.00 |
| 1584 | 3-(Butanoyloxy)-estra-1,3,5(10)-trien-17-one | 57.70 | 70.30 | 69.70 | -12.00 |
| 1585 | Cholestanole | 54.70 | 67.00 | 66.70 | -12.00 |
| 1586 | n-Decanoic acid | 97.40 | 109.80 | 109.40 | -12.00 |
| 1587 | N-Methylthalidomide | 41.90 | 55.30 | 53.90 | -12.00 |
| 1588 | 5-(4-Chloro-2-nitrophenylazo)-1-ethyl-1,2-dihydro-6-hydroxy-4-methyl-2-oxo-3-pyridinecarbonitrile | 70.30 | 80.80 | 82.30 | -12.00 |
| 1589 | 4,4'-Dichlorobenzophenone | 71.71 | 59.40 | 59.70 | 12.01 |
| 1590 | 2-Cyano-4'-methylbiphenyl | 57.92 | 45.90 |  | 12.02 |
| 1591 | Tolan | 61.40 | 50.60 | 49.30 | 12.10 |
| 1592 | 1-Butanol | 50.79 | 62.50 | 62.90 | -12.11 |
| 1593 | 2,6-Di-t-butyl-4-methoxyphenol | 71.86 | 59.80 | 59.70 | 12.16 |
| 1594 | 2,3,4,5,6-Pentachlorobiphenyl | 54.83 | 67.20 | 67.00 | -12.17 |
| 1595 | Torasemide | 85.60 | 74.50 | 73.40 | 12.20 |
| 1596 | p-Methacryloyloxybenzoic acid | 75.00 | 61.00 | 62.80 | 12.20 |
| 1597 | 1,4,7,10-Tetrathiacyclododecane | 62.10 | 71.80 | 74.30 | -12.20 |
| 1598 | 1,3-Benzoxazine-2,4-dione | 57.20 | 44.30 | 45.00 | 12.20 |
| 1599 | Dimethyl carbonate | 28.10 | 38.90 | 40.30 | -12.20 |
| 1600 | Cyclooctatetraene | 41.49 | 50.80 | 53.70 | -12.21 |
| 1601 | 3-Methylthiophene | 51.62 | 40.60 | 39.40 | 12.22 |
| 1602 | 1,7-Bis(methoxycarbonyl)naphthalene | 55.07 | 66.90 | 67.30 | -12.23 |
| 1603 | Diclobenil | 62.73 | 50.30 | 50.50 | 12.23 |
| 1604 | o-Anthranilic acid | 49.07 | 61.70 | 61.30 | -12.23 |
| 1605 | 1,3-Bis(trimethylsilyl)propane | 71.76 | 81.60 | 84.00 | -12.24 |
| 1606 | Myristyl alcohol | 158.75 | 147.10 | 146.50 | 12.25 |
| 1607 | Triptane | 28.53 | 41.00 | 40.80 | -12.27 |
| 1608 | Methyl 3-((hydroxyimino)(4-methoxyphenyl)methyl)-1,2,2-trimethylcyclopentanecarboxylate | 85.40 | 72.70 | 73.10 | 12.30 |
| 1609 | Sulfisoxazole | 62.50 | 72.70 | 74.80 | -12.30 |
| 1610 | 3-Methyl-2-butanone | 51.90 | 39.00 | 39.60 | 12.30 |
| 1611 | Phenylacetamide Deferoxamine | 265.80 | 254.80 | 253.50 | 12.30 |
| 1612 | Lophine | 65.30 | 55.40 | 53.00 | 12.30 |
| 1613 | 1,2,3,4-Tetrahydro-N-methylcarbazole | 45.80 | 59.60 | 58.10 | -12.30 |
| 1614 | 1-Chloro-1-methylcyclopentane | 43.60 | 54.60 | 55.90 | -12.30 |
| 1615 | Testosterone acetate | 67.51 | 55.20 | 55.20 | 12.31 |
| 1616 | Phthalic anhydride | 57.25 | 44.70 | 44.90 | 12.35 |
| 1617 | Hydrocortisone acetate | 74.49 | 63.40 | 62.10 | 12.39 |
| 1618 | Eicosane | 198.50 | 187.20 | 186.10 | 12.40 |
| 1619 | Flurbiprofen | 72.20 | 59.80 | 59.80 | 12.40 |
| 1620 | Benzo[b]fluoranthene | 56.50 | 45.20 | 44.10 | 12.40 |
| 1621 | N-(4-Bromophenyl)-4H-3,1-benzothiazin-2-amine | 51.20 | 61.20 | 63.60 | -12.40 |
| 1622 | 4-(2,4,5-Trichlorophenoxy)butanoic acid | 78.30 | 90.90 | 90.70 | -12.40 |
| 1623 | Decanamide | 96.90 | 109.20 | 109.30 | -12.40 |
| 1624 | 2-Phenylbenzimidazole | 38.75 | 50.00 | 51.20 | -12.45 |
| 1625 | 2-Methyldiphenylmethane | 68.78 | 56.90 | 56.30 | 12.48 |
| 1626 | N-Methyl-N-(3-nitrophenyl)nitramine | 72.18 | 60.90 | 59.70 | 12.48 |
| 1627 | trans-15-Octadecenoic acid | 178.20 | 165.50 | 165.70 | 12.50 |
| 1628 | 3-(4-Methoxy-2,6-dimethylbenzoyl)-1,2,2-trimethylcyclopentanecarboxylic acid | 67.90 | 55.20 | 55.40 | 12.50 |
| 1629 | Benzophenone anilide | 74.30 | 64.20 | 61.80 | 12.50 |
| 1630 | Pivalamide | 56.70 | 45.10 | 44.20 | 12.50 |
| 1631 | 10-Methylacridin-9(10H)-one | 62.00 | 50.30 | 49.50 | 12.50 |
| 1632 | 3-((4-(2-Chloro-4-nitrophenylazo)phenyl)(2-hydroxyethyl)amino)propanenitrile | 61.40 | 73.10 | 73.90 | -12.50 |
| 1633 | Oxamyl | 81.04 | 76.50 | 68.50 | 12.54 |
| 1634 | Diethyl sulphide | 70.35 | 58.40 | 57.80 | 12.55 |
| 1635 | 1,3,5-Tris(methoxycarbonyl)naphthalene | 64.35 | 76.20 | 76.90 | -12.55 |
| 1636 | 5-Methyl-2-nitrophenol | 68.66 | 56.40 | 56.10 | 12.56 |
| 1637 | Methyl 4-(2,4-dichlorophenoxy)butyrate | 105.40 | 93.10 | 92.80 | 12.60 |
| 1638 | Valium | 63.20 | 70.30 | 75.80 | -12.60 |
| 1639 | Undecanedioic acid | 112.90 | 125.40 | 125.50 | -12.60 |
| 1640 | Oxirane | 32.20 | 43.80 | 44.80 | -12.60 |
| 1641 | (4-Chloro-2-tolyloxy)acetic acid | 76.31 | 64.50 | 63.70 | 12.61 |
| 1642 | 1-Nonene | 104.23 | 91.70 | 91.60 | 12.63 |
| 1643 | Caproic aldehyde | 63.27 | 75.20 | 75.90 | -12.63 |
| 1644 | 2-(4-Methoxyphenyl)propiophenone | 66.74 | 79.10 | 79.40 | -12.66 |
| 1645 | 1,3-Benzoxazole-2-thione | 53.19 | 43.60 | 40.50 | 12.69 |
| 1646 | 3-Bromocinnamic acid | 70.10 | 57.50 | 57.40 | 12.70 |
| 1647 | 10-Ethylacridin-9(10H)-one | 63.40 | 51.40 | 50.70 | 12.70 |
| 1648 | 2-Heptanone | 82.90 | 70.30 | 70.20 | 12.70 |
| 1649 | 4,8,12-Trimethyl-1,5,9-trioxacyclododeca-2,6,10-trione | 56.60 | 67.40 | 69.30 | -12.70 |
| 1650 | 1,1,2,2-Tetrachloroethane | 41.50 | 49.10 | 54.20 | -12.70 |
| 1651 | trans-Stilbene | 68.81 | 56.60 | 56.10 | 12.71 |
| 1652 | 1,4-Oxathiane 4,4-dioxide | 50.09 | 61.20 | 62.80 | -12.71 |
| 1653 | 18-Hexylpentatriacontane | 340.76 | 350.50 | 353.50 | -12.74 |
| 1654 | Benzotrifluoride | 56.45 | 44.80 | 43.70 | 12.75 |
| 1655 | Pyrimethanil | 57.25 | 66.80 | 70.00 | -12.75 |
| 1656 | Cyclohexanone | 44.63 | 57.30 | 57.40 | -12.77 |
| 1657 | 3-Phenoxypropionic acid | 85.19 | 73.10 | 72.40 | 12.79 |
| 1658 | 1,1-Dimethylcyclooctadecane | 84.21 | 99.00 | 97.00 | -12.79 |
| 1659 | 1,2,3,4,7,8-Hexachlorodibenzo-p-dioxin | 88.00 | 75.50 | 75.20 | 12.80 |
| 1660 | Ethyl ether | 46.60 | 59.30 | 59.40 | -12.80 |
| 1661 | trans-1,3-Dimethylcyclopentane | 53.04 | 40.10 | 40.20 | 12.84 |
| 1662 | 1,2,3,7-Tetrachlorodibenzo-p-dioxin | 82.00 | 69.30 | 69.10 | 12.90 |
| 1663 | (4-Fluorophenyl)-(3-thia-1-aza-bicyclo[3.3.1]non-2-ylidene)-amine | 61.80 | 48.00 | 48.90 | 12.90 |
| 1664 | Musk ketone | 58.30 | 45.00 | 45.40 | 12.90 |
| 1665 | 5-nitro-1,3-Benzodioxole | 67.10 | 52.40 | 54.20 | 12.90 |
| 1666 | Methyl t-butyl sulfide | 44.09 | 56.20 | 57.00 | -12.91 |
| 1667 | Buprenorphine | 54.55 | 69.40 | 67.50 | -12.95 |
| 1668 | Hexamethyldisilane | 54.43 | 67.40 |  | -12.97 |
| 1669 | 1-Dodecene | 104.92 | 117.10 | 117.90 | -12.98 |
| 1670 | Heptyl bromide | 101.50 | 87.10 | 88.50 | 13.00 |
| 1671 | 4-Methoxy-4'-hexyloxy-trans-stilbene | 96.20 | 110.30 | 109.20 | -13.00 |
| 1672 | Triadimenol | 77.80 | 89.40 | 90.80 | -13.00 |
| 1673 | 3-(4-Bromophenyl)-3-hydroxypropanoic acid | 89.36 | 76.00 | 76.30 | 13.06 |
| 1674 | Alachlor | 80.13 | 71.40 | 93.20 | -13.07 |
| 1675 | 13-Octadecynoic acid | 172.40 | 159.50 | 159.30 | 13.10 |
| 1676 | 1,4,7,10,13,16-Hexaoxacyclooctadecane | 108.90 | 97.80 | 95.80 | 13.10 |
| 1677 | 4-Biphenylmethanol | 71.90 | 59.00 | 58.80 | 13.10 |
| 1678 | Ibuprofen | 73.40 | 60.70 | 60.30 | 13.10 |
| 1679 | 4-Chlorophenyl 2-cyano-6-methoxy-1(2H)-quinolinecarboxylate | 59.70 | 72.10 | 72.80 | -13.10 |
| 1680 | 2-Phenylaniline | 43.40 | 56.60 | 56.50 | -13.10 |
| 1681 | trans-1,2-Dimethylcyclohexane | 56.72 | 43.50 | 43.60 | 13.12 |
| 1682 | 2,2',3,4',5'-Pentachlorobiphenyl | 53.77 | 67.20 | 66.90 | -13.13 |
| 1683 | 3-Tridecyloxy-1,2-propanediol | 158.54 | 171.60 | 171.70 | -13.16 |
| 1684 | Diuron | 78.87 | 65.20 | 65.70 | 13.17 |
| 1685 | Methylenebisthiopropionic acid | 91.68 | 81.70 | 78.50 | 13.18 |
| 1686 | 1,3,5-Triphenylbenzene | 74.89 | 61.90 | 61.70 | 13.19 |
| 1687 | 2,2',3,4,4',5'-Hexachlorobiphenyl | 56.50 | 70.30 | 69.70 | -13.20 |
| 1688 | Tulobuterol | 74.50 | 86.90 | 87.70 | -13.20 |
| 1689 | Diphenylacetic acid | 74.34 | 61.40 | 61.10 | 13.24 |
| 1690 | 1,1'-Diphenyl-1,1'-bicyclooctyl | 83.26 | 98.90 | 96.50 | -13.24 |
| 1691 | Heptachlor | 70.96 | 61.00 | 57.70 | 13.26 |
| 1692 | 2-Ethylhexyl methacrylate | 104.60 | 91.60 | 91.30 | 13.30 |
| 1693 | 16-Octadecynoic acid | 173.20 | 159.50 | 159.90 | 13.30 |
| 1694 | 2,5-Dinonyloxy-1,4-benzoquinone | 202.70 | 190.20 | 189.40 | 13.30 |
| 1695 | 8-Isopropyltheophylline | 60.43 | 47.80 | 47.10 | 13.33 |
| 1696 | N-Laurylnonanamide | 196.70 | 184.40 | 183.30 | 13.40 |
| 1697 | 1,3,9,11-Tetraoxacyclohexadecane | 107.10 | 95.20 | 93.70 | 13.40 |
| 1698 | Thiourea | 34.10 | 47.50 | 47.50 | -13.40 |
| 1699 | 1,1,1-Trifluoroacetylacetone | 36.40 | 48.00 | 49.80 | -13.40 |
| 1700 | 4-Pentoxy-2',3',4'-trifluorodiphenylacetylene | 104.81 | 92.00 | 91.40 | 13.41 |
| 1701 | 1-Methylcyclohexene | 43.16 | 56.40 | 56.60 | -13.44 |
| 1702 | 15-Triacontene | 248.51 | 259.80 | 262.00 | -13.49 |
| 1703 | 8-Octadecynoic acid | 172.80 | 159.50 | 159.30 | 13.50 |
| 1704 | 5-Methyluracil | 54.50 | 43.10 | 41.00 | 13.50 |
| 1705 | 2,2',4,5,5'-PCB | 53.70 | 67.20 | 67.20 | -13.50 |
| 1706 | N-(3-Methoxyphenylmethyl)-N'-2-propenylthiourea | 49.30 | 65.30 | 62.80 | -13.50 |
| 1707 | Cannabidiol | 83.40 | 96.60 | 96.90 | -13.50 |
| 1708 | Heptadecane | 174.61 | 161.80 | 161.10 | 13.51 |
| 1709 | trans-2-Pentene | 62.83 | 48.30 | 49.30 | 13.53 |
| 1710 | 1,2,3-Tris(methoxycarbonyl)benzene | 87.04 | 74.20 | 73.50 | 13.54 |
| 1711 | Triclopyr | 73.63 | 81.50 | 87.20 | -13.57 |
| 1712 | Triphenylamine | 62.21 | 72.80 | 75.80 | -13.59 |
| 1713 | 9-Octadecynoic acid | 172.00 | 159.50 | 158.40 | 13.60 |
| 1714 | Ethinyl Estradiol | 60.40 | 46.70 | 46.80 | 13.60 |
| 1715 | N-Acetylglycyl-L-prolinamide | 71.40 | 58.80 | 57.80 | 13.60 |
| 1716 | Hesperetin | 71.90 | 84.20 | 85.50 | -13.60 |
| 1717 | 2-(3-Hydroxy-2-quinolinyl-1H-indene-3-ol-1-one | 57.30 | 70.20 | 70.90 | -13.60 |
| 1718 | [2,2]Metacyclophane | 53.00 | 66.40 | 66.60 | -13.60 |
| 1719 | 2,2',4,4',5,5'-Hexachlorobiphenyl | 56.50 | 70.30 | 70.10 | -13.60 |
| 1720 | Thiophene | 28.45 | 40.90 | 42.10 | -13.65 |
| 1721 | N-Methyl-N-nitrobutylamine | 113.46 | 100.00 | 99.80 | 13.66 |
| 1722 | 2-Methylnaphthalene | 58.87 | 45.20 | 45.20 | 13.67 |
| 1723 | Naphthalene-1,8-disulfide | 32.93 | 39.90 | 46.60 | -13.67 |
| 1724 | Phenol | 36.63 | 50.20 | 50.30 | -13.67 |
| 1725 | Phenylaminoethyl methacrylate | 85.60 | 70.80 | 71.90 | 13.70 |
| 1726 | Isatin | 58.50 | 45.40 | 44.80 | 13.70 |
| 1727 | Phenacetin | 75.50 | 61.80 | 61.80 | 13.70 |
| 1728 | Methyl N-phenylurethane | 44.80 | 57.40 | 58.50 | -13.70 |
| 1729 | Ethylene oxalate | 32.29 | 46.80 | 46.00 | -13.71 |
| 1730 | Preforan | 50.58 | 63.90 | 64.30 | -13.72 |
| 1731 | 1,3,5-Trinitrobenzene | 44.08 | 56.00 | 57.80 | -13.72 |
| 1732 | Trifluralin | 69.45 | 81.40 | 83.20 | -13.75 |
| 1733 | Methyl perfluorobutanoate | 61.50 | 48.70 | 47.70 | 13.80 |
| 1734 | 1,4-Dinitrobenzene | 39.40 | 52.80 | 53.20 | -13.80 |
| 1735 | Diphenyl sulfide | 54.19 | 66.00 | 68.00 | -13.81 |
| 1736 | 1-Acetyl-2-naphthol | 63.32 | 50.40 | 49.50 | 13.82 |
| 1737 | Indene | 37.55 | 49.70 | 51.40 | -13.85 |
| 1738 | 1-Pentanethiol | 88.78 | 74.90 | 74.90 | 13.88 |
| 1739 | 4-Benzhydryl-2,5-di(4'-chlorophenyl)-4,5-dimethyl-1,3-dioxa-4-aza-2-boracyclohexane | 52.90 | 64.40 | 66.80 | -13.90 |
| 1740 | N-(2-Chlorophenyl)benzenesulfonamide | 84.10 | 70.50 | 70.20 | 13.90 |
| 1741 | 2-(4-Nitrophenyl)-1-(4-pentylphenyl)ethanone | 81.10 | 95.40 | 95.00 | -13.90 |
| 1742 | 2-Methylpiperidine | 49.50 | 63.20 | 63.40 | -13.90 |
| 1743 | 1,2-Bis(bromomethyl)benzene | 73.73 | 59.80 | 59.80 | 13.93 |
| 1744 | 1,3,5-Trihydroxybenzene | 70.15 | 57.50 | 56.20 | 13.95 |
| 1745 | Neopentyl 4,4,4-trinitrobutyrate | 67.75 | 79.60 | 81.70 | -13.95 |
| 1746 | Dimethyl diacetyltartrate | 73.03 | 84.70 | 87.00 | -13.97 |
| 1747 | Glimepiride | 109.70 | 98.60 | 95.70 | 14.00 |
| 1748 | N,N'-Bis(3-methoxyphenylmethyl)thiourea | 93.70 | 80.20 | 79.70 | 14.00 |
| 1749 | N-Acetyl-D-leucine amide | 50.00 | 63.30 | 64.00 | -14.00 |
| 1750 | Cortisol | 73.75 | 59.60 | 59.70 | 14.05 |
| 1751 | 2-Butanol | 66.15 | 52.00 | 52.10 | 14.05 |
| 1752 | trans-Cyclohexyl-1,4-diamine | 78.92 | 93.40 | 93.00 | -14.08 |
| 1753 | Pentyl 4-aminobenzoate | 73.61 | 88.10 | 87.70 | -14.09 |
| 1754 | Cyclooctane | 48.89 | 63.40 | 63.00 | -14.11 |
| 1755 | trans-2,2,5,5-Tetramethyl-3-hexene | 61.03 | 47.00 | 46.90 | 14.13 |
| 1756 | 4-Ethoxy-2',4'-difluorodiphenylacetylene | 78.63 | 64.90 | 64.50 | 14.13 |
| 1757 | 1,3-Bis(methoxycarbonyl)naphthalene | 80.54 | 66.90 | 66.40 | 14.14 |
| 1758 | 2,4,6-Collidine | 41.64 | 55.70 | 55.80 | -14.16 |
| 1759 | endo-2-Cyanonorbornane | 21.64 | 35.50 | 35.80 | -14.16 |
| 1760 | 1,4,8,11-Tetrathiacyclotetradecane | 91.80 | 79.90 | 77.60 | 14.20 |
| 1761 | 1,5,9-Trithiacyclododecane | 86.30 | 73.80 | 72.10 | 14.20 |
| 1762 | 4-Chloro-2'-hydroxy-4'-propoxyazobenzene | 80.30 | 66.00 | 66.10 | 14.20 |
| 1763 | 8-Quinolinol | 63.70 | 49.90 | 49.50 | 14.20 |
| 1764 | Pyromellitic dianhydride | 28.40 | 42.80 | 42.60 | -14.20 |
| 1765 | 3,3',4,4',5,5'-Hexachlorobiphenyl | 56.50 | 70.30 | 70.70 | -14.20 |
| 1766 | Propanamide | 36.60 | 50.00 | 50.80 | -14.20 |
| 1767 | p-Benzidine | 47.70 | 61.50 | 61.90 | -14.20 |
| 1768 | 2,2',3,3',4,4',5,5',6,6'-Decachlorobiphenyl | 68.10 | 82.80 | 82.30 | -14.20 |
| 1769 | 48-Crown-16 | 189.40 | 208.90 | 203.60 | -14.20 |
| 1770 | 2-Propanol | 57.91 | 43.60 | 43.70 | 14.21 |
| 1771 | 2,4,5-T | 88.13 | 74.00 | 73.90 | 14.23 |
| 1772 | 4-Acetoxyacetophenone | 64.20 | 49.60 | 49.90 | 14.30 |
| 1773 | 1,2,7,8-Tetrachlorodibenzofuran | 76.00 | 61.70 | 61.70 | 14.30 |
| 1774 | 2,4,5-Trinitrotoluene | 66.00 | 52.70 | 51.70 | 14.30 |
| 1775 | Ethanol | 31.30 | 45.50 | 45.60 | -14.30 |
| 1776 | 1,13-Tridecanedioic acid | 127.90 | 142.30 | 142.20 | -14.30 |
| 1777 | Celecoxib | 78.80 | 70.90 | 64.40 | 14.40 |
| 1778 | 1,2-Dihydronaphthalene | 39.80 | 53.70 | 54.20 | -14.40 |
| 1779 | Acrylic acid | 33.30 | 48.10 | 47.70 | -14.40 |
| 1780 | 2,4,6-Trinitrotoluene | 66.52 | 52.70 | 52.10 | 14.42 |
| 1781 | Terbuthylazine | 74.84 | 65.00 | 60.40 | 14.44 |
| 1782 | 1,4-Cyclohexanedione bis(ethylene ketal) | 72.97 | 61.20 | 58.50 | 14.47 |
| 1783 | 2-Methyl-1-propanethiol | 38.83 | 52.00 | 53.30 | -14.47 |
| 1784 | Hexachlorobenzene | 50.20 | 65.20 | 64.70 | -14.50 |
| 1785 | Benzo[ghi]perylene | 31.30 | 46.00 | 45.80 | -14.50 |
| 1786 | 1,5-Dimethylnaphthalene | 56.31 | 42.00 | 41.80 | 14.51 |
| 1787 | 3,5-Dichloroanisole | 76.26 | 61.70 | 61.70 | 14.56 |
| 1788 | cis-1,2-Dimethylcyclopentane | 54.56 | 40.10 | 40.00 | 14.56 |
| 1789 | 2,4-Dimethyl-3-pentanone | 54.58 | 41.60 | 40.00 | 14.58 |
| 1790 | 2-Nitroaniline | 47.00 | 60.90 | 61.60 | -14.60 |
| 1791 | 3-Nonylamino-1,2-propanediol | 155.01 | 141.40 | 140.40 | 14.61 |
| 1792 | Tris(N,N-diisobutylamino)-s-triazine | 96.11 | 83.10 | 81.50 | 14.61 |
| 1793 | Binapacryl | 55.37 | 70.00 | 70.00 | -14.63 |
| 1794 | 1-Chloro-1,1-difluoroethane | 18.86 | 31.50 | 33.50 | -14.64 |
| 1795 | Cortisone | 74.46 | 61.40 | 59.80 | 14.66 |
| 1796 | Methyl docosanoate | 231.00 | 216.60 | 216.30 | 14.70 |
| 1797 | trans-10-Octadecenoic acid | 179.50 | 165.50 | 164.80 | 14.70 |
| 1798 | cis-2-Butene | 54.40 | 39.80 | 39.70 | 14.70 |
| 1799 | o-Xylene | 54.90 | 40.00 | 40.20 | 14.70 |
| 1800 | Dimethylene urethane | 48.10 | 32.30 | 33.40 | 14.70 |
| 1801 | Prazepam | 66.10 | 75.50 | 80.80 | -14.70 |
| 1802 | 2-Imidazolidinone | 39.11 | 24.70 | 24.40 | 14.71 |
| 1803 | Indane | 38.77 | 53.10 | 53.50 | -14.73 |
| 1804 | Prednisolone | 75.75 | 62.70 | 61.00 | 14.75 |
| 1805 | 2-(1-Naphthoxy)propionamide | 80.16 | 64.10 | 65.40 | 14.76 |
| 1806 | Cholest-5-en-3-ol | 62.72 | 78.30 | 77.50 | -14.78 |
| 1807 | 3-(4-Methylbenzoyl)-1,2,2-trimethylcyclopentanecarboxylic acid | 64.20 | 49.50 | 49.40 | 14.80 |
| 1808 | 4-Dimethylaminoazobenzene | 59.30 | 44.80 | 44.50 | 14.80 |
| 1809 | Carbic anhydride | 51.30 | 36.80 | 36.50 | 14.80 |
| 1810 | 3-(Dimethylamino)propylamine | 63.70 | 77.90 | 78.50 | -14.80 |
| 1811 | Cubane | 36.57 | 40.10 | 51.40 | -14.83 |
| 1812 | Lactide | 62.20 | 47.30 | 47.30 | 14.90 |
| 1813 | Ethriol | 64.30 | 78.80 | 79.20 | -14.90 |
| 1814 | Methyl tetryl | 51.50 | 63.90 | 66.40 | -14.90 |
| 1815 | trans,cis-2,6-Octadiene-1,8-dioic acid | 60.00 | 76.00 | 74.90 | -14.90 |
| 1816 | 10-Methyl-2-nitroacridin-9(10H)-one | 67.02 | 53.50 | 52.10 | 14.92 |
| 1817 | 4-Methylbenzylalcohol | 65.85 | 50.70 | 50.90 | 14.95 |
| 1818 | Trimethylethylene | 54.37 | 39.60 | 39.40 | 14.97 |
| 1819 | Procymidone | 68.67 | 55.70 | 53.70 | 14.97 |
| 1820 | Propanil | 50.22 | 65.50 | 65.20 | -14.98 |
| 1821 | 3-Azabicyclo[3.2.2]nonane | 63.69 | 50.10 | 48.70 | 14.99 |
| 1822 | 3-(Pentoyloxy)-estra-1,3,5(10)-trien-17-one | 62.81 | 78.60 | 77.80 | -14.99 |
| 1823 | 2,5-Diundecyloxy-1,4-benzoquinone | 239.10 | 224.00 | 224.10 | 15.00 |
| 1824 | Ethyl 8-(hydroxymethyl)-6-chloro-2-oxo-2H-1-benzopyran-3-carboxylate | 90.10 | 73.90 | 75.10 | 15.00 |
| 1825 | 4-Benzyl-5,6-dimethyl-2,5-diphenyl-1,3-dioxa-4-aza-2-boracyclohexane | 31.90 | 44.60 | 46.90 | -15.00 |
| 1826 | 4-Octyloxy-N-(3,5-dimethoxybenzylidene)aniline | 111.60 | 125.40 | 126.60 | -15.00 |
| 1827 | 2-(3-Chlorophenoxy)propanoic acid | 80.83 | 65.80 | 65.80 | 15.03 |
| 1828 | 2,6-Xylenol | 59.26 | 43.70 | 44.20 | 15.06 |
| 1829 | 4-Phenylazophenol (hydrazono form) | 77.59 | 62.80 | 62.50 | 15.09 |
| 1830 | 1,2,3,7,8,9-Hexachlorodibenzo-p-dioxin | 93.00 | 75.50 | 77.90 | 15.10 |
| 1831 | 2,9-Dimethyl-1,10-phenanthroline | 40.40 | 58.40 | 55.50 | -15.10 |
| 1832 | Ethyl undecanoate | 139.50 | 125.50 | 124.30 | 15.20 |
| 1833 | Tetramethyltetraphenylcyclotetrasiloxane | 121.80 | 109.40 | 106.60 | 15.20 |
| 1834 | cis-3,6-Dimethylpiperazine-2,5-dione | 55.10 | 43.90 | 39.90 | 15.20 |
| 1835 | N-Allyl-N'-phenylthiourea | 73.60 | 61.90 | 58.40 | 15.20 |
| 1836 | Tolfenamic acid | 84.90 | 70.90 | 69.70 | 15.20 |
| 1837 | 2-Adamantanol | 46.80 | 31.60 | 31.60 | 15.20 |
| 1838 | Malonamide | 71.71 | 56.60 | 56.50 | 15.21 |
| 1839 | Ioxynil | 69.64 | 55.10 | 54.40 | 15.24 |
| 1840 | Prothionamide | 56.05 | 69.60 | 71.30 | -15.25 |
| 1841 | 9,10-Anthraquinone | 58.70 | 44.50 | 43.40 | 15.30 |
| 1842 | Tetrahydro-2H-1,3-oxazine-2-thione | 46.00 | 33.70 | 30.70 | 15.30 |
| 1843 | Picotamide | 70.40 | 83.50 | 85.70 | -15.30 |
| 1844 | 1,26-Dimethoxynona(oxyethylene) | 255.60 | 274.60 | 270.90 | -15.30 |
| 1845 | Estrone acetate | 37.59 | 53.20 | 52.90 | -15.31 |
| 1846 | 11-Cyclohexyleicosane | 180.44 | 195.80 | 195.80 | -15.36 |
| 1847 | 4,4,7,7,13,13,16,16-Octamethylcyclooctadecane-1,10-dione | 102.80 | 88.20 | 87.40 | 15.40 |
| 1848 | 9-Fluorenylmethanol | 69.80 | 54.60 | 54.40 | 15.40 |
| 1849 | Fenbufen | 88.80 | 74.10 | 73.40 | 15.40 |
| 1850 | Lindane | 58.00 | 66.90 | 73.40 | -15.40 |
| 1851 | 1,1,9,9-Tetramethylcyclohexadecane | 68.93 | 86.00 | 84.40 | -15.47 |
| 1852 | 2-Methyldecane | 111.79 | 96.70 | 96.30 | 15.49 |
| 1853 | 1,4-Bis(phenylglyoxaloyl)benzene | 76.00 | 63.10 | 60.50 | 15.50 |
| 1854 | 2,2'-((3-Methyl-4-(4-nitrophenylazo)phenyl)imino)bisethanol | 83.00 | 68.20 | 67.50 | 15.50 |
| 1855 | 2,2,6,6,10,10,14,14-Octamethyl-1,3,9,11-tetraoxacyclohexadecane | 60.67 | 75.30 | 76.20 | -15.53 |
| 1856 | Decane | 117.99 | 102.60 | 102.40 | 15.59 |
| 1857 | 2,2',3,3',6,6'-Hexachlorobiphenyl | 54.81 | 70.30 | 70.40 | -15.59 |
| 1858 | Tetradecanediamide | 165.00 | 149.70 | 149.40 | 15.60 |
| 1859 | Tetratetracontane | 403.10 | 390.20 | 387.50 | 15.60 |
| 1860 | 1,1,4,6-Tetramethylindane | 57.53 | 41.70 | 41.90 | 15.63 |
| 1861 | 1,2-Diphenylhydrazine | 47.73 | 50.40 | 32.10 | 15.63 |
| 1862 | 1-Methylcyclohexanol | 36.34 | 51.40 | 52.00 | -15.66 |
| 1863 | Propylamide deferoxamine | 260.00 | 246.50 | 244.30 | 15.70 |
| 1864 | Stanolone | 59.60 | 44.50 | 43.90 | 15.70 |
| 1865 | 1,I-Dimethylazoxyethane | 71.00 | 61.30 | 55.30 | 15.70 |
| 1866 | 1,3-Dithiane sulfone | 53.10 | 64.40 | 68.80 | -15.70 |
| 1867 | 3-Acetylthiophene | 56.70 | 42.20 | 41.00 | 15.70 |
| 1868 | 11-Decylheneicosane | 252.20 | 265.90 | 267.90 | -15.70 |
| 1869 | trans-Decahydroquinoline | 80.02 | 65.20 | 64.30 | 15.72 |
| 1870 | Ethyl eicosanoate | 217.78 | 201.70 | 202.00 | 15.78 |
| 1871 | 2-Amino-1,3,4-triazole | 51.20 | 53.90 | 67.00 | -15.80 |
| 1872 | Methyl 3-(hydroxyiminophenylmethyl)-1,2,2-trimethylcyclopentanecarboxylate | 80.10 | 63.80 | 64.30 | 15.80 |
| 1873 | 4,11-Diamino-2-butyl-1H-naphth[2,3-f]isoindole-1,3,5,10(2H)-tetraone | 50.69 | 66.40 | 66.50 | -15.81 |
| 1874 | 4-Butoxy-4'-trifluoromethyldiphenyldiacetylene | 61.24 | 75.70 | 77.10 | -15.86 |
| 1875 | 17-Methyltestosterone | 63.30 | 47.00 | 47.40 | 15.90 |
| 1876 | 2-(4-((2-Acetoxyethyl)butylamino)-2-methylphenylazo)-5-nitro-1,3-benzenedicarbonitrile | 89.30 | 73.40 | 73.40 | 15.90 |
| 1877 | 2-(Diethylaminothioxomethyl)-N,N-diethylbenzamide | 81.40 | 66.80 | 65.50 | 15.90 |
| 1878 | Perfluoroethane | 51.40 | 37.90 | 35.50 | 15.90 |
| 1879 | 3,3'-Bis(1-cyclohexylethyl)-5,5'-dimethyldiphenylmethane-2,2'-diol | 73.09 | 88.50 | 89.00 | -15.91 |
| 1880 | Fenoprop | 87.83 | 72.00 | 71.90 | 15.93 |
| 1881 | 2,2-Dimethylpentane | 39.55 | 55.40 | 55.50 | -15.95 |
| 1882 | Hexafluorobenzene | 41.65 | 56.60 | 57.60 | -15.95 |
| 1883 | Arachic alcohol | 214.20 | 197.80 | 198.20 | 16.00 |
| 1884 | 1-Hexadecanol | 179.20 | 164.00 | 163.20 | 16.00 |
| 1885 | 1,4-Bis(2,4,5-triphenylcyclopentadienone-3-yl)benzene | 115.80 | 103.40 | 99.80 | 16.00 |
| 1886 | N,N-Dimethylurea | 65.20 | 49.70 | 49.20 | 16.00 |
| 1887 | 2-Diphenylmethyl-2-methyl-1,3-cyclopentanedione | 87.01 | 70.00 | 71.00 | 16.01 |
| 1888 | 3-Pentanol | 44.47 | 60.50 | 60.50 | -16.03 |
| 1889 | 2-Methyl-2-propanol | 22.76 | 39.50 | 38.80 | -16.04 |
| 1890 | Undecanedinitrile | 97.71 | 112.60 | 113.80 | -16.09 |
| 1891 | 1,2,4,7,8-Pentachlorodibenzo-p-dioxin | 88.00 | 72.40 | 71.90 | 16.10 |
| 1892 | Ethylcarbamate | 64.80 | 50.10 | 48.70 | 16.10 |
| 1893 | Oxolinic acid | 73.60 | 59.30 | 57.50 | 16.10 |
| 1894 | 2-Phenylsuccinic acid | 84.90 | 68.60 | 68.80 | 16.10 |
| 1895 | Succinonitrile | 37.78 | 53.40 | 53.90 | -16.12 |
| 1896 | 1,3-Diethylbarbituric acid | 60.10 | 45.60 | 43.90 | 16.20 |
| 1897 | 1,3-Oxazolidine-2-thione | 43.00 | 29.90 | 26.80 | 16.20 |
| 1898 | Cyclohexanol | 39.30 | 55.50 | 55.50 | -16.20 |
| 1899 | 2,2-Dinitro-1,3-propanediol | 62.50 | 75.90 | 78.70 | -16.20 |
| 1900 | 2,6-Dioxaadamantane | 29.70 | 45.10 | 45.90 | -16.20 |
| 1901 | Thiofanox | 60.04 | 72.50 | 76.30 | -16.26 |
| 1902 | 1,1,10,10-Tetramethylcyclooctadecane | 110.19 | 94.10 | 93.90 | 16.29 |
| 1903 | 4-Octyloxy-N-(3,5-dimethylbenzylidene)aniline | 116.20 | 101.00 | 99.90 | 16.30 |
| 1904 | Ethyl 8-(hydroxymethyl)-6-phenyl-2-oxo-2H-1-benzopyran-3-carboxylate | 90.70 | 75.90 | 74.40 | 16.30 |
| 1905 | (4-Methylphenyl)-(3-thia-1-aza-bicyclo[3.3.1]non-2-ylidene)-amine | 59.00 | 43.10 | 42.70 | 16.30 |
| 1906 | N-Acetyl-L-prolinamide | 70.30 | 53.10 | 54.00 | 16.30 |
| 1907 | Azelamide | 122.11 | 107.40 | 105.80 | 16.31 |
| 1908 | p-Chlorobenzyl p-chlorophenyl sulfide | 93.71 | 79.00 | 77.40 | 16.31 |
| 1909 | Chlorfenac | 51.89 | 68.20 | 68.20 | -16.31 |
| 1910 | Bayleton | 65.06 | 82.40 | 81.40 | -16.34 |
| 1911 | 2-Oxetanone | 39.23 | 54.70 | 55.60 | -16.37 |
| 1912 | Decylcyclohexane | 142.29 | 125.60 | 125.90 | 16.39 |
| 1913 | Nonyl acrylate | 98.80 | 114.70 | 115.20 | -16.40 |
| 1914 | Valdecoxib | 68.00 | 78.70 | 84.40 | -16.40 |
| 1915 | 1,9-Cyclohexadecanedione | 82.47 | 99.70 | 98.90 | -16.43 |
| 1916 | 4-Hexyl-3',4'-difluorodiphenylacetylene | 77.17 | 93.00 | 93.60 | -16.43 |
| 1917 | 2,5-Diheptadecyloxy-1,4-benzoquinone | 339.70 | 325.50 | 323.20 | 16.50 |
| 1918 | N-Decanoylpyrazinamide | 140.30 | 126.50 | 123.80 | 16.50 |
| 1919 | Methyl hexadecanoate | 181.40 | 165.80 | 164.90 | 16.50 |
| 1920 | N-(4-Chloro-3-(3-methyl-2-butenyloxy)phenyl)-2-methyl-3-furancarbothiamide | 92.20 | 76.80 | 75.70 | 16.50 |
| 1921 | exo-2-Methylnorbornane | 51.00 | 34.70 | 34.50 | 16.50 |
| 1922 | 3-Octyloxybenzoic acid | 95.42 | 112.20 | 112.00 | -16.58 |
| 1923 | 1,8-Bis(4-(4'-ethylbiphenyl))octane | 122.50 | 140.00 | 139.10 | -16.60 |
| 1924 | n-Nonanal | 116.60 | 100.50 | 100.00 | 16.60 |
| 1925 | 2,6-Dichlorobiphenyl | 41.00 | 57.90 | 57.60 | -16.60 |
| 1926 | Cyclohexanone oxime | 45.80 | 63.00 | 62.40 | -16.60 |
| 1927 | 2,2',3,4,4',5,5'-Heptachlorobiphenyl | 56.50 | 73.40 | 73.10 | -16.60 |
| 1928 | 4-Chlorophenoxyacetic acid | 84.42 | 67.80 | 67.80 | 16.62 |
| 1929 | 3-Undecyloxy-1,2-propanediol | 138.27 | 154.70 | 154.90 | -16.63 |
| 1930 | 3-Heptyloxy-1,2-propanediol | 104.06 | 120.80 | 120.70 | -16.64 |
| 1931 | 1,2,3,4-Tetra(methoxycarbonyl)benzene | 99.79 | 83.50 | 83.10 | 16.69 |
| 1932 | Fenchlorphos | 60.51 | 76.00 | 77.20 | -16.69 |
| 1933 | 4-Chlorobiphenyl | 38.21 | 54.80 | 54.90 | -16.69 |
| 1934 | N-acetyl-isoleucine amide | 78.90 | 63.30 | 62.20 | 16.70 |
| 1935 | Isopropyl Naltrexone-3-O-carbonate | 62.30 | 49.10 | 45.60 | 16.70 |
| 1936 | Astemizole | 114.20 | 98.20 | 97.50 | 16.70 |
| 1937 | 3,3',4,4'-Tetraaminodiphenyl ether | 62.80 | 79.20 | 79.50 | -16.70 |
| 1938 | 2,3-Butyleneglycol | 35.20 | 52.20 | 51.90 | -16.70 |
| 1939 | Methyl heptadecanoate | 158.10 | 174.30 | 174.80 | -16.70 |
| 1940 | 1,1,3,3-Tetramethyl-5,5-diphenylcyclotrisiloxane | 65.66 | 81.30 | 82.40 | -16.74 |
| 1941 | 3,4-Lutidine | 55.96 | 40.10 | 39.20 | 16.76 |
| 1942 | Terbacil | 27.92 | 43.80 | 44.70 | -16.78 |
| 1943 | Propazine | 85.39 | 71.50 | 68.60 | 16.79 |
| 1944 | Methyl octadecanoate | 198.50 | 182.80 | 181.70 | 16.80 |
| 1945 | 3-Octyloxy-1,2-propanediol | 112.80 | 129.30 | 129.60 | -16.80 |
| 1946 | N-Acetylglycinamide | 62.70 | 47.20 | 45.90 | 16.80 |
| 1947 | 2-Methylpropyl 6-(acetylamino)-2-cyano-1(2H)-quinolinecarboxylate | 49.00 | 65.00 | 65.80 | -16.80 |
| 1948 | 4-Pentyl-3',4'-difluorodiphenylacetylene | 68.40 | 84.50 | 85.20 | -16.80 |
| 1949 | Dicyclohexylsulfide | 56.40 | 72.20 | 73.20 | -16.80 |
| 1950 | 1-Decanethiol | 134.42 | 117.20 | 117.60 | 16.82 |
| 1951 | 8-Ethyltheophylline | 68.22 | 53.70 | 51.40 | 16.82 |
| 1952 | 1,6-Bis(methylnitramino)hexane | 186.35 | 165.20 | 169.50 | 16.85 |
| 1953 | Prednisolone acetate | 75.67 | 60.50 | 58.80 | 16.87 |
| 1954 | 1,1,5,5,11,11,15,15-Octamethylcycloeicosane | 108.60 | 92.40 | 91.70 | 16.90 |
| 1955 | p-Terphenyl | 74.10 | 56.80 | 57.20 | 16.90 |
| 1956 | 6,7,8,9-Tetrahydro-6-(hydroxymethyl)-1,6-dimethylphenanthro[1,2-b]furan-10,11-dione | 49.50 | 65.80 | 66.40 | -16.90 |
| 1957 | Coronene | 27.00 | 42.80 | 43.90 | -16.90 |
| 1958 | Dimethyl disulfide | 48.78 | 60.00 | 65.70 | -16.92 |
| 1959 | N,N-Dimethylaniline | 41.46 | 57.80 | 58.40 | -16.94 |
| 1960 | 2,3:6,7-Dibenzocycloocta-2,6-dien-1-one | 46.90 | 63.90 | 63.90 | -17.00 |
| 1961 | Hexyl urea | 58.49 | 76.30 | 75.50 | -17.01 |
| 1962 | N-2-(6-Picolyl)-N'-phenylthiourea | 94.42 | 79.90 | 77.40 | 17.02 |
| 1963 | 1,1,3,3-Tetramethyl-1,3-disilacyclobutane | 38.58 | 55.60 |  | -17.02 |
| 1964 | Perfluoroacetone | 56.75 | 39.50 | 39.70 | 17.05 |
| 1965 | 2-Fluorenyl-2-methyl-1,3-cyclohexanedione | 79.65 | 63.50 | 62.60 | 17.05 |
| 1966 | Enanthic acid | 67.15 | 84.40 | 84.20 | -17.05 |
| 1967 | N,N'-Dipropyladipamide | 79.91 | 97.00 | 97.00 | -17.09 |
| 1968 | 1,3,7,9-Tetrachlorodibenzofuran | 80.00 | 61.70 | 62.90 | 17.10 |
| 1969 | Monomethyl terephthalate | 76.50 | 59.70 | 59.40 | 17.10 |
| 1970 | Ethyl 6-(acetylamino)-2-cyano-1(2H)-quinolinecarboxylate | 79.40 | 62.40 | 62.30 | 17.10 |
| 1971 | 3,3-Dimethyl-1-butanol | 40.50 | 57.60 | 57.60 | -17.10 |
| 1972 | 4-Cyano-4'-octylbiphenyl | 87.30 | 105.10 | 104.40 | -17.10 |
| 1973 | 1-Benzoyl-2-naphthol | 75.71 | 58.80 | 58.60 | 17.11 |
| 1974 | n-Heptaldehyde | 99.83 | 83.60 | 82.70 | 17.13 |
| 1975 | 1,8-Cyclotetradecadiyne | 61.06 | 78.30 | 78.20 | -17.14 |
| 1976 | Thioxanthone | 72.76 | 57.20 | 55.60 | 17.16 |
| 1977 | 11-Octadecynoic acid | 174.90 | 159.50 | 157.70 | 17.20 |
| 1978 | Methyl 3-((hydroxyimino)(5,6,7,8-tetrahydro-2-naphthalenyl)methyl)-1,2,2-trimethylcyclopentanecarboxylate | 90.30 | 74.00 | 73.10 | 17.20 |
| 1979 | 1-Pentanol | 53.70 | 70.90 | 70.90 | -17.20 |
| 1980 | 1-Pentadecanol | 172.86 | 155.50 | 155.60 | 17.26 |
| 1981 | 2,3-Xylenol | 60.76 | 43.70 | 43.50 | 17.26 |
| 1982 | Desmetryn | 58.98 | 42.40 | 41.70 | 17.28 |
| 1983 | Hexatriacontane | 337.70 | 322.60 | 320.40 | 17.30 |
| 1984 | 1,7-Phenanthroline | 62.00 | 46.00 | 44.70 | 17.30 |
| 1985 | 1-Phenyl-1-cyclohexyldodecane | 127.70 | 145.00 | 145.00 | -17.30 |
| 1986 | 5-Hydroxymethyl-5,6-dihydro-7H-dibenzo[a,c]cycloheptane | 45.40 | 62.60 | 62.70 | -17.30 |
| 1987 | 4-Propanoyl-4'-heptanoyloxyazobenzene | 67.95 | 87.40 | 85.30 | -17.35 |
| 1988 | 3-(4-Methoxy-3-aminobenzoyl)-1,2,2-trimethylcyclopentanecarboxylic acid | 82.90 | 66.60 | 65.50 | 17.40 |
| 1989 | 4-Methoxy-4'-heptyloxy-trans-stilbene | 101.10 | 118.80 | 118.50 | -17.40 |
| 1990 | exo-5-Norbornene-2,3-dicarboxylic anhydride | 52.30 | 36.80 | 34.90 | 17.40 |
| 1991 | Salicaine | 83.90 | 100.80 | 101.30 | -17.40 |
| 1992 | N-2-Pyridyl-N'-(2-chlorophenyl) thiourea | 65.90 | 83.20 | 83.30 | -17.40 |
| 1993 | 9,9'-Bifluorenyl | 71.07 | 53.10 | 53.60 | 17.47 |
| 1994 | Corticosterone | 73.39 | 57.60 | 55.90 | 17.49 |
| 1995 | Benzalaniline | 61.90 | 45.80 | 44.40 | 17.50 |
| 1996 | Propanolamine | 59.50 | 76.30 | 77.00 | -17.50 |
| 1997 | 4-Aminoazobenzene | 54.50 | 38.50 | 37.00 | 17.50 |
| 1998 | 5,5-Bis(3,3-dimethylbutyl)-2,2,8,8-tetramethylnonane | 102.67 | 120.40 | 120.20 | -17.53 |
| 1999 | Pyrrole | 31.66 | 47.10 | 49.20 | -17.54 |
| 2000 | Tri-t-butylmethanol | 32.64 | 50.20 | 50.20 | -17.56 |
| 2001 | Hexyl bromide | 95.98 | 78.70 | 78.40 | 17.58 |
| 2002 | Perfluoro-n-octane | 55.48 | 37.30 | 37.90 | 17.58 |
| 2003 | 2,4,8-Trichlorodibenzofuran | 76.00 | 58.60 | 58.40 | 17.60 |
| 2004 | 3-Trichlorosilylpropionitrile | 69.00 | 55.40 | 51.40 | 17.60 |
| 2005 | Pimelonitrile | 62.10 | 78.70 | 79.70 | -17.60 |
| 2006 | 4,4'-Didodecanoyloxydiphenyldiacetylene | 243.95 | 228.40 | 226.30 | 17.65 |
| 2007 | 2-Phenyl-5-(p-terphenyl-4-yl)oxazole | 83.30 | 69.80 | 65.60 | 17.70 |
| 2008 | Butanoic acid | 41.40 | 59.00 | 59.10 | -17.70 |
| 2009 | 4-Nitrophenyl-4'-octyloxybenzoate | 106.40 | 124.50 | 124.10 | -17.70 |
| 2010 | 1-(Methylphenethylamino)-3,5-bis(dimethylamino)-s-triazine | 59.96 | 79.10 | 77.70 | -17.74 |
| 2011 | 2,4-Dinitrophenylhydrazine | 39.84 | 54.20 | 57.60 | -17.76 |
| 2012 | Octadecanamide | 158.84 | 176.90 | 176.60 | -17.76 |
| 2013 | 5-[2-Chloro-4-(trifluoromethyl)phenoxy]-2-nitrobenzoic acid | 86.27 | 67.80 | 68.50 | 17.77 |
| 2014 | Chloramben | 78.68 | 61.60 | 60.90 | 17.78 |
| 2015 | 1-(3-Pyridinylcarbonyl)-1,2-dihydro-2-quinolinecarbonitrile | 75.20 | 58.00 | 57.40 | 17.80 |
| 2016 | Leflunomide | 74.00 | 57.10 | 56.20 | 17.80 |
| 2017 | 1,5-Pentanediol | 63.40 | 81.50 | 81.20 | -17.80 |
| 2018 | N-2-(6-Picolyl)-N'-2-tolylthiourea | 94.10 | 76.70 | 76.30 | 17.80 |
| 2019 | n-Nonylmercaptan | 125.14 | 108.70 | 107.30 | 17.84 |
| 2020 | 1,6-Hexamethylene diisocyanate | 90.46 | 108.30 |  | -17.84 |
| 2021 | 4-Pyridinemethanol | 36.22 | 54.00 | 54.10 | -17.88 |
| 2022 | 2-Propyl-6-octyldecalin | 133.00 | 115.80 | 115.10 | 17.90 |
| 2023 | Ketoprofen | 76.80 | 59.70 | 58.90 | 17.90 |
| 2024 | Tribromsalan | 57.60 | 75.70 | 75.50 | -17.90 |
| 2025 | 3,5-Dibromo-4-hydroxybenzonitrile | 69.03 | 52.00 | 51.10 | 17.93 |
| 2026 | 2-Octanone | 96.57 | 78.80 | 78.60 | 17.97 |
| 2027 | 1,2,3,4,7-Pentachlorodibenzo-p-dioxin | 90.00 | 72.40 | 72.00 | 18.00 |
| 2028 | 2,2',4,4',6-Pentachlorodiphenyl ether | 56.50 | 75.00 | 74.50 | -18.00 |
| 2029 | 2,2,3,3-Tetrafluoropropyl 2-cyanoacrylate | 71.50 | 58.20 | 53.50 | 18.00 |
| 2030 | l,1'-Diadamantyl ketone | 48.00 | 31.50 | 30.00 | 18.00 |
| 2031 | 2,2-Dinitropropane | 57.45 | 42.10 | 39.40 | 18.05 |
| 2032 | 1-Bromooctane | 113.20 | 95.60 | 95.10 | 18.10 |
| 2033 | 2,6-Diheptyldecalin | 123.00 | 141.10 | 141.10 | -18.10 |
| 2034 | Metazachlor | 55.30 | 41.50 | 37.20 | 18.10 |
| 2035 | 2,7-Dimethylnaphthalene | 59.97 | 42.00 | 41.80 | 18.17 |
| 2036 | Dodecylcyclohexane | 160.40 | 142.50 | 142.20 | 18.20 |
| 2037 | 3,5-Di-t-butyl-o-quinone | 68.40 | 50.70 | 50.20 | 18.20 |
| 2038 | 1-Propanol | 36.30 | 54.00 | 54.50 | -18.20 |
| 2039 | Dimethylether | 37.49 | 55.30 | 55.70 | -18.21 |
| 2040 | 1-Cyanoadamantane | 52.39 | 33.20 | 34.10 | 18.29 |
| 2041 | 3-Methyl-2,5-piperazinedione | 56.30 | 38.80 | 38.00 | 18.30 |
| 2042 | Trifluoromethanethiol | 42.43 | 55.80 | 60.80 | -18.37 |
| 2043 | Cyclooctanone | 84.20 | 65.40 | 65.80 | 18.40 |
| 2044 | 1,2,3,5-Tetramethylbenzene | 52.01 | 33.50 | 33.60 | 18.41 |
| 2045 | 3-Methyl-4-nitrophenol | 68.33 | 50.10 | 49.90 | 18.43 |
| 2046 | 1,1'-Diphenyl-1,1'-bicyclohexyl | 65.27 | 82.80 | 83.70 | -18.43 |
| 2047 | 3,4-Diethyl-3,4-bis(4-t-butylphenyl)hexane | 74.27 | 93.30 | 92.70 | -18.43 |
| 2048 | Hexaethylcyclotrisiloxane | 92.46 | 77.70 | 74.00 | 18.46 |
| 2049 | Promecarb | 63.77 | 46.00 | 45.30 | 18.47 |
| 2050 | 1-Heptanethiol | 110.40 | 91.80 | 91.90 | 18.50 |
| 2051 | Monomethyl isophthalate | 78.20 | 59.70 | 59.70 | 18.50 |
| 2052 | 2-Pentanol | 42.40 | 60.50 | 60.90 | -18.50 |
| 2053 | Phenylhydrazine | 56.11 | 41.70 | 37.60 | 18.51 |
| 2054 | Pimelic acid | 73.17 | 91.60 | 91.70 | -18.53 |
| 2055 | 2,2,10,10-Tetramethyl-1,3,9,11-tetraoxacyclohexadecane | 69.87 | 85.00 | 88.40 | -18.53 |
| 2056 | Tetryl | 51.46 | 67.20 | 70.00 | -18.54 |
| 2057 | 4-Nitrobenzoic acid | 72.00 | 53.60 | 53.40 | 18.60 |
| 2058 | Decyl 2-cyanoacrylate | 142.00 | 123.60 | 123.40 | 18.60 |
| 2059 | 1,2,4,6,8-Pentachloronaphthalene | 46.00 | 64.00 | 64.60 | -18.60 |
| 2060 | 1,4-Dimethylbenzene | 59.14 | 40.00 | 40.50 | 18.64 |
| 2061 | 1,12-Phenyleneperylene | 31.91 | 47.90 | 50.60 | -18.69 |
| 2062 | Bicalutamide | 93.10 | 76.90 | 74.40 | 18.70 |
| 2063 | 2,2',5,5'-Tetrachlorobiphenyl | 46.10 | 64.10 | 64.80 | -18.70 |
| 2064 | 1,4-Di-t-butylbenzene | 65.80 | 47.20 | 47.10 | 18.70 |
| 2065 | Pethidine | 79.80 | 61.20 | 61.10 | 18.70 |
| 2066 | Neopentyl alcohol | 30.90 | 49.10 | 49.60 | -18.70 |
| 2067 | 2-Methyl-2-butanol | 31.10 | 47.90 | 49.80 | -18.70 |
| 2068 | 4-Propanoyl-4'-tridecanoyloxyazobenzene | 119.50 | 138.20 | 138.20 | -18.70 |
| 2069 | Testosterone | 68.81 | 51.30 | 50.10 | 18.71 |
| 2070 | Acetaldehyde | 22.48 | 41.30 | 41.20 | -18.72 |
| 2071 | Ethyl palmitate | 186.96 | 167.80 | 168.20 | 18.76 |
| 2072 | 1,2,3,7,8-Pentachloronaphthalene | 46.00 | 64.00 | 64.80 | -18.80 |
| 2073 | 2-Hydroxymethyl-1,4-benzodioxan | 79.40 | 60.00 | 60.60 | 18.80 |
| 2074 | 1,2,4-Trimethylbenzene | 55.30 | 36.80 | 36.50 | 18.80 |
| 2075 | 2-(4-Nitrophenyl)-1-(4-butoxyphenyl)ethanone | 74.20 | 92.70 | 93.00 | -18.80 |
| 2076 | 4-Propanoyl-4'-heptadecanoyloxyazobenzene | 154.70 | 172.10 | 173.50 | -18.80 |
| 2077 | Progesterone | 66.80 | 46.80 | 48.00 | 18.80 |
| 2078 | Trichlorotoluene | 59.11 | 42.30 | 40.30 | 18.81 |
| 2079 | Quercetin | 69.72 | 85.90 | 88.60 | -18.88 |
| 2080 | trans-2-Butene | 58.20 | 39.80 | 39.30 | 18.90 |
| 2081 | Ketorolac | 66.40 | 50.00 | 47.50 | 18.90 |
| 2082 | N-Myristylheptanamide | 164.40 | 184.40 | 183.30 | -18.90 |
| 2083 | 4-Propanoyl-4'-pentadecanoyloxyazobenzene | 136.70 | 155.10 | 155.60 | -18.90 |
| 2084 | B-Carotene | 122.81 | 110.70 | 103.90 | 18.91 |
| 2085 | Pentaerythritol tetrabromide | 64.52 | 78.10 | 83.50 | -18.98 |
| 2086 | Ribitol | 100.30 | 82.00 | 81.30 | 19.00 |
| 2087 | Undecanediamide | 142.70 | 124.30 | 123.70 | 19.00 |
| 2088 | 5-Hydroxymethylene-5H-6,7-dihydrodibenzo[a.c]cyclohepten-6-one | 47.30 | 66.70 | 66.30 | -19.00 |
| 2089 | 4-(Trifluoromethyl)-7-(N-ethylamino)coumarin | 70.50 | 54.40 | 51.50 | 19.00 |
| 2090 | Hydromorphone | 66.00 | 49.30 | 47.00 | 19.00 |
| 2091 | 3-Methoxy-alpha-D-glucopyranoside | 97.00 | 77.90 | 78.00 | 19.00 |
| 2092 | Nonadecafluoro-10-eicosanol | 104.50 | 120.40 | 123.50 | -19.00 |
| 2093 | 4-Ethylbenzoic acid | 36.40 | 55.70 | 55.40 | -19.00 |
| 2094 | 4-Methoxy-4'-dodecyloxy-trans-stilbene | 141.30 | 161.10 | 160.30 | -19.00 |
| 2095 | 1,2'-Dinaphthylmethane | 82.64 | 64.00 | 63.60 | 19.04 |
| 2096 | 10,10,13,13-Tetramethylcyclohexadeca-1,5-diyne | 58.25 | 76.60 | 77.30 | -19.05 |
| 2097 | Undecanolactone | 59.23 | 77.50 | 78.30 | -19.07 |
| 2098 | Dicapthon | 89.78 | 72.90 | 70.70 | 19.08 |
| 2099 | Parathion | 56.55 | 73.80 | 75.70 | -19.15 |
| 2100 | Simetryn | 68.00 | 54.00 | 48.80 | 19.20 |
| 2101 | Tetradecamethylcycloheptasiloxane | 87.80 | 108.90 | 107.00 | -19.20 |
| 2102 | Benzyl alcohol | 34.82 | 53.90 | 54.10 | -19.28 |
| 2103 | trans-11-Octadecenoic acid | 184.50 | 165.50 | 165.20 | 19.30 |
| 2104 | 1,3-Dimethyl-5-isopropyluracil | 63.00 | 42.20 | 43.70 | 19.30 |
| 2105 | 2,2',4,4',5-Pentachlorodiphenyl ether | 56.50 | 75.00 | 75.80 | -19.30 |
| 2106 | 2,4,6,N-Tetranitro-N-ethylaniline | 83.60 | 68.30 | 64.30 | 19.30 |
| 2107 | 4,4'-Diisocyanatodiphenylmethane | 87.10 | 67.80 |  | 19.30 |
| 2108 | 2-Thiopheneacetic acid | 41.50 | 60.40 | 60.80 | -19.30 |
| 2109 | Cyclohexylacetic acid | 45.60 | 65.10 | 64.90 | -19.30 |
| 2110 | 1,1-Dimethylazoethane | 59.91 | 49.80 | 40.60 | 19.31 |
| 2111 | 1,1,1-Trichloro-3,3,3-trifluoropropane | 60.45 | 43.90 | 41.10 | 19.35 |
| 2112 | Methyl 3-((hydroxyimino)(4-ethoxyphenyl)methyl)-1,2,2-trimethylcyclopentanecarboxylate | 91.65 | 74.70 | 72.30 | 19.35 |
| 2113 | 1,2,4,6,7,9-Hexachlorodibenzo-p-dioxin | 94.00 | 75.50 | 74.60 | 19.40 |
| 2114 | Dodecane | 138.80 | 119.50 | 119.40 | 19.40 |
| 2115 | 4-Hydroxy-2-pyrrolidone | 59.70 | 41.00 | 40.30 | 19.40 |
| 2116 | Arabitol | 100.30 | 82.00 | 80.80 | 19.50 |
| 2117 | 1,3,5-Tri-t-butylcyclohexane | 68.10 | 48.30 | 48.60 | 19.50 |
| 2118 | 2-(4-Nitrophenyl)-1-(4-(4-propylcyclohexylethyl)phenyl)ethanone | 84.70 | 104.00 | 104.20 | -19.50 |
| 2119 | 4-Methoxyphenyl 4-(11-(1,1,3,3-tetramethyldisiloxanyl)undecanoyloxy)benzoate | 78.70 | 98.20 |  | -19.50 |
| 2120 | n-Octanoic acid | 73.90 | 92.90 | 93.40 | -19.50 |
| 2121 | N-Pentylthalidomide | 63.40 | 81.80 | 82.90 | -19.50 |
| 2122 | Undecyl urea | 99.60 | 118.60 | 119.10 | -19.50 |
| 2123 | cis-2,4,6-Trimethyl-1,3,5-trioxane | 47.32 | 53.80 | 66.90 | -19.58 |
| 2124 | Benzo[c]cinnoline | 48.40 | 28.80 |  | 19.60 |
| 2125 | 4-Butyl-4'-fluorodiphenylacetylene | 56.08 | 74.40 | 75.70 | -19.62 |
| 2126 | 2-Chloroisonitrosoacetanilide | 68.64 | 51.20 | 49.00 | 19.64 |
| 2127 | N,N,N-Tris(3-methylphenyl)amine | 41.76 | 63.00 | 61.40 | -19.64 |
| 2128 | Methacrylic acid | 28.04 | 47.90 | 47.70 | -19.66 |
| 2129 | trans-6-Octadecenoic acid | 184.50 | 165.50 | 164.80 | 19.70 |
| 2130 | Fenofibrate | 91.60 | 73.70 | 71.90 | 19.70 |
| 2131 | 1,3,6,8-Tetrachlorodibenzofuran | 81.00 | 61.70 | 61.30 | 19.70 |
| 2132 | Disperse Red 9 | 65.00 | 47.40 | 45.30 | 19.70 |
| 2133 | 2-Methoxycarbonylnaphthalene | 77.40 | 57.70 | 57.70 | 19.70 |
| 2134 | 2,5-Dibutoxy-1,4-benzoquinone | 87.20 | 105.60 | 106.90 | -19.70 |
| 2135 | 4-Propanoyl-4'-octadecanoyloxyazobenzene | 156.60 | 180.50 | 176.30 | -19.70 |
| 2136 | 3-Tridecylamino-1,2-propanediol | 193.58 | 175.30 | 173.80 | 19.78 |
| 2137 | N-Cyclohexyl-2,4,6-trimethylbenzaldehyde imine | 75.50 | 57.40 | 55.70 | 19.80 |
| 2138 | [3,3]Paracyclophane | 54.70 | 74.40 | 74.50 | -19.80 |
| 2139 | Propylenecarbonate | 42.80 | 24.10 | 23.00 | 19.80 |
| 2140 | Glyceryltriacetate | 93.80 | 76.40 | 74.00 | 19.80 |
| 2141 | 4,4'-Di(2-methoxyethoxy)biphenyl | 97.80 | 118.30 | 117.60 | -19.80 |
| 2142 | Bis(3,5-di-t-butyl-4-hydroxybenzyl)sulfide | 103.30 | 83.70 | 83.40 | 19.90 |
| 2143 | Phenyl 2-cyano-1(2H)-quinolinecarboxylate | 79.00 | 57.90 | 59.10 | 19.90 |
| 2144 | 2:3,6:7-Dibenzobicyclo[3.2.2]nona-2,6-dien-4-one | 28.40 | 47.90 | 48.30 | -19.90 |
| 2145 | 2-t-Butylcarbonyl-indan-1,3-dione | 68.12 | 47.20 | 48.20 | 19.92 |
| 2146 | Dipropyl disulfide | 73.57 | 88.40 | 93.50 | -19.93 |
| 2147 | 3-Heptylthio-1,2-propanediol | 100.11 | 119.90 | 120.10 | -19.99 |
| 2148 | 2,2-Bis(4-cyanatophenyl)propane | 80.00 | 63.20 | 60.00 | 20.00 |
| 2149 | 1,3,5,7,9-Pentoxecane | 65.40 | 75.30 | 85.40 | -20.00 |
| 2150 | Pyracarbolid | 50.40 | 69.20 | 70.40 | -20.00 |
| 2151 | N-Stearylpropanamide | 165.60 | 184.40 | 185.60 | -20.00 |
| 2152 | N-Tetradecylleucine | 162.37 | 144.10 | 142.30 | 20.07 |
| 2153 | 1-Bromononane | 123.40 | 104.00 | 103.30 | 20.10 |
| 2154 | 2,2,6,6,10,10,14,14,18,18,22,22-Dodecamethyl-1,3,13,15-tetraoxacyclotetracosane | 115.90 | 97.80 | 95.80 | 20.10 |
| 2155 | Salicin | 116.90 | 98.10 | 96.80 | 20.10 |
| 2156 | Hexabromobenzene | 41.10 | 59.50 | 61.20 | -20.10 |
| 2157 | p-Acetotoluide | 68.23 | 47.60 | 48.10 | 20.13 |
| 2158 | Octachlorodibenzo-p-dioxin | 102.00 | 81.80 | 81.80 | 20.20 |
| 2159 | Norethindrone benzoate | 78.20 | 58.80 | 58.00 | 20.20 |
| 2160 | Trifluoromethyl (2-hydroxy-I-propenyl) ketone | 36.40 | 55.70 | 56.60 | -20.20 |
| 2161 | Tebuthiuron | 67.72 | 53.60 | 47.40 | 20.32 |
| 2162 | 1,3-Dimethyl-5-propyluracil | 74.20 | 56.60 | 53.80 | 20.40 |
| 2163 | 2,4,6-Trichlorodibenzofuran | 79.00 | 58.60 | 58.60 | 20.40 |
| 2164 | N-Heptylmyristamide | 163.60 | 184.40 | 184.00 | -20.40 |
| 2165 | Nitralin | 66.10 | 83.70 | 86.50 | -20.40 |
| 2166 | trans-2-Cyclohexylcyclohexanol | 44.60 | 64.10 | 65.00 | -20.40 |
| 2167 | 4-Hexyloxybenzylideneamino-4'-fluorobenzene | 70.70 | 92.20 | 91.10 | -20.40 |
| 2168 | 1,4-Diaminoanthraquinone | 49.98 | 67.00 | 70.40 | -20.42 |
| 2169 | N-Salicylidene-3-aminobenzoic acid | 71.36 | 53.30 | 50.90 | 20.46 |
| 2170 | Bromoxynil octanoate | 83.23 | 103.90 | 103.70 | -20.47 |
| 2171 | 1,2,3,7,8-Pentachlorodibenzofuran | 85.00 | 64.80 | 64.50 | 20.50 |
| 2172 | Di-t-butylmethanol | 29.90 | 50.70 | 50.40 | -20.50 |
| 2173 | 2-(4-Nitrophenyl)-1-(4-(4-pentylcyclohexyl)phenyl)ethanone | 82.90 | 104.00 | 103.50 | -20.60 |
| 2174 | cis-1,4-Di-t-butyl-cyclohexane | 30.00 | 50.70 | 50.60 | -20.60 |
| 2175 | Diethylene glycol dimethyl ether | 85.10 | 104.00 | 105.70 | -20.60 |
| 2176 | Cholesteryl stearate | 198.30 | 217.60 | 218.90 | -20.60 |
| 2177 | 1,10-Cyclooctadecanedione bis (ethylene ketal) | 88.72 | 109.70 | 109.40 | -20.68 |
| 2178 | 1-Adamantanol | 54.00 | 33.40 | 33.30 | 20.70 |
| 2179 | 2,4,5-Tribromostyrene | 73.80 | 55.80 | 53.10 | 20.70 |
| 2180 | Acetylbuprenorphine | 50.90 | 70.60 | 71.60 | -20.70 |
| 2181 | Tetroxoprim | 109.50 | 122.60 | 130.20 | -20.70 |
| 2182 | 1-Thiomorpholinyl-3,5-bis(dimethylamino)-s-triazine | 74.33 | 56.50 | 53.60 | 20.73 |
| 2183 | Chlorocyclohexane | 36.35 | 57.30 | 57.10 | -20.75 |
| 2184 | Bulan | 46.65 | 60.40 | 67.40 | -20.75 |
| 2185 | Heptacosane | 265.68 | 246.40 | 244.90 | 20.78 |
| 2186 | 2-Isopropyl-5-methylphenol | 67.88 | 46.20 | 47.10 | 20.78 |
| 2187 | 3-Diphenylmethyl-3-ethyl-2,4-pentanedione | 89.39 | 69.40 | 68.60 | 20.79 |
| 2188 | 2,4,6-Trimethyl-2,4,6-triphenylcyclotrisiloxane | 115.50 | 89.80 | 94.70 | 20.80 |
| 2189 | 1,4,7-Trithiacyclononane | 81.90 | 61.80 | 61.10 | 20.80 |
| 2190 | Thiobarbituric acid | 31.30 | 50.80 | 52.10 | -20.80 |
| 2191 | Pentacosofluorotetradecane | 60.43 | 43.90 | 39.60 | 20.83 |
| 2192 | 1-Methyl alpha-D-mannose | 98.20 | 77.90 | 77.30 | 20.90 |
| 2193 | 4-Ethoxy-4'-trifluoromethyldiphenyldiacetylene | 77.03 | 58.80 | 56.10 | 20.93 |
| 2194 | 1-Octadecanol | 201.80 | 180.90 | 180.80 | 21.00 |
| 2195 | Atrazine | 84.84 | 66.30 | 63.70 | 21.14 |
| 2196 | 1-Methylcyclopentanol | 27.11 | 48.00 | 48.30 | -21.19 |
| 2197 | 1,2,3,7-Tetrachlorodibenzofuran | 83.00 | 61.70 | 61.80 | 21.20 |
| 2198 | 1,3-Dimethyluracil | 58.90 | 39.90 | 37.70 | 21.20 |
| 2199 | 3,3,6,6,10,10,13,13-Octamethylcyclotetradecane-1,8-dione | 50.20 | 72.00 | 71.40 | -21.20 |
| 2200 | Hexa-2,4-diyne-1,6-diyl-bis(4-hexyloxybenzoate) | 147.40 | 168.10 | 168.60 | -21.20 |
| 2201 | 1,3-Dioxolan-2-one | 42.96 | 26.10 | 21.70 | 21.26 |
| 2202 | 4-Propionyl-4'-n-butanoyloxyazobenzene | 41.04 | 62.10 | 62.30 | -21.26 |
| 2203 | Pentacosane | 251.20 | 229.50 | 229.90 | 21.30 |
| 2204 | 1,2,3,4,6,7,8-Heptachlorodibenzo-p-dioxin | 100.00 | 78.60 | 78.70 | 21.30 |
| 2205 | Azelanitrile | 74.40 | 95.70 | 95.70 | -21.30 |
| 2206 | 2,2',3,4,4',5'-Hexachlorodiphenyl ether | 56.50 | 78.10 | 77.80 | -21.30 |
| 2207 | 2,2',4,4',5,5'-Hexachlorodiphenyl ether | 56.50 | 78.10 | 77.80 | -21.30 |
| 2208 | 1,3,5-Tris(1-naphthyl)benzene | 89.53 | 67.70 | 68.20 | 21.33 |
| 2209 | Bromophos | 95.74 | 75.00 | 74.40 | 21.34 |
| 2210 | Carbofuran | 71.17 | 47.60 | 49.70 | 21.47 |
| 2211 | 1,2,3,4-Tetrachlorodibenzofuran | 83.00 | 61.70 | 61.50 | 21.50 |
| 2212 | N-(4-Acetylphenyl)-4H-3,1-benzothiazin-2-amine | 36.90 | 57.40 | 58.40 | -21.50 |
| 2213 | Isoprocarb | 70.78 | 49.20 | 49.20 | 21.58 |
| 2214 | Pentaerythritol | 108.60 | 87.30 | 87.00 | 21.60 |
| 2215 | N-2-(5-Picolyl)-N'-(2-chlorophenyl) thiourea | 52.60 | 73.60 | 74.20 | -21.60 |
| 2216 | Trimellitic anhydride | 27.20 | 48.60 | 48.80 | -21.60 |
| 2217 | Quinalphos | 83.50 | 70.50 | 61.80 | 21.70 |
| 2218 | 2,2',3,4,4',5-Hexachlorodiphenyl ether | 56.50 | 78.10 | 78.20 | -21.70 |
| 2219 | 2,2',4,4',5,6'-Hexachlorodiphenyl ether | 56.50 | 78.10 | 78.20 | -21.70 |
| 2220 | 4-Propionyl-4'-undecanoyloxyazobenzene | 101.10 | 121.30 | 122.80 | -21.70 |
| 2221 | 4-Butoxy-2',3',4'-trifluorodiphenylacetylene | 104.53 | 83.50 | 82.80 | 21.73 |
| 2222 | PCNB | 44.04 | 65.20 | 65.80 | -21.76 |
| 2223 | Carvedilol | 148.70 | 129.60 | 126.90 | 21.80 |
| 2224 | Acetophenone ethylene glycol ketal | 75.50 | 54.30 | 53.70 | 21.80 |
| 2225 | 1,2-Dibenzoylethane | 93.30 | 71.70 | 71.50 | 21.80 |
| 2226 | 1-(4-Nitrophenylethynyl)-4-pentylbenzene | 62.60 | 84.40 | 84.40 | -21.80 |
| 2227 | 2-Mercapto-2-thiazoline | 44.60 | 66.40 |  | -21.80 |
| 2228 | Isopropylamine | 41.15 | 62.40 | 63.00 | -21.85 |
| 2229 | 20-Methylnonatriacontane | 362.10 | 342.00 | 340.20 | 21.90 |
| 2230 | 1,2-Bis(5-(beta-azastyryl)-2-thienyl)-trans-ethylene | 91.60 | 72.40 | 69.70 | 21.90 |
| 2231 | 2,4,6-Trinitrophenol | 43.40 | 65.90 | 65.30 | -21.90 |
| 2232 | 5,5'-Diphenyl-3,3',4,4'-tetramethyl-2,2'-bioxazolidine | 81.00 | 69.60 | 59.10 | 21.90 |
| 2233 | Aminopyrine | 71.50 | 55.60 | 49.60 | 21.90 |
| 2234 | 1,2-Dihydro-6-neopentyl-2-oxonicotinic acid | 41.20 | 62.00 | 63.10 | -21.90 |
| 2235 | N-Hexadecylleucine | 183.57 | 161.00 | 161.60 | 21.97 |
| 2236 | Cholesteryl 2,2,3,3-tetrafluoropropionate | 67.70 | 84.40 | 89.80 | -22.10 |
| 2237 | 5,5-Diphenyl-3,3',4,4'-tetramethyl-2,2'-bioxazolidine | 80.96 | 73.80 | 58.80 | 22.16 |
| 2238 | 2,6-Di-t-butyl-p-cresol | 69.39 | 47.60 | 47.20 | 22.19 |
| 2239 | [2,2]Paracyclophane-1-ene | 37.50 | 59.30 | 59.70 | -22.20 |
| 2240 | (4-Chlorophenyl)-(3-thia-1-aza-bicyclo[3.3.1]non-2-ylidene)-amine | 28.60 | 49.50 | 50.80 | -22.20 |
| 2241 | 1,2-Bis(methylnitramino)ethane | 147.13 | 131.30 | 124.90 | 22.23 |
| 2242 | Propanone | 59.06 | 36.50 | 36.80 | 22.26 |
| 2243 | DCP | 90.08 | 72.10 | 67.80 | 22.28 |
| 2244 | 1-Methyl-3,5-diphenylpyrazole | 52.50 | 70.00 | 74.80 | -22.30 |
| 2245 | 2,3',4,4',5,5'-Hexachlorodiphenyl ether | 56.50 | 78.10 | 78.80 | -22.30 |
| 2246 | Nonanedioic acid | 85.98 | 108.50 | 108.30 | -22.32 |
| 2247 | Tetraethylurea | 81.23 | 60.50 | 58.90 | 22.33 |
| 2248 | Cyclododecamethylhexasilane | 55.33 | 42.30 | 33.00 | 22.33 |
| 2249 | 1,3,5,5-Tetranitro-1,3-diazacyclohexane | 68.31 | 85.70 | 90.70 | -22.39 |
| 2250 | Nonacosane | 286.10 | 263.30 | 263.70 | 22.40 |
| 2251 | 2-Hexadecyloxyethanol | 165.13 | 188.40 | 187.60 | -22.47 |
| 2252 | N-Dodecylglycine | 123.12 | 143.30 | 145.60 | -22.48 |
| 2253 | Cryofluorane | 39.00 | 28.00 | 16.50 | 22.50 |
| 2254 | 2,2-Dimethyl-3-hydroxy-3-phenylpropanoic acid | 92.22 | 69.00 | 69.70 | 22.52 |
| 2255 | N-Hexyl decanamide | 119.61 | 142.10 | 142.20 | -22.59 |
| 2256 | 2,2',3,3',4,4',6-Heptachlorobiphenyl | 51.04 | 73.40 | 73.70 | -22.66 |
| 2257 | Methylenecyclobutane | 41.60 | 62.90 | 64.30 | -22.70 |
| 2258 | 1,1,1,2,2,3,3,4,4,5,5,6,6,7,7-Pentadecafluoro-8-hexadecanone | 119.70 | 96.70 | 97.00 | 22.70 |
| 2259 | Cholesteryl 2-methylpropionate | 62.90 | 84.70 | 85.60 | -22.70 |
| 2260 | 4-Propanoyl-4'-octanoyloxyazobenzene | 74.36 | 95.90 | 97.10 | -22.74 |
| 2261 | Methylamine | 34.13 | 56.10 | 56.90 | -22.77 |
| 2262 | 1,3,5-Tri(2-naphthyl)benzene | 89.90 | 67.70 | 67.10 | 22.80 |
| 2263 | Oxalamide | 25.70 | 48.20 | 48.50 | -22.80 |
| 2264 | Atenolol | 90.80 | 111.10 | 113.60 | -22.80 |
| 2265 | Cyclooctadecane | 126.74 | 103.80 | 103.90 | 22.84 |
| 2266 | 1,3,6-Trimethyluracil | 55.10 | 35.90 | 32.20 | 22.90 |
| 2267 | 1,1,1-Trichlorotrifluoroethane | 14.30 | 35.40 | 37.20 | -22.90 |
| 2268 | N-2-(6-Picolyl)-N'-4-tolylthiourea | 95.90 | 76.70 | 72.90 | 23.00 |
| 2269 | N-(4-Chloro-2-(2-chloro-4-nitrophenylazo)-5-(2-hydroxypropylamino)phenyl)acetamide | 82.50 | 59.90 | 59.50 | 23.00 |
| 2270 | 2,2-Dimethoxybutane | 53.56 | 76.30 | 76.60 | -23.04 |
| 2271 | 1,4,5,8-Tetrakis(propoxy)-anthracene | 107.10 | 128.10 | 130.20 | -23.10 |
| 2272 | Diethyl disulfide | 54.79 | 71.40 | 77.90 | -23.11 |
| 2273 | N-Nitro-bis(N,N-cyanomethyl)amine | 105.34 | 85.80 | 82.20 | 23.14 |
| 2274 | Norethindrone dimethylpropionate | 75.60 | 54.00 | 52.40 | 23.20 |
| 2275 | Nonadecane | 202.10 | 178.70 | 178.90 | 23.20 |
| 2276 | trans-5-Octadecenoic acid | 141.30 | 165.50 | 164.50 | -23.20 |
| 2277 | Chloroambucil | 86.10 | 106.80 | 109.30 | -23.20 |
| 2278 | 4-Ethoxy-4'-fluorodiphenyldiacetylene | 84.71 | 62.20 | 61.50 | 23.21 |
| 2279 | Hexazinone | 52.26 | 67.20 | 75.50 | -23.24 |
| 2280 | 2,5-Dinonadecyloxy-1,4-benzoquinone | 380.20 | 359.40 | 356.90 | 23.30 |
| 2281 | 12-Tricosanone | 228.04 | 205.70 | 204.70 | 23.34 |
| 2282 | Methyl tetradecanoate | 172.17 | 148.90 | 148.70 | 23.47 |
| 2283 | 2,6-Dimethylnaphthalene | 65.39 | 42.00 | 41.90 | 23.49 |
| 2284 | 4-Propanoyl-4'-hexadecanoyloxyazobenzene | 140.00 | 163.60 | 163.50 | -23.50 |
| 2285 | 2-Oxaadamantane | 14.30 | 38.30 | 37.80 | -23.50 |
| 2286 | Solan | 46.28 | 70.20 | 69.80 | -23.52 |
| 2287 | 2,2',3,3',5,5',6,6'-Octachlorobiphenyl | 52.72 | 76.50 | 76.30 | -23.58 |
| 2288 | Cetane | 176.79 | 153.40 | 153.20 | 23.59 |
| 2289 | 2,5-Xylenol | 67.17 | 43.70 | 43.50 | 23.67 |
| 2290 | 2,2,12,12-Tetramethyl-1,3,11,13-tetraoxycycloeicosane | 123.40 | 101.20 | 99.70 | 23.70 |
| 2291 | 1-(4-Nitrophenylethynyl)-4-butoxybenzene | 58.30 | 81.60 | 82.00 | -23.70 |
| 2292 | N-Hexyl tetradecanamide | 151.94 | 176.00 | 175.70 | -23.76 |
| 2293 | 4-Nitrobenzylideneaniline | 70.70 | 48.90 | 46.90 | 23.80 |
| 2294 | 4-Nitro-4'-methylbenzylidene aniline | 67.90 | 45.70 | 44.10 | 23.80 |
| 2295 | 1,1,1,2-Tetrachlorodifluoroethane | 12.70 | 30.50 | 36.50 | -23.80 |
| 2296 | N-Acetyl valinamide | 76.80 | 54.90 | 53.00 | 23.80 |
| 2297 | 6-Dodecyloxyisoquinoline | 121.20 | 144.10 | 145.10 | -23.90 |
| 2298 | Itraconazole | 159.40 | 144.20 | 135.50 | 23.90 |
| 2299 | 3-Acetoxyacetophenone | 73.40 | 49.60 | 49.50 | 23.90 |
| 2300 | 2,6-Dinitrotoluene | 72.82 | 49.60 | 48.90 | 23.92 |
| 2301 | 1,1'-(1,10-Decanediyl)bisthymine | 93.50 | 117.70 | 117.50 | -24.00 |
| 2302 | 2-Amino-4,6-dimethoxypyrimidine | 80.50 | 67.80 | 56.50 | 24.00 |
| 2303 | Dichloroacetic acid | 70.00 | 49.20 | 46.00 | 24.00 |
| 2304 | Naltrexone | 34.90 | 56.10 | 58.90 | -24.00 |
| 2305 | 4-Propanoyl-4'-tetradecanoyloxyazobenzene | 122.20 | 146.70 | 146.20 | -24.00 |
| 2306 | 3-(3,4-Dimethylbenzoyl)-1,2,2-trimethylcyclopentanecarboxylic acid | 70.20 | 46.20 | 46.10 | 24.10 |
| 2307 | 2-(4-Nitrophenyl)-1-(4-(4-butylcyclohexylethyl)phenyl)ethanone | 87.86 | 112.50 | 112.00 | -24.14 |
| 2308 | 4-Ethoxy-2',3',4'-trifluorodiphenylacetylene | 90.25 | 66.60 | 66.10 | 24.15 |
| 2309 | 1,2,7,8-Tetrachloronaphthalene | 36.43 | 60.90 | 60.60 | -24.17 |
| 2310 | 1,3-Dinitro-1,3-diazacyclopentane | 62.30 | 83.30 | 86.50 | -24.20 |
| 2311 | 2,2',3,4,4',5,5'-Heptachlorodiphenyl ether | 56.50 | 81.20 | 80.70 | -24.20 |
| 2312 | Dimethoxymethane | 49.59 | 73.00 | 73.80 | -24.21 |
| 2313 | t-Butylmethylsulfone | 69.03 | 56.10 | 44.80 | 24.23 |
| 2314 | N-2-(6-Picolyl)-N'-(2-chlorophenyl) thiourea | 60.70 | 83.10 | 85.00 | -24.30 |
| 2315 | 1,8-Cyclotetradecanedione | 65.99 | 91.60 | 90.30 | -24.31 |
| 2316 | 3-(Hexoyloxy)-estra-1,3,5(10)-trien-17-one | 62.16 | 87.20 | 86.50 | -24.34 |
| 2317 | Ethanethiol | 25.48 | 49.50 | 49.90 | -24.42 |
| 2318 | Cyclopentene | 29.83 | 52.90 | 54.30 | -24.47 |
| 2319 | 2,2-Dimethoxy-2-phenylacetophonone | 61.63 | 86.20 | 86.10 | -24.47 |
| 2320 | Tetraethylmethane | 48.19 | 72.30 | 72.70 | -24.51 |
| 2321 | 2,5-Dipentadecyloxy-1,4-benzoquinone | 314.70 | 291.70 | 290.10 | 24.60 |
| 2322 | 1,2,4,7,8-Pentachlorodibenzofuran | 89.00 | 64.80 | 64.40 | 24.60 |
| 2323 | Methyl naltrexone-3-O-carbonate | 27.70 | 49.10 | 52.30 | -24.60 |
| 2324 | 2,2-Bis(hydroxymethyl)propanoic acid | 98.05 | 73.20 | 73.40 | 24.65 |
| 2325 | Tetradecane | 161.54 | 136.40 | 136.80 | 24.74 |
| 2326 | Tricosane | 237.69 | 212.60 | 212.90 | 24.79 |
| 2327 | 1,1,4,4,10,10,13,13-0ctamethylcyclooctadecane | 62.30 | 84.30 | 87.10 | -24.80 |
| 2328 | 2,2',4,4',6,6'-Hexachlorobiphenyl | 45.25 | 70.30 | 70.10 | -24.85 |
| 2329 | Sulfamethoxypyridazine | 49.20 | 74.10 |  | -24.90 |
| 2330 | 2,2',3,4,4',5,6'-Heptachlorodiphenyl ether | 56.50 | 81.20 | 81.50 | -25.00 |
| 2331 | Procyazine | 51.34 | 72.40 | 76.40 | -25.06 |
| 2332 | 2-Bromoethyl 2-cyano-6-nitro-1(2H)-quinolinecarboxylate | 47.20 | 71.20 | 72.30 | -25.10 |
| 2333 | 2,2',3,4,4',6,6'-Heptachlorodiphenyl ether | 56.50 | 81.20 | 81.60 | -25.10 |
| 2334 | 4-Benzhydryl-5,6-dimethyl-2,5-di(4'-methylphenyl)-1,3-dioxa-4-aza-2-boracyclohexane | 71.67 | 51.60 | 46.40 | 25.27 |
| 2335 | N-Tetradecylglycine | 137.43 | 160.20 | 162.70 | -25.27 |
| 2336 | (3-Methylphenyl)-(3-thia-1-aza-bicyclo[3.3.1]non-2-ylidene)-amine | 66.50 | 43.10 | 41.20 | 25.30 |
| 2337 | 1,2,4,6,8,9-Hexachlorodibenzofuran | 93.00 | 67.90 | 67.70 | 25.30 |
| 2338 | 2-(4-Nitrophenyl)-1-(4-pentyloxyphenyl)ethanone | 76.60 | 101.20 | 101.90 | -25.30 |
| 2339 | Mercaptodimethur | 77.11 | 55.50 | 51.80 | 25.31 |
| 2340 | 2,4-Hexadiyne | 8.48 | 32.80 | 33.80 | -25.32 |
| 2341 | 2,3,5-Tris(methoxycarbonyl)naphthalene | 101.96 | 76.20 | 76.60 | 25.36 |
| 2342 | Octylbenzene | 127.91 | 102.50 | 102.50 | 25.41 |
| 2343 | N-Butyl tetradecanamide | 133.89 | 159.00 | 159.30 | -25.41 |
| 2344 | Tetramethylbutanedinitrile | 68.65 | 43.60 | 43.20 | 25.45 |
| 2345 | 2,3,4,7,8-Pentachlorodibenzofuran | 90.00 | 64.80 | 64.40 | 25.60 |
| 2346 | Hexamethylbenzene | 51.60 | 27.00 | 26.00 | 25.60 |
| 2347 | 4-Propionyl-4'-decanoyloxyazobenzene | 88.72 | 112.80 | 114.40 | -25.68 |
| 2348 | Isonitrosoacetanilide | 23.20 | 48.10 | 48.90 | -25.70 |
| 2349 | N,N'-Bis(3-methoxyphenyl)thiourea | 108.17 | 84.00 | 82.30 | 25.87 |
| 2350 | Dimethoate | 72.18 | 65.40 | 46.30 | 25.88 |
| 2351 | 4-Chlorophenyl 2-cyano-1(2H)-quinolinecarboxylate | 87.70 | 61.10 | 61.80 | 25.90 |
| 2352 | N-(2-Methyl-4-chlorophenyl)-4H-3,1-benzothiazin-2-amine | 35.30 | 58.90 | 61.30 | -26.00 |
| 2353 | 9-((2-Acetoxyethoxy)methyl)-2-amino-9h-purine | 99.27 | 83.50 | 73.20 | 26.07 |
| 2354 | 1,2,4,5-Tetramethylbenzene | 59.25 | 33.50 | 33.10 | 26.15 |
| 2355 | Ethyl docosanoate | 245.27 | 218.60 | 219.00 | 26.27 |
| 2356 | Sebacamide | 141.90 | 115.80 | 115.60 | 26.30 |
| 2357 | Cyclohexanecarboxylic acid | 30.50 | 56.60 | 56.80 | -26.30 |
| 2358 | 2,2-Bis(3,5,5-trimethylhexanoyloxymethyl)-1,3-propanediyl bis(3,5,5-trimethylhexanoate | 168.80 | 188.50 | 195.10 | -26.30 |
| 2359 | 2,3,4,6,7,8-Hexachlorodibenzofuran | 94.00 | 67.90 | 67.60 | 26.40 |
| 2360 | 1,2,3,4,5,6,7-Heptafluoronaphthalene | 38.00 | 60.20 | 64.40 | -26.40 |
| 2361 | 5,6,7,8-Tetrahydroquinoline | 40.75 | 66.30 | 67.20 | -26.45 |
| 2362 | [2,2]Metaparacyclophane | 39.20 | 66.40 | 65.70 | -26.50 |
| 2363 | Methyl tris(cyanatophenyl)silane | 74.80 | 89.20 | 101.30 | -26.50 |
| 2364 | Oxacyclohexadecan-2-one | 119.12 | 93.70 | 92.60 | 26.52 |
| 2365 | 1,7-Heptanediol | 72.15 | 98.50 | 98.70 | -26.55 |
| 2366 | 1,3-Cyclohexadiene | 26.11 | 53.50 | 52.70 | -26.59 |
| 2367 | Harmine | 90.90 | 65.70 | 64.30 | 26.60 |
| 2368 | Heneicosafluoroeicosane | 86.50 | 111.70 | 113.10 | -26.60 |
| 2369 | N,N'-Bis(2-hydroxyethyl)-1,4-diaminoanthraquinone | 62.05 | 86.30 | 88.70 | -26.65 |
| 2370 | (9Z)-Octadecenoic acid | 138.24 | 165.50 | 164.90 | -26.66 |
| 2371 | Trichloroacetic acid | 17.78 | 41.10 | 44.50 | -26.72 |
| 2372 | Octamethylcyclotetrasiloxane | 100.67 | 75.80 | 73.90 | 26.77 |
| 2373 | Pyrazophos | 84.22 | 102.10 | 111.00 | -26.78 |
| 2374 | 2,5,8,11-Tetraoxododecane | 103.40 | 128.40 | 130.20 | -26.80 |
| 2375 | Methyl heneicosanoate | 233.80 | 208.10 | 206.90 | 26.90 |
| 2376 | Benefin | 107.83 | 81.40 | 80.90 | 26.93 |
| 2377 | Xylylcarb | 71.17 | 43.50 | 44.20 | 26.97 |
| 2378 | N-Hexadecyl-L-alanine | 174.55 | 150.00 | 147.50 | 27.05 |
| 2379 | 4,5,6,7-Tetrachloro-2-(4-n-tridecafluorohexylphenyl)isoindole-1,3-dione | 88.00 | 64.40 | 60.90 | 27.10 |
| 2380 | 2,2',3,3',4,4',6,6'-Octachlorodiphenyl ether | 56.60 | 84.30 | 83.70 | -27.10 |
| 2381 | 2,2',3,3',4,4',5,6'-Octachlorodiphenyl ether | 56.50 | 84.30 | 83.70 | -27.20 |
| 2382 | 3,4,5,6-Tetrahydrophthalic anhydride | 34.60 | 60.70 | 61.80 | -27.20 |
| 2383 | Cetene | 124.39 | 151.00 | 151.60 | -27.21 |
| 2384 | Cholesteryl 2-chloropropionate | 118.55 | 92.60 | 91.30 | 27.25 |
| 2385 | Cyclopentadecane | 65.25 | 91.70 | 92.50 | -27.25 |
| 2386 | Hexachloroethane | 53.19 | 33.00 | 25.90 | 27.29 |
| 2387 | 1,8-Octanediamine | 157.00 | 130.40 | 129.70 | 27.30 |
| 2388 | Adipamide | 109.10 | 82.00 | 81.80 | 27.30 |
| 2389 | 1,2,3,4,6,8-Hexachlorodibenzofuran | 95.00 | 67.90 | 67.70 | 27.30 |
| 2390 | 5,5,9,9,17,17,21,21-Octamethylcyclotetracosane-1,13-dione | 85.70 | 112.40 | 113.00 | -27.30 |
| 2391 | 4-Butoxybenzoic acid | 51.58 | 78.30 | 78.90 | -27.32 |
| 2392 | 1-Nonadecanol | 217.90 | 189.40 | 190.50 | 27.40 |
| 2393 | 2,2'-Diphenyl-bis(5,5-dimethyl-1,3-dioxan-2-yl) | 98.20 | 72.00 | 70.80 | 27.40 |
| 2394 | t-Butylurea | 73.60 | 46.30 | 46.10 | 27.50 |
| 2395 | 1,2,3,6,7,8-Hexachlorodibenzofuran | 95.00 | 67.90 | 67.50 | 27.50 |
| 2396 | 1,3-Dinitro-1,3-diazacycloheptane | 66.57 | 91.10 | 94.10 | -27.53 |
| 2397 | 1,omega-Dimethoxyoctadeca(oxyethylene) | 513.50 | 493.90 | 485.90 | 27.60 |
| 2398 | 1-(Hexamethyleneimino)-3,5-bis(dimethylamino)-s-triazine | 48.60 | 74.70 | 76.20 | -27.60 |
| 2399 | N-2-(4-Picolyl)-N'-(2-chlorophenyl) thiourea | 100.90 | 73.60 | 73.30 | 27.60 |
| 2400 | 1,8-Cyclotetradecanedione bis(ethylene ketal) | 67.08 | 93.60 | 94.70 | -27.62 |
| 2401 | Acetopiperone | 73.17 | 47.50 | 45.50 | 27.67 |
| 2402 | 1,10-Decanediamine | 173.70 | 147.30 | 146.00 | 27.70 |
| 2403 | Methylethyl 6-(acetylamino)-2-cyano-1(2H)-quinolinecarboxylate | 33.60 | 60.40 | 61.30 | -27.70 |
| 2404 | 4-Pentyloxybenzoic acid | 59.70 | 86.80 | 87.40 | -27.70 |
| 2405 | 3,6-Dichlorodibenzofuran | 83.00 | 55.50 | 55.20 | 27.80 |
| 2406 | 2-Butyl-2-ethyl-1,3-propanediol | 65.60 | 93.60 | 93.40 | -27.80 |
| 2407 | 1,2,3,7,8,9-Hexachlorodibenzofuran | 96.00 | 67.90 | 68.10 | 27.90 |
| 2408 | 4,4'-Bis(2-thienylmethylidenamino)stilbene | 79.50 | 58.10 | 51.60 | 27.90 |
| 2409 | N-Hexyl hexadecanamide | 166.13 | 192.90 | 194.20 | -28.07 |
| 2410 | Danazol | 70.80 | 50.00 | 42.70 | 28.10 |
| 2411 | 1,4-Cyclohexadiene | 29.76 | 57.90 | 57.90 | -28.14 |
| 2412 | 4-Propanoyl-4'-dodecanoyloxyazobenzene | 101.90 | 129.70 | 130.10 | -28.20 |
| 2413 | 3-Aminophenylacetic acid | 91.20 | 63.90 | 63.00 | 28.20 |
| 2414 | 3-Decyloxy-1,2-propanediol | 125.08 | 152.50 | 153.30 | -28.22 |
| 2415 | 2,3-Dimethyl-2,3-bis(phenylazo)butane | 61.60 | 48.40 | 33.30 | 28.30 |
| 2416 | Acetamide deferoxamine | 263.80 | 238.00 | 235.50 | 28.30 |
| 2417 | N-(3-Methoxyphenylmethyl)-N'-octadecylthiourea | 171.00 | 198.20 | 199.30 | -28.30 |
| 2418 | Bendiocarb | 73.14 | 47.50 | 44.80 | 28.34 |
| 2419 | 1-(2-Hydroxyethylmethylamino)-3,5-bis(dimethylamino)-s-triazine | 46.40 | 72.90 | 74.80 | -28.40 |
| 2420 | Pentachlorophenol | 37.08 | 65.70 | 65.50 | -28.42 |
| 2421 | Methyl 11-(1,1,3,3-tetramethyldisiloxanyl)undecanoate | 100.70 | 72.20 |  | 28.50 |
| 2422 | 1,2,3,4,7,8-Hexachlorodibenzofuran | 96.00 | 67.90 | 67.50 | 28.50 |
| 2423 | N-Theonylthiocarbamic-O-hexyl ester | 65.00 | 90.70 | 93.50 | -28.50 |
| 2424 | 2-(4-Nitrophenyl)-1-(4-(4-pentylcyclohexylethyl)phenyl)ethanone | 92.79 | 120.90 | 121.30 | -28.51 |
| 2425 | Cyclopentanol | 24.26 | 52.10 | 52.80 | -28.54 |
| 2426 | 4-Pentanoyl-4'-butanoyloxyazobenzene | 52.35 | 79.00 | 80.90 | -28.55 |
| 2427 | Dimethyl fumarate | 94.00 | 64.80 | 65.40 | 28.60 |
| 2428 | Artemisinin | 53.30 | 34.20 | 24.70 | 28.60 |
| 2429 | 4-Amino-4'-nitroazobenzene | 65.30 | 41.70 | 36.70 | 28.60 |
| 2430 | 1,2-Dibromoacenaphthene | 63.22 | 51.90 | 34.60 | 28.62 |
| 2431 | 8-Heptyltheophylline | 69.81 | 96.00 | 98.50 | -28.69 |
| 2432 | Cannabinol | 48.30 | 76.00 | 77.00 | -28.70 |
| 2433 | 1,1,3,3-Tetraethyl-5,5-diphenylcylotrisiloxane | 66.01 | 90.20 | 94.80 | -28.79 |
| 2434 | Antipyrine | 63.60 | 46.60 | 34.80 | 28.80 |
| 2435 | 2-Chloroethyl 2-cyano-6-nitro-1(2H)-quinolinecarboxylate | 41.00 | 70.00 | 69.80 | -28.80 |
| 2436 | 1,2,4,6,7,8-Hexachlorodibenzofuran | 97.00 | 67.90 | 68.10 | 28.90 |
| 2437 | Dimethyl cubane-1,4-dicarboxylate | 87.00 | 67.10 | 58.10 | 28.90 |
| 2438 | 2,2'-Binaphthyl | 84.40 | 55.50 | 55.50 | 28.90 |
| 2439 | Norbornane | 12.30 | 40.70 | 41.20 | -28.90 |
| 2440 | 4-Methoxy-4'-octyloxy-trans-stilbene | 98.90 | 127.20 | 127.80 | -28.90 |
| 2441 | 3-Aminoacetophenone | 78.12 | 49.80 | 49.20 | 28.92 |
| 2442 | Desciclovir | 91.30 | 68.20 | 62.30 | 29.00 |
| 2443 | Behenyl alcohol | 185.62 | 214.70 | 214.70 | -29.08 |
| 2444 | Mestranol | 81.50 | 52.00 | 52.40 | 29.10 |
| 2445 | Pentacosafluorohexacosane | 116.20 | 145.40 | 145.30 | -29.10 |
| 2446 | 4-(1-Methylheptyloxy)-4'-cyanobiphenyl | 72.27 | 100.40 | 101.40 | -29.13 |
| 2447 | 3-Hexyloxybenzoic acid | 66.20 | 95.20 | 95.40 | -29.20 |
| 2448 | Propyl 2-cyano-6-methoxy-1(2H)-quinolinecarboxylate | 45.90 | 75.50 | 75.10 | -29.20 |
| 2449 | Elaidic acid | 193.80 | 165.50 | 164.50 | 29.30 |
| 2450 | Cyclopentylamine | 43.65 | 70.90 | 73.00 | -29.35 |
| 2451 | N-2-(4,6-Lutidyl)-N'-phenylthiourea | 103.90 | 76.70 | 74.50 | 29.40 |
| 2452 | Ethyl tetracosanoate | 265.30 | 235.50 | 235.80 | 29.50 |
| 2453 | Dibenzo-24-crown-8-ether | 143.60 | 116.10 | 114.10 | 29.50 |
| 2454 | 4,5-Dipropyl-4,5-bis-(4-t-butylphenyl)octane | 96.86 | 127.10 | 126.40 | -29.54 |
| 2455 | 1,1,1-Trichloroethane | 6.43 | 33.90 | 36.00 | -29.57 |
| 2456 | Dimethylamine | 32.83 | 60.70 | 62.40 | -29.57 |
| 2457 | 3,3',5,5'-Tetra-t-butyldiphenylmethane-4,4'-diol | 95.98 | 68.70 | 66.10 | 29.88 |
| 2458 | Dimethyl 2,6-cuneanedicarboxylate | 59.60 | 33.10 | 29.70 | 29.90 |
| 2459 | 3-(Undecanoyloxy)-estra-1,3,5(10)-trien-17-one | 98.60 | 129.40 | 128.50 | -29.90 |
| 2460 | 10-Nonadecanone | 202.04 | 171.90 | 172.10 | 29.94 |
| 2461 | Octadecane | 200.70 | 170.30 | 170.70 | 30.00 |
| 2462 | 5-Nonanone | 117.53 | 87.20 | 87.50 | 30.03 |
| 2463 | 1H,1H-Perfluorotetradecan-1-ol | 76.00 | 45.90 |  | 30.10 |
| 2464 | 3-Decylthio-1,2-propanediol | 114.73 | 145.30 | 144.90 | -30.17 |
| 2465 | Estra-1,3,5(10)-triene-3,17-diol(17beta) 3-benzoate | 90.00 | 59.70 | 59.80 | 30.20 |
| 2466 | Pindolol | 130.70 | 101.60 | 100.40 | 30.30 |
| 2467 | Norethindrone heptanoate | 63.50 | 92.80 | 93.80 | -30.30 |
| 2468 | 11-Phenyleicosane | 220.08 | 189.60 | 189.70 | 30.38 |
| 2469 | 2-Tetradecanone | 160.20 | 129.60 | 129.60 | 30.60 |
| 2470 | N-Decyl hexadecanamide | 196.57 | 226.70 | 227.20 | -30.63 |
| 2471 | Hexacontane | 500.41 | 525.60 | 531.10 | -30.69 |
| 2472 | 9-Heptadecanol | 130.80 | 162.00 | 161.50 | -30.70 |
| 2473 | Pentachloroaniline | 36.97 | 67.00 | 67.70 | -30.73 |
| 2474 | 3,3'-Di-naltrexonyl-carbonate | 35.67 | 58.70 | 66.40 | -30.73 |
| 2475 | Cycloheptanol | 28.53 | 59.60 | 59.30 | -30.77 |
| 2476 | Cyclododecanone | 50.20 | 81.60 | 81.00 | -30.80 |
| 2477 | N-2-(3-Picolyl)-N'-(4-chlorophenyl) thiourea | 42.90 | 73.60 | 73.70 | -30.80 |
| 2478 | Levulinic acid | 30.11 | 60.60 | 61.00 | -30.89 |
| 2479 | 1,3-Benzothiazole-2-thione | 48.90 | 79.80 |  | -30.90 |
| 2480 | 2,4-Bis(2-oxo-oxazolidin-3-ylcarbonylamino)toluene | 10.84 | 36.60 | 41.80 | -30.96 |
| 2481 | N-(4-Methoxyphenyl)-4H-3,1-benzothiazin-2-amine | 36.90 | 68.00 | 67.90 | -31.00 |
| 2482 | 1,6-Bis(4-(4'-ethylbiphenyl))hexane | 92.83 | 123.10 | 124.10 | -31.27 |
| 2483 | 3-Decyloxybenzoic acid | 98.20 | 129.10 | 129.50 | -31.30 |
| 2484 | Methyl arachidate | 231.00 | 199.70 | 199.40 | 31.60 |
| 2485 | 3-Amino-2-methylbenzoic acid | 83.90 | 52.10 | 52.30 | 31.60 |
| 2486 | Basketene | 8.20 | 30.50 | 39.80 | -31.60 |
| 2487 | 2,2',3,3',4,5,5',6,6'-Nonachlorobiphenyl | 49.45 | 79.60 | 81.20 | -31.75 |
| 2488 | 2,5-Dihexyloxy-1,4-benzoquinone | 110.30 | 139.40 | 142.10 | -31.80 |
| 2489 | Ethyl azoxybenzenedicarboxylate | 52.90 | 74.20 | 84.70 | -31.80 |
| 2490 | 54-Crown-18 | 257.20 | 231.10 | 225.20 | 32.00 |
| 2491 | 2,4-Dimethylthiazole | 13.00 | 38.80 | 45.00 | -32.00 |
| 2492 | Isobutyl p-aminobenzoate | 32.64 | 65.20 | 64.70 | -32.06 |
| 2493 | 2-Methyl-2-methylsulfonyl-propanal oxime | 71.01 | 53.40 | 38.90 | 32.11 |
| 2494 | Pentacosofluorotetracosane | 99.80 | 128.50 | 132.00 | -32.20 |
| 2495 | 1,23-Dimethoxyocta(oxyethylene) | 217.60 | 250.20 | 249.90 | -32.30 |
| 2496 | 1,1,3,3-Tetramethyl-5,5,7,7-tetraphenylcyclotetrasiloxane | 83.29 | 109.40 | 115.60 | -32.31 |
| 2497 | Cyclododecane | 47.36 | 79.60 | 79.80 | -32.44 |
| 2498 | Perfluoroheptane | 68.28 | 37.40 | 35.80 | 32.48 |
| 2499 | Tetracosane | 253.90 | 221.00 | 221.40 | 32.50 |
| 2500 | 5,6-Dichloro-2-(4-n-tridecafluorohexylphenyl)isoindole-1,3-dione | 88.60 | 58.20 | 56.10 | 32.50 |
| 2501 | N-Dodecyl-L-valine | 87.08 | 118.70 | 119.60 | -32.52 |
| 2502 | 1,2-Diphenyl-2-(N-piperidinyl)-1-ethanone | 97.16 | 67.80 | 64.50 | 32.66 |
| 2503 | Dulcose | 141.40 | 109.60 | 108.70 | 32.70 |
| 2504 | 3-(Heptoyloxy)-estra-1,3,5(10)-trien-17-one | 62.10 | 95.70 | 94.80 | -32.70 |
| 2505 | 2,3-Dimethyl-2,3-bis(4-t-butylphenyl)butane | 89.11 | 59.40 | 56.30 | 32.81 |
| 2506 | Cyclooctan-1,4-dione | 34.95 | 67.30 | 67.90 | -32.95 |
| 2507 | 4-Butylphenyl-(6-(4-butylphenyl)aminomethylene-9-methyl-9H-carbazol-3-ylmethylene)-amine | 64.30 | 91.50 | 97.40 | -33.10 |
| 2508 | Cinnamic anhydride | 102.02 | 69.80 | 68.90 | 33.12 |
| 2509 | 6-(4-Biphenyl)-1-hexene | 55.00 | 88.30 | 88.50 | -33.50 |
| 2510 | Suberamide | 131.00 | 98.90 | 97.40 | 33.60 |
| 2511 | Succinamide deferoxamine | 231.50 | 262.10 | 265.10 | -33.60 |
| 2512 | Benzoyl 2,4,6-trichlorophenylhydrazide | 74.40 | 58.10 | 40.70 | 33.70 |
| 2513 | 8-(4-Biphenyl)-1-octene | 72.00 | 105.20 | 105.80 | -33.80 |
| 2514 | N,N,N',N'-Tetrapropyloxamide | 66.20 | 99.10 | 100.20 | -34.00 |
| 2515 | 4-Butoxyphenyl-(6-(4-butoxyphenyl)aminomethylene-9-methyl-9H-carbazol-3-ylmethylene)-amine | 72.40 | 102.90 | 106.40 | -34.00 |
| 2516 | Heneicosofluorodocosane | 97.80 | 128.60 | 131.80 | -34.00 |
| 2517 | 45-crown-15 | 227.00 | 197.80 | 192.90 | 34.10 |
| 2518 | 1,3-Bis(biphenyl-4-yl)benzene | 100.30 | 67.00 | 66.10 | 34.20 |
| 2519 | 5,5-Dimethyltetrahydro-1,3-oxazin-2-one | 64.80 | 31.20 | 30.40 | 34.40 |
| 2520 | 1,4-Dinitropiperazine | 74.40 | 103.20 | 108.80 | -34.40 |
| 2521 | 2-Acetylamino-9-((2-hydroxyethoxy)methyl)-9H-purine | 109.81 | 86.00 | 75.40 | 34.41 |
| 2522 | Dimethylnitramine | 115.16 | 82.00 | 80.70 | 34.46 |
| 2523 | 2-Chloroanthraquinone | 80.74 | 47.60 | 46.20 | 34.54 |
| 2524 | Aciclovir | 57.63 | 80.40 | 92.20 | -34.57 |
| 2525 | Nonyl N-phenylcarbamate | 85.80 | 118.60 | 120.40 | -34.60 |
| 2526 | 1-Adamantanecarboxylic acid | 4.29 | 39.60 | 38.90 | -34.61 |
| 2527 | Octachlorodibenzofuran | 108.00 | 74.10 | 73.30 | 34.70 |
| 2528 | Tetraallylsilane | 104.50 | 84.90 | 69.80 | 34.70 |
| 2529 | Ethyl triacontanoate | 320.31 | 286.30 | 285.60 | 34.71 |
| 2530 | N-2-(5-Picolyl)-N'-(4-chlorophenyl) thiourea | 107.90 | 73.60 | 73.10 | 34.80 |
| 2531 | 1,2-Bis(5-(4-methoxy-beta-azastyryl)-2-thienyl)-trans-ethylene | 119.40 | 90.30 | 84.40 | 35.00 |
| 2532 | TNX | 84.52 | 49.50 | 49.50 | 35.02 |
| 2533 | Methyl tetrachloroterephthalate | 38.01 | 72.10 | 73.10 | -35.09 |
| 2534 | Quadricyclane | 44.80 | 12.50 | 9.70 | 35.10 |
| 2535 | Octadecamethylcyclononasiloxane | 104.10 | 131.10 | 139.20 | -35.10 |
| 2536 | 4,4'-Didecanoyloxydiphenyldiacetylene | 226.70 | 194.50 | 191.50 | 35.20 |
| 2537 | 2-Norbornanone | 9.19 | 44.70 | 44.40 | -35.21 |
| 2538 | Cyclotetraeicosane | 161.49 | 128.10 | 126.20 | 35.29 |
| 2539 | 2-Propyl-5-(4-bromophenyl)-thiophene | 43.56 | 76.20 | 78.90 | -35.34 |
| 2540 | 3-(Octoyloxy)-estra-1,3,5(10)-trien-17-one | 69.00 | 104.10 | 104.40 | -35.40 |
| 2541 | N-Palmitoyl-pyrazinamide | 142.87 | 177.30 | 178.40 | -35.53 |
| 2542 | 1,1-Dimethylcyclohexadecane | 56.72 | 90.90 | 92.30 | -35.58 |
| 2543 | Methyl 3-((hydroxyimino)(3,4-dimethylphenyl)methyl)-1,2,2-trimethylcyclopentanecarboxylate | 91.90 | 57.30 | 56.30 | 35.60 |
| 2544 | Hexadecanedioic acid | 132.00 | 167.70 | 167.70 | -35.70 |
| 2545 | Norethindrone 6-(4-chlorophenyl)-hexanoate | 69.73 | 104.20 | 105.50 | -35.77 |
| 2546 | 1,2,3,4,6,7,8-Heptachlorodibenzofuran | 106.00 | 71.00 | 70.20 | 35.80 |
| 2547 | Norethindrone | 82.70 | 46.60 | 46.60 | 36.10 |
| 2548 | Methyl pentacosanoate | 276.94 | 242.00 | 240.80 | 36.14 |
| 2549 | Pentafluoroethane | 13.00 | 38.70 | 49.20 | -36.20 |
| 2550 | 4-Hexylbenzoic acid | 53.21 | 89.50 | 89.60 | -36.39 |
| 2551 | Inositol | 96.40 | 62.70 | 59.90 | 36.50 |
| 2552 | 2-Pentadecanone | 174.80 | 138.00 | 138.10 | 36.70 |
| 2553 | Bis(2-cyanoethyl)-N-nitroamine | 137.57 | 102.70 | 100.70 | 36.87 |
| 2554 | 4,7-Dichloro-2-(4-n-tridecafluorohexylphenyl)isoindole-1,3-dione | 92.20 | 58.20 | 55.30 | 36.90 |
| 2555 | 1,12-Dodecanediamine | 197.10 | 164.20 | 160.10 | 37.00 |
| 2556 | Simazine | 94.23 | 61.20 | 57.10 | 37.13 |
| 2557 | Disperse Blue 79 | 136.00 | 99.70 | 98.80 | 37.20 |
| 2558 | 2,2'-Dimethylbiphenyl | 7.78 | 45.20 | 45.00 | -37.22 |
| 2559 | 1,3,5-Cycloheptatriene | 21.11 | 54.30 | 58.40 | -37.29 |
| 2560 | trans-Azobenzene | 65.40 | 33.60 | 28.00 | 37.40 |
| 2561 | 3-(Decanoyloxy)-estra-1,3,5(10)-trien-17-one | 84.30 | 121.00 | 121.70 | -37.40 |
| 2562 | 11-Heneicosanone | 226.31 | 188.80 | 188.90 | 37.41 |
| 2563 | Simvastatin | 71.50 | 104.40 | 109.10 | -37.60 |
| 2564 | 4-Hexylresorcinol | 55.75 | 92.90 | 93.40 | -37.65 |
| 2565 | Dibenzo-18-crown-6 | 128.40 | 93.90 | 90.70 | 37.70 |
| 2566 | Methyl tetracosanoate | 271.70 | 233.50 | 234.00 | 37.70 |
| 2567 | N-Decylleucine | 75.28 | 110.30 | 113.00 | -37.72 |
| 2568 | Pentacyclo[5.4.02,603,1005,9]undecane | 42.98 | 6.80 | 5.10 | 37.88 |
| 2569 | 1,2,3,4,7,8,9-Heptachlorodibenzofuran | 109.00 | 71.00 | 71.00 | 38.00 |
| 2570 | 2-Nonadecanone | 209.30 | 171.90 | 171.10 | 38.20 |
| 2571 | Dulcitol | 141.40 | 103.30 | 103.20 | 38.20 |
| 2572 | Dodecanediamide | 170.20 | 132.80 | 131.70 | 38.50 |
| 2573 | 3-Phenyl-p-quaterphenyl | 105.40 | 67.00 | 66.90 | 38.50 |
| 2574 | 3-Nonyloxy-1,2-propanediol | 99.26 | 137.80 | 138.00 | -38.74 |
| 2575 | 3,3',4,4'-Biphenyltetracarboxy-N,N'-bis(hexylphenyl)diimide | 113.90 | 145.10 | 152.70 | -38.80 |
| 2576 | p-Diacetylbenzene diethyl ketal | 80.19 | 113.90 | 119.10 | -38.91 |
| 2577 | 17-Tetratriacontene | 254.00 | 293.60 | 293.00 | -39.00 |
| 2578 | 1,1,3,3-Tetraethyl-5,5-dimethylcyclotrisiloxane | 37.29 | 73.30 | 76.30 | -39.01 |
| 2579 | 2-t-Butyl-4-methyl-6-alpha-methylbenzylphenol | 92.92 | 54.90 | 53.90 | 39.02 |
| 2580 | Ethirimol | 46.98 | 74.80 | 86.10 | -39.12 |
| 2581 | Globularicitrin | 182.80 | 150.10 | 143.60 | 39.20 |
| 2582 | 3-Heptylamino-1,2-propanediol | 88.64 | 124.50 | 127.90 | -39.26 |
| 2583 | DABCO | 47.27 | 16.40 | 8.00 | 39.27 |
| 2584 | 4-Pentylbenzoic acid | 41.47 | 81.00 | 80.90 | -39.43 |
| 2585 | Docosane | 243.60 | 204.10 | 203.90 | 39.70 |
| 2586 | 2-Hydroxy-4,6,2',4',6'-pentamethylacetophenone | 2.21 | 40.60 | 42.00 | -39.79 |
| 2587 | Camphor | 78.50 | 38.60 | 38.50 | 40.00 |
| 2588 | Diflubenzuron | 112.08 | 73.20 | 71.80 | 40.28 |
| 2589 | 3-Dodecylthio-1,2-propanediol | 122.90 | 162.20 | 163.20 | -40.30 |
| 2590 | 1,2,3,4,6,8,9-Heptachlorodibenzofuran | 111.00 | 71.00 | 70.60 | 40.40 |
| 2591 | 2-Acetoxyacetophenone | 89.30 | 49.60 | 48.30 | 41.00 |
| 2592 | Norethindrone 3-(4-butylcyclohexyl)propionate | 60.16 | 101.40 | 101.30 | -41.14 |
| 2593 | Pentacosofluorododecane | 66.76 | 37.80 | 25.60 | 41.16 |
| 2594 | Ethyl hexacosanoate | 293.77 | 252.40 | 252.40 | 41.37 |
| 2595 | 1,8-Diaza-2,9-dioxocyclotetradecane | 106.10 | 65.90 | 64.60 | 41.50 |
| 2596 | Cholesteryl palmitate | 160.40 | 200.60 | 201.90 | -41.50 |
| 2597 | 4,4'-Bis(maleimidophenyl)methane | 42.30 | 75.10 | 83.90 | -41.60 |
| 2598 | 4-Methoxyisonitrosoacetanilide | 18.10 | 57.00 | 59.80 | -41.70 |
| 2599 | Methyl heptacosanoate | 299.50 | 258.90 | 257.70 | 41.80 |
| 2600 | Octacosane | 297.30 | 254.90 | 255.40 | 41.90 |
| 2601 | Camphoric anhydride | 81.58 | 40.70 | 39.50 | 42.08 |
| 2602 | 4,4'-Dinitrodiphenyl ether | 24.60 | 65.70 | 67.20 | -42.60 |
| 2603 | Hexacosane | 279.30 | 238.00 | 236.50 | 42.80 |
| 2604 | Vioxx | 24.90 | 63.00 | 67.70 | -42.80 |
| 2605 | 3-(Nonanoyloxy)-estra-1,3,5(10)-trien-17-one | 71.20 | 112.60 | 114.10 | -42.90 |
| 2606 | Octaethylcyclotetrasiloxane | 122.94 | 93.20 | 80.00 | 42.94 |
| 2607 | N-Dodecylleucine | 87.44 | 127.20 | 130.90 | -43.46 |
| 2608 | 1,7-Cyclododecanedione | 38.93 | 83.50 | 82.50 | -43.57 |
| 2609 | 1-(2-Chloro-3-pyridinylcarbonyl)-1,2-dihydro-2-quinolinecarbonitrile | 103.90 | 66.20 | 60.20 | 43.70 |
| 2610 | Diamantane | 47.95 | 7.40 | 4.10 | 43.85 |
| 2611 | N-(2-(2-Bromo-4,6-dinitrophenylazo)-5-((2-cyanoethyl)allylamino)-4-methoxyphenyl)acetamide | 127.00 | 84.00 | 82.90 | 44.10 |
| 2612 | 2,5-Dioctyloxy-1,4-benzoquinone | 132.20 | 173.30 | 176.30 | -44.10 |
| 2613 | 2-Acetyl-3,5-dimethylanisole | 3.06 | 47.30 | 47.20 | -44.14 |
| 2614 | Tricyclo[4.2.2.02,5]deca-3,7,9-triene | 4.97 | 44.90 | 49.20 | -44.23 |
| 2615 | 4-Chloroazobenzene | 75.30 | 36.70 | 31.00 | 44.30 |
| 2616 | 1,1'-(1,12-Dodecanediyl)bisthymine | 95.10 | 134.70 | 139.60 | -44.50 |
| 2617 | 2-Acetyl-3,5-dimethylphenol | 4.08 | 48.30 | 48.60 | -44.52 |
| 2618 | Triacontane | 315.00 | 271.80 | 270.40 | 44.60 |
| 2619 | 2-Heneicosanone | 232.55 | 188.80 | 187.90 | 44.65 |
| 2620 | Sufentanil | 64.40 | 104.20 | 109.10 | -44.70 |
| 2621 | 1,1,4,4,12,12,15,15-Octamethylcyclodocosane | 142.45 | 100.40 | 97.60 | 44.85 |
| 2622 | 4,4-Methylenedianiline | 25.40 | 70.00 | 70.30 | -44.90 |
| 2623 | Methyl trans-cinnamate | 107.10 | 60.70 | 61.80 | 45.30 |
| 2624 | Norethindrone 4-hexylcyclohexylcarboxylate | 56.78 | 101.40 | 102.20 | -45.42 |
| 2625 | Docosanamide | 165.10 | 210.70 | 210.60 | -45.50 |
| 2626 | Nonadecylcyclohexane | 246.00 | 201.70 | 200.40 | 45.60 |
| 2627 | 3-(Octyloxy)-estra-1,3,5(10)-trien-17-ol | 62.10 | 108.50 | 107.70 | -45.60 |
| 2628 | Hexadecafluoro-(trifluoromethyl)eicosane | 109.60 | 150.90 | 155.30 | -45.70 |
| 2629 | 4-Ethoxyisonitrosoacetanilide | 15.50 | 59.00 | 61.40 | -45.90 |
| 2630 | Terfenadine | 137.40 | 94.00 | 91.40 | 46.00 |
| 2631 | Perfluorocyclopentane | 54.50 | 9.20 | 8.40 | 46.10 |
| 2632 | 4-Methyl-4-propyl-1,8-diphenyl-2,3,5-trioxabicyclo[4.3.0]non-7-ene | 47.27 | 80.70 | 93.40 | -46.13 |
| 2633 | 3-Tetradecylthio-1,2-propanediol | 133.55 | 179.10 | 179.70 | -46.15 |
| 2634 | Dyrene | 73.04 | 96.30 | 119.30 | -46.26 |
| 2635 | 3-(Dodecanoyloxy)-estra-1,3,5(10)-trien-17-one | 90.60 | 137.80 | 137.10 | -46.50 |
| 2636 | 4-Heptadecanol | 114.60 | 162.00 | 162.40 | -47.80 |
| 2637 | 1-Fluoroadamantane | 6.77 | 49.40 | 54.60 | -47.83 |
| 2638 | N-2-(3-Picolyl)-N'-(2-chlorophenyl) thiourea | 28.00 | 73.60 | 76.00 | -48.00 |
| 2639 | 1,2,3,4,5,6-Hexamethyl-1,2,3,4,5,6-hexaethylcyclohexasilane | 20.89 | 55.60 | 68.90 | -48.01 |
| 2640 | 4,4'-Diundecanoyloxydiphenyldiacetylene | 165.53 | 211.40 | 213.60 | -48.07 |
| 2641 | Cyclooctanol | 15.09 | 63.60 | 63.30 | -48.21 |
| 2642 | 2-Hydroxy-4,6-dimethylacetophenone | 1.65 | 50.40 | 50.70 | -49.05 |
| 2643 | 2-Ethylbiphenyl | 7.74 | 56.90 | 56.90 | -49.16 |
| 2644 | 1,2,3,4-Tetrahydrodibenzothiophene | 116.50 | 68.70 | 66.80 | 49.70 |
| 2645 | Diamantan-4-ol | 55.69 | 9.40 | 5.40 | 50.29 |
| 2646 | Diethyl 2,4,6,8-tetrahydro-4,8-ethanobenzo[1,2-c:4,5-c']dipyrrole-1,7-dicarboxylate | 41.40 | 81.40 | 91.70 | -50.30 |
| 2647 | N,N'-Dihexylsebacamide | 129.30 | 181.60 | 179.60 | -50.30 |
| 2648 | 5,6-Dibutyl-5,6-bis(4-t-butylphenyl)decane | 111.65 | 161.00 | 162.10 | -50.45 |
| 2649 | 1,1-Diphenyl-3,3,5,5,7,7-hexamethylcyclotetrasiloxane | 140.10 | 92.40 | 89.40 | 50.70 |
| 2650 | Cholesteryl myristate | 134.60 | 183.70 | 185.50 | -50.90 |
| 2651 | 2-Buty1-5-(4'-bromobiphenyl-4-yl)thiophene | 42.70 | 89.80 | 93.70 | -51.00 |
| 2652 | Hexadecafluoro-2-(trifluoromethyl)octadecane | 87.54 | 134.00 | 138.90 | -51.36 |
| 2653 | Tetracontane | 405.92 | 356.40 | 354.50 | 51.42 |
| 2654 | 9-Heptadecanone | 205.87 | 154.90 | 154.30 | 51.57 |
| 2655 | Pentacosofluorotriacontane | 130.90 | 179.20 | 182.50 | -51.60 |
| 2656 | N-Tetradecyl-L-valine | 184.00 | 135.60 | 132.10 | 51.90 |
| 2657 | Benzene hexa-n-pentanoate | 183.30 | 227.60 | 235.20 | -51.90 |
| 2658 | 2-Hydroxy-2',4',6'-trimethylacetophenone | 1.39 | 53.40 | 53.70 | -52.31 |
| 2659 | Methyl hexacosanoate | 301.31 | 250.40 | 248.80 | 52.51 |
| 2660 | 4-Phenylthiazole-2-thione | 128.00 | 75.40 |  | 52.60 |
| 2661 | 3-(Octyloxy)-estra-1,3,5(10)-trien-17-one | 57.40 | 110.30 | 110.50 | -53.10 |
| 2662 | Ethyl 4,7-dihydro-4,7-ethano-2H-isoindole-1-carboxylate | 113.00 | 63.70 | 59.80 | 53.20 |
| 2663 | 4-Aminosalicylic acid | 117.90 | 65.30 | 64.70 | 53.20 |
| 2664 | 1,11-Cycloeicosanedione | 168.28 | 115.80 | 114.60 | 53.68 |
| 2665 | Bis(4-aminophenyl) ether | 16.60 | 69.30 | 71.10 | -54.50 |
| 2666 | Trielaidin | 499.00 | 446.50 | 444.10 | 54.90 |
| 2667 | Methyl octacosanoate | 322.46 | 267.40 | 267.00 | 55.46 |
| 2668 | 3-Undecylthio-1,2-propanediol | 99.11 | 153.70 | 154.70 | -55.59 |
| 2669 | N,N'-Dihexyladipamide | 94.56 | 147.80 | 150.20 | -55.64 |
| 2670 | Alemcinal | 84.39 | 125.60 | 140.20 | -55.81 |
| 2671 | 3-(Hexylthio)-1,2-propanediol | 166.78 | 111.40 | 110.80 | 55.98 |
| 2672 | 2,2,6,6,9,9,13,13,17,17,20,20-Dodecamethyl-1,3,12,14-tetraoxacyclodocosane | 141.00 | 89.70 | 85.00 | 56.00 |
| 2673 | Pentatriacontane | 368.05 | 314.10 | 312.00 | 56.05 |
| 2674 | Perfluoro-cis-decahydronaphthalene | 61.09 | 19.10 | 4.90 | 56.19 |
| 2675 | Dipyridamole | 99.50 | 133.20 | 155.70 | -56.20 |
| 2676 | Dotriacontane | 345.10 | 288.70 | 288.50 | 56.60 |
| 2677 | 17beta-4-Heptyloxybenzoyloxy testosterone | 58.98 | 116.70 | 115.60 | -56.62 |
| 2678 | Perfluoro-trans-decahydronaphthalene | 61.09 | 19.10 | 4.30 | 56.79 |
| 2679 | 16-Hentriacontanone | 328.60 | 273.40 | 271.70 | 56.90 |
| 2680 | N-2-(4,6-Lutidyl)-N'-(4-chlorophenyl) thiourea | 133.10 | 79.80 | 76.10 | 57.00 |
| 2681 | Cyclotridecane | 28.34 | 83.60 | 85.40 | -57.06 |
| 2682 | Nonadecafluoro-10-eicosanone | 167.30 | 113.40 | 110.00 | 57.30 |
| 2683 | Dibenzyldisulfide | 130.80 | 88.20 | 72.80 | 58.00 |
| 2684 | 5-Amino-2-hydroxybenzoic acid | 123.70 | 65.30 | 65.20 | 58.50 |
| 2685 | 1,7-Diacetoxy-2,4,6-trinitro-2,4,6-triazaheptane | 91.11 | 121.70 | 150.00 | -58.89 |
| 2686 | 1,4-Bis(4-(4'-butylbiphenyl))butane | 81.30 | 140.00 | 140.20 | -58.90 |
| 2687 | 1-Heptadecanol | 113.30 | 172.40 | 172.50 | -59.20 |
| 2688 | HMX | 126.00 | 94.40 | 66.70 | 59.30 |
| 2689 | Pyrimethanil laurate | 209.10 | 153.50 | 148.80 | 60.30 |
| 2690 | Trilaurin | 386.57 | 330.20 | 326.00 | 60.57 |
| 2691 | 1,1,1,2,3,3,4,4,5,5,6,6-Dodecafluoro-2-(trifluoromethyl)hexadecane | 82.60 | 134.20 | 143.20 | -60.60 |
| 2692 | 2-Methoxybenzophenone | 1.94 | 62.20 | 63.20 | -61.26 |
| 2693 | 5-Phenoyxmethyl-3-phenylcarbamoyl-2-oxazolidone | 31.00 | 64.10 | 92.40 | -61.40 |
| 2694 | 4-Hexyloxybenzylideneamino-4'-chlorobenzene | 33.20 | 93.70 | 95.20 | -62.00 |
| 2695 | N-Decylundecanamide | 123.60 | 184.40 | 185.60 | -62.00 |
| 2696 | Ciprofloxacin | 119.08 | 64.70 | 55.90 | 63.18 |
| 2697 | Pentacontane | 504.20 | 441.00 | 440.70 | 63.50 |
| 2698 | 3-Tridecylthio-1,2-propanediol | 106.72 | 170.70 | 171.20 | -64.48 |
| 2699 | [2.2]Paracyclophane | 0.65 | 66.80 | 66.20 | -65.55 |
| 2700 | Diamantan-1-ol | 74.33 | 9.40 | 8.10 | 66.23 |
| 2701 | Ethyl beta-cyano-3,4-dimethoxy-alph,alpha-dimethyl-beta-(1-methylethyl)benzenepropanoate | 22.80 | 86.10 | 89.20 | -66.40 |
| 2702 | 1,4,5,8-Tetrakis(propoxy)-9,10-anthraquinone | 60.40 | 122.10 | 127.60 | -67.20 |
| 2703 | 2-(Docosanoxy)ethanol | 171.50 | 239.10 | 238.90 | -67.40 |
| 2704 | 1-Heptyl-4-(4-nonanoylphenyl)cyclohexane | 92.60 | 160.20 | 160.70 | -68.10 |
