# Supplementary material for: Calculation of Five Thermodynamic Molecular Descriptors by Means of a General Computer Algorithm Based on the Group-Additivity Method: Standard Enthalpies of Vaporization, Sublimation and Solvation, and Entropy of Fusion of Ordinary Organic Molecules and Total Phase-Change Entropy of Liquid Crystals
Source: Molecules. 2017 Jun 25;22(7):1059. doi: 10.3390/molecules22071059 (PMC6152037; doi:10.3390/molecules22071059)
Supplement: Supplementary file 1 [file molecules-22-01059-s001.zip › S15. Experimental and Calculated Tpc-Entropy Data Table.doc]

|  | Molecules sorted by deviation |  |  |  |  |
| --- | --- | --- | --- | --- | --- |
| No. | Molecule name | ΔS(tpc) exp | ΔS(tpc) calc | ΔS(tpc) test | Deviation |
|  |  |  |  |  |  |
| 1 | 4,4'''-Diheptyloxy-p-quaterphenyl | 98.01 | 98.90 | 98.00 | 0.01 |
| 2 | (3,4-Dicyano-2,5-thiophenediyl) bis(ethynediyl-4,1-phenylene) 3,4-bis(pentyloxy)benzoate | 101.39 | 103.10 | 101.40 | -0.01 |
| 3 | 4-Pentylphenyl 4'-nonyloxythiobenzoate | 111.03 | 109.40 | 111.00 | 0.03 |
| 4 | 1-Methylheptyl 4-(4-(4-tetradecyloxycinnamoyloxy)benzoyloxy)benzoate | 137.38 | 136.80 | 137.30 | 0.08 |
| 5 | 3-(4-Heptylphenyl)-6-hexyloxy-1,2,4,5-tetrazine | 99.48 | 99.40 |  | 0.08 |
| 6 | 1-(2-(5-Pentylthienyl))-3-(4-fluorophenylamino)-2-propen-1-one | 61.62 | 62.20 | 61.70 | -0.08 |
| 7 | Bis(4-propoxyphenyl) 2,2'-bithiophene-5,5'-dicarboxylate | 95.89 | 95.40 | 95.80 | 0.09 |
| 8 | 1,9-Bis(4-nitroazobenzene-4'-oxy)nonane | 108.91 | 108.70 | 109.00 | -0.09 |
| 9 | Pentyl 4-(4-(4-heptyloxy-2,3,5,6-tetrafluorophenylethynyl)benzyloxy)benzoate | 106.31 | 104.70 | 106.40 | -0.09 |
| 10 | 4'-(2-(4-Butylphenyl)-1-ethynyl)-2'-ethyl-4-propyltolane | 63.30 | 62.80 | 63.40 | -0.10 |
| 11 | 6-Pentyloxy-2-(4-propoxystyryl)quinoline | 79.41 | 81.50 | 79.30 | 0.11 |
| 12 | 4-Dodecyloxybenzylidene-4'-pentylaniline | 128.21 | 129.10 | 128.10 | 0.11 |
| 13 | 4-Bromo-N-(4-(4-methoxybenzoyloxy)benzylidene)aniline | 84.24 | 81.90 | 84.10 | 0.14 |
| 14 | 2-Heptanoyl-1,4-phenylene bis(4-nonyloxybenzoate) | 158.64 | 157.90 | 158.50 | 0.14 |
| 15 | 4-Methoxy-N-(4-pyridinylmethylene)-1,1-biphenyl-4'-amine | 78.27 | 80.30 | 78.10 | 0.17 |
| 16 | N-(4-Methoxybenzylidene)-4-hexylaniline | 91.19 | 90.10 | 91.00 | 0.19 |
| 17 | 3-(4-Decyloxyphenyl)-5-(4-methoxyphenyl)pyrazole | 110.81 | 110.50 | 110.60 | 0.21 |
| 18 | (1-Butoxycarbonyl)ethyl 5-(3-fluoro-4-decyloxybenzoyloxyphenylethynyl)-thiophene-2-carboxylate | 112.79 | 112.20 | 113.00 | -0.21 |
| 19 | 4-(2-(4-Pentylcyclohexyl)ethyl)-4'-nitrotolane | 71.22 | 68.90 | 71.00 | 0.22 |
| 20 | 3-Methylbutyl 4-(4-(4-chlorobenzoyloxy)benzylideneamino)benzoate | 80.17 | 80.40 | 80.40 | -0.23 |
| 21 | Bis(4-(4-(4-butyoxyphenyliminomethyl)phenoxy)butyl) malonate | 137.85 | 137.00 | 138.10 | -0.25 |
| 22 | 1-(2-(5-Butylthienyl))-3-(4-bromophenylamino)-2-propen-1-one | 58.65 | 56.40 | 58.90 | -0.25 |
| 23 | Phenyl 4-(4-ethoxyphenyliminomethyl)benzoate | 86.48 | 85.40 | 86.20 | 0.28 |
| 24 | Cholesteryl thiobutyrate | 64.10 | 64.40 | 63.80 | 0.30 |
| 25 | N-(4-Hexyloxybenzylidene)-4-aminobenzonitrile | 87.90 | 88.10 | 88.20 | -0.30 |
| 26 | 5alpha-Cholestan-3beta-ol hexanoate | 84.00 | 88.90 | 84.30 | -0.30 |
| 27 | 1,3-Phenylene bis(4-(4-octyloxyphenyliminomethyl)benzoate) | 140.02 | 138.90 | 139.70 | 0.32 |
| 28 | 1-(2-(5-Pentylthienyl))-3-(4-bromophenylamino)-2-propen-1-one | 60.87 | 60.40 | 61.20 | -0.33 |
| 29 | 2-Cyano-1,3-phenylene bis(4-(4'-hexadecylbiphenyl-4-carbonyloxy)-3-fluorobenzoate) | 189.69 | 189.60 | 189.30 | 0.39 |
| 30 | 4,6-Dichloro-1,3-phenylene bis(4-(4-octyloxy-3-fluorophenyliminomethyl)benzoate) | 127.39 | 126.50 | 127.00 | 0.39 |
| 31 | 4-Nonyl-N-(4-pentadecyloxyphenylmethylene)benzenamine | 158.70 | 157.40 | 158.30 | 0.40 |
| 32 | 2-(4-Heptyloxy)phenylazo-5-(2-heptyl)thien-5-yl-1,3,4-thiadiazole | 85.60 | 88.90 | 85.20 | 0.40 |
| 33 | Ethyl 4-(4-(4-cyanobenzoyloxy)benzylideneamino)benzoate | 79.78 | 79.90 | 80.20 | -0.42 |
| 34 | 4'-(2-Diethylaminoethoxy)biphenyl-4-yl 4-octyloxybenzoate | 24.17 | 24.40 | 24.60 | -0.43 |
| 35 | 2-Cyano-1,3-phenylene bis(4-(4'-decylbiphenyl-4-carbonyloxy)-2-fluorobenzoate) | 143.84 | 141.10 | 143.40 | 0.44 |
| 36 | 4-Methoxy-N-(2-pyridinylmethylene)-1,1-biphenyl-4'-amine | 52.96 | 54.90 | 53.40 | -0.44 |
| 37 | 3,3',4,4'-Biphenyltetracarboxy-N,N'-bis(heptylphenyl)diimide | 114.45 | 115.00 | 114.90 | -0.45 |
| 38 | 4-(4-Ethylphenyl)-2,6-bis(3,4,5-trioctyloxyphenyl)pyrimidine | 128.54 | 130.00 | 129.00 | -0.46 |
| 39 | 4-(4-(1-Methylheptyloxycarbonyl)phenyloxycarbonyl)phenyl 4-(6-(4-(4-decyloxybenzoyloxy)benzoyloxy)hexyloxy)benzoate | 154.64 | 155.40 | 155.10 | -0.46 |
| 40 | 1,5-Bis(4-(2-hydroxy-4-heptyloxybenzylideneamino)benzoyloxy)-2,2-dimethylpentane | 130.03 | 130.80 | 130.50 | -0.47 |
| 41 | 6-Pentyloxy-2-(4'-pentyloxyphenyliminomethyl)quinoline | 100.83 | 103.50 | 101.30 | -0.47 |
| 42 | 4-Pentanoyl-4'-dodecanoyloxyazobenzene | 137.20 | 137.20 | 137.70 | -0.50 |
| 43 | 4-(10-Undecenyloxy)-4'-trifluoromethyltolane | 88.76 | 86.00 | 88.20 | 0.56 |
| 44 | 3,3',4,4'-Biphenyltetracarboxy-N,N'-bis(pentylphenyl)diimide | 98.06 | 98.80 | 97.50 | 0.56 |
| 45 | 1,5-Bis(4-(2-hydroxy-4-heptyloxybenzylideneamino)benzoyloxy)-3,3-dimethylpentane | 131.77 | 130.80 | 131.20 | 0.57 |
| 46 | 4-Octanoyloxy-2,3-dimethyl-4'-ethoxyazobenzene | 94.63 | 96.10 | 95.20 | -0.57 |
| 47 | Methyl 5-((4'-decyloxybiphenyl-4-yl)oxycarbonyl)-1,3-dioxoisoindole-2-acetate | 99.40 | 97.40 | 98.80 | 0.60 |
| 48 | 1,3-Bis(4-butoxyazobenzene-4'-carbonyloxy)propane | 115.70 | 117.80 | 116.30 | -0.60 |
| 49 | 2,5-Thiophenediylbis(ethynediyl-4,1-phenylene) 3,4-bis(dodecyloxy)benzoate | 227.91 | 227.30 | 227.30 | 0.61 |
| 50 | 4-Ethoxyphenyl 4-(phenylmethyleneamino)benzoate | 85.05 | 85.40 | 85.70 | -0.65 |
| 51 | 1,4-Bis(4-propoxycarbonylphenyliminomethylene)benzene | 91.94 | 93.60 | 92.60 | -0.66 |
| 52 | Phenyl 4-(4-benzoyloxyphenylmethyleneimino)benzoate | 94.37 | 93.00 | 93.70 | 0.67 |
| 53 | 7-Hexyloxy-3-(4-decyloxyphenyl)-3H-1-benzopyran-4-one | 95.88 | 95.50 | 95.20 | 0.68 |
| 54 | N-(4-Dodecloxybenzylidene)-4-butylaniline | 125.52 | 125.10 | 126.20 | -0.68 |
| 55 | 2-(5-(4-Hexyloxyphenyl)-1,3,4-thiadiazol-2-yliminomethyl)-5-(octyloxy)phenol | 75.22 | 73.90 | 74.50 | 0.72 |
| 56 | 3,4-Dimethyl-N-(4-(4-methoxybenzoyloxy)benzylidene)aniline | 67.56 | 67.50 | 68.30 | -0.74 |
| 57 | N-(4-Hexyloxybenzylidene)-4-octylaniline | 117.26 | 117.00 | 118.00 | -0.74 |
| 58 | 4-(3-(4-(Hexyloxy)phenyl)-1-oxo-2-propenyloxy)phenyl 2-chloro-4-methylpentanoate | 81.86 | 83.20 | 82.60 | -0.74 |
| 59 | 4-Pentylphenyl 4'-heptyloxythiobenzoate | 101.95 | 101.30 | 101.20 | 0.75 |
| 60 | Phenyl 4-((4-methoxyphenyliminomethyl)phenyl benzoate | 81.95 | 82.70 | 82.70 | -0.75 |
| 61 | 4-((4-Methoxyphenylimino)methyl)phenyl benzoate | 82.15 | 82.70 | 82.90 | -0.75 |
| 62 | 5,5'''-Didecylquaterthiophene | 120.97 | 134.90 | 120.20 | 0.77 |
| 63 | Cholest-5-en-3-ol | 73.42 | 74.50 | 74.20 | -0.78 |
| 64 | 4-Methoxyphenyl 4-(4-(4-cyanophenyliminomethyl))benzoate | 76.53 | 74.50 | 75.70 | 0.83 |
| 65 | 3,5-Di(4-octyloxyphenyl)pyrazole | 129.06 | 129.30 | 129.90 | -0.84 |
| 66 | 4-(Phenylmethyleneamino)phenyl 4-(phenylazo)benzoate | 94.75 | 93.70 | 93.90 | 0.85 |
| 67 | 4'-(2-(4-Propylphenyl)-1-ethynyl)-2'-methyl-4-propyltolane | 55.17 | 54.70 | 54.30 | 0.87 |
| 68 | N,N'-Diundecylbenzene-1,2-dicarboxamide | 167.42 | 168.20 | 168.30 | -0.88 |
| 69 | 6-Decyloxy-2-(4-nonyloxybenzylidenamino)benzothiazole | 120.41 | 123.20 | 121.30 | -0.89 |
| 70 | Di(4'-Ethylphenyl)-cyclohexane-1,4-dicarboxylate | 80.30 | 78.90 | 79.40 | 0.90 |
| 71 | 4-(2-(4-(11-Hydroxyundecyloxy)phenyl)vinyl)benzonitrile | 108.74 | 110.00 | 107.80 | 0.94 |
| 72 | 4-Bromo-4'-(3-butenyloxy)biphenyl | 80.84 | 82.00 | 79.90 | 0.94 |
| 73 | Butyl 4-(4-(4-Chlorobenzoyloxy)benzylideneamino)benzoate | 87.85 | 89.00 | 88.80 | -0.95 |
| 74 | Di(4'-pentylphenyl)-cyclohexane-1,4-dicarboxylate | 105.86 | 103.20 | 104.90 | 0.96 |
| 75 | 4-Octyloxyphenyl 4-hexyloxythiobenzoate | 111.37 | 110.60 | 110.40 | 0.97 |
| 76 | 4-(Phenylazo)phenyl biphenyl-4-carboxylate | 84.90 | 85.80 | 85.90 | -1.00 |
| 77 | 4-Pentylphenyl 4'-octyloxythiobenzoate | 103.89 | 105.30 | 104.90 | -1.01 |
| 78 | Bis(4-(4-hexadecyloxybenzylideneamino)phenyl) hexanedioate | 226.79 | 225.40 | 227.80 | -1.01 |
| 79 | 4-(4-Heptyloxyphenyl)acetylene-2,3,5,6-tetrafluorophenyl 4'-chlorobenzoate | 90.83 | 90.70 | 91.90 | -1.07 |
| 80 | 4-((Phenylmethylene)amino)phenyl 4-methoxybenzoate | 81.41 | 82.70 | 82.50 | -1.09 |
| 81 | 3-Pyridyl 4-(4-hexyloxybenzylideneamino)benzoate | 110.31 | 110.00 | 109.20 | 1.11 |
| 82 | Bis(4-(4-decyloxybenzylideneamino)phenyl decanedioate | 194.11 | 193.10 | 193.00 | 1.11 |
| 83 | 4,4'-Dioctyloxyazoxybenzene | 126.47 | 127.00 | 127.60 | -1.13 |
| 84 | N-(4-Propoxybenzylidene)-4-butylaniline | 88.16 | 88.70 | 89.30 | -1.14 |
| 85 | 1-(4-(4-Formyl)phenylazophenyl)-4-(1-oxodecyl)piperazine | 51.34 | 52.20 | 52.50 | -1.16 |
| 86 | N-(7-Oxo-4-(4-(1-hexadecanoylamino)phenyl)-1,3,5-cycloheptatrien-1-yl)dodecamide | 127.74 | 125.70 | 128.90 | -1.16 |
| 87 | 1-(2-(5-Pentylthienyl))-3-(4-chlorophenylamino)-2-propen-1-one | 53.92 | 54.00 | 55.10 | -1.18 |
| 88 | 1-Methylheptyl 4-(4-(4-hexyloxycinnamoyloxy)benzoyloxy)benzoate | 102.81 | 104.50 | 101.60 | 1.21 |
| 89 | 4,4'-Bis(7-(4'-(1-methylheptyloxycarbonyl)biphenyl-4-yl)oxycarbonyl)heptyloxy)biphenyl | 145.88 | 148.50 | 147.10 | -1.22 |
| 90 | 4-Dodecyloxy-3'-stilbazole N-oxide | 100.08 | 101.00 | 101.30 | -1.22 |
| 91 | 4-Pentyl-4'-octadecyloxyazobenzene | 155.25 | 157.10 | 156.50 | -1.25 |
| 92 | N-(4-Methoxybenzylidene)-4-octylaniline | 97.15 | 98.20 | 98.40 | -1.25 |
| 93 | 4-(3-Pyridiniminomethyl)phenyl 4-propoxybenzoate | 99.57 | 97.90 | 98.30 | 1.27 |
| 94 | 1-(2-(5-Chlorothienyl))-3-(4-methoxyphenylamino)-2-propen-1-one | 54.62 | 55.60 | 55.90 | -1.28 |
| 95 | 1-Methylheptyl 5-(3-fluoro-4-hexadecyloxybenzoyloxyphenylethynyl)-thiophene-2-carboxylate | 140.61 | 140.70 | 141.90 | -1.29 |
| 96 | 1-Methylheptyl 4'-(2-fluoro-4-tetradecyloxyphenylpropiolyloxy)biphenyl-4-carboxylate | 128.01 | 129.10 | 129.30 | -1.29 |
| 97 | 4,4'''-Didecyloxy-p-quaterphenyl | 125.00 | 123.10 | 123.70 | 1.30 |
| 98 | 4-Isopropylphenyl 4-(4-hexyloxybenzylideneamino)benzoate | 87.10 | 89.40 | 88.40 | -1.30 |
| 99 | Cholesteryl thiopentanoate | 66.10 | 68.50 | 67.40 | -1.30 |
| 100 | 4'-(2-Diethylaminoethoxy)biphenyl-4-yl 4-dodecyloxybenzoate | 41.82 | 40.60 | 40.50 | 1.32 |
| 101 | 1,3-Phenylene bis(4-(4'-hexylbiphenyl-4-carbonyloxy)-2-ethylbenzoate) | 106.04 | 107.80 | 107.40 | -1.36 |
| 102 | 4-(Phenyliminomethyl)phenyl 4-(phenylazo)benzoate | 95.47 | 93.70 | 94.10 | 1.37 |
| 103 | Cholesteryl thiononadecanoate | 126.10 | 125.00 | 124.70 | 1.40 |
| 104 | 4-(4-Butylcyclohexyl)-4'-nitrotolane | 59.61 | 56.70 | 58.20 | 1.41 |
| 105 | 2-Acetyl-1,4-phenylene bis(4-butoxybenzoate) | 95.48 | 97.30 | 96.90 | -1.42 |
| 106 | Bis(4-(3-butoxy-3-oxo-1-propenyl)phenyl terephthalate | 91.48 | 95.70 | 92.90 | -1.42 |
| 107 | 4-Decyloxybiphenyl-4'-yl 4-(1-methylheptyloxy)benzoate | 115.23 | 113.00 | 113.80 | 1.43 |
| 108 | Cholesteryl beta-bromopropionate | 56.73 | 55.30 |  | 1.43 |
| 109 | 1,3-Phenylene bis(4-(4'-octylbiphenyl-4-carbonyloxy)-2-methylbenzoate) | 116.07 | 115.90 | 117.50 | -1.43 |
| 110 | 4'-(2-(4-Butylphenyl)-1-ethynyl)-2'-methyl-4-ethyltolane | 52.84 | 54.70 | 54.30 | -1.46 |
| 111 | Phenyl 4-(4-ethoxybenzylideneamino)benzoate | 84.01 | 85.40 | 85.50 | -1.49 |
| 112 | 4-(4-Octadecyloxyphenylazoxy)phenyl 2-chloro-3-methylpentanoate | 130.60 | 135.40 | 132.10 | -1.50 |
| 113 | 4-Ethylphenyl 4-(4-propoxybenzylideneamino)benzoate | 85.00 | 85.90 | 86.50 | -1.50 |
| 114 | 2-Cyano-1,3-phenylene bis(4-(4'-nonylbiphenyl-4-carbonyloxy)-2-fluorobenzoate) | 130.69 | 133.00 | 132.20 | -1.51 |
| 115 | Cholesteryl 6-(4-pentyloxybiphenyl-4'-oxy)hexanoate | 97.12 | 94.20 | 95.60 | 1.52 |
| 116 | 2-Methylpentyl 4-(4-(4-chlorobenzoyloxy)benzylideneamino)benzoate | 85.94 | 84.50 | 84.40 | 1.54 |
| 117 | 4-(omega-Aminoheptyloxy)-4'-cyanobiphenyl | 63.96 | 65.10 | 65.50 | -1.54 |
| 118 | 1,3-Phenylene bis(4-(4-tridecyloxybenzoylthio)benzoate) | 164.95 | 164.10 | 163.40 | 1.55 |
| 119 | 1-Methylheptyl 4-(4-(3-fluoro-4-nonyloxybenzoyloxy)-3-fluorobenzoyloxy)benzoate | 124.50 | 124.60 | 122.90 | 1.60 |
| 120 | 4-((3-Pyridinimino)methyl)phenyl 4-ethoxybenzoate | 96.00 | 93.80 | 94.40 | 1.60 |
| 121 | Cholesteryl 5-phenylpentanoate | 84.47 | 85.00 | 86.10 | -1.63 |
| 122 | 4,4'-Bis(2-(4-propylcyclohexyl)ethyl)biphenyl | 68.66 | 71.00 | 70.30 | -1.64 |
| 123 | 5-(4-Octyloxyphenyl)-N-(4-(2-methylbutoxy)phenylmethylene)-1,3,4-thiadiazol-2-amine | 66.04 | 66.20 | 67.70 | -1.66 |
| 124 | 1,4-Bis(4-butoxyazobenzene-4'-carbonyloxy)butane | 118.72 | 121.80 | 120.40 | -1.68 |
| 125 | 4,4'''-Dinonyloxy-p-quaterphenyl | 116.00 | 115.10 | 114.30 | 1.70 |
| 126 | Hexyl 4-(4-(4-(1-oxo-10-undecenyl)-1-piperazinyl)phenylazo)benzoate | 69.80 | 72.80 | 71.50 | -1.70 |
| 127 | Cholesteryl thiooctanoate | 80.10 | 80.60 | 81.80 | -1.70 |
| 128 | 2-(Perfluorohexyl)ethyl 4-nitrothiobenzoate | 91.24 | 90.20 | 89.50 | 1.74 |
| 129 | Bis(4-(5-pentylthio-1,3,4-oxadiazole-2-yl)phenyl)-2,5-thiophenedicarboxylate | 78.12 | 77.10 | 79.90 | -1.78 |
| 130 | 1-(4-Dodecylbiphenyl-4'-yl)-3-(3-chlorophenyl)propane-1,3-dione | 105.00 | 108.20 | 106.80 | -1.80 |
| 131 | 4-Butyl-4'-nonyloxyazobenzene | 118.02 | 116.60 | 116.20 | 1.82 |
| 132 | 4-(omega-Aminopentyloxy)-4'-cyanobiphenyl | 55.57 | 57.10 | 57.40 | -1.83 |
| 133 | 4-(2-(4-(5-Vinyloxypentyloxy)phenyl)vinyl)benzonitrile | 74.75 | 77.30 | 72.90 | 1.85 |
| 134 | 2-Methylpentyl 4-(4-(4-methylbenzoyloxy)benzylideneamino)benzoate | 86.26 | 84.10 | 84.40 | 1.86 |
| 135 | 4'-(2-Diethylaminopropoxy)biphenyl-4-yl 4-undecyloxybenzoate | 38.92 | 40.60 | 40.80 | -1.88 |
| 136 | 4-(4-Octyloxybenzylideneamino)phenyl benzoate | 111.49 | 109.60 | 109.60 | 1.89 |
| 137 | 1,2-Dimethylpropyl 4-(4-(4-methoxybenzoyloxy)benzylideneamino)benzoate | 66.09 | 63.90 | 64.20 | 1.89 |
| 138 | 5-(4-Pentyloxyphenyl)-2-(4-octyloxy)phenylamido-1,3,4-thiadiazole | 56.89 | 60.50 | 58.80 | -1.91 |
| 139 | 4-Pentylphenyl 4'-dodecyloxythiobenzoate | 120.99 | 121.50 | 122.90 | -1.91 |
| 140 | 2-(4-Heptyloxyphenyl)-5-(4-decyloxy)phenylazo-1,3,4-thiadiazole | 100.82 | 98.70 | 98.90 | 1.92 |
| 141 | 4-Fluoro-N-(4-(4-methoxybenzoyloxy)benzylidene)aniline | 80.68 | 83.70 | 82.60 | -1.92 |
| 142 | 4-Thiocyanophenyl 4-(4-nonyloxybenzoyloxy)benzoate | 102.36 | 106.00 | 104.30 | -1.94 |
| 143 | Cholesteryl 8-(2,3-difluoro-4-decyloxybiphenyl-4'-oxy)octanoate | 123.52 | 124.50 | 125.50 | -1.98 |
| 144 | 2-Methylhexyl 4-(4-(4-methoxybenzoyloxy)benzylideneamino)benzoate | 92.70 | 90.70 | 90.70 | 2.00 |
| 145 | N-(4-Hexyloxybenzylidene)-4-butylaniline | 103.22 | 100.90 | 101.20 | 2.02 |
| 146 | 7-Decyloxy-3-(4-decyloxyphenyl)-3H-1-benzopyran-4-one | 111.87 | 111.70 | 113.90 | -2.03 |
| 147 | 4-(3-Pyridylmethyleneamino)phenyl 4-hexyloxybenzoate | 111.15 | 110.00 | 109.10 | 2.05 |
| 148 | 1-(4-Dodecylbiphenyl-4'-yl)-3-(2-pentyloxyphenyl)propane-1,3-dione | 125.45 | 125.20 | 123.40 | 2.05 |
| 149 | (1-Butoxycarbonyl)ethyl 5-(3-fluoro-4-tridecyloxybenzoyloxyphenylethynyl)-thiophene-2-carboxylate | 121.35 | 124.40 | 123.40 | -2.05 |
| 150 | 4-(Benzoyloxy)phenyl 4-(phenyliminomethyl)benzoate | 91.85 | 93.00 | 93.90 | -2.05 |
| 151 | 4-Methyl-2'-hydroxy-4'-octyloxyazobenzene | 90.52 | 91.50 | 92.60 | -2.08 |
| 152 | 4-Octyloxy-4'-stilbazole N-oxide | 82.21 | 84.90 | 84.30 | -2.09 |
| 153 | 4,6-Dichloro-1,3-phenylene bis(4-(4-decyloxy-3-fluorophenyliminomethyl)benzoate) | 144.91 | 142.60 | 142.80 | 2.11 |
| 154 | 3-Methylpentyl 4-(4-(4-methylbenzoyloxy)benzylideneamino)benzoate | 87.63 | 84.10 | 85.50 | 2.13 |
| 155 | 1-Methylpropyl 4-(4-(4-acetoxybenzoyloxy)benzylideneamino)benzoate | 83.24 | 80.80 | 81.10 | 2.14 |
| 156 | 4-(4-Pyridylmethyleneamino)phenyl 4-butoxybenzoate | 100.26 | 101.90 | 102.40 | -2.14 |
| 157 | 4-Chloro-2'-hydroxy-4'-pentyloxyazobenzene | 81.88 | 79.80 | 79.70 | 2.18 |
| 158 | 3-(4-(4-Heptylcyclohexyl)phenyl)-5-(4-methoxyphenyl)isoxazole | 63.12 | 65.50 | 65.30 | -2.18 |
| 159 | 4-(2-(4-Butylcyclohexyl)ethyl)-4'-nitrotolane | 63.21 | 64.80 | 65.40 | -2.19 |
| 160 | 1-Methylheptyl 4'-(4''-eicosyloxy- 2'',3''-difluorobenzoyloxy)tolan-4-carboxylate | 158.00 | 154.30 | 155.80 | 2.20 |
| 161 | 4-(2-Methylbutoxy)phenyl 4-(4-octyloxy-2,3,5,6-tetrafluorophenylethynyl)benzoate | 99.18 | 99.10 | 101.40 | -2.22 |
| 162 | 1-(2-(5-Butylthienyl))-3-(4-chlorophenylamino)-2-propen-1-one | 56.03 | 49.90 | 53.80 | 2.23 |
| 163 | 4-(Phenoxycarbonyl)phenyl 4-(benzoyloxy)benzoate | 98.64 | 96.00 | 96.40 | 2.24 |
| 164 | 3-Pyridyl 4-((4-methoxybenzylidene)amino) benzoate | 93.74 | 91.20 | 91.50 | 2.24 |
| 165 | 4-(3-Ethylmercapto-2-methylpropionyloxy)phenyl 4'-dodecyloxybiphenyl-4-carboxylate | 116.74 | 113.50 | 114.50 | 2.24 |
| 166 | 4-Methyl-2'-hydroxy-4'-nonyloxyazobenzene | 96.25 | 95.50 | 94.00 | 2.25 |
| 167 | 7-Propoxy-3-(4-decyloxyphenyl)-3H-1-benzopyran-4-one | 86.75 | 83.40 | 84.50 | 2.25 |
| 168 | 4-(3-(4-(Dodecyloxy)phenyl)-1-oxo-2-propenyloxy)phenyl 2-chloro-4-methylpentanoate | 105.64 | 107.50 | 107.90 | -2.26 |
| 169 | N-(2-(6-Decyloxy)benzothiazolyl)-4-nonyloxybenzamide | 111.49 | 104.80 | 109.20 | 2.29 |
| 170 | Cholesteryl thiohexanoate | 71.20 | 72.50 | 73.50 | -2.30 |
| 171 | 1,1-Dimethylpropyl 4-(4-(4-methoxybenzoyloxy)benzylideneamino)benzoate | 112.43 | 109.80 | 110.10 | 2.33 |
| 172 | 4-Nonyloxybenzoic acid | 116.96 | 117.80 | 119.30 | -2.34 |
| 173 | 2,2-Dimethylpropyl 4-(4-(4-nitrobenzoyloxy)benzylideneamino)benzoate | 91.07 | 90.00 | 88.70 | 2.37 |
| 174 | 4,6-Dichloro-1,3-phenylene bis(4-(4-undecyloxy-3-fluorophenyliminomethyl)benzoate) | 152.69 | 150.70 | 150.30 | 2.39 |
| 175 | 1,3-Bis(4-(4-hexyloxybenzoyloxy)benzylideneamino)benzene | 125.49 | 122.80 | 123.10 | 2.39 |
| 176 | 4-Tetradecyloxy-3-nitrobenzoic acid | 131.61 | 129.70 | 129.20 | 2.41 |
| 177 | 4-(Phenoxycarbonyl)phenyl 4-(phenyliminomethyl)benzoate | 90.69 | 93.00 | 93.10 | -2.41 |
| 178 | 6-Decyloxy-2-(4-octyloxybenzylidenamino)benzothiazole | 122.12 | 119.10 | 119.70 | 2.42 |
| 179 | 4-Hexyl-N-(4-pentadecyloxyphenylmethylene)benzenamine | 149.15 | 145.30 | 146.70 | 2.45 |
| 180 | 1,3-Phenylene bis(4-(4'-decylbiphenyl-4-carbonyloxy)benzoate) | 148.26 | 147.30 | 145.80 | 2.46 |
| 181 | 1-Methylheptyl 4-(4-(4-tetradecyloxy-alpha-methylcinnamoyloxy)benzoyloxy)benzoate | 133.78 | 131.90 | 131.30 | 2.48 |
| 182 | 4,4'''-Dipentyloxy-p-quaterphenyl | 81.01 | 82.70 | 83.50 | -2.49 |
| 183 | 1-(4-Ethylcyclohexylethynyl)-4-(4-butylcyclohexylethynyl)benzene | 49.01 | 53.00 | 51.50 | -2.49 |
| 184 | Cholesteryl thiohexadecanoate | 116.20 | 112.90 | 113.70 | 2.50 |
| 185 | Pyridine-2,6-diylbis(1,4-phenylene) bis(4-(4-nonylbenzoyloxy)benzoate) | 100.50 | 96.80 | 98.00 | 2.50 |
| 186 | Butyl 4-(4-(4-acetoxybenzoyloxy)benzylideneamino)benzoate | 97.87 | 99.50 | 100.40 | -2.53 |
| 187 | 4-(4-Ethoxybenzylidene)-ethoxyaniline-N-oxide | 100.16 | 102.90 | 102.70 | -2.54 |
| 188 | Butyl 2-cyano-3-(4-(4-decanoyl-1-piperazinyl)phenylazophenyl)-2-propenoate | 46.16 | 46.80 | 48.70 | -2.54 |
| 189 | N-(4-Butoxybenzylidene)-4-aminobenzonitrile | 77.63 | 80.00 | 80.20 | -2.57 |
| 190 | N-(4-Propoxybenzylidene)-4-heptylaniline | 98.63 | 100.90 | 101.20 | -2.57 |
| 191 | 5-(4-Pyridyl)-2-(4-hexyloxy)phenylamino-1,3,4-thiadiazole | 51.39 | 48.80 | 48.80 | 2.59 |
| 192 | 6-S-Tetradecyl-6-thio-alpha-D-galactopyranose | 118.00 | 116.50 | 115.40 | 2.60 |
| 193 | 4-(4'-Hexylbicyclohexyl)benzene-isothiocyanate | 86.10 | 84.70 | 83.50 | 2.60 |
| 194 | Bis(4-pentyloxyphenyl) terephthalate | 106.97 | 110.20 | 109.60 | -2.63 |
| 195 | 4-Hexanoyloxy-2,3-dimethyl-4'-ethoxyazobenzene | 92.74 | 88.10 | 90.10 | 2.64 |
| 196 | 4-Chloro-2'-hydroxy-4'-decyloxyazobenzene | 96.93 | 100.00 | 99.60 | -2.67 |
| 197 | Cholesteryl benzoate | 70.18 | 68.80 | 67.50 | 2.68 |
| 198 | 2,2-Dimethylpropyl 4-(4-(4-chlorobenzoyloxy)benzylideneamino)benzoate | 95.59 | 91.00 | 92.90 | 2.69 |
| 199 | Cholesteryl thiotridecanoate | 104.00 | 100.80 | 101.30 | 2.70 |
| 200 | Bis(4-(2-methylbutoxycarbonyl)phenyl) terephthalate | 88.48 | 90.50 | 91.20 | -2.72 |
| 201 | 5-Chloro-2-(4-n-tridecafluorohexylphenyl)isoindole-1,3-dione | 81.80 | 78.80 | 79.00 | 2.80 |
| 202 | 4-(4'-Pentyloxy)biphenyl 5-methoxy-2-thiophenecarboxylate | 218.01 | 216.50 | 215.20 | 2.81 |
| 203 | 4-Ethoxyphenyl 4-(phenyliminomethyl)benzoate | 88.62 | 85.40 | 85.80 | 2.82 |
| 204 | Di(4'-propylphenyl) cyclohexane-1,4-dicarboxylate | 91.62 | 87.00 | 88.80 | 2.82 |
| 205 | (1-Butoxycarbonyl)ethyl 5-(3-fluoro-4-dodecyloxybenzoyloxyphenylethynyl)-thiophene-2-carboxylate | 122.53 | 120.30 | 119.70 | 2.83 |
| 206 | 1-Methylheptyl 5-(4-octyloxybenzoyloxyphenylethynyl)-thiophene-2-carboxylate | 110.03 | 107.30 | 107.20 | 2.83 |
| 207 | 2-Fluoro-4-octyloxyphenyl 4-hexyloxythiobenzoate | 108.37 | 111.60 | 111.20 | -2.83 |
| 208 | 1-(4-Undecylbiphenyl-4'-yl)-3-(4-chlorophenyl)propane-1,3-dione | 101.55 | 104.20 | 104.40 | -2.85 |
| 209 | 4-Propylcyclohexyl 4-methoxycinnamate | 59.79 | 57.30 | 56.90 | 2.89 |
| 210 | 1-Methylpentyl 4-(4-(4-methoxybenzoyloxy)benzylideneamino)benzoate | 83.40 | 80.60 | 80.50 | 2.90 |
| 211 | 4-((4-Methoxyphenyl)methyleneamino)phenyl benzoate | 85.47 | 82.70 | 82.50 | 2.97 |
| 212 | 4-(4-(4-Butoxyphenyliminomethyl)-3-(hydroxyphenoxy)butylmethacrylate | 115.97 | 112.30 | 113.00 | 2.97 |
| 213 | 1-(4'-Pentylbicyclohexyl)-3,4-difluorobenzene | 68.73 | 70.20 | 71.70 | -2.97 |
| 214 | 4-(3-(4-(Nonyloxy)phenyl)-1-oxo-2-propenyloxy)phenyl 2-chloro-3-methylpentanoate | 93.12 | 95.30 | 96.10 | -2.98 |
| 215 | 4'-Dodecyloxy-3'-nitrobiphenyl-4-carboxylic acid | 119.09 | 116.00 | 116.10 | 2.99 |
| 216 | 4-Methoxyphenyl 4-(4-(4-fluorophenylimino)methyl)benzoate | 87.41 | 83.70 | 84.40 | 3.01 |
| 217 | 1,3-Phenylene bis(4-(4'-tetradecylbiphenyl-4-carbonyloxy)benzoate) | 181.25 | 179.60 | 178.20 | 3.05 |
| 218 | cholesteryl 4-phenylbutyrate | 77.45 | 80.90 | 80.50 | -3.05 |
| 219 | 4-Pentyloxyphenyl 7-decanoyloxychromone-2-carboxylate | 67.76 | 67.20 | 64.70 | 3.06 |
| 220 | 4-(3,3,4,4,5,5,6,6,7,7,8,8,8-Tridecafluorooctyloxycarbonyl)phenyl 4-((perfluorobutyl)propoxy)benzoate | 102.43 | 105.70 | 105.50 | -3.07 |
| 221 | 4-(3-Ethylmercapto-2-methylpropionyloxy)phenyl 4'-tetradecyloxybiphenyl-4-carboxylate | 121.68 | 121.60 | 118.60 | 3.08 |
| 222 | 1,5-Bis(4-(2-hydroxy-4-octyloxybenzylideneamino)benzoyloxy)-3,3-dimethylpentane | 135.52 | 138.90 | 138.60 | -3.08 |
| 223 | Bis(4-(3-hexyloxy-3-oxo-1-propenyl)phenyl terephthalate | 115.99 | 111.90 | 112.90 | 3.09 |
| 224 | 5-(4-Nonyloxyphenyl)-N-(4-(2-methylbutoxy)phenylmethylene)-1,3,4-thiadiazol-2-amine | 65.21 | 70.30 | 68.30 | -3.09 |
| 225 | 4-Hydroxybutyl 4-((4'-octyloxybiphenyl-4-yl)oxymethyl)benzoate | 116.31 | 120.00 | 119.40 | -3.09 |
| 226 | 4-(3-Decyloxyphenyliminomethyl)phenyl 4-decyloxybenzoate | 144.71 | 147.60 | 147.80 | -3.09 |
| 227 | 4-(2-Ethylheptyloxycarbonyl)phenyl 4-dodecanoyloxytolane-4'-carboxylate | 132.11 | 135.80 | 135.20 | -3.09 |
| 228 | 4'-Pentadecyloxy-3'-nitrobiphenyl-4-carboxylic acid | 134.61 | 128.10 | 131.50 | 3.11 |
| 229 | Isopropyl 4-(4-(4-acetoxybenzoyloxy)benzylideneamino)benzoate | 74.19 | 76.80 | 77.30 | -3.11 |
| 230 | 4-((4-cyanophenyl)methyleneamino)phenyl benzoate | 83.72 | 79.60 | 80.60 | 3.12 |
| 231 | 2-Methyl-1,3-phenylene bis(4-(3-chloro-4-undecyloxyphenylethenyl)benzoate) | 110.58 | 113.30 | 113.70 | -3.12 |
| 232 | 4-Chlorophenyl 4-decyloxythiobenzoate | 101.78 | 97.70 | 98.60 | 3.18 |
| 233 | Butyl 4-(4-(4-methylbenzoyloxy)benzylideneamino)benzoate | 91.28 | 88.60 | 88.10 | 3.18 |
| 234 | Phenyl 4-(4-benzoyloxyphenyliminomethyl)benzoate | 96.30 | 93.00 | 93.10 | 3.20 |
| 235 | N-(4-Ethoxybenzylidene)-4-octylaniline | 98.48 | 100.90 | 101.70 | -3.22 |
| 236 | 5-(4-Hexyloxyphenyl)-N-(4-(2-methylbutoxy)phenyl)methylene-1,3,4-thiadiazol-2-amine | 54.67 | 58.20 | 57.90 | -3.23 |
| 237 | 4-(2-(4-(7-Vinyloxyheptyloxy)phenyl)vinyl)benzonitrile | 87.25 | 85.30 | 84.00 | 3.25 |
| 238 | 4-(3,3,4,4,5,5,6,6,7,7,8,8,8-Tridecafluorooctyloxycarbonyl)phenyl 4-(hexyloxy)benzoate | 108.45 | 113.40 | 111.70 | -3.25 |
| 239 | 1,3-Phenylene bis(4-(4'-heptylbiphenyl-4-carbonyloxy)-2-fluorobenzoate) | 120.62 | 125.10 | 123.90 | -3.28 |
| 240 | Di-(4-butoxycarbonylphenyl) terephthalate | 105.02 | 107.60 | 108.30 | -3.28 |
| 241 | 4-Methyl-2'-hydroxy-4'-decyloxyazobenzene | 96.40 | 99.60 | 99.70 | -3.30 |
| 242 | 4-(4-Ethoxyphenylazo)phenyl hexanoate | 106.42 | 103.30 | 103.10 | 3.32 |
| 243 | 4-Methyl-2'-hydroxy-4'-pentyloxyazobenzene | 84.32 | 79.40 | 81.00 | 3.32 |
| 244 | 4-Nonyloxyphenyl 3-fluoro-4-thiocyanatophenyl terephthalate | 112.24 | 107.00 | 108.90 | 3.34 |
| 245 | 1,3-Phenylene bis(4-(4'-octylbiphenyl-4-carbonyloxy)benzoate) | 132.40 | 131.20 | 129.00 | 3.40 |
| 246 | 2-Octanoyl-5-(4-ethoxyphenyl)thiophene | 94.01 | 95.20 | 90.60 | 3.41 |
| 247 | 4-(4-Butylcyclohexyl)isothiocyanatobenzene | 83.49 | 87.00 | 86.90 | -3.41 |
| 248 | 2-Cyano-1,3-phenylene bis(4-(4'-decylbiphenyl-4-carbonyloxy)-3-fluorobenzoate) | 138.18 | 141.10 | 141.60 | -3.42 |
| 249 | 2-(4-Hexyloxyphenylazo)-5-(5'-heptyl-2'-thienyl)-1,3,4-thiadiazole | 81.68 | 84.90 | 85.10 | -3.42 |
| 250 | 4'-(2-(4-Ethylphenyl)-1-ethynyl)-2'-methyl-4-hexyltolane | 64.43 | 62.80 | 61.00 | 3.43 |
| 251 | 2-(4-Butoxyphenyl)-5-(4-(2-methyl-2,3-undecadienyloxy)phenyl-1,3,4-thiadiazole | 88.84 | 85.60 | 85.40 | 3.44 |
| 252 | 5-(4-Hexyloxyphenyl)-2-(4-octyloxy)phenylamido-1,3,4-thiadiazole | 66.15 | 64.50 | 62.70 | 3.45 |
| 253 | 4'-Eicosyloxy-3'-nitrobiphenyl-4-carboxylic acid | 145.64 | 148.30 | 149.10 | -3.46 |
| 254 | 1,2-Bis-5-(4-(5-undecyl-1,3,4-thiadiazol-2-yl)phenoxy)pentanoyloxy)-8-(4'-cyanobiphenyl-4-yloxy)octane | 216.87 | 210.10 | 213.40 | 3.47 |
| 255 | 4-(3-(4-(Decyloxy)phenyl)-1-oxo-2-propenyloxy)phenyl 2-chloro-3-methylpentanoate | 105.08 | 99.40 | 101.60 | 3.48 |
| 256 | 4-Propanoyl-4'-octanoyloxyazobenzene | 116.79 | 113.00 | 113.30 | 3.49 |
| 257 | N,N'-Didecanoyl-3,4,5,6-tetramethylbenzene-1,2-diamine | 83.49 | 78.50 | 80.00 | 3.49 |
| 258 | 1-(6-Octyloxy-2-naphthyl)-4-(3,4-difluorophenyl)diacetylene | 75.60 | 80.90 | 79.10 | -3.50 |
| 259 | Propane-1,3-diyl bis(4-(4'-octyloxybiphenyl-4-yl)oxymethyl)benzoate) | 128.11 | 126.00 | 124.60 | 3.51 |
| 260 | 6-Decyloxy-2-(4-pentyloxybenzylidenamino)benzothiazole | 105.09 | 107.00 | 108.60 | -3.51 |
| 261 | 4-(4'-Butylbicyclohexyl)benzene-isothiocyanate | 73.29 | 76.70 | 76.80 | -3.51 |
| 262 | 7-Nonyloxy-3-(4-decyloxyphenyl)-3H-1-benzopyran-4-one | 106.78 | 107.60 | 110.30 | -3.52 |
| 263 | 2-(5-(4-Heptyloxyphenyl)-1,3,4-thiadiazol-2-yliminomethyl)-5-(octyloxy)phenol | 83.13 | 77.90 | 79.60 | 3.53 |
| 264 | Bis(4-(4-decyloxyphenyliminomethyl)phenyl) heptanedioate | 176.45 | 181.00 | 180.00 | -3.55 |
| 265 | 4-(3-Ethylmercapto-2-methylpropionyloxy)phenyl 4'-undecyloxybiphenyl-4-carboxylate | 105.14 | 109.50 | 108.70 | -3.56 |
| 266 | 4-(4-Ethoxyphenyliminomethyl)phenyl benozate | 89.77 | 85.40 | 86.20 | 3.57 |
| 267 | 1-Methylheptyl 5-(3-fluoro-4-dodecyloxybenzoyloxyphenylethynyl)-thiophene-2-carboxylate | 120.83 | 124.50 | 124.40 | -3.57 |
| 268 | 4-Cyanophenyl 4-(3-(4-(4'-decylbiphenyl-4-carbonyloxy)-2-fluorobenzoyloxy)benzoyloxy)benzoate | 112.41 | 117.00 | 116.00 | -3.59 |
| 269 | 4-(4-Cyanophenyliminomethyl)phenyl heptanoate | 93.31 | 97.80 | 96.90 | -3.59 |
| 270 | 4-(3-Pyridylmethylene)amino)phenyl 4-methoxybenzoate | 88.10 | 91.20 | 91.70 | -3.60 |
| 271 | Bis(4-ethoxycarbonylphenyl) terephthalate | 95.11 | 91.40 | 91.50 | 3.61 |
| 272 | Bis(4-(3-pentyloxy-3-oxo-1-propenyl)phenyl terephthalate | 100.69 | 103.80 | 104.30 | -3.61 |
| 273 | 4-(Phenylmethyleneamino)phenyl 4-ethoxybenzoate | 81.48 | 85.40 | 85.10 | -3.62 |
| 274 | 2-Cyano-1,3-phenylene bis(4-(4-tetradecyloxyphenyliminomethyl)benzoate) | 175.24 | 179.20 | 178.90 | -3.66 |
| 275 | 5-(4-Pyridyl)-2-(4-heptyloxy)phenylamino-1,3,4-thiadiazole | 57.08 | 54.00 | 53.40 | 3.68 |
| 276 | 1,7-Bis(4-(4-octyloxybenzoyloxy)benzylideneamino)heptane | 173.99 | 169.60 | 170.30 | 3.69 |
| 277 | 4-Heptyloxybiphenylyl 4-(1-methylheptyloxy)thiobenzoate | 93.60 | 90.30 | 89.90 | 3.70 |
| 278 | (1-Butoxycarbonyl)ethyl 5-(3-fluoro-4-octyloxybenzoyloxyphenylethynyl)-thiophene-2-carboxylate | 100.30 | 104.20 | 104.00 | -3.70 |
| 279 | 4-Hydroxypentyl 4-((4'-octyloxybiphenyl-4-yl)oxymethyl)benzoate | 109.21 | 105.30 | 105.50 | 3.71 |
| 280 | 2-Cyano-1,3-phenylene bis(4-(4-hexadecyloxyphenyliminomethyl)benzoate) | 198.71 | 195.30 | 195.00 | 3.71 |
| 281 | 1,3-Phenylene bis(4-(4'-heptylbiphenyl-4-carbonyloxy)-2-methylbenzoate) | 113.52 | 107.80 | 109.80 | 3.72 |
| 282 | 1-(2-(5-Chlorothienyl))-3-(4-octyloxyphenylamino)-2-propen-1-one | 85.93 | 82.50 | 82.20 | 3.73 |
| 283 | 1,3-Phenylene bis(4-(4'-pentylbiphenyl-4-carbonyloxy)-2-fluorobenzoate) | 104.36 | 108.90 | 108.10 | -3.74 |
| 284 | 2-Cyano-1,3-phenylene bis(4-(4'-dodecylbiphenyl-4-carbonyloxy)-2-fluorobenzoate) | 153.71 | 157.30 | 157.50 | -3.79 |
| 285 | 6-O-Hexadecyl-alpha-D-galactopyranose | 131.89 | 129.40 | 128.10 | 3.79 |
| 286 | 4-Nonyloxyphenyl 7-decanoyloxychromone-2-carboxylate | 76.09 | 83.30 | 79.90 | -3.81 |
| 287 | Bis(4-(3-propoxy-3-oxo-1-propenyl)phenyl terephthalate | 84.28 | 87.60 | 88.10 | -3.82 |
| 288 | 4-Methoxyphenyl 4-(4-(2-fluorophenylimino)methyl)benzoate | 79.95 | 83.70 | 83.80 | -3.85 |
| 289 | 2,2-Dimethylpropyl 4-(4-(4-methoxybenzylideneamino)benzoyloxy)benzoate | 96.47 | 93.20 | 92.60 | 3.87 |
| 290 | 1,3-Bis(4-(4-heptyloxybenzoyloxy)benzylideneamino)benzene | 127.02 | 130.80 | 130.90 | -3.88 |
| 291 | 7-(4'-Ethoxybenzoyloxy)isoflavone | 61.79 | 54.60 | 57.90 | 3.89 |
| 292 | 4-Tetradecyloxybenzoic acid | 132.69 | 138.00 | 136.60 | -3.91 |
| 293 | Di(4'-tetradecyloxyphenyl)-cyclohexane-1,4-dicarboxylate | 183.31 | 178.20 | 179.40 | 3.91 |
| 294 | 1-(4-Ethylcyclohexylethynyl)-4-(4-ethylphenylethynyl)benzene | 45.28 | 49.60 | 49.20 | -3.92 |
| 295 | 1,3-Phenylene bis(4-(4'-nonylbiphenyl-4-carbonyloxy)-2-ethylbenzoate) | 129.67 | 132.10 | 133.60 | -3.93 |
| 296 | Butyl 2-cyano-3-(4-(4-octanoyl-1-piperazinyl)phenylazophenyl)-2-propenoate | 44.43 | 38.70 | 40.50 | 3.93 |
| 297 | Phenyl 4-(4-methoxybenzylideneamino)benzoate | 78.52 | 82.70 | 82.50 | -3.98 |
| 298 | 4-Methyl-N-(4-(4-methoxybenzoyloxy)benzylidene)aniline | 71.62 | 75.10 | 75.60 | -3.98 |
| 299 | Bis(4-propoxycarbonylphenyl) terephthalate | 104.09 | 99.50 | 100.10 | 3.99 |
| 300 | 4-(Biphenyl-4-ylmethyleneamino)phenyl benzoate | 76.69 | 82.10 | 80.70 | -4.01 |
| 301 | 4-(1-Methylhepthyloxycarbonyl)phenyl 4'-(3-(perfluorobutanoyloxy)propyl)biphenyl-4-carboxylate | 84.59 | 89.90 | 88.60 | -4.01 |
| 302 | 4-Ethylphenyl 4-(4-methoxybenzylideneamino)benzoate | 77.09 | 79.20 | 81.10 | -4.01 |
| 303 | 4-(3-Pyridylmethyleneamino)phenyl 4-propoxybenzoate | 101.25 | 97.90 | 97.20 | 4.05 |
| 304 | 2-(4-Octyloxyphenyl)-5-(4-(2-methyl-2,3-pentadecadienyloxy)phenyl-1,3,4-thiadiazole | 121.86 | 117.90 | 117.80 | 4.06 |
| 305 | Cholesteryl propionate | 67.81 | 70.90 | 71.90 | -4.09 |
| 306 | 4-Methoxyphenyl 4-((phenylmethylene)amino)benzoate | 78.59 | 82.70 | 82.70 | -4.11 |
| 307 | N,N'-Didodecanoyl-3,4,5,6-tetramethylbenzene-1,2-diamine | 90.59 | 94.60 | 94.70 | -4.11 |
| 308 | 2-Bromo-3,6,7,10,11-pentakis(hexyloxy)triphenylene | 117.83 | 118.10 | 113.70 | 4.13 |
| 309 | N-(2-(6-Decyloxy)benzothiazolyl)-4-octyloxybenzamide | 95.86 | 100.80 | 100.00 | -4.14 |
| 310 | 4-Heptyl-4'-nonyloxyazobenzene | 124.16 | 128.80 | 128.30 | -4.14 |
| 311 | Bis(4-(4-hexadecyloxyphenyliminomethyl)phenyl) decanedioate | 246.05 | 241.60 | 241.90 | 4.15 |
| 312 | 1,3-Phenylene bis(4-(4-butoxyphenyliminomethyl)benzoate) | 102.54 | 106.60 | 106.70 | -4.16 |
| 313 | 4-(4'-Hexylcyclohexyl)isothiocyanatobenzene | 98.87 | 95.10 | 94.70 | 4.17 |
| 314 | 4-Cyano-4'-hexyloxybiphenyl | 84.78 | 80.20 | 80.60 | 4.18 |
| 315 | 2,3,6,7-Tetrakis(heptyloxy)-1,5-bis(2-(tetrahydropyran-2-yloxy)ethoxy)-9,10-anthraquinone | 137.32 | 141.80 | 141.50 | -4.18 |
| 316 | 4'-(2-(4-Propylphenyl)-1-ethynyl)-2'-methyl-4-butyltolane | 62.20 | 58.70 | 58.00 | 4.20 |
| 317 | 4-Pentylcyclohexyl 4-pentylcinnamate | 75.28 | 79.00 | 79.50 | -4.22 |
| 318 | 4-(4-(1-Methylheptyloxycarbonyl)phenyloxycarbonyl)phenyl 4-(4-(4-(4-decyloxybenzoyloxy)-benzoyloxy)butoxy)benzoate | 151.32 | 147.30 | 147.10 | 4.22 |
| 319 | 4-(Phenylmethyleneimino)phenyl 4-(phenylmethyleneimino)benzoate | 93.53 | 90.00 | 89.30 | 4.23 |
| 320 | 1,7-Bis(4-dodecyloxyazobenzene-4'-carbonyloxy)heptane | 196.42 | 198.60 | 200.70 | -4.28 |
| 321 | 4-Pentyl-4'-thiocyanatobiphenyl | 66.81 | 72.60 | 71.10 | -4.29 |
| 322 | 4'-(2-Diethylaminopropoxy)biphenyl-4-yl 4-nonyloxybenzoate | 36.40 | 32.50 | 32.10 | 4.30 |
| 323 | 1,9-Bis(4-(4-pentyloxybenzoyloxy)benzylideneamino)nonane | 150.00 | 153.40 | 154.30 | -4.30 |
| 324 | 4-Ethylphenyl 4-(4-hexyloxybenzylideneamino)benzoate | 94.17 | 98.00 | 98.50 | -4.33 |
| 325 | 4,5-Dichloro-1,3-phenylene bis(4-(4-butoxybenzylideneamino)benzoate) | 90.07 | 92.10 | 94.40 | -4.33 |
| 326 | 1-((5-Hexen-1-yl)phenyl)-4-(4-propylphenyl)-1,3-butadiyne | 78.66 | 82.60 | 83.00 | -4.34 |
| 327 | 1-(4-Dodecylbiphenyl-4'-yl)-3-(3-methylphenyl)propane-1,3-dione | 105.06 | 107.80 | 109.40 | -4.34 |
| 328 | 2-Cyano-1,3-phenylene bis(4-(4-octadecyloxyphenyliminomethyl)benzoate) | 216.35 | 211.50 | 212.00 | 4.35 |
| 329 | 1-(4-Hexylbiphenyl-4'-yl)-3-(4-cyanobiphenyl-4'-yl)propane-1,3-dione | 81.58 | 77.30 | 77.20 | 4.38 |
| 330 | 5-(4-Heptyloxyphenyl)-N-(4-(2-methylbutoxy)phenyl)methylene-1,3,4-thiadiazol-2-amine | 57.81 | 62.20 | 62.20 | -4.39 |
| 331 | 4-Ethyl-N-(4-heptyloxyphenylmethylene)benzenamine | 93.30 | 96.80 | 97.70 | -4.40 |
| 332 | 1,3-Phenylene bis(4-(4'-pentylbiphenyl-4-carbonyloxy)-3-fluorobenzoate) | 106.59 | 108.90 | 111.00 | -4.41 |
| 333 | 3-(4-(4-Heptylcyclohexyl)phenyl)-5-(4-octyloxyphenyl)isoxazole | 86.66 | 92.40 | 91.10 | -4.44 |
| 334 | 4-((Phenylmethylene)amino)phenyl 4-nitrobenzoate | 84.96 | 79.50 | 80.50 | 4.46 |
| 335 | Ethylphenyl 4-(4-octyloxybenzylideneamino)benzoate | 102.14 | 106.10 | 106.60 | -4.46 |
| 336 | 4-(2-Ethylheptyloxycarbonyl)phenyl 4-tridecanoyloxytolane-4'-carboxylate | 144.88 | 139.80 | 140.40 | 4.48 |
| 337 | 1-(4'-Hexylazobenzene-4-oxy)-6-(4'-methylazobenzene-4-oxy)hexane | 122.89 | 118.10 | 118.40 | 4.49 |
| 338 | N-(4-Ethoxybenzylidene)-4-butylaniline | 88.71 | 84.70 | 84.20 | 4.51 |
| 339 | Cholesteryl 4-(4-(4-hexylphenylethynyl)phenoxy)butanoate | 89.23 | 85.20 | 84.70 | 4.53 |
| 340 | 1-(6-Propoxy-2-naphthyl)-4-(4-fluorophenyl)diacetylene | 62.84 | 59.70 | 58.30 | 4.54 |
| 341 | 4'-(2-Diethylaminoethoxy)biphenyl-4-yl 4-decyloxybenzoate | 28.53 | 32.50 | 33.10 | -4.57 |
| 342 | 2-Methyl-4-(2-(4-pentylcyclohexyl)ethyl)phenylethynyl-1-(4-pentyloxyphenylethynyl)benzene | 74.28 | 69.70 | 69.70 | 4.58 |
| 343 | 6-O-Undecanoyl-alpha-D-galactopyranose | 73.02 | 79.80 | 77.60 | -4.58 |
| 344 | N,N',N''-Trioctanoyl-2,4,6-trimethyl-1,3,5-benzenetriamine | 82.79 | 76.10 | 78.20 | 4.59 |
| 345 | Bis(4-(4-dodecyloxybenzylideneamino)phenyl decanedioate | 205.01 | 209.20 | 209.60 | -4.59 |
| 346 | 1,2,2-Trimethylpropyl 4-(4-(4-acetoxybenzoyloxy)benzylideneamino)benzoate | 92.91 | 86.90 | 88.30 | 4.61 |
| 347 | 2-(4-Nonyloxyphenyl)-5-(4-decyloxy)phenylazo-1,3,4-thiadiazole | 112.52 | 106.70 | 107.90 | 4.62 |
| 348 | 4'-(2-(4-Ethylphenyl)-1-ethynyl)-2'-methyl-4-pentyltolane | 53.08 | 58.70 | 57.70 | -4.62 |
| 349 | 2-Methylbutyl 4-(4-(4-methoxybenzylideneamino)benzoyloxy)benzoate | 87.13 | 82.60 | 82.50 | 4.63 |
| 350 | 4-Hexyloxybenzylideneamino-4'-fluorobenzene | 93.66 | 97.40 | 98.30 | -4.64 |
| 351 | 4-Butoxy-4'-isothiocyanatotolane | 94.55 | 89.10 | 89.90 | 4.65 |
| 352 | 1,5-Bis(4-(4-heptyloxybenzoyloxy)benzylideneamino)pentane | 156.46 | 153.40 | 151.80 | 4.66 |
| 353 | 2-Decanoyl-1,4-phenylene bis(4-octyloxybenzoate) | 166.67 | 161.90 | 162.00 | 4.67 |
| 354 | 4-Pentylphenyl 4'-undecyloxythiobenzoate | 123.88 | 117.50 | 119.20 | 4.68 |
| 355 | 4-(((4-Ethoxyphenyl)methylene)amino)phenyl benzoate | 81.02 | 85.40 | 85.70 | -4.68 |
| 356 | 3-(4-Butoxyphenyl)-6-cyano-1,2,4,5-tetrazine | 55.22 | 59.90 |  | -4.68 |
| 357 | 1,5-Pentanediyl bis(4-(4-octyloxybiphenyl-4'-oxymethyl)benzoate) | 141.20 | 134.00 | 136.50 | 4.70 |
| 358 | 4-Propanoyl-4'-tetradecanoyloxyazobenzene | 141.80 | 137.20 | 137.10 | 4.70 |
| 359 | 4-((Phenylimino)methyl)phenyl 4-methoxybenzoate | 77.98 | 82.70 | 82.70 | -4.72 |
| 360 | 4-(3-Pyridinylmethyleneamino)phenyl 4-butoxybenzoate | 106.85 | 101.90 | 102.10 | 4.75 |
| 361 | N-(Biphenyl-4-yl)methylene-4-(biphenyl)amine | 75.36 | 71.20 | 70.60 | 4.76 |
| 362 | 6-Decyloxy-2-(4-heptyloxybenzylidenamino)benzothiazole | 110.64 | 115.10 | 115.40 | -4.76 |
| 363 | 4-Dodecyl-N-(4-pentadecyloxyphenylmethylene)benzenamine | 163.92 | 169.50 | 168.70 | -4.78 |
| 364 | 5-(4-Heptyloxyphenyl)-2-(4-octyloxy)benzylideneamino-1,3,4-thiadiazole | 91.79 | 86.90 | 87.00 | 4.79 |
| 365 | 2,3,6,7-Tetrakis(hexyloxy)-1,5-bis(2-(tetrahydropyran-2-yloxy)ethoxy)-9,10-anthraquinone | 128.90 | 125.70 | 124.10 | 4.80 |
| 366 | 1-Methylheptyl 4-(2-fluoro-4-(4-dodecyloxybenzoyloxy)phenylethynyl)benzoate | 125.90 | 121.00 | 121.10 | 4.80 |
| 367 | 3-Cyano-6-(4-heptylphenyl)-1,2,4,5-tetrazine | 75.70 | 70.90 |  | 4.80 |
| 368 | 4-Tridecyloxybenzoic acid | 140.80 | 134.00 | 136.00 | 4.80 |
| 369 | 1,8-Bis(4-(4-ethoxybenzoyloxybenzylideneamino)octane | 129.01 | 125.10 | 124.20 | 4.81 |
| 370 | Phenyl 4-(benzoyloxy)phenyl 1,4-benzenedicarboxylate | 100.51 | 96.00 | 95.70 | 4.81 |
| 371 | 4-Octyloxy-4'-cyanobiphenyl | 83.19 | 88.30 | 88.00 | -4.81 |
| 372 | Biphenyl-4-yl 4-(benzoyloxy)benzoate | 89.81 | 85.10 | 85.00 | 4.81 |
| 373 | 1,3-Phenylene bis(4-(4-heptyloxyphenyliminomethyl)benzoate) | 126.08 | 130.80 | 130.90 | -4.82 |
| 374 | 7-Pentyloxy-3-(4-decyloxyphenyl)-3H-1-benzopyran-4-one | 83.78 | 91.50 | 88.60 | -4.82 |
| 375 | 2-Methylpropyl 4-(4-(4-acetoxybenzoyloxy)benzylideneamino)benzoate | 79.57 | 86.90 | 84.40 | -4.83 |
| 376 | 4-Chloro-2'-hydroxy-4'-octyloxyazobenzene | 87.96 | 91.90 | 92.80 | -4.84 |
| 377 | 4-(2-Butoxy-1-methyl-2-oxoethoxycarbonyl)phenyl 4-(3-(6-hexyloxy-2-naphthalenyl)-1-oxo-2-propenyloxy)benzoate | 83.65 | 90.20 | 88.50 | -4.85 |
| 378 | 1,2-Dimethylpropyl 4-(4-(4-cyanobenzoyloxy)benzylideneamino)benzoate | 53.95 | 60.80 | 58.80 | -4.85 |
| 379 | Bis(4-ethoxyphenyl) 2-(8-(4-(4-biphenylazo)phenoxy)octyloxy) terephthalate | 120.26 | 115.20 | 115.40 | 4.86 |
| 380 | 4'-(2-(4-Methylphenyl)-1-ethynyl)-2'-methyl-4-hexyltolane | 62.58 | 58.70 | 57.70 | 4.88 |
| 381 | Bis(4-ethoxyphenyl) 2,2'-bithiophene-5,5'-dicarboxylate | 98.58 | 87.30 | 93.70 | 4.88 |
| 382 | 7-(4'-Heptyloxybenzoyloxy)isoflavone | 82.19 | 74.80 | 77.30 | 4.89 |
| 383 | N-(4-Octyloxybenzylidene)-4-pentylaniline | 118.32 | 113.00 | 113.40 | 4.92 |
| 384 | 3,3',4,4'-Biphenyltetracarboxy-N,N'-bis(hexyloxyphenyl)diimide | 104.48 | 109.30 | 109.40 | -4.92 |
| 385 | 1,10-Bis(4-(5-(4-(1,4,7-trioxaoctyl)phenyl)-1,3,4-thiadiazol-2-yl)phenyl)-1,4,7,10-tetraoxadecane | 86.77 | 94.30 | 91.70 | -4.93 |
| 386 | 4-Heptyloxy-4'-cyanobiphenyl | 88.24 | 84.20 | 83.30 | 4.94 |
| 387 | 5-(2-Chlorophenyl)-3-(4-(4-pentylcyclohexyl)phenyl)-1,2,4-oxadiazole | 71.64 | 63.20 | 66.70 | 4.94 |
| 388 | Phenyl 4-(4-hexyloxybenzylideneamino)benzoate | 106.26 | 101.60 | 101.30 | 4.96 |
| 389 | 4-Octyloxy-N-(4-bromobenzylidene)aniline | 108.67 | 103.60 | 103.70 | 4.97 |
| 390 | 1,7-Bis(4-(4-butoxybenzoyloxy)benzylideneamino)heptane | 132.11 | 137.20 | 137.10 | -4.99 |
| 391 | Phenyl 4-((4-nitrophenylmethylene)amino)benzoate | 85.80 | 79.50 | 80.80 | 5.00 |
| 392 | 1,3-Bis(4-(4-decyloxyphenylazo)phenoxy)-2-propanol | 151.30 | 157.70 | 156.30 | -5.00 |
| 393 | 4-Chlorobenzylidene 4'-heptylaniline | 86.79 | 92.00 | 91.80 | -5.01 |
| 394 | Cholesteryl cinnamate | 67.42 | 62.90 | 62.40 | 5.02 |
| 395 | 2-Decanoyl-1,4-phenylene bis(4-nonyloxybenzoate) | 175.73 | 170.00 | 170.70 | 5.03 |
| 396 | 1,3-Phenylene bis(4-(4-hexyloxyphenyliminomethyl)benzoate) | 127.13 | 122.80 | 122.10 | 5.03 |
| 397 | Cholesteryl 5-(4-(4-pentylphenyl)phenoxy)pentanoate | 96.23 | 90.20 | 91.20 | 5.03 |
| 398 | 3-Pyridyl 4-(4-butoxybenzylideneamino)benzoate | 97.37 | 101.90 | 102.40 | -5.03 |
| 399 | 1,6-Bis(4-(4-hexyloxybenzoyloxybenzylideneamino)hexane | 152.84 | 149.40 | 147.80 | 5.04 |
| 400 | Bis(4-ethoxyphenyl) 2-(4-(4-(4-butylphenylazo)phenoxy)butoxy) terephthalate | 113.24 | 109.20 | 108.20 | 5.04 |
| 401 | Pentyl 4-(4-(4-chlorobenzoyloxy)benzylideneamino)benzoate | 97.37 | 93.00 | 92.30 | 5.07 |
| 402 | p-Quinquephenyl | 65.47 | 57.60 | 60.40 | 5.07 |
| 403 | 6-Decyloxy-2-(4-propoxyphenylazo)benzothiazole | 94.42 | 102.60 | 99.50 | -5.08 |
| 404 | 4-Isopropylphenyl 4-(4-pentyloxybenzylideneamino)benzoate | 92.39 | 85.40 | 87.30 | 5.09 |
| 405 | Cholesteryl thiopropionate | 64.30 | 60.40 | 59.20 | 5.10 |
| 406 | 4-((4-cyanophenylimino)methyl)phenyl benzoate | 83.42 | 79.60 | 78.30 | 5.12 |
| 407 | 4-(4-Hexyloxyphenylazoxy)phenyl 2-chloro-3-methyl-pentanoate | 92.82 | 87.00 | 87.70 | 5.12 |
| 408 | 2-Methylbutyl 4-(4-(4-chlorobenzoyloxy)benzylideneamino)benzoate | 77.06 | 80.40 | 82.20 | -5.14 |
| 409 | 4-Pentyl-N-(4-nonyloxyphenylmethylene)benzenamine | 112.26 | 117.00 | 117.40 | -5.14 |
| 410 | 2-Chloro-3-methylpentyl 4-(3-(4-nonyloxyphenyl)-1-oxo-2-propenyloxy)benzoate | 97.45 | 92.40 | 92.30 | 5.15 |
| 411 | 1-(4-Butylphenyl)-4-(4-ethylphenyl)-1,3-butadiyne | 71.95 | 68.00 | 66.80 | 5.15 |
| 412 | 4-(4-Propoxyphenylazoxy)phenyl 2-chloro-3-methylpentanoate | 79.47 | 74.80 | 74.30 | 5.17 |
| 413 | 1-Methylheptyl 5-(4-decyloxybenzoyloxyphenylethynyl)-thiophene-2-carboxylate | 110.63 | 115.40 | 115.80 | -5.17 |
| 414 | 1-(4-Ethylcyclohexylethynyl)-4-(propylcyclohexylethynyl)benzene | 41.82 | 48.90 | 47.00 | -5.18 |
| 415 | 2,5-Bis(3,4,5-tris(tetradecyloxyphenyl))-1,3,4-oxadiazole | 191.81 | 194.80 | 197.00 | -5.19 |
| 416 | Di(4'-undecylphenyl)-cyclohexane-1,4-dicarboxylate | 145.21 | 151.70 | 150.40 | -5.19 |
| 417 | Bis(4-butoxyphenyl) 2,2'-bithiophene-5,5'-dicarboxylate | 108.22 | 103.50 | 103.00 | 5.22 |
| 418 | 4-Decyl-N-(4-octadecyloxyphenylmethylene)benzenamine | 179.83 | 173.60 | 174.60 | 5.23 |
| 419 | 1-Methylheptyl 5-(2,3-difluoro-4-hexadecyloxybenzoyloxyphenylethynyl)-thiophene-2-carboxylate | 146.23 | 141.70 | 141.00 | 5.23 |
| 420 | 4'-(4-Propylcyclohexyl)-4-isothiocyanatotolane | 67.37 | 73.50 | 72.60 | -5.23 |
| 421 | 4-Octyloxybenzylidene-4-heptylaniline | 125.64 | 121.00 | 120.40 | 5.24 |
| 422 | 4-((3-Pyridinyl)methyleneamino)phenyl 4-ethoxybenzoate | 89.06 | 93.80 | 94.30 | -5.24 |
| 423 | 2-Methylpentyl 4-(4-(4-methoxybenzylideneamino)benzoyloxy)benzoate | 93.05 | 86.60 | 87.80 | 5.25 |
| 424 | Bis(4-(5-decylthio-1,3,4-oxadiazole-2-yl)phenyl) terephthalate | 106.75 | 113.90 | 112.00 | -5.25 |
| 425 | 1-(6-Octyloxy-2-naphthyl)-2-(4-fluorophenyl)acetylene | 88.46 | 83.70 | 83.20 | 5.26 |
| 426 | 4-Isothiocyanatophenyl 4-(4-ethylcyclohexyl)benzoate | 79.01 | 84.20 | 84.30 | -5.29 |
| 427 | 4-Chloro-1,3-phenylene bis(4-(4-octyloxyphenyliminomethyl)benzoate) | 125.80 | 131.70 | 131.10 | -5.30 |
| 428 | 7-(4'-Octyloxybenzoyloxy)isoflavone | 75.19 | 78.90 | 80.50 | -5.31 |
| 429 | 6-Decyloxy-2-(4-hexyloxybenzylidenamino)benzothiazole | 115.92 | 111.10 | 110.60 | 5.32 |
| 430 | 6-O-Octyl-alpha-D-galactopyranose | 100.72 | 97.10 | 95.40 | 5.32 |
| 431 | 4-Undecyloxybenzoic acid | 120.07 | 125.90 | 125.40 | -5.33 |
| 432 | 4-Ethyl-4'-thiocyanatobiphenyl | 54.35 | 60.50 | 59.70 | -5.35 |
| 433 | Cholesteryl 5-(4-octyloxybiphenyl-4'-oxy)pentanoate | 108.17 | 102.30 | 102.80 | 5.37 |
| 434 | 4-Hexyloxybenzylidene-4'-toluidine | 94.72 | 88.70 | 89.30 | 5.42 |
| 435 | 4'-(2-Diethylaminoethoxy)biphenyl-4-yl 4-nonyloxybenzoate | 33.22 | 28.50 | 27.80 | 5.42 |
| 436 | 4,5-Dichloro-1,3-phenylene bis(4-(4-propoxybenzylideneamino)benzoate) | 88.73 | 84.00 | 83.30 | 5.43 |
| 437 | 2-(5-(4-(4-Pentylcyclohexyl)phenyl)-1,2,4-oxadiazol-3-yl)pyridine | 58.43 | 53.40 | 53.00 | 5.43 |
| 438 | 2-(4-Octyloxyphenyl)-5-(4-(2-methyl-2,3-undecadienyloxy)phenyl-1,3,4-thiadiazole | 97.45 | 101.80 | 102.90 | -5.45 |
| 439 | 4'-Undecyloxybiphenyl-4-yl 2-(1-methylpropyl)-3-oxo-1-thioxoisoindole-5-carboxylate | 80.65 | 84.60 | 86.10 | -5.45 |
| 440 | 4-Isopropylphenyl 4-(4-methoxybenzylideneamino)benzoate | 75.26 | 70.60 | 69.80 | 5.46 |
| 441 | Bis(4-(4-(4-butyoxyphenyliminomethyl)phenoxy)butyl) 2-ethylmalonate | 140.18 | 132.50 | 134.70 | 5.48 |
| 442 | Di(4'-hexadecyloxyphenyl)-cyclohexane-1,4-dicarboxylate | 189.51 | 194.40 | 195.00 | -5.49 |
| 443 | 4-Hexadecyloxybenzoic acid | 153.02 | 146.10 | 147.50 | 5.52 |
| 444 | N,N'-Dioctadecanoyl-2,5,6-trimethyl-4-octadecanoyloxy-1,3-benzenediamine | 214.37 | 218.60 | 219.90 | -5.53 |
| 445 | 1,1,2-Trimethylpropyl 4-(4-(4-methoxybenzoyloxy)benzylideneamino)benzoate | 94.96 | 101.30 | 100.50 | -5.54 |
| 446 | 4'-Hexadecyloxy-3'-nitrobiphenyl-4-carboxylic acid | 136.89 | 132.10 | 131.30 | 5.59 |
| 447 | Bis(4-Hexyloxyphenyl) 2,5-thiophenedicarboxylate | 131.19 | 121.80 | 125.60 | 5.59 |
| 448 | 1-(6-Butoxy-2-naphthyl)-4-(4-fluorophenyl)diacetylene | 67.19 | 63.70 | 61.60 | 5.59 |
| 449 | Di(4'-hexyloxyphenyl)-cyclohexane-1,4-dicarboxylate | 109.70 | 113.60 | 115.30 | -5.60 |
| 450 | Bis(4-(4-dodecyloxybenzylideneamino)phenyl) hexanedioate | 187.79 | 193.10 | 193.40 | -5.61 |
| 451 | 1,9-Bis(4-(4-octyloxybenzoyloxy)benzylideneamino)nonane | 170.99 | 177.60 | 176.60 | -5.61 |
| 452 | 4-(Phenyliminomethyl)phenyl biphenyl-4-carboxylate | 78.28 | 82.10 | 83.90 | -5.62 |
| 453 | 4-(2-Ethylheptyloxycarbonyl)phenyl 4-pentadecanoyloxytolane-4'-carboxylate | 154.43 | 147.90 | 148.80 | 5.63 |
| 454 | 4-(Benzoyloxy)phenyl 4-(phenylazo)benzoate | 91.27 | 96.70 | 96.90 | -5.63 |
| 455 | 4-Cyanophenyl 4-((phenylimino)methyl)benzoate | 85.15 | 79.60 | 79.50 | 5.65 |
| 456 | 4-Hexyloxy-3-fluoro-4'-isothiocyanatotolane | 92.75 | 98.20 | 98.40 | -5.65 |
| 457 | 4-(Phenyliminomethyl)phenyl 4-ethoxybenzoate | 79.84 | 85.40 | 85.50 | -5.66 |
| 458 | 4-(1-Methylheptyloxycarbonyl)phenyl 4-(4-hexadecyloxybenzoylthio)benzoate | 135.13 | 140.20 | 140.80 | -5.67 |
| 459 | 4'-(2-Diethylaminopropoxy)biphenyl-4-yl 4-octyloxybenzoate | 33.68 | 28.50 | 28.00 | 5.68 |
| 460 | 1-Methylheptyl 4-(4-(4-octyloxy-alpha-methylcinnamoyloxy)benzoyloxy)benzoate | 113.49 | 107.70 | 107.80 | 5.69 |
| 461 | 4-(4-Chlorophenylthiocarbonyl)phenyl 4-(hexyloxy)benzoate | 93.49 | 86.70 | 87.80 | 5.69 |
| 462 | 3-Heptylthio-1,2-propanediol | 100.10 | 104.30 | 105.80 | -5.70 |
| 463 | 4-(1-Methylheptyloxycarbonyl)phenyl 4-(2-perfluorohexylethoxy)biphenyl-4'-carboxylate | 102.81 | 97.10 | 97.10 | 5.71 |
| 464 | Diphenyl 4,4'-biphenyldicarboxylate | 90.74 | 85.10 | 85.00 | 5.74 |
| 465 | 2-Propanoyl-1,4-phenylene bis(4-heptyloxybenzoate) | 120.46 | 125.60 | 126.20 | -5.74 |
| 466 | 2-Butoxy-1-methyl-2-oxoethyl 4-(3-(6-tetradecyloxy-2-naphthalenyl)-1-oxo-2-propenyloxy)benzoate | 121.66 | 117.40 | 115.90 | 5.76 |
| 467 | 4-(5-(4-(4-Propylcyclohexyl)phenyl)-1,2,4-oxadiazol-3-yl)pyridine | 64.54 | 70.80 | 70.30 | -5.76 |
| 468 | 4-Cyanophenyl 4-(3-(4-(4'-dodecylbiphenyl-4-carbonyloxy)benzoyloxy)benzoyloxy)benzoate | 131.08 | 124.10 | 125.30 | 5.78 |
| 469 | 1-(4-Dodecylbiphenyl-4'-yl)-3-(2-hexyloxyphenyl)propane-1,3-dione | 133.69 | 129.20 | 127.90 | 5.79 |
| 470 | 4'-(2-(4-Methylphenyl)-1-ethynyl)-2'-methyl-4-pentyltolane | 60.81 | 54.70 | 55.00 | 5.81 |
| 471 | 1-(6-Butoxy-2-naphthyl)-2-(4-fluorophenyl)acetylene | 74.13 | 67.50 | 68.30 | 5.83 |
| 472 | 4-(Phenylazo)phenyl 4-(phenylmethyleneamino)benzoate | 87.97 | 93.70 | 93.80 | -5.83 |
| 473 | 1-(4'-Pentylazobenzene-4-oxy)-6-(4'-ethylazobenzene-4-oxy)hexane | 110.66 | 118.10 | 116.50 | -5.84 |
| 474 | 4-Methoxy-N-((5-methyl-2-thienyl)methylene)-1,1-biphenyl-4'-amine | 73.84 | 67.70 | 68.00 | 5.84 |
| 475 | 5-Fluoro-2-(4-n-tridecafluorohexylphenyl)isoindole-1,3-dione | 79.25 | 87.00 | 85.10 | -5.85 |
| 476 | 1,6-Bis(4'-ethylazobenzene-4-oxy)hexane | 100.34 | 105.90 | 106.20 | -5.86 |
| 477 | 4-(3-Pyridinylmethyleneamino)phenyl 4-octyloxybenzoate | 113.84 | 118.10 | 119.70 | -5.86 |
| 478 | 2,6,10-Tris(pentyloxy)-3,7,11-tris(butoxy)triphenylene | 91.24 | 96.30 | 97.10 | -5.86 |
| 479 | 4-Propanoyl-4'-tridecanoyloxyazobenzene | 138.87 | 133.20 | 133.00 | 5.87 |
| 480 | (1-Butoxycarbonyl)ethyl 5-(3-fluoro-4-undecyloxybenzoyloxyphenylethynyl)-thiophene-2-carboxylate | 122.87 | 116.30 | 117.00 | 5.87 |
| 481 | 1,5-Bis(4-(2-hydroxy-4-pentyloxybenzylideneamino)benzoyloxy)-3,3-dimethylpentane | 120.18 | 114.70 | 114.30 | 5.88 |
| 482 | 4'-(4-Butylcyclohexyl)-4-isothiocyanatotolane | 72.29 | 77.50 | 78.20 | -5.91 |
| 483 | 2-Methyl-1,3-phenylene bis(4-(3-chloro-4-decyloxyphenylethenyl)benzoate) | 112.23 | 105.30 | 106.30 | 5.93 |
| 484 | 4-Hexyloxybenzylideneamino-4'-chlorobenzene | 95.16 | 89.10 | 89.20 | 5.96 |
| 485 | Cholesteryl 5-(4-(4-butylphenylethynyl)phenoxy)pentanoate | 78.14 | 81.20 | 84.10 | -5.96 |
| 486 | Bicyclohexyl | 59.13 | 67.20 | 65.10 | -5.97 |
| 487 | 4-(4-Ethoxybenzoyloxy)-2-hexyloxy-4'-(4-butyoxysalicylaldimino)azobenzene | 108.58 | 101.70 | 102.60 | 5.98 |
| 488 | 4'-(4-Ethylcyclohexyl)-4-isothiocyanatotolane | 63.77 | 69.50 | 69.80 | -6.03 |
| 489 | 2-Octyloxy-6-(4-(4-octyloxyphenyl)buta-1,3-diynyl)naphthalene | 106.04 | 100.70 | 100.00 | 6.04 |
| 490 | 4-(2-Methylbutoxy)phenyl 4-(4-hexyloxy-2,3,5,6-tetrafluorophenylethynyl)benzoate | 94.75 | 91.00 | 88.70 | 6.05 |
| 491 | 2-Cyano-1,3-phenylene bis(4-(4-decyloxyphenyliminomethyl)benzoate) | 140.25 | 146.80 | 146.30 | -6.05 |
| 492 | 4-Butoxyphenyl 7-decanoyloxychromone-2-carboxylate | 58.43 | 63.10 | 64.50 | -6.07 |
| 493 | 4-Chloro-1,3-phenylene bis(4-(4-nonyloxybenzylideneamino)benzoate) | 133.86 | 139.80 | 140.00 | -6.14 |
| 494 | 1-Methylbutyl 4-(4-(4-chlorobenzoyloxy)benzylideneamino)benzoate | 79.57 | 74.40 | 73.40 | 6.17 |
| 495 | 4-Octyl-4'-thiocyanatobiphenyl | 90.18 | 84.70 | 84.00 | 6.18 |
| 496 | 2-Butanoyl-1,4-phenylene bis(4-octyloxybenzoate) | 130.71 | 137.70 | 136.90 | -6.19 |
| 497 | 1,6-Bis(4-(4-pentyloxybenzoyloxybenzylideneamino)hexane | 137.70 | 141.30 | 143.90 | -6.20 |
| 498 | 1-(4-(N-Ethoxycarbonylpiperazinyl)phenyl)-3-(4-decyloxyphenyl)-1-propargyl-3-imine | 85.32 | 80.40 | 79.10 | 6.22 |
| 499 | 2,3,6,7-Tetrakis(decyloxy)-1,5-bis(2-(tetrahydropyran-2-yl)ethoxy)-9,10-anthraquinone | 197.43 | 190.30 | 191.20 | 6.23 |
| 500 | 4-(4-Propylcyclohexyl)benzonitrile | 67.95 | 62.20 | 61.70 | 6.25 |
| 501 | 1-Methylheptyl 4'-(4''-dodecyloxy- 2'',3''-difluorobenzoyloxy)tolan-4-carboxylate | 115.92 | 122.00 | 122.20 | -6.28 |
| 502 | 4-Hexyl-N-(4-octadecyloxyphenylmethylene)benzenamine | 164.49 | 157.40 | 158.20 | 6.29 |
| 503 | 4-(4-Butoxy-2,3,5,6-tetrafluorophenylacetylenyl)phenyl 4'-chlorobenzoate | 83.89 | 78.60 | 77.60 | 6.29 |
| 504 | 4-Chloro-N-(4-(4-methylbenzoyloxy)benzylidene)aniline | 66.71 | 72.90 | 73.00 | -6.29 |
| 505 | 4-Pentadecyloxy-3-nitrobenzoic acid | 141.20 | 133.80 | 134.90 | 6.30 |
| 506 | N-(4-(4-Methoxybenzoyloxy)benzylidene)-2-hydroxyaniline | 85.40 | 91.60 | 91.70 | -6.30 |
| 507 | 4-Cyano-4'-(5-hydroxypentoxy)-stilbene | 92.82 | 85.70 | 86.50 | 6.32 |
| 508 | 4-(3-Pyridiniminomethyl)phenyl 4-butoxybenzoate | 95.08 | 101.90 | 101.40 | -6.32 |
| 509 | Bis(4-ethoxyphenyl) 2-(10-(4-(4-biphenylazo)phenoxy)decyloxy) terephthalate | 117.06 | 123.30 | 123.40 | -6.34 |
| 510 | 1,8-Bis(4-pentylazobenzene-4'-oxy)octane | 146.15 | 138.30 | 139.80 | 6.35 |
| 511 | 4-Nitrophenyl-4'-octyloxybenzoate | 108.55 | 99.10 | 102.20 | 6.35 |
| 512 | 4,5-Dichloro-1,3-phenylene bis(4-(4-octyloxybenzylideneamino)benzoate) | 130.87 | 124.40 | 124.50 | 6.37 |
| 513 | 4-Hexadecyloxy-4'-stilbazole N-oxide | 123.02 | 117.20 | 116.60 | 6.42 |
| 514 | 1-(4-Butylcyclohexylethynyl)-4-(4-ethylphenylethynyl)benzene | 50.68 | 57.60 | 57.10 | -6.42 |
| 515 | 4-(Phenyliminomethyl)phenyl 4-cyanobenzoate | 73.18 | 79.60 | 79.60 | -6.42 |
| 516 | 4-(2,2,3,3,4,4,4-Heptafluorobutyloxycarbonyl)phenyl 4-(octyloxy)benzoate | 97.66 | 103.40 | 104.10 | -6.44 |
| 517 | 1-Methylheptyl 4-(4-(4-undecyloxy-alpha-methylcinnamoyloxy)benzoyloxy)benzoate | 112.85 | 119.80 | 119.30 | -6.45 |
| 518 | 5-(4-Pentyloxyphenyl)-N-(4-(2-methylbutoxy)phenyl)methylene-1,3,4-thiadiazol-2-amine | 61.77 | 54.10 | 55.30 | 6.47 |
| 519 | 4-(4-Undecyloxyphenylazoxy)phenyl 2-bromo-3-methylpentanoate | 122.09 | 127.10 | 128.60 | -6.51 |
| 520 | N-(4-Pentyloxybenzylidene)-4-butylaniline | 102.71 | 96.80 | 96.20 | 6.51 |
| 521 | 4-(Phenoxycarbonyl)phenyl 4-(phenylmethyleneimino)benzoate | 98.62 | 93.00 | 92.10 | 6.52 |
| 522 | 2-Methylbutyl 4-(4-(4-methoxybenzoyloxy)benzylideneamino)benzoate | 73.98 | 82.60 | 80.50 | -6.52 |
| 523 | 4-(4-Propoxyphenylmethyleneamino)phenyl 3-pyridinecarboxylate | 92.87 | 97.90 | 99.40 | -6.53 |
| 524 | 4,4'-Bis(4-(4-butoxybenzoyloxy)benzylideneamino)diphenylamine | 95.06 | 88.20 | 88.50 | 6.56 |
| 525 | 2-Methyl-1,3-phenylene bis(4-(3-chloro-4-nonyloxyphenylethenyl)benzoate) | 100.97 | 97.20 | 94.40 | 6.57 |
| 526 | 4-(4-Benzoyloxyphenyliminomethyl)phenyl benzoate | 86.40 | 93.00 | 93.00 | -6.60 |
| 527 | 4-Dodecyloxy-4'-stilbazole N-oxide | 107.01 | 101.00 | 100.40 | 6.61 |
| 528 | 2-(4-Octyloxy)phenylazo-5-(2-heptyl)thien-5-yl-1,3,4-thiadiazole | 85.89 | 92.90 | 92.50 | -6.61 |
| 529 | 4-Chlorophenyl 4-undecyloxythiobenzoate | 109.72 | 101.70 | 103.10 | 6.62 |
| 530 | 1-(4'-Hexylazobenzene-4-oxy)-6-(4'-pentylazobenzene-4-oxy)hexane | 140.93 | 134.20 | 134.30 | 6.63 |
| 531 | 4-Chlorophenyl 4-heptyloxythiobenzoate | 92.94 | 85.50 | 86.30 | 6.64 |
| 532 | N,N'-Dioctanoyl-3,4,5,6-tetramethylbenzene-1,2-diamine | 67.05 | 62.30 | 60.40 | 6.65 |
| 533 | 1-(6-Propoxy-2-naphthyl)-2-(3,4-difluorophenyl)acetylene | 71.66 | 64.50 | 65.00 | 6.66 |
| 534 | 4-(4-Ethoxyphenylazo)phenyl 4-octyloxybenzoate | 105.42 | 110.90 | 112.10 | -6.68 |
| 535 | 4-(1-Methylheptyloxycarbonyl)phenyl 4-(4-octyloxybenzoylthio)benzoate | 115.59 | 107.90 | 108.90 | 6.69 |
| 536 | 1-(4-Pentylphenyl)-4-(4-tolyl)-1,3-butadiyne | 77.40 | 68.00 | 70.70 | 6.70 |
| 537 | Propyl 4-(4-(4-Chlorobenzoyloxy)benzylideneamino)benzoate | 90.90 | 84.90 | 84.20 | 6.70 |
| 538 | N,N'-Dinonanoyl-3,4,5,6-tetramethylbenzene-1,2-diamine | 63.99 | 70.40 | 70.70 | -6.71 |
| 539 | 4-(4-Octyloxybenzylideneamino)phenyl 4-isopropylbenzoate | 91.84 | 97.50 | 98.60 | -6.76 |
| 540 | 4-Ethylphenyl 4-(4-decyloxybenzylideneamino)benzoate | 107.63 | 114.10 | 114.40 | -6.77 |
| 541 | 4-Chlorobenzylidene 4'-nonylaniline | 106.58 | 100.10 | 99.80 | 6.78 |
| 542 | 4-(2-Ethylheptyloxycarbonyl)phenyl 4-heptadecanoyloxytolane-4'-carboxylate | 163.50 | 156.00 | 156.70 | 6.80 |
| 543 | 4-Butyl-4'-octyloxyazobenzene | 105.90 | 112.60 | 112.70 | -6.80 |
| 544 | 6-Pentyloxy-2-(4-methoxystyryl)quinoline | 80.91 | 74.90 | 74.10 | 6.81 |
| 545 | 2-(4-Pentyloxyphenylazo)-5-(5'-heptyl-2'-thienyl)-1,3,4-thiadiazole | 76.68 | 80.80 | 83.50 | -6.82 |
| 546 | 4-Nitrophenyl 4-((phenylimino)methyl)benzoate | 85.95 | 79.50 | 79.10 | 6.85 |
| 547 | N-(4-Methoxyphenyl)-alpha-(4-octyloxyphenyl)nitrone | 130.96 | 124.50 | 124.10 | 6.86 |
| 548 | 1-(4-(4-Formylphenylazo)phenyl)-4-(1-oxooctyl)piperazine | 49.36 | 44.10 | 42.50 | 6.86 |
| 549 | 1-Methylheptyl 4'-(4''-undecyloxy- 2'',3''-difluorobenzoyloxy)tolan-4-carboxylate | 126.37 | 118.00 | 119.50 | 6.87 |
| 550 | 6-Pentyloxy-2-(4-hexyloxystyryl)quinoline | 89.33 | 93.70 | 96.20 | -6.87 |
| 551 | N-(4-Ethoxybenzylidene)-4-hexylaniline | 85.31 | 92.80 | 92.20 | -6.89 |
| 552 | 4-(4-Butoxyphenylmethyleneamino)phenyl 3-pyridinecarboxylate | 108.02 | 101.90 | 101.10 | 6.92 |
| 553 | 3-Methylbutyl 4-(4-(4-acetoxybenzoyloxy)benzylideneamino)benzoate | 83.58 | 90.90 | 90.50 | -6.92 |
| 554 | 4-(3-Ethylmercapto-2-methylpropionyloxy)phenyl 4'-tridecyloxybiphenyl-4-carboxylate | 112.45 | 117.60 | 119.40 | -6.95 |
| 555 | (1-Butoxycarbonyl)ethyl 5-(3-fluoro-4-nonyloxybenzoyloxyphenylethynyl)-thiophene-2-carboxylate | 112.30 | 108.20 | 105.30 | 7.00 |
| 556 | 4-(4'-Heptylcyclohexyl)isothiocyanatobenzene | 106.50 | 99.10 | 99.50 | 7.00 |
| 557 | Cholesteryl thiododecanoate | 104.00 | 96.70 | 97.00 | 7.00 |
| 558 | 1,3-Phenylene bis(4-(4-tetradecyloxybenzoylthio)benzoate) | 165.88 | 172.20 | 172.90 | -7.02 |
| 559 | 3-Cyanophenyl 2,5-bis(4-octyloxybenzoyloxy)benzoate | 124.97 | 131.40 | 132.00 | -7.03 |
| 560 | 4-(4-(Phenylazo)phenyliminomethyl)phenyl benzoate | 87.27 | 93.70 | 94.30 | -7.03 |
| 561 | 3-Methylbutyl 4-(4-(4-methylbenzoyloxy)benzylideneamino)benzoate | 73.36 | 80.00 | 80.40 | -7.04 |
| 562 | 1-(4-Dodecylbiphenyl-4'-yl)-3-(2-butoxyphenyl)propane-1,3-dione | 128.55 | 121.10 | 121.50 | 7.05 |
| 563 | Phenyl 4-(4-butoxybenzylideneamino)benzoate | 86.74 | 93.50 | 93.80 | -7.06 |
| 564 | 1-(4-Fluorophenyl)-4-(4-pentylphenyl)-1,3-butadiyne | 69.72 | 76.60 | 76.80 | -7.08 |
| 565 | 4-Hexyl-4'-thiocyanatobiphenyl | 68.81 | 76.60 | 75.90 | -7.09 |
| 566 | Cholesteryl thiooctadecanoate | 127.60 | 121.00 | 120.50 | 7.10 |
| 567 | Pentaerythritol mono-4-pentylcyclohexylmethyl ether | 124.02 | 117.10 | 116.90 | 7.12 |
| 568 | 1-(2-(5-Chlorothienyl))-3-(4-ethoxyphenylamino)-2-propen-1-one | 52.47 | 58.20 | 59.60 | -7.13 |
| 569 | 4'-(4-Pentenyloxy)biphenyl 5-cyano-2-thiophenecarboxylate | 94.85 | 87.40 | 87.70 | 7.15 |
| 570 | 5-Decyl-2-(4-isothiocyanatophenyl)-1,3-dioxane | 121.35 | 115.50 | 114.20 | 7.15 |
| 571 | 4-(4-Ethoxybenzoyloxy)-2-decyloxy-4'-(4-butyoxysalicylaldimino)azobenzene | 111.24 | 117.90 | 118.40 | -7.16 |
| 572 | 1-(6-Propoxy-2-naphthyl)-2-(4-fluorophenyl)acetylene | 71.57 | 63.50 | 64.40 | 7.17 |
| 573 | 1,10-Dipropyl(permethyl)decasilane | 114.28 | 107.10 |  | 7.18 |
| 574 | 1-Methylbutyl 4-(4-(4-acetoxybenzoyloxy)benzylideneamino)benzoate | 77.12 | 84.90 | 84.30 | -7.18 |
| 575 | 4-(Phenylmethyleneamino)phenyl biphenyl-4-carboxylate | 90.30 | 82.10 | 83.10 | 7.20 |
| 576 | 1,6-Bis(4-butoxyazobenzene-4'-carbonyloxy)hexane | 135.61 | 129.90 | 128.40 | 7.21 |
| 577 | 4-Pentylphenyl 4'-tridecyloxythiobenzoate | 132.53 | 125.50 | 125.30 | 7.23 |
| 578 | 2,3,6,7,11,12-Hexakis(decyloxy)-10,13-dimethylbenzo[b]triphenylene | 210.89 | 204.30 | 203.60 | 7.29 |
| 579 | 1-Methylheptyl 4'-(4''-octadecyloxy- 2'',3''-difluorobenzoyloxy)tolan-4-carboxylate | 152.89 | 146.20 | 145.60 | 7.29 |
| 580 | 4-(4-Dodecyloxyphenyliminomethyl)benzoic acid | 139.71 | 132.20 | 132.40 | 7.31 |
| 581 | 4-Nitrobenzylidene 4'-dodecylaniline | 118.12 | 111.10 | 110.80 | 7.32 |
| 582 | 4-Hexadecyloxy-3'-stilbazole N-oxide | 123.46 | 117.20 | 116.10 | 7.36 |
| 583 | 4,6-Dichloro-1,3-phenylene bis(4-(4-octyloxyphenyliminomethyl)benzoate) | 116.62 | 124.40 | 124.00 | -7.38 |
| 584 | 1-Methylbutyl 4-(4-(4-methoxybenzylideneamino)benzoyloxy)benzoate | 85.31 | 76.50 | 77.90 | 7.41 |
| 585 | 1-((4-Penten-1-yl)phenyl)-4-(4-tolyl)-1,3-butadiyne | 74.91 | 70.50 | 67.50 | 7.41 |
| 586 | 1-(2-(5-Heptylthienyl))-3-(4-bromophenylamino)-2-propen-1-one | 72.94 | 68.50 | 65.50 | 7.44 |
| 587 | 1,3-Phenylene bis(4-(3-chloro-4-octyloxyphenylethenyl)benzoate) | 91.23 | 96.70 | 98.70 | -7.47 |
| 588 | 4-(3-(4-(Hexyloxy)phenyl)-1-oxo-2-propenyloxy)phenyl 2-chloro-3-methylpentanoate | 73.93 | 83.20 | 81.40 | -7.47 |
| 589 | 4-(3,3,4,4,5,5,6,6,7,7,8,8,8-Tridecafluorooctyloxycarbonyl)phenyl 4-((perfluorohexyl)hexyloxy)benzoate | 121.01 | 127.20 | 128.50 | -7.49 |
| 590 | 5-Octyl-2-(4-isothiocyanatophenyl)-1,3-dioxane | 98.71 | 107.50 | 106.20 | -7.49 |
| 591 | 1,8-Bis(4-butylbiphenyl-4'-yl)octane | 97.80 | 104.60 | 105.30 | -7.50 |
| 592 | Cholesteryl 8-phenyloctanoate | 104.71 | 97.10 | 97.20 | 7.51 |
| 593 | 3-(4-(4-Cyanophenoxycarbonyl)phenoxycarbonyl)phenyl 2-fluoro-4-(4-octadecyloxybenzoyloxy)benzoate | 163.51 | 156.20 | 156.00 | 7.51 |
| 594 | 2-Cyano-3,6,7,10,11-pentakis(hexyloxy)triphenylene | 117.83 | 110.70 | 110.30 | 7.53 |
| 595 | 4-((4-Ethoxyphenylmethyleneamino)phenyl 3-pyridinecarboxylate | 85.57 | 93.80 | 93.10 | -7.53 |
| 596 | 4-Thiocyanophenyl 4-pentyloxybiphenyl-4'-carboxylate | 85.94 | 79.00 | 78.40 | 7.54 |
| 597 | 5-Hexyl-2-(4-isothiocyanatophenyl)-1,3-dioxane | 93.15 | 99.40 | 100.70 | -7.55 |
| 598 | 2-(5-(4-Octyloxyphenyl)-1,3,4-thiadiazol-2-yliminomethyl)-5-(octyloxy)phenol | 88.86 | 82.00 | 81.30 | 7.56 |
| 599 | 5-Methyl-5'-(2-(4-octyloxyphenyl)-2-hydroxyethyl)-2,2'-bipyridine | 46.82 | 54.20 | 54.40 | -7.58 |
| 600 | Bis(4-heptyloxyphenyl) 2,2'-bithiophene-5,5'-dicarboxylate | 134.60 | 127.70 | 127.00 | 7.60 |
| 601 | 4'-(2-Diethylaminoethoxy)biphenyl-4-yl 4-undecyloxybenzoate | 43.55 | 36.50 | 35.90 | 7.65 |
| 602 | 4'-Tridecyloxy-3'-nitrobiphenyl-4-carboxylic acid | 112.22 | 120.00 | 119.90 | -7.68 |
| 603 | 7-(4'-Dodecyloxybenzoyloxy)isoflavone | 86.79 | 95.00 | 94.50 | -7.71 |
| 604 | 4-(4-(1-Methylheptyloxycarbonyl)phenyloxycarbonyl)phenyl 4-(6-(4-(4-dodecyloxybenzoyloxy)benzoyloxy)pentyloxy)benzoate | 167.02 | 159.50 | 159.30 | 7.72 |
| 605 | 4-Cyanophenyl 4-(3-(4-(4'-octylbiphenyl-4-carbonyloxy)benzoyloxy)benzoyloxy)benzoate | 114.23 | 107.90 | 106.50 | 7.73 |
| 606 | 1-(6-Hexyloxy-2-naphthyl)-4-(4-fluorophenyl)diacetylene | 66.06 | 71.80 | 73.80 | -7.74 |
| 607 | 3-Methyl-4-(4-pentylcyclohexylethynyl)-1-(3,4,5-trifluorophenylethynyl)benzene | 67.60 | 60.70 | 59.80 | 7.80 |
| 608 | 4-(2-Methylbutoxy)phenyl 4-(4-methoxy-2,3,5,6-tetrafluorophenylethynyl)benzoate | 66.29 | 72.20 | 74.10 | -7.81 |
| 609 | 4,4'-Dihexyloxyazoxybenzene | 119.92 | 110.90 | 112.10 | 7.82 |
| 610 | 4,4'''-Ditridecyloxy-p-quaterphenyl | 138.96 | 147.40 | 146.80 | -7.84 |
| 611 | 4-(Phenylazo)phenyl 4-(phenylazo)benzoate | 105.16 | 97.40 | 97.30 | 7.86 |
| 612 | 2-Cyano-1,3-phenylene bis(4-(4'-undecylbiphenyl-4-carbonyloxy)-2-fluorobenzoate) | 157.31 | 149.20 | 149.40 | 7.91 |
| 613 | 4-Cyano-4'-octylbiphenyl | 78.89 | 87.10 | 86.80 | -7.91 |
| 614 | 4-Pentanoyl-4'-tetradecanoyloxyazobenzene | 152.63 | 145.30 | 144.70 | 7.93 |
| 615 | 2,6-Bis(4-((4Z)-4-decenyloxy)phenyl)anthracene | 70.77 | 80.70 | 78.70 | -7.93 |
| 616 | 1,3-Phenylene bis(4-(4-pentyloxyphenyliminomethyl)benzoate) | 107.17 | 114.70 | 115.10 | -7.93 |
| 617 | 5-(4-Decyloxyphenyl)-2-(2-hydroxy-4-nonyloxy)benzylideneamino-1,3,4-thiadiazole | 85.96 | 94.10 | 93.90 | -7.94 |
| 618 | 2-Methyl-3-chloro-N-(4-(4-methoxybenzoyloxy)benzylidene)aniline | 76.16 | 67.90 | 68.20 | 7.96 |
| 619 | 2-Heptanoyl-1,4-phenylene bis(4-hexyloxybenzoate) | 142.21 | 133.70 | 134.20 | 8.01 |
| 620 | 1-Methylpentyl 4-(4-(4-methoxybenzylideneamino)benzoyloxy)benzoate | 89.13 | 80.60 | 81.10 | 8.03 |
| 621 | 1-(4-Propylcyclohexylethynyl)-4-(4-pentylcyclohexylethynyl)benzene | 54.07 | 61.00 | 62.10 | -8.03 |
| 622 | 5-(4-Heptyloxyphenyl)-2-(4-octyloxy)phenylamido-1,3,4-thiadiazole | 61.06 | 68.60 | 69.10 | -8.04 |
| 623 | 2-Dodecanoyl-1,4-phenylene bis(4-octyloxybenzoate) | 162.56 | 170.00 | 170.60 | -8.04 |
| 624 | 1-Ethylbutyl 4-(4-(4-methoxybenzylideneamino)benzoyloxy)benzoate | 89.75 | 80.60 | 81.70 | 8.05 |
| 625 | 4-Heptylcyclohexyl 4-ethoxycinnamate | 85.97 | 76.10 | 77.90 | 8.07 |
| 626 | t-Butyl 4-(4-(4-Nitrobenzoyloxy)benzylideneamino)benzoate | 93.93 | 102.60 | 102.00 | -8.07 |
| 627 | 4,5-Dichloro-1,3-phenylene bis(4-(4-pentyloxybenzylideneamino)benzoate) | 94.33 | 100.20 | 102.40 | -8.07 |
| 628 | 4-(4-Pentylcyclohexyl)-4'-nitrotolane | 68.49 | 60.80 | 60.40 | 8.09 |
| 629 | 4-Bromo-4'-(5-hexenyloxy)biphenyl | 85.40 | 90.10 | 93.50 | -8.10 |
| 630 | N-(2-Hydroxy-4-propoxybenzylidene)-4'-dodecylphenylaniline | 115.03 | 106.40 | 106.90 | 8.13 |
| 631 | 6-Pentyloxy-2-(4-decyloxystyryl)quinoline | 118.84 | 109.80 | 110.70 | 8.14 |
| 632 | 1-Methylheptyl 5-(4-tridecyloxybenzoyloxyphenylethynyl)-thiophene-2-carboxylate | 120.34 | 127.50 | 128.50 | -8.16 |
| 633 | 4-(2-Methylbutoxycarbonyl)phenyl 4-(4-ethoxy-2,3,4,5-tetrafluorophenylethynyl)benzoate | 84.97 | 77.60 | 76.80 | 8.17 |
| 634 | 5-(4-Nonyloxyphenyl)-2-(4-octyloxy)benzylideneamino-1,3,4-thiadiazole | 104.47 | 95.00 | 96.30 | 8.17 |
| 635 | 4-Isothiocyanatophenyl 4-(4-octylcyclohexyl)benzoate | 116.48 | 108.40 | 108.30 | 8.18 |
| 636 | 4,6-Dichloro-1,3-phenylene bis(4-(4-dodecyloxy-3-fluorophenyliminomethyl)benzoate) | 166.58 | 158.80 | 158.40 | 8.18 |
| 637 | 4-(4-Ethoxybenzoyloxy)-2-butoxy-4'-(4-butyoxysalicylaldimino)azobenzene | 102.60 | 93.70 | 94.40 | 8.20 |
| 638 | 1-Methylheptyl 4'-(4''-decyloxy- 2'',3''-difluorobenzoyloxy)tolan-4-carboxylate | 105.00 | 113.90 | 113.20 | -8.20 |
| 639 | 2-Chloro-4-methylpentyl 4-(3-(4-octyloxyphenyl)-1-oxo-2-propenyloxy)benzoate | 80.49 | 88.30 | 88.70 | -8.21 |
| 640 | 4-Propanoyl-4'-dodecanoyloxyazobenzene | 120.78 | 129.20 | 129.00 | -8.22 |
| 641 | 4-Octyloxyphenyl 7-decanoyloxychromone-2-carboxylate | 72.68 | 79.30 | 80.90 | -8.22 |
| 642 | N,N'-Didodecanoyl-2,5,6-trimethyl-4-dodecanoyloxy-1,3-benzenediamine | 135.68 | 145.90 | 143.90 | -8.22 |
| 643 | Benzo[ghi]fluoranthene | 27.83 | 24.30 | 19.60 | 8.23 |
| 644 | 4-Thiocyanophenyl 4-(4-octyloxybenzoyloxy)benzoate | 109.65 | 102.00 | 101.40 | 8.25 |
| 645 | 3,3',4,4'-Biphenyltetracarboxy-N,N'-bis(hexylphenyl)diimide | 113.87 | 106.90 | 105.60 | 8.27 |
| 646 | 4-((4-Nitrophenylmethylene)amino)phenyl benzoate | 70.42 | 79.50 | 78.70 | -8.28 |
| 647 | 4-Benzylideneamino-4'-methoxybiphenyl | 80.20 | 71.90 | 71.90 | 8.30 |
| 648 | 4-(3-(4-(Nonyloxy)phenyl)-1-oxo-2-propenyloxy)phenyl 2-chloro-4-methylpentanoate | 86.30 | 95.30 | 94.60 | -8.30 |
| 649 | Ethanol | 93.61 | 86.30 | 85.30 | 8.31 |
| 650 | Bis(4-(4-dodecyloxyphenyliminomethyl)phenyl) decanedioate | 200.08 | 209.20 | 208.40 | -8.32 |
| 651 | 4-Pentylphenyl 4'-decyloxythiobenzoate | 122.23 | 113.40 | 113.90 | 8.33 |
| 652 | 1,3-Phenylene bis(4-(4'-undecylbiphenyl-4-carbonyloxy)benzoate) | 148.35 | 155.40 | 156.70 | -8.35 |
| 653 | 5-(4'-Hexyloxybiphenyl-4-yl)-2-(4-methylphenyl)pyridine | 43.41 | 52.50 | 51.80 | -8.39 |
| 654 | 4-Undecanoyloxy-2,3-dimethyl-4'-ethoxyazobenzene | 116.61 | 108.30 | 108.20 | 8.41 |
| 655 | 4-Propyl-4'-thiocyanatobiphenyl | 70.91 | 64.50 | 62.50 | 8.41 |
| 656 | 1-Methylheptyl 4'-(4''-octyloxy-2'',3''-difluorobenzoyloxy)tolan-4-carboxylate | 96.99 | 105.80 | 105.40 | -8.41 |
| 657 | 4-Cyanobiphenyl-4'-yl 3-(4'-decyloxybiphenyl-4-yloxy)-2-propenoate | 86.06 | 93.40 | 94.50 | -8.44 |
| 658 | 4-Isopropylphenyl 4-(4-decyloxybenzylideneamino)benzoate | 114.78 | 105.60 | 106.30 | 8.48 |
| 659 | 4-(4-Hexyloxyphenylmethyleneamino)phenyl 3-pyridinecarboxylate | 118.99 | 110.00 | 110.50 | 8.49 |
| 660 | 4-Pentanoyl-4'-ethanoyloxyazobenzene | 89.90 | 96.90 | 98.40 | -8.50 |
| 661 | 4'-(3-Diethylaminopropoxy)biphenyl-4-yl 4-dodecyloxybenzoate | 36.99 | 44.60 | 45.50 | -8.51 |
| 662 | 2-(4-Nonyloxyphenylazo)-5-(5'-heptyl-2'-thienyl)-1,3,4-thiadiazole | 91.07 | 97.00 | 99.60 | -8.53 |
| 663 | Di(4'-hexylphenyl)-cyclohexane-1,4-dicarboxylate | 103.07 | 111.30 | 111.60 | -8.53 |
| 664 | 1,5-Bis(4-(2-hydroxy-4-octyloxybenzylideneamino)benzoyloxy)-2,2-dimethylpentane | 132.27 | 138.90 | 140.80 | -8.53 |
| 665 | 1,3-Phenylene bis(4-(4'-dodecylbiphenyl-4-carbonyloxy)-2-methylbenzoate) | 155.24 | 148.20 | 146.70 | 8.54 |
| 666 | 1-(4-Butoxyphenyl)-4-(4-cyanophenyl)-1,3-butadiene | 65.04 | 60.20 | 56.50 | 8.54 |
| 667 | Biphenyl-4-yl 4-(phenylazo)benzoate | 77.33 | 85.80 | 85.90 | -8.57 |
| 668 | 4-(1-Methyloctyloxycarbonyl)phenyl 4-octyloxybiphenyl-4'-carboxylate | 103.57 | 111.70 | 112.20 | -8.63 |
| 669 | 4-Hexyl-4'-fluorodiphenyldiacetylene | 89.54 | 80.70 | 80.90 | 8.64 |
| 670 | 4'-(3-Diethylaminopropoxy)biphenyl-4-yl 4-decyloxybenzoate | 28.91 | 36.50 | 37.60 | -8.69 |
| 671 | 4-(Phenylazo)phenyl 4-(benzoyloxy)benzoate | 89.29 | 96.70 | 98.00 | -8.71 |
| 672 | 4-Methylpentyl 4-(4-(4-methylbenzoyloxy)benzylideneamino)benzoate | 75.79 | 84.10 | 84.50 | -8.71 |
| 673 | Bis(4-pentyloxycarbonyl)phenyl) terephthalate | 107.09 | 115.70 | 115.80 | -8.71 |
| 674 | N-(4-(5-oxo-4-dodecanoylamino-1,3,6-cycloheptatrien-1-yl)phenyl)dodecamide | 118.03 | 109.60 | 109.30 | 8.73 |
| 675 | Cholesteryl 5-(4-heptyloxybiphenyl-4'-oxy)pentanoate | 107.13 | 98.30 | 98.40 | 8.73 |
| 676 | 7-(4-(4-(4-Nonylphenoxycarbonyl)phenoxycarbonyl)phenolheptyl) 4'-nonylbiphenylcarboxylate | 154.34 | 162.20 | 163.10 | -8.76 |
| 677 | N-(4-(5-oxo-4-tridecanoylamino-1,3,6-cycloheptatrien-1-yl)phenyl)tridecamide | 123.07 | 117.70 | 114.30 | 8.77 |
| 678 | N-(2-Hydroxy-4-methoxybenzylidene)-4-butylaniline | 80.27 | 73.00 | 71.50 | 8.77 |
| 679 | 7-Heptyloxy-3-(4-decyloxyphenyl)-3H-1-benzopyran-4-one | 107.28 | 99.50 | 98.50 | 8.78 |
| 680 | 4-((4-Methoxyphenylmethylene)amino)phenyl 3-pyridinecarboxylate | 102.20 | 91.20 | 93.40 | 8.80 |
| 681 | 4-(4-Ethoxyphenylazo)phenyl 4-(oddecyloxy)benzoate | 135.91 | 127.10 | 127.10 | 8.81 |
| 682 | 4-Tridecanoyloxy-3-methyl-4'-ethoxyazobenzene | 132.92 | 124.00 | 124.10 | 8.82 |
| 683 | 3-Methyl-4-(4-pentylcyclohexylethynyl)-1-(4-pentyloxyphenylethynyl)benzene | 77.24 | 67.30 | 68.40 | 8.84 |
| 684 | 4-Pentanoyloxy-2,3-dimethyl-4'-ethoxyazobenzene | 90.37 | 84.00 | 81.50 | 8.87 |
| 685 | 4-Cyano-1,3-phenylene bis(4-(4-octyloxyphenyliminomethyl)benzoate) | 121.33 | 130.70 | 130.20 | -8.87 |
| 686 | 4-Pentyloxy-3-fluoro-4'-isothiocyanatotolane | 100.98 | 94.10 | 92.10 | 8.88 |
| 687 | 4-Thiocyanophenyl 4-hexyloxybiphenyl-4'-carboxylate | 91.38 | 83.00 | 82.50 | 8.88 |
| 688 | 3,4-Dihexadecyloxycinnamic aldehyde-2',4'-dinitrophenylhydrazone | 146.88 | 138.60 | 138.00 | 8.88 |
| 689 | 2-(4-Cyanophenyl)-7-butylfluorene | 44.72 | 50.80 | 53.60 | -8.88 |
| 690 | N,N'-Diundecanoyl-3,4,5,6-tetramethylbenzene-1,2-diamine | 78.52 | 86.50 | 87.40 | -8.88 |
| 691 | 4-(2,2,3,3,4,4,4-Heptafluorobutyloxycarbonyl)phenyl 4-((perfluorohexyl)pentyloxy)benzoate | 115.09 | 105.10 | 106.20 | 8.89 |
| 692 | 4-Methyl-2'-hydroxy-4'-butoxyazobenzene | 84.91 | 75.30 | 76.00 | 8.91 |
| 693 | 4-(1-Methylheptyloxycarbonyl)phenyl 4-(4-decyloxy-3-fluorobenzoyloxy)benzoate | 117.58 | 127.60 | 126.50 | -8.92 |
| 694 | 2-(4-Octyloxyphenyl)-5-(4-decyloxy)phenylazo-1,3,4-thiadiazole | 93.97 | 102.70 | 102.90 | -8.93 |
| 695 | 1-(4'-Propylbicyclohexyl)-3,4,5-trifluorobenzene | 70.85 | 63.10 | 61.90 | 8.95 |
| 696 | Hexane-2,5-diyl bis(4-(4'-octyloxybiphenyl-4-yl)oxymethyl)benzoate) | 107.65 | 100.80 | 98.70 | 8.95 |
| 697 | 4-(Phenoxycarbonyl)phenyl 4-(phenylazo)benzoate | 107.45 | 96.70 | 98.50 | 8.95 |
| 698 | 4-Cyanophenyl 4-((phenylmethylene)amino)benzoate | 88.56 | 79.60 | 79.60 | 8.96 |
| 699 | Biphenyl-4-yl 4-(phenyliminomethyl)benzoate | 92.06 | 82.10 | 83.10 | 8.96 |
| 700 | 6-Decyloxy-2-(4'-hexyloxyphenyliminomethyl)quinoline | 118.53 | 127.70 | 127.50 | -8.97 |
| 701 | 1,3-Phenylene bis(4-(4-dodecyloxybenzoylthio)benzoate) | 148.72 | 156.00 | 157.70 | -8.98 |
| 702 | 6-Decyloxy-2-(4-hexyloxyphenylazo)benzothiazole | 121.89 | 114.70 | 112.90 | 8.99 |
| 703 | Cholesteryl 4-(4-(4-pentylphenylethynyl)phenoxy)butanoate | 71.90 | 81.20 | 80.90 | -9.00 |
| 704 | 4-(4-Methoxybenzylidene)-ethoxyaniline-N-oxide | 91.59 | 100.20 | 100.60 | -9.01 |
| 705 | 4-Cyanophenyl 4-(3-(4-(4'-hexylbiphenyl-4-carbonyloxy)benzoyloxy)benzoyloxy)benzoate | 109.82 | 99.90 | 100.80 | 9.02 |
| 706 | Phenyl 4-((4-cyanophenylimino)methyl)phenyl)benzoate | 71.57 | 79.60 | 80.60 | -9.03 |
| 707 | 2-Butoxy-1-methyl-2-oxoethyl 4-(3-(6-dodecyloxy-2-naphthalenyl)-1-oxo-2-propenyloxy)benzoate | 118.96 | 109.30 | 109.90 | 9.06 |
| 708 | N,N'-Didecanoyl-2,3,5,6-tetrakis(decanoyloxy)-1,4-benzenediamine | 246.93 | 251.30 | 256.00 | -9.07 |
| 709 | Cholesteryl p-phenylazobenzoate | 66.91 | 74.70 | 76.00 | -9.09 |
| 710 | Biphenyl-4-yl 4-(phenylmethyleneamino)benzoate | 91.40 | 82.10 | 82.30 | 9.10 |
| 711 | 4-Chlorophenyl 4-hexyloxythiobenzoate | 73.00 | 81.50 | 82.10 | -9.10 |
| 712 | N-(2-(4-(4-Pentylcyclohexyl)cyclohexyl)ethyl)-3,4-difluoropyrrole | 94.00 | 101.40 | 103.10 | -9.10 |
| 713 | 1,6-Bis(4-(4-octyloxybenzoyloxybenzylideneamino)hexane | 172.51 | 165.50 | 163.40 | 9.11 |
| 714 | Dicholesteryl adipate | 69.79 | 79.10 | 78.90 | -9.11 |
| 715 | 1-Methylheptyl 4'-(4''-undecyloxy-3''-fluorobenzoyloxy)tolan-4-carboxylate | 108.26 | 116.90 | 117.40 | -9.14 |
| 716 | Phenyl 4-(4-phenoxycarbonylphenyliminomethyl)benzoate | 84.52 | 93.00 | 93.70 | -9.18 |
| 717 | 4-(3-(4-(Octyloxy)phenyl)-1-oxo-2-propenyloxy)phenyl 2-chloro-4-methylpentanoate | 84.11 | 91.30 | 93.30 | -9.19 |
| 718 | Cholesteryl thiopentadecanoate | 91.70 | 101.50 | 100.90 | -9.20 |
| 719 | 4-(3-(4-(Undecyloxy)phenyl)-1-oxo-2-propenyloxy)phenyl 2-chloro-4-methylpentanoate | 94.60 | 103.40 | 103.80 | -9.20 |
| 720 | 4-(1-Methylheptyloxycarbonyl)phenyl 4-(4-dodecyloxybenzoylthio)benzoate | 133.54 | 124.10 | 124.30 | 9.24 |
| 721 | 2-Butoxy-1-methyl-2-oxoethyl 4-(3-(6-nonyloxy-2-naphthalenyl)-1-oxo-2-propenyloxy)benzoate | 89.95 | 97.20 | 99.20 | -9.25 |
| 722 | 2-(4-(4-Tetradecyloxybenzoyloxy)-3-chlorophenylazo)naphthalene | 112.33 | 120.30 | 121.60 | -9.27 |
| 723 | 4-Decyl-4''-cyano-p-terphenyl | 99.08 | 89.50 | 89.80 | 9.28 |
| 724 | Phenyl 4-(phenoyxcarbonyl)phenyl 1,4-benzenedicarboxylate | 103.69 | 96.00 | 94.40 | 9.29 |
| 725 | 4-Propylcyclohexylbenzyl perfluorobutyl thioether | 69.20 | 62.30 | 59.90 | 9.30 |
| 726 | 3-(2-Chlorophenyl-5-(4-(4-pentylcyclohexyl)phenyl)-1,2,4-oxadiazole | 76.00 | 63.20 | 66.70 | 9.30 |
| 727 | 4-Nonyloxybiphenylyl 4-(1-methylheptyloxy)thiobenzoate | 106.30 | 98.30 | 97.00 | 9.30 |
| 728 | 1-(4-((3-Buten-1-yl)phenyl)-4-(4-fluorophenyl)-1,3-butadiyne | 66.07 | 75.10 | 75.40 | -9.33 |
| 729 | 1,5-Bis(4-(2-hydroxy-4-butoxybenzylideneamino)benzoyloxy)pentane | 108.74 | 100.50 | 99.40 | 9.34 |
| 730 | 4-(2-(4-(9-Hydroxynonyloxy)phenyl)vinyl)benzonitrile | 111.37 | 101.90 | 102.00 | 9.37 |
| 731 | 3-Phenyl-5-(4-(4-propylcyclohexyl)phenyl)-1,2,4-oxadiazole | 56.72 | 62.30 | 66.10 | -9.38 |
| 732 | 4,4'-Bis(5-(4'-(1-methylheptyloxycarbonyl)biphenyl-4-yl)oxycarbonyl)pentyloxy)biphenyl | 136.29 | 128.30 | 126.90 | 9.39 |
| 733 | 4,4'''-Didodecyloxy-p-quaterphenyl | 148.99 | 139.30 | 139.60 | 9.39 |
| 734 | 2,5-Dihydro-2,5-dimethyl-3,6-bis(4-decyloxyphenyl)pyrrolo[3,4-c]pyrrole-1,4-dione | 189.20 | 173.00 | 179.80 | 9.40 |
| 735 | 2,3-Dimethylbutane | 74.82 | 61.80 | 65.40 | 9.42 |
| 736 | 4'-(2-(4-Propylphenyl)-1-ethynyl)-2'-ethyl-4-ethyltolane | 44.88 | 54.70 | 54.30 | -9.42 |
| 737 | 4-(Phenyliminomethyl)phenyl 4-(benzoyloxy)benzoate | 102.53 | 93.00 | 93.10 | 9.43 |
| 738 | Cholesteryl 4-(4-(4-butylphenylethynyl)phenoxy)butanoate | 86.93 | 77.10 | 77.50 | 9.43 |
| 739 | 2,5-Bis(4-decyloxyphenyl)benzoic anhydride | 177.72 | 185.20 | 187.20 | -9.48 |
| 740 | 2-(4-Octyloxyphenyl)-5-(4-(2-ethyl-2,3-undecadienyloxy)phenyl-1,3,4-thiadiazole | 97.01 | 105.80 | 106.50 | -9.49 |
| 741 | 4-Butyl-4'-hexadecyloxyazobenzene | 135.21 | 144.90 | 144.70 | -9.49 |
| 742 | 4,5-Dichloro-1,3-phenylene bis(4-(4-ethoxybenzylideneamino)benzoate) | 85.23 | 76.00 | 75.70 | 9.53 |
| 743 | 4-(2-Methylbutoxycarbonyl)phenyl 4-(4-butoxy-2,3,4,5-tetrafluorophenylethynyl)benzoate | 75.67 | 85.70 | 85.20 | -9.53 |
| 744 | 1,5-Bis(4-(2-hydroxy-4-butoxybenzylideneamino)benzoyloxy)-2,2-dimethylpentane | 97.07 | 106.60 | 106.60 | -9.53 |
| 745 | 1-Methylpropyl 4-(4-(4-Chlorobenzoyloxy)benzylideneamino)benzoate | 79.35 | 70.30 | 69.80 | 9.55 |
| 746 | 1-(2-(5-Hexylthienyl))-3-(4-chlorophenylamino)-2-propen-1-one | 49.74 | 58.00 | 59.30 | -9.56 |
| 747 | 2-Methylbutyl 4-(4-(4-methylbenzoyloxy)benzylideneamino)benzoate | 69.34 | 80.00 | 78.90 | -9.56 |
| 748 | Pentyl 4-(4-(4-methylbenzoyloxy)benzylideneamino)benzoate | 101.70 | 92.60 | 92.10 | 9.60 |
| 749 | 4-Tetradecyl-N-(4-pentadecyloxyphenylmethylene)benzenamine | 168.97 | 177.60 | 178.60 | -9.63 |
| 750 | Bis(4-ethoxyphenyl) 2-(12-(4-(4-biphenylazo)phenoxy)dodecyloxy) terephthalate | 138.44 | 131.30 | 128.80 | 9.64 |
| 751 | p-Azoxyanisole | 81.47 | 73.20 | 71.80 | 9.67 |
| 752 | N-(7-Oxo-4-(4-(1-decanoylamino)phenyl)-1,3,5-cycloheptatrien-1-yl)hexadecamide | 127.08 | 117.70 | 117.40 | 9.68 |
| 753 | 7-Octyloxy-3-(4-decyloxyphenyl)-3H-1-benzopyran-4-one | 95.90 | 103.60 | 105.60 | -9.70 |
| 754 | N-(7-Oxo-4-(4-(1-octanoylamino)phenyl)-1,3,5-cycloheptatrien-1-yl)octadecamide | 124.02 | 117.70 | 114.30 | 9.72 |
| 755 | Cholesteryl 5-(4-(4-heptylphenylethynyl)phenoxy)pentanoate | 103.54 | 93.30 | 93.80 | 9.74 |
| 756 | 4-(3,4-Bis(4-pentyloxybenzoyloxy)benzylideneamino)azobenzene | 128.74 | 118.40 | 119.00 | 9.74 |
| 757 | 7-(4'-Octadecyloxybenzoyloxy)isoflavone | 128.05 | 119.30 | 118.30 | 9.75 |
| 758 | N-(4-Propoxybenzylidene)-4-hexylaniline | 87.95 | 96.80 | 97.70 | -9.75 |
| 759 | 4-(4-Octyloxyphenyl)acetylene-2,3,5,6-tetrafluorophenyl 4'-chlorobenzoate | 86.15 | 94.70 | 95.90 | -9.75 |
| 760 | 5-(4-Octyloxyphenyl)-2-(4-octyloxy)benzylideneamino-1,3,4-thiadiazole | 100.86 | 90.90 | 91.10 | 9.76 |
| 761 | 5-(4-Decyloxyphenyl)-2-(2-hydroxy-4-heptyloxy)benzylideneamino-1,3,4-thiadiazole | 96.79 | 86.00 | 87.00 | 9.79 |
| 762 | Phenyl 4-(4-propoxybenzylideneamino)benzoate | 99.62 | 89.40 | 89.80 | 9.82 |
| 763 | 4-Chlorophenyl 4-octyloxythiobenzoate | 99.13 | 89.60 | 89.30 | 9.83 |
| 764 | 4-(4-Cyanophenoxycarbonyl)phenyl 3-(4-(4-octadecyloxybenzoyloxy)benzoyloxy)benzoate | 165.16 | 155.20 | 155.30 | 9.86 |
| 765 | 4'-(2-(4-Methylphenyl)-1-ethynyl)-2'-methyl-4-butyltolane | 58.68 | 50.70 | 48.80 | 9.88 |
| 766 | Bis(4-(3-octyloxy-3-oxo-1-propenyl)phenyl terephthalate | 140.19 | 128.00 | 130.30 | 9.89 |
| 767 | Propyl 4-(4-(4-methylbenzoyloxy)benzylideneamino)benzoate | 95.11 | 84.50 | 85.20 | 9.91 |
| 768 | 3,3',4,4'-Biphenyltetracarboxy-N,N'-bis(butoxyphenyl)diimide | 81.47 | 93.10 | 91.40 | -9.93 |
| 769 | 1,3-Phenylene bis(4-(4'-decylbiphenyl-4-carbonyloxy)-2-ethylbenzoate) | 129.06 | 140.20 | 139.00 | -9.94 |
| 770 | 4-Isothiocyanatophenyl 4-(4-hexylcyclohexyl)benzoate | 109.57 | 100.30 | 99.60 | 9.97 |
| 771 | 4-Propoxyphenyl 7-decanoyloxychromone-2-carboxylate | 48.52 | 59.10 | 58.50 | -9.98 |
| 772 | 4-(4-Pentylcyclohexyl)-4'-isothiocyanatotolane | 71.89 | 81.60 | 81.90 | -10.01 |
| 773 | Bis(4-(4-dodecyloxyphenyliminomethyl)phenyl) heptanedioate | 188.67 | 197.10 | 198.70 | -10.03 |
| 774 | 4-Propyl-N-(4-heptyloxyphenylmethylene)benzenamine | 91.63 | 100.90 | 101.70 | -10.07 |
| 775 | 4,6-Dichloro-1,3-phenylene bis(4-(4-nonyloxy-3-fluorophenyliminomethyl)benzoate) | 125.31 | 134.50 | 135.40 | -10.09 |
| 776 | 6-Pentyloxy-2-(4-nonyloxystyryl)quinoline | 115.01 | 105.80 | 104.90 | 10.11 |
| 777 | 6-S-Dodecyl-6-thio-alpha-D-galactopyranose | 121.51 | 108.40 | 111.40 | 10.11 |
| 778 | 4-(2,2,3,3,4,4,5,5-Octafluoropentyloxycarbonyl)phenyl 4-(perfluorohexylpentyloxy)benzoate | 104.83 | 96.20 | 94.70 | 10.13 |
| 779 | 4-Ethylphenyl 4-(4-butoxybenzylideneamino)benzoate | 79.57 | 89.90 | 89.70 | -10.13 |
| 780 | 2-Undecanoyl-1,4-phenylene bis(4-octyloxybenzoate) | 155.76 | 166.00 | 165.90 | -10.14 |
| 781 | 4,4'-Bis(4-(4-hexyloxybenzylideneamino)benzoyloxy)diphenylmethane | 110.54 | 121.10 | 120.70 | -10.16 |
| 782 | 2-Chloro-3-methylpentyl 4-(3-(4-octyloxyphenyl)-1-oxo-2-propenyloxy)benzoate | 98.87 | 88.30 | 88.70 | 10.17 |
| 783 | 1-Methylheptyl 4-(4-(4-heptyloxycinnamoyloxy)benzoyloxy)benzoate | 98.93 | 108.50 | 109.10 | -10.17 |
| 784 | Pentyl 4-(4-dodecyloxybenzylideneamino)cinnamate | 117.71 | 127.10 | 127.90 | -10.19 |
| 785 | Pentyl 4-(4-(4-hexyloxy-2,3,5,6-tetrafluorophenylethynyl)benzyloxy)benzoate | 110.10 | 100.60 | 99.90 | 10.20 |
| 786 | 6-O-Hexadecanoyl-alpha-D-galactopyranose | 113.38 | 100.00 | 103.10 | 10.28 |
| 787 | 4-(omega-Aminooctyloxy)-4'-cyanobiphenyl | 77.49 | 69.20 | 67.20 | 10.29 |
| 788 | 4-Pentyloxybenzylidene 4'-dodecylaniline | 139.90 | 129.10 | 129.60 | 10.30 |
| 789 | 3-Pyridyl 4-(4-heptyloxybenzylideneamino)benzoate | 123.52 | 114.00 | 113.20 | 10.32 |
| 790 | 4-(4-Ethoxyphenylazo)-2,3-dimethylphenyl 4-octyloxybenzoate | 106.44 | 95.70 | 96.10 | 10.34 |
| 791 | 4-(4-(2-Ethoxy-1-methyl-2-oxoethoxycarbonyl)phenylethynyl)phenyl 4-tetradecyloxybenzoate | 125.54 | 115.80 | 115.20 | 10.34 |
| 792 | Cholesteryl 6-phenylhexanoate | 78.25 | 89.00 | 88.60 | -10.35 |
| 793 | 4-(1-Methylheptyloxycarbonyl)phenyl 4-(4-nonyloxybenzoylthio)benzoate | 121.46 | 112.00 | 111.10 | 10.36 |
| 794 | Di-(4-propoxycarbonylphenyl) 2-chloroterephthalate | 81.94 | 92.30 | 92.30 | -10.36 |
| 795 | 4-(1-Methylheptyloxycarbonyl)phenyl 4-(4-heptyloxybenzoylthio)benzoate | 93.93 | 103.90 | 104.30 | -10.37 |
| 796 | 4'-Cyanobiphenyl-4-yl 3-(4-decyloxy)phenoxy)-2-propenoate | 107.98 | 99.10 | 97.60 | 10.38 |
| 797 | 2-Chloro-3-methylpentyl 4-(3-(4-decyloxyphenyl)-1-oxo-2-propenyloxy)benzoate | 106.50 | 96.40 | 96.10 | 10.40 |
| 798 | Phenyl 4-(4-octyloxybenzylideneamino)benzoate | 121.00 | 109.60 | 110.60 | 10.40 |
| 799 | 4-Cyanophenyl 4-(3-(4-(4'-nonylbiphenyl-4-carbonyloxy)-2-fluorobenzoyloxy)benzoyloxy)benzoate | 121.61 | 113.00 | 111.20 | 10.41 |
| 800 | 1,5-Bis(4-(2-hydroxy-4-hexyloxybenzylideneamino)benzoyloxy)-3,3-dimethylpentane | 113.96 | 122.80 | 124.40 | -10.44 |
| 801 | 4-(4'-Decylcyclohexyl)isothiocyanatobenzene | 122.51 | 111.30 | 112.00 | 10.51 |
| 802 | 4-Methyl-2'-hydroxy-4'-undecyloxyazobenzene | 115.92 | 103.60 | 105.40 | 10.52 |
| 803 | 1,5-Bis(4-(2-hydroxy-4-tetradecyloxybenzylideneamino)benzoyloxy)-2,2-dimethylpentane | 178.37 | 187.40 | 188.90 | -10.53 |
| 804 | 4-(3,3,4,4,5,5,6,6,7,7,8,8,8-Tridecafluorooctyloxycarbonyl)phenyl 4-(undecyloxy)benzoate | 124.15 | 133.60 | 134.70 | -10.55 |
| 805 | N-(4-(4-Methoxybenzoyloxy)benzylidene)-4-cyanoaniline | 85.19 | 74.50 | 74.60 | 10.59 |
| 806 | 4-Ethoxyphenyl 4'-pentylbenzoate | 102.20 | 91.70 | 91.60 | 10.60 |
| 807 | 4-Nitrobenzylidene 4'-decylaniline | 112.30 | 103.10 | 101.70 | 10.60 |
| 808 | 4,4'-Diheptyloxyazoxybenzene | 124.60 | 118.90 | 114.00 | 10.60 |
| 809 | 1,3-Phenylene bis(4-(4-nonyloxyphenyliminomethyl)benzoate) | 135.90 | 147.00 | 146.50 | -10.60 |
| 810 | 2-(4-Octyloxyphenyl)-5-(4-octyloxyphenylethynyl)-3,4-thiophenedicarbonitrile | 90.70 | 101.30 | 101.30 | -10.60 |
| 811 | 4,5-Dichloro-1,3-phenylene bis(4-(4-nonyloxybenzylideneamino)benzoate) | 121.29 | 132.50 | 131.90 | -10.61 |
| 812 | Ethyl azoxybenzenedicarboxylate | 66.74 | 84.00 | 77.40 | -10.66 |
| 813 | Cholesteryl 6-(4-(4-pentylphenylethynyl)phenoxy)hexanoate | 78.20 | 89.20 | 88.90 | -10.70 |
| 814 | 4-Chloro-1,3-phenylene bis(4-(4-octyloxybenzylideneamino)benzoate) | 141.71 | 131.70 | 131.00 | 10.71 |
| 815 | 1-(4-Propylcyclohexylethynyl)-4-(4-butylphenylethynyl)benzene | 50.58 | 61.70 | 61.30 | -10.72 |
| 816 | 1-(4-Heptylbiphenyl-4'-yl)-3-(4-chlorophenyl)propane-1,3-dione | 77.38 | 88.00 | 88.10 | -10.72 |
| 817 | Bis(4-(3-heptyloxy-3-oxo-1-propenyl)phenyl terephthalate | 111.17 | 119.90 | 121.90 | -10.73 |
| 818 | 4-Hexyloxyphenyl 7-decanoyloxychromone-2-carboxylate | 78.54 | 71.20 | 67.80 | 10.74 |
| 819 | 4-Tridecyloxybenzylidene-4'-ethylaniline | 109.65 | 121.00 | 120.40 | -10.75 |
| 820 | 4'-Decyloxybiphenyl 4-(2-chloro-3-methylbutanoyloxy)-3-methylbenzoate | 74.74 | 88.00 | 85.50 | -10.76 |
| 821 | 2-(4-Cyanophenyl)-7-heptylfluorene | 51.84 | 62.90 | 62.60 | -10.76 |
| 822 | 4-(4-Dodecyloxyphenylazoxy)phenyl 2-bromo-3-ethylpentanoate | 145.97 | 135.20 |  | 10.77 |
| 823 | 4-Iodo-N-(4-(4-methoxybenzoyloxy)benzylidene)aniline | 44.92 | 44.00 | 55.70 | -10.78 |
| 824 | Bis(4-hexyloxyphenyl) terephthalate | 108.59 | 118.30 | 119.40 | -10.81 |
| 825 | Ethyl 4-(4-(4-(1-oxohexyl)-1-piperazinyl)phenylazo)benzoate | 24.18 | 34.00 | 35.00 | -10.82 |
| 826 | 4-Octyloxy-N-(4-chlorobenzylidene)aniline | 108.55 | 97.20 | 97.70 | 10.85 |
| 827 | Phenyl 4-(phenylmethyleneimino)phenyl 1,4-benzenedicarboxylate | 103.87 | 93.00 | 93.00 | 10.87 |
| 828 | 2-(4-Decyloxyphenyl)-5-(4-decyloxy)phenylazo-1,3,4-thiadiazole | 121.30 | 110.80 | 110.40 | 10.90 |
| 829 | 4,4'-Diethoxyazobenzene | 70.30 | 81.50 | 81.20 | -10.90 |
| 830 | 3-Methylpentyl 4-(4-(4-chlorobenzoyloxy)benzylideneamino)benzoate | 95.81 | 84.50 | 84.90 | 10.91 |
| 831 | 1,8-Bis(4-(4-hexyloxybenzoyloxybenzylideneamino)octane | 146.99 | 157.40 | 157.90 | -10.91 |
| 832 | 4,4'-Bis(4-(4-pentyloxybenzoyloxy)benzylideneamino)diphenylamine | 86.26 | 96.30 | 97.20 | -10.94 |
| 833 | 7-(Undec-10-enyloxy)-3-(4-dodecyloxystyryl)quinoline | 158.64 | 170.10 | 169.60 | -10.96 |
| 834 | 4-(4-Ethoxyphenylazo)-2,3-dimethylphenyl 4-(tetradecyloxy)benzoate | 131.92 | 119.90 | 120.90 | 11.02 |
| 835 | 1-Methylheptyl 5-(4-undecyloxybenzoyloxyphenylethynyl)-thiophene-2-carboxylate | 109.78 | 119.50 | 120.80 | -11.02 |
| 836 | 2-Chloro-3-methylpentyl 4-(3-(4-hexyloxyphenyl)-1-oxo-2-propenyloxy)benzoate | 69.07 | 80.30 | 80.10 | -11.03 |
| 837 | 1,3-Phenylene bis(4-(4'-pentylbiphenyl-4-carbonyloxy)benzoate) | 117.04 | 106.90 | 106.00 | 11.04 |
| 838 | 4-(4-(1-Methylheptyloxycarbonyl)phenyloxycarbonyl)phenyl 4-(8-(4-(4-decyloxybenzoyloxy)benzoyloxy)octyloxy)benzoate | 152.15 | 163.50 | 163.20 | -11.05 |
| 839 | 1,3-Phenylene bis(4-(4'-nonylbiphenyl-4-carbonyloxy)-2-methylbenzoate) | 111.44 | 124.00 | 122.50 | -11.06 |
| 840 | 1-(4'-Ethylbicyclohexyl)-2,3,4-trifluorobenzene | 69.00 | 59.10 | 57.90 | 11.10 |
| 841 | 2,3,6,7,10,11-Hexakis(pentyloxy)triphenylene | 121.51 | 108.40 | 110.40 | 11.11 |
| 842 | 4-t-Butylphenyl 4-(4-octyloxybenzylideneamino)benzoate | 101.89 | 112.10 | 113.00 | -11.11 |
| 843 | 1,3-Phenylene bis(4-(4-decyloxyphenyliminomethyl)benzoate) | 143.09 | 155.10 | 154.20 | -11.11 |
| 844 | 5-(4-Decyloxyphenyl)-2-(2-hydroxy-4-pentyloxy)benzylideneamino-1,3,4-thiadiazole | 89.72 | 77.90 | 78.60 | 11.12 |
| 845 | N-(4-Methoxybenzylidene)-4-pentylaniline | 74.56 | 86.10 | 85.70 | -11.14 |
| 846 | Methyl 4-(4-(4-octyloxy-2,3,5,6-tetrafluorophenylethynyl)benzyloxy)benzoate | 105.46 | 93.90 | 94.30 | 11.16 |
| 847 | 4-((4-Nitrophenylimino)methyl)phenyl benzoate | 92.69 | 79.50 | 81.50 | 11.19 |
| 848 | 4-(4-Ethoxyphenylazo)phenyl 10-undecenoate | 137.32 | 126.00 | 126.10 | 11.22 |
| 849 | 4-Ethylphenyl 4-(4-heptyloxybenzylideneamino)benzoate | 90.57 | 102.00 | 101.80 | -11.23 |
| 850 | 1-(6-(3,6,7,10,11-Pentakis(pentyloxy)triphenylen-2-yloxy)hexyloxy)-9,10-anthraquinone | 80.16 | 93.70 | 91.40 | -11.24 |
| 851 | 4,4'-Dipentyloxyazoxybenzene | 109.16 | 102.80 | 97.90 | 11.26 |
| 852 | 1-Methylheptyl 5-(2,3-difluoro-4-dodecyloxybenzoyloxyphenylethynyl)-thiophene-2-carboxylate | 136.37 | 125.50 | 125.10 | 11.27 |
| 853 | 7-Undec-10-enyloxy-3-(4'-butyoxystyryl)quinoline | 126.21 | 137.70 | 137.50 | -11.29 |
| 854 | 4'-(2-(4-Propylphenyl)-1-ethynyl)-2'-methyl-4-ethyltolane | 60.10 | 50.70 | 48.80 | 11.30 |
| 855 | 4-Methoxyphenyl 4-(4-(4-methylphenyliminomethyl))benzoate | 86.91 | 75.10 | 75.60 | 11.31 |
| 856 | Di(4'-methylphenyl)cyclohexane-1,4-dicarboxylate | 81.11 | 70.90 | 69.80 | 11.31 |
| 857 | N-(4-Pentyloxybenzylidene)-4-aminobenzonitrile | 73.89 | 84.10 | 85.20 | -11.31 |
| 858 | N-(4-Pentyloxybenzylidene)-4-octylaniline | 125.24 | 113.00 | 113.90 | 11.34 |
| 859 | N,N'-Dinonanoyl-2,3,5,6-tetrakis(nonanoyloxy)-1,4-benzenediamine | 218.86 | 227.10 | 230.20 | -11.34 |
| 860 | 2-(1,4,7-Trioxaoctyl)-3,6,7,10,11-pentakis(hexyloxy)triphenylene | 122.75 | 117.30 | 111.40 | 11.35 |
| 861 | 1-Methylheptyl 5-(4-tetradecyloxybenzoyloxyphenylethynyl)-thiophene-2-carboxylate | 142.45 | 131.60 | 131.10 | 11.35 |
| 862 | 1-(4'-Butylbicyclohexyl)-2,3,4-trifluorobenzene | 80.36 | 67.20 | 69.00 | 11.36 |
| 863 | Dicholesteryl sebacate | 106.27 | 95.30 | 94.90 | 11.37 |
| 864 | Phenyl 4-phenylazophenyl terephthalate | 108.19 | 96.70 | 96.80 | 11.39 |
| 865 | 1,3-Phenylene bis(4-(4'-heptylbiphenyl-4-carbonyloxy)-2-ethylbenzoate) | 106.21 | 115.90 | 117.60 | -11.39 |
| 866 | 6-Decyloxy-2-(4-butoxybenzylidenamino)benzothiazole | 116.43 | 103.00 | 105.00 | 11.43 |
| 867 | 1,5-Bis(4-(2-hydroxy-4-nonyloxybenzylideneamino)benzoyloxy)-2,2-dimethylpentane | 134.97 | 147.00 | 146.40 | -11.43 |
| 868 | Cholesteryl 5-(4-hexyloxybiphenyl-4'-oxy)hexanoate | 86.67 | 98.30 | 98.10 | -11.43 |
| 869 | 1-(2-(5-Nonylthienyl))-3-(4-iodophenylamino)-2-propen-1-one | 56.87 | 38.60 | 45.40 | 11.47 |
| 870 | N,N'-Dihexanoyl-2,3,5,6-tetrakis(hexanoyloxy)-1,4-benzenediamine | 145.62 | 154.30 | 157.10 | -11.48 |
| 871 | 4,5-Dichloro-1,3-phenylene bis(4-(4-heptyloxybenzylideneamino)benzoate) | 106.40 | 116.40 | 117.90 | -11.50 |
| 872 | 4-Heptyl-4'-cyanobiphenyl | 96.41 | 83.10 | 84.90 | 11.51 |
| 873 | 2-Cyano-1,3-phenylene bis(4-(4-undecyloxyphenyliminomethyl)benzoate) | 164.42 | 154.90 | 152.90 | 11.52 |
| 874 | 7-(4'-Pentyloxybenzoyloxy)isoflavone | 78.42 | 66.80 | 66.90 | 11.52 |
| 875 | 2-(4-Decyloxyphenylazo)-5-(5'-heptyl-2'-thienyl)-1,3,4-thiadiazole | 90.42 | 101.00 | 102.00 | -11.58 |
| 876 | 4-(4-Ethoxyphenylazo)phenyl 4-tetradecyloxybenzoate | 147.45 | 135.10 | 135.80 | 11.65 |
| 877 | N-(2-(6-Decyloxy)benzothiazolyl)-4-decyloxybenzamide | 119.55 | 108.90 | 107.90 | 11.65 |
| 878 | N-(4-Dodecyloxybenzylidene)-4-hexylaniline | 121.83 | 133.20 | 133.50 | -11.67 |
| 879 | 4-Heptyloxy-4''-cyano-p-terphenyl | 91.00 | 78.60 | 79.30 | 11.70 |
| 880 | 4-Heptyl-4'-undecyloxyazobenzene | 124.90 | 136.80 | 136.60 | -11.70 |
| 881 | 6-S-Decyl-6-thio-alpha-D-galactopyranose | 89.00 | 100.30 | 100.70 | -11.70 |
| 882 | 4-Propylcyclohexyl 4-pentylcinnamate | 60.09 | 70.90 | 71.80 | -11.71 |
| 883 | 4-(4-Hexyloxyphenyl)acetylene-2,3,5,6-tetrafluorophenyl 4'-chlorobenzoate | 98.62 | 86.60 | 86.90 | 11.72 |
| 884 | N-(4-Heptyloxybenzylidene)-4-aminobenzonitrile | 102.62 | 92.20 | 90.90 | 11.72 |
| 885 | 2,3-Difluorobenzoic acid, 1,2,4-oxadiazole-3,5-diylyl-4,1-phenylene ester | 96.13 | 86.70 | 84.40 | 11.73 |
| 886 | 1,10-Bis(4-(4-ethoxybenzoyloxybenzylideneamino)decane | 142.47 | 133.20 | 130.70 | 11.77 |
| 887 | 4-((3-Pyridinimino)methyl)phenyl 4-methoxybenzoate | 79.73 | 91.20 | 91.50 | -11.77 |
| 888 | 5-(4'-Butoxybiphenyl-4-yl)-2-(4-methylphenyl)pyridine | 33.43 | 44.40 | 45.20 | -11.77 |
| 889 | 2-(Perfluorohexyl)ethyl 4-allyloxythiobenzoate | 90.92 | 102.60 | 102.70 | -11.78 |
| 890 | 2,3-Dimethyl-N-(4-(4-methoxybenzoyloxy)benzylidene)aniline | 78.59 | 67.50 | 66.80 | 11.79 |
| 891 | 6-(4-(4-Heptyloxyphenyliminomethyl)-3-(hydroxyphenoxy)hexyl methacrylate | 99.11 | 114.70 | 110.90 | -11.79 |
| 892 | Phenyl 4-(4-pentyloxybenzylideneamino)benzoate | 109.70 | 97.50 | 97.90 | 11.80 |
| 893 | 4,4'-Diheptanoyloxydiphenyldiacetylene | 125.71 | 114.00 | 113.90 | 11.81 |
| 894 | 2-(4-Cyanophenyl)-7-propylfluorene | 35.08 | 46.70 | 46.90 | -11.82 |
| 895 | 1-(2-(5-Cyanothienyl))-3-(4-undecyloxyphenylamino)-2-propen-1-one | 86.44 | 78.40 | 74.60 | 11.84 |
| 896 | 4-(4-Propylcyclohexylethyl)-4-isothiocyanatotolane | 70.06 | 81.60 | 81.90 | -11.84 |
| 897 | 4-(1-Methylheptyloxycarbonyl)-4'-(4-octadecyloxy-3-fluorobenzoyloxy)tolane | 157.45 | 145.20 | 145.60 | 11.85 |
| 898 | 2,5-Bis(4-dodecyloxyphenylethynyl)-3,4-thiophenedicarbonitrile | 140.88 | 129.90 | 129.00 | 11.88 |
| 899 | 1-(6-Pentyloxy-2-naphthyl)-2-(4-fluorophenyl)acetylene | 57.49 | 71.50 | 69.40 | -11.91 |
| 900 | 2-Bromo-6,7,10,11-tetrakis(pentyloxy)-3-pentylthiotriphenylene | 69.58 | 79.00 | 81.50 | -11.92 |
| 901 | Phenyl 4-(biphenyl-4-ylmethyleneamine)benzoate | 95.83 | 82.10 | 83.90 | 11.93 |
| 902 | Di-(4-ethoxycarbonylphenyl) 2-chloroterephthalate | 94.93 | 84.20 | 83.00 | 11.93 |
| 903 | 4-(4-Pentoxyphenyl)acetylene-2,3,5,6-tetrafluorophenyl 4'-chlorobenzoate | 93.34 | 82.60 | 81.40 | 11.94 |
| 904 | 4,6-Dichloro-1,3-phenylene bis(4-(4-hexyloxyphenyliminomethyl)benzoate) | 119.55 | 108.30 | 107.60 | 11.95 |
| 905 | 1-(4'-Hexylazobenzene-4-oxy)-6-(4'-butylazobenzene-4-oxy)hexane | 119.55 | 130.20 | 131.50 | -11.95 |
| 906 | 4-(4-(4-(4-Heptyloxybenzoyloxy)benzoyl)-1-piperazinyl)phenyl 4-(4-heptyloxybenzoyloxy)benzoate | 53.33 | 64.70 | 65.30 | -11.97 |
| 907 | Bis(10-(4-(4-butoxyphenyliminomethyl)phenoxy)decyl) malonate | 169.92 | 185.50 | 181.90 | -11.98 |
| 908 | 4,4'-Dipropanoyloxydiphenyldiacetylene | 70.71 | 81.70 | 82.70 | -11.99 |
| 909 | 1-((4-Penten-1-yl)phenyl)-4-(4-ethylphenyl)-1,3-butadiyne | 67.67 | 74.50 | 79.70 | -12.03 |
| 910 | 1-(4'-Hexylazobenzene-4-oxy)-6-(azobenzene-4-oxy)hexane | 138.67 | 125.70 | 126.60 | 12.07 |
| 911 | 3-Methylpentyl 4-(4-(4-methoxybenzylideneamino)benzoyloxy)benzoate | 98.67 | 86.60 | 86.60 | 12.07 |
| 912 | 2-Heptanoyl-1,4-phenylene bis(4-heptyloxybenzoate) | 152.80 | 141.70 | 140.70 | 12.10 |
| 913 | 4-(2-Methylbutoxycarbonyl)phenyl 4-(4-dodecyloxy-2,3,4,5-tetrafluorophenylethynyl)benzoate | 104.19 | 118.00 | 116.30 | -12.11 |
| 914 | N-(4-Dodecyloxybenzylidene)-4-octylaniline | 153.83 | 141.20 | 141.70 | 12.13 |
| 915 | N,N'-Diheptanoyl-2,3,5,6-tetrakis(heptanoyloxy)-1,4-benzenediamine | 166.17 | 178.60 | 178.30 | -12.13 |
| 916 | 2-Chloro-4-methylpentyl 4-(3-(4-dodecyloxyphenyl)-1-oxo-2-propenyloxy)benzoate | 117.25 | 104.50 | 105.10 | 12.15 |
| 917 | 4-Methoxyphenyl 4-(4-(4-bromophenylimino)methyl)benzoate | 96.25 | 81.90 | 84.10 | 12.15 |
| 918 | 1-(2-(5-Nonylthienyl))-3-(4-fluorophenylamino)-2-propen-1-one | 94.16 | 78.40 | 82.00 | 12.16 |
| 919 | Bis(4-butoxyphenyl) 4,4'-biphenyldicarboxylate | 83.32 | 96.40 | 95.50 | -12.18 |
| 920 | 4-(3-Ethylmercapto-2-methylpropionyloxy)phenyl 4'-decyloxybiphenyl-4-carboxylate | 94.59 | 105.40 | 106.80 | -12.21 |
| 921 | 1-(4'-Pentylazobenzene-4-oxy)-6-(4'-methylazobenzene-4-oxy)hexane | 126.62 | 114.00 | 114.40 | 12.22 |
| 922 | 1,9-Bis(4-(4-ethoxybenzoyloxy)benzylideneamino)nonane | 116.97 | 129.20 | 129.20 | -12.23 |
| 923 | 1,3-Bis(4-(2-hydroxy-4-pentyloxybenzylideneamino)benzoyloxy)benzene | 109.25 | 96.70 | 97.00 | 12.25 |
| 924 | Bis(4-heptyloxyphenyl) terephthalate | 138.36 | 126.40 | 126.10 | 12.26 |
| 925 | N,N'-Didodecylbenzene-1,2-dicarboxamide | 177.76 | 176.20 | 165.50 | 12.26 |
| 926 | 4-(Phenylmethyleneamino)phenyl 4-cyanobenzoate | 67.28 | 79.60 | 79.60 | -12.32 |
| 927 | 4-Cyanobenzyl 5-(4-octyloxybenzoyloxy)-2-undecyloxybenzoate | 125.57 | 135.40 | 137.90 | -12.33 |
| 928 | 4-Methoxyphenyl 4-(4-(4-chlorophenylimino)methyl)benzoate | 89.54 | 75.50 | 77.20 | 12.34 |
| 929 | 4-(4-Ethoxyphenylazo)-2,3-dimethylphenyl 4-(decyloxy)benzoate | 93.55 | 103.70 | 105.90 | -12.35 |
| 930 | 4-(3,3,4,4,5,5,6,6,7,7,8,8,8-Tridecafluorooctyloxycarbonyl)phenyl 4-((perfluorohexyl)pentyloxy)benzoate | 110.45 | 123.10 | 122.80 | -12.35 |
| 931 | 2,6,10-Tris(hexyloxy)-3,7,11-tris(butoxy)triphenylene | 120.77 | 108.40 | 108.40 | 12.37 |
| 932 | 11-(Pentakis(4-pentylphenylethynyl)phenoxy)undecanoic acid | 122.60 | 128.80 | 135.00 | -12.40 |
| 933 | 4-(3,3,4,4,5,5,6,6,7,7,8,8,8-tridecafluorooctyloxycarbonyl)phenyl 4-(heptyloxy)benzoate | 104.39 | 117.40 | 116.80 | -12.41 |
| 934 | Isopropyl 4-(4-(4-Chlorobenzoyloxy)benzylideneamino)benzoate | 77.83 | 66.30 | 65.40 | 12.43 |
| 935 | Phenyl 4-(biphenyl-4-yliminomethyl)benzoate | 94.06 | 82.10 | 81.60 | 12.46 |
| 936 | 4-(2-Ethylheptyloxycarbonyl)phenyl 4-undecanoyloxytolane-4'-carboxylate | 118.92 | 131.70 | 131.40 | -12.48 |
| 937 | 1,2-Dimethylpropyl 4-(4-(4-acetoxybenzoyloxy)benzylideneamino)benzoate | 84.29 | 72.30 | 71.80 | 12.49 |
| 938 | (1-Butoxycarbonyl)ethyl 5-(3-fluoro-4-octadecyloxybenzoyloxyphenylethynyl)-thiophene-2-carboxylate | 133.21 | 144.60 | 145.70 | -12.49 |
| 939 | Di-(4-methoxycarbonylphenyl) 2-chloroterephthalate | 91.34 | 78.90 | 78.80 | 12.54 |
| 940 | 1,7-Bis(4-(4-ethoxybenzoyloxy)benzylideneamino)heptane | 110.43 | 121.10 | 123.00 | -12.57 |
| 941 | 1-(4-Cyanophenyl)-4-(4-pentylphenyl)-1,3-butadiyne | 79.29 | 67.40 | 66.70 | 12.59 |
| 942 | 1,3-Phenylene bis(4-(4'-decylbiphenyl-4-carbonyloxy)-2-methylbenzoate) | 146.51 | 132.10 | 133.90 | 12.61 |
| 943 | 6-O-Decanoyl-alpha-D-galactopyranose | 81.09 | 93.60 | 93.70 | -12.61 |
| 944 | 2,3,6,7-Tetrakis(pentyloxy)-1,5-bis(2-(tetrahydropyran-2-yloxy)ethoxy)-9,10-anthraquinone | 100.77 | 109.50 | 113.40 | -12.63 |
| 945 | 2-Octanoyl-1,4-phenylene bis(4-hexyloxybenzoate) | 152.55 | 137.70 | 139.90 | 12.65 |
| 946 | 4,4'-Bis(4-(4-butoxybenzylideneamino)benzoyloxy)diphenylmethane | 93.05 | 105.00 | 105.70 | -12.65 |
| 947 | 1-(4-((5-Hexen-1-yl)phenyl)-4-(4-fluorophenyl)-1,3-butadiyne | 70.04 | 83.10 | 82.70 | -12.66 |
| 948 | 4-(2-Methylbutoxy)phenyl 4-(4-pentyloxy-2,3,5,6-tetrafluorophenylethynyl)benzoate | 76.22 | 87.00 | 88.90 | -12.68 |
| 949 | 4-(4-Hexyloxy-2,3,5,6-tetrafluorophenylacetylenyl)phenyl 4'-chlorobenzoate | 98.09 | 86.60 | 85.40 | 12.69 |
| 950 | 1-(4-Fluorophenyl)-4-(4-propylphenyl)-1,3-butadiyne | 56.71 | 68.50 | 69.40 | -12.69 |
| 951 | 4-Iodophenyl cholest-5-ene-3beta-carboxylate | 26.74 | 43.10 | 39.50 | -12.76 |
| 952 | 1-(6-Octyloxy-2-naphthyl)-4-(4-fluorophenyl)diacetylene | 66.93 | 79.90 | 79.70 | -12.77 |
| 953 | 4-Nitrophenyl 3-(4'-decyloxybiphenyl-4-yloxy)-2-propenoate | 108.39 | 99.10 | 95.60 | 12.79 |
| 954 | 6-O-tetradecyl-D-galacitol | 128.71 | 140.10 | 141.50 | -12.79 |
| 955 | 5-(4-Decyloxyphenyl)-2-(2-hydroxy-4-hexyloxy)benzylideneamino-1,3,4-thiadiazole | 94.10 | 82.00 | 81.30 | 12.80 |
| 956 | 4-Heptylcyclohexyl 4-butoxycinnamate | 74.00 | 84.20 | 86.80 | -12.80 |
| 957 | 1,3-Phenylene bis(methylideneamino-4,1-phenylene) 4'-decyloxybiphenyl-4-carboxylate | 128.89 | 143.70 | 141.70 | -12.81 |
| 958 | 4'-(4-Pentylcyclohexylethyl)-4-isothiocyanatotolane | 77.18 | 89.70 | 90.00 | -12.82 |
| 959 | 6-Decyloxy-2-(4'-octyloxyphenyliminomethyl)quinoline | 149.25 | 135.80 | 136.40 | 12.85 |
| 960 | 4-Propoxyphenyl 3-fluoro-4-thiocyanatophenyl terephthalate | 94.58 | 82.80 | 81.70 | 12.88 |
| 961 | 4-Thiocyanophenyl 4-(4-heptyloxybenzoyloxy)benzoate | 87.32 | 97.90 | 100.20 | -12.88 |
| 962 | 4-(4-(1-Methylheptyloxycarbonyl)phenyloxycarbonyl)phenyl 4-(5-(4-(4-tridecyloxybenzoyloxy)benzoyloxy)pentyloxy)benzoate | 177.40 | 163.50 | 164.50 | 12.90 |
| 963 | Cholesteryl thiotetradecanoate | 91.70 | 104.80 | 104.60 | -12.90 |
| 964 | 4-Pentyl-4'-tetradecyloxyazobenzene | 129.63 | 140.90 | 142.60 | -12.97 |
| 965 | 1,6-Bis(4-(4-heptyloxybenzoyloxybenzylideneamino)hexane | 171.18 | 157.40 | 158.20 | 12.98 |
| 966 | 4-Heptyl-4'-thiocyanatobiphenyl | 67.02 | 80.70 | 80.00 | -12.98 |
| 967 | Cholesteryl 7-phenylheptanoate | 106.80 | 93.10 | 93.80 | 13.00 |
| 968 | 4-(1-Methylheptyloxycarbonyl)phenyl 4-(4-nonyloxyphenylethyl)benzoate | 107.30 | 119.70 | 120.30 | -13.00 |
| 969 | 2-Fluoro-4-octyloxyphenyl 4-decyloxythiobenzoate | 141.82 | 127.70 | 128.80 | 13.02 |
| 970 | 5-(4-Hexyloxyphenyl)-2-(4-octyloxy)benzylideneamino-1,3,4-thiadiazole | 95.03 | 82.90 | 82.00 | 13.03 |
| 971 | 4'-(2-(4-Pentylphenyl)-1-ethynyl)-2'-methyl-4-ethyltolane | 46.31 | 58.70 | 59.40 | -13.09 |
| 972 | 4-n-Octyloxycinnamic acid | 122.70 | 107.80 | 109.60 | 13.10 |
| 973 | 4-(4-Ethylcyclohexyl)-4'-nitrotolane | 59.90 | 48.70 | 46.80 | 13.10 |
| 974 | 4-Isopropylphenyl 4-(4-butoxybenzylideneamino)benzoate | 68.89 | 81.40 | 82.00 | -13.11 |
| 975 | 4-(1-Methylheptyloxycarbonyl)phenyl 4-(4-octadecyloxybenzoylthio)benzoate | 136.05 | 148.30 | 149.20 | -13.15 |
| 976 | 4-Chlorobenzylidene 4'-octylaniline | 109.86 | 96.00 | 96.70 | 13.16 |
| 977 | 4-(4-Ethoxyphenylazo)-2-methylphenyl 4-dodecyloxybenzoate | 105.83 | 119.40 | 119.00 | -13.17 |
| 978 | 6,7,10,11-Tetrakis(pentyloxy)-3-pentylsulphanyl-2-cyanotriphenylene | 83.17 | 71.60 | 70.00 | 13.17 |
| 979 | 1-(4-Dodecyloxybiphenyl-4'-yl)-3-(4-cyanobiphenyl)propane-1,3-dione | 118.28 | 102.70 | 105.10 | 13.18 |
| 980 | 4-(2-Methylbutoxy)phenyl 4-(4-propoxy-2,3,5,6-tetrafluorophenylethynyl)benzoate | 67.40 | 78.90 | 80.60 | -13.20 |
| 981 | 5-(4-Nonyloxyphenyl)-N-(5-octyl-2-thienylmethylene)-1,3,4-thiadiazole-2-amine | 111.03 | 97.30 | 97.80 | 13.23 |
| 982 | 1,6-Bis(4-(4-butoxybenzoyloxybenzylideneamino)hexane | 147.54 | 133.20 | 134.30 | 13.24 |
| 983 | 4-Methoxyphenyl 4-(phenyliminomethyl)benzoate | 94.97 | 82.70 | 81.70 | 13.27 |
| 984 | 4-Pentanoyl-4'-pentadecanoyloxyazobenzene | 163.18 | 149.40 | 149.90 | 13.28 |
| 985 | 4-(4-Ethoxybenzoyloxy)-2-octyloxy-4'-(4-butyoxysalicylaldimino)azobenzene | 123.79 | 109.80 | 110.50 | 13.29 |
| 986 | 4'-(2-(4-Ethylphenyl)-1-ethynyl)-2'-ethyl-4-propyltolane | 42.60 | 54.70 | 55.90 | -13.30 |
| 987 | 4-Nonyl-4''-cyano-p-terphenyl | 72.38 | 85.50 | 85.70 | -13.32 |
| 988 | N-(4-Ethoxybenzylidene)-4-pentylaniline | 76.13 | 88.70 | 89.50 | -13.37 |
| 989 | Di(4'-heptylphenyl)-cyclohexane-1,4-dicarboxylate | 107.73 | 119.30 | 121.10 | -13.37 |
| 990 | 4-(1-Methylheptyloxycarbonyl)phenyl 4-(4-octyloxyphenylethyl)benzoate | 101.70 | 115.70 | 115.10 | -13.40 |
| 991 | 4-Isothiocyanatophenyl 4-(4-nonylcyclohexyl)benzoate | 100.20 | 112.40 | 113.60 | -13.40 |
| 992 | 4,4'''-Ditetradecyloxy-p-quaterphenyl | 169.01 | 155.50 | 155.60 | 13.41 |
| 993 | 4,4'-Diundecyloxyazoxybenzene | 141.59 | 151.30 | 155.00 | -13.41 |
| 994 | 3-(4-Decyloxyphenyl)-5-(4-bromophenyl)pyrazole | 123.72 | 114.70 | 110.30 | 13.42 |
| 995 | 7-(4'-Butoxybenzoyloxy)isoflavone | 76.32 | 62.70 | 62.90 | 13.42 |
| 996 | 1-(4-Propylcyclohexylethynyl)-4-(4-propylphenylethynyl)benzene | 44.47 | 57.60 | 57.90 | -13.43 |
| 997 | 4-Cyanophenyl 4-(3-(4-(4'-dodecylbiphenyl-4-carbonyloxy)-2-fluorobenzoyloxy)benzoyloxy)benzoate | 111.40 | 125.10 | 124.90 | -13.50 |
| 998 | 2-Cyano-1,3-phenylene bis(4-(4-dodecyloxyphenyliminomethyl)benzoate) | 149.79 | 163.00 | 163.30 | -13.51 |
| 999 | 4-(2-Chloro-3-methylpentanoyloxy)biphenyl-4'-yl 4-decyloxy-2,3-difluorobenzoate | 88.17 | 101.70 | 101.70 | -13.53 |
| 1000 | 1-Methylheptyl 4'-(4''-nonyloxy- 2'',3''-difluorobenzoyloxy)tolan-4-carboxylate | 124.24 | 109.90 | 110.70 | 13.54 |
| 1001 | 1-(6-Hexyloxy-2-naphthyl)-2-(4-fluorophenyl)acetylene | 63.36 | 75.60 | 76.90 | -13.54 |
| 1002 | N,N'-Diundecanoyl-2,5,6-trimethyl-4-undecanoyloxy-1,3-benzenediamine | 118.94 | 133.80 | 132.50 | -13.56 |
| 1003 | 4-Isopropylphenyl 4-(4-heptyloxybenzylideneamino)benzoate | 104.67 | 93.50 | 91.10 | 13.57 |
| 1004 | 1-Methylheptyl 4-(4-(4-decyloxycinnamoyloxy)benzoyloxy)benzoate | 107.11 | 120.60 | 120.70 | -13.59 |
| 1005 | 4-(2-Methylbutoxy)phenyl 4-(4-ethoxy-2,3,5,6-tetrafluorophenylethynyl)benzoate | 87.00 | 74.80 | 73.40 | 13.60 |
| 1006 | 4-(2,2,3,3,4,4,4-Heptafluorobutyloxycarbonyl)phenyl 4-(3-perfluorobutyl)propoxy)benzoate | 74.60 | 87.70 | 88.20 | -13.60 |
| 1007 | 1,3-Phenylene bis(4-(4'-octylbiphenyl-4-carbonyloxy)-2-fluorobenzoate) | 122.08 | 133.20 | 135.70 | -13.62 |
| 1008 | 4-(4-Ethylcyclohexylethynyl)-4'-propyltolane | 42.26 | 53.60 | 55.90 | -13.64 |
| 1009 | 4-(4-(4-(4-Nonyloxybenzoyloxy)benzoyl)-1-piperazinyl)phenyl 4-(4-nonyloxybenzoyloxy)benzoate | 68.15 | 80.90 | 81.80 | -13.65 |
| 1010 | 4-Chloro-N-(4-(4-methoxybenzoyloxy)benzylidene)aniline | 63.52 | 75.50 | 77.20 | -13.68 |
| 1011 | 4-Heptyloxyphenyl 7-decanoyloxychromone-2-carboxylate | 64.41 | 75.30 | 78.10 | -13.69 |
| 1012 | 1,6-Bis(4-nitroazobenzene-4'-oxy)hexane | 104.01 | 96.50 | 90.30 | 13.71 |
| 1013 | 2,2-Dimethylpropyl 4-(4-(4-cyanobenzoyloxy)benzylideneamino)benzoate | 76.38 | 90.00 | 90.10 | -13.72 |
| 1014 | 4-(Phenylazo)phenyl 4-(phenyliminomethyl)benzoate | 108.03 | 93.70 | 94.30 | 13.73 |
| 1015 | 1-(4-Decylbiphenyl-4'-yl)-3-(4-cyanobiphenyl)propane-1,3-dione | 80.56 | 93.50 | 94.30 | -13.74 |
| 1016 | (1-Butoxycarbonyl)ethyl 5-(3-fluoro-4-heptyloxybenzoyloxyphenylethynyl)-thiophene-2-carboxylate | 116.06 | 100.10 | 102.30 | 13.76 |
| 1017 | 4-(4-Ethoxyphenylazo)-2,3-dimethylphenyl 4-dodecyloxybenzoate | 99.34 | 111.80 | 113.10 | -13.76 |
| 1018 | 4-(4-Iodophenyl)-2,6-bis(3,4,5-trioctyloxyphenyl)pyrimidine | 80.34 | 94.80 | 94.10 | -13.76 |
| 1019 | 4-(Phenylmethyleneimino)phenyl 4-(benzoyloxy)benzoate | 79.31 | 93.00 | 93.10 | -13.79 |
| 1020 | 5-(4-Pyridyl)-2-(4-decyloxybenzylideneamino)-1,3,4-thiadiazole | 72.31 | 85.60 | 86.10 | -13.79 |
| 1021 | 3-(4-(4-Cyanophenoxycarbonyl)phenoxycarbonyl)phenyl 2-fluoro-4-(4-hexadecyloxybenzoyloxy)benzoate | 161.10 | 148.10 | 147.30 | 13.80 |
| 1022 | 4-Dodecyl-4'-cyanobiphenyl | 116.50 | 103.30 | 102.70 | 13.80 |
| 1023 | 5-(2-Chlorophenyl)-3-(4'-pentylbiphenyl-4-yl)-1,2,4-oxadiazole | 77.30 | 67.90 | 63.50 | 13.80 |
| 1024 | N-(2-Hydroxy-4-methoxybenzylidene)-4'-(4''-dodecylphenyl)aniline | 114.12 | 99.70 | 100.30 | 13.82 |
| 1025 | N-(4-Octyloxybenzylidene)-4-aminobenzonitrile | 108.75 | 96.20 | 94.90 | 13.85 |
| 1026 | 2-Methylpentyl 4-(4-(4-methoxybenzoyloxy)benzylideneamino)benzoate | 99.77 | 86.60 | 85.90 | 13.87 |
| 1027 | 3-Fluoro-N-(4-(4-methoxybenzoyloxy)benzylidene)aniline | 96.49 | 83.70 | 82.60 | 13.89 |
| 1028 | 5-(4-Decyloxyphenyl)-2-(2-hydroxy-4-octyloxy)benzylideneamino-1,3,4-thiadiazole | 92.60 | 107.90 | 106.50 | -13.90 |
| 1029 | 4-Octyloxybiphenylyl 4-(1-methylheptyloxy)thiobenzoate | 80.08 | 94.30 | 94.00 | -13.92 |
| 1030 | 4-Propanoyl-4'-hexanoyloxyazobenzene | 91.68 | 104.90 | 105.60 | -13.92 |
| 1031 | 1-(4-Ethylcyclohexylethynyl)-4-(4-pentylphenylethynyl)benzene | 48.45 | 61.70 | 62.40 | -13.95 |
| 1032 | Isobutyl 4-(4-(4-Nitrobenzoyloxy)benzylideneamino)benzoate | 89.86 | 75.30 | 75.90 | 13.96 |
| 1033 | 4-(4-Tetradecyloxyphenylazoxy)phenyl 2-bromo-3-methylpentanoate | 153.17 | 139.20 |  | 13.97 |
| 1034 | 1-(4-Decyloxybiphenyl-4'-yl)-3-(4-cyanobiphenyl)propane-1,3-dione | 106.57 | 94.60 | 92.60 | 13.97 |
| 1035 | 4-Pentyloxy-4'-isothiocyanatotolane | 106.92 | 93.10 | 92.90 | 14.02 |
| 1036 | 2-(4-Cyanophenyl)-7-octylfluorene | 56.63 | 66.90 | 70.70 | -14.07 |
| 1037 | 4-Pentanoyl-4'-octadecanoyloxyazobenzene | 174.79 | 161.50 | 160.70 | 14.09 |
| 1038 | 6-O-Decyl-D-galacitol | 137.71 | 123.90 | 123.60 | 14.11 |
| 1039 | 1-(4-Butylcyclohexylethynyl)-4-(4-butylphenylethynyl)benzene | 50.89 | 65.70 | 65.00 | -14.11 |
| 1040 | 4-Pentanoyl-4'-undecanoyloxyazobenzene | 119.38 | 133.20 | 133.50 | -14.12 |
| 1041 | 4-Butyl-N-(4-pentadecyloxyphenylmethylene)benzenamine | 123.55 | 137.20 | 137.70 | -14.15 |
| 1042 | 4-(4-(4-(4-Decyloxybenzoyloxy)benzoyl)-1-piperazinyl)phenyl 4-(4-decyloxybenzoyloxy)benzoate | 75.24 | 89.00 | 89.40 | -14.16 |
| 1043 | 2-(Perfluorooctyl)ethyl 4-phenylthiobenzoate | 92.44 | 102.20 | 106.60 | -14.16 |
| 1044 | 3-Hydroxybutyl 4-((4'-octyloxybiphenyl-4-yl)oxymethyl)benzoate | 116.57 | 101.30 | 102.40 | 14.17 |
| 1045 | N-(4-Ethoxybenzylidene)-4-heptylaniline | 82.91 | 96.80 | 97.10 | -14.19 |
| 1046 | 4-(3,4-Bis(4-butoxybenzoyloxy)benzylideneamino)azobenzene | 96.60 | 110.30 | 110.80 | -14.20 |
| 1047 | 4-Cyanophenyl 4-(3-(4-(4'-tetradecylbiphenyl-4-carbonyloxy)benzoyloxy)benzoyloxy)benzoate | 146.41 | 132.20 | 132.20 | 14.21 |
| 1048 | 4-Ethylphenyl 4-(4-ethoxybenzylideneamino)benzoate | 95.63 | 81.80 | 81.40 | 14.23 |
| 1049 | 4-Octyloxy-N-(4-nitrobenzylidene)aniline | 109.43 | 96.20 | 95.20 | 14.23 |
| 1050 | 1,8-Bis(4-(4-heptyloxybenzoyloxybenzylideneamino)octane | 152.07 | 165.50 | 166.30 | -14.23 |
| 1051 | 1-Methylheptyl 4-(4-(4-undecyloxy-3-fluorobenzoyloxy)-3-fluorobenzoyloxy)benzoate | 116.95 | 132.60 | 131.20 | -14.25 |
| 1052 | 2-Cyano-1,3-phenylene bis(4-(4'-dodecylbiphenyl-4-carbonyloxy)-3-fluorobenzoate) | 172.83 | 157.30 | 158.50 | 14.33 |
| 1053 | 4-Cyanophenyl 4-(3-(4-(4'-octylbiphenyl-4-carbonyloxy)-3-fluorobenzoyloxy)benzoyloxy)benzoate | 94.46 | 109.00 | 108.80 | -14.34 |
| 1054 | 1-Methylbutyl 4-(4-(4-methoxybenzoyloxy)benzylideneamino)benzoate | 89.56 | 76.50 | 75.20 | 14.36 |
| 1055 | 1,5-Bis(4-(2-hydroxy-4-hexyloxybenzylideneamino)benzoyloxy)pentane | 129.69 | 116.70 | 115.30 | 14.39 |
| 1056 | N-(4-Pentyloxybenzylidene)-4-hexylaniline | 89.91 | 104.90 | 104.30 | -14.39 |
| 1057 | 4-(4-Ethoxyphenylazo)-3,5-dimethylphenyl 4-(decyloxy)benzoate | 120.32 | 103.70 | 105.90 | 14.42 |
| 1058 | Phenyl 4-(4-heptyloxybenzylideneamino)benzoate | 119.94 | 105.60 | 105.50 | 14.44 |
| 1059 | 4-(3-Pyridiniminomethyl)phenyl 4-heptyloxybenzoate | 127.65 | 114.00 | 113.20 | 14.45 |
| 1060 | (1-Butoxycarbonyl)ethyl 5-(3-fluoro-4-tetradecyloxybenzoyloxyphenylethynyl)-thiophene-2-carboxylate | 114.53 | 128.40 | 129.00 | -14.47 |
| 1061 | 4-(Benzoyloxy)phenyl 4-(phenylmethyleneimino)benzoate | 78.63 | 93.00 | 93.10 | -14.47 |
| 1062 | 1,2,13,14-Tetrahydroxytetradecane | 131.33 | 144.00 | 145.80 | -14.47 |
| 1063 | 1-(4-Trifluoromethylphenyl)-4-((4-penten-1-yl)phenyl)-1,3-butadiyne | 45.38 | 56.80 | 59.90 | -14.52 |
| 1064 | 6-(4-(4-Octyloxyphenyliminomethyl)-3-(hydroxyphenoxy)hexyl methacrylate | 135.73 | 118.70 | 121.20 | 14.53 |
| 1065 | 2-(4-Cyanophenyl)-7-nonylfluorene | 58.67 | 71.00 | 73.20 | -14.53 |
| 1066 | 2,5,2',5'-Tetrakis(4-decyloxyphenyl)azobenzene | 197.94 | 183.20 | 183.40 | 14.54 |
| 1067 | 4-(Benzoyloxy)phenyl 4-(benzoyloxy)benzoate | 81.43 | 96.00 | 96.00 | -14.57 |
| 1068 | 3-(4-(4-Heptylcyclohexyl)phenyl)-5-(4-heptyloxyphenyl)isoxazole | 72.12 | 88.40 | 86.70 | -14.58 |
| 1069 | 2,5-Thiophenediylbis(ethynediyl-4,1-phenylene) 3,4-bis(decyloxy)benzoate | 181.81 | 195.00 | 196.40 | -14.59 |
| 1070 | 2-(4-Pentyloxy)phenyl-5-(2-octylthien-5-yl)methyleneimino-1,3,4-thiadiazole | 96.92 | 81.20 | 82.30 | 14.62 |
| 1071 | 4-(2-Naphthalenylazo)-1-naphthalenyl 4-hexadecyloxybenzoate | 107.98 | 125.50 | 122.60 | -14.62 |
| 1072 | Pentyl 4-(4-(4-butoxy-2,3,5,6-tetrafluorophenylethynyl)benzyloxy)benzoate | 78.97 | 92.60 | 93.60 | -14.63 |
| 1073 | 1,11-Bis(4'-pentylazobenzene-4-oxy)undecane | 164.44 | 150.40 | 149.80 | 14.64 |
| 1074 | 4-(2,2,3,3,4,4,5,5-octafluoropentyloxycarbonyl)phenyl 4-(octyloxy)benzoate | 80.84 | 94.60 | 95.50 | -14.66 |
| 1075 | 6-(4-(4-Hexyloxyphenyliminomethyl)-3-(hydroxyphenoxy)hexyl methacrylate | 131.17 | 110.60 | 116.50 | 14.67 |
| 1076 | 5-(4-Decyloxyphenyl)-2-(4-octyloxy)benzylideneamino-1,3,4-thiadiazole | 112.99 | 99.00 | 98.30 | 14.69 |
| 1077 | 4'-(2-(4-Pentylphenyl)-1-ethynyl)-2'-ethyl-4-ethyltolane | 47.70 | 62.80 | 62.40 | -14.70 |
| 1078 | Isobutyl 4-(4-(4-methylbenzoyloxy)benzylideneamino)benzoate | 91.02 | 76.00 | 76.30 | 14.72 |
| 1079 | 3,3',4,4'-Biphenyltetracarboxy-N,N'-bis(butylphenyl)diimide | 77.38 | 90.80 | 92.10 | -14.72 |
| 1080 | 4-Isopropylphenyl 4-(4-octyloxybenzylideneamino)benzoate | 82.65 | 97.50 | 97.40 | -14.75 |
| 1081 | 2-Cyano-1,3-phenylene bis(4-(4'-octadecylbiphenyl-4-carbonyloxy)-2-fluorobenzoate) | 221.37 | 205.70 | 206.60 | 14.77 |
| 1082 | 4-(Phenoxycarbonyl)phenyl biphenyl-4-carboxylate | 98.58 | 85.10 | 83.80 | 14.78 |
| 1083 | 4-Cyanophenyl 4-(3-(4-(4'-octylbiphenyl-4-carbonyloxy)-2-fluorobenzoyloxy)benzoyloxy)benzoate | 124.88 | 109.00 | 110.10 | 14.78 |
| 1084 | 1,6-Bis(4-octyloxyazobenzene-4'-carbonyloxy)hexane | 177.09 | 162.20 | 162.30 | 14.79 |
| 1085 | 4'-Tetradecyloxy-3'-nitrobiphenyl-4-carboxylic acid | 106.81 | 124.00 | 121.60 | -14.79 |
| 1086 | Phenyl 4-(4-(phenylazo)phenyliminomethyl)benzoate | 79.81 | 93.70 | 94.60 | -14.79 |
| 1087 | 1-Methylheptyl 4-(2-fluoro-4-(4-undecyloxybenzoyloxy)phenylethynyl)benzoate | 132.10 | 116.90 | 117.30 | 14.80 |
| 1088 | 4-Thiocyanophenyl 4-(4-hexyloxybenzoyloxy)benzoate | 78.67 | 93.90 | 93.50 | -14.83 |
| 1089 | 7-(Undec-10-enyloxy)-3-(4-decyloxystyryl)quinoline | 146.66 | 162.00 | 161.50 | -14.84 |
| 1090 | 1,3-Phenylene bis(4-(4-ethoxyphenyliminomethyl)benzoate) | 106.45 | 90.40 | 91.60 | 14.85 |
| 1091 | 4-Decyloxybiphenyl-4'-yl 4-(2-dodecyloxypropanoyl-3-methylbenzoate | 119.73 | 132.90 | 134.60 | -14.87 |
| 1092 | 1-Methylheptyl 5-(4-hexadecyloxybenzoyloxyphenylethynyl)-thiophene-2-carboxylate | 155.38 | 139.70 | 140.50 | 14.88 |
| 1093 | Propyl 4-(4-(4-octyloxy-2,3,5,6-tetrafluorophenylethynyl)benzyloxy)benzoate | 115.25 | 100.60 | 100.30 | 14.95 |
| 1094 | Bis(4-(4-hexadecyloxyphenyliminomethyl)phenyl) heptanedioate | 213.32 | 229.40 | 228.30 | -14.98 |
| 1095 | 4-(2-Chloro-3-methylbutanoyloxy)biphenyl-4'-yl 4-octyloxy-2,3-difluorobenzoate | 72.47 | 89.50 | 87.50 | -15.03 |
| 1096 | 1,6-Bis(4'-propylazobenzene-4-oxy)hexane | 99.86 | 114.00 | 114.90 | -15.04 |
| 1097 | 2-Chloro-4-methylpentyl 4-(3-(4-decyloxyphenyl)-1-oxo-2-propenyloxy)benzoate | 113.35 | 96.40 | 98.30 | 15.05 |
| 1098 | Cholesteryl crotonate | 66.95 | 46.20 | 51.90 | 15.05 |
| 1099 | 4-((4-Propylphenylmethylene)amino)benzonitrile | 59.65 | 74.80 | 74.70 | -15.05 |
| 1100 | 4-Propanoyl-4'-hexadecanoyloxyazobenzene | 160.97 | 145.30 | 145.90 | 15.07 |
| 1101 | 4-Thiocyanophenyl 4-(4-pentyloxybenzoyloxy)benzoate | 78.63 | 89.90 | 93.70 | -15.07 |
| 1102 | N-(2-Hydroxy-4-butoxybenzylidene)-4'-dodecylphenylaniline | 125.30 | 110.40 | 110.20 | 15.10 |
| 1103 | 4-((4-Methoxyphenylmethylene)amino)phenol acetate | 67.60 | 80.80 | 82.70 | -15.10 |
| 1104 | N-(4-Undecyloxybenzylidene)-4-hexadecylaniline | 153.60 | 169.50 | 168.70 | -15.10 |
| 1105 | Cholesteryl 6-(4-(4-heptylphenylethynyl)phenoxy)hexanoate | 81.68 | 97.30 | 96.80 | -15.12 |
| 1106 | 4-(4-Nonyloxyphenylazoxy)phenyl 2-chloro-3-methylpentanoate | 117.23 | 99.10 | 102.10 | 15.13 |
| 1107 | 5-(4'-Heptyloxybiphenyl-4-yl)-2-(4-methylphenyl)pyridine | 40.36 | 56.50 | 55.50 | -15.14 |
| 1108 | 4'-(4-Pentylcyclohexyl)-3-fluoro-4-isothiocyanatotolane | 66.43 | 82.60 | 81.60 | -15.17 |
| 1109 | 4-(2-Methylbutoxycarbonyl)phenyl 4-(4-propoxy-2,3,4,5-tetrafluorophenylethynyl)benzoate | 67.70 | 81.60 | 82.90 | -15.20 |
| 1110 | 1-(4'-Propylazobenzene-4-oxy)-6-(4'-ethylazobenzene-4-oxy)hexane | 95.69 | 110.00 | 110.90 | -15.21 |
| 1111 | Cholesteryl decanoate | 85.25 | 99.20 | 100.50 | -15.25 |
| 1112 | 4-(4-Methoxyphenylmethylene)aminophenyl butanoate | 73.54 | 88.90 | 88.80 | -15.26 |
| 1113 | 2-(5-(4-(4-Propylcyclohexyl)phenyl)-1,2,4-oxadiazol-3-yl)pyridine | 59.39 | 45.30 | 44.10 | 15.29 |
| 1114 | 4-(3-Pyridiniminomethyl)phenyl 4-hexyloxybenzoate | 93.90 | 110.00 | 109.20 | -15.30 |
| 1115 | Cholesteryl thioundecanoate | 78.40 | 92.70 | 93.70 | -15.30 |
| 1116 | 4-(1-Methylheptyloxycarbonyl)-4'-(4-tetradecyloxy-3-fluorobenzoyloxy)tolane | 114.89 | 129.10 | 130.20 | -15.31 |
| 1117 | 2-Decyloxy-6-(4-decyloxyphenyl)anthracene | 99.87 | 114.40 | 115.20 | -15.33 |
| 1118 | 4-(4-(1-Methylheptyloxycarbonyl)phenyloxycarbonyl)phenyl 4-(5-(4-(4-decyloxybenzoyloxy)benzoyloxy)pentyloxy)benzoate | 168.04 | 151.40 | 152.70 | 15.34 |
| 1119 | N-(4-Ethoxybenzylidene)-4-hexadecylaniline | 147.84 | 133.20 | 132.50 | 15.34 |
| 1120 | 2,5-Bis(3,4,5-tris(dodecyloxyphenyl))-1,3,4-oxadiazole | 137.13 | 146.30 | 152.50 | -15.37 |
| 1121 | 3,5-Di(4-decyloxyphenyl)pyrazole | 136.71 | 145.50 | 152.10 | -15.39 |
| 1122 | 4-Cyanophenyl 4-(3-(4-(4'-hexadecylbiphenyl-4-carbonyloxy)-2-fluorobenzoyloxy)benzoyloxy)benzoate | 125.87 | 141.30 | 141.30 | -15.43 |
| 1123 | 4-(3,4-Bis(4-octyloxybenzoyloxy)benzylideneamino)azobenzene | 158.24 | 142.60 | 142.80 | 15.44 |
| 1124 | Bis(4-(5-hexylthio-1,3,4-oxadiazole-2-yl)phenyl)-2,5-thiophenedicarboxylate | 70.95 | 85.10 | 86.40 | -15.45 |
| 1125 | 4-Cyanophenyl 4-(3-(4-(4'-decylbiphenyl-4-carbonyloxy)benzoyloxy)benzoyloxy)benzoate | 130.26 | 116.00 | 114.80 | 15.46 |
| 1126 | 1-Methylheptyl 4'-(4''-decyloxy-3''-fluorobenzoyloxy)tolan-4-carboxylate | 96.73 | 112.90 | 112.20 | -15.47 |
| 1127 | 1-(4-Dodecylbiphenyl-4'-yl)-3-(4-hexyloxyphenyl)propane-1,3-dione | 146.18 | 129.20 | 130.70 | 15.48 |
| 1128 | 1-(6-Hexyloxy-2-naphthyl)-4-(3,4-difluorophenyl)diacetylene | 59.40 | 72.80 | 74.90 | -15.50 |
| 1129 | 4-Chloro-2'-hydroxy-4'-nonyloxyazobenzene | 81.28 | 95.90 | 96.80 | -15.52 |
| 1130 | 1-(2-(5-Chlorothienyl))-3-(4-heptyloxyphenylamino)-2-propen-1-one | 92.33 | 78.40 | 76.80 | 15.53 |
| 1131 | 6-Decyloxy-2-(4-octyloxyphenylazo)benzothiazole | 137.26 | 122.80 | 121.70 | 15.56 |
| 1132 | 2-(4-Cyanophenyl)-7-hexylfluorene | 48.61 | 58.90 | 64.20 | -15.59 |
| 1133 | Cholesteryl thioheptanoate | 85.60 | 69.20 | 70.00 | 15.60 |
| 1134 | 3-(4-(4-Heptylcyclohexyl)phenyl)-5-(5-dodecyl-2-thienyl)isoxazole | 96.67 | 110.90 | 112.30 | -15.63 |
| 1135 | 1,6-Bis(4-(4-propoxybenzoyloxybenzylideneamino)hexane | 140.86 | 125.10 | 125.20 | 15.66 |
| 1136 | 4-Heptyl-N-(4-octadecyloxyphenylmethylene)benzenamine | 144.34 | 161.40 | 160.00 | -15.66 |
| 1137 | Bis(4-(5-heptylthio-1,3,4-oxadiazole-2-yl)phenyl)-2,5-thiophenedicarboxylate | 76.63 | 93.20 | 92.30 | -15.67 |
| 1138 | 4-(4-Butoxyphenyl)acetylene-2,3,5,6-tetrafluorophenyl 4'-chlorobenzoate | 64.12 | 78.60 | 79.80 | -15.68 |
| 1139 | 4-(4-Ethoxyphenylazo)-3-methylphenyl 4-octyloxybenzoate | 118.42 | 103.30 | 102.70 | 15.72 |
| 1140 | 4,4'-Diethoxyazoxybenzene | 66.67 | 78.50 | 82.40 | -15.73 |
| 1141 | 4-(2-Propenyloxy)biphen-4'-yl 5-methoxy-2-thiophenecarboxylate | 198.07 | 210.90 | 213.80 | -15.73 |
| 1142 | Bis(4-hexyloxycarbonylphenyl) terephthalate | 109.26 | 123.70 | 125.00 | -15.74 |
| 1143 | 4,4'-Bis(4-(4-octyloxybenzylideneamino)benzoyloxy)diphenylmethane | 153.15 | 137.30 | 137.40 | 15.75 |
| 1144 | 4-Isopropylphenyl 4-(4-ethoxybenzylideneamino)benzoate | 89.69 | 73.30 | 73.90 | 15.79 |
| 1145 | 2,5,6,9,12,13-Hexabutoxydibenzo[fg.op]naphthacene | 71.50 | 57.30 | 55.70 | 15.80 |
| 1146 | 6-O-Dodecanoyl-alpha-D-galactopyranose | 71.90 | 83.80 | 87.70 | -15.80 |
| 1147 | 1-(2-(5-Cyanothienyl))-3-(4-octyloxyphenylamino)-2-propen-1-one | 50.30 | 66.30 | 66.10 | -15.80 |
| 1148 | 2-Methylpentyl 4-(4-(4-acetoxybenzoyloxy)benzylideneamino)benzoate | 78.68 | 95.00 | 94.50 | -15.82 |
| 1149 | 4,4'-Bis(4-(4-hexyloxybenzoyloxy)benzylideneamino)diphenylamine | 86.78 | 104.30 | 102.60 | -15.82 |
| 1150 | 1-(4-Butylphenyl)-4-(4-fluorophenyl)-1,3-butadiyne | 55.64 | 72.60 | 71.50 | -15.86 |
| 1151 | 1-Methylheptyl 4'-(4''-hexadecyloxy- 2'',3''-difluorobenzoyloxy)tolan-4-carboxylate | 155.17 | 138.20 | 139.30 | 15.87 |
| 1152 | 4,4'-Didecyloxyazoxybenzene | 123.62 | 143.20 | 139.50 | -15.88 |
| 1153 | 4,4'-Bis(4-(4-heptyloxybenzylideneamino)benzoyloxy)diphenylmethane | 112.66 | 129.20 | 128.60 | -15.94 |
| 1154 | Bis(4-ethoxyphenyl) 2-(5-(4-(4-biphenylazo)phenoxy)pentyloxy) terephthalate | 119.14 | 103.10 | 103.20 | 15.94 |
| 1155 | 4-(2-Chloro-3-methylpentanoyloxy)biphenyl-4'-yl 4-nonyloxy-2,3-difluorobenzoate | 112.40 | 97.60 | 96.40 | 16.00 |
| 1156 | 1-(4-Ethylcyclohexylethynyl)-4-(4-butylphenylethynyl)benzene | 43.88 | 57.60 | 59.90 | -16.02 |
| 1157 | Cholesteryl 8-(4-(4-pentylphenylethynyl)phenoxy)octanoate | 112.83 | 97.30 | 96.80 | 16.03 |
| 1158 | 4-Butoxyphenyl 4'-pentylbenzoate | 83.27 | 99.80 | 99.30 | -16.03 |
| 1159 | 4-(4-Ethoxyphenyl)azophenyl 2-butenoate | 65.72 | 81.20 | 81.80 | -16.08 |
| 1160 | 1-(4-(4-Formyl)phenylazophenyl)-4-(1-oxohexyl)piperazine | 48.10 | 36.00 | 32.00 | 16.10 |
| 1161 | 1-(4-Dodecylbiphenyl-4'-yl)-3-(2-propoxyphenyl)propane-1,3-dione | 133.68 | 117.10 | 117.50 | 16.18 |
| 1162 | 1-(4'-Pentylbicyclohexyl)-3,4,5-trifluorobenzene | 89.29 | 71.20 | 73.10 | 16.19 |
| 1163 | N,N',N''-Triheptanoyl-2,4,6-trimethyl-1,3,5-benzenetriamine | 78.20 | 64.00 | 62.00 | 16.20 |
| 1164 | 3,4,5-Trioctyloxybenzaldehyde-2',4'-dinitrophenyl hydrazone | 88.48 | 101.70 | 104.70 | -16.22 |
| 1165 | Decyl 2,5-bis(4-octyloxybenzoyloxy)benzoate | 184.33 | 167.10 | 168.10 | 16.23 |
| 1166 | 1-(4-Butylcyclohexylethynyl)-4-(4-propylphenylethynyl)benzene | 47.66 | 61.70 | 63.90 | -16.24 |
| 1167 | 1,1,2-Trimethylpropyl 4-(4-(4-methylbenzoyloxy)benzylideneamino)benzoate | 115.25 | 98.70 | 99.00 | 16.25 |
| 1168 | 1-(4-Propylcyclohexylethynyl)-4-(4-propylcyclohexylethynyl)benzene | 34.72 | 53.00 | 51.00 | -16.28 |
| 1169 | N-(4-Nonyloxybenzylidene)-4-ethylaniline | 121.69 | 104.90 | 105.40 | 16.29 |
| 1170 | 4,4'-Dinonyloxyazoxybenzene | 118.19 | 135.10 | 134.50 | -16.31 |
| 1171 | 3-Pyridyl 4-(4-propoxybenzylideneamino)benzoate | 81.96 | 97.90 | 98.30 | -16.34 |
| 1172 | 1-Methylheptyl 4'-(4''-tetradecyloxy- 2'',3''-difluorobenzoyloxy)tolan-4-carboxylate | 146.95 | 130.10 | 130.60 | 16.35 |
| 1173 | Bis(4-(4-decyloxyphenyliminomethyl)phenyl) decanedioate | 176.95 | 193.10 | 193.30 | -16.35 |
| 1174 | 1,2,12,13-Tetrahydroxytridecane | 154.96 | 140.00 | 138.60 | 16.36 |
| 1175 | 4-Pentanoyl-4'-heptadecanoyloxyazobenzene | 173.17 | 157.50 | 156.80 | 16.37 |
| 1176 | 1-Methylheptyl 4-(2-fluoro-4-(4-tridecyloxybenzoyloxy)phenylethynyl)benzoate | 141.37 | 125.00 | 125.00 | 16.37 |
| 1177 | 1,3-Phenylene bis(4-(4'-dodecylbiphenyl-4-carbonyloxy)-2-fluorobenzoate) | 180.48 | 165.50 | 164.10 | 16.38 |
| 1178 | 4-(4-Propylcyclohexyl)-4'-nitrotolane | 65.99 | 52.70 | 49.60 | 16.39 |
| 1179 | 4-(2,2,3,3,4,4,4-Heptafluorobutyloxycarbonyl)phenyl 4-(4-perfluorobutyl)pentyloxy)benzoate | 107.61 | 95.70 | 91.20 | 16.41 |
| 1180 | N-(2-Hydroxy-4-pentyloxybenzylidene)-4'-dodecylphenylaniline | 130.61 | 114.50 | 114.20 | 16.41 |
| 1181 | 1-(4'-Pentylazobenzene-4-oxy)-6-(azobenzene-4-oxy)hexane | 138.42 | 121.60 | 122.00 | 16.42 |
| 1182 | 7-(4'-Hexyloxybenzoyloxy)isoflavone | 55.08 | 70.80 | 71.50 | -16.42 |
| 1183 | 1-Methylheptyl 4-(3-chloro-4-hexadecyloxybenzoyloxy)biphenyl-4'-carboxylate | 149.66 | 132.70 | 133.20 | 16.46 |
| 1184 | 1-(6-Propoxy-2-naphthyl)-4-(3,4-difluorophenyl)diacetylene | 77.37 | 60.70 | 60.90 | 16.47 |
| 1185 | 4-Nonyloxy-4'-cyanobiphenyl | 109.37 | 92.30 | 92.90 | 16.47 |
| 1186 | 1,10-Bis(4-(4-octyloxybenzoyloxybenzylideneamino)decane | 164.83 | 181.70 | 181.30 | -16.47 |
| 1187 | 2-Fluoro-4-hexyloxyphenyl 4-hexyloxythiobenzoate | 119.98 | 103.50 | 103.50 | 16.48 |
| 1188 | Phenyl 4-((4-cyanophenylmethylene)amino)benzoate | 61.79 | 79.60 | 78.30 | -16.51 |
| 1189 | N,N'-Dioctanoyl-2,3,5,6-tetrakis(octanoyloxy)-1,4-benzenediamine | 189.38 | 202.80 | 205.90 | -16.52 |
| 1190 | 7-(4'-Nonyloxybenzoyloxy)isoflavone | 67.57 | 82.90 | 84.10 | -16.53 |
| 1191 | 1,4-Bis(4-(4-butoxybenzoyloxy)benzylideneamino)butane | 142.94 | 125.10 | 126.40 | 16.54 |
| 1192 | 5-(4-Pyridyl)-2-(4-octylphenylamido)-1,3,4-thiadiazole | 72.75 | 58.10 | 56.20 | 16.55 |
| 1193 | 4-(4-Propylcyclohexylethynyl)-4'-ethyltolane | 36.65 | 53.60 | 53.20 | -16.55 |
| 1194 | N,N'-Dinonanoyl-2,5,6-trimethyl-4-nonanoyloxy-1,3-benzenediamine | 91.05 | 109.50 | 107.60 | -16.55 |
| 1195 | 5-Methyl-5'-(2-(4-decyloxyphenyl)-2-hydroxyethyl)-2,2'-bipyridine | 80.37 | 62.20 | 63.80 | 16.57 |
| 1196 | 4-Chloro-1,3-phenylene bis(4-(4-methoxybenzylideneamino)benzoate) | 94.28 | 77.90 | 77.70 | 16.58 |
| 1197 | 4'-Octadecyloxy-3'-nitrobiphenyl-4-carboxylic acid | 158.39 | 140.20 | 141.80 | 16.59 |
| 1198 | 4,5-Dichloro-1,3-phenylene bis(4-(4-hexyloxybenzylideneamino)benzoate) | 123.70 | 108.30 | 107.10 | 16.60 |
| 1199 | 1-(4-Dodecylbiphenyl-4'-yl)-3-(3-bromophenyl)propane-1,3-dione | 131.61 | 114.60 | 115.00 | 16.61 |
| 1200 | 2-Ethylbutyl 4-(4-(4-methoxybenzoyloxy)benzylideneamino)benzoate | 71.28 | 86.60 | 87.90 | -16.62 |
| 1201 | 4-Pentylphenyl 4'-butyloxythiobenzoate | 71.78 | 89.20 | 88.40 | -16.62 |
| 1202 | 2-(5-(4-Butoxyphenyl)-1,3,4-thiadiazol-2-yliminomethyl)-5-(octyloxy)phenol | 81.74 | 65.80 | 65.10 | 16.64 |
| 1203 | Bis(4-pentyloxyphenyl) 2,2'-bithiophene-5,5'-dicarboxylate | 101.16 | 111.60 | 117.80 | -16.64 |
| 1204 | 4-(3,4-Bis(4-undecyloxybenzoyloxy)benzylideneamino)azobenzene | 184.27 | 166.80 | 167.60 | 16.67 |
| 1205 | 1,2-Dimethylpropyl 4-(4-(4-nitrobenzoyloxy)benzylideneamino)benzoate | 77.17 | 60.70 | 60.50 | 16.67 |
| 1206 | Bis(4-(4-hexadecyloxyphenyliminomethyl)phenyl) hexanedioate | 208.33 | 225.40 | 225.00 | -16.67 |
| 1207 | 4'-(2-(4-Hexylphenyl)-1-ethynyl)-2'-methyl-4-ethyltolane | 45.60 | 62.80 | 62.30 | -16.70 |
| 1208 | Cholesteryl thiodecanoate | 73.10 | 88.70 | 89.80 | -16.70 |
| 1209 | 4-Thiocyanophenyl 4-(4-ethoxybenzoyloxy)benzoate | 93.62 | 77.70 | 76.90 | 16.72 |
| 1210 | 4-Methoxyphenyl 3-fluoro-4-thiocyanatophenyl terephthalate | 91.42 | 76.10 | 74.70 | 16.72 |
| 1211 | 3-(4-(4-Heptylcyclohexyl)phenyl)-5-(5-heptyl-2-thienyl)isoxazole | 75.98 | 90.70 | 92.70 | -16.72 |
| 1212 | 4-(3,3,4,4,5,5,6,6,7,7,8,8,8-Tridecafluorooctyloxycarbonyl)phenyl 4-(perfluorobutylpentyloxy)benzoate | 96.88 | 113.80 | 113.60 | -16.72 |
| 1213 | 1-Methylheptyl 4'-(3-chloro-4-octadecyloxybenzoyloxy)biphenyl-4-carboxylate | 157.45 | 140.80 | 140.70 | 16.75 |
| 1214 | 1,3-Phenylene bis(4-(4'-hexylbiphenyl-4-carbonyloxy)-2-fluorobenzoate) | 135.75 | 117.00 | 119.00 | 16.75 |
| 1215 | 1,3-Phenylene bis(4-(4'-tetradecylbiphenyl-4-carbonyloxy)-3-fluorobenzoate) | 166.15 | 181.70 | 182.90 | -16.75 |
| 1216 | N-(4-Methoxybenzylidene)-4-n-butylaniline | 62.93 | 82.00 | 79.70 | -16.77 |
| 1217 | 4-(Phenyliminomethyl)phenyl 4-(phenyliminomethyl)benzoate | 72.39 | 90.00 | 89.20 | -16.81 |
| 1218 | 4-Octyloxy-3'-stilbazole N-oxide | 70.97 | 84.90 | 87.80 | -16.83 |
| 1219 | 3-Pyridyl 4-(4-ethoxybenzylideneamino)benzoate | 76.24 | 93.80 | 93.10 | -16.86 |
| 1220 | 4-(1-Methylheptyloxycarbonyl)phenyl 4-(4-dodecyloxyphenylethyl)benzoate | 149.17 | 131.90 | 132.30 | 16.87 |
| 1221 | 1,8-Bis(4-ethoxyazobenzene-4'-carbonyloxy)octane | 136.48 | 121.80 | 119.60 | 16.88 |
| 1222 | 4-Heptadecyloxybenzoic acid | 166.89 | 150.10 | 150.00 | 16.89 |
| 1223 | 4-(4-Hexadecyloxyphenylazoxy)phenyl 2-chloro-3-methylpentanoate | 113.16 | 127.40 | 130.10 | -16.94 |
| 1224 | 1-(4-Heptanoyl)phenyl-4-heptylcyclohexane | 84.13 | 102.00 | 101.10 | -16.97 |
| 1225 | 5-(4-Pyridyl)-2-(4-nonyloxybenzylideneamino)-1,3,4-thiadiazole | 65.33 | 81.60 | 82.30 | -16.97 |
| 1226 | 4-Isothiocyanatophenyl 4-(4-decylcyclohexyl)benzoate | 133.59 | 116.50 | 116.60 | 16.99 |
| 1227 | 4'-Decyloxybiphenyl-4-yl 4-(2-dodecyloxy-1-oxopropoxy)benzoate | 121.98 | 140.60 | 139.00 | -17.02 |
| 1228 | 1-(4'-Butylazobenzene-4-oxy)-6-(azobenzene-4-oxy)hexane | 135.52 | 117.60 | 118.50 | 17.02 |
| 1229 | 4-(4-Nonyloxy-2,3,5,6-tetrafluorophenylacetylenyl)phenyl 4'-chlorobenzoate | 82.47 | 98.80 | 99.50 | -17.03 |
| 1230 | 4-Decyloxyphenyl 7-decanoyloxychromone-2-carboxylate | 72.06 | 87.40 | 89.10 | -17.04 |
| 1231 | 1-(4-(3-Buten-1-yl)phenyl)-4-(4-ethylphenyl)-1,3-butadiyne | 53.20 | 70.50 | 70.30 | -17.10 |
| 1232 | Cholesteryl thioeicosanoate | 145.20 | 129.10 | 128.10 | 17.10 |
| 1233 | Cholesteryl thiononanoate | 68.30 | 84.60 | 85.40 | -17.10 |
| 1234 | 2-Hydroxyethyl 4-((4'-octyloxybiphenyl-4-yl)oxymethyl)benzoate | 94.89 | 111.90 | 112.00 | -17.11 |
| 1235 | 2,5-Bis(3,4,5-tris(hexadecyloxyphenyl))-1,3,4-oxadiazole | 256.32 | 243.20 | 239.20 | 17.12 |
| 1236 | 2,3,4-Trichloro-N-(4-(4-methylbenzoyloxy)benzylidene)aniline | 74.54 | 58.50 | 57.40 | 17.14 |
| 1237 | 4-Propanoyl-4'-pentadecanoyloxyazobenzene | 157.24 | 141.30 | 140.10 | 17.14 |
| 1238 | N,N'-Didodecanoyl-2,3,5,6-tetrakis(dodecanoyloxy)-1,4-benzenediamine | 285.94 | 299.80 | 303.10 | -17.16 |
| 1239 | Cholesteryl thioheptadecanoate | 132.90 | 116.90 | 115.70 | 17.20 |
| 1240 | Bis(4-(4-decyloxybenzylideneamino)phenyl) hexanedioate | 161.60 | 176.90 | 178.80 | -17.20 |
| 1241 | 4-(4-(4-(4-Undecyloxybenzoyloxy)benzoyl)-1-piperazinyl)phenyl 4-(4-undecyloxybenzoyloxy)benzoate | 80.19 | 97.10 | 97.40 | -17.21 |
| 1242 | 4'-(4-Butylcyclohexyl)-3-fluoro-4-isothiocyanatotolane | 61.59 | 78.50 | 78.80 | -17.21 |
| 1243 | 4-Dodecanoyloxy-2,3-dimethyl-4'-ethoxyazobenzene | 130.32 | 112.30 | 113.10 | 17.22 |
| 1244 | 4-(4,4,4-Trifluorobut-2-enyloxy)-4'-(4-pentylcyclohexyl)biphenyl | 69.15 | 50.90 | 51.90 | 17.25 |
| 1245 | 1-Methylheptyl 5-(4-nonyloxybenzoyloxyphenylethynyl)-thiophene-2-carboxylate | 128.56 | 111.40 | 111.30 | 17.26 |
| 1246 | 4-(4'-Undecylcyclohexyl)isothiocyanatobenzene | 133.46 | 115.30 | 116.20 | 17.26 |
| 1247 | 4'-Undecyloxybiphenyl-4-yl 2-(1-methylpropyl)-1-oxo-3-thioxoisoindole-5-carboxylate | 71.82 | 84.60 | 89.10 | -17.28 |
| 1248 | 4-(4'-Pentylbicyclohexyl)benzene-isothiocyanate | 65.02 | 80.70 | 82.30 | -17.28 |
| 1249 | 1-(4'-Hexylazobenzene-4-oxy)-6-(4'-propylazobenzene-4-oxy)hexane | 110.92 | 126.10 | 128.20 | -17.28 |
| 1250 | 6-Pentyloxy-2-(4-butoxystyryl)quinoline | 67.50 | 85.60 | 84.80 | -17.30 |
| 1251 | Cholesteryl 5-(4-(4-pentylphenylethynyl)phenoxy)pentanoate | 68.17 | 85.20 | 85.50 | -17.33 |
| 1252 | 1-(4'-Methylazobenzene-4-oxy)-6-(azobenzene-4-oxy)hexane | 122.22 | 105.50 | 104.80 | 17.42 |
| 1253 | 4,6-Dichloro-1,3-phenylene bis(4-(4-octyloxy-3-chlorophenyliminomethyl)benzoate) | 127.13 | 110.00 | 109.70 | 17.43 |
| 1254 | 2-Methylpropyl 4-(4-(4-cyanobenzoyloxy)benzylideneamino)benzoate | 92.87 | 75.40 | 75.40 | 17.47 |
| 1255 | 3,4,5-Trihexadecyloxybenzaldehyde-2',4'-dinitrophenyl hydrazone | 184.72 | 198.70 | 202.20 | -17.48 |
| 1256 | 6-O-Octadecyl-alpha-D-galactopyranose | 139.69 | 119.60 | 122.20 | 17.49 |
| 1257 | 4-Cyanophenyl 4-(3-(4-(4'-hexadecylbiphenyl-4-carbonyloxy)-3-fluorobenzoyloxy)benzoyloxy)benzoate | 125.00 | 141.30 | 142.50 | -17.50 |
| 1258 | Methyl 5-((4-(4-decyloxybenzoyloxy)phenyloxycarbonyl)-1,3-dioxoisoindole-2-acetate | 87.20 | 108.30 | 104.70 | -17.50 |
| 1259 | 1-(6-Pentyloxy-2-naphthyl)-4-(3,4-difluorophenyl)diacetylene | 86.41 | 68.70 | 68.90 | 17.51 |
| 1260 | 4-Pentyloxy-4'-cyanobiphenyl | 93.52 | 76.20 | 76.00 | 17.52 |
| 1261 | t-Butyl 4-(4-(4-methoxybenzoyloxy)benzylideneamino)benzoate | 122.14 | 105.80 | 104.60 | 17.54 |
| 1262 | 1-(4'-Propylbicyclohexyl)-2,3,4-trifluorobenzene | 79.74 | 63.10 | 62.20 | 17.54 |
| 1263 | 6-O-Hexadecyl-D-galacitol | 167.14 | 148.20 | 149.60 | 17.54 |
| 1264 | 2-Hydroxypropyl 4-((4'-octyloxybiphenyl-4-yl)oxymethyl)benzoate | 115.05 | 97.30 | 97.50 | 17.55 |
| 1265 | N,N'-Didecanoyl-2,5,6-trimethyl-4-decanoyloxy-1,3-benzenediamine | 104.84 | 121.70 | 122.40 | -17.56 |
| 1266 | Octakis(nonanoyloxy)-9,10-anthraquinone | 294.53 | 306.80 | 312.10 | -17.57 |
| 1267 | 1-(4-Dodecylbiphenyl-4'-yl)-3-(3-hexyloxyphenyl)propane-1,3-dione | 146.18 | 129.20 | 128.60 | 17.58 |
| 1268 | 1-(4-Dodecylbiphenyl-4'-yl)-3-(2-methoxyphenyl)propane-1,3-dione | 63.40 | 45.80 | 45.80 | 17.60 |
| 1269 | 2-(4-Cyanophenyl)-7-pentylfluorene | 37.07 | 54.80 | 54.70 | -17.63 |
| 1270 | 6-Decyloxy-2-(4'-butoxyphenyliminomethyl)quinoline | 101.67 | 119.60 | 119.30 | -17.63 |
| 1271 | 1-(2-(5-Chlorothienyl))-3-(4-undecyloxyphenylamino)-2-propen-1-one | 110.04 | 94.60 | 92.40 | 17.64 |
| 1272 | 4-Pentyl-4'-cyanobiphenyl | 57.16 | 75.00 | 74.80 | -17.64 |
| 1273 | 4-Pentanoyl-4'-butanoyloxyazobenzene | 86.24 | 104.90 | 103.90 | -17.66 |
| 1274 | 4-Cyano-4'-undecyloxybiphenyl | 117.71 | 100.40 | 100.00 | 17.71 |
| 1275 | 4-Cyanophenyl 4-(3-(4-(4'-undecylbiphenyl-4-carbonyloxy)benzoyloxy)benzoyloxy)benzoate | 103.49 | 120.10 | 121.20 | -17.71 |
| 1276 | 3,4,5-Tris(heptyloxy)benzamide | 67.52 | 52.40 | 49.80 | 17.72 |
| 1277 | Cholesteryl anisoate | 80.32 | 63.70 | 62.60 | 17.72 |
| 1278 | 6-O-Nonanoyl-alpha-D-galactopyranose | 91.47 | 107.40 | 109.20 | -17.73 |
| 1279 | m-Terphenyl-4,4''-diyl bis(4-(4-octylbenzoyloxy)benzoate) | 149.85 | 131.20 | 132.10 | 17.75 |
| 1280 | 4-Methylpentyl 4-(4-(4-methoxybenzoyloxy)benzylideneamino)benzoate | 104.26 | 86.60 | 86.50 | 17.76 |
| 1281 | 2-(4-Butoxyphenyl)-5-(4-(2-ethyl-2,3-undecadienyloxy)phenyl-1,3,4-thiadiazole | 105.22 | 89.60 | 87.40 | 17.82 |
| 1282 | 4-Octyloxy-4''-pentylterphenyl | 81.58 | 99.40 | 99.40 | -17.82 |
| 1283 | 4-Heptylcyclohexyl 4-propoxycinnamate | 97.93 | 80.10 | 80.10 | 17.83 |
| 1284 | 4-(2-Methylbutoxycarbonyl)phenyl 4-(4-pentyloxy-2,3,4,5-tetrafluorophenylethynyl)benzoate | 73.77 | 89.70 | 91.60 | -17.83 |
| 1285 | 4-Cyanophenyl 4-(3-(4-(4'-octadecylbiphenyl-4-carbonyloxy)benzoyloxy)benzoyloxy)benzoate | 167.04 | 148.30 | 149.20 | 17.84 |
| 1286 | 1,10-Dibutyl(permethyl)decasilane | 133.09 | 115.20 |  | 17.89 |
| 1287 | 4'-(2-(4-Propylphenyl)-1-ethynyl)-2'-methyl-4-pentyltolane | 45.19 | 62.80 | 63.10 | -17.91 |
| 1288 | 4-Butoxy-4'-(4-pentylcyclohexyl)biphenyl | 95.32 | 78.50 | 77.40 | 17.92 |
| 1289 | N-(4-Methoxybenzylidene)-4-heptylaniline | 111.23 | 94.20 | 93.30 | 17.93 |
| 1290 | Dipentyl N,N'-(1,4-phenylene-bis(methylidene))-bis(aminobenzoate) | 94.75 | 109.70 | 112.70 | -17.95 |
| 1291 | 2-Methylpropyl 4-(4-(4-methoxybenzoyloxy)benzylideneamino)benzoate | 95.37 | 78.50 | 77.40 | 17.97 |
| 1292 | 2-Methylbutyl 4-(4-(4-acetoxybenzoyloxy)benzylideneamino)benzoate | 74.21 | 90.90 | 92.20 | -17.99 |
| 1293 | 1-(4-Undecyloxybiphenyl-4'-yl)-3-(4-cyanobiphenyl)propane-1,3-dione | 80.61 | 98.70 | 98.60 | -17.99 |
| 1294 | Propyl 4-(4-(4-methoxybenzoyloxy)benzylideneamino)benzoate | 105.90 | 87.10 | 87.90 | 18.00 |
| 1295 | 3,4-Dihexdecyloxybenzaldehyde-2',4'-dinitrophenyl hydrazone | 159.22 | 144.50 | 141.20 | 18.02 |
| 1296 | Bis(6-(5-(4-butyoxyphenyliminomethyl)phenoxy)pentyl) malonate | 166.28 | 145.10 | 148.20 | 18.08 |
| 1297 | 1,8-Bis(4-(4-pentyloxybenzoyloxybenzylideneamino)octane | 165.93 | 149.40 | 147.80 | 18.13 |
| 1298 | 4-(2,2,3,3,4,4,4-Heptafluorobutyloxycarbonyl)phenyl 4-(heptyloxy)benzoate | 81.96 | 99.40 | 100.10 | -18.14 |
| 1299 | 1-(2-(5-Chlorothienyl))-3-(4-decyloxyphenylamino)-2-propen-1-one | 106.85 | 90.60 | 88.70 | 18.15 |
| 1300 | 2-Chloro-3-methylpentyl 4-(3-(4-heptyloxyphenyl)-1-oxo-2-propenyloxy)benzoate | 102.86 | 84.30 | 84.70 | 18.16 |
| 1301 | 1-Methylheptyl 4-(4-(4-octyloxycinnamoyloxy)benzoyloxy)benzoate | 129.30 | 112.60 | 111.10 | 18.20 |
| 1302 | 1-(2-(5-Nonylthienyl))-3-(4-bromophenylamino)-2-propen-1-one | 98.90 | 76.60 | 80.70 | 18.20 |
| 1303 | 3-Pyridyl 4-(4-octyloxybenzylideneamino)benzoate | 100.49 | 118.10 | 118.70 | -18.21 |
| 1304 | 1,3-Bis(4-(4-heptyloxybenzoyloxy)benzylideneamino)propane | 128.08 | 145.30 | 146.30 | -18.22 |
| 1305 | 1,3-Bis(4-(2-hydroxy-4-butoxybenzylideneamino)benzoyloxy)benzene | 105.77 | 88.60 | 87.50 | 18.27 |
| 1306 | 4'-(2-(4-Ethylphenyl)-1-ethynyl)-2'-methyl-4-ethyltolane | 64.18 | 46.60 | 45.90 | 18.28 |
| 1307 | Di(4'-methoxyphenyl)cyclohexane-1,4-dicarboxylate | 92.89 | 76.00 | 74.60 | 18.29 |
| 1308 | 4-(2-Methylbutoxy)phenyl 4-(4-butoxy-2,3,5,6-tetrafluorophenylethynyl)benzoate | 61.00 | 82.90 | 79.30 | -18.30 |
| 1309 | 4-Pentyl-4'-dodecyloxyazobenzene | 115.00 | 132.80 | 133.30 | -18.30 |
| 1310 | 2-Cyano-1,3-phenylene bis(4-(4'-octylbiphenyl-4-carbonyloxy)-3-fluorobenzoate) | 108.99 | 124.90 | 127.30 | -18.31 |
| 1311 | 4'-(2-(4-Hexylphenyl)-1-ethynyl)-2'-ethyl-4-propyltolane | 53.18 | 70.90 | 71.50 | -18.32 |
| 1312 | 1,4-Bis(4-(4-octyloxybenzoyloxybenzylideneamino)butane | 173.53 | 157.40 | 155.20 | 18.33 |
| 1313 | 4-(Biphenyl-4-yliminomethyl)phenyl benzoate | 100.25 | 82.10 | 81.90 | 18.35 |
| 1314 | 4-Chlorophenyl 4-pentyloxythiobenzoate | 95.06 | 77.40 | 76.70 | 18.36 |
| 1315 | 4-Pentadecyloxybenzoic acid | 161.89 | 142.10 | 143.50 | 18.39 |
| 1316 | 4-(4'-Heptylbicyclohexyl)benzene-isothiocyanate | 106.60 | 88.80 | 88.20 | 18.40 |
| 1317 | 1-Methylheptyl 4'-(4''-nonyloxy-3''-fluorobenzoyloxy)tolan-4-carboxylate | 89.89 | 108.90 | 108.30 | -18.41 |
| 1318 | 4-(4'-Octylcyclohexyl)isothiocyanatobenzene | 120.73 | 103.20 | 102.30 | 18.43 |
| 1319 | 1-(2-(5-Heptylthienyl))-3-(4-fluorophenylamino)-2-propen-1-one | 87.29 | 70.30 | 68.80 | 18.49 |
| 1320 | 2-(4-Butoxyphenyl)-5-(4-(2-butyl-2,3-undecadienyloxy)phenyl-1,3,4-thiadiazole | 114.30 | 97.70 | 95.80 | 18.50 |
| 1321 | 4-Methoxy-4'-heptyloxy-trans-stilbene | 101.10 | 81.50 | 82.60 | 18.50 |
| 1322 | 2,4-Dimethyl-N-(4-(4-methoxybenzoyloxy)benzylidene)aniline | 88.33 | 67.50 | 69.80 | 18.53 |
| 1323 | 4-Propyl-4'-pentyloxyazobenzene | 115.77 | 97.60 | 97.20 | 18.57 |
| 1324 | 4-Chlorobenzylidene-4'-hexylaniline | 69.93 | 87.90 | 88.50 | -18.57 |
| 1325 | 1-Methylpropyl 4-(4-(4-methoxybenzylideneamino)benzoyloxy)benzoate | 89.68 | 72.50 | 71.10 | 18.58 |
| 1326 | 4-(4-Octyloxybenzylideneamino)phenyl 4-ethylbenzoate | 87.50 | 106.10 | 106.10 | -18.60 |
| 1327 | 2-Butoxy-1-methyl-2-oxoethyl 4-(3-(6-octyloxy-2-naphthalenyl)-1-oxo-2-propenyloxy)benzoate | 112.31 | 93.10 | 93.70 | 18.61 |
| 1328 | 2,2-Dimethylpropyl 4-(4-(4-methoxybenzoyloxy)benzylideneamino)benzoate | 73.89 | 93.20 | 92.50 | -18.61 |
| 1329 | 4-Butoxy-3-fluoro-4'-isothiocyanatotolane | 72.49 | 90.10 | 91.10 | -18.61 |
| 1330 | 4-(3,4-Bis(4-hexyloxybenzoyloxy)benzylideneamino)azobenzene | 145.64 | 126.40 | 127.00 | 18.64 |
| 1331 | 5-(4-Heptyloxyphenyl)-N-(5-octyl-2-thienylmethylene)-1,3,4-thiadiazole-2-amine | 70.86 | 89.30 | 89.50 | -18.64 |
| 1332 | 1-(4'-Hexylazobenzene-4-oxy)-6-(4'-ethylazobenzene-4-oxy)hexane | 103.66 | 122.10 | 122.30 | -18.64 |
| 1333 | 3-Methylbutyl 4-(4-(4-methoxybenzylideneamino)benzoyloxy)benzoate | 102.35 | 82.60 | 83.70 | 18.65 |
| 1334 | 1-Methylpentyl 4-(4-(4-chlorobenzoyloxy)benzylideneamino)benzoate | 96.48 | 78.40 | 77.80 | 18.68 |
| 1335 | 6-Pentyloxy-2-(4-octyloxystyryl)quinoline | 83.92 | 101.70 | 102.60 | -18.68 |
| 1336 | 1,4-Bis(4-(4-pentyloxybenzoyloxy)benzylideneamino)butane | 154.50 | 133.20 | 135.80 | 18.70 |
| 1337 | 1,3-Phenylene bis(4-(4'-nonylbiphenyl-4-carbonyloxy)-2-fluorobenzoate) | 121.37 | 141.30 | 140.10 | -18.73 |
| 1338 | 4-Heptyl-4'-decyloxyazobenzene | 115.76 | 132.80 | 134.50 | -18.74 |
| 1339 | 1-(4-Nonylbiphenyl-4'-yl)-3-(4-chlorophenyl)propane-1,3-dione | 78.36 | 96.10 | 97.10 | -18.74 |
| 1340 | N,N'-Diheptanoyl-2,3,5-trimethylbenzene-1,4-diamine | 77.75 | 61.80 | 59.00 | 18.75 |
| 1341 | 5-(4-(Hexyloxy)phenyl)-N-(5-octyl-2-thienylmethylene)-1,3,4-thiadiazole-2-amine | 66.94 | 85.20 | 85.70 | -18.76 |
| 1342 | 1-(4-Butylcyclohexylethynyl)-4-(4-pentylcyclohexylethynyl)benzene | 45.64 | 65.10 | 64.40 | -18.76 |
| 1343 | 1-(4-Hexylaminophenyl)-4-(4-nitrophenyl)-1,3-butadiyne | 99.49 | 80.20 | 80.70 | 18.79 |
| 1344 | Di(4'-octylphenyl)-cyclohexane-1,4-dicarboxylate | 108.91 | 127.40 | 127.70 | -18.79 |
| 1345 | 4-(4-Ethoxyphenylazo)-3-methylphenyl 4-tetradecyloxybenzoate | 145.91 | 127.50 | 127.10 | 18.81 |
| 1346 | 5-(4'-Pentyloxybiphenyl-4-yl)-2-(4-methylphenyl)pyridine | 31.29 | 48.40 | 50.10 | -18.81 |
| 1347 | 2-(4-Cyanophenyl)-7-ethylfluorene | 24.49 | 42.70 | 43.30 | -18.81 |
| 1348 | 4-(2,2,3,3,4,4,4-Heptafluorobutyloxycarbonyl)phenyl 4-(undecyloxy)benzoate | 96.18 | 115.50 | 115.00 | -18.82 |
| 1349 | 4-Octanoyloxy-2-methyl-4'-ethoxyazobenzene | 124.35 | 103.80 | 105.50 | 18.85 |
| 1350 | 4-Chloro-2'-hydroxy-4'-undecyloxyazobenzene | 121.96 | 104.00 | 103.10 | 18.86 |
| 1351 | 4-Heptyl-4'-dodecyloxyazobenzene | 122.70 | 140.90 | 141.60 | -18.90 |
| 1352 | 4-Pentyloxy-4''-cyano-p-terphenyl | 52.20 | 70.50 | 71.10 | -18.90 |
| 1353 | Methyl 5-((4'-decyloxybiphenyl-4-yl)oxycarbonyl)-1,3-dioxoisoindole-2-butanonate | 117.61 | 105.50 | 98.70 | 18.91 |
| 1354 | 2,4-Dichloro-N-(4-(4-methylbenzoyloxy)benzylidene)aniline | 85.52 | 65.70 | 66.60 | 18.92 |
| 1355 | Butyl 4-(4-(4-octyloxy-2,3,5,6-tetrafluorophenylethynyl)benzyloxy)benzoate | 87.48 | 104.70 | 106.40 | -18.92 |
| 1356 | 4-(Benzoyloxy)phenyl biphenyl-4-carboxylate | 105.73 | 85.10 | 86.80 | 18.93 |
| 1357 | 2,5-Pentanediyl bis(4-(4-octyloxybiphenyl-4'-oxymethyl)benzoate) | 96.93 | 115.40 | 115.90 | -18.97 |
| 1358 | 4-Hexyloxy-4'-isothiocyanatotolane | 115.88 | 97.20 | 96.90 | 18.98 |
| 1359 | 1,4-Bis(4-ethoxyazobenzene-4'-carbonyloxy)butane | 122.46 | 105.70 | 103.40 | 19.06 |
| 1360 | 4-(2-(1-Ethylpropoxy)-1-methyl-2-oxoethoxy)phenyl (4'-nonyloxy)biphenyl-4-carboxylate | 72.60 | 90.10 | 91.70 | -19.10 |
| 1361 | 4-Decyloxycarbonylbenzoic acid | 105.28 | 124.60 | 124.40 | -19.12 |
| 1362 | Di(4'-butylphenyl)-cyclohexane-1,4-dicarboxylate | 76.28 | 95.10 | 95.40 | -19.12 |
| 1363 | 1,3-Phenylene bis(4-(4'-hexylbiphenyl-4-carbonyloxy)benzoate) | 136.28 | 115.00 | 117.10 | 19.18 |
| 1364 | 4-Pentanoyl-4'-decanoyloxyazobenzene | 110.48 | 129.20 | 129.70 | -19.22 |
| 1365 | 4-Octyl-4'-cyano-p-terphenyl | 62.47 | 81.40 | 81.70 | -19.23 |
| 1366 | 1,1-Dimethylpropyl 4-(4-(4-chlorobenzoyloxy)benzylideneamino)benzoate | 91.57 | 107.70 | 110.80 | -19.23 |
| 1367 | 4-(4-Ethoxyphenylazo)-2,3,6-trimethylphenyl 4-decyloxybenzoate | 115.75 | 96.10 | 96.50 | 19.25 |
| 1368 | 2,6,10-Tris(pentyloxy)-3,7,11-tris(hexyloxy)triphenylene | 103.23 | 120.60 | 122.50 | -19.27 |
| 1369 | 1-(4-Ethylcyclohexylethynyl)-4-(4-pentylcyclohexylethynyl)benzene | 38.73 | 57.00 | 58.00 | -19.27 |
| 1370 | 4-Decyl-4'-cyanobiphenyl | 113.90 | 95.20 | 94.60 | 19.30 |
| 1371 | 1-(4-Pentyloxyphenylethynyl)-3-methyl-4-(4-butylphenylethynyl)benzene | 48.99 | 68.00 | 68.30 | -19.31 |
| 1372 | 2,5-Dihydro-2,5-dimethyl-3,6-bis(4-octyloxyphenyl)pyrrolo(3,4-c)pyrrole-1,4-dione | 158.31 | 142.10 | 139.00 | 19.31 |
| 1373 | 1,7-Bis(4'-butylazobenzene-4-oxy)heptane | 106.97 | 126.10 | 126.30 | -19.33 |
| 1374 | 1,8-Bis(4-octyloxyazobenzene-4'-carbonyloxy)octane | 189.64 | 170.30 | 170.30 | 19.34 |
| 1375 | 1-(4-Propylcyclohexylethynyl)-4-(4-butylcyclohexylethynyl)benzene | 40.78 | 57.00 | 60.20 | -19.42 |
| 1376 | 4-Tetradecyl-N-(4-octadecyloxyphenylmethylene)benzenamine | 172.17 | 189.70 | 191.60 | -19.43 |
| 1377 | 1-(4-(N-Ethoxycarbonylpiperazinyl)phenyl)-3-(4-octyloxyphenyl)-1-propargyl-3-imine | 55.65 | 72.30 | 75.10 | -19.45 |
| 1378 | 4-n-Decyloxycinnamic acid | 133.07 | 115.90 | 113.60 | 19.47 |
| 1379 | 4-Heptanoyloxy-3-methyl-4'-ethoxyazobenzene | 118.67 | 99.70 | 99.20 | 19.47 |
| 1380 | 4-Cyanophenyl 4-(3-(4-(4'-tetradecylbiphenyl-4-carbonyloxy)-3-fluorobenzoyloxy)benzoyloxy)benzoate | 112.42 | 133.20 | 131.90 | -19.48 |
| 1381 | 1-Methylheptyl 4-(4-(4-decyloxy-3-fluorobenzoyloxy)-3-fluorobenzoyloxy)benzoate | 110.30 | 128.60 | 129.80 | -19.50 |
| 1382 | 2-Cyano-1,3-phenylene bis(4-(4'-tetradecylbiphenyl-4-carbonyloxy)-3-fluorobenzoate) | 154.87 | 173.40 | 174.40 | -19.53 |
| 1383 | 1,5-Bis(4-(2-hydroxy-4-pentyloxybenzylideneamino)benzoyloxy)pentane | 127.04 | 108.60 | 107.50 | 19.54 |
| 1384 | 4-(3,3,4,4,5,5,6,6,7,7,8,8,8-Tridecafluorooctyloxycarbonyl)phenyl 4-((perfluorohexyl)butoxy)benzoate | 138.39 | 119.10 | 118.80 | 19.59 |
| 1385 | 2-Methylhexyl 4-(4-(4-methoxybenzylideneamino)benzoyloxy)benzoate | 73.81 | 90.70 | 93.40 | -19.59 |
| 1386 | 2-(p-Terphenyl-4-yl)-5-phenylthiophene | 45.01 | 61.20 | 64.60 | -19.59 |
| 1387 | 4-(4-Undecyloxyphenylazoxy)phenyl 2-chloro-3-methylpentanoate | 123.51 | 107.20 | 103.90 | 19.61 |
| 1388 | Di(4'-butoxyphenyl)-cyclohexane-1,4-dicarboxylate | 79.55 | 97.40 | 99.20 | -19.65 |
| 1389 | 2-Decyloxy-6-(4-decyloxyphenylethynyl)naphthalene | 100.83 | 120.60 | 120.50 | -19.67 |
| 1390 | 6-Pentyloxy-2-(4-pentyloxystyryl)quinoline | 71.03 | 89.60 | 90.70 | -19.67 |
| 1391 | 4-(But-2-enyloxy)-4'-(4-pentylcyclohexyl)biphenyl | 83.10 | 64.50 | 63.40 | 19.70 |
| 1392 | 4-Butyl-4'-heptyloxyazobenzene | 88.80 | 108.60 | 108.50 | -19.70 |
| 1393 | 4-Heptyl-4'-octyloxyazobenzene | 105.25 | 124.70 | 125.00 | -19.75 |
| 1394 | 4-(3-Pyridiniminomethyl)phenyl 4-octyloxybenzoate | 98.95 | 118.10 | 118.70 | -19.75 |
| 1395 | 4-Propylcyclohexylbenzyl perfluorooctyl thioether | 95.47 | 80.90 | 75.70 | 19.77 |
| 1396 | 4,6-Dichloro-1,3-phenylene bis(4-(4-heptyloxyphenyliminomethyl)benzoate) | 95.53 | 116.40 | 115.30 | -19.77 |
| 1397 | 5-(4-Pyridyl)-2-(4-octyloxybenzylideneamino)-1,3,4-thiadiazole | 57.82 | 77.60 | 77.60 | -19.78 |
| 1398 | 3,5-Dihydroxycyclohexyl 3,4-bis(dodecyloxy)benzoate | 142.71 | 120.60 | 122.90 | 19.81 |
| 1399 | 4-Pentanoyl-4'-heptanoyloxyazobenzene | 97.56 | 117.00 | 117.40 | -19.84 |
| 1400 | 1-(4'-Propylazobenzene-4-oxy)-6-(4'-methylazobenzene-4-oxy)hexane | 125.46 | 105.90 | 105.60 | 19.86 |
| 1401 | 1-(2-(5-Hexylthienyl))-3-(4-bromophenylamino)-2-propen-1-one | 85.08 | 64.40 | 65.20 | 19.88 |
| 1402 | 2-Cyano-1,3-phenylene bis(4-(4'-undecylbiphenyl-4-carbonyloxy)-3-fluorobenzoate) | 130.62 | 149.20 | 150.50 | -19.88 |
| 1403 | 1,8-Bis(4-(4-butoxybenzoyloxybenzylideneamino)octane | 123.39 | 141.30 | 143.30 | -19.91 |
| 1404 | 1-(2-(5-Cyanothienyl))-3-(4-nonyloxyphenylamino)-2-propen-1-one | 50.85 | 70.30 | 70.80 | -19.95 |
| 1405 | 4-Nonyl-4'-cyanobiphenyl | 110.45 | 91.20 | 90.50 | 19.95 |
| 1406 | 4'-(2-(4-Propylphenyl)-1-ethynyl)-2'-methyl-4-hexyltolane | 47.36 | 66.80 | 67.40 | -20.04 |
| 1407 | Cholesteryl 3-phenylpropionate | 96.68 | 76.90 | 76.60 | 20.08 |
| 1408 | 4-Cyanophenyl 3-(4'-decyloxybiphenyl-4-yloxy)-2-propenoate | 121.71 | 99.10 | 101.60 | 20.11 |
| 1409 | 2-Acetyl-1,4-phenylene bis(4-octyloxybenzoate) | 110.19 | 129.60 | 130.30 | -20.11 |
| 1410 | 1,11-Bis(4-(2-hydroxy-4-octyloxyphenyliminomethyl)phenyloxy)undecane | 209.63 | 187.30 | 189.50 | 20.13 |
| 1411 | (1-Butoxycarbonyl)ethyl 5-(3-fluoro-4-hexadecyloxybenzoyloxyphenylethynyl)-thiophene-2-carboxylate | 116.27 | 136.50 | 136.40 | -20.13 |
| 1412 | 5-(4-Octyloxyphenyl)-N-(5-octyl-2-thienylmethylene)-1,3,4-thiadiazole-2-amine | 72.56 | 93.30 | 92.70 | -20.14 |
| 1413 | (3,4-Dicyano-2,5-thiophenediyl) bis(ethynediyl-4,1-phenylene) 3,4-bis(hexyloxy)benzoate | 103.85 | 119.30 | 124.00 | -20.15 |
| 1414 | Cholesteryl 6-(4-heptyloxybiphenyl-4'-oxy)hexanoate | 80.34 | 102.30 | 100.50 | -20.16 |
| 1415 | Cholesteryl 6-(4-butoxybiphenyl-4'-oxy)hexanoate | 69.79 | 90.20 | 90.00 | -20.21 |
| 1416 | Bis(4-heptyloxycarbonylphenyl) terephthalate | 111.97 | 131.80 | 132.20 | -20.23 |
| 1417 | 4-Cyano-4'-hexylbiphenyl | 59.36 | 79.00 | 79.60 | -20.24 |
| 1418 | 1-(2-(5-Cyanothienyl))-3-(4-heptyloxyphenylamino)-2-propen-1-one | 40.43 | 62.20 | 60.70 | -20.27 |
| 1419 | Cholesteryl 8-(4-(4-butylphenylethynyl)phenoxy)octanoate | 113.88 | 93.30 | 93.60 | 20.28 |
| 1420 | 4-(4-Hexyloxyphenylazo)phenyl 3-(4-(4-octyloxybenzoyloxy)phenylmethyleneamino)benzoate | 114.62 | 134.50 | 134.90 | -20.28 |
| 1421 | 4-Octylphenyl 2-chloro-(4-heptylbenzoyloxy)benzoate | 99.90 | 120.80 | 120.20 | -20.30 |
| 1422 | 4-Pentylcyclohexyl 4-pentyloxycinnamate | 62.50 | 80.10 | 82.80 | -20.30 |
| 1423 | 4-(4-Cyanophenyl)-1-(4-octyloxyphenyl)-butadiene | 96.51 | 76.40 | 76.20 | 20.31 |
| 1424 | 4-(4-Chlorophenylthiocarbonyl)phenyl 4-(heptyloxy)benzoate | 111.13 | 90.70 | 90.80 | 20.33 |
| 1425 | 5,5''''-Didecylquinquethiophene | 114.30 | 132.70 | 134.70 | -20.40 |
| 1426 | Butyl 2-cyano-3-(4-(4-dodecanoyl-1-piperazinyl)phenylazophenyl)-2-propenoate | 37.79 | 54.80 | 58.20 | -20.41 |
| 1427 | 4-Bromophenyl cholest-5-ene-3beta-carboxylate | 62.47 | 81.10 | 82.90 | -20.43 |
| 1428 | 1,4-Bis(4-nitroazobenzene-4'-oxy)butane | 102.76 | 88.50 | 82.30 | 20.46 |
| 1429 | 3-Methylpentyl 4-(4-(4-acetoxybenzoyloxy)benzylideneamino)benzoate | 72.24 | 95.00 | 92.70 | -20.46 |
| 1430 | 1-(6-Pentyloxy-2-naphthyl)-2-(3,4-difluorophenyl)acetylene | 52.33 | 72.50 | 72.80 | -20.47 |
| 1431 | N,N'-Ditetradecanoyl-2,3,5,6-tetrakis(tetradecanoyloxy)-1,4-benzenediamine | 329.60 | 348.30 | 350.10 | -20.50 |
| 1432 | Benzene hexa-n-nonanoate | 291.71 | 269.70 | 271.20 | 20.51 |
| 1433 | 4-Chlorophenyl 4-nonyloxythiobenzoate | 115.12 | 93.60 | 94.60 | 20.52 |
| 1434 | 4-(3,3,4,4,5,5,6,6,7,7,8,8,8-Tridecafluorooctyloxycarbonyl)phenyl 4-(nonyloxy)benzoate | 104.58 | 125.50 | 125.10 | -20.52 |
| 1435 | Di-(4-(2-ethoxycarbonylvinyl)phenyl terephthalate | 100.83 | 79.50 | 80.30 | 20.53 |
| 1436 | 3,4,5-Tris(dodecyloxy)benzamide | 95.85 | 113.00 | 116.40 | -20.55 |
| 1437 | 1,3-Phenylene bis(4-(4-dodecyloxyphenyliminomethyl)benzoate) | 150.23 | 171.20 | 170.80 | -20.57 |
| 1438 | 4-Cyanophenyl 4-heptylbenzoate | 114.88 | 94.00 | 94.30 | 20.58 |
| 1439 | Cholesteryl dodecanoate | 87.40 | 107.30 | 108.00 | -20.60 |
| 1440 | 5,5''''-Didecylsexithiophene | 149.52 | 130.60 | 128.90 | 20.62 |
| 1441 | 4-(4-(1-Methylheptyloxycarbonyl)phenyloxycarbonyl)phenyl 4-(5-(4-(4-pentadecyloxybenzoyloxy)benzoyloxy)pentyloxy)benzoate | 192.53 | 171.60 | 171.90 | 20.63 |
| 1442 | 4-(2,2,3,3,4,4,5,5-octafluoropentyloxycarbonyl)phenyl 4-(heptyloxy)benzoate | 71.74 | 90.50 | 92.40 | -20.66 |
| 1443 | 2-Octanoyl-5-(4-butoxyphenyl)thiophene | 82.14 | 103.30 | 102.80 | -20.66 |
| 1444 | 4-(4-Cyanophenoxycarbonyl)phenyl 3-(4-(4-dodecyloxybenzoyloxy)benzoyloxy)benzoate | 111.12 | 131.00 | 131.80 | -20.68 |
| 1445 | 4'-(2-(4-Hexylphenyl)-1-ethynyl)-2'-ethyl-4-ethyltolane | 45.69 | 66.80 | 66.40 | -20.71 |
| 1446 | 4-n-Dodecyloxycinnamic acid | 144.22 | 124.00 | 123.50 | 20.72 |
| 1447 | N-(4-Hexyloxyphenyl)-6-decyloxyquinoline-2-carboxamide | 131.53 | 109.40 | 110.80 | 20.73 |
| 1448 | 4-Cyanophenyl 4-(3-(4-(4'-octadecylbiphenyl-4-carbonyloxy)-2-fluorobenzoyloxy)benzoyloxy)benzoate | 129.86 | 149.40 | 150.60 | -20.74 |
| 1449 | 4,4'-Dihexanoyloxydiphenyldiacetylene | 127.68 | 105.90 | 106.90 | 20.78 |
| 1450 | 2-Chloro-4-methylpentyl 4-(3-(4-heptyloxyphenyl)-1-oxo-2-propenyloxy)benzoate | 105.59 | 84.30 | 84.80 | 20.79 |
| 1451 | 2,6,10-Tris(pentyloxy)-3,7,11-tris(propoxy)triphenylene | 104.39 | 84.20 | 83.60 | 20.79 |
| 1452 | 4-Isopropylphenyl 4-(4-propoxybenzylideneamino)benzoate | 98.29 | 77.30 | 77.50 | 20.79 |
| 1453 | Cholesteryl nonanoate | 73.11 | 95.20 | 93.90 | -20.79 |
| 1454 | 4-Pentyloxybenzylidene 4'-tetradecylaniline | 158.84 | 137.20 | 138.00 | 20.84 |
| 1455 | 5-(4-Octyloxyphenyl)-2-(4-octyloxy)phenylamido-1,3,4-thiadiazole | 52.56 | 72.60 | 73.40 | -20.84 |
| 1456 | 3-(2-Methylphenyl-5-(4-(4-pentylcyclohexyl)phenyl)-1,2,4-oxadiazole | 83.46 | 62.80 | 62.60 | 20.86 |
| 1457 | 5-(4-Pyridyl)-2-(4-octyloxyphenylamido)-1,3,4-thiadiazole | 39.23 | 59.20 | 60.10 | -20.87 |
| 1458 | 4-(4-Ethoxyphenylazo)phenyl 4-decyloxybenzoate | 99.65 | 119.00 | 120.60 | -20.95 |
| 1459 | N-(7-Oxo-4-(4-(1-octadecanoylamino)phenyl)-1,3,5-cycloheptatrien-1-yl)octadecamide | 143.15 | 158.10 | 164.10 | -20.95 |
| 1460 | 4-Heptanoyloxy-2-methyl-4'-ethoxyazobenzene | 119.86 | 99.70 | 98.90 | 20.96 |
| 1461 | Bis(4-ethoxyphenyl) 2-(9-(4-(4-biphenylazo)phenoxy)nonyloxy) terephthalate | 139.16 | 119.20 | 118.20 | 20.96 |
| 1462 | Diethyl N,N'-(1,4-phenylene-bis(methylidene))-bis(aminobenzoate) | 64.53 | 85.50 | 85.50 | -20.97 |
| 1463 | 3,3',4,4'-Biphenyltetracarboxy-N,N'-bis(octylphenyl)diimide | 142.28 | 123.10 | 121.30 | 20.98 |
| 1464 | 3,4,5-Tridodecyloxybenzaldehyde-2',4'-dinitrophenyl hydrazone | 127.91 | 150.20 | 148.90 | -20.99 |
| 1465 | 4-Decyloxy-4'-cyanobiphenyl | 118.31 | 96.40 | 97.30 | 21.01 |
| 1466 | Isopropyl 4-(4-(4-methoxybenzoyloxy)benzylideneamino)benzoate | 90.03 | 68.40 | 69.00 | 21.03 |
| 1467 | S-(4-Octyloxyphenyl) 4-butoxythiobenzoate | 124.84 | 102.50 | 103.80 | 21.04 |
| 1468 | Ethyl 4-(4-ethoxybenzylideneamino)cinnamate | 95.47 | 74.60 | 74.40 | 21.07 |
| 1469 | 1-Methylheptyl 4-(2-fluoro-4-(4-decyloxybenzoyloxy)phenylethynyl)benzoate | 92.79 | 112.90 | 113.90 | -21.11 |
| 1470 | 5-(4'-Octyloxybiphenyl-4-yl)-2-(4-methylphenyl)pyridine | 40.29 | 60.50 | 61.40 | -21.11 |
| 1471 | 4-Hexadecyloxyphenyl 4-cyanobenzoate | 152.22 | 131.50 | 131.10 | 21.12 |
| 1472 | 1,3-Phenylene bis(4-(4-ethoxybenzylideneamino)benzoate) | 69.46 | 90.40 | 90.60 | -21.14 |
| 1473 | Phenyl 4-(phenyliminomethyl)phenyl 1,4-benzenedicarboxylate | 114.37 | 93.00 | 93.20 | 21.17 |
| 1474 | (3,4-Dicyano-2,5-thiophenediyl) bis(ethynediyl-4,1-phenylene) 3,4-bis(butoxy)benzoate | 107.93 | 87.00 | 86.70 | 21.23 |
| 1475 | 4-(3,3,4,4,5,5,6,6,7,7,8,8,8-Tridecafluorooctyloxycarbonyl)phenyl 4-(octyloxy)benzoate | 100.65 | 121.50 | 121.90 | -21.25 |
| 1476 | 3,3',4,4'-Biphenyltetracarboxy-N,N'-bis(heptyloxyphenyl)diimide | 136.66 | 117.30 | 115.40 | 21.26 |
| 1477 | N-(4-Hexyloxyphenylmethylene)-5-(4-octyloxyphenyl)-1,3,4-thiadiazol-2-amine | 103.67 | 82.90 | 82.40 | 21.27 |
| 1478 | 1,10-Bis(4-nitroazobenzene-4'-oxy)decane | 132.60 | 112.70 | 111.30 | 21.30 |
| 1479 | 1-Methylheptyl 4'-(4''-octyloxy-3''-fluorobenzoyloxy)tolan-4-carboxylate | 83.59 | 104.80 | 104.90 | -21.31 |
| 1480 | 3-(2-(2-Bromoethoxy)ethoxy)-cholest-5-ene | 68.12 | 46.80 |  | 21.32 |
| 1481 | Isopropyl 4-(4-(4-cyanobenzoyloxy)benzylideneamino)benzoate | 86.71 | 65.30 | 65.30 | 21.41 |
| 1482 | 4-Octadecyloxybenzoic acid | 177.08 | 154.20 | 155.60 | 21.48 |
| 1483 | 1,5-Bis(4-(2-hydroxy-4-undecyloxybenzylideneamino)benzoyloxy)-3,3-dimethylpentane | 183.38 | 163.20 | 161.90 | 21.48 |
| 1484 | 4-Chlororesorcinyl bis(4-(4-decyloyxbenzoyloxy)benzoate) | 174.19 | 153.80 | 152.70 | 21.49 |
| 1485 | 4,4'''-Dipentadecyloxy-p-quaterphenyl | 186.91 | 163.50 | 165.40 | 21.51 |
| 1486 | 4-Pentylphenyl 2-chloro-(4-pentylbenzoyloxy)benzoate | 79.08 | 100.60 | 100.60 | -21.52 |
| 1487 | 4-Chloro-2'-hydroxy-4'-heptyloxyazobenzene | 109.03 | 87.80 | 87.50 | 21.53 |
| 1488 | 1,3-Phenylene bis(4-(4-(7-octenyloxy)phenyliminomethyl)benzoate) | 166.85 | 143.90 | 145.30 | 21.55 |
| 1489 | 2,4-Bis(4-dodecyloxyazobenzene-4'-amino)-6-methoxy-1,3,5-triazine | 115.04 | 136.60 |  | -21.56 |
| 1490 | Bis(4-(4-(4-butyoxyphenyliminomethyl)phenoxy)butyl) 2-methylmalonate | 107.02 | 128.50 | 128.60 | -21.58 |
| 1491 | 3'-Fluoro-4-(4,4,4-trifluorobut-2-enyloxy)-4'-(4-pentylcyclohexyl)biphenyl | 68.18 | 51.90 | 46.60 | 21.58 |
| 1492 | 1,3-Bis(4-(4-octyloxyphenylazo)phenoxy)-2-propanol | 122.42 | 141.50 | 144.00 | -21.58 |
| 1493 | Bis(4-Octyloxyphenyl) 2,5-thiophenedicarboxylate | 160.00 | 138.00 | 138.40 | 21.60 |
| 1494 | 4-(2,2,3,3,4,4,4-Heptafluorobutyloxycarbonyl)phenyl 4-(hexyloxy)benzoate | 75.36 | 95.30 | 97.00 | -21.64 |
| 1495 | 1-(4-Decylbiphenyl-4'-yl)-3-(4-chlorophenyl)propane-1,3-dione | 121.07 | 100.10 | 99.40 | 21.67 |
| 1496 | 1-(6-Hexyloxy-2-naphthyl)-2-(3,4-difluorophenyl)acetylene | 54.82 | 76.60 | 76.50 | -21.68 |
| 1497 | 3,6,7,10,11-Pentabutoxy-2-triphenylenyl 2-chloro-4-methylpentanoate | 46.00 | 68.40 | 67.70 | -21.70 |
| 1498 | 1-Methylheptyl 4-(2-fluoro-4-(4-pentadecyloxybenzoyloxy)phenylethynyl)benzoate | 154.52 | 133.10 | 132.80 | 21.72 |
| 1499 | 1-(4-(4-Formyl)phenylazophenyl)-4-(1-oxododecyl)piperazine | 43.98 | 60.30 | 65.70 | -21.72 |
| 1500 | Pentyl 4-(4-(4-acetoxybenzoyloxy)benzylideneamino)benzoate | 81.37 | 103.50 | 103.10 | -21.73 |
| 1501 | 4,4'-Bis(4-propylcyclohexylethyl)benzene | 54.75 | 76.70 | 76.50 | -21.75 |
| 1502 | 1,5-(4-(2-Hydroxy-4-dodecyloxybenzylideneamino)benzoyloxy)-2,2-dimethylpentane | 149.51 | 171.20 | 171.30 | -21.79 |
| 1503 | 4-(2-Methylbutoxy)phenyl 4-(4-heptyloxy-2,3,5,6-tetrafluorophenylethynyl)benzoate | 73.30 | 95.00 | 95.10 | -21.80 |
| 1504 | 4,4'''-Diundecyloxy-p-quaterphenyl | 152.02 | 131.20 | 130.20 | 21.82 |
| 1505 | 4-(3-(4-(Decyloxy)phenyl)-1-oxo-2-propenyloxy)phenyl 2-chloro-4-methylpentanoate | 79.48 | 99.40 | 101.30 | -21.82 |
| 1506 | 1-(2-(5-Butylthienyl))-3-(4-fluorophenylamino)-2-propen-1-one | 77.75 | 58.20 | 55.90 | 21.85 |
| 1507 | 1-(4-Pentyloxyphenylethynyl)-4-(4-cyanophenylethynyl)benzene | 42.45 | 62.90 | 64.30 | -21.85 |
| 1508 | Di(4'-pentyloxyphenyl)-cyclohexane-1,4-dicarboxylate | 84.65 | 105.50 | 106.50 | -21.85 |
| 1509 | N,N'-Didecylbenzene-1,2-dicarboxamide | 141.15 | 160.10 | 163.00 | -21.85 |
| 1510 | 5-(4-Decyloxyphenyl)-N-(4-(2-methylbutoxy)phenylmethylene)-1,3,4-thiadiazol-2-amine | 97.15 | 74.30 | 75.30 | 21.85 |
| 1511 | 5-(4-Octyloxyphenyl)-N-(4-butoxyphenylmethylene)-1,3,4-thiadiazol-2-amine | 95.88 | 74.80 | 74.00 | 21.88 |
| 1512 | 4-Cyanophenyl 4-(3-(4-(4'-nonylbiphenyl-4-carbonyloxy)benzoyloxy)benzoyloxy)benzoate | 91.42 | 112.00 | 113.30 | -21.88 |
| 1513 | 4-(4-Ethoxybenzoyloxy)-2-dodecyloxy-4'-(4-butyoxysalicylaldimino)azobenzene | 149.00 | 126.00 | 127.10 | 21.90 |
| 1514 | Bis(4-hexyloxyphenyl) 4,4'-biphenylenedicarboxylate | 135.72 | 112.60 | 113.80 | 21.92 |
| 1515 | 1-(4-Heptylbiphenyl-4'-yl)-3-(4-cyanobiphenyl-4'-yl)propane-1,3-dione | 59.34 | 81.40 | 81.30 | -21.96 |
| 1516 | 2-Octanoyl-1,4-phenylene bis(4-octyloxybenzoate) | 175.87 | 153.90 | 153.90 | 21.97 |
| 1517 | N-(4-Tetradecloxybenzylidene)-4-ethylaniline | 148.04 | 125.10 | 126.00 | 22.04 |
| 1518 | 1-(4-(N-Ethoxycarbonylpiperazinyl)phenyl)-3-(4-heptyloxyphenyl)-1-propargyl-3-imine | 50.45 | 68.20 | 72.50 | -22.05 |
| 1519 | 4'-Undecyloxybiphenyl-4-yl 2-(2-methylbutyl)-1,3-dioxoisoindole-5-carboxylate | 71.55 | 95.70 | 93.60 | -22.05 |
| 1520 | 4-Heptyl-4'-hexadecyloxyazobenzene | 178.76 | 157.10 | 156.70 | 22.06 |
| 1521 | 4-(4-Chlorophenyl)-2,6-bis(3,4,5-trioctyloxyphenyl)pyrimidine | 107.53 | 126.40 | 129.60 | -22.07 |
| 1522 | 4-(4-(2-Ethoxy-1-methyl-2-oxoethoxycarbonyl)phenylethynyl)phenyl 2,3-difluoro-4-octadecyloxybenzoate | 156.07 | 134.00 | 134.00 | 22.07 |
| 1523 | 1-Methylpropyl 4-(4-(4-cyanobenzoyloxy)benzylideneamino)benzoate | 89.37 | 69.30 | 67.30 | 22.07 |
| 1524 | Pentadecyl 2-(4-decyloxyphenyl)-6-cycloheptimidazolecarboxylate | 59.83 | 84.30 | 81.90 | -22.07 |
| 1525 | 4-(4-Decyloxybenzylideneamino)phenyl 4-ethylbenzoate | 92.21 | 114.10 | 114.30 | -22.09 |
| 1526 | 4-Cyanophenyl 4-(3-(4-(4'-decylbiphenyl-4-carbonyloxy)-3-fluorobenzoyloxy)benzoyloxy)benzoate | 95.28 | 117.00 | 117.40 | -22.12 |
| 1527 | 1-(6-Butoxy-2-naphthyl)-4-(3,4-difluorophenyl)diacetylene | 86.65 | 64.70 | 64.50 | 22.15 |
| 1528 | 5-(4-Decyloxyphenyl)-N-(5-octyl-2-thienylmethylene)-1,3,4-thiadiazole-2-amine | 120.59 | 101.40 | 98.40 | 22.19 |
| 1529 | Ethyl 4-(4-(4-methylbenzoyloxy)benzylideneamino)benzoate | 102.24 | 80.50 | 80.00 | 22.24 |
| 1530 | 2-Nonanoyl-1,4-phenylene bis(4-octyloxybenzoate) | 180.74 | 157.90 | 158.50 | 22.24 |
| 1531 | 1,6-Bis(4-(4-ethoxybenzoyloxybenzylideneamino)hexane | 138.75 | 117.00 | 116.50 | 22.25 |
| 1532 | 4-Thiocyanophenyl 4-(4-butoxybenzoyloxy)benzoate | 108.09 | 85.80 | 85.80 | 22.29 |
| 1533 | 1-Methylpentyl 4-(4-(4-acetoxybenzoyloxy)benzylideneamino)benzoate | 66.91 | 88.90 | 89.20 | -22.29 |
| 1534 | 5alpha-Cholestan-3beta-ol octanoate | 116.40 | 96.90 | 94.10 | 22.30 |
| 1535 | 4'-(2-(4-Hexylphenyl)-1-ethynyl)-3-methyl-4-propyltolane | 88.42 | 66.80 | 66.10 | 22.32 |
| 1536 | 4-Chlororesorcinyl bis(4-(4-undecyloyxbenzoyloxy)benzoate) | 184.35 | 161.90 | 162.00 | 22.35 |
| 1537 | 1,6-Bis(4-ethoxyazobenzene-4'-carbonyloxy)hexane | 133.87 | 113.70 | 111.50 | 22.37 |
| 1538 | 4-(4-Heptyloxyphenylmethyleneamino)phenyl 3-pyridinecarboxylate | 138.00 | 114.00 | 115.60 | 22.40 |
| 1539 | 4-Methylpentyl 4-(4-(4-acetoxybenzoyloxy)benzylideneamino)benzoate | 72.58 | 95.00 | 95.00 | -22.42 |
| 1540 | 1,5-Bis(4-(2-hydroxy-4-dodecyloxybenzylideneamino)benzoyloxy)pentane | 143.48 | 165.20 | 165.90 | -22.42 |
| 1541 | Hexa(4-heptyloxyphenoxymethyl)benzene | 208.44 | 184.30 | 186.00 | 22.44 |
| 1542 | 1-(4-Dodecylbiphenyl-4'-yl)-3-(4-chlorophenyl)propane-1,3-dione | 129.27 | 108.20 | 106.80 | 22.47 |
| 1543 | 1,3-Phenylene bis(4-(4'-dodecylbiphenyl-4-carbonyloxy)-3-fluorobenzoate) | 143.63 | 165.50 | 166.10 | -22.47 |
| 1544 | Hexyl 4-(4-(4-methoxybenzylideneamino)benzoyloxy)benzoate | 122.11 | 99.20 | 99.60 | 22.51 |
| 1545 | 3-(4-Decyloxyphenyl)-5-(4-chlorophenyl)pyrazole | 127.09 | 108.30 | 104.50 | 22.59 |
| 1546 | 4-(2,2,3,3,4,4,5,5-Octafluoropentyloxycarbonyl)phenyl 4-(decyloxy)benzoate | 82.18 | 102.60 | 104.80 | -22.62 |
| 1547 | 5-(4-Pentyloxyphenyl)-2-(4-octyloxy)benzylideneamino-1,3,4-thiadiazole | 100.63 | 78.80 | 78.00 | 22.63 |
| 1548 | 3-(11-Bromoundecyloxy)-cholest-5-ene | 58.77 | 81.40 |  | -22.63 |
| 1549 | 2-Propanoyl-1,4-phenylene bis(4-octyloxybenzoate) | 155.60 | 133.70 | 132.90 | 22.70 |
| 1550 | N-(4-Butoxybenzylidene)-4-pentylaniline | 118.90 | 96.80 | 96.20 | 22.70 |
| 1551 | Methyl 5-((4-(4-decyloxyphenylmethyleneimino)phenyloxycarbonyl)-1,3-dioxoisoindole-2-acetate | 85.50 | 105.40 | 108.20 | -22.70 |
| 1552 | 1-Methylheptyl 4-(2-fluoro-4-(4-octadecyloxybenzoyloxy)phenylethynyl)benzoate | 167.82 | 145.20 | 145.10 | 22.72 |
| 1553 | 4-(4-(2-Ethoxy-1-methyl-2-oxoethoxycarbonyl)phenylethynyl)phenyl 3-fluoro-4-octadecyloxybenzoate | 110.98 | 133.00 | 133.70 | -22.72 |
| 1554 | 4-(2,2,3,3,4,4,4-Heptafluorobutyloxycarbonyl)phenyl 4-(dodecyloxy)benzoate | 97.97 | 119.60 | 120.70 | -22.73 |
| 1555 | 3,3',4,4'-Biphenyltetracarboxy-N,N'-bis(octyloxyphenyl)diimide | 104.84 | 125.40 | 127.60 | -22.76 |
| 1556 | 7-(4'-Tetradecyloxybenzoyloxy)isoflavone | 83.39 | 103.10 | 106.20 | -22.81 |
| 1557 | 4-Butylcyclohexyl 4-methoxycinnamate | 82.52 | 61.30 | 59.70 | 22.82 |
| 1558 | 7-(4'-Propoxybenzoyloxy)isoflavone | 80.13 | 58.70 | 57.30 | 22.83 |
| 1559 | 2,4-Bis(4-decyloxyazobenzene-4'-amino)-6-methoxy-1,3,5-triazine | 97.56 | 120.40 |  | -22.84 |
| 1560 | 1,3-Phenylene bis(4-(4'-undecylbiphenyl-4-carbonyloxy)-3-fluorobenzoate) | 134.04 | 157.40 | 156.90 | -22.86 |
| 1561 | 6-O-Tetradecanoyl-alpha-D-galactopyranose | 72.23 | 91.90 | 95.10 | -22.87 |
| 1562 | 6-O-Octadecanoyl-alpha-D-galactopyranose | 87.92 | 108.00 | 110.80 | -22.88 |
| 1563 | 4-(5-(4-Propylbiphenyl-1'-yl)-1,2,4-oxadiazol-3-yl)pyridine | 54.81 | 75.40 | 77.70 | -22.89 |
| 1564 | 4-(2-Methylbutoxycarbonyl)phenyl 4-(4-hexyloxy-2,3,4,5-tetrafluorophenylethynyl)benzoate | 69.19 | 93.70 | 92.10 | -22.91 |
| 1565 | 4'-(2-(4-Butylphenyl)-1-ethynyl)-2'-ethyl-4-ethyltolane | 81.23 | 58.70 | 58.30 | 22.93 |
| 1566 | 4-Butoxy-4'-methoxy-trans-stilbene | 89.70 | 69.30 | 66.70 | 23.00 |
| 1567 | N-(4-Butoxybenzylidene)-4-octylaniline | 132.51 | 108.90 | 109.50 | 23.01 |
| 1568 | N,N'-Diheptanoyl-2,3,5,6-tetramethylbenzene-1,4-diamine | 78.02 | 54.20 | 55.00 | 23.02 |
| 1569 | 4-Isothiocyanatophenyl 4-(4-butylcyclohexyl)benzoate | 69.67 | 92.20 | 92.70 | -23.03 |
| 1570 | 4-Pentanoyl-4'-propanoyloxyazobenzene | 79.36 | 100.90 | 102.40 | -23.04 |
| 1571 | 4-(4-Ethoxyphenylazo)-2,6-dimethylphenyl 4-(decyloxy)benzoate | 126.66 | 103.70 | 103.60 | 23.06 |
| 1572 | 2-Methyl-4-(4-pentylcyclohexylethynyl)-1-((4-cyanophenyl)ethynyl)benzene | 72.66 | 49.40 | 49.60 | 23.06 |
| 1573 | 1,3-Phenylene bis(4-(3-chloro-4-nonyloxyphenylethenyl)benzoate) | 79.01 | 104.80 | 102.10 | -23.09 |
| 1574 | Bis(4-ethoxyphenyl) 2-(5-(4-(4-butylphenylazo)phenoxy)pentyloxy) terephthalate | 137.00 | 113.20 | 113.90 | 23.10 |
| 1575 | 2-(4-Octyloxyphenyl)-5-(4-(4-ethyl-2,3-undecadienyloxy)phenyl-1,3,4-thiadiazole | 85.89 | 105.80 | 109.00 | -23.11 |
| 1576 | 4-Pentanoyl-4'-octanoyloxyazobenzene | 97.07 | 121.10 | 120.20 | -23.13 |
| 1577 | 6-O-Pentadecyl-D-galacitol | 164.45 | 144.10 | 141.30 | 23.15 |
| 1578 | 5-Methyl-5'-(2-(4-hexyloxyphenyl)-2-hydroxyethyl)-2,2'-bipyridine | 66.36 | 46.10 | 43.20 | 23.16 |
| 1579 | 1,3-Phenylene bis(4-(4'-octylbiphenyl-4-carbonyloxy)-2-ethylbenzoate) | 102.84 | 124.00 | 126.00 | -23.16 |
| 1580 | 4'-Undecyloxybiphenyl-4-yl 2-(2-methylbutyl)-1,3-dithioxoisoindole-5-carboxylate | 73.83 | 85.30 | 97.00 | -23.17 |
| 1581 | 3-(2-Bromophenyl-5-(4-(4-pentylcyclohexyl)phenyl)-1,2,4-oxadiazole | 92.88 | 69.60 | 69.70 | 23.18 |
| 1582 | 2,5-Dihydro-2,5-diethyl-3,6-bis(4-(4-pentylcyclohexyl)biphenyl-4'-yl)pyrrolo(3,4-c)pyrrole-1,4-dione | 73.20 | 58.60 | 50.00 | 23.20 |
| 1583 | 2-(4-Hexyloxyphenyl)-5-(4-decyloxy)phenylazo-1,3,4-thiadiazole | 118.20 | 94.60 | 95.00 | 23.20 |
| 1584 | 1-(4-Dodecylbiphenyl-4'-yl)-3-(3-octyloxyphenyl)propane-1,3-dione | 160.80 | 137.30 | 137.60 | 23.20 |
| 1585 | 5-(4'-Nonyloxybiphenyl-4-yl)-2-(4-methylphenyl)pyridine | 40.53 | 64.60 | 63.80 | -23.27 |
| 1586 | 1-Heptyl-4-(4-nonanoylphenyl)cyclohexane | 86.92 | 110.10 | 110.20 | -23.28 |
| 1587 | 4-Heptyloxy-4'-isothiocyanatotolane | 123.72 | 101.20 | 100.40 | 23.32 |
| 1588 | 1,3-Phenylene bis(4-(4-undecyloxybenzoylthio)benzoate) | 126.36 | 147.90 | 149.70 | -23.34 |
| 1589 | 4-Propoxyphenylbenzyl perfluorohexyl thioether | 59.15 | 77.50 | 82.50 | -23.35 |
| 1590 | 4-Nonyl-N-(4-octadecyloxyphenylmethylene)benzenamine | 194.56 | 169.50 | 171.20 | 23.36 |
| 1591 | 4-(4-Octyloxyphenylmethyleneamino)phenyl 3-pyridinecarboxylate | 140.57 | 118.10 | 117.20 | 23.37 |
| 1592 | 1-Methylheptyl 4-(4-(4-dodecyloxycinnamoyloxy)benzoyloxy)benzoate | 106.71 | 128.70 | 130.10 | -23.39 |
| 1593 | 4-(4-Octyloxyphenylazoxy)phenyl 2-chloro-3-methylpentanoate | 118.29 | 95.00 | 94.90 | 23.39 |
| 1594 | 4'-(2-(4-Propylphenyl)-1-ethynyl)-2'-methyl-4-methyltolane | 69.01 | 46.60 | 45.60 | 23.41 |
| 1595 | 4-(3-Decyloxyphenyliminomethyl)phenyl 4-dodecyloxybenzoate | 178.55 | 155.70 | 155.10 | 23.45 |
| 1596 | 4'-Undecyloxybiphenyl-4-yl 2-(2-methylbutyl)-1-oxo-3-thioxoisoindole-5-carboxylate | 70.63 | 90.50 | 94.10 | -23.47 |
| 1597 | 1,3-Phenylene bis(4-(4'-hexylbiphenyl-4-carbonyloxy)-3-fluorobenzoate) | 94.19 | 117.00 | 117.70 | -23.51 |
| 1598 | 1,5-Bis(4-(2-hydroxy-4-pentyloxybenzylideneamino)benzoyloxy)-2,2-dimethylpentane | 91.19 | 114.70 | 114.70 | -23.51 |
| 1599 | N-(4-Propoxybenzylidene)-4-octylaniline | 127.72 | 104.90 | 104.20 | 23.52 |
| 1600 | 4-(4-Octyloxy-2,3,5,6-tetrafluorophenylacetylenyl)phenyl 4'-chlorobenzoate | 71.43 | 94.70 | 95.00 | -23.57 |
| 1601 | 1-(2-(5-Cyanothienyl))-3-(4-hexyloxyphenylamino)-2-propen-1-one | 34.96 | 58.20 | 58.60 | -23.64 |
| 1602 | 4-(1-Methylheptyloxycarbonyl)phenyl 4-(4-decyloxyphenylethyl)benzoate | 99.49 | 123.80 | 123.20 | -23.71 |
| 1603 | Cholesteryl ethyl carbonate | 60.97 | 84.70 |  | -23.73 |
| 1604 | Di-(4-butoxycarbonylphenyl) 2-chloroterephthalate | 77.17 | 100.30 | 100.90 | -23.73 |
| 1605 | 1-(4'-Butylazobenzene-4-oxy)-6-(4'-propylazobenzene-4-oxy)hexane | 96.37 | 118.10 | 120.10 | -23.73 |
| 1606 | 4'-Decyloxybiphenyl-4-yl 2-butyl-1,3-dioxoisoindole-5-carboxylate | 78.86 | 100.20 | 102.60 | -23.74 |
| 1607 | 1-Methylheptyl 4-(4-(3-fluoro-4-dodecyloxybenzoyloxy)-3-fluorobenzoyloxy)-2-fluorobenzoate | 113.05 | 137.70 | 136.80 | -23.75 |
| 1608 | Cyclononanone | 64.90 | 87.30 | 88.70 | -23.80 |
| 1609 | 4-(3-(4-(Undecyloxy)phenyl)-1-oxo-2-propenyloxy)phenyl 2-chloro-3-methylpentanoate | 80.98 | 103.40 | 104.80 | -23.82 |
| 1610 | 4'-(2-(4-Hexylphenyl)-1-ethynyl)-2'-methyl-4-propyltolane | 46.55 | 66.80 | 70.40 | -23.85 |
| 1611 | 1-Methylheptyl 4-(4-(4-undecyloxycinnamoyloxy)benzoyloxy)benzoate | 102.74 | 124.70 | 126.60 | -23.86 |
| 1612 | 1-(2-(5-Chlorothienyl))-3-(4-pentyloxyphenylamino)-2-propen-1-one | 48.96 | 70.40 | 72.90 | -23.94 |
| 1613 | Bis(4-(4-hexadecyloxybenzylideneamino)phenyl decanedioate | 265.26 | 241.60 | 241.30 | 23.96 |
| 1614 | 2-Methoxy-4'-pentyloxy-trans-stilbene | 96.47 | 73.40 | 72.50 | 23.97 |
| 1615 | 4-Dodecyloxy-2-hydroxybenzoic acid | 99.17 | 120.90 | 123.20 | -24.03 |
| 1616 | 4-Thiocyanophenyl 4-(4-methoxybenzoyloxy)benzoate | 98.66 | 75.10 | 74.60 | 24.06 |
| 1617 | 4-(4-(1-Methylheptyloxycarbonyl)phenyloxycarbonyl)phenyl 4-(5-(4-(4-tetradecyloxybenzoyloxy)benzoyloxy)pentyloxy)benzoate | 192.40 | 167.60 | 168.30 | 24.10 |
| 1618 | 4-(2-(4-(7-hydroxyheptyloxy)phenyl)vinyl)benzonitrile | 70.09 | 93.80 | 94.20 | -24.11 |
| 1619 | 4,4'-Dibutoxyazoxybenzene | 73.87 | 94.70 | 98.00 | -24.13 |
| 1620 | 2-Cyano-1,3-phenylene bis(4-(4-hexyloxyphenyliminomethyl)benzoate) | 89.85 | 114.50 | 114.00 | -24.15 |
| 1621 | N-(4-Pentyloxyphenyl)-6-decyloxyquinoline-2-carboxamide | 130.97 | 105.40 | 106.80 | 24.17 |
| 1622 | Heptyl 4-(4-(4-methoxybenzylideneamino)benzoyloxy)benzoate | 125.38 | 103.30 | 101.20 | 24.18 |
| 1623 | 1-(4-Butylcyclohexylethynyl)-4-(4-pentylphenylethynyl)benzene | 46.82 | 69.80 | 71.00 | -24.18 |
| 1624 | 5-(4'-Decyloxybiphenyl-4-yl)-2-(4-methylphenyl)pyridine | 44.32 | 68.60 | 68.50 | -24.18 |
| 1625 | Cholesteryl 5-(2,3-difluoro-4-decyloxybiphenyl-4'-oxy)pentanoate | 135.62 | 112.40 | 111.40 | 24.22 |
| 1626 | Bis(4-(3-decyloxy-3-oxo-1-propenyl)phenyl terephthalate | 168.63 | 144.20 | 144.40 | 24.23 |
| 1627 | 4-(2-Chloro-3-methylpentanoyloxy)biphenyl-4'-yl 4-dodecyloxy-2,3-difluorobenzoate | 132.97 | 109.70 | 108.70 | 24.27 |
| 1628 | 2,6,10-Tris(pentyloxy)-3,7,11-tris(heptyloxy)triphenylene | 109.17 | 132.70 | 133.50 | -24.33 |
| 1629 | 4-Dodecyloxybenzoic acid | 107.16 | 129.90 | 131.50 | -24.34 |
| 1630 | 1-(2-(5-Cyanothienyl))-3-(4-pentyloxyphenylamino)-2-propen-1-one | 30.64 | 54.20 | 55.00 | -24.36 |
| 1631 | 4-Cyanophenyl 4-(3-(4-(4'-tetradecylbiphenyl-4-carbonyloxy)-2-fluorobenzoyloxy)benzoyloxy)benzoate | 109.00 | 133.20 | 133.40 | -24.40 |
| 1632 | 2-Methyl-4-(6,7,10,11-tetrakis(pentyloxy)-3-pentylthio-2-triphenylenyl)-3-butyn-2-ol | 80.19 | 106.70 | 104.60 | -24.41 |
| 1633 | 4-(4'-Nonylcyclohexyl)isothiocyanatobenzene | 131.75 | 107.20 | 107.30 | 24.45 |
| 1634 | 1,10-Bis(4-(4-heptyloxybenzoyloxybenzylideneamino)decane | 151.84 | 173.60 | 176.30 | -24.46 |
| 1635 | 2,5-Thiophenediylbis(ethynediyl-4,1-phenylene) 3,4-bis(heptyloxy)benzoate | 172.77 | 146.50 | 148.30 | 24.47 |
| 1636 | 4,4'-Bis(4-(4-decyloxybenzylideneamino)benzoyloxy)diphenylmethane | 179.42 | 153.40 | 154.90 | 24.52 |
| 1637 | 1-Methpropyl 4-(4-(4-Nitrobenzoyloxy)benzylideneamino)benzoate | 43.78 | 69.30 | 68.30 | -24.52 |
| 1638 | Bis(4-(5-pentylthio-1,3,4-oxadiazole-2-yl)phenyl) terephthalate | 96.02 | 73.50 | 71.50 | 24.52 |
| 1639 | Pentyl 4-(4-(4-methoxybenzylideneamino)benzoyloxy)benzoate | 71.03 | 95.20 | 95.60 | -24.57 |
| 1640 | 4-(4-Decyloxyphenylmethyleneamino)phenyl 3-pyridinecarboxylate | 149.88 | 126.20 | 125.30 | 24.58 |
| 1641 | 2-Acetyl-1,4-phenylene bis(4-heptyloxybenzoate) | 144.89 | 121.50 | 120.30 | 24.59 |
| 1642 | 4-(4-Fluorophenyl)-2,6-bis(3,4,5-trioctyloxyphenyl)pyrimidine | 108.46 | 134.60 | 133.10 | -24.64 |
| 1643 | 1-Methylheptyl 5-(4-octadecyloxybenzoyloxyphenylethynyl)-thiophene-2-carboxylate | 123.42 | 147.70 | 148.10 | -24.68 |
| 1644 | 4-Methylpentyl 4-(4-(4-chlorobenzoyloxy)benzylideneamino)benzoate | 107.29 | 84.50 | 82.60 | 24.69 |
| 1645 | 1,3-Phenylene bis(4-(4'-decylbiphenyl-4-carbonyloxy)-2-fluorobenzoate) | 125.41 | 149.30 | 150.10 | -24.69 |
| 1646 | 1,4-Bis(4-pentylazobenzene-4'-oxy)butane | 98.51 | 122.10 | 123.20 | -24.69 |
| 1647 | N,N',N''-Tridecanoyl-2,4,6-trimethyl-1,3,5-benzenetriamine | 75.51 | 100.40 | 100.20 | -24.69 |
| 1648 | 5-Methyl-5'-(2-(4-dodecyloxyphenyl)-2-hydroxyethyl)-2,2'-bipyridine | 93.81 | 70.30 | 69.10 | 24.71 |
| 1649 | 2-Methylpropyl 4-(4-(4-methoxybenzylideneamino)benzoyloxy)benzoate | 103.12 | 78.50 | 78.40 | 24.72 |
| 1650 | 7-(4'-Methoxybenzoyloxy)isoflavone | 29.18 | 52.00 | 53.90 | -24.72 |
| 1651 | 4-Cyano-1,3-phenylene bis(4-(4-hexyloxyphenyliminomethyl)benzoate) | 89.36 | 114.50 | 114.10 | -24.74 |
| 1652 | 1,3-Phenylene bis(4-(4'-undecylbiphenyl-4-carbonyloxy)-2-fluorobenzoate) | 131.22 | 157.40 | 156.00 | -24.78 |
| 1653 | 4,6-Dichloro-1,3-phenylene bis(4-(4-decyloxyphenyliminomethyl)benzoate) | 117.60 | 140.60 | 142.40 | -24.80 |
| 1654 | 2-Cyano-1,3-phenylene bis(4-(4-heptyloxyphenyliminomethyl)benzoate) | 98.37 | 122.60 | 123.20 | -24.83 |
| 1655 | 4-Octyloxy-N-(4-cyanobenzylidene)aniline | 121.06 | 96.20 | 96.20 | 24.86 |
| 1656 | 1,10-Diethyl(permethyl)decasilane | 74.10 | 99.00 |  | -24.90 |
| 1657 | 2-Cyano-1,3-phenylene bis(4-(4'-hexadecylbiphenyl-4-carbonyloxy)-2-fluorobenzoate) | 215.51 | 189.60 | 190.60 | 24.91 |
| 1658 | Bis(4-(5-octylthio-1,3,4-oxadiazole-2-yl)phenyl)-2,5-thiophenedicarboxylate | 78.59 | 101.30 | 103.50 | -24.91 |
| 1659 | 1,10-Bis(4-(4-butyloxybenzoyloxybenzylideneamino)decane | 172.72 | 149.40 | 147.80 | 24.92 |
| 1660 | 4-(4-Pentoxy-2,3,5,6-tetrafluorophenylacetylenyl)phenyl 4'-chlorobenzoate | 57.95 | 82.60 | 82.90 | -24.95 |
| 1661 | 1,4-Bis(4-(4-heptyloxybenzoyloxybenzylideneamino)butane | 175.07 | 149.40 | 150.10 | 24.97 |
| 1662 | 1,3-Bis(4-(4-pentyloxybenzoyloxy)benzylideneamino)benzene | 89.01 | 114.70 | 114.00 | -24.99 |
| 1663 | 4-Heptylcyclohexyl 4-heptyloxycinnamate | 120.82 | 96.30 | 95.80 | 25.02 |
| 1664 | 4-Propanoyl-4'-heptanoyloxyazobenzene | 82.95 | 109.00 | 108.00 | -25.05 |
| 1665 | 4-Pentylphenyl 4'-tetradecyloxythiobenzoate | 153.26 | 129.60 | 128.20 | 25.06 |
| 1666 | 1-(4-Dodecylbiphenyl-4'-yl)-3-(4-heptyloxyphenyl)propane-1,3-dione | 157.70 | 133.20 | 132.60 | 25.10 |
| 1667 | 1-Methylheptyl 4-(2-fluoro-4-(4-nonyloxybenzoyloxy)phenylethynyl)benzoate | 84.14 | 108.90 | 109.30 | -25.16 |
| 1668 | 4-(1-Methylhepthyloxycarbonyl)phenyl 4'-(3-(perfluorobutanoyloxy)propoxy)biphenyl-4-carboxylate | 66.32 | 91.10 | 91.50 | -25.18 |
| 1669 | 4-(2-(1-Ethylpropoxy)-1-methyl-2-oxoethoxy)phenyl 4'-octyloxybiphenyl-4-carboxylate | 61.11 | 86.10 | 86.30 | -25.19 |
| 1670 | 1-(4-Dodecylbiphenyl-4'-yl)-3-(4-octyloxyphenyl)propane-1,3-dione | 160.80 | 137.30 | 135.60 | 25.20 |
| 1671 | Cholesteryl myristate | 141.21 | 115.40 | 116.00 | 25.21 |
| 1672 | Bis(4-(3-nonyloxy-3-oxo-1-propenyl)phenyl terephthalate | 157.34 | 136.10 | 132.10 | 25.24 |
| 1673 | 5-(2-Bromophenyl)-3-(4-(4-pentylcyclohexyl)phenyl)-1,2,4-oxadiazole | 92.88 | 69.60 | 67.60 | 25.28 |
| 1674 | 1,2-Dimethylpropyl 4-(4-(4-chlorobenzoyloxy)benzylideneamino)benzoate | 87.78 | 61.80 | 62.50 | 25.28 |
| 1675 | 1,3-Phenylene bis(4-(4'-pentylbiphenyl-4-carbonyloxy)-2-ethylbenzoate) | 124.21 | 99.80 | 98.90 | 25.31 |
| 1676 | 4-(1-Methylhepthyloxycarbonyl)phenyl 4'-(3-(butanoyloxy)propoxy)biphenyl-4-carboxylate | 78.05 | 103.40 | 103.40 | -25.35 |
| 1677 | 1-(4-Dodecylbiphenyl-4'-yl)-3-(2-nonyloxyphenyl)propane-1,3-dione | 115.24 | 141.30 | 140.60 | -25.36 |
| 1678 | 1,10-Bis(4-(4-pentyloxybenzoyloxybenzylideneamino)decane | 134.12 | 157.40 | 159.50 | -25.38 |
| 1679 | 2-Acetyl-1,4-phenylene bis(4-pentyloxybenzoate) | 82.31 | 105.40 | 107.70 | -25.39 |
| 1680 | Bis(4-ethoxyphenyl) 2-(7-(4-(4-biphenylazo)phenoxy)heptyloxy) terephthalate | 87.21 | 111.10 | 112.60 | -25.39 |
| 1681 | 4-(4-Tetradecyloxyphenylazoxy)phenyl 2-chloro-3-methylpentanoate | 144.70 | 119.30 | 119.30 | 25.40 |
| 1682 | 4-(2-Methylbutoxycarbonyl)phenyl 4-(4-nonyloxy-2,3,4,5-tetrafluorophenylethynyl)benzoate | 80.60 | 105.80 | 106.00 | -25.40 |
| 1683 | Bis(4-octyloxyphenyl) 2,2'-bipyridine-5,5'-dicarboxylate | 119.71 | 94.80 | 94.30 | 25.41 |
| 1684 | Bis(4-ethoxyphenyl) 2-(6-(4-(4-biphenylazo)phenoxy)hexyloxy) terephthalate | 131.95 | 107.10 | 106.50 | 25.45 |
| 1685 | 4-(2-Chloro-3-methylbutanoyloxy)biphenyl-4'-yl 4-decyloxy-2,3-difluorobenzoate | 74.22 | 97.60 | 99.70 | -25.48 |
| 1686 | Isopropyl 4-(4-(4-methoxybenzylideneamino)benzoyloxy)benzoate | 93.78 | 68.40 | 68.30 | 25.48 |
| 1687 | N-(4-(5-oxo-4-hexadecanoylamino-1,3,6-cycloheptatrien-1-yl)phenyl)hexaadecamide | 119.81 | 141.90 | 145.30 | -25.49 |
| 1688 | 1-(4-Dodecylbiphenyl-4'-yl)-3-(2-heptyloxyphenyl)propane-1,3-dione | 157.70 | 133.20 | 132.20 | 25.50 |
| 1689 | 1,7-Bis(4'-pentylazobenzene-4-oxy)heptane | 109.00 | 134.20 | 134.50 | -25.50 |
| 1690 | 1,4-Bis(4-octyloxyazobenzene-4'-carbonyloxy)butane | 128.80 | 154.10 | 154.30 | -25.50 |
| 1691 | N,N'-Didecanoyl-2,3,5-trimethylbenzene-1,4-diamine | 111.72 | 86.10 | 86.20 | 25.52 |
| 1692 | 4,6-Dichloro-1,3-phenylene bis(4-(4-dodecyloxyphenyliminomethyl)benzoate) | 129.76 | 156.80 | 155.30 | -25.54 |
| 1693 | 2,5-Dihydro-2,5-dimethyl-3,6-bis(4-hexyloxybiphenyl-4'-yl)pyrrolo[3,4-c]pyrrole-1,4-dione | 94.61 | 114.60 | 120.20 | -25.59 |
| 1694 | 1-(4'-Propylazobenzene-4-oxy)-6-(azobenzene-4-oxy)hexane | 137.10 | 113.60 | 111.50 | 25.60 |
| 1695 | 4-Pentanoyloxy-3-methyl-4'-ethoxyazobenzene | 116.83 | 91.60 | 91.20 | 25.63 |
| 1696 | Decyl 4-(4-(4-(1-oxohexyl)-1-piperazinyl)phenylazo)benzoate | 89.83 | 66.30 | 64.20 | 25.63 |
| 1697 | 4-Decanoyloxy-2,3-dimethyl-4'-ethoxyazobenzene | 130.65 | 104.20 | 105.00 | 25.65 |
| 1698 | 2-Chloro-3-methylpentyl 4-(3-(4-undecyloxyphenyl)-1-oxo-2-propenyloxy)benzoate | 126.40 | 100.50 | 100.70 | 25.70 |
| 1699 | 4-Propanoyl-4'-octadecanoyloxyazobenzene | 178.51 | 153.40 | 152.80 | 25.71 |
| 1700 | Phenyl 4-(4-decyloxybenzylideneamino)benzoate | 143.45 | 117.70 | 117.70 | 25.75 |
| 1701 | 4-Hexadecyloxy-3-nitrobenzoic acid | 162.17 | 137.80 | 136.40 | 25.77 |
| 1702 | 3-Methylpentyl 4-(4-(4-methoxybenzoyloxy)benzylideneamino)benzoate | 111.22 | 86.60 | 85.40 | 25.82 |
| 1703 | 1,3-Phenylene bis(4-(3-chloro-4-decyloxyphenylethenyl)benzoate) | 87.18 | 112.90 | 113.00 | -25.82 |
| 1704 | 4-Nonanoyloxy-2,3-dimethyl-4'-ethoxyazobenzene | 126.88 | 100.20 | 101.00 | 25.88 |
| 1705 | 3-(4-Decyloxyphenyl)-5-(4-cyanophenyl)pyrazole | 88.72 | 107.30 | 114.60 | -25.88 |
| 1706 | 1,4-Bis(4-(4-hexyloxybenzoyloxy)benzylideneamino)butane | 165.60 | 141.30 | 139.70 | 25.90 |
| 1707 | 2,5-Dihydro-2,5-dimethyl-3,6-bis(4-(4-pentylcyclohexyl)biphenyl-4'-yl)pyrrolo[3,4-c]pyrrole-1,4-dione | 60.50 | 83.50 | 86.40 | -25.90 |
| 1708 | 4-(3-Ethylmercapto-2-methylpropionyloxy)phenyl 4'-nonyloxybiphenyl-4-carboxylate | 126.75 | 101.40 | 100.80 | 25.95 |
| 1709 | Bis(4-(5-nonylthio-1,3,4-oxadiazole-2-yl)phenyl) terephthalate | 129.68 | 105.90 | 103.70 | 25.98 |
| 1710 | 4-(4-(4-Hexyloxybenzoyloxy)benzoyloxyphenyl)-6'-phenyl-2,2'-bipyridine | 52.08 | 28.10 | 26.10 | 25.98 |
| 1711 | 4'-(2-(4-Hexylphenyl)-1-ethynyl)-2-methyl-4-propyltolane | 41.91 | 66.80 | 67.90 | -25.99 |
| 1712 | 4-Tridecyloxy-3-nitrobenzoic acid | 98.48 | 125.70 | 124.50 | -26.02 |
| 1713 | 4-(4-Hexadecyloxyphenylazoxy)phenyl 2-bromo-3-methylpentanoate | 127.88 | 147.30 | 153.90 | -26.02 |
| 1714 | 4-Octyloxyphenyl 4'-nitrobenzoate | 74.38 | 99.10 | 100.40 | -26.02 |
| 1715 | 3,6,7,10,11-Pentabutoxy-2-triphenylenyl 2-chloro-3-methylpentanoate | 46.86 | 68.40 | 72.90 | -26.04 |
| 1716 | 4-Cyanophenyl 4-(3-(4-(4'-hexylbiphenyl-4-carbonyloxy)-3-fluorobenzoyloxy)benzoyloxy)benzoate | 126.05 | 100.90 | 99.90 | 26.15 |
| 1717 | 1,3-Phenylene bis(4-(4'-hexadecylbiphenyl-4-carbonyloxy)benzoate) | 171.93 | 195.80 | 198.10 | -26.17 |
| 1718 | 1,7-Bis(4-(4-hexyloxybenzoyloxy)benzylideneamino)heptane | 128.11 | 153.40 | 154.30 | -26.19 |
| 1719 | 1-(2-(5-Heptylthienyl))-3-(4-chlorophenylamino)-2-propen-1-one | 87.50 | 62.10 | 61.30 | 26.20 |
| 1720 | 4-(2-Methylbutoxycarbonyl)phenyl 4-(4-octyloxy-2,3,4,5-tetrafluorophenylethynyl)benzoate | 75.98 | 101.80 | 102.20 | -26.22 |
| 1721 | 4-(4-Ethoxyphenylazo)-2-methylphenyl 4-tetradecyloxybenzoate | 100.93 | 127.50 | 127.20 | -26.27 |
| 1722 | 4-Isothiocyanatophenyl 4-(4-heptylcyclohexyl)benzoate | 77.62 | 104.40 | 103.90 | -26.28 |
| 1723 | N,N'-Diundecanoyl-2,3,5,6-tetrakis(undecanoyloxy)-1,4-benzenediamine | 249.30 | 275.50 | 275.60 | -26.30 |
| 1724 | 4-Pentanoyl-4'-nonanoyloxyazobenzene | 98.57 | 125.10 | 124.90 | -26.33 |
| 1725 | 1-(4-Propylcyclohexylethynyl)-4-(4-pentylphenylethynyl)benzene | 40.16 | 65.70 | 66.50 | -26.34 |
| 1726 | 2-Chloro-4-methylpentyl 4-(3-(4-undecyloxyphenyl)-1-oxo-2-propenyloxy)benzoate | 127.22 | 100.50 | 100.80 | 26.42 |
| 1727 | 1,8-Bis(4-nitroazobenzene-4'-oxy)octane | 124.85 | 104.60 | 98.40 | 26.45 |
| 1728 | N,N'-Ditetradecanoyl-3,4,5,6-tetramethylbenzene-1,2-diamine | 89.85 | 110.80 | 116.30 | -26.45 |
| 1729 | 4-Methylpentyl 4-(4-(4-methoxybenzylideneamino)benzoyloxy)benzoate | 111.90 | 86.60 | 85.40 | 26.50 |
| 1730 | 4-(3-Decyloxyphenyliminomethyl)phenyl 4-nonyloxybenzoate | 169.61 | 143.60 | 143.10 | 26.51 |
| 1731 | 2-Methyl-4-octyloxyphenyl 4-octyloxythiobenzoate | 137.62 | 111.00 | 111.10 | 26.52 |
| 1732 | N,N'-Ditetradecanoyl-2,6-dimethylbenzene-1,4-diamine | 152.14 | 126.00 | 125.60 | 26.54 |
| 1733 | 1-(4-Hexylphenylethynyl)-4-(4-propylphenylethynyl)benzene | 46.75 | 74.40 | 73.40 | -26.65 |
| 1734 | 3-Methylbutyl 4-(4-(4-methoxybenzoyloxy)benzylideneamino)benzoate | 108.46 | 82.60 | 81.80 | 26.66 |
| 1735 | 4-(3-(4-(Heptyloxy)phenyl)-1-oxo-2-propenyloxy)phenyl 2-chloro-4-methylpentanoate | 113.96 | 87.30 | 87.30 | 26.66 |
| 1736 | Octakis(octanoyloxy)-9,10-anthraquinone | 304.86 | 274.50 | 278.20 | 26.66 |
| 1737 | 4-(3,4-Bis(4-heptyloxybenzoyloxy)benzylideneamino)azobenzene | 160.18 | 134.50 | 133.50 | 26.68 |
| 1738 | 4-(1-Methylheptyloxycarbonyl)phenyl 4-(4-undecyloxybenzoylthio)benzoate | 146.89 | 120.00 | 120.20 | 26.69 |
| 1739 | 4-(3,4-Bis(4-decyloxybenzoyloxy)benzylideneamino)azobenzene | 185.22 | 158.80 | 158.50 | 26.72 |
| 1740 | 2,3,6,7-Tetrakis(nonyloxy)-1,5-bis(2-(tetrahydropyran-2-yl)ethoxy)-9,10-anthraquinone | 199.83 | 174.20 | 173.00 | 26.83 |
| 1741 | 4,4'''-Dihexyloxy-p-quaterphenyl | 64.97 | 90.80 | 91.80 | -26.83 |
| 1742 | 1,5-Bis(4-(2-hydroxy-4-decyloxybenzylideneamino)benzoyloxy)-2,2-dimethylpentane | 129.76 | 155.10 | 156.60 | -26.84 |
| 1743 | 4-Isothiocyanatophenyl 4-(4-dodecylcyclohexyl)benzoate | 150.86 | 124.50 | 124.00 | 26.86 |
| 1744 | 4'-(2-(4-Propylphenyl)-1-ethynyl)-2'-ethyl-4-pentyltolane | 41.04 | 66.80 | 67.90 | -26.86 |
| 1745 | 4-(4-Ethoxyphenylazo)-2,3,6-trimethylphenyl 4-dodecyloxybenzoate | 129.77 | 104.20 | 102.90 | 26.87 |
| 1746 | 1,2-Bis-5-(4-(5-undecyl-1,3,4-thiadiazol-2-yl)phenoxy)pentanoyloxy)-6-(4'-cyanobiphenyl-4-yloxy)hexane | 221.37 | 202.00 | 194.50 | 26.87 |
| 1747 | Bis(4-(5-decylthio-1,3,4-oxadiazole-2-yl)phenyl)-2,5-thiophenedicarboxylate | 93.31 | 117.50 | 120.20 | -26.89 |
| 1748 | 6-Pentyloxy-2-(4-ethoxystyryl)quinoline | 105.21 | 77.50 | 78.30 | 26.91 |
| 1749 | Bis(4-(5-heptylthio-1,3,4-oxadiazole-2-yl)phenyl) terephthalate | 65.42 | 89.70 | 92.40 | -26.98 |
| 1750 | 1,3-Bis(4-(2-hydroxy-4-heptyloxybenzylideneamino)benzoyloxy)benzene | 87.91 | 112.90 | 114.90 | -26.99 |
| 1751 | 1,5-Bis(4-ethoxyazobenzene-4'-carbonyloxy)pentane | 81.30 | 109.70 | 108.30 | -27.00 |
| 1752 | 1-Methylpropyl 4-(4-(4-methoxybenzoyloxy)benzylideneamino)benzoate | 99.23 | 72.50 | 72.20 | 27.03 |
| 1753 | 1-(2-(5-Octylthienyl))-3-(4-bromophenylamino)-2-propen-1-one | 96.55 | 72.50 | 69.50 | 27.05 |
| 1754 | 2-Fluoro-4-octyloxyphenyl 4-octyloxythiobenzoate | 146.08 | 119.60 | 119.00 | 27.08 |
| 1755 | 4-(2-Chloro-3-methylbutanoyloxy)biphenyl-4'-yl 4-dodecyloxy-2,3-difluorobenzoate | 82.80 | 105.70 | 109.90 | -27.10 |
| 1756 | 1,8-Bis(4-(4-propoxybenzoyloxybenzylideneamino)octane | 103.58 | 133.20 | 130.70 | -27.12 |
| 1757 | 4,4'-Dipentanoyloxydiphenyldiacetylene | 70.57 | 97.90 | 97.70 | -27.13 |
| 1758 | 4-Decyloxyphenyl 4-nitrobenzoate | 81.35 | 107.20 | 108.50 | -27.15 |
| 1759 | Ethyl 4-(4-(4-octyloxy-2,3,5,6-tetrafluorophenylethynyl)benzyloxy)benzoate | 123.06 | 96.60 | 95.80 | 27.26 |
| 1760 | 4-Pentyl-4'-hexadecyloxyazobenzene | 120.34 | 149.00 | 147.60 | -27.26 |
| 1761 | Bis(4-pentyloxyphenyl) 4,4'-biphenyldicarboxylate | 132.17 | 104.50 | 104.90 | 27.27 |
| 1762 | Hexa(4-pentyloxyphenoxymethyl)benzene | 164.08 | 135.80 | 136.80 | 27.28 |
| 1763 | Bis(4-(3-methylbutoxycarbonyl)phenyl) terephthalate | 120.28 | 90.50 | 93.00 | 27.28 |
| 1764 | Bis(4-hexyloxyphenyl) 2,2'-bithiophene-5,5'-dicarboxylate | 93.52 | 119.60 | 120.80 | -27.28 |
| 1765 | 4,4'-Bis(4-(4-heptyloxybenzoyloxy)benzylideneamino)diphenylamine | 87.20 | 112.40 | 114.50 | -27.30 |
| 1766 | 4-Propanoyl-4'-heptadecanoyloxyazobenzene | 175.94 | 149.40 | 148.60 | 27.34 |
| 1767 | 1-(4'-Butylazobenzene-4-oxy)-6-(4'-ethylazobenzene-4-oxy)hexane | 86.93 | 114.00 | 114.30 | -27.37 |
| 1768 | 2-Acetyl-1,4-phenylene bis(4-nonyloxybenzoate) | 110.81 | 137.70 | 138.20 | -27.39 |
| 1769 | 4-Cyanophenyl 4-hexylbenzoate | 118.31 | 89.90 | 90.90 | 27.41 |
| 1770 | Cholesteryl 6-(4-(4-(2-chloro-3-methylpentoyloxy)phenyliminomethyl)phenoxy)hexanoate | 54.99 | 82.30 | 82.40 | -27.41 |
| 1771 | 2,5-Bis(4-octyloxyphenylethynyl)-3,4-thiophenedicarbonitrile | 123.72 | 97.50 | 96.30 | 27.42 |
| 1772 | N-(4-Decyloxyphenyl)-6-decyloxyquinoline-2-carboxamide | 152.93 | 125.60 | 125.50 | 27.43 |
| 1773 | 1-(2-(5-Nonylthienyl))-3-(4-chlorophenylamino)-2-propen-1-one | 98.47 | 70.10 | 71.00 | 27.47 |
| 1774 | 3-(4-(4-Heptylcyclohexyl)phenyl)-5-(4-nonyloxyphenyl)isoxazole | 72.02 | 96.50 | 99.50 | -27.48 |
| 1775 | 4-Pentyl-4'-methoxyazobenzene | 61.87 | 89.70 | 89.40 | -27.53 |
| 1776 | 4-Pentyl-4''-cyano-p-terphenyl | 43.35 | 69.30 | 70.90 | -27.55 |
| 1777 | 2-Fluoro-4-octyloxyphenyl 4-cyanothiobenzoate | 117.59 | 89.60 | 90.00 | 27.59 |
| 1778 | N,N'-Dipentanoyl-2,3,5,6-tetrakis(pentanoyloxy)-1,4-benzenediamine | 157.40 | 130.10 | 129.80 | 27.60 |
| 1779 | 1,3-Phenylene bis(4-(4-tetradecyloxyphenyliminomethyl)benzoate) | 160.09 | 187.40 | 187.70 | -27.61 |
| 1780 | Ethyl 4-(4-(4-methoxybenzoyloxy)benzylideneamino)benzoate | 110.74 | 83.10 | 83.10 | 27.64 |
| 1781 | 2,5-Thiophenediylbis(ethynediyl-4,1-phenylene) 3,4-bis(octyloxy)benzoate | 190.15 | 162.70 | 162.50 | 27.65 |
| 1782 | 4-Butoxy-2'-(4-methacryloyloxybutoxy)-4'-(4-butoxybenzoyloxy)azobenzene | 142.66 | 118.00 | 115.00 | 27.66 |
| 1783 | 4'-(2-(4-Ethylphenyl)-1-ethynyl)-2'-ethyl-4-pentyltolane | 38.63 | 62.80 | 66.30 | -27.67 |
| 1784 | 4-(2,2,3,3,4,4,4-Heptafluorobutyloxycarbonyl)phenyl 4-(perfluorohexylhexyloxy)benzoate | 136.41 | 109.10 | 108.70 | 27.71 |
| 1785 | 4-Pentanoyl-4'-hexadecanoyloxyazobenzene | 179.91 | 153.40 | 152.10 | 27.81 |
| 1786 | N,N',N''-Triundecanoyl-2,4,6-trimethyl-1,3,5-benzenetriamine | 79.78 | 112.50 | 107.60 | -27.82 |
| 1787 | 4-(2-Methylbutoxy)phenyl 4-(4-nonyloxy-2,3,5,6-tetrafluorophenylethynyl)benzoate | 76.52 | 103.10 | 104.40 | -27.88 |
| 1788 | 2,5-Bis(4-decyloxyphenylethynyl)-3,4-thiophenedicarbonitrile | 134.54 | 113.70 | 106.60 | 27.94 |
| 1789 | 1-Methylheptyl 4'-(4''-hexadecyloxy- 3''-fluorobenzoyloxy)tolan-4-carboxylate | 165.15 | 137.10 | 137.20 | 27.95 |
| 1790 | Bis(4-Heptyloxyphenyl) 2,5-thiophenedicarboxylate | 152.56 | 129.90 | 124.60 | 27.96 |
| 1791 | 4-Cyanophenyl 4-(3-(4-(4'-nonylbiphenyl-4-carbonyloxy)-3-fluorobenzoyloxy)benzoyloxy)benzoate | 84.81 | 113.00 | 112.80 | -27.99 |
| 1792 | 1,8-Bis(4-butoxyazobenzene-4'-carbonyloxy)octane | 167.00 | 138.00 | 139.00 | 28.00 |
| 1793 | 1-(4-Fluorophenyl)-4-((4-penten-1-yl)phenyl)-1,3-butadiyne | 56.30 | 79.10 | 84.30 | -28.00 |
| 1794 | 4'-(Propyl-(1,1')-bicyclohexyl-4-carbonitrile | 86.73 | 57.50 | 58.70 | 28.03 |
| 1795 | 4-(1-Methylheptyloxycarbonyl)phenyl 4-(4-undecyloxyphenylethyl)benzoate | 156.29 | 127.80 | 128.20 | 28.09 |
| 1796 | 4-Cyano-1,3-phenylene bis(4-(4-nonyloxyphenyliminomethyl)benzoate) | 164.89 | 138.80 | 136.80 | 28.09 |
| 1797 | 1,3-Bis(4-(4-octyloxybenzoyloxy)benzylideneamino)benzene | 167.42 | 138.90 | 139.30 | 28.12 |
| 1798 | N-(4-Hexadecyloxybenzylidene)-4-hexadecylaniline | 162.28 | 189.70 | 190.40 | -28.12 |
| 1799 | N-(4-Nonyloxyphenyl)-6-decyloxyquinoline-2-carboxamide | 148.65 | 121.50 | 120.50 | 28.15 |
| 1800 | 4-(4-Ethoxybenzoyloxy)-2-ethoxy-4'-(4-butyoxysalicylaldimino)azobenzene | 112.08 | 85.60 | 83.90 | 28.18 |
| 1801 | 4-Decanoyloxy-2-methyl-4'-ethoxyazobenzene | 140.20 | 111.80 | 112.00 | 28.20 |
| 1802 | 2-Heptanoyl-1,4-phenylene bis(4-octyloxybenzoate) | 180.11 | 149.80 | 151.90 | 28.21 |
| 1803 | 2,3,6,7-Tetrakis(octyloxy)-1,5-bis(2-(tetrahydropyran-2-yl)ethoxy)-9,10-anthraquinone | 130.86 | 158.00 | 159.10 | -28.24 |
| 1804 | 1,5-Bis(4-(2-hydroxy-4-hexyloxybenzylideneamino)benzoyloxy)-2,2-dimethylpentane | 94.23 | 122.80 | 122.50 | -28.27 |
| 1805 | 1-(6-Octyloxy-2-naphthyl)-2-(3,4-difluorophenyl)acetylene | 113.50 | 84.70 | 85.20 | 28.30 |
| 1806 | N,N'-Dioctanoyl-2,3,5-trimethylbenzene-1,4-diamine | 97.60 | 69.90 | 69.30 | 28.30 |
| 1807 | 4-Octyloxyphenyl 4'-pentylbenzoate | 89.09 | 115.90 | 117.40 | -28.31 |
| 1808 | 1,3-Bis(4-(4-octyloxybenzoyloxy)benzylideneamino)propane | 179.53 | 153.40 | 151.20 | 28.33 |
| 1809 | 4-(3-(4-(Heptyloxy)phenyl)-1-oxo-2-propenyloxy)phenyl 2-chloro-3-methylpentanoate | 113.25 | 87.30 | 84.90 | 28.35 |
| 1810 | 1,9-Bis(4-(4-hexyloxybenzoyloxy)benzylideneamino)nonane | 135.19 | 161.50 | 163.60 | -28.41 |
| 1811 | N-(4-Methoxyphenyl)-alpha-(4-hexadecyloxyphenyl)nitrone | 182.42 | 156.80 | 154.00 | 28.42 |
| 1812 | 4-Cyano-4'-undecylbiphenyl | 127.05 | 99.20 | 98.60 | 28.45 |
| 1813 | 1-(4-Pentyloxyphenylethynyl)-4-(4-butylphenylethynyl)benzene | 47.34 | 75.60 | 75.80 | -28.46 |
| 1814 | (3,4-Dicyano-2,5-thiophenediyl) bis(ethynediyl-4,1-phenylene) 3,4-bis(undecyloxy)benzoate | 171.00 | 200.10 | 199.50 | -28.50 |
| 1815 | 4-Heptyl-4'-heptyloxyazobenzene | 92.10 | 120.70 | 120.60 | -28.50 |
| 1816 | 1,3-Dimethylbutyl 4-(4-(4-methoxybenzylideneamino)benzoyloxy)benzoate | 95.71 | 68.00 | 67.20 | 28.51 |
| 1817 | 1,2,18,19-Tetrahydroxynonadecane | 192.12 | 164.20 | 163.60 | 28.52 |
| 1818 | 1-(2-(5-Chlorothienyl))-3-(4-dodecyloxyphenylamino)-2-propen-1-one | 124.03 | 98.60 | 95.50 | 28.53 |
| 1819 | 1-(4-(N-Ethoxycarbonylpiperazinyl)phenyl)-4-(4-decyloxyphenyl)but-3-en-1-yne | 42.29 | 66.50 | 70.90 | -28.61 |
| 1820 | 3,4-Didodecyloxybenzaldehyde-2',4'-dinitrophenyl hydrazone | 139.52 | 112.20 | 110.90 | 28.62 |
| 1821 | 4-Dodecyloxybiphenyl-4'-yl 4-(1-methylheptyloxy)thiobenzoate | 82.75 | 110.50 | 111.40 | -28.65 |
| 1822 | N-(4-(5-oxo-4-tetradecanoylamino-1,3,6-cycloheptatrien-1-yl)phenyl)tetradecamide | 103.03 | 125.70 | 131.70 | -28.67 |
| 1823 | Cholesteryl 5-(2,3-difluoro-4-octyloxybiphenyl-4'-oxy)pentanoate | 132.81 | 104.30 | 104.10 | 28.71 |
| 1824 | 4-Decyl-4'-thiocyanatobiphenyl | 119.42 | 92.80 | 90.70 | 28.72 |
| 1825 | m-Terphenyl-4,4''-diyl bis(4-(5-hexylpyrimidin-2-yl)benzoate) | 98.49 | 73.60 | 69.70 | 28.79 |
| 1826 | 4-Thiocyanophenyl 4-heptyloxybiphenyl-4'-carboxylate | 114.62 | 87.10 | 85.80 | 28.82 |
| 1827 | 4-Butyl-4'-tetradecyloxyazobenzene | 108.78 | 136.80 | 137.60 | -28.82 |
| 1828 | 4-(3-Ethylmercapto-2-methylpropionyloxy)phenyl 4'-hexadecyloxybiphenyl-4-carboxylate | 105.58 | 129.70 | 134.40 | -28.82 |
| 1829 | 1,3-Phenylene bis(4-(4-nonylthiophenyliminomethyl)benzoate) | 121.34 | 109.20 | 92.50 | 28.84 |
| 1830 | N-(2-(6-Decyloxy)benzothiazolyl)-4-heptyloxybenzamide | 123.47 | 96.80 | 94.60 | 28.87 |
| 1831 | Bis(4-(4-decyloxyphenyliminomethyl)phenyl) hexanedioate | 147.80 | 176.90 | 176.70 | -28.90 |
| 1832 | 4-(3-Pyridinylmethyleneamino)phenyl 4-heptyloxybenzoate | 85.59 | 114.00 | 114.50 | -28.91 |
| 1833 | 4-(2-Chloro-3-methylbutanoyloxy)biphenyl-4'-yl 4-heptyloxy-2,3-difluorobenzoate | 57.39 | 85.50 | 86.30 | -28.91 |
| 1834 | 5-Methoxy-2-(4-n-tridecafluorohexylphenyl)isoindole-1,3-dione | 109.42 | 80.90 | 80.50 | 28.92 |
| 1835 | 4,5-Dichloro-1,3-phenylene bis(4-(4-methoxybenzylideneamino)benzoate) | 98.63 | 70.60 | 69.70 | 28.93 |
| 1836 | 4-Ethoxyphenyl 3-fluoro-4-thiocyanatophenyl terephthalate | 48.47 | 78.80 | 77.40 | -28.93 |
| 1837 | 1,3-Bis(4-(4-nonyloxybenzoyloxy)benzylideneamino)benzene | 175.97 | 147.00 | 147.00 | 28.97 |
| 1838 | 1,3-Phenylene bis(4-(4-(5-hexenyloxy)phenyliminomethyl)benzoate) | 155.18 | 127.70 | 126.20 | 28.98 |
| 1839 | 4-Cyanophenyl 4-(3-(4-(4'-hexylbiphenyl-4-carbonyloxy)-2-fluorobenzoyloxy)benzoyloxy)benzoate | 129.71 | 100.90 | 100.70 | 29.01 |
| 1840 | 4-Butyl-4'-hexyloxyazobenzene | 74.69 | 104.50 | 103.70 | -29.01 |
| 1841 | 3-(4-(4-Cyanophenoxycarbonyl)phenoxycarbonyl)phenyl 3-fluoro-4-(4-octadecyloxybenzoyloxy)benzoate | 128.29 | 156.20 | 157.30 | -29.01 |
| 1842 | 4-Pentyl-N-(4-pentadecyloxyphenylmethylene)benzenamine | 169.12 | 141.20 | 140.10 | 29.02 |
| 1843 | 4-Butyl-4'-methoxyazobenzene | 56.15 | 85.70 | 85.20 | -29.05 |
| 1844 | 4-Cyanophenyl 4-(3-(4-(4'-dodecylbiphenyl-4-carbonyloxy)-3-fluorobenzoyloxy)benzoyloxy)benzoate | 97.65 | 125.10 | 126.70 | -29.05 |
| 1845 | (3,4-Dicyano-2,5-thiophenediyl) bis(ethynediyl-4,1-phenylene) 3,4-bis(dodecyloxy)benzoate | 244.26 | 216.30 | 215.20 | 29.06 |
| 1846 | N-(4-Octyloxyphenyl)-6-decyloxyquinoline-2-carboxamide | 146.85 | 117.50 | 117.70 | 29.15 |
| 1847 | 4'-(4-Butylcyclohexylethyl)-4-isothiocyanatotolane | 116.38 | 85.60 | 87.20 | 29.18 |
| 1848 | 3-(4-Decyloxyphenyl)-5-(4-methoxyphenyl)isoxazole | 118.01 | 89.20 | 88.80 | 29.21 |
| 1849 | 4-(2-(4-Pentylcyclohexyl)ethyl)-4'-(2-(4-propylcyclohexyl)ethyl)biphenyl | 49.69 | 79.10 | 78.90 | -29.21 |
| 1850 | Pentyl 4-(4-(4-pentyloxy-2,3,5,6-tetrafluorophenylethynyl)benzyloxy)benzoate | 67.47 | 96.60 | 96.70 | -29.23 |
| 1851 | 1-(4'-Ethylazobenzene-4-oxy)-6-(azobenzene-4-oxy)hexane | 136.95 | 109.50 | 107.70 | 29.25 |
| 1852 | Nonyl 2,5-bis(4-octyloxybenzoyloxy)benzoate | 133.82 | 163.00 | 163.10 | -29.28 |
| 1853 | N,N'-Didodecanoyl-2,3,5-trimethylbenzene-1,4-diamine | 130.64 | 102.20 | 101.30 | 29.34 |
| 1854 | 4-(2-Methylbutoxycarbonyl)phenyl 4-(4-heptyloxy-2,3,4,5-tetrafluorophenylethynyl)benzoate | 68.16 | 97.80 | 97.50 | -29.34 |
| 1855 | 2-(4-Pentyloxyphenyl)-5-(4-decyloxy)phenylazo-1,3,4-thiadiazole | 121.09 | 90.60 | 91.70 | 29.39 |
| 1856 | 4-Cyano-4'-tetradecylbiphenyl | 140.90 | 111.30 | 111.50 | 29.40 |
| 1857 | 1,3-Phenylene bis(4-(3-nitro-4-dodecyloxyphenylethenyl)benzoate) | 103.99 | 127.00 | 133.40 | -29.41 |
| 1858 | Bis(4-Pentyloxyphenyl) 2,5-thiophenedicarboxylate | 144.62 | 113.70 | 115.20 | 29.42 |
| 1859 | 1-(4-Undecylbiphenyl-4'-yl)-3-(4-cyanobiphenyl)propane-1,3-dione | 70.04 | 97.50 | 99.50 | -29.46 |
| 1860 | 1,3-Phenylene bis(4-(4'-octadecylbiphenyl-4-carbonyloxy)benzoate) | 182.80 | 212.00 | 212.30 | -29.50 |
| 1861 | 1,5-Bis(4'-pentylazobenzene-4-oxy)pentane | 98.70 | 126.10 | 128.20 | -29.50 |
| 1862 | Bis(4-(5-nonylthio-1,3,4-oxadiazole-2-yl)phenyl)-2,5-thiophenedicarboxylate | 82.46 | 109.40 | 112.00 | -29.54 |
| 1863 | 1-(6-Pentyloxy-2-naphthyl)-4-(4-fluorophenyl)diacetylene | 98.96 | 67.70 | 69.40 | 29.56 |
| 1864 | 1,2-Bis(4-n-decyloxybenzoyl)hydrazine | 146.88 | 117.30 |  | 29.58 |
| 1865 | 4-t-Butylphenyl 4-(4-decyloxybenzylideneamino)benzoate | 86.81 | 120.20 | 116.40 | -29.59 |
| 1866 | 4-Pentanoyl-4'-hexanoyloxyazobenzene | 82.71 | 113.00 | 112.30 | -29.59 |
| 1867 | 2-Cyano-1,3-phenylene bis(4-(4'-nonylbiphenyl-4-carbonyloxy)-3-fluorobenzoate) | 162.22 | 133.00 | 132.60 | 29.62 |
| 1868 | 1,10-Bis(4-(4-hexyloxybenzoyloxybenzylideneamino)decane | 135.77 | 165.50 | 165.40 | -29.63 |
| 1869 | 1-Methylheptyl 4'-(4''-dodecyloxy-3''-fluorobenzoyloxy)tolan-4-carboxylate | 91.67 | 121.00 | 121.30 | -29.63 |
| 1870 | N,N'-Dioctanoyl-2,5,6-trimethyl-4-octanoyloxy-1,3-benzenediamine | 71.83 | 97.40 | 101.50 | -29.67 |
| 1871 | 1-(4-Penty)phenyl)-4-(4-butylaminophenyl)-1,3-butadiyne | 67.23 | 88.90 | 96.90 | -29.67 |
| 1872 | 4-(4-Decyloxybenzylideneamino)phenyl benzoate | 147.82 | 117.70 | 118.10 | 29.72 |
| 1873 | m-Terphenyl-4,4''-diyl bis(4-(4-nonyloxybenzoyloxy)benzoate) | 113.87 | 141.60 | 143.60 | -29.73 |
| 1874 | 1,7-Bis(4-(4-pentyloxybenzoyloxy)benzylideneamino)heptane | 116.31 | 145.30 | 146.10 | -29.79 |
| 1875 | 4-Heptyl-4'-methoxyazobenzene | 67.90 | 97.80 | 97.70 | -29.80 |
| 1876 | 4-Octanoyloxy-3-methyl-4'-ethoxyazobenzene | 134.81 | 103.80 | 105.00 | 29.81 |
| 1877 | Pentyl 4-(4-(4-methoxybenzoyloxy)benzylideneamino)benzoate | 125.35 | 95.20 | 95.50 | 29.85 |
| 1878 | 4-(4-Heptyloxy-2,3,5,6-tetrafluorophenylacetylenyl)phenyl 4'-chlorobenzoate | 119.71 | 90.70 | 89.80 | 29.91 |
| 1879 | 1,3-Phenylene bis(4-(4'-hexadecylbiphenyl-4-carbonyloxy)-3-fluorobenzoate) | 166.84 | 197.80 | 196.80 | -29.96 |
| 1880 | 4-Cyanophenyl 4-(3-(4-(4'-octadecylbiphenyl-4-carbonyloxy)-3-fluorobenzoyloxy)benzoyloxy)benzoate | 119.35 | 149.40 | 149.40 | -30.05 |
| 1881 | 3-(4-Decyloxyphenyl)-5-(4-chlorophenyl)isoxazole | 115.97 | 87.00 | 85.90 | 30.07 |
| 1882 | 2-(4-Butylphenyl)-5-phenylpyridine | 26.03 | 56.50 | 56.10 | -30.07 |
| 1883 | Isopropyl 4-(4-(4-nitrobenzoyloxy)benzylideneamino)benzoate | 94.28 | 65.20 | 64.20 | 30.08 |
| 1884 | 4-Heptyloxyphenyl 4'-pentylbenzoate | 82.89 | 111.90 | 113.00 | -30.11 |
| 1885 | 4-(2-(4-(9-Vinyloxynonyloxy)phenyl)vinyl)benzonitrile | 61.97 | 93.40 | 92.20 | -30.23 |
| 1886 | 6-Decyloxy-2-(4'-nonyloxyphenyliminomethyl)quinoline | 169.35 | 139.80 | 139.10 | 30.25 |
| 1887 | 4,4'''-Dibutoxy-p-quaterphenyl | 46.01 | 74.70 | 76.30 | -30.29 |
| 1888 | 1-(4-Dodecylbiphenyl-4'-yl)-3-(2-octyloxyphenyl)propane-1,3-dione | 108.41 | 137.30 | 138.80 | -30.39 |
| 1889 | 1-(4-(N-Ethoxycarbonylpiperazinyl)phenyl)-4-(4-heptyloxyphenyl)but-3-en-1-yne | 80.01 | 54.40 | 49.60 | 30.41 |
| 1890 | 1-(4'-Propylbicyclohexyl)-3,4-difluorobenzene | 87.42 | 62.10 | 57.00 | 30.42 |
| 1891 | 2-(Perfluorohexyl)ethyl 4-phenylthiobenzoate | 120.64 | 92.80 | 90.20 | 30.44 |
| 1892 | 5-(4-Decyloxyphenyl)-2-(4-octyloxy)phenylamido-1,3,4-thiadiazole | 48.44 | 80.70 | 78.90 | -30.46 |
| 1893 | 4-Butylbenzoic acid | 66.07 | 96.40 | 96.60 | -30.53 |
| 1894 | 4-(4-(1-Methylheptyloxycarbonyl)phenyloxycarbonyl)phenyl 4-(6-(4-(4-decyloxybenzoyloxy)benzoyloxy)heptyloxy)benzoate | 189.55 | 159.50 | 159.00 | 30.55 |
| 1895 | 1-(4-Pentylcyclohexylethynyl)-4-(4-propylphenylethynyl)benzene | 35.94 | 65.70 | 66.50 | -30.56 |
| 1896 | 3-Pyridyl 4-(4-decyloxybenzylideneamino)benzoate | 155.76 | 126.20 | 125.10 | 30.66 |
| 1897 | 4-Decyl-N-(4-butoxyphenylmethylene)benzenamine | 148.73 | 117.00 | 118.00 | 30.73 |
| 1898 | 3,5-Dihydroxycyclohexyl 3,4-bis(decyloxy)benzoate | 74.66 | 104.50 | 105.40 | -30.74 |
| 1899 | 4-(4-(2-Ethoxy-1-methyl-2-oxoethoxycarbonyl)phenylethynyl)phenyl 2,3-difluoro-4-tetradecyloxybenzoate | 149.90 | 117.80 | 119.10 | 30.80 |
| 1900 | 1,4-Bis(4-pentylcyclohexylethynyl)benzene | 41.47 | 69.10 | 72.30 | -30.83 |
| 1901 | 4-Pentanoyloxy-2-methyl-4'-ethoxyazobenzene | 121.45 | 91.60 | 90.60 | 30.85 |
| 1902 | 1-(4-Dodecylbiphenyl-4'-yl)-3-(4-cyanobiphenyl)propane-1,3-dione | 70.85 | 101.50 | 101.70 | -30.85 |
| 1903 | 2,3,4-Trichloro-N-(4-(4-methoxybenzoyloxy)benzylidene)aniline | 92.87 | 61.00 | 62.00 | 30.87 |
| 1904 | 4-(3-(4-(Dodecyloxy)phenyl)-1-oxo-2-propenyloxy)phenyl 2-chloro-3-methylpentanoate | 139.69 | 107.50 | 108.80 | 30.89 |
| 1905 | 3,4-Dichloro-N-(4-(4-methylbenzoyloxy)benzylidene)aniline | 97.51 | 65.70 | 66.60 | 30.91 |
| 1906 | 1,3-Phenylene bis(4-(3-chloro-4-undecyloxyphenylethenyl)benzoate) | 88.54 | 121.00 | 119.50 | -30.96 |
| 1907 | 4-Undecyloxyphenyl 4-nitrobenzoate | 142.87 | 111.20 | 111.90 | 30.97 |
| 1908 | 4'-(2-(4-Ethylphenyl)-1-ethynyl)-4-hexyltolane | 39.38 | 70.40 | 70.40 | -31.02 |
| 1909 | 1,9-Bis(4-pentylazobenzene-4'-oxy)nonane | 111.97 | 142.30 | 143.00 | -31.03 |
| 1910 | 2-Ethylbutyl 4-(4-(4-methoxybenzylideneamino)benzoyloxy)benzoate | 56.06 | 86.60 | 87.20 | -31.14 |
| 1911 | 4'-(2-(4-Ethylphenyl)-1-ethynyl)-2'-methyl-4-methyltolane | 74.34 | 42.60 | 43.10 | 31.24 |
| 1912 | (3,4-Dicyano-2,5-thiophenediyl) bis(ethynediyl-4,1-phenylene) 3,4-bis(decyloxy)benzoate | 153.35 | 183.90 | 184.60 | -31.25 |
| 1913 | Cholesteryl 4-(2,3-difluoro-4-octyloxybiphenyl-4'-oxy)butanoate | 133.29 | 100.30 | 102.00 | 31.29 |
| 1914 | 4-(omega-Aminononyloxy)-4'-cyanobiphenyl | 98.29 | 73.20 | 67.00 | 31.29 |
| 1915 | Cholesteryl undecanoate | 72.40 | 103.20 | 103.70 | -31.30 |
| 1916 | Bis(4-methoxycarbonylphenyl) terephthalate | 113.26 | 86.10 | 81.90 | 31.36 |
| 1917 | 1-Methylheptyl 4-(2-fluoro-4-(4-tetradecyloxybenzoyloxy)phenylethynyl)benzoate | 159.67 | 129.10 | 128.30 | 31.37 |
| 1918 | 4-Dodecyloxy-3-nitrobenzoic acid | 90.13 | 121.60 | 121.50 | -31.37 |
| 1919 | 4-Methyl-2'-hydroxy-4'-dodecyloxyazobenzene | 76.70 | 107.70 | 108.10 | -31.40 |
| 1920 | 2,5-Dihydro-2,5-dimethyl-3,6-bis(4-(4-propylcyclohexyl)phenyl)pyrrolo[3,4-c]pyrrole-1,4-dione | 52.88 | 78.70 | 84.30 | -31.42 |
| 1921 | Cholesteryl 5-(4-(4-hexylphenylethynyl)phenoxy)pentanoate | 58.53 | 89.20 | 90.00 | -31.47 |
| 1922 | 4,4'-Di(2-methoxyethoxy)biphenyl | 97.77 | 67.90 | 66.30 | 31.47 |
| 1923 | 4-(4-(4-(4-Dodecyloxybenzoyloxy)benzoyl)-1-piperazinyl)phenyl 4-(4-dodecyloxybenzoyloxy)benzoate | 75.63 | 105.10 | 107.10 | -31.47 |
| 1924 | 4-Nitrophenyl 2,5-bis(4-octyloxybenzoyloxy)benzoate | 101.60 | 131.40 | 133.20 | -31.60 |
| 1925 | 1,6-Bis(4'-pentylazobenzene-4-oxy)hexane | 100.63 | 130.20 | 132.30 | -31.67 |
| 1926 | 1-(4'-Ethylazobenzene-4-oxy)-6-(4'-methylazobenzene-4-oxy)hexane | 131.78 | 101.90 | 100.10 | 31.68 |
| 1927 | 1,5-Bis(4-dodecyloxyazobenzene-4'-carbonyloxy)pentane | 157.51 | 190.50 | 189.20 | -31.69 |
| 1928 | 4-(1-Methylheptyloxycarbonyl)phenyl 4-(4-heptyloxyphenylethyl)benzoate | 143.30 | 111.70 | 111.60 | 31.70 |
| 1929 | 3-(5-(4-(4-Propylcyclohexyl)phenyl)-1,2,4-oxadiazol-3-yl)pyridine | 41.98 | 70.80 | 73.70 | -31.72 |
| 1930 | 1-Methylheptyl 4-(4-(4-tetradecyloxy-3-fluorobenzoyloxy)-3-fluorobenzoyloxy)benzoate | 114.04 | 144.80 | 145.80 | -31.76 |
| 1931 | Cholesteryl pentadecanoate | 151.73 | 119.40 | 119.90 | 31.83 |
| 1932 | 1,3-Phenylene bis(4-(4-hexadecyloxybenzoylthio)benzoate) | 156.22 | 188.30 | 188.10 | -31.88 |
| 1933 | 2-Methyl-1,3-phenylene bis(4-(4-octyloxyphenyliminomethyl)benzoate) | 100.21 | 131.30 | 132.10 | -31.89 |
| 1934 | 4-(4-Ethoxyphenylazo)-2-methylphenyl 4-decyloxybenzoate | 142.92 | 111.40 | 111.00 | 31.92 |
| 1935 | 4-Nitrophenyl 4-hexadecyloxybenzoate | 164.52 | 131.40 | 132.60 | 31.92 |
| 1936 | 1-Methylheptyl 4-(4-(4-tridecyloxy-3-fluorobenzoyloxy)-3-fluorobenzoyloxy)benzoate | 108.09 | 140.70 | 140.10 | -32.01 |
| 1937 | 2,5-Bis(4-methoxyphenyl)-thiazolo[5,4-d]thiazole | 76.93 | 105.60 | 109.00 | -32.07 |
| 1938 | 3-(4-(2-(4-Dodecyloxy-3-nitrophenyl)-1-ethenyl)benzoyloxy)phenyl 4-(2-(3-fluoro-4-dodecyloxyphenyl)-1-ethenyl)benzoate | 103.80 | 136.20 | 135.90 | -32.10 |
| 1939 | 1,3-Phenylene bis(4-(4'-nonylbiphenyl-4-carbonyloxy)benzoate) | 172.33 | 139.20 | 140.20 | 32.13 |
| 1940 | 4-(3,4-Bis(4-nonyloxybenzoyloxy)benzylideneamino)azobenzene | 183.70 | 150.70 | 151.40 | 32.30 |
| 1941 | 4,4'-Bis(4-(4-pentyloxybenzylideneamino)benzoyloxy)diphenylmethane | 81.70 | 113.00 | 114.00 | -32.30 |
| 1942 | 4-Pentyl-4'-undecyloxyazobenzene | 96.94 | 128.80 | 129.30 | -32.36 |
| 1943 | 4,5-Dichloro-1,3-phenylene bis(4-(4-hexadecyloxybenzylideneamino)benzoate) | 157.00 | 189.10 | 189.40 | -32.40 |
| 1944 | 1-(4-Nonylbiphenyl-4'-yl)-3-(4-cyanobiphenyl-4'-yl)propane-1,3-dione | 54.59 | 89.40 | 87.00 | -32.41 |
| 1945 | 2-Chloro-1,4-bis(4-octyloxyphenylmethyleneamino)benzene | 152.84 | 121.30 | 120.40 | 32.44 |
| 1946 | 1-Methylheptyl 5-(4-pentadecyloxybenzoyloxyphenylethynyl)-thiophene-2-carboxylate | 169.35 | 135.60 | 136.90 | 32.45 |
| 1947 | 1,3-Bis(4-(2-hydroxy-4-octyloxybenzylideneamino)benzoyloxy)benzene | 90.31 | 120.90 | 122.80 | -32.49 |
| 1948 | 4-(4-Butoxyphenylazo)phenyl 3'-bromo-4'-methoxybiphenyl-4-carboxylate | 52.50 | 85.60 | 85.00 | -32.50 |
| 1949 | 1-(4'-Pentylazobenzene-4-oxy)-6-(4'-butylazobenzene-4-oxy)hexane | 91.88 | 126.10 | 124.40 | -32.52 |
| 1950 | 1-Methylheptyl 4-(4-(4-nonyloxy-alpha-methylcinnamoyloxy)benzoyloxy)benzoate | 143.87 | 111.70 | 111.20 | 32.67 |
| 1951 | 4-(3,3,4,4,5,5,6,6,7,7,8,8,8-Tridecafluorooctyloxycarbonyl)phenyl 4-(decyloxy)benzoate | 99.82 | 129.60 | 132.50 | -32.68 |
| 1952 | 4-(4-(2-Ethoxy-1-methyl-2-oxoethoxycarbonyl)phenylethynyl)phenyl 3-fluoro-4-tetradecyloxybenzoate | 149.59 | 116.80 | 116.90 | 32.69 |
| 1953 | Cholesteryl 6-(4-(4-dodecylphenyliminomethyl)phenoxy)hexanoate | 96.91 | 129.20 | 129.60 | -32.69 |
| 1954 | 2,6-Bis(4-((4Z)-4-decyloxy)phenyl)anthracene | 75.11 | 108.70 | 107.80 | -32.69 |
| 1955 | 4'-(2-(4-Methylphenyl)-1-ethynyl)-2'-methyl-4-ethyltolane | 75.90 | 42.60 | 43.10 | 32.80 |
| 1956 | 4,5-Dichloro-1,3-phenylene bis(4-(4-tetradecyloxybenzylideneamino)benzoate) | 141.09 | 172.90 | 173.90 | -32.81 |
| 1957 | 1-(4-Hexylphenyl)-4-(4-propylphenyl)-1,3-butadiyne | 47.44 | 80.10 | 80.30 | -32.86 |
| 1958 | Octyl 4-(4-(4-(1-oxohexyl)-1-piperazinyl)phenylazo)benzoate | 90.49 | 58.20 | 57.60 | 32.89 |
| 1959 | 4-Chloro-1,3-phenylene bis(4-(4-dodecyloxybenzylideneamino)benzoate) | 131.61 | 164.00 | 164.50 | -32.89 |
| 1960 | 4-(2-(4-Butylcyclohexyl)ethyl)-4'-(2-(4-pentylcyclohexyl)ethyl)biphenyl | 52.58 | 83.20 | 85.50 | -32.92 |
| 1961 | N,N',N''-Trinonanoyl-2,4,6-trimethyl-1,3,5-benzenetriamine | 55.28 | 88.20 | 88.20 | -32.92 |
| 1962 | 4'-Decyloxybiphenyl-4-yl 4-(2-decyloxy-1-oxopropoxy)benzoate | 165.39 | 132.50 | 132.40 | 32.99 |
| 1963 | N-(4-Methoxyphenyl)-alpha-(4-pentyloxyphenyl)nitrone | 82.91 | 112.30 | 115.90 | -32.99 |
| 1964 | 4-(2,2,3,3,4,4,4-heptafluorobutyloxycarbonyl)phenyl 4-(nonyloxy)benzoate | 74.31 | 107.50 | 107.30 | -32.99 |
| 1965 | 4-Undecyloxybiphenyl-4'-yl 4-(1-methylheptyloxy)thiobenzoate | 74.30 | 106.40 | 107.30 | -33.00 |
| 1966 | 4-(3,4-Bis(4-dodecyloxybenzoyloxy)benzylideneamino)azobenzene | 207.04 | 174.90 | 174.00 | 33.04 |
| 1967 | 4-Dodecanoyloxy-3-methyl-4'-ethoxyazobenzene | 153.05 | 119.90 | 120.00 | 33.05 |
| 1968 | 4-(4-(4-(4-Butoxybenzoyloxy)benzoyl)-1-piperazinyl)phenyl 4-(4-butoxybenzoyloxy)benzoate | 73.57 | 40.50 | 40.40 | 33.17 |
| 1969 | 1,12-Bis(4'-pentylazobenzene-4-oxy)dodecane | 186.00 | 154.40 | 152.80 | 33.20 |
| 1970 | 1,4-Bis(4-(4-propoxybenzoyloxy)benzylideneamino)butane | 150.32 | 117.00 | 117.10 | 33.22 |
| 1971 | N-(4-Methoxyphenyl)-alpha-(4-heptyloxyphenyl)nitrone | 90.97 | 120.40 | 124.20 | -33.23 |
| 1972 | 1,5-Bis(4-(2-hydroxy-4-undecyloxybenzylideneamino)benzoyloxy)-2,2-dimethylpentane | 129.76 | 163.20 | 163.00 | -33.24 |
| 1973 | Cholesteryl 6-(4-octyloxybiphenyl-4'-oxy)hexanoate | 73.46 | 106.30 | 106.70 | -33.24 |
| 1974 | N-(4-Methoxyphenyl)-alpha-(4-tetradecyloxyphenyl)nitrone | 178.36 | 148.70 | 145.10 | 33.26 |
| 1975 | Ethyl 4-(4-(4-Methoxybenzylideneamino)benzoyloxy)benzoate | 116.78 | 83.10 | 83.50 | 33.28 |
| 1976 | 4-Octyloxy-3-nitrobenzoic acid | 75.28 | 105.50 | 108.60 | -33.32 |
| 1977 | 4-Cyanophenyl 4-(3-(4-(4'-undecylbiphenyl-4-carbonyloxy)-3-fluorobenzoyloxy)benzoyloxy)benzoate | 89.37 | 121.10 | 122.70 | -33.33 |
| 1978 | 4-(3-(4-(Octyloxy)phenyl)-1-oxo-2-propenyloxy)phenyl 2-chloro-3-methylpentanoate | 60.13 | 91.30 | 93.50 | -33.37 |
| 1979 | 4'-(2-(4-Propylphenyl)-1-ethynyl)-2'-ethyl-4-hexyltolane | 41.21 | 70.90 | 74.60 | -33.39 |
| 1980 | 1,3-Bis(4-(4-(4-octyloxybenzoyloxy)benzylideneamino)phenoxy)-2-propanol | 110.08 | 144.60 | 143.50 | -33.42 |
| 1981 | 1,2,19,20-Tetrahydroxyeicosane | 138.57 | 168.30 | 172.00 | -33.43 |
| 1982 | 1,6-Bis(4'-methylazobenzene-4-oxy)hexane | 130.85 | 97.90 | 97.40 | 33.45 |
| 1983 | 4,4'-Bis(4-(4-ethoxybenzylideneamino)benzoyloxy)diphenylmethane | 122.45 | 88.80 | 89.00 | 33.45 |
| 1984 | 4,4'''-Dihexadecyloxy-p-quaterphenyl | 204.99 | 171.60 | 171.50 | 33.49 |
| 1985 | 1,4-Bis(4-(4-ethoxybenzoyloxy)benzylideneamino)butane | 141.75 | 109.00 | 108.20 | 33.55 |
| 1986 | 4-(4-Decyloxybenzylideneamino)phenyl 4-isopropylbenzoate | 72.69 | 105.60 | 106.30 | -33.61 |
| 1987 | 6-O-Octyl-D-galacitol | 148.78 | 115.90 | 115.10 | 33.68 |
| 1988 | 1,5-Bis(4-(2-hydroxy-4-hexadecyloxybenzylideneamino)benzoyloxy)-2,2-dimethylpentane | 236.79 | 203.60 | 203.10 | 33.69 |
| 1989 | 2-Methyl-1,3-phenylene bis(4-(4-nonyloxyphenyliminomethyl)benzoate) | 172.79 | 139.40 | 139.10 | 33.69 |
| 1990 | 1,3-Phenylene bis(4-(4'-tetradecylbiphenyl-4-carbonyloxy)-2-methylbenzoate) | 132.37 | 164.40 | 166.10 | -33.73 |
| 1991 | 1,5-Bis(4-(2-hydroxy-4-octyloxyphenyliminomethyl)phenyloxy)pentane | 196.96 | 163.10 | 163.20 | 33.76 |
| 1992 | 4,5-Dichloro-1,3-phenylene bis(4-(4-decyloxybenzylideneamino)benzoate) | 174.97 | 140.60 | 141.20 | 33.77 |
| 1993 | N,N',N''-Trihexanoyl-2,4,6-trimethyl-1,3,5-benzenetriamine | 85.98 | 51.90 | 52.20 | 33.78 |
| 1994 | 1-(4-(N-Ethoxycarbonylpiperazinyl)phenyl)-3-(4-nonyloxyphenyl)-1-propargyl-3-imine | 104.69 | 76.30 | 70.90 | 33.79 |
| 1995 | 4-(10-Undecenyloxy)biphen-4'-yl 5-methoxy-2-thiophenecarboxylate | 264.00 | 243.20 | 230.20 | 33.80 |
| 1996 | 3-Nonylthio-1,2-propanediol | 82.44 | 112.40 | 116.30 | -33.86 |
| 1997 | 1-Methylheptyl 4-(4-(4-decyloxy-alpha-methylcinnamoyloxy)benzoyloxy)benzoate | 147.77 | 115.70 | 113.90 | 33.87 |
| 1998 | 1-(4-Dodecylbiphenyl-4'-yl)-3-(3-butoxyphenyl)propane-1,3-dione | 154.49 | 121.10 | 120.60 | 33.89 |
| 1999 | 1-(4-Dodecylbiphenyl-4'-yl)-3-(4-butoxyphenyl)propane-1,3-dione | 154.49 | 121.10 | 120.60 | 33.89 |
| 2000 | 4-(4-Ethoxyphenylazo)-3-methylphenyl 4-dodecyloxybenzoate | 152.29 | 119.40 | 118.30 | 33.99 |
| 2001 | 6-O-Dodecyl-D-galacitol | 163.32 | 132.00 | 129.20 | 34.12 |
| 2002 | 2-Octanoyl-5-(4-hexyloxyphenyl)thiophene | 76.06 | 111.40 | 110.20 | -34.14 |
| 2003 | 4-(5-(4-(4-Pentylcyclohexyl)phenyl)-1,2,4-oxadiazol-3-yl)pyridine | 47.74 | 78.90 | 81.90 | -34.16 |
| 2004 | 3,4-Difluoro-2,5-bis(4-dodecyloxyphenylethynyl)thiophene | 235.79 | 210.20 | 201.60 | 34.19 |
| 2005 | 1,5-Bis(4-(2-hydroxy-4-tetradecyloxybenzylideneamino)benzoyloxy)pentane | 144.94 | 181.30 | 179.20 | -34.26 |
| 2006 | 4'-Undecyloxy-3'-nitrobiphenyl-4-carboxylic acid | 77.10 | 111.90 | 111.40 | -34.30 |
| 2007 | 1-(4-Octylbiphenyl-4'-yl)-3-(4'-cyanobiphenyl)propane-1,3-dione | 51.48 | 85.40 | 85.80 | -34.32 |
| 2008 | N,N',N''-Tributanoyl-2,4,6-trimethyl-1,3,5-benzenetriamine | 57.35 | 27.60 | 23.00 | 34.35 |
| 2009 | 1,5-Bis(4-(2-hydroxy-4-nonyloxybenzylideneamino)benzoyloxy)-3,3-dimethylpentane | 180.20 | 147.00 | 145.80 | 34.40 |
| 2010 | 4-(4-Methylphenylazo)phenyl 4'-methoxybiphenyl-4-carboxylate | 42.08 | 73.10 | 76.50 | -34.42 |
| 2011 | 4-Propyl-4'-butoxyazobenzene | 126.74 | 92.40 | 92.30 | 34.44 |
| 2012 | 2-Propanoyl-1,4-phenylene bis(4-nonyloxybenzoate) | 176.27 | 141.70 | 141.80 | 34.47 |
| 2013 | Bis(4-ethoxyphenyl) 2-(6-(4-(4-butylphenylazo)phenoxy)hexyloxy) terephthalate | 153.66 | 117.30 | 119.10 | 34.56 |
| 2014 | 1,7-Bis(4-butoxyazobenzene-4'-carbonyloxy)heptane | 98.90 | 133.90 | 133.50 | -34.60 |
| 2015 | 1-Methylheptyl 5-(3-fluoro-4-tetradecyloxybenzoyloxyphenylethynyl)-thiophene-2-carboxylate | 99.67 | 132.60 | 134.30 | -34.63 |
| 2016 | 6-O-Undecyl-D-galacitol | 162.76 | 128.00 | 128.10 | 34.66 |
| 2017 | Ethyl 4-(4-(4-(1-oxo-10-undecenyl)-1-piperazinyl)phenylazo)benzoate | 26.21 | 56.70 | 61.00 | -34.79 |
| 2018 | 6-Decyloxy-2-(4'-decyloxyphenyliminomethyl)quinoline | 177.89 | 143.90 | 143.10 | 34.79 |
| 2019 | 2-Octanoyl-5-(4-pentyloxyphenyl)thiophene | 75.09 | 107.30 | 109.90 | -34.81 |
| 2020 | 4-(4-(1-Methylheptyloxycarbonyl)phenyloxycarbonyl)phenyl 4-(5-(4-(4-nonyloxybenzoyloxy)-benzoyloxy)pentoxy)benzoate | 182.56 | 147.30 | 147.70 | 34.86 |
| 2021 | 4-Octyl-N-(4-octadecyloxyphenylmethylene)benzenamine | 131.30 | 165.50 | 166.20 | -34.90 |
| 2022 | Butyl 4-(4-(4-(1-oxo-10-undecenyl)-1-piperazinyl)phenylazo)benzoate | 28.59 | 64.70 | 63.50 | -34.91 |
| 2023 | 4-Decyloxybiphenyl-4'-yl 4-(2-decyloxypropanoyl-3-methylbenzoate | 88.19 | 124.80 | 123.10 | -34.91 |
| 2024 | 1-(4'-Butylazobenzene-4-oxy)-6-(4'-methylazobenzene-4-oxy)hexane | 144.63 | 110.00 | 109.70 | 34.93 |
| 2025 | N-(4-Nonyloxybenzylidene)-4-nonylaniline | 167.44 | 133.20 | 132.50 | 34.94 |
| 2026 | 3-Hydroxypropyl 4-((4'-octyloxybiphenyl-4-yl)oxymethyl)benzoate | 82.48 | 115.90 | 117.50 | -35.02 |
| 2027 | 1,6-Bis(4'-butylazobenzene-4-oxy)hexane | 88.14 | 122.10 | 123.20 | -35.06 |
| 2028 | 1,3-Phenylene bis(4-(4'-decylbiphenyl-4-carbonyloxy)-3-fluorobenzoate) | 115.70 | 149.30 | 150.80 | -35.10 |
| 2029 | 1-Methylheptyl 4-(4-(4-nonyloxycinnamoyloxy)benzoyloxy)benzoate | 83.30 | 116.60 | 118.50 | -35.20 |
| 2030 | 1,2,2-Trimethylpropyl 4-(4-(4-methoxybenzoyloxy)benzylideneamino)benzoate | 112.82 | 78.60 | 77.50 | 35.32 |
| 2031 | 4-Isothiocyanatophenyl 4-(4-pentylcyclohexyl)benzoate | 61.65 | 96.30 | 97.00 | -35.35 |
| 2032 | 2-(4-Pentylphenyl)-5-phenylpyridine | 24.13 | 60.60 | 59.50 | -35.37 |
| 2033 | 1-Methylheptyl 4-(4-(3-fluoro-4-octyloxybenzoyloxy)-3-fluorobenzoyloxy)benzoate | 84.03 | 120.50 | 119.40 | -35.37 |
| 2034 | N,N'-Dihexadecanoyl-2,3,5,6-tetrakis(hexadecanoyloxy)-1,4-benzenediamine | 352.00 | 378.90 | 387.40 | -35.40 |
| 2035 | 1-(4-Dodecylbiphenyl-4'-yl)-3-(3-pentyloxyphenyl)propane-1,3-dione | 161.51 | 125.20 | 126.10 | 35.41 |
| 2036 | 3-(4-(4-Heptylcyclohexyl)phenyl)-5-(5-decyl-2-thienyl)isoxazole | 71.38 | 102.90 | 106.80 | -35.42 |
| 2037 | 1,3-Phenylene bis(4-(4'-heptylbiphenyl-4-carbonyloxy)-3-fluorobenzoate) | 92.21 | 125.10 | 127.70 | -35.49 |
| 2038 | 1,5-(4-(2-Hydroxy-4-butoxyb1,5-Bis(4-(2-hydroxy-4-butoxybenzylideneamino)benzoyloxy)-3,3-dimethylpentaneenzylideneamino)benzoyloxy)-3,3-dimethylpentane | 141.96 | 106.60 | 106.30 | 35.66 |
| 2039 | Cholesteryl 5-(4-butoxybiphenyl-4'-oxy)pentanoate | 50.92 | 86.10 | 86.60 | -35.68 |
| 2040 | Hexyl 4-(4-(4-methoxybenzoyloxy)benzylideneamino)benzoate | 135.39 | 99.20 | 99.70 | 35.69 |
| 2041 | Methyl 4-(4-(4-Methoxybenzylideneamino)benzoyloxy)benzoate | 116.81 | 80.40 | 81.10 | 35.71 |
| 2042 | 2-Methyl-1,3-phenylene bis(4-(3-chloro-4-pentadecyloxyphenylethenyl)benzoate) | 183.53 | 145.70 | 147.70 | 35.83 |
| 2043 | 4-Dodecyloxy-4'-cyanobiphenyl | 139.93 | 104.40 | 104.10 | 35.83 |
| 2044 | N-(4-Heptyloxyphenyl)-6-decyloxyquinoline-2-carboxamide | 147.16 | 113.40 | 111.30 | 35.86 |
| 2045 | 5-(4-Nonyloxyphenyl)-2-(4-octyloxy)phenylamido-1,3,4-thiadiazole | 42.63 | 76.70 | 78.50 | -35.87 |
| 2046 | 3,5-Dihydroxycyclohexyl 3,4-bis(octyloxy)benzoate | 51.82 | 88.30 | 87.70 | -35.88 |
| 2047 | Propyl 4-(4-(4-methoxybenzylideneamino)benzoyloxy)benzoate | 121.10 | 87.10 | 85.20 | 35.90 |
| 2048 | 4-(2,2,3,3,4,4,4-Heptafluorobutyloxycarbonyl)phenyl 4-(4-(perfluorohexyl)butoxy)benzoate | 135.81 | 101.00 | 99.90 | 35.91 |
| 2049 | 4-(2-Butoxy-1-methyl-2-oxoethoxycarbonyl)phenyl 4-(3-(6-nonyloxy-2-naphthalenyl)-1-oxo-2-propenyloxy)benzoate | 67.27 | 102.40 | 103.20 | -35.93 |
| 2050 | 4-Methyl-3-chloro-N-(4-(4-methoxybenzoyloxy)benzylidene)aniline | 102.66 | 67.90 | 66.70 | 35.96 |
| 2051 | 1-(2-(5-Octylthienyl))-3-(4-chlorophenylamino)-2-propen-1-one | 101.41 | 66.10 | 65.40 | 36.01 |
| 2052 | 1-(4-Dodecylbiphenyl-4'-yl)-3-(4-pentyloxyphenyl)propane-1,3-dione | 161.51 | 125.20 | 125.50 | 36.01 |
| 2053 | N-4-Butyoxyphenyl-6-decyloxyquinoline-2-carboxamide | 137.52 | 101.30 | 101.50 | 36.02 |
| 2054 | 4,4'-Bis(4-(4-methoxybenzylideneamino)benzoyloxy)diphenylmethane | 117.55 | 83.50 | 81.50 | 36.05 |
| 2055 | 4-(6-Acryloyloxyhexyloxy)benzoic acid | 88.69 | 120.10 | 124.80 | -36.11 |
| 2056 | 4-(4-(1-Methylheptyloxycarbonyl)phenyloxycarbonyl)phenyl 4-(5-(4-(4-undecyloxybenzoyloxy)benzoyloxy)pentyloxy)benzoate | 191.56 | 155.40 | 155.40 | 36.16 |
| 2057 | 3-(4-(4-Cyanophenoxycarbonyl)phenoxycarbonyl)phenyl 3-fluoro-4-(4-hexadecyloxybenzoyloxy)benzoate | 183.88 | 148.10 | 147.70 | 36.18 |
| 2058 | 2,5-Bis(4-octyloxyphenyl)-thiazolo[5.4-d]thiazole | 187.58 | 159.40 | 151.40 | 36.18 |
| 2059 | N,N'-Diundecanoyl-2,3,5-trimethylbenzene-1,4-diamine | 130.92 | 94.20 | 94.70 | 36.22 |
| 2060 | 6-O-Tetradecyl-alpha-D-galactopyranose | 120.73 | 85.60 | 84.40 | 36.33 |
| 2061 | 2-Fluoro-4-octyloxyphenyl 4-butoxythiobenzoate | 138.81 | 103.50 | 102.40 | 36.41 |
| 2062 | 1-(4-Pentylcyclohexylethynyl)-4-(4-butylphenylethynyl)benzene | 35.59 | 69.80 | 72.00 | -36.41 |
| 2063 | 7-(4-(4-(4-Nonylphenoxycarbonyl)phenoxycarbonyl)phenolheptyl) 4'-heptylbiphenylcarboxylate | 117.61 | 154.10 | 154.10 | -36.49 |
| 2064 | 4-(4-(1-Methylheptyloxycarbonyl)phenyloxycarbonyl)phenyl 4-(5-(4-(4-decyloxybenzoyloxy)benzoyloxy)nonyloxy)benzoate | 203.70 | 167.60 | 167.20 | 36.50 |
| 2065 | (3,4-Dicyano-2,5-thiophenediyl) bis(ethynediyl-4,1-phenylene) 3,4-bis(octyloxy)benzoate | 115.58 | 151.60 | 152.10 | -36.52 |
| 2066 | 1-(4-Octylbiphenyl-4'-yl)-3-(4-chlorophenyl)propane-1,3-dione | 57.84 | 92.10 | 94.40 | -36.56 |
| 2067 | 4-(4-Dodecyloxyphenylazo)phenyl 3-(4-(4-octyloxybenzoyloxy)phenylmethyleneamino)benzoate | 195.60 | 158.80 | 159.00 | 36.60 |
| 2068 | 4-(2,2,3,3,4,4,5,5-Octafluoropentyloxycarbonyl)phenyl 4-(nonyloxy)benzoate | 65.39 | 98.60 | 102.00 | -36.61 |
| 2069 | 1,8-Bis(4-(4-octyloxybenzoyloxybenzylideneamino)octane | 137.66 | 173.60 | 174.30 | -36.64 |
| 2070 | Pentyl 4-(4-(4-nonyloxy-2,3,5,6-tetrafluorophenylethynyl)benzyloxy)benzoate | 148.92 | 112.80 | 112.20 | 36.72 |
| 2071 | 1-(4-Butylcyclohexylethynyl)-4-(4-butylcyclohexylethynyl)benzene | 24.51 | 61.00 | 61.40 | -36.89 |
| 2072 | 1-(4-Pentylcyclohexylethynyl)-4-(4-ethylphenylethynyl)benzene | 24.40 | 61.70 | 61.30 | -36.90 |
| 2073 | 2-Cyano-1,3-phenylene bis(4-(4'-octadecylbiphenyl-4-carbonyloxy)-3-fluorobenzoate) | 167.96 | 205.70 | 204.90 | -36.94 |
| 2074 | 1-(4-Dodecylbiphenyl-4'-yl)-3-(2-decyloxyphenyl)propane-1,3-dione | 109.03 | 145.40 | 146.00 | -36.97 |
| 2075 | 1-(4'-Pentylazobenzene-4-oxy)-6-(4'-propylazobenzene-4-oxy)hexane | 86.13 | 122.10 | 123.20 | -37.07 |
| 2076 | 4-Heptyloxybenzoic acid | 74.08 | 109.70 | 111.20 | -37.12 |
| 2077 | N-(2-(4-(4-Propylcyclohexyl)cyclohexyl)ethyl)-3,4-difluoropyrrole | 67.64 | 93.30 | 104.80 | -37.16 |
| 2078 | 1-(2-(5-Hexylthienyl))-3-(4-fluorophenylamino)-2-propen-1-one | 102.92 | 66.30 | 65.70 | 37.22 |
| 2079 | 3-Cyclohexyl-5-(4-(4-propylcyclohexyl)phenyl)-1,2,4-oxadiazole | 25.16 | 58.60 | 62.40 | -37.24 |
| 2080 | N,N'-Dinonanoyl-2,3,5-trimethylbenzene-1,4-diamine | 114.57 | 78.00 | 77.30 | 37.27 |
| 2081 | 2,2-Dimethylpropyl 4-(4-(4-acetoxybenzoyloxy)benzylideneamino)benzoate | 64.83 | 101.50 | 102.10 | -37.27 |
| 2082 | 4-(4-Ethoxyphenylazo)-2,3,5,6-tetramethylphenyl 4-(dodecyloxy)benzoate | 134.50 | 96.60 | 97.20 | 37.30 |
| 2083 | 4-(4-(4-(4-Hexyloxybenzoyloxy)benzoyl)-1-piperazinyl)phenyl 4-(4-hexyloxybenzoyloxy)benzoate | 93.47 | 56.70 | 56.10 | 37.37 |
| 2084 | 2,3,6,7,10,11-Hexakis(hexyloxy)triphenylene | 170.88 | 132.70 | 133.50 | 37.38 |
| 2085 | Bis(4-(4-dodecyloxyphenyliminomethyl)phenyl) hexanedioate | 155.45 | 193.10 | 192.90 | -37.45 |
| 2086 | 1-((5-Hexen-1-yl)phenyl)-4-(4-pentylphenyl)-1,3-butadiyne | 52.91 | 90.70 | 90.40 | -37.49 |
| 2087 | 3-(4-(2-(4-Dodecyloxy-3-nitrophenyl)-1-ethenyl)benzoyloxy)-2-methylphenyl 4-(2-(3-iodo-4-dodecyloxyphenyl)-1-ethenyl)benzoate | 120.00 | 88.80 | 82.50 | 37.50 |
| 2088 | 1,3-Phenylene bis(4-(4'-hexylbiphenyl-4-carbonyloxy)-2-methylbenzoate) | 135.61 | 99.80 | 98.10 | 37.51 |
| 2089 | 4-Nitrobenzylidene 4'-nonylaniline | 61.05 | 99.00 | 98.60 | -37.55 |
| 2090 | 2-Methyl-4-octyloxyphenyl 4'-butoxythiobenzoate | 134.36 | 94.90 | 96.80 | 37.56 |
| 2091 | 4-(4-Propylcyclohexyl)isothiocyanatobenzene | 44.82 | 83.00 | 82.40 | -37.58 |
| 2092 | 4-Chloro-1,3-phenylene bis(4-(4-decyloxybenzylideneamino)benzoate) | 111.12 | 147.80 | 148.70 | -37.58 |
| 2093 | 4-(4-(1-Methylheptyloxycarbonyl)phenyloxycarbonyl)phenyl 4-(3-(4-(4-decyloxybenzoyloxy)-benzoyloxy)propoxy)benzoate | 180.60 | 143.30 | 143.00 | 37.60 |
| 2094 | 3-(4-(2-(4-Tetradecyloxy-3-nitrophenyl)-1-ethenyl)benzoyloxymethyl)benzyl 4-(2-(2-nitro-4-tetradecyloxyphenyl)-1-ethenyl)benzoate | 98.10 | 137.20 | 135.80 | -37.70 |
| 2095 | 4-(2-Chloro-3-methylpentanoyloxy)biphenyl-4'-yl 4-heptyloxy-2,3-difluorobenzoate | 52.15 | 89.50 | 89.90 | -37.75 |
| 2096 | 1,2,17,18-Tetrahydroxyoctadecane | 123.64 | 160.20 | 161.40 | -37.76 |
| 2097 | 1-Methylheptyl 4'-(3-chloro-4-tetradecyloxybenzoyloxy)biphenyl-4-carboxylate | 86.92 | 124.60 | 124.70 | -37.78 |
| 2098 | 1,9-Bis(4-cyanobiphenyl-4'-yloxy)nonane | 123.96 | 85.60 | 86.10 | 37.86 |
| 2099 | 4-(omega-Aminohexyloxy)-4'-cyanobiphenyl | 30.68 | 61.10 | 68.60 | -37.92 |
| 2100 | 1,3-Phenylene bis(4-(4'-heptylbiphenyl-4-carbonyloxy)benzoate) | 161.94 | 123.10 | 124.00 | 37.94 |
| 2101 | N,N'-Dipentanoyl-3,4,5,6-tetramethylbenzene-1,2-diamine | 76.80 | 38.10 | 38.80 | 38.00 |
| 2102 | 1-(4-Dodecylbiphenyl-4'-yl)-3-(2-undecyloxyphenyl)propane-1,3-dione | 111.96 | 149.40 | 150.00 | -38.04 |
| 2103 | Bis(4-heptyloxyphenyl) 4,4'-biphenyldicarboxylate | 158.15 | 120.70 | 120.10 | 38.05 |
| 2104 | 3-Methyl-4-(4-pentylcyclohexylethynyl)-1-((4-cyanophenyl)ethynyl)benzene | 89.75 | 49.40 | 51.70 | 38.05 |
| 2105 | 4-Propyl-N-(4-pentadecyloxyphenylmethylene)benzenamine | 96.04 | 133.20 | 134.10 | -38.06 |
| 2106 | 4-(4-Cyanophenyl)-1-(4-dodecyloxyphenyl)-butadiene | 132.47 | 92.60 | 94.40 | 38.07 |
| 2107 | 4-Nonyloxyphenyl 4-nitrobenzoate | 139.49 | 103.20 | 101.40 | 38.09 |
| 2108 | Hexa(4-hexyloxyphenoxymethyl)benzene | 120.60 | 160.00 | 158.70 | -38.10 |
| 2109 | 2-(4-(4-Decyloxybenzoyloxy)-3-chlorophenylazo)naphthalene | 66.25 | 104.10 | 104.40 | -38.15 |
| 2110 | 4-(Pyridine-4-ylmethyleneamino)phenyl 4-heptyloxybenzoate | 76.24 | 114.00 | 114.50 | -38.26 |
| 2111 | N,N'-Dioctanoyl-2,3,5,6-tetramethylbenzene-1,4-diamine | 98.07 | 62.30 | 59.80 | 38.27 |
| 2112 | N-(4-Pentyloxybenzylidene)-4-hexadecylaniline | 182.97 | 145.30 | 144.60 | 38.37 |
| 2113 | Pentyl 4-(4-(4-octyloxy-2,3,5,6-tetrafluorophenylethynyl)benzyloxy)benzoate | 146.24 | 108.70 | 107.80 | 38.44 |
| 2114 | 2-Nonanoyl-1,4-phenylene bis(4-nonyloxybenzoate) | 128.16 | 166.00 | 166.60 | -38.44 |
| 2115 | 5-Methyl-5'-(2-(4-decyloxyphenyl)ethyl)-2,2'-bipyridine | 103.77 | 65.40 | 65.30 | 38.47 |
| 2116 | 1,5-Bis(4-(2-hydroxy-4-decyloxybenzylideneamino)benzoyloxy)-3,3-dimethylpentane | 191.41 | 155.10 | 152.90 | 38.51 |
| 2117 | 4-(4-Ethoxyphenylazo)-2-methylphenyl 4-octyloxybenzoate | 142.01 | 103.30 | 103.40 | 38.61 |
| 2118 | 1,3-Bis(4-(4-(4-decyloxybenzoyloxy)benzylideneamino)phenoxy)-2-propanol | 122.57 | 160.70 | 161.30 | -38.73 |
| 2119 | Ethyl 4-(4-(4-chlorobenzoyloxy)benzylideneamino)benzoate | 120.26 | 80.90 | 81.50 | 38.76 |
| 2120 | Butyl 4-(4-(4-methoxybenzoyloxy)benzylideneamino)benzoate | 131.17 | 91.10 | 92.40 | 38.77 |
| 2121 | Butyl 4-(4-(4-methoxybenzylideneamino)benzoyloxy)benzoate | 129.97 | 91.10 | 91.20 | 38.77 |
| 2122 | 4-Hexyl-4'-(2-methylbutoxy)biphenyl | 124.04 | 84.40 | 85.20 | 38.84 |
| 2123 | 4'-Undecyloxybiphenyl-4-yl 2-(2-methylbutyl)-3-oxo-1-thioxoisoindole-5-carboxylate | 56.81 | 90.50 | 95.70 | -38.89 |
| 2124 | 4-Pentyloxyphenyl 4'-pentylbenzoate | 64.00 | 103.80 | 102.90 | -38.90 |
| 2125 | 4,4'-Didodecyloxyazoxybenzene | 121.80 | 159.30 | 160.70 | -38.90 |
| 2126 | 1,3-Bis(4-(2-hydroxy-4-nonyloxybenzylideneamino)benzoyloxy)benzene | 90.69 | 129.00 | 129.60 | -38.91 |
| 2127 | Pentaerythritol mono-4-propylcyclohexane-1- carboxylate | 80.09 | 115.30 | 119.00 | -38.91 |
| 2128 | 4-(7-Octenyloxy)biphen-4'-yl 5-methoxy-2-thiophenecarboxylate | 198.67 | 231.10 | 237.60 | -38.93 |
| 2129 | 4-(4-Ethoxyphenylazo)-2,6-dimethylphenyl 4-dodecyloxybenzoate | 148.86 | 111.80 | 109.90 | 38.96 |
| 2130 | 4'-Hexadecyloxy-3'-cyanobiphenyl-4-carboxylic acid | 171.23 | 132.20 | 132.20 | 39.03 |
| 2131 | 4-Undecanoyloxy-3-methyl-4'-ethoxyazobenzene | 154.55 | 115.90 | 115.50 | 39.05 |
| 2132 | Cholesteryl 5-(4-hexyloxybiphenyl-4'-oxy)pentanoate | 131.53 | 94.20 | 92.40 | 39.13 |
| 2133 | 4-Octyloxyphenyl 3-fluoro-4-thiocyanatophenyl terephthalate | 67.34 | 103.00 | 106.50 | -39.16 |
| 2134 | 4-(4-Decyloxyphenylazoxy)phenyl 2-chloro-3-methylpentanoate | 139.07 | 103.10 | 99.90 | 39.17 |
| 2135 | 4-Cyanophenyl 4-(3-(4-(4'-hexadecylbiphenyl-4-carbonyloxy)benzoyloxy)benzoyloxy)benzoate | 101.83 | 140.30 | 141.00 | -39.17 |
| 2136 | Octakis(tridecanoyloxy)-9,10-anthraquinone | 477.18 | 436.10 | 438.00 | 39.18 |
| 2137 | 2-Chloro-4-methylpentyl 4-(3-(4-nonyloxyphenyl)-1-oxo-2-propenyloxy)benzoate | 129.60 | 92.40 | 90.40 | 39.20 |
| 2138 | 4-(4-Pyridylmethyleneamino)phenyl 4-hexyloxybenzoate | 71.24 | 110.00 | 110.50 | -39.26 |
| 2139 | N,N'-Dipentadecanoyl-2,5,6-trimethyl-4-pentadecanoyloxy-1,3-benzenediamine | 142.96 | 182.30 | 182.30 | -39.34 |
| 2140 | 1,3-Phenylene bis(4-(4-pentadecyloxybenzoylthio)benzoate) | 141.86 | 180.20 | 181.30 | -39.44 |
| 2141 | 6-(9-Decenyloxy)-2-naphthoic acid | 152.55 | 114.30 | 113.10 | 39.45 |
| 2142 | 1,3-Bis(4-(2-hydroxy-4-hexyloxybenzylideneamino)benzoyloxy)benzene | 64.44 | 104.80 | 103.90 | -39.46 |
| 2143 | 1,3-Bis(4-(4-(4-hexyloxybenzoyloxy)benzylideneamino)phenoxy)-2-propanol | 89.42 | 128.40 | 128.90 | -39.48 |
| 2144 | m-Terphenyl-4,4''-diyl bis(4-(4-octyloxybenzoyloxy)benzoate) | 93.22 | 133.50 | 132.70 | -39.48 |
| 2145 | 4-Heptyl-4'-hexyloxyazobenzene | 76.10 | 116.60 | 115.60 | -39.50 |
| 2146 | 4-Heptyl-4'-ethoxyazobenzene | 61.65 | 100.50 | 101.20 | -39.55 |
| 2147 | 3,3',4,4'-Biphenyltetracarboxy-N,N'-bis(pentyloxyphenyl)diimide | 65.03 | 101.20 | 104.60 | -39.57 |
| 2148 | 4-(4-Ethoxyphenylazo)-3-methylphenyl 4-decyloxybenzoate | 150.52 | 111.40 | 110.90 | 39.62 |
| 2149 | 1-Methylheptyl 4'-(3-chloro-4-dodecyloxybenzoyloxy)biphenyl-4-carboxylate | 76.97 | 116.50 | 116.60 | -39.63 |
| 2150 | 4-Heptylcyclohexyl 4-methoxycinnamate | 113.99 | 73.40 | 74.30 | 39.69 |
| 2151 | 2-Octanoyl-5-(4-heptyloxyphenyl)thiophene | 75.40 | 115.40 | 115.10 | -39.70 |
| 2152 | Di(4'-propoxyphenyl) cyclohexane-1,4-dicarboxylate | 130.13 | 89.40 | 90.40 | 39.73 |
| 2153 | 1,9-Bis(4-butoxyazobenzene-4'-carbonyloxy)nonane | 103.77 | 142.00 | 143.50 | -39.73 |
| 2154 | 1,3-Phenylene bis(4-(4'-nonylbiphenyl-4-carbonyloxy)-3-fluorobenzoate) | 180.65 | 141.30 | 140.90 | 39.75 |
| 2155 | 4-Ethylphenyl 4-(4-pentyloxybenzylideneamino)benzoate | 54.35 | 93.90 | 94.10 | -39.75 |
| 2156 | 4,4'-Dipropoxyazoxybenzene | 127.74 | 86.60 | 87.80 | 39.94 |
| 2157 | Phenyl cholest-5-ene-3beta-carboxylate | 44.23 | 81.90 | 84.20 | -39.97 |
| 2158 | Cholesteryl 6-(4-(4-(4,5-bis(ethoxycarbonyl)-1,3-dioxolan-2-yl)phenyliminomethyl)phenoxy)hexanoate | 48.32 | 85.80 | 88.30 | -39.98 |
| 2159 | 1-(4-Pentylcyclohexylethynyl)-4-(4-pentylphenylethynyl)benzene | 33.80 | 73.80 | 73.80 | -40.00 |
| 2160 | 4-Butyl-4'-ethoxyazobenzene | 49.01 | 88.40 | 89.10 | -40.09 |
| 2161 | 3-(4-(2-(4-Dodecyloxy-3-nitrophenyl)-1-ethenyl)benzoyloxy)-2-methylphenyl 4-(2-(3-methoxy-4-dodecyloxyphenyl)-1-ethenyl)benzoate | 81.40 | 122.50 | 121.50 | -40.10 |
| 2162 | 4-Heptyl-4'-tetradecyloxyazobenzene | 109.28 | 149.00 | 149.40 | -40.12 |
| 2163 | 2,5-Bis(4-pentyloxyphenyl)-thiazolo[5,4-d]thiazole | 98.34 | 135.10 | 138.50 | -40.16 |
| 2164 | 1,5-Bis(4-(2-hydroxy-4-dodecyloxybenzylideneamino)benzoyloxy)-3,3-dimethylpentane | 210.17 | 171.20 | 170.00 | 40.17 |
| 2165 | 1-(4-Dodecylbiphenyl-4'-yl)-3-(2-ethoxyphenyl)propane-1,3-dione | 79.52 | 48.50 | 39.30 | 40.22 |
| 2166 | 2-Bromo-4-octyloxyphenyl 4-octyloxythiobenzoate | 156.88 | 117.80 | 116.60 | 40.28 |
| 2167 | 2,4-Dichloro-N-(4-(4-methoxybenzoyloxy)benzylidene)aniline | 107.49 | 68.30 | 67.20 | 40.29 |
| 2168 | 4-(2,2,3,3,4,4,4-Heptafluorobutyloxycarbonyl)phenyl 4-(decyloxy)benzoate | 72.18 | 111.50 | 112.50 | -40.32 |
| 2169 | 2-Cyano-1,3-phenylene bis(4-(4-nonyloxyphenyliminomethyl)benzoate) | 98.48 | 138.80 | 138.80 | -40.32 |
| 2170 | 1-(2-(5-Cyanothienyl))-3-(4-decyloxyphenylamino)-2-propen-1-one | 33.64 | 74.40 | 74.00 | -40.36 |
| 2171 | N,N'-Dioctadecanoyl-2,3,5,6-tetramethylbenzene-1,4-diamine | 103.44 | 143.10 | 143.80 | -40.36 |
| 2172 | 6-Decyloxy-2-(4-butoxyphenylazo)benzothiazole | 144.04 | 106.70 | 103.60 | 40.44 |
| 2173 | 1,11-Bis(4'-nitroazobenzene-4-oxy)undecane | 158.37 | 116.70 | 117.90 | 40.47 |
| 2174 | 4-(4-Ethoxyphenylazo)phenyl heptanoate | 67.14 | 107.30 | 107.70 | -40.56 |
| 2175 | 4-Butoxy-3-fluoro-4'-(4-pentylcyclohexyl)biphenyl | 40.14 | 79.50 | 80.90 | -40.76 |
| 2176 | 1-Isothiocyanato-4-(4'-propylbicyclohexyl)benzene | 32.68 | 72.60 | 73.50 | -40.82 |
| 2177 | 4,4'-Bis(4-(4-octyloxybenzoyloxy)benzylideneamino)diphenylamine | 83.93 | 120.50 | 124.80 | -40.87 |
| 2178 | 4,5-Dichloro-1,3-phenylene bis(4-(4-dodecyloxybenzylideneamino)benzoate) | 116.92 | 156.80 | 157.80 | -40.88 |
| 2179 | 4-Hexyloxyphenyl 3-fluoro-4-thiocyanatophenyl terephthalate | 55.98 | 94.90 | 96.90 | -40.92 |
| 2180 | Pentadecyl 2-(4-dodecyloxyphenyl)-6-cycloheptimidazolecarboxylate | 54.63 | 92.40 | 95.60 | -40.97 |
| 2181 | 4-Nonyl-4'-thiocyanatobiphenyl | 127.76 | 88.80 | 86.70 | 41.06 |
| 2182 | 4-Butyl-N-(4-octadecyloxyphenylmethylene)benzenamine | 190.74 | 149.30 | 149.60 | 41.14 |
| 2183 | 4'-(10-Undecenyloxy)biphenyl 5-methoxy-2-thiophenecarboxylate | 264.00 | 240.70 | 222.80 | 41.20 |
| 2184 | 4-Hexyloxy-4''-cyano-p-terphenyl | 34.95 | 74.50 | 76.20 | -41.25 |
| 2185 | Bis(4-methoxyphenyl) 2,2'-bithiophene-5,5'-dicarboxylate | 112.88 | 82.00 | 71.60 | 41.28 |
| 2186 | 1-Ethylheptyl 4-(4-(4-tetradecyloxy-3-fluorobenzoyloxy)-3-fluorobenzoyloxy)-2-fluorobenzoate | 106.99 | 149.80 | 148.30 | -41.31 |
| 2187 | 1,3-Phenylene bis(4-(4-hexadecyloxyphenyliminomethyl)benzoate) | 164.39 | 203.60 | 205.70 | -41.31 |
| 2188 | 1,9-Bis(4-(4-heptyloxybenzoyloxy)benzylideneamino)nonane | 210.58 | 169.60 | 169.20 | 41.38 |
| 2189 | 4-Pentyl-4'-heptyloxyazobenzene | 71.02 | 112.60 | 112.40 | -41.38 |
| 2190 | 1,3-Phenylene bis(4-(3-chloro-4-dodecyloxyphenylethenyl)benzoate) | 88.42 | 129.00 | 129.80 | -41.38 |
| 2191 | 1,10-Bis(4-(2-hydroxy-4-octyloxyphenyliminomethyl)phenyloxy)decane | 227.50 | 183.30 | 186.10 | 41.40 |
| 2192 | 4-(2,2,3,3,4,4,4-Heptafluorobutyloxycarbonyl)phenyl 4-(4-perfluorobutyl)butoxy)benzoate | 130.51 | 91.70 | 89.10 | 41.41 |
| 2193 | N,N'-Dioctadecanoyl-2,3,5-trimethylbenzene-1,4-diamine | 192.88 | 150.70 | 151.40 | 41.48 |
| 2194 | 4-(4-Butoxyphenylazo)phenyl 3-(4-(4-octyloxybenzoyloxy)phenylmethyleneamino)benzoate | 85.09 | 126.40 | 126.60 | -41.51 |
| 2195 | 4-Pentanoyl-4'-pentanoyloxyazobenzene | 68.01 | 109.00 | 109.60 | -41.59 |
| 2196 | N-(4-Butoxybenzylidene)-4-hexylaniline | 58.52 | 100.90 | 100.20 | -41.68 |
| 2197 | Bis(4-dodecyloxyphenyl) 2,2'-bipyridine-5,5'-dicarboxylate | 87.97 | 127.10 | 129.70 | -41.73 |
| 2198 | 4-(2,2,3,3,4,4,5,5-Octafluoropentyloxycarbonyl)phenyl 4-(perfluorohexylhexyloxy)benzoate | 138.59 | 100.20 | 96.80 | 41.79 |
| 2199 | Bis(4-butylphenyl)diazene N-oxide | 46.91 | 92.40 | 88.80 | -41.89 |
| 2200 | 1-(4-Pentyloxyphenylethynyl)-4-(3,5-dimethyl-4-cyanophenylethynyl)benzene | 90.21 | 47.60 | 48.30 | 41.91 |
| 2201 | Butyl 4-(4-Pentyloxycarbonyloxyphenyl)iminomethyl)benzoate | 82.29 | 124.20 |  | -41.91 |
| 2202 | 2-Decyloxy-6-(4-(4-decyloxyphenyl)buta-1,3-diynyl)naphthalene | 75.74 | 116.80 | 117.70 | -41.96 |
| 2203 | 4-(Pyridine-4-ylmethyleneamino)phenyl 4-pentyloxybenzoate | 64.42 | 106.00 | 106.40 | -41.98 |
| 2204 | 1,3-Bis(4-(4-(4-pentyloxybenzoyloxy)benzylideneamino)phenoxy)-2-propanol | 77.12 | 120.30 | 119.20 | -42.08 |
| 2205 | 1-(4'-Pentylbicyclohexyl)-2,3,4-trifluorobenzene | 114.90 | 71.20 | 72.80 | 42.10 |
| 2206 | 4-Heptanoyloxy-2,3-dimethyl-4'-ethoxyazobenzene | 131.63 | 92.10 | 89.50 | 42.13 |
| 2207 | 4-((4-Propylphenylmethylene)amino)benzoic acid | 52.94 | 94.60 | 95.10 | -42.16 |
| 2208 | Butane-1,3-diyl bis(4-(4'-octyloxybiphenyl-4-yl)oxymethyl)benzoate) | 151.70 | 111.30 | 109.50 | 42.20 |
| 2209 | 4,4'-Bis(4-(4-propoxybenzylideneamino)benzoyloxy)diphenylmethane | 139.41 | 96.90 | 97.10 | 42.31 |
| 2210 | 1,2,21,22-Tetrahydroxydocosane | 134.02 | 176.40 | 176.40 | -42.38 |
| 2211 | 4-(4-Ethoxyphenylazo)-2,3,5-trimethylphenyl 4-dodecyloxybenzoate | 145.79 | 104.20 | 103.40 | 42.39 |
| 2212 | 11-(Pentakis(4-methoxyphenylethynyl)phenoxy)undecan-1-ol | 91.70 | 57.10 | 49.30 | 42.40 |
| 2213 | 4-Hexyl-N-(4-octyloxyphenylmethylene)benzenamine | 158.56 | 117.00 | 116.10 | 42.46 |
| 2214 | 2-(4-Butoxyphenyl)-5-(4-((4-ethyl-2,3-undecadienyl)oxy)phenyl)pyrimidine | 99.40 | 63.90 | 56.80 | 42.60 |
| 2215 | 1,3-Bis(4-(2-hydroxy-4-decyloxybenzylideneamino)benzoyloxy)benzene | 97.75 | 137.10 | 140.40 | -42.65 |
| 2216 | 4-Chlororesorcinyl bis(4-(4-nonyloyxbenzoyloxy)benzoate) | 103.25 | 145.70 | 145.90 | -42.65 |
| 2217 | N-(2-Hydroxy-4-hexyloxybenzylidene)-4'-dodecylphenylaniline | 75.75 | 118.50 | 118.40 | -42.65 |
| 2218 | 4-(2-(4-(11-Vinyloxyundecyloxy)phenyl)vinyl)benzonitrile | 65.27 | 101.50 | 108.00 | -42.73 |
| 2219 | N,N',N''-Tripentanoyl-2,4,6-trimethyl-1,3,5-benzenetriamine | 79.25 | 39.80 | 36.50 | 42.75 |
| 2220 | 4-(2-Butoxy-1-methyl-2-oxoethoxycarbonyl)phenyl 4-(3-(6-octyloxy-2-naphthalenyl)-1-oxo-2-propenyloxy)benzoate | 57.96 | 98.30 | 100.80 | -42.84 |
| 2221 | 2-Cyano-1,3-phenylene bis(4-(4-octyloxyphenyliminomethyl)benzoate) | 90.36 | 130.70 | 133.40 | -43.04 |
| 2222 | 2-Butyl-5-(4'-cyanobiphen-4-yl)thiophene | 27.89 | 68.80 | 71.00 | -43.11 |
| 2223 | 1,2-Bis(4-n-octyloxybenzoyl)hydrazine | 144.22 | 101.10 |  | 43.12 |
| 2224 | 4-(4-Ethoxyphenylazo)-2,3,5,6-tetramethylphenyl 4-decyloxybenzoate | 128.36 | 88.50 | 85.10 | 43.26 |
| 2225 | 1,3-Phenylene bis(4-(3-fluoro-4-decyloxybenzyoyloxy)-2-fluorobenzoate) | 206.84 | 165.10 | 163.50 | 43.34 |
| 2226 | Bis(4-ethoxyphenyl) 2-(3-(4-(4-butylphenylazo)phenoxy)propyloxy)terephthalate | 62.55 | 105.20 | 105.90 | -43.35 |
| 2227 | 1-Methylheptyl 4'-(3-fluoro-4-tetradecyloxyphenylpropiolyloxy)biphenyl-4-carboxylate | 85.35 | 129.10 | 128.70 | -43.35 |
| 2228 | N,N'-Didodecanoyl-2,6-dimethylbenzene-1,4-diamine | 153.16 | 109.90 | 109.80 | 43.36 |
| 2229 | 5alpha-Cholestan-3beta-ol decanoate | 145.20 | 105.00 | 101.80 | 43.40 |
| 2230 | 2,5-Bis(4-propoxyphenyl)-thiazolo[5,4-d]thiazole | 71.80 | 119.00 | 115.30 | -43.50 |
| 2231 | 6-S-Hexadecyl-6-thio-alpha-D-galactopyranose | 164.14 | 139.70 | 120.60 | 43.54 |
| 2232 | 1,3-Phenylene bis(4-(2-fluoro-4-undecyloxybenzyoyloxy)-2-fluorobenzoate) | 127.93 | 173.10 | 171.50 | -43.57 |
| 2233 | 1,3-Phenylene bis(4-(4-hexyloxybenzoylthio)benzoate) | 65.03 | 107.50 | 108.70 | -43.67 |
| 2234 | 5alpha-Cholestan-3beta-ol dodecanoate | 159.00 | 113.10 | 115.30 | 43.70 |
| 2235 | 2-Chloro-4-methylpentyl 4-(3-(4-hexyloxyphenyl)-1-oxo-2-propenyloxy)benzoate | 123.78 | 80.30 | 79.90 | 43.88 |
| 2236 | 3-(4-Decyloxyphenyl)-5-(4-bromophenyl)isoxazole | 131.30 | 93.40 | 87.40 | 43.90 |
| 2237 | 1-Ethylheptyl 4-(4-(3-fluoro-4-undecyloxybenzoyloxy)-3-fluorobenzoyloxy)-2-fluorobenzoate | 94.96 | 137.70 | 138.90 | -43.94 |
| 2238 | 4-(2-Butoxy-1-methyl-2-oxoethoxycarbonyl)phenyl 4-(3-(6-decyloxy-2-naphthalenyl)-1-oxo-2-propenyloxy)benzoate | 62.39 | 106.40 | 106.40 | -44.01 |
| 2239 | 4-Propylcyclohexylbenzyl perfluorohexyl thioether | 106.52 | 71.60 | 62.40 | 44.12 |
| 2240 | 1,5-Bis(4-butoxyazobenzene-4'-carbonyloxy)pentane | 82.69 | 125.90 | 126.90 | -44.21 |
| 2241 | (2-Fluoro-4-hexyloxyphenyl) 4-ethoxythiobenzoate | 133.53 | 87.30 | 89.30 | 44.23 |
| 2242 | 6-O-Dodecyl-alpha-D-galactopyranose | 116.44 | 77.60 | 72.20 | 44.24 |
| 2243 | 6-Decyloxy-2-(4'-pentyloxyphenyliminomethyl)quinoline | 82.65 | 123.70 | 126.90 | -44.25 |
| 2244 | 1-(4-Dodecylbiphenyl-4'-yl)-3-(3-nonyloxyphenyl)propane-1,3-dione | 183.85 | 141.30 | 139.60 | 44.25 |
| 2245 | 4-Heptyloxyphenyl 3-fluoro-4-thiocyanatophenyl terephthalate | 58.12 | 99.00 | 102.50 | -44.38 |
| 2246 | 4-(4-(2-Ethoxy-1-methyl-2-oxoethoxycarbonyl)phenylethynyl)phenyl 4-octadecyloxybenzoate | 176.89 | 132.00 | 132.50 | 44.39 |
| 2247 | 4-(2-Chloro-3-methylbutanoyloxy)biphenyl-4'-yl 4-nonyloxy-2,3-difluorobenzoate | 137.74 | 93.60 | 93.30 | 44.44 |
| 2248 | N-(2-Hydroxy-4-decyloxybenzylidene)-4'-dodecylphenylaniline | 179.66 | 134.70 | 135.10 | 44.56 |
| 2249 | 4-Benzoyloxybenzoic acid | 52.41 | 97.20 | 97.20 | -44.79 |
| 2250 | 2,4-Bis(4-hexadecyloxyazobenzene-4'-amino)-6-methoxy-1,3,5-triazine | 213.70 | 168.90 |  | 44.80 |
| 2251 | 4-Nonanoyloxy-3-methyl-4'-ethoxyazobenzene | 150.71 | 107.80 | 105.90 | 44.81 |
| 2252 | 4-(4-Methoxyphenylazo)phenyl biphenyl-4-carboxylate | 36.13 | 80.70 | 81.00 | -44.87 |
| 2253 | 4-Pentyl-4'-propoxyazobenzene | 51.53 | 96.40 | 96.40 | -44.87 |
| 2254 | 4-Decyloxybenzoic acid | 76.93 | 121.90 | 121.80 | -44.87 |
| 2255 | 1,3-Phenylene bis(4-(4-octyloxybenzoylthio)benzoate) | 80.88 | 123.70 | 125.80 | -44.92 |
| 2256 | 4-Pentyl-N-(4-octadecyloxyphenylmethylene)benzenamine | 199.14 | 153.40 | 154.10 | 45.04 |
| 2257 | 4-(2,2,3,3,4,4,5,5-Octafluoropentyloxycarbonyl)phenyl 4-(undecyloxy)benzoate | 65.92 | 106.70 | 111.00 | -45.08 |
| 2258 | 4-(4-isobutyloxyphenylazo)phenyl 4-dodecyloxybenzoate | 77.79 | 122.60 | 122.90 | -45.11 |
| 2259 | Ethyl 11-(pentakis(4-pentylphenylethynyl)phenoxy)undecanoate | 157.34 | 117.50 | 112.10 | 45.24 |
| 2260 | Octyl 2,5-bis(4-octyloxybenzoyloxy)benzoate | 113.94 | 159.00 | 159.30 | -45.36 |
| 2261 | 4-Methoxy-N-(3-pyridinylmethylene)-1,1-biphenyl-4'-amine | 35.33 | 80.30 | 80.80 | -45.47 |
| 2262 | N,N'-Ditetradecanoyl-2,3,5-trimethylbenzene-1,4-diamine | 164.60 | 118.40 | 119.10 | 45.50 |
| 2263 | 4-(2,2,3,3,4,4,5,5-Octafluoropentyloxycarbonyl)phenyl 4-(5-(perfluorobutyl)pentyloxy)benzoate | 126.70 | 86.90 | 81.20 | 45.50 |
| 2264 | 4,4'''-Dioctadecyloxy-p-quaterphenyl | 142.03 | 187.80 | 187.60 | -45.57 |
| 2265 | 4'-Undecyloxybiphenyl-4-yl 2-(1-methylpropyl)-1,3-dioxoisoindole-5-carboxylate | 59.87 | 89.80 | 105.50 | -45.63 |
| 2266 | 1-Methylheptyl 4-(4-(4-hexadecyloxycinnamoyloxy)benzoyloxy)benzoate | 99.68 | 144.90 | 145.40 | -45.72 |
| 2267 | t-Butyl 4-(4-(4-chlorobenzoyloxy)benzylideneamino)benzoate | 141.04 | 103.60 | 95.30 | 45.74 |
| 2268 | N-(4-Pentyloxybenzylidene)-4-heptylaniline | 63.72 | 108.90 | 109.50 | -45.78 |
| 2269 | 1,3-Phenylene bis(4-(4'-octylbiphenyl-4-carbonyloxy)-3-fluorobenzoate) | 177.92 | 133.20 | 132.00 | 45.92 |
| 2270 | 2-(4-Butoxyphenyl)-5-(4-(3-methyl-3,4-dodecadienyloxy)phenyl-1,3,4-thiadiazole | 48.72 | 89.60 | 94.70 | -45.98 |
| 2271 | 2-(4-Decyloxyphenyl)-5-(4-decyloxyphenylethynyl)-3,4-thiophenedicarbonitrile | 70.60 | 117.50 | 116.60 | -46.00 |
| 2272 | 4-(4-Methylphenylazo)phenyl biphenyl-4-carboxylate | 32.29 | 78.20 | 78.40 | -46.11 |
| 2273 | 4,4'''-Dioctyloxy-p-quaterphenyl | 60.99 | 107.00 | 107.10 | -46.11 |
| 2274 | N-(4-Hexyloxybenzylidene)-4-hexylaniline | 62.11 | 108.90 | 108.30 | -46.19 |
| 2275 | 2,6,10-Tris(pentyloxy)-3,7,11-tris(nonyloxy)triphenylene | 202.64 | 156.90 | 156.30 | 46.34 |
| 2276 | Di(4'-dodecylphenyl)-cyclohexane-1,4-dicarboxylate | 204.66 | 159.70 | 158.30 | 46.36 |
| 2277 | N-(4-Methoxyphenyl)-alpha-(4-hexyloxyphenyl)nitrone | 75.36 | 116.40 | 121.80 | -46.44 |
| 2278 | Bis(7-(4-(4-butyoxyphenyliminomethyl)phenoxy)heptyl) malonate | 207.07 | 161.20 | 160.60 | 46.47 |
| 2279 | Bis(4-(5-hexylthio-1,3,4-oxadiazole-2-yl)phenyl) terephthalate | 124.12 | 81.60 | 77.60 | 46.52 |
| 2280 | 4-(4-Methoxyphenyl)-2,6-bis(3,4,5-trioctyloxyphenyl)pyrimidine | 174.58 | 128.50 | 128.00 | 46.58 |
| 2281 | 4-(Pyridine-4-ylmethyleneamino)phenyl 4-octyloxybenzoate | 73.11 | 118.10 | 119.70 | -46.59 |
| 2282 | 6-Decyloxy-2-(4-pentyloxyphenylazo)benzothiazole | 65.85 | 110.70 | 112.50 | -46.65 |
| 2283 | 1,1,2-Trimethylpropyl 4-(4-(4-chlorobenzoyloxy)benzylideneamino)benzoate | 137.48 | 99.10 | 90.80 | 46.68 |
| 2284 | 1-(2-(5-Cyanothienyl))-3-(4-dodecyloxyphenylamino)-2-propen-1-one | 36.19 | 82.40 | 82.90 | -46.71 |
| 2285 | Bis(3-(4-(4-butyoxyphenyliminomethyl)phenoxy)propyl) malonate | 83.02 | 128.90 | 129.90 | -46.88 |
| 2286 | 4-Pentadecyloxybenzylidene-4'-ethylaniline | 175.29 | 129.10 | 128.40 | 46.89 |
| 2287 | 6-O-Decyl-alpha-D-galactopyranose | 61.04 | 105.20 | 108.00 | -46.96 |
| 2288 | 2,5-Dihydro-2,5-dimethyl-3,6-bis(4-dodecyloxyphenyl)pyrrolo[3,4-c]pyrrole-1,4-dione | 226.27 | 189.20 | 179.30 | 46.97 |
| 2289 | 1,3-Phenylene bis(4-(4'-octadecylbiphenyl-4-carbonyloxy)-2-fluorobenzoate) | 260.05 | 214.00 | 213.00 | 47.05 |
| 2290 | 4-(3-Decyloxyphenyliminomethyl)phenyl 4-undecyloxybenzoate | 198.40 | 151.70 | 151.30 | 47.10 |
| 2291 | 6-O-Octadecyl-D-galacitol | 108.51 | 156.30 | 155.70 | -47.19 |
| 2292 | 4-Nonanoyloxy-2-methyl-4'-ethoxyazobenzene | 155.12 | 107.80 | 107.90 | 47.22 |
| 2293 | 1,10-Bis(4-cyanobiphenyl-4'-yloxy)decane | 138.96 | 89.60 | 91.70 | 47.26 |
| 2294 | 1-(4-Dodecylbiphenyl-4'-yl)-3-(3-decyloxyphenyl)propane-1,3-dione | 191.66 | 145.40 | 144.30 | 47.36 |
| 2295 | 4-Heptylbenzoic acid | 62.69 | 108.60 | 110.10 | -47.41 |
| 2296 | 2-(5-(4-Nonyloxyphenyl)-1,3,4-thiadiazol-2-yliminomethyl)-5-(octyloxy)phenol | 59.32 | 103.80 | 106.90 | -47.58 |
| 2297 | 1-(2-(5-Butylthienyl))-3-(4-iodophenylamino)-2-propen-1-one | 60.29 | 18.40 | 12.70 | 47.59 |
| 2298 | m-Terphenyl-4,4''-diyl bis(4-(5-butylpyrimidin-2-yl)benzoate) | 103.02 | 57.40 | 55.40 | 47.62 |
| 2299 | Bis(4-pentylphenyl)diazene N-oxide | 55.21 | 100.40 | 102.90 | -47.69 |
| 2300 | Cholesteryl palmitate | 170.46 | 123.40 | 122.70 | 47.76 |
| 2301 | 4-n-Butyloxycinnamic acid | 139.75 | 91.70 | 91.60 | 48.15 |
| 2302 | Di(4'-Ethoxyphenyl)-cyclohexane-1,4-dicarboxylate | 129.75 | 81.30 | 81.60 | 48.15 |
| 2303 | 1,3-Phenylene bis(4-(4-butoxybenzylideneamino)benzoate) | 59.31 | 106.60 | 107.50 | -48.19 |
| 2304 | 2-Hexyl-5-(4'-cyanobiphen-4-yl)thiophene | 29.15 | 76.90 | 77.60 | -48.45 |
| 2305 | 1,3-Phenylene bis(4-(4-heptyloxybenzoylthio)benzoate) | 69.19 | 115.60 | 117.80 | -48.61 |
| 2306 | 1-(6-(3,6,7,10,11-Pentakis(octyloxy)triphenylen-2-yloxy)hexyloxy)-9,10-anthraquinone | 106.45 | 154.20 | 155.20 | -48.75 |
| 2307 | 2-Chloro-3-methylpentyl 4-(3-(4-dodecyloxyphenyl)-1-oxo-2-propenyloxy)benzoate | 151.28 | 104.50 | 102.50 | 48.78 |
| 2308 | 3-(4-(2-(4-Dodecyloxy-3-nitrophenyl)-1-ethenyl)benzoyloxy)phenyl 4-(2-(3-iodo-4-dodecyloxyphenyl)-1-ethenyl)benzoate | 51.50 | 96.40 | 100.30 | -48.80 |
| 2309 | 2-(4-Dodecyloxyphenyl)-5-(4-dodecyloxyphenylethynyl)-3,4-thiophenedicarbonitrile | 84.75 | 133.70 | 133.60 | -48.85 |
| 2310 | 4-Propoxyphenylbenzyl perfluorobutyl thioether | 30.23 | 68.10 | 79.10 | -48.87 |
| 2311 | 4-(2,2,3,3,4,4,5,5-octafluoropentyloxycarbonyl)phenyl 4-(dodecyloxy)benzoate | 66.72 | 110.70 | 115.60 | -48.88 |
| 2312 | Bis(4-ethoxyphenyl) 2-(10-(4-(4-butylphenylazo)phenoxy)decyloxy) terephthalate | 182.33 | 133.40 | 133.40 | 48.93 |
| 2313 | 4'-Decyloxybiphenyl-4-yl 4-(2-heptyloxy-1-oxopropoxy)-3-methoxybenzoate | 67.94 | 115.30 | 117.10 | -49.16 |
| 2314 | 3,4,5-Tris(undecyloxy)benzamide | 60.30 | 100.90 | 109.50 | -49.20 |
| 2315 | 1,3-Bis(4-(4-(4-nonyloxybenzoyloxy)benzylideneamino)phenoxy)-2-propanol | 104.16 | 152.60 | 153.40 | -49.24 |
| 2316 | 7-(Undec-10-enyloxy)-3-(4-octyloxystyryl)quinoline | 112.16 | 153.90 | 161.40 | -49.24 |
| 2317 | 2-Octanoyl-5-(4-nonyloxyphenyl)thiophene | 74.04 | 123.50 | 123.30 | -49.26 |
| 2318 | 1,6-Bis(4'-hexylazobenzene-4-oxy)hexane | 187.82 | 138.30 | 138.50 | 49.32 |
| 2319 | 2-Methyl-1,3-phenylene bis(2-fluoro-4-(4-hexadecylbenzoyloxy)benzoate) | 153.57 | 201.60 | 202.90 | -49.33 |
| 2320 | 4-Undecyloxy-3-nitrobenzoic acid | 68.07 | 117.60 | 117.40 | -49.33 |
| 2321 | 4-(4-Octyloxyphenylazo)phenyl 3-(4-(4-octyloxybenzoyloxy)phenylmethyleneamino)benzoate | 93.66 | 142.60 | 143.00 | -49.34 |
| 2322 | 3,8-Bis(4-pentylphenyl)-1,10-phenanthroline | 91.58 | 139.90 | 141.00 | -49.42 |
| 2323 | 4-Pentyloxybenzoic acid | 53.62 | 101.70 | 103.20 | -49.58 |
| 2324 | 2-(Perfluorobutyl)ethyl 4-phenylthiobenzoate | 116.54 | 83.50 | 66.90 | 49.64 |
| 2325 | 4-(4-(4-Dodecyloxybenzoyloxy)benzoyloxyphenyl)-6'-phenyl-2,2'-bipyridine | 97.34 | 52.30 | 47.60 | 49.74 |
| 2326 | Bis(4-nonyloxyphenyl) 2,5-thiophenedicarboxylate | 195.67 | 146.00 | 145.80 | 49.87 |
| 2327 | 4-(4-Nonyloxyphenyliminomethyl)benzoic acid | 72.51 | 120.00 | 122.40 | -49.89 |
| 2328 | 4-Pentyl-4'-ethoxyazobenzene | 42.34 | 92.40 | 92.30 | -49.96 |
| 2329 | 1,3-Phenylene bis(4-(4'-dodecylbiphenyl-4-carbonyloxy)benzoate) | 213.62 | 163.50 | 163.30 | 50.32 |
| 2330 | 1-Methylheptyl 4-(4-(4-octadecyloxy-3-fluorobenzoyloxy)-3-fluorobenzoyloxy)benzoate | 110.75 | 160.90 | 161.10 | -50.35 |
| 2331 | Cholesteryl heptadecanoate | 178.36 | 127.50 | 128.00 | 50.36 |
| 2332 | 4-(4-Hexyloxyphenyliminomethyl)benzoic acid | 56.03 | 107.90 | 106.40 | -50.37 |
| 2333 | Bis(10-(4-(4-decyloxyphenyliminomethyl)phenoxy)decyl) malonate | 282.24 | 234.00 | 231.70 | 50.54 |
| 2334 | N-(4-Hexadecyloxybenzylidene)-4-ethylaniline | 83.03 | 133.20 | 133.60 | -50.57 |
| 2335 | 4-Hexadecyloxyphenyl 4-nitrobenzoate | 182.72 | 131.40 | 132.10 | 50.62 |
| 2336 | N-(4-Dodecyloxybenzylidene)-4-dodecylaniline | 208.96 | 157.40 | 158.30 | 50.66 |
| 2337 | 1,3-Bis(4-(2-hydroxy-4-octyloxyphenyliminomethyl)phenyloxy)propane | 106.44 | 155.00 | 157.10 | -50.66 |
| 2338 | N,N'-Dihexanoyl-3,4,5,6-tetramethylbenzene-1,2-diamine | 95.74 | 46.10 | 44.90 | 50.84 |
| 2339 | m-Terphenyl-4,4''-diyl bis(4-(5-octylpyrimidin-2-yl)benzoate) | 132.54 | 89.70 | 81.70 | 50.84 |
| 2340 | 4-Butoxybenzoic acid | 48.22 | 97.60 | 99.10 | -50.88 |
| 2341 | 4-Decanoyloxy-3-methyl-4'-ethoxyazobenzene | 160.81 | 111.80 | 109.90 | 50.91 |
| 2342 | 4-(2-Chloro-3-methylbutanoyloxy)biphenyl-4'-yl 4-undecyloxy-2,3-difluorobenzoate | 49.88 | 101.70 | 100.80 | -50.92 |
| 2343 | 4-(2,2,3,3,4,4,5,5-Octafluoropentyloxycarbonyl)phenyl 4-hexyloxybenzoate | 128.18 | 86.50 | 77.20 | 50.98 |
| 2344 | 4,4'-Dinonanoyloxydiphenyldiacetylene | 179.98 | 130.20 | 128.80 | 51.18 |
| 2345 | 1-(4-Dodecylbiphenyl-4'-yl)-3-(4-dodecyloxyphenyl)propane-1,3-dione | 204.29 | 153.40 | 153.10 | 51.19 |
| 2346 | 4-(2-Chloro-3-methylpentanoyloxy)biphenyl-4'-yl 4-octyloxy-2,3-difluorobenzoate | 44.38 | 93.60 | 95.60 | -51.22 |
| 2347 | 4-Butyl-4'-butoxyazobenzene | 46.16 | 96.40 | 97.40 | -51.24 |
| 2348 | 3-(4-(4-Cyanophenoxycarbonyl)phenoxycarbonyl)phenyl 3-fluoro-4-(4-pentadecyloxybenzoyloxy)benzoate | 196.65 | 144.10 | 145.40 | 51.25 |
| 2349 | N,N'-Dinonanoyl-2,3,5,6-tetramethylbenzene-1,4-diamine | 119.16 | 70.40 | 67.90 | 51.26 |
| 2350 | 1,3-Bis(4-(2-hydroxy-4-dodecyloxybenzylideneamino)benzoyloxy)benzene | 103.00 | 153.30 | 154.30 | -51.30 |
| 2351 | 4-(3,3,4,4,5,5,6,6,7,7,8,8,8-Tridecafluorooctyloxycarbonyl)phenyl 4-(tetradecyloxy)benzoate | 94.89 | 145.70 | 146.30 | -51.41 |
| 2352 | N-(4-Nonyloxybenzylidene)-4-octylaniline | 179.84 | 129.10 | 128.40 | 51.44 |
| 2353 | 7-(Undec-10-enyloxy)-3-(4-tetradecyloxystyryl)quinoline | 132.06 | 178.10 | 183.60 | -51.54 |
| 2354 | 1-(2-(5-Chlorothienyl))-3-(4-hexyloxyphenylamino)-2-propen-1-one | 28.65 | 74.40 | 80.20 | -51.55 |
| 2355 | 1-Methylheptyl 4-(4-(4-heptyloxy-3-fluorobenzoyloxy)-3-fluorobenzoyloxy)benzoate | 65.82 | 116.50 | 117.40 | -51.58 |
| 2356 | Cholesteryl 6-(4-(4-(3-methylpentoyloxy)phenyliminomethyl)phenoxy)hexanoate | 47.98 | 99.30 | 99.70 | -51.72 |
| 2357 | 2,3,6,7-Tetrakis(octyloxy)-1,5-bis(2-hydroxyethoxy)-9,10-anthraquinone | 214.38 | 167.90 | 162.50 | 51.88 |
| 2358 | 2'-Nitro-m-terphenyl-4,4''-diyl bis(4-(4-nonyloxybenzoyloxy)benzoate) | 83.85 | 133.30 | 136.10 | -52.25 |
| 2359 | 1,8-Bis(4-dodecyloxyazobenzene-4'-carbonyloxy)octane | 253.24 | 202.60 | 200.80 | 52.44 |
| 2360 | 1,9-Bis(4-(4-propoxybenzoyloxy)benzylideneamino)nonane | 84.75 | 137.20 | 137.20 | -52.45 |
| 2361 | 1-Methylheptyl 5-(4-dodecyloxybenzoyloxyphenylethynyl)-thiophene-2-carboxylate | 173.36 | 123.50 | 120.80 | 52.56 |
| 2362 | 4'-Decyloxybiphenyl-4-yl 4-(2-octyloxy-1-oxopropoxy)-3-methoxybenzoate | 70.22 | 119.30 | 122.80 | -52.58 |
| 2363 | 1,3-Bis(4-(2-hydroxy-4-undecyloxybenzylideneamino)benzoyloxy)benzene | 96.07 | 145.20 | 148.70 | -52.63 |
| 2364 | 4-Hexylbenzoic acid | 53.22 | 104.50 | 106.00 | -52.78 |
| 2365 | 1-((5-Hexen-1-yl)phenyl)-4-(4-butylphenyl)-1,3-butadiyne | 31.92 | 86.60 | 84.70 | -52.78 |
| 2366 | Bis(4-(5-octylthio-1,3,4-oxadiazole-2-yl)phenyl) terephthalate | 145.02 | 97.80 | 92.20 | 52.82 |
| 2367 | Bis(4-hexylphenyl)diazene N-oxide | 59.69 | 108.50 | 112.60 | -52.91 |
| 2368 | Heptyl 4-(4-(4-methoxybenzoyloxy)benzylideneamino)benzoate | 156.98 | 103.30 | 104.00 | 52.98 |
| 2369 | 1,7-Bis(4-(4-heptyloxybenzoyloxy)benzylideneamino)heptane | 213.61 | 161.50 | 160.50 | 53.11 |
| 2370 | Hexyl 2,5-bis(4-octyloxybenzoyloxy)benzoate | 98.95 | 150.90 | 152.10 | -53.15 |
| 2371 | N,N',N''-Tritetradecanoyl-2,4,6-trimethyl-1,3,5-benzenetriamine | 96.25 | 148.80 | 149.40 | -53.15 |
| 2372 | N-(2-Hydroxy-4-heptyloxybenzylidene)-4'-dodecylphenylaniline | 178.56 | 122.50 | 125.40 | 53.16 |
| 2373 | Bis(4-decyloxyphenyl) 2,5-thiophenedicarboxylate | 206.85 | 154.10 | 153.60 | 53.25 |
| 2374 | 4-(4-(4-Pentyloxyphenyliminomethyl)-3-hydroxyphenoxy)butylmethacrylate | 154.31 | 98.50 | 100.90 | 53.41 |
| 2375 | Hexakis(4-(5'-dodecyl-2'-thienyl)phenyl)benzene | 214.30 | 254.20 | 267.80 | -53.50 |
| 2376 | 4-(4-Dodecylphenylazo)phenyl 3-(4-(4-octyloxybenzoyloxy)phenylmethyleneamino)benzoate | 103.88 | 157.60 | 157.40 | -53.52 |
| 2377 | 4-(2,2,3,3,4,4,5,5-Octafluoropentyloxycarbonyl)phenyl 4-((perfluorohexyl)butoxy)benzoate | 135.63 | 92.20 | 82.00 | 53.63 |
| 2378 | 4-(4-Decyloxyphenylazo)phenyl 3-(4-(4-octyloxybenzoyloxy)phenylmethyleneamino)benzoate | 97.77 | 150.70 | 151.40 | -53.63 |
| 2379 | N-(2-Hydroxy-4-octyloxybenzylidene)-4'-dodecylphenylaniline | 74.05 | 126.60 | 127.70 | -53.65 |
| 2380 | 1-(4-(4-Cyanophenylazo)phenyl)-4-(1-oxo-10-undecenyl)piperazine | 99.50 | 48.10 | 45.80 | 53.70 |
| 2381 | 3-(4-(2-(4-Dodecyloxy-3-nitrophenyl)-1-ethenyl)benzoyloxy)-2-methylphenyl 4-(2-(3-chloro-4-dodecyloxyphenyl)-1-ethenyl)benzoate | 68.10 | 120.40 | 121.80 | -53.70 |
| 2382 | Bis(6-(4-(4-butyoxyphenyliminomethyl)phenoxy)hexyl) malonate | 206.05 | 153.20 | 152.20 | 53.85 |
| 2383 | Di(4'-nonylphenyl)-cyclohexane-1,4-dicarboxylate | 187.48 | 135.50 | 133.50 | 53.98 |
| 2384 | 4-Chlorophenyl 4-tetradecyloxythiobenzoate | 166.13 | 113.80 | 112.10 | 54.03 |
| 2385 | 4-Octyl-N-(4-pentadecyloxyphenylmethylene)benzenamine | 206.19 | 153.40 | 152.10 | 54.09 |
| 2386 | 4-(4-(4-(4-Pentoxybenzoyloxy)benzoyl)-1-piperazinyl)phenyl 4-(4-pentoxybenzoyloxy)benzoate | 102.34 | 48.60 | 48.20 | 54.14 |
| 2387 | 4-Pentyl-4'-butoxyazobenzene | 47.92 | 100.50 | 102.10 | -54.18 |
| 2388 | 3-(4-(2-(4-Dodecyloxy-3-nitrophenyl)-1-ethenyl)benzoyloxomethyl)benzyl 4-(2-(3-nitro-4-dodecyloxyphenyl)-1-ethenyl)benzoate | 170.50 | 121.10 | 116.30 | 54.20 |
| 2389 | 4-(4-Ethoxyphenylazo)-3,5-dimethylphenyl 4-dodecyloxybenzoate | 167.16 | 111.80 | 112.90 | 54.26 |
| 2390 | 4-Hexyloxyphenyl 4'-pentylbenzoate | 53.02 | 107.90 | 107.30 | -54.28 |
| 2391 | 4-Cyanophenyl 4-hexadecyloxybenzoate | 185.80 | 131.50 | 131.40 | 54.40 |
| 2392 | 6-(5-Hexenyloxy)-2-naphthoic acid | 147.90 | 98.10 | 93.50 | 54.40 |
| 2393 | 4-(4'-Heptyloxybenzoyloxy)benzylidene 4'-isopropylaniline | 39.79 | 93.50 | 94.20 | -54.41 |
| 2394 | 1,7-Bis(4-nitroazobenzene-4'-oxy)heptane | 155.37 | 100.60 | 100.90 | 54.47 |
| 2395 | 4-Octyloxy-4'-(4-pentylbicyclo[2.2.2]oct-1-yl)biphenyl | 65.90 | 118.00 | 120.40 | -54.50 |
| 2396 | 2,5-Bis(4-ethoxyphenyl)-thiazolo[5,4-d]thiazole | 61.58 | 110.90 | 116.10 | -54.52 |
| 2397 | 4-Nitrobenzylidene 4'-undecylaniline | 52.28 | 107.10 | 106.80 | -54.52 |
| 2398 | N-(4-Octadecyloxybenzylidene)-4-hexadecylaniline | 253.33 | 197.80 | 198.80 | 54.53 |
| 2399 | 4-Chlorophenyl 4-pentadecyloxythiobenzoate | 173.34 | 117.80 | 118.70 | 54.64 |
| 2400 | 4'-Docosanyloxy-3'-nitrobiphenyl-4-carboxylic acid | 210.37 | 156.40 | 155.70 | 54.67 |
| 2401 | 4-Proyl-N-(4-octadecyloxyphenylmethylene)benzenamine | 201.38 | 145.30 | 146.70 | 54.68 |
| 2402 | 6-S-Octadecyl-6-thio-alpha-D-galactopyranose | 180.12 | 132.70 | 125.30 | 54.82 |
| 2403 | 4-Decyloxy-3-nitrobenzoic acid | 59.25 | 113.60 | 114.10 | -54.85 |
| 2404 | 2,4- Bis(3',4',5'-tridecyloxyphenyl)-6-(4'-decyloxyphenyl)pyrimidine | 158.18 | 212.00 | 213.10 | -54.92 |
| 2405 | 4-Dodecyloxyphenyl 4-nitrobenzoate | 169.87 | 115.30 | 114.80 | 55.07 |
| 2406 | 4-(2-Butoxy-1-methyl-2-oxoethoxycarbonyl)phenyl 4-(3-(6-tetradecyloxy-2-naphthalenyl)-1-oxo-2-propenyloxy)benzoate | 68.23 | 122.60 | 123.30 | -55.07 |
| 2407 | 10-Hydroxydecyl 4-((4'-octyloxybiphenyl-4-yl)oxymethyl)benzoate | 198.03 | 144.20 | 142.90 | 55.13 |
| 2408 | N,N'-Didodecanoyl-2,3,5,6-tetramethylbenzene-1,4-diamine | 147.28 | 94.60 | 92.10 | 55.18 |
| 2409 | 4-Heptylcyclohexyl 4-hexyloxycinnamate | 144.89 | 92.30 | 89.70 | 55.19 |
| 2410 | 1-Methylheptyl 4-(4-(4-dodecyloxy-alpha-methylcinnamoyloxy)benzoyloxy)benzoate | 174.81 | 123.80 | 119.30 | 55.51 |
| 2411 | 2,5-Bis(4-decyloxyphenyl)-thiazolo[5.4-d]thiazole | 223.30 | 175.50 | 167.70 | 55.60 |
| 2412 | 1-(2-(5-Cyanothienyl))-3-(4-tetradecyloxyphenylamino)-2-propen-1-one | 37.11 | 90.50 | 92.90 | -55.79 |
| 2413 | Hexyl 4-(4-(4-(1-oxohexyl)-1-piperazinyl)phenylazo)benzoate | 107.18 | 50.20 | 51.30 | 55.88 |
| 2414 | 1-Ethylheptyl 4-(4-(3-fluoro-4-octyloxybenzoyloxy)-3-fluorobenzoyloxy)benzoate | 69.81 | 124.60 | 125.70 | -55.89 |
| 2415 | 1-(2-(5-Octylthienyl))-3-(4-fluorophenylamino)-2-propen-1-one | 128.70 | 74.30 | 72.80 | 55.90 |
| 2416 | N-(4-Hexyloxybenzylidene)-4-heptylaniline | 56.28 | 113.00 | 112.30 | -56.02 |
| 2417 | 4-Heptyl-4'-octadecyloxyazobenzene | 220.80 | 165.10 | 164.60 | 56.20 |
| 2418 | 7-Undec-10-enyloxy-3-(4'-hexylyoxystyryl)quinoline | 97.05 | 145.80 | 153.30 | -56.25 |
| 2419 | 1-Methylheptyl 4'-(4''-eicosyloxy- 3''-fluorobenzoyloxy)tolan-4-carboxylate | 209.60 | 153.30 | 153.30 | 56.30 |
| 2420 | 2-Chloro-4-octyloxyphenyl 4-octyloxythiobenzoate | 168.23 | 111.40 | 111.90 | 56.33 |
| 2421 | N-(4-Methoxyphenyl)-alpha-(4-octadecyloxyphenyl)nitrone | 215.04 | 164.90 | 158.70 | 56.34 |
| 2422 | 2-Octanoyl-5-(4-octyloxyphenyl)thiophene | 63.46 | 119.40 | 119.90 | -56.44 |
| 2423 | 3-(4-(2-(4-Tetradecyloxy-3-nitrophenyl)-1-ethenyl)benzoyloxy)-2-methylphenyl 4-(2-(3-chloro-4-tetradecyloxyphenyl)-1-ethenyl)benzoate | 84.40 | 136.50 | 140.90 | -56.50 |
| 2424 | 4-(4-(4-Decyloxyphenyliminomethyl)-3-(hydroxyphenoxy)butyl methacrylate | 188.17 | 136.60 | 131.60 | 56.57 |
| 2425 | 2,5-Bis(4-heptyloxyphenyl)-thiazolo[5,4-d]thiazole | 203.25 | 151.30 | 146.50 | 56.75 |
| 2426 | N-(4-Hexyloxybenzylidene)-4-pentylaniline | 48.94 | 104.90 | 105.70 | -56.76 |
| 2427 | 4-Butyl-4'-(5-hexenyl)biphenyl | 39.26 | 94.20 | 96.10 | -56.84 |
| 2428 | 1,5-Bis(4-(2-hydroxy-4-hexadecyloxybenzylideneamino)benzoyloxy)pentane | 140.63 | 197.50 | 197.50 | -56.87 |
| 2429 | 4-Heptylcyclohexyl 4-pentyloxycinnamate | 145.06 | 88.20 | 88.10 | 56.96 |
| 2430 | Pentane-2,4-diyl bis(4-(4'-octyloxybiphenyl-4-yl)oxymethyl)benzoate) | 153.90 | 96.70 | 96.90 | 57.00 |
| 2431 | 4-Propoxybenzoic acid | 38.10 | 93.60 | 95.10 | -57.00 |
| 2432 | 1-(4-Dodecylbiphenyl-4'-yl)-3-(2-dodecyloxyphenyl)propane-1,3-dione | 97.97 | 153.40 | 155.00 | -57.03 |
| 2433 | 4-Ethyl-4'-(7-octenyl)biphenyl | 43.20 | 94.20 | 100.30 | -57.10 |
| 2434 | 1,9-Bis(4,4''-didecyloxy-p-terphenyl-2'-yl)-2,5,8-trioxanonane | 231.71 | 176.70 | 174.60 | 57.11 |
| 2435 | 1-((4-Penten-1-yl)phenyl)-4-(4-hexylphenyl)-1,3-butadiyne | 36.88 | 90.70 | 94.20 | -57.32 |
| 2436 | 1,3-Phenylene bis(4-(2-fluoro-4-tetradecyloxybenzyoyloxy)-2-fluorobenzoate) | 253.08 | 197.40 | 195.50 | 57.58 |
| 2437 | Bis(10-(4-(4-decylphenyliminomethyl)phenoxy)decyl) malonate | 288.29 | 231.60 | 230.60 | 57.69 |
| 2438 | 4-(4-Methylphenyl)-2,6-bis(3,4,5-trioctyloxyphenyl)pyrimidine | 71.71 | 126.00 | 129.60 | -57.89 |
| 2439 | Propyl 2,5-bis(4-octyloxybenzoyloxy)benzoate | 82.09 | 138.80 | 140.00 | -57.91 |
| 2440 | 4-(4-(4-(4-Hexadecyloxybenzoyloxy)benzoyl)-1-piperazinyl)phenyl 4-(4-hexadecyloxybenzoyloxy)benzoate | 81.27 | 137.50 | 139.30 | -58.03 |
| 2441 | N,N'-Diheptanoyl-2,4-bis(heptanoyloxy)-1,3-benzenediamine | 72.69 | 131.60 | 130.80 | -58.11 |
| 2442 | 1,3-Phenylene bis(4-(4-nonyloxybenzoylthio)benzoate) | 75.43 | 131.80 | 133.60 | -58.17 |
| 2443 | 3,4,5-Tris(nonyloxy)benzamide | 125.27 | 76.60 | 66.90 | 58.37 |
| 2444 | Terephthalylidene-bis(4-hexadecylaniline) | 207.69 | 154.10 | 149.20 | 58.49 |
| 2445 | 6-Decyloxy-2-(4-nonyloxyphenylazo)benzothiazole | 72.14 | 126.90 | 130.70 | -58.56 |
| 2446 | 4-Pentyl-4'-pentyloxyazobenzene | 45.41 | 104.50 | 104.10 | -58.69 |
| 2447 | 1-(4-Pentyloxyphenylethynyl)-3-methyl-4-(3-methyl-4-cyanophenylethynyl)benzene | 104.36 | 47.60 | 45.50 | 58.86 |
| 2448 | 3-(4-(2-(4-Dodecyloxy-3-nitrophenyl)-1-ethenyl)benzoyloxy)phenyl 4-(2-(3-chloro-4-dodecyloxyphenyl)-1-ethenyl)benzoate | 73.50 | 128.00 | 132.40 | -58.90 |
| 2449 | 4-Chlorophenyl 4-hexadecyloxythiobenzoate | 179.48 | 121.90 | 120.50 | 58.98 |
| 2450 | 4-Heptyl-4'-pentyloxyazobenzene | 55.27 | 112.60 | 114.30 | -59.03 |
| 2451 | 4-(4'-Dodecylcyclohexyl)isothiocyanatobenzene | 179.00 | 119.30 | 119.60 | 59.40 |
| 2452 | 2-Methyl-1,3-phenylene bis(4-(3-fluoro-4-octyloxyphenyliminomethyl)benzoate) | 75.05 | 133.30 | 134.50 | -59.45 |
| 2453 | N,N'-Dioctadecyl-3,4,9,10-perylenebiscarboximide | 175.61 | 127.40 | 115.70 | 59.91 |
| 2454 | 11-(Pentakis(4-pentylphenylethynyl)phenoxy)undecan-1-ol | 76.51 | 125.10 | 136.50 | -59.99 |
| 2455 | 1-Ethylheptyl 4-(4-(4-dodecyloxy-3-fluorobenzoyloxy)-3-fluorobenzoyloxy)-2-fluorobenzoate | 83.40 | 141.70 | 143.40 | -60.00 |
| 2456 | 4-(2-Naphthylazo)-1-naphthyl 4-(heptyloxy)benzoate | 31.60 | 89.20 | 91.60 | -60.00 |
| 2457 | 4-(4-Ethoxyphenylazo)-3,6-dimethylphenyl 4-dodecyloxybenzoate | 172.35 | 111.80 | 112.30 | 60.05 |
| 2458 | 2,4-Bis(3',4',5'-tridecyloxyphenyl)-6-phenylpyrimidine | 120.47 | 182.10 | 180.70 | -60.23 |
| 2459 | 7-(4'-Hexadecyloxybenzoyloxy)isoflavone | 168.99 | 111.20 | 108.70 | 60.29 |
| 2460 | N,N',N''-Trihexadecanoyl-2,4,6-trimethyl-1,3,5-benzenetriamine | 112.95 | 173.10 | 173.40 | -60.45 |
| 2461 | 2-Octanoyl-1,4-phenylene bis(4-nonyloxybenzoate) | 223.12 | 161.90 | 162.60 | 60.52 |
| 2462 | 1,1-Dimethylbutyl 4-(4-(4-methoxybenzoyloxy)benzylideneamino)benzoate | 60.76 | 113.90 | 121.30 | -60.54 |
| 2463 | 4-(2-Butoxy-1-methyl-2-oxoethoxy)phenyl biphenyl-4-carboxylate | 18.13 | 78.90 | 78.70 | -60.57 |
| 2464 | 4-Heptyloxyphenyl 4-nitrobenzoate | 37.47 | 95.10 | 98.10 | -60.63 |
| 2465 | 4-Pentylbenzoic acid | 41.47 | 100.50 | 102.10 | -60.63 |
| 2466 | N,N'-Dihexadecanoyl-2,3,5-trimethylbenzene-1,4-diamine | 195.16 | 134.60 | 134.50 | 60.66 |
| 2467 | 4,4'-Bis(2-methylbutoxy)biphenyl | 124.38 | 68.90 | 63.70 | 60.68 |
| 2468 | 1-(4-Dodecylbiphenyl-4'-yl)-3-(3-undecyloxyphenyl)propane-1,3-dione | 211.50 | 149.40 | 150.80 | 60.70 |
| 2469 | 4'-Decyloxybiphenyl-4-yl 4-(2-nonyloxy-1-oxopropoxy)-3-methoxybenzoate | 63.68 | 123.40 | 124.40 | -60.72 |
| 2470 | 2,5,6,9,12,13-Hexapentyloxydibenzo[fg,op]naphthacene | 140.59 | 81.60 | 79.50 | 61.09 |
| 2471 | 2,4,6-Tri-1-dodecynyl-N,N',N''-tris(2-phenylpropyl)-1,3,5-benzenetricarboxamide | 211.71 | 170.10 | 150.60 | 61.11 |
| 2472 | 4-(2-Butoxy-1-methyl-2-oxoethoxycarbonyl)phenyl 4-(3-(6-dodecyloxy-2-naphthalenyl)-1-oxo-2-propenyloxy)benzoate | 55.28 | 114.50 | 116.50 | -61.22 |
| 2473 | N,N'-Ditetradecanoyl-2,3,5,6-tetramethylbenzene-1,4-diamine | 170.52 | 110.80 | 109.20 | 61.32 |
| 2474 | N,N'-Dihexadecanoyl-2,3,5,6-tetramethylbenzene-1,4-diamine | 186.29 | 126.90 | 124.90 | 61.39 |
| 2475 | 1,3-Bis(4-(2-hydroxy-4-tetradecyloxybenzylideneamino)benzoyloxy)benzene | 105.84 | 169.40 | 167.30 | -61.46 |
| 2476 | 1-Methylheptyl 5-(2,3-difluoro-4-tetradecyloxybenzoyloxyphenylethynyl)-thiophene-2-carboxylate | 197.17 | 133.60 | 135.50 | 61.67 |
| 2477 | 4-Decyloxybiphenyl-4'-yl 4-(2-decyloxypropanoyl-3-methoxybenzoate | 64.90 | 127.40 | 126.80 | -61.90 |
| 2478 | Hexakis(4-(4-methylnonyl)phenylethynyl)benzene | 65.68 | 120.40 | 127.70 | -62.02 |
| 2479 | Heptyl 2,5-bis(4-octyloxybenzoyloxy)benzoate | 93.95 | 155.00 | 156.10 | -62.15 |
| 2480 | 4-Heptyl-4'-butoxyazobenzene | 45.93 | 108.60 | 108.10 | -62.17 |
| 2481 | 1,3-Phenylene bis(4-(3-bromo-4-dodecyloxyphenylethenyl)benzoate) | 90.02 | 141.90 | 152.20 | -62.18 |
| 2482 | N,N'-Diundecanoyl-2,3,5,6-tetramethylbenzene-1,4-diamine | 148.79 | 86.50 | 86.60 | 62.19 |
| 2483 | 4-Butyl-4'-octadecyloxyazobenzene | 214.74 | 153.00 | 152.50 | 62.24 |
| 2484 | Butyl 2,5-bis(4-octyloxybenzoyloxy)benzoate | 80.56 | 142.80 | 142.90 | -62.34 |
| 2485 | 4-Methoxyphenyl 4-(4-(4-iodophenylimino)methyl)benzoate | 99.58 | 44.00 | 36.90 | 62.68 |
| 2486 | Isobutyl 4-(4-(4-Chlorobenzoyloxy)benzylideneamino)benzoate | 137.41 | 76.40 | 74.70 | 62.71 |
| 2487 | 1,3-Phenylene bis(4-(4'-pentylbiphenyl-4-carbonyloxy)-2-methylbenzoate) | 154.05 | 91.70 | 91.30 | 62.75 |
| 2488 | 4,4'''-Diheptadecyloxy-p-quaterphenyl | 242.00 | 179.70 | 179.20 | 62.80 |
| 2489 | 8-(4-(4'-Butylbiphenyl))-1-octene | 39.30 | 102.30 |  | -63.00 |
| 2490 | N,N-Bis(2-(3,4-bis(tridecyloxy)benzoylamino)ethyl)-3,4-bis(tridecyloxy)benzamide | 286.70 | 338.50 | 349.70 | -63.00 |
| 2491 | 1-(2-(5-Cyanothienyl))-3-(4-tridecyloxyphenylamino)-2-propen-1-one | 26.73 | 86.50 | 89.80 | -63.07 |
| 2492 | 2,5-Bis(4-hexyloxyphenyl)-thiazolo[5,4-d]thiazole | 85.91 | 143.20 | 149.00 | -63.09 |
| 2493 | Methyl 5-((4'-decyloxybiphenyl-4-yl)oxycarbonyl)-1,3-dioxoisoindole-2-propionate | 41.21 | 101.50 | 104.60 | -63.39 |
| 2494 | 4-Chloro-1,3-phenylene bis(4-(4-nonyloxyphenyliminomethyl)benzoate) | 75.10 | 139.80 | 138.70 | -63.60 |
| 2495 | 4-Butyl-4'-pentyloxyazobenzene | 38.19 | 100.50 | 102.10 | -63.91 |
| 2496 | 1,3-Bis(4-(2-hydroxy-4-hexadecyloxybenzylideneamino)benzoyloxy)benzene | 248.45 | 185.60 | 184.50 | 63.95 |
| 2497 | 1-Ethylheptyl 4-(4-(3-fluoro-4-decyloxybenzoyloxy)-3-fluorobenzoyloxy)-2-fluorobenzoate | 70.68 | 133.70 | 134.70 | -64.02 |
| 2498 | 4-(4-Cyanophenoxycarbonyl)phenyl 3-(4-(4-tetradecyloxybenzoyloxy)benzoyloxy)benzoate | 203.86 | 139.00 | 139.00 | 64.86 |
| 2499 | 4-(4-Ethoxyphenylazo)-2,3,5-trimethylphenyl 4-decyloxybenzoate | 160.82 | 96.10 | 95.90 | 64.92 |
| 2500 | N,N'-Didecanoyl-2,3,5,6-tetramethylbenzene-1,4-diamine | 141.50 | 78.50 | 76.50 | 65.00 |
| 2501 | 4-(3,3,4,4,5,5,6,6,7,7,8,8,8-Tridecafluorooctyloxycarbonyl)phenyl 4-(dodecyloxy)benzoate | 73.62 | 137.60 | 138.90 | -65.28 |
| 2502 | 5-Methyl-2-(4-n-tridecafluorohexylphenyl)isoindole-1,3-dione | 141.96 | 78.40 | 76.60 | 65.36 |
| 2503 | 1,5-Bis(4-(4-octyloxybenzoyloxy)benzylideneamino)pentane | 224.86 | 161.50 | 159.30 | 65.56 |
| 2504 | Bis-((4,4'-didecyloxy-p-terphenyl-2'-yl)methyl)carbonate | 263.48 | 197.40 |  | 66.08 |
| 2505 | N-(4-Butoxybenzylidene)-4-butylaniline | 26.90 | 92.80 | 93.10 | -66.20 |
| 2506 | 4-Heptyl-N-(4-pentadecyloxyphenylmethylene)benzenamine | 217.17 | 149.30 | 150.90 | 66.27 |
| 2507 | Di(4'-heptyloxyphenyl)-cyclohexane-1,4-dicarboxylate | 189.40 | 121.70 | 122.90 | 66.50 |
| 2508 | 3,5-Dihydroxycyclohexyl 3,4-bis(nonyloxy)benzoate | 33.30 | 96.40 | 99.80 | -66.50 |
| 2509 | Benzene hexa-n-heptanoate | 159.50 | 221.20 | 226.10 | -66.60 |
| 2510 | (3,4-Dicyano-2,5-thiophenediyl) bis(ethynediyl-4,1-phenylene) 3,4-bis(nonyloxy)benzoate | 103.47 | 167.80 | 170.20 | -66.73 |
| 2511 | 4,4'-Dioctanoyloxydiphenyldiacetylene | 187.64 | 122.10 | 120.90 | 66.74 |
| 2512 | 1,3-Phenylene bis(4-(3-chloro-4-pentadecyloxyphenylethenyl)benzoate) | 86.76 | 153.30 | 153.50 | -66.74 |
| 2513 | 4-Hexyloxybenzoic acid | 38.32 | 105.70 | 105.20 | -66.88 |
| 2514 | N,N-Bis(2-(3,4-bis(pentyloxy)benzoylamino)ethyl)-3,4-bis(pentyloxy)benzamide | 88.77 | 144.50 | 155.80 | -67.03 |
| 2515 | 4-(4-Hexyloxybenzoyloxy)phenyl (3,4-dimethoxy)benzoate | 26.85 | 94.40 | 94.00 | -67.15 |
| 2516 | 4-Propyl-4'-ethoxyazobenzene | 151.60 | 84.30 | 84.20 | 67.40 |
| 2517 | 1-Hexyl-4-(2-(4-(2-(4-propylcyclohexyl)ethyl)phenyl)ethyl)bicyclo[2.2.2]octane | 51.90 | 112.10 | 119.30 | -67.40 |
| 2518 | 4-(2-(1-Ethylpropoxy)-1-methyl-2-oxoethoxy)phenyl (4'-undecyloxy)biphenyl-4-carboxylate | 165.38 | 98.20 | 97.80 | 67.58 |
| 2519 | 1,10-Decanediyl bis(4-(4'-octyloxybiphenyl-4-yloxymethyl)benzoate) | 86.74 | 154.20 | 154.90 | -68.16 |
| 2520 | 4-Nonyl-N-(4-pentoxyphenylmethylene)benzenamine | 48.52 | 117.00 | 116.70 | -68.18 |
| 2521 | 4,6-Dichloro-1,3-phenylene bis(4-(4-tetradecyloxyphenyliminomethyl)benzoate) | 106.86 | 172.90 | 175.10 | -68.24 |
| 2522 | N-(4-Pentyloxybenzylidene)-4-pentylaniline | 33.28 | 100.90 | 101.70 | -68.42 |
| 2523 | 1,12-Bis(4,4''-didecyloxy-p-terphenyl-2'-yl)-2,5,8,11-tetraoxadodecane | 236.20 | 178.40 | 167.50 | 68.70 |
| 2524 | N,N',N''-Tridodecanoyl-2,4,6-trimethyl-1,3,5-benzenetriamine | 63.71 | 124.60 | 132.60 | -68.89 |
| 2525 | 1,3-Phenylene bis(4-(4'-tetradecylbiphenyl-4-carbonyloxy)-2-fluorobenzoate) | 251.82 | 181.70 | 182.50 | 69.32 |
| 2526 | 4-Octylbenzoic acid | 44.52 | 112.60 | 114.60 | -70.08 |
| 2527 | 4-((5-(4-(3,4-Bis(dodecyloxy)benzoyloxy)phenyl)-3,4-dicyano-2-thienylethynyl)phenyl 3,4-bis(dodecyloxy)benzoate | 155.40 | 220.10 | 225.90 | -70.50 |
| 2528 | 1-(4-Dodecylbiphenyl-4'-yl)-3-(3-heptyloxyphenyl)propane-1,3-dione | 62.36 | 133.20 | 132.90 | -70.54 |
| 2529 | 4-Dodecyl-N-(4-octadecyloxyphenylmethylene)benzenamine | 254.58 | 181.60 | 184.00 | 70.58 |
| 2530 | 2-Decyloxy-6-(4-(4-dodecyloxyphenyl)buta-1,3-diynyl)naphthalene | 57.38 | 124.90 | 129.00 | -71.62 |
| 2531 | N-(2-Hydroxy-4-octadecyloxybenzylidene)-4'-dodecylphenylaniline | 238.63 | 167.00 | 167.00 | 71.63 |
| 2532 | 1,2,3,5,6,7-Hexaoctyloxy-9,10-anthraquinone | 118.35 | 185.40 | 190.00 | -71.65 |
| 2533 | N-(4-Butoxybenzylidene)-4-heptylaniline | 34.11 | 104.90 | 105.80 | -71.69 |
| 2534 | 2,5-Bis(4-butoxyphenyl)-thiazolo(5,4-d)thiazole | 54.50 | 127.10 | 126.20 | -71.70 |
| 2535 | 1-Ethylheptyl 4-(4-(4-nonyloxy-3-fluorobenzoyloxy)-3-fluorobenzoyloxy)-2-fluorobenzoate | 56.91 | 129.60 | 128.80 | -71.89 |
| 2536 | N,N'-Bis(4,4''-didecyloxy-p-terphenyl-2'-carbonyl)hydrazine | 82.08 | 154.40 |  | -72.32 |
| 2537 | Cholesteryl nonadecanoate | 207.30 | 135.60 | 134.90 | 72.40 |
| 2538 | 4-Decyloxybiphenyl-4'-yl 4-(2-dodecyloxypropanoyl-3-methoxybenzoate | 64.81 | 135.50 | 137.30 | -72.49 |
| 2539 | 1,4-Bis(4-(2-hydroxy-4-octyloxyphenyliminomethyl)phenyloxy)butane | 230.31 | 159.00 | 157.70 | 72.61 |
| 2540 | 4-Dodecyloxybenzylidene-4'-isopropylaniline | 37.01 | 108.50 | 109.70 | -72.69 |
| 2541 | 4-(4-(4-Octyloxyphenyliminomethyl)-3-(hydroxyphenoxy)butyl methacrylate | 180.03 | 110.60 | 107.30 | 72.73 |
| 2542 | Octakis(undecanoyloxy)-9,10-anthraquinone | 445.50 | 371.40 | 372.20 | 73.30 |
| 2543 | 4-(4-Bromophenyl)-2,6-bis(3,4,5-trioctyloxyphenyl)pyrimidine | 60.28 | 132.80 | 133.60 | -73.32 |
| 2544 | 1,9-Bis(4-(4'-hexyloxyphenylethynyl)phenoxy)nonane | 194.72 | 121.90 | 121.20 | 73.52 |
| 2545 | Di(4'-octyloxyphenyl)-cyclohexane-1,4-dicarboxylate | 203.70 | 129.80 | 130.00 | 73.70 |
| 2546 | 1,3-Phenylene bis(4-(2-fluoro-4-hexadecyloxybenzyoyloxy)-2-fluorobenzoate) | 287.21 | 213.50 | 213.40 | 73.81 |
| 2547 | N,N'-Didodecyl-3,4,9,10-perylenebiscarboximide | 132.31 | 78.90 | 58.40 | 73.91 |
| 2548 | 6-Decyloxy-2-naphthoic acid | 40.14 | 111.80 | 114.10 | -73.96 |
| 2549 | 4-Decyl-N-(4-pentadecyloxyphenylmethylene)benzenamine | 234.92 | 161.40 | 160.90 | 74.02 |
| 2550 | N,N'-Ditetradecylbenzene-1,2-dicarboxamide | 257.84 | 192.40 | 183.70 | 74.14 |
| 2551 | 4-Heptyl-4'-propoxyazobenzene | 31.01 | 104.50 | 105.40 | -74.39 |
| 2552 | 4-(4-Heptyloxybenzoyloxy)phenyl 3,4-dimethoxybenzoate | 29.35 | 98.40 | 103.90 | -74.55 |
| 2553 | 4-(4-(4-Octyloxybenzoyloxy)benzoyloxyphenyl)-6'-phenyl-2,2'-bipyridine | 108.35 | 36.20 | 33.50 | 74.85 |
| 2554 | 1-(6-(3,6,7,10,11-Pentakis(hexyloxy)triphenylen-2-yloxy)hexyloxy)-9,10-anthraquinone | 47.02 | 113.80 | 122.20 | -75.18 |
| 2555 | Tetrakis(4-(3,4,5-trisdodecyloxyphenyl)phenyl)ethene | 438.30 | 496.00 | 513.70 | -75.40 |
| 2556 | 4-(4-Cyanophenoxycarbonyl)phenyl 3-(4-(4-tridecyloxybenzoyloxy)benzoyloxy)benzoate | 211.63 | 135.00 | 135.80 | 75.83 |
| 2557 | N-(4-Nonyloxybenzylidene)-4-decylaniline | 212.07 | 137.20 | 136.10 | 75.97 |
| 2558 | 4-(4-Nonyloxybenzoyloxy)phenyl (3,4-dimethoxy)benzoate | 30.02 | 106.50 | 106.10 | -76.08 |
| 2559 | 1,7-Bis(4-(4'-hexyloxyphenylethynyl)phenoxy)heptane | 188.73 | 113.80 | 112.60 | 76.13 |
| 2560 | 1,9-Bis(4-(4'-decyloxyphenylethynyl)phenoxy)nonane | 230.03 | 154.20 | 153.30 | 76.73 |
| 2561 | N-(2-Hydroxy-4-nonyloxybenzylidene)-4'-dodecylphenylaniline | 207.96 | 130.60 | 131.10 | 76.86 |
| 2562 | 4-(3-Decyloxyphenyliminomethyl)phenyl 4-hexadecyloxybenzoate | 250.82 | 171.90 | 173.20 | 77.62 |
| 2563 | Bis(8-(4-(4-butyoxyphenyliminomethyl)phenoxy)octyl) malonate | 243.63 | 169.30 | 165.80 | 77.83 |
| 2564 | 4-(4'-Dodecyloxybenzoyloxy)benzylidene 4'-isopropylaniline | 37.50 | 113.70 | 115.70 | -78.20 |
| 2565 | 3-(4-(2-(4-Dodecyloxy-3-nitrophenyl)-1-ethenyl)benzoyloxy)phenyl 4-(2-(3-iodo-4-hexadecyloxyphenyl)-1-ethenyl)benzoate | 41.60 | 112.60 | 119.80 | -78.20 |
| 2566 | 6-Decyloxy-2-(4'-heptyloxyphenyliminomethyl)quinoline | 207.82 | 131.80 | 129.60 | 78.22 |
| 2567 | N,N'-Dinonanoyl-2,4-bis(nonanoyloxy)-1,3-benzenediamine | 89.71 | 164.00 | 168.20 | -78.49 |
| 2568 | 1,5-Diundecyloxy-2,3,6,7-tetraoctyloxy-9,10-anthraquinone | 135.50 | 209.60 | 214.20 | -78.70 |
| 2569 | N,N'-Dihexadecanoyl-2,4-bis(hexadecanoyloxy)-1,3-benzenediamine | 200.80 | 277.10 | 279.70 | -78.90 |
| 2570 | 2-(Perfluorohexyl)ethyl 4-(4-(allyloxy)benzoyloxy)thiobenzoate | 43.67 | 107.80 | 122.60 | -78.93 |
| 2571 | 1,9-Bis(4-octyloxyazobenzene-4'-carbonyloxy)nonane | 96.56 | 174.30 | 175.50 | -78.94 |
| 2572 | 1,3-Phenylene bis(4-(4'-hexadecylbiphenyl-4-carbonyloxy)-2-methylbenzoate) | 257.92 | 180.60 | 178.90 | 79.02 |
| 2573 | 4-(4-Octyloxybenzoyloxy)phenyl (3,4-dimethoxy)benzoate | 27.43 | 102.50 | 106.50 | -79.07 |
| 2574 | 4'-Undecyloxybiphenyl-4-yl 2-(1-methylpropyl)-1,3-dithioxoisoindole-5-carboxylate | 126.45 | 79.40 | 47.30 | 79.15 |
| 2575 | 2-Hexanoyl-1,4-phenylene bis(4-octyloxybenzoate) | 225.93 | 145.80 | 146.40 | 79.53 |
| 2576 | 4-(4-Decylphenylazo)phenyl 3-(4-(4-octyloxybenzoyloxy)phenylmethyleneamino)benzoate | 70.35 | 149.50 | 150.10 | -79.75 |
| 2577 | 3,4-Difluoro-2,5-bis(4-octyloxyphenylethynyl)thiophene | 118.61 | 177.90 | 199.00 | -80.39 |
| 2578 | 4-Chloro-1,3-phenylene bis(4-(4-dodecyloxyphenyliminomethyl)benzoate) | 83.02 | 164.00 | 163.90 | -80.88 |
| 2579 | 1,7-Bis(4-(2-hydroxy-4-octyloxyphenyliminomethyl)phenyloxy)heptane | 246.99 | 171.10 | 165.60 | 81.39 |
| 2580 | 4-Propyl-4'-methoxyazobenzene | 162.90 | 81.70 | 81.10 | 81.80 |
| 2581 | Octakis(dodecanoyloxy)-9,10-anthraquinone | 480.76 | 403.70 | 398.90 | 81.86 |
| 2582 | 1,8-Bis(4-(2-hydroxy-4-octyloxyphenyliminomethyl)phenyloxy)octane | 254.68 | 175.20 | 172.70 | 81.98 |
| 2583 | 1,3-Phenylene bis(4-(3-fluoro-4-tetradecyloxybenzyoyloxy)-2-fluorobenzoate) | 277.80 | 197.40 | 195.70 | 82.10 |
| 2584 | 3-(4-(2-(4-dodecyloxy-3-chlorophenyl)-1-ethenyl)benzoyloxomethyl)benzyl 4-(2-(3-chloro-4-dodecyloxyphenyl)-1-ethenyl)benzoate | 200.10 | 123.10 | 117.70 | 82.40 |
| 2585 | 1,3-Phenylene bis(4-(4'-octadecylbiphenyl-4-carbonyloxy)-2-methylbenzoate) | 280.82 | 196.70 | 198.10 | 82.72 |
| 2586 | 4-(4-Cyanophenoxycarbonyl)phenyl 3-(4-(4-hexadecyloxybenzoyloxy)benzoyloxy)benzoate | 228.91 | 147.10 | 145.90 | 83.01 |
| 2587 | 4-Octyloxybenzoic acid | 32.12 | 113.80 | 115.30 | -83.18 |
| 2588 | 2,5-Bis(4-nonyloxyphenyl)-thiazolo[5.4-d]thiazole | 249.74 | 167.50 | 166.50 | 83.24 |
| 2589 | 4-(4-Butoxybenzoyloxy)phenyl 3,4-dibutoxybenzoate | 26.16 | 107.80 | 109.40 | -83.24 |
| 2590 | 3-(4-(4-Cyanophenoxycarbonyl)phenoxycarbonyl)phenyl 3-fluoro-4-(4-tridecyloxybenzoyloxy)benzoate | 221.08 | 136.00 | 137.30 | 83.78 |
| 2591 | Isophthalydene bis(4-(4-nonyloxyphenyloxycarbonyl)aniline) | 230.40 | 147.00 | 146.50 | 83.90 |
| 2592 | 4-(4-Butoxybenzoyloxy)phenyl (3,4-dipentyloxy)benzoate | 33.72 | 115.90 | 117.70 | -83.98 |
| 2593 | Isophthalydene bis(4-(4-decyloxyphenyloxycarbonyl)aniline) | 238.23 | 155.10 | 154.20 | 84.03 |
| 2594 | Hexadecyl pentakis(4-methylphenylethynyl)phenyl ether | 128.33 | 49.00 | 44.30 | 84.03 |
| 2595 | Di(4'-decylphenyl)-cyclohexane-1,4-dicarboxylate | 226.61 | 143.60 | 142.40 | 84.21 |
| 2596 | 3,4-Difluoro-2,5-bis(4-hexadecyloxyphenylethynyl)thiophene | 309.78 | 242.60 | 224.90 | 84.88 |
| 2597 | 4-(4-Ethoxyphenylazo)-3,6-dimethylphenyl 4-decyloxybenzoate | 190.16 | 103.70 | 105.10 | 85.06 |
| 2598 | 4-(4-Tetradecyloxyphenyliminomethyl)benzoic acid | 55.36 | 140.20 | 140.50 | -85.14 |
| 2599 | 4-(4-Decyloxybenzoyloxy)phenyl 3,4-dimethoxybenzoate | 26.90 | 110.50 | 112.30 | -85.40 |
| 2600 | 3-(4-(4-Cyanophenoxycarbonyl)phenoxycarbonyl)phenyl 3-fluoro-4-(4-dodecyloxybenzoyloxy)benzoate | 215.27 | 132.00 | 129.50 | 85.77 |
| 2601 | Ethoxyethyl 4-(4-(4-octyloxybenzoyloxy)benzylideneamino)benzoate | 28.93 | 111.70 | 115.30 | -86.37 |
| 2602 | 2,5-Bis(4-dodecyloxyphenyl)-thiazolo[5.4-d]thiazole | 274.18 | 191.70 | 187.70 | 86.48 |
| 2603 | Terephthalylidene-bis(4-tetradecylaniline) | 227.30 | 138.00 | 140.20 | 87.10 |
| 2604 | Isophthalydene bis(4-(4-dodecyloxyphenyloxycarbonyl)aniline) | 257.89 | 171.20 | 170.70 | 87.19 |
| 2605 | Pyridine-2,6-diylbis(1,4-phenylene) bis(4-(5-hexylpyrimidin-2-yl)benzoate) | 112.40 | 31.10 | 25.10 | 87.30 |
| 2606 | 2-(4-(4'-dodecyloxybenzoyloxy)benzylideneamino)naphthalene | 29.42 | 115.70 | 117.00 | -87.58 |
| 2607 | 2,3,6,7-Tetrakis(decyloxy)-1,5-bis(2-hydroxyethoxy)-9,10-anthraquinone | 286.19 | 200.20 | 198.60 | 87.59 |
| 2608 | 4-(4-Isoamyloxyphenylazo)phenyl 4-dodecyloxybenzoate | 38.34 | 126.60 | 126.10 | -87.76 |
| 2609 | 4-Propanoyl-4'-nonanoyloxyazobenzene | 29.90 | 117.00 | 118.50 | -88.60 |
| 2610 | 3-(4-(4-Cyanophenoxycarbonyl)phenoxycarbonyl)phenyl 3-fluoro-4-(4-tetradecyloxybenzoyloxy)benzoate | 229.00 | 140.10 | 139.90 | 89.10 |
| 2611 | N-(4-Dodecyloxybenzylidene)-4-tetradecylaniline | 255.38 | 165.50 | 166.20 | 89.18 |
| 2612 | N,N'-Didecanoyl-2,4-bis(decanoyloxy)-1,3-benzenediamine | 92.20 | 180.10 | 181.60 | -89.40 |
| 2613 | 3-(4-(2-(4-Dodecyloxy-3-chlorophenyl)-1-ethenyl)benzoyloxy)-2-methylphenyl 4-(2-(3-fluoro-4-dodecyloxyphenyl)-1-ethenyl)benzoate | 42.90 | 129.70 | 132.50 | -89.60 |
| 2614 | 3-(4-(2-(4-Dodecyloxy-3-nitrophenyl)-1-ethenyl)benzoyloxomethyl)benzyl 4-(2-(3-chloro-4-dodecyloxyphenyl)-1-ethenyl)benzoate | 210.70 | 122.10 | 120.80 | 89.90 |
| 2615 | 3,4,5-Tris(octyloxy)benzamide | 139.83 | 64.50 | 49.80 | 90.03 |
| 2616 | N-(2-Hydroxy-4-undecyloxybenzylidene)-4'-dodecylphenylaniline | 227.05 | 138.70 | 136.90 | 90.15 |
| 2617 | 4-(4-Dodecyloxybenzoyloxy)phenyl (3,4-dimethoxy)benzoate | 32.40 | 118.60 | 122.70 | -90.30 |
| 2618 | (1-Butoxycarbonyl)ethyl 5-(3-fluoro-4-eicosyloxybenzoyloxyphenylethynyl)-thiophene-2-carboxylate | 63.24 | 152.60 | 153.70 | -90.46 |
| 2619 | 2,5,6,9,12,13-Hexaheptyloxydibenzo[fg,op]naphthacene | 213.09 | 130.10 | 122.60 | 90.49 |
| 2620 | 1,3-Bis(4-(4-(4-heptyloxybenzoyloxy)benzylideneamino)phenoxy)-2-propanol | 46.40 | 136.50 | 138.20 | -91.80 |
| 2621 | 4-(2-(1-Ethylpropoxy)-1-methyl-2-oxoethoxy)phenyl (4'-dodecyloxy)biphenyl-4-carboxylate | 193.21 | 102.20 | 101.20 | 92.01 |
| 2622 | 1,5-Diheptyloxy-2,3,6,7-tetraoctyloxy-9,10-anthraquinone | 90.50 | 177.30 | 182.70 | -92.20 |
| 2623 | 4-(4-Cyanophenoxycarbonyl)phenyl 3-(4-(4-pentadecyloxybenzoyloxy)benzoyloxy)benzoate | 234.03 | 143.10 | 141.40 | 92.63 |
| 2624 | 2-Methyl-1,3-phenylene bis(2-fluoro-4-(4-octadecylbenzoyloxy)benzoate) | 310.18 | 217.70 | 217.50 | 92.68 |
| 2625 | 1,7-Bis(4-(4'-decyloxyphenylethynyl)phenoxy)heptane | 239.43 | 146.20 | 146.60 | 92.83 |
| 2626 | Ethoxyethyl 4-(4-decyloxybenzoyloxybenzylideneamino)benzoate | 28.00 | 119.70 | 121.00 | -93.00 |
| 2627 | 1,3-Phenylene bis(4-(4'-hexadecylbiphenyl-4-carbonyloxy)-2-fluorobenzoate) | 289.94 | 197.80 | 196.80 | 93.14 |
| 2628 | 4-(4-Dodecyloxyphenylazoxy)phenyl 2-chloro-3-methylpentanoate | 17.60 | 111.20 | 111.20 | -93.60 |
| 2629 | 1,3-Phenylene bis(4-(3-fluoro-4-nonyloxybenzyoyloxy)-2-fluorobenzoate) | 248.08 | 157.00 | 154.10 | 93.98 |
| 2630 | N-(2-Hydroxy-4-dodecyloxybenzylidene)-4'-dodecylphenylaniline | 235.49 | 142.70 | 140.90 | 94.59 |
| 2631 | 4-(2-Propenyloxy)biphen-4'-yl 5-cyano-2-thiophenecarboxylate | 164.75 | 79.30 | 69.80 | 94.95 |
| 2632 | N,N'-Diundecanoyl-2,4-bis(undecanoyloxy)-1,3-benzenediamine | 103.20 | 196.30 | 198.30 | -95.10 |
| 2633 | 1,6-Bis(4-(4'-hexyloxyphenylethynyl)phenoxy)hexane | 204.50 | 109.80 | 109.00 | 95.50 |
| 2634 | 1,3-Phenylene bis(4-(2-fluoro-4-octadecyloxybenzyoyloxy)-2-fluorobenzoate) | 322.85 | 229.70 | 227.30 | 95.55 |
| 2635 | 1,9-Bis(4-(4-butoxybenzoyloxy)benzylideneamino)nonane | 51.87 | 145.30 | 148.00 | -96.13 |
| 2636 | 4,6-Dichloro-1,3-phenylene bis(4-(4-nonyloxyphenyliminomethyl)benzoate) | 37.90 | 132.50 | 134.30 | -96.40 |
| 2637 | 3,4,5-Tris(tetradecyloxy)benzamide | 56.46 | 137.20 | 152.90 | -96.44 |
| 2638 | N,N'-Dihexadecanoyl-2,5,6-trimethyl-4-hexadecanoyloxy-1,3-benzenediamine | 98.50 | 194.40 | 195.30 | -96.80 |
| 2639 | 4-(4-Octyloxybenzoyloxy)phenyl (3,4-dibutoxy)benzoate | 26.60 | 123.90 | 123.60 | -97.00 |
| 2640 | 6-(6-Heptenyloxy)-2-naphthoic acid | 198.20 | 102.10 | 100.70 | 97.50 |
| 2641 | 1,6-Bis(4-(2-hydroxy-4-octyloxyphenyliminomethyl)phenyloxy)hexane | 260.06 | 167.10 | 162.50 | 97.56 |
| 2642 | 4,4'-Dioctadecyloxyazoxybenzene | 301.62 | 207.80 | 204.00 | 97.62 |
| 2643 | N,N'-Ditetradecanoyl-2,4-bis(tetradecanoyloxy)-1,3-benzenediamine | 143.10 | 244.80 | 241.10 | -98.00 |
| 2644 | N-(4-Dodecyloxybenzylidene)-4-hexadecylaniline | 271.27 | 173.60 | 173.00 | 98.27 |
| 2645 | Cholesteryl 4-(4-(4-heptylphenylethynyl)phenoxy)butanoate | 188.20 | 89.20 | 89.60 | 98.60 |
| 2646 | 1,8-Bis(4-(4'-hexyloxyphenylethynyl)phenoxy)octane | 216.80 | 117.90 | 117.40 | 99.40 |
| 2647 | Isophthalydene bis(4-(4-octyloxyphenyloxycarbonyl)aniline) | 238.45 | 138.90 | 138.90 | 99.55 |
| 2648 | Di(4'-dodecyloxyphenyl)-cyclohexane-1,4-dicarboxylate | 260.50 | 162.10 | 160.70 | 99.80 |
| 2649 | 4-(7-Octenyloxy)biphen-4'-yl 5-cyano-2-thiophenecarboxylate | 196.20 | 99.50 | 96.20 | 100.00 |
| 2650 | 2-Dodecanoyl-1,4-phenylene bis(4-nonyloxybenzoate) | 277.50 | 178.10 | 177.50 | 100.00 |
| 2651 | 4-Propyl-4'-hexyloxyazobenzene | 200.50 | 100.50 | 99.70 | 100.80 |
| 2652 | 4-(4-Tetradecyloxybenzoyloxy)phenyl (3,4-dimethoxy)benzoate | 29.90 | 126.70 | 132.10 | -102.20 |
| 2653 | 4-(4-Butoxybenzoyloxy)phenyl (3,4-dihexyloxy)benzoate | 20.40 | 123.90 | 125.30 | -104.90 |
| 2654 | 2,4- Bis(3',4'-didecyloxyphenyl)-6-(3',4',5'-tridecyloxyphenyl)pyrimidine | 110.70 | 212.00 | 217.70 | -107.00 |
| 2655 | 4-Cyano-1,3-phenylene bis(4-(4-dodecylphenyliminomethyl)benzoate) | 56.20 | 160.70 | 163.80 | -107.60 |
| 2656 | 1-(6-(3,6,7,10,11-Pentakis(nonyloxy)triphenylen-2-yloxy)hexyloxy)-9,10-anthraquinone | 71.80 | 174.40 | 180.00 | -108.20 |
| 2657 | N,N'-Didodecanoyl-2,4-bis(dodecanoyloxy)-1,3-benzenediamine | 108.70 | 212.40 | 217.90 | -109.20 |
| 2658 | 1,3,5-Tris(3,4,5-tridodecyloxybenzoylamino)benzene | 476.47 | 374.00 | 366.80 | 109.67 |
| 2659 | Octakis(decanoyloxy)-9,10-anthraquinone | 440.50 | 339.10 | 330.40 | 110.10 |
| 2660 | Benzene hexa-n-octanoate | 347.60 | 245.40 | 237.50 | 110.10 |
| 2661 | Di-dodecyl 4,4'-azoxy-alpha-methylcinnamate | 238.20 | 143.10 | 126.60 | 111.60 |
| 2662 | 1,6-Bis(5-(5-(5-butyl-2-thienylvinyl)-2-thienylvinyl)-2-thienyl)hexane | 167.10 | 82.60 | 52.40 | 114.70 |
| 2663 | 4,4'-Dicyclopentylbiphenyl | 60.29 | 123.70 | 175.40 | -115.11 |
| 2664 | 1,6-Bis(4-(4'-decyloxyphenylethynyl)phenoxy)hexane | 258.60 | 142.10 | 142.40 | 116.20 |
